# Supplementary material for: Identification and Tissue Expression Profiles of Odorant Receptor Genes in the Green Peach Aphid Myzus persicae
Source: Insects. 2022 Apr 20;13(5):398. doi: 10.3390/insects13050398 (PMC9147661; doi:10.3390/insects13050398)
Supplement: Supplementary file 1 [file insects-13-00398-s001.zip › Supplementary tables and figures.pdf]

Table S1.

The gene-specific primers for *MperORs* cloning and sequencing.

Gene	Forward primers (5′-3′)	Reverse primers (5′-3′)
MperORco	ATGCAATGCCAGCCGTTGTTCA	TTATTTAAGCTGCACCAAAACCATG
MperOR2	ATGCAGAAGTCCGAGCGTTTCA	CTACTTGGCGTTAGCCGCCCTGAG
MperOR3	ATGAAAACGTCAGAGAATATTA	TTATGAAGAATTGTATAACACATTGAG
MperOR4	ATGACATTATTAATTGAAA	TTATGTTTTCAACAATACATTTAG
MperOR5	ATGCCGCACATAGACACCATCA	TTATGACAGCTTGTGAGGTTTTATAG
MperOR9	ATGGAGCACATAGTTGACATA	TTATTTAAATTGGCGATTGATTG
MperOR10	ATGGCGCACACAGTAGCTGACT	TCATATTGCAGATTCTTTAAATTG
MperOR17	ATGACGACGACGGGGGCGACTA	TCATTTTTGGACTCTTGAATTTAATAT
MperOR20	ATGCGGTCGTCGTCAGCGACGG	TTATGATCTTGAATTTAGTAATAC
MperOR21	ATGGAAAATCCCGAGAGAGTCG	TTAATTTAGTAACACGGATACTATCG
MperOR22	ATGAGACGTTCGAAAATAGCTT	TTAACTTAGTAATGTAGATACTATCG
MperOR23	ATGAATTTCAATGATGAGAAAA	TTATTTAGTATTAGTATTTCCATAAC
MperOR24	ATGGGATTGCAGAATGAGCATTC	CTATAATAGAATAACTTGAATCATTA
MperOR25	ATGGTTATCGGGACAATAATTGT	TCACGTCTTGTTTGCACACATCTTT
MperOR29	ATGGTATTGGAAAAAGAGCCCA	TTATGTTTGTTTTGCAAGCTGATC
MperOR35	ATGGACAAGTTGAGAGTCGAAG	TTAACTCGTGGTTTCCACTGCTTTCA
MperOR36	ATGGAAGATTTTAGAGATGAAGA	TTAATTATTTGAATCCATGCTTTTC
MperOR37	ATGAAATGGTTACAAGATCACGAAG	TTAATTTTTCCCTTTCATTGTTTTG
MperOR38	ATGAACTCATTGGAATCCAACGAAG	TTATAGTATGCTATCAAATATTACGCTA
MperOR39	ATGAATTTGGAGAATTTGTTC	TCATACATTTGCGAAAAATGGCCG
MperOR40	ATGAATCCTTCAGAGAAAAATTATGC	TTATTTACTTATTTTATGAATA
MperOR41	ATGAATAAACAGAAAATCTACGATG	TTAGTTAATATTGGAATTAATCAATACGG
MperOR42	ATGCTGAATTATTCCAAAGACGAG	TTATAGTCGATCTACTATCTTC
MperOR43	ATGGATTCAAAACAAGAAAAACAA	CTATTTCATTTTATTTTTATTATAATT
MperOR44	ATGTTCACTACAGAATTTTCGTATC	TTAAAACAATTTTGCTTTTTTAC
MperOR45	ATGAGTATTCTTCAAATCAATAAC	TTATTCTTTTTTCCAATTTATCAAC
MperOR47	ATGGACATTCGGAACGAAAACAACC	TTAATCATTCTTTGCATTTATATAATGT
MperOR51	ATGGATATTCGTAACGAGAAAAAC	TTATTCAACTTTTCTTTGGATATAAT
MperOR53	ATGGACATTTGGGACCAGAAGAACC	TTATTCATTCTTTGCTTTTATAC
MperOR64	ATGGATGTTCGTAACGAGAAAAAC	TTATTCAACTTTTCTTTCAATGTA
MperOR67	ATGGACATTCAGAACGAGAAGAACC	TTATTTAACATTTCTATAGATACA
MperOR69	ATTCTTCTCGTTTCGAATATCCAT	CTAGTCTTCAACTATTTCAAGAGTT
MperOR78	ATGGACATTCAGAACGAGAAGAACC	TTATTTAACATTTCTATAGATAC

Table S2.

Primers used in RT-qPCR for determination expression levels of *MperORs*.

Gene	Forward primer (5′-3′)	Reverse primer (5′-3′)	PCR efficiency
MperORco	CTGGATCACTACAATAATAA	TAGAGTTCTGTAGAACGACT	1.92
MperOR2	CGCACGTCTTACGGAGAC	CAGATATCATTTGATTGACG	1.91
MperOR3	CCTCCTGCACGCTGGCACTG	GGGTGGTCGTCATACGCGTCC	1.92
MperOR4	GGATAACCGAGCACATTTCG	CATTTCAAACTGGTTAGTTA	1.94
MperOR5	CCGCGAGATGCTCCAGTAC	TGCCGGTCTGTATCGGTAATAC	1.95
MperOR9	CATCAGTCACAATTTCGCGA	TGTGCAAAGTACACGGGCAA	1.92
MperOR10	GACGTGTCTGTCGATCGTCTAC	TGTGGTATGTGCGGTATTCT	1.93
MperOR17	CGACGGTGACTAACGCCTTTC	ACGATAAACGTGCGGAGA	1.94
MperOR20	GGGTTACGTGCTGGTGACCA	ACACGACCAGCGTGCCATAGC	1.93
MperOR21	GCAGCCAACGCGGACCGG	GAACCACGGCATTACCACCCA	1.94
MperOR22	CACGTTCGTCGGGCTCAGC	CACGAAACACATGGTGACC	1.90
MperOR23	ACCAGTGACATATGTTCAAC	TAACTGATACTCATTGCTGTC	1.93
MperOR24	GAACACCAACTGAAGACTA	TTGAAATGATTCAGCTGCTAG	1.92
MperOR25	CACGATAGACGCATACTCGA	TACGTGTTCCCGGCAACCGATC	1.95
MperOR29	ATGCTTGAAGTTGGCCGAAA	TGTACTATGAATCCCAACAATC	1.94
MperOR35	CTCCCAAATAGTGTATTATGA	ACAGGATGTGATTCCTTTGAC	1.95
MperOR36	CTGTTGCGTTATAATTGCTCA	AACCACGATCAACACATATGC	1.94
MperOR37	ATCATGTGGTGTGGATTATAGT	TTATATCCATCTTCGTCCAAT	1.96
MperOR38	TGCTACGATGTTTATGGAG	CTCATATTGAGCTATTATGACA	1.91
MperOR39	TGCTGTCGTGCGACCCATTAC	TGTATAATCCAAAGTTCACATC	1.92
MperOR40	GGATTGTCAAACAATTTATAAG	TTGAGCTGTTCACTCGCATTG	1.92
MperOR41	CGTCCAATCGGTTTGATTCAG	CTACAATACATGTATGACTGCAC	1.91
MperOR42	GACACTGTCGTAGTAGGACTG	AGTGATATTGTAGACGTTAGTA	1.92
MperOR43	TGACGGTTCATACAGCAAATAC	ATGCCGTGTTTCTCATGCAGTG	1.94
MperOR44	GAATCGGTTGGACATGAATCC	ACGTTATCACAGCCATAGATTC	1.95
MperOR45	TAGGCACTATGGCAGTGACA	TACATTTCATGTCCATTTCTG	1.92
MperOR47	GTCGAGACGTTGTTCGTTGC	CATGGGTGTTATCAACTGCATT	1.93
MperOR51	GGATCATCAAGCAGTTATGG	TTGAATCTTGTTGACTGTGT	1.94
MperOR53	CGGTCTATGACCCAACCATTG	CCAAACCATGATGAGCGATAG	1.93
MperOR64	AGACGAGTGATGTTGTTAC	TCGGTCCAATTACTACTGTACA	1.94
MperOR67	CATGTTTAGACTAGTGATAC	CTGCTATACAACCCGAATATTA	1.90
MperOR69	CATTGTACAGCAGTAATTG	GGTAACGAAGAAATGTCCAT	1.93
MperOR78	ACTGGACCGTTGGCGAG	GCGATGAAATAGAAATTCATG	1.94

Table S3.

The protein names and sequences of 606 ORs from 7 species of insects used in Figure S3.

Protein name	Protein sequence
AlucOR1	AAAAEASDMGHLLTPLHWTGVLRHPRYSHQSPLLFRLYTAAVSSFALCFICSEAAALVHDDTGDMDVIILLISTINTASIWIIRMVHIAVFERDFHKLAVQVGHDFAEFLTWDDIPLLTSQSRVVRRFSKLYMWGGVGACAYFLVSPVCPEGLPYILALPFDAMQPLGFAVTWVFCSVVTLHAVVMTMVLDSFNVSVIAQLRIQLKLLSTKIVNLSKEILNTDVDSSEANVYQELYYRLEKCIRHHEAIIKNADLLERSLGTMLLAQSISIGASTCFQMFQLATRANGLQQAGKFGCYLFAMLAELFVYCWFGDDLITESENVALAAYEAVTSLQGCPLSMKKSLLLVMHRAQRPLRVTAGGFFPLSRESFVSVVNVSYSFFAILRNFKMEEQ
AlucOR10	AARGVTSYYVGVGGAAVVVWNLMPLLLEAGRSLPTIAWYPYDETKTPFFEVTYLLQGISTFYCCITNVGLNVFLVSLMIYISDELKNLNDSISSINYRSIYNCCCNGDKQIGLLNWSSKIVTDDCDINVDTHIAHSKHIRQRAEFCCVVKAQDYLRMCLQYHQELIRTVKKLETTITAIVFIEFVAGIIVTCLTLFQAAVNAGNMALFVKFIMYLLYMTVGMFIYCWYGQDLMQKSEDIKWAAYSCNWQGAPKSFTDLLKIVILWAQQPLTLSAGKYYKISLKTFVTLLNASYSYYAVLRQMSDTQK
AlucOR100	AEFEVLSDNFAALHKVHEQSEKGKIRGTVYERQIKEESPPASCAHIYIECVSDEQMYRQLTQNVRHHQALLRSISLLQTAMSAPIYLVLFVNMVNLCTNLFIATLLLQRDGSFSKALAVLLTIPTLLSQTAIYCLFGHALTEKSEKLTQSAFSSGWPECDVRFRRGLLMVMTLAEQPAEVTVGKMTKLSKQTLLQVLNGTYGLLNMFYQLHSQM
AlucOR101	AFMMPLWLPLDTQASPTYELLLGVQVPCCWICSETSVLLDCAMLALMLQAAAELAVLNDRLSAVGPGQRRAADDKRDLQANDHMFSGLVDNINHHQIIITYMHLLETLLSRGISVLLICNTISICFHIVATVALLQEDIEPVGMTKMVLGSTLYAYQTAILCLLGQRITTQSERLPVSAFSCDWPSADGRFRKLLMVFCLRSSQALIIRVCGLYSLSRETLLQVLKAAYTLFNFVYQTVGEEEPLN
AlucOR103	AGLGSQLACLTAAKAMKDHVQSDGQVRDTTQEVEELLGFSAVLMRWMGLWQPPGGAPAGPKLLIALSFDAMFFVIVAGGFASVVLDPPPMDSVLEVVLTLASSVTWGVRNVAILVRQNRLQNLILDVLDMKKRFAENGTEFRKAFQRRAKIRTALLVGIPMLGIPMWLVEPAFSKTIVATSENTTVVVRKTPLVMWMPMDTQTHPNYEITYAFQMLLISIVVDANVIIDIFFSCLLITVTADIAVLNNNVANMRLCKDDGFTKESGKEVNLSATWETTYKSKKSLEEKDQLYDGNTDVYASHTTDPSTQLYRTLVKNIQHHQVLMSIVNDLESIMSESSVLMLVVNSINICTQGIGFVDGFRPGSNKTTLMKRFLTFPAYVNQTAHFCWYGQDIIDQSERLLESAFNCDWANADSRFCSTLRIFMLQTSRPLKMQIGKIFTLSRNMFLQILNTSYTIFNMFINF
AlucOR105	ATARYTDRDTATPVYELLCVLQYFSMQYSHFAAMCIDLFFACLIIHVAAQLAVLNVRIGQIREEYYRGGAEEAVPPEERRAREDEAWQQLRECVEHHKLAIKLVGDLEDLANVIILSQFMGATIIICVTLFLITTSEQHFAALVKLQGYLIVVVYEIFMYCWFGDDVMYQNSRLATSVYTCGWPGAPQKLQRALLIILMRSQRPLGVTAGKFYRVTRETFVSLMKASYSYYALLNQMNK
AlucOR106	ATSAGKLCLDTPQELAAVADYGYAVFHLSAVTVKVACFILQRSTIEELVNLLDETRKTYGKTEANYQVRQLYQRRATNIYRVLQALAVAVLCMWISSPVIQRQGLKEGERPPPNPIWMPDNSPGYEIVYSVQSLCGSAAVQASMLIDTSFYKLTLMVTAELQILNDNLARLGRAAEAADRKGTAAKQDGKEVAVPAIKQTSAPVTEDNDQLLNDQMVDNVRHHQAIIKCFDLLQSVITYSVSIVLLTNILTVCFSIFVVYVLLQSDGGLKSASKTIIGIPSVLGETGMFCIFGQMVINQSERLRSSAYSCGWPDADGRFKRALLILVLRTSQPLQFTVGKLIHLSNETFLQILNCSYTLINVLYQFQGSKE
AlucOR107	ATSRTCLVAGCYLALVAFAGSQLLAEPWRPLEELAIAALIFVTSASFTFKFAMFLRYRPRLLKLATLLCGGGLRPAEGEETRRRYRERGRQVFLYLQAILSVPTLLWAMQPLLVPPGGSAANLRNATAPVRMRSTPLPMWLPPALQQSPAYELVYALQVVSMLIVLQTSVFTGVFFLVLMLSIAAELHVLNDSISGNRSKPSSSSGRRCSSLSMEIHCPSNGRLVNDSSVINFEGRLFRQQMYCDLVKNIRHHQLIIMCVKELEAIMNLPIFVLLFLHMVNICAQIFVTSLLL

	QKDNDSTTMFKVLFTLPIYLYETGLYCVFGQIIIDQSDRLPTSAYSGAWLQGDARFRRALLLLTSRASRPLTLTVGKTYTLSRHTFLQILNGSYSLFNMLYQVQGNK
AlucOR109	AYAGCVVSALLFLVASQLSAMLHFWGDLLSATNNACVTSSYSMAVFKFFAFRAMRPSVEHLVRDLDRCLQVYGREYAAEKEAGFGACERQARLVSRLQVSMGVSVYLSWVVLPAIRAQACRSTDCRVHAGFPALVWYPFAFTEPPVYPVIYAVLSIGLFYGCIIFTSQDGFFWSLIIYVGAHLRFLNFMVANMSSSEADAKIPRGSPEMEENMRSRLKECVRYHNDIDRCVQRLSSLLGPVMLGQFLTDIVTISASAFVATMLKADSGWLLKYGSYLSGTIEHMFLFCWFGNDILTESARLQLSAY
AlucOR11	CEMAVLGADWETRMLTHGAPCLDLTVYLQPILKPLAATGMWSSSIYSNSVHKRAARAAGLSLLALFLAELCAELVGTPLVVARGGGVDRLIHHLSVLGIHLDSFWKWLFMLTQRRRLTCLLRLLQRCFQLGVLASPTHHLPLRNTYIQKPQQSLDPQPQAMPAQLSEVLGKTRMRSWLVTVVWTVSCVLGASHWFFVPLLKGDTTLRFDALYPFDSQQPTTKQTVVYWLQYVCSIYSLLLLCFFDCLLVWLQQLLCVQLRCLATNLRELSPNDEHRRKLAICVSHHEHILRAMRELNAFVAPLLFLQCFKNMIVLCVVAFLASVAGVNDLLELSSLVLYFMAACQQLFFYCWCCEELTHLGMEVCDAAYDSGWENWDVSSQKSIIIIMWRAQKPFFFRGGWFYTLNVATFVDLIRLSFSYYTVLRSMREG
AlucOR12	CSATSVSSCRRATCRVVNADGLPFVLALPYDATRPLAFAATWLFSVYIVFCVHIGTMAADSFNVTVILQLHNQLDLLGRNLRSLKDSVSHVKSASTETLPKRMRHSDNSRDIHSRLRKSVLHHQAIIRNVQLLEECLGGMLLGQSLSIGTSFCLQLFQAATRAKRVQELGKTCSYLVTAFSMLFIYCWFGDDLISESEKLAFSAYDAVTSLQSSPASIKRSLLLLMVRAQRPLQLTAGGFFSLSRESFVAVLNASYSFFAILRNFKEEYD
AlucOR13	DLDHLLRPLHWTATLRHPQSAHSSPLLFRLCKLVIITIIFSFFCSEATVLFRVGAGDLDVFTITIGVADTNIIWFCRMVHISVCERAFHKLALQVGQDFAEFLSWEDLPLLRAQSGAVRRFTRLYVWFGVGCVVYYLFSPASDAGLPVILALPYDMHRPLAYALTWLYVSVTTFHVVVMTMVFDSFNVSLMAQLRTQLSLLSRKVVSLAKEMSEKPVHSPETSAYRELHSRLEKCVRHHQAIINNSDLLERSIGPMLLAQCLAIGACACFQMFQVATNTNGLQETGKYGAHLVVMLAELFEYCWFGEGLITESENMALAAYDAVTSLQDCPISIKRSLLLMLQRAQRPLRITAGGFFPLSRESFVSVVNVSYSFFAILRNFKNEEE
AlucOR14	EGLPLQLVLPFDTSRPAGWAAGWLFCAFLTVHCVVMNMMADAFNVSLIAQLRMQLMLLRRKIVRIAEEGSSRSLDSLHTTDYRDLHYRLHKCVLHHQAIIRNADLLESCLGAMLLGQSISIGTVACFQMFQVAMSANGLQQVGKFGCYLFAMLAELFIYCWFGDDLITESENVALAAYEAVTSLQGCPLSMKKSLLLVMHRAQRPLRVTAGGFFPLSRESFVSVVNVSYSFFAILRNFKMEDQ
AlucOR16	EGLPLQLVLPFDTSRPAGWAAGWLFCAFLTVHCVVMNMMADAFNVSLMAQLRTQLILLTRHLTDLAGDVQSEGPSLPQTPRKVVEQPVKFYTPVYRHYSLEGTSSHYQKTNPSVVDAEVQRSQHSYEPSNSVDYRLQKIILHHQTIIRNVDRLQQCLGGVLLAQSLSLGSAICMLLFQVALSAQGAQETGKICGYLCAMFTELSVYCWFGDQLMSESEKVAFAAYDAVTSLQECPISMKRSLLLMMHRAQRPLCITAAGFYPLSREAFVSILNVSYSFFAILRNFKEE
AlucOR17	ELQVLNDNLASVKAAPLLRSSPYSGWNRATNVTNESWFHSGVSSPTGAIACNSYQSSGDRSESTTSITGYTNRTAVEMYRALANSIKHHQAIIRCVEELESAMTYSIFVLLFLNMMNICVHIFVTSVLLQKEVERTTMSKMLCTLPIYMYETGLYCVFGQTIIDQHGCDVLQSEQLTASAFSGDWPEGDARMRKALLLLMLRASRPLQLTVGKMYVLSRHTFLQILNGSYTLFNMLYQVQKNK
AlucOR18	ELSYAVQCVAGLYLSQISFSMDCLFAAVMILVAAQLKIVSCRILKLKAEESGEQGGGSGDVGGGVALENEQKPYENLCQCIESHQQILRFIIRLQNAMSPIAMTQFFFSVLVACMALFQATYSKDFTAVFRCVAFMPIPCGQVLLYCLAAHDVTEQAEAVSQAAYSCSWVEADRRFKRALRLVMCRAQRPLVLTAGRLYPIDRGAFLTLVNASYSYYALLGHINRRSMQDQL
AlucOR2	EPADDLLDLTWTVFILFGGAIVFVKMVCFVHQRAVMQEAFQLLLACRNCHYGGDSIRTIRGSYQRLGNITYFSIQVMVIVAWMIIVFMPLLNHRISAAGREPTAKEHGPLPIWLPFEVHNSPFYEFTYAFQALWLAFVAETSICVDCIFVNLMLMITAEIHILNSTLSTLQEHSIINKVPVVTTNQSTEDILPFTHSSRIHDSKQLFEKLISIEGTDYSPSDMCDISCAESQKIIKQQIYHQLLQNVQHHQTVIKCTSSAQKAMNFSVFVLLSTNIIEICSSIFGTVELLKNDMPA

	AAMKTLCVIPIILSQSGMYCFFGQMISDESEKLLQSAYNCDWYEGDIHFQRVLFILMLRATGPLKLKVGKTMS<br>LSRQTFLQVLNGAYALLNMAYHVSK
AlucOR21	ERRPRLYALYTASVLASQAACIAMGLRHALDSWPDTDAVMLTFVNTATLLGGVAKLAHLCAHVRDYRRLV<br>AALRGLVAAQWPACRRDPRLLAAFRRSYRRALRLTFGMIAYLHFIGPIWYAMPLVARATGGEQRQLPFVDL<br>RGAVKEDLSLYVSVYLLQCHAIFFWCFVSPSLDMFFVTCMLHVAAQLGILNARLSELGGVRVPDGGEMVAL<br>PAGRRKSRNLEQHSDDSDISEELRDCVKIHQDILSFLQDMQRVMSKVAMAQFVCSSVSICITLFQATCNPEG<br>NSNLKCFMYLPMPAFQIFIYCYGGHELIDQGLAVSLAAYSCAWVGATRRVTSSLHIMMCRAQKPLTLTAGKL<br>YPINRITFVSLLNASYSFYALLRQTRDR
AlucOR22	GAGGDSPRLLPLPAWLPLDLQASPTYELVYAAQVLLIPLSTTSVCSDFVFIGLMLRISAELEILNDSISGLHKIR<br>KDVPTAPKNEAKSIGRVSDGEINLHISRNVKHHQAILKSVALLEEAMSTAIFILFLGTMIAISINIFAATAVLQT<br>VGGMTKALKMITAIPPIMFEVGLYCIFGQIVTDQSEKLMHSAYSCGWVDCDPRFRHSLLTFCVGSRRPLEITV<br>GTVYKLSKETFLQVLNASYAMFNMLYGFQSNT
AlucOR23	GGSALAVHAYYCTVLLANTAKTLVFAACRHSLGRAIHVLSRCPRAERSEKSGASARLTFALPQVMVLLAVAT<br>HSLVPLLGAGDGACADPEATGQGGGGCFSGRFPLELWYPAAALATPLYQVVYALQLVAIYCTCHTAINVDL<br>FFFAVTNHASSHLQELNDALCRMGVRHTPVNRRRGSDESDHSADGKHGNGPLRHTSEESLVRAEHQRARY<br>QELVRLIRVHQTITRAIKELEPVISYALFGPILTNVLNICLHMLVLTTERDNMGTNSKAFVGILFNLIQNGLYCS<br>FGETLTHQSDRLFISIYSSGWENGCRRFKKAAAILMFQTRKPVQIKVAKLYTLSRRTFLQLLNNSYGLFNLLYQ<br>VKNPE
AlucOR24	GPALHPGAALRLVHAFGLRRHVALLHGGHRARRGDDHGVGRLQRQPHRSVAHAAHVAQHRKLVTIAND<br>SSMRPLDSLRTTDYRELHCKLRKCVLHHQTIIRNADLLERCLSGILLGQSISIGSVACFQMFQVALILLIYNWN<br>HRTSLKKLSFVIGKFGFYLVAMLAELFIYCWFGDDLITESENLALAAYDAVTSLQGCPMSMKKSLLLVMHRA<br>QRPLRITAGGFFPLSRESFVAVVNMSYSFFAILRNFKDQKV
AlucOR25	HDWSGDGGSFYWPSYAQQCCSAFWLGKISVVLDCQFAAIMVLVTAQLEILSARLANLRPDGRALRESPNCK<br>VKLSYVDHDSEMYDELRRAIQSHQEILSFVSCLQQVMSPLAMTQFVCSVIVICVVLFQATYSQDFATVLKCVA<br>FLPVPCGQVFIYCWAADNMTEQAKEVSSAAYRCCWVDAGPRFKRCLLLVIRRAQRRLVLNAGHLNIDRAA<br>FLSLVNASYSFYTLLAQMNRS
AlucOR26	KLSLSTLRPRRYRRLMDHVTRRLAAHLRHDRAGRLRLRRDSRAAHAFTMGFVVCGHVTVASWSLLPLLLK<br>PADKLRLPLVAWTPFDSSHGTGFLVTYIYQFTCTLFMAWTSGATDLICVNVVMQLCSHLDILCSHLERVGRR<br>CCREGDGGRCAHGHLDDGDQRPAPGRDDLCGQLRGCIRYHQDIIRCRVAREMDSMLWTIVLSQCLCGMG<br>VLCLLLFQTAMYTLTIETIAKYLSYMASILLQIFCYCWFGDNLSSKSTAVARTAYNCDWTRGSSAFGRSLCILM<br>ARAHRPLTVTGGSFYVLSREAFIRILNASYSYFAVLYTMSDE
AlucOR27	LASRLFSSDDVVEETSSPLKTPLPTWTPAAAQRSPAYEALYLFEALCLTASSQALLCIDVFFIDLMLLVAAELRV<br>LNDNVAAVSAGAARSDSREDTAGHGSGVSTVQQRSREFPDTFSAFDVLVDRRMSEDMYRQLVGNIRHHQM<br>IIECVELLQMTMTYSIFALLFFNMTSICLNIFVTASLLQSDADLVTAMKAVFTTPVFLYESAMYCIFGQMIIDQS<br>EQLPLSAFNCGWPETHTRLQRALLVFMLRSSQPLRIQVGKTYELSKETFVRVLNGSYALFNMLYTFQGNK
AlucOR28	LLDISSPLDGLVGGRTRQALLLLDQAAVAFNHLCSVAAFTTMFVHFVVIACRHLQRSIDDLTADNCDIAAVV<br>RHHQQILRFIREIEEAYCILMFWLFLPMMVVMCLIMFAFLTMTSLDIEFLEMLAFFLIYCVTNGVISICGSMLTS<br>KAERVMVAAYSSAWPERSRGFSGAVRVVMVRFLQPAQLTVGKFVPLSINTFSKLLQESFSYLMVMLSLVNEK<br>DSEAQPGVVVEATANHSAYH
AlucOR29	LTALCLIWALSPVVVHPGQRWLPFNHFPLEPAPLPLYYELSYAVQSASSLLYIQVSFGVDFFFTVVMILITEQLM<br>ILNARLAQLHLYAGGGKSRAAATRVMTTAATEDRDEMYEELCHCIDTHKDIMRLISFLDSVMNPIVLTQFTL<br>SVMAACLTLYQQTYSPDGNSVMKSASYLPTPGIQVFVYCWGAHSIMEQGEAVSAAA
AlucOR3	MAAAAPESSGGDVEYLLRLLHWSGTLRHPRAGRGASLAFYARNAAVATAVLLFVLSQAAVILGEGPADLDR<br>FTRALCFFNTSLTWLLRLAHVALREKQFHAIALQVGSDFGEFLTPRDAETLGRRGRALRRFVLAYLCFGVAA

	GAGWDVFPVVRHGVCGDGLPFHMALPYRVDRPLPFAATWFYCFCMTMHVAVVTMVFDSFNVSLMAQLR<br>QQLSVLSGNIRSLADEQRRTASSGVDSPEHTERVRYRIRTIVRHHQAIIRNVESLEKCLGDMLLGQSLSIGASIC<br>FQLFQSAESLAEAGKFGCYLSVMLAQLFVYCWFGDDLITESEKVSAAVYSVVPSLQGCPTSVKRSLLLVMRR<br>AQRPLRLTAGGFFDLSRESFVQVLNVSYSFFAILRNFKEE
AlucOR30	MAAGELPEPLVDLRLPCGVLRAVGLWRTGEGGLFYSAYTVWCLLMLVCSVLGQLAGLQGHWANLPTVSTS<br>VCLTLTTSCTIFKALVFLRQRRRVDALVSRVERSVSAFWPLPPARRAAMLLEARRSALLMFSIFFGIGAVALSLF<br>YAGPALQNIKDRELIDSAPDNATLDRHLGRNLPMLIWWYGGQPVEAPYYQLAYALICYWFMLIYLSTSTLD<br>AFYVTFIIFLSSQLKMLNAALADAVQSAGLSDELGNRSGGQPTGAAAGRRADYRRLVHCILFHQQIIQSVHE<br>MESLLSPSVLTQFATSTLVICFTAFAVTTSTKKQEMPAYATYLATMFYELFMYCWYGNELLEQSDRLRLSAYSS<br>AWPDAGGHFQHSLCIVLSRAQRPVCLTAAKLYKLSRETFLVLLKGSYTYFALLHQMNDRQYNA
AlucOR31	MAASAAAAAAPEESVGSAAQSACDLGYLLTFLHWTGTMRHPRAGRRASRAYAAANAAVTLAFVYFVCSQ<br>VVVLFRAGTADLDNFTLTLSLIDTQGTWLLRIRHIAAMEPHFHRLAYQVGRDFGQFASAEDVRVLREGSRR<br>MRTVMLLYLAFGLAECCVWLTAPASETGLPFVLALPYDVTRPAAYVATAVYCCFITLHTIMANFAADAFNAS<br>LIVQLRMQLALLNRNIVNVNRIVEQERSSPYKSESADALRPYKSYSTSDVNERLRKNILHHQAIIRNVQLLQS<br>CLGSVLLGQSLSIGISVCFQLYQVAKSAESLQDAGKYSSYLFTMFAELFVYCWFADDLISESENVAQAAYEAVP<br>SLLECPTPVKRSLLILMQRAQRPLTITAAGLFPLSRESFVSIVNVSYSFFAILRNFKED
AlucOR32	MAATERRQGEVGEDEVLRSNVRLLQLAGAWPPTAPRGLGRLFPLHLASVYLSQLANIAMALRLMQTARGD<br>MHEITQALMNAMTLVGGILKLLHLSTHVPAYRRLVLALRDVIRIQRHQCERDPHVASLLARAHRKALRLTF<br>WPIAYLNMLALGWYSIPVVFWALGWEKRLLPFFTLHGVDSYDFVLYAAIYFVQCHAIFYWCFISIGQDMFFV<br>TCMVHVAAQLQILNARLSNLGGGQGASNELGRLYSCRAEEDGPCERKPISDTMYTELRNCIKTHQEILKFV<br>QLLQQVMSPVAMAQFLCSVGAACVTLYQATFNPEGNSSLKCLMYLPIPAFQIFVYCWGGHELMENEENIGS<br>VRACRSSASQWLFAGRRVTSSLRTLMCRAQRPLLLHAGKLYPVSRDTFLSLINASFSFYTVLRHMNNR
AlucOR34	MACTFSLPTGCTTRGLTAAPCGADGVGGAFAGGPQRVGAAAAGAVAALVSAASVVQLAVDTPTDPETLRD<br>VLFTSTCGLAWASRMVLFMKQGVRLQRLVTTLLVTRKRYAEEFPGIRNSYDRAAAVIFYAWQVLPLTAVSLW<br>ALGPVTGAPLAVASGNYTVLERREPLVMWLPVDTQRSPTYELVFAMEMVGVYAVAEISILLDIFLVCLMILVT<br>AEVAVLNRNVSSTRLSRLDQDQPGGVTAIGREGYGGLSADGEWSAAANQTLPNSTLLPYQDTDAAKRRLY<br>DCLKRNIQHHQTIMTRREQRTAFYYLGSLPASHKSVNLFHNLVSSLAGIRFLKYAAIKLVLRPELQMLVFGVL<br>VTIIKTALFCLIGQSLTDNSERLLDSAFSCGWPTADRRFCSALLIFMQQASQPLSIRVGKIVTLSRNSFLQVMN<br>VSYTIFNMLLNTQ
AlucOR35	MADSKDPFIMLRRIFIDVNSYKITKLCDVSVVTFHSLVLCLQLYYMIANFDVNVLIINGPPTIVFLLMTVSAVLS<br>EAMAKDIFKGITFFQQIRWSLDVIEKNARIKLERKCQTINICITCILLFLSTTMVINMPFLGNPRQFFISIQIFEEY<br>FGKWSVLLNVLYFTGLPYLGYHAVKLCFAFVYAILEIELQFSLIEEYLFQMYEVDYLKSCKYLQDARYQQEIG<br>NSLRRCIIHHIALKKMVKMLVEVVLKCMPFYLVLGVLLLITCFAFIINFADTTTSTIKIQIFMFVASTLCITVLFC<br>WNGQQLKDVTSNIFFTLGGAPWYFWNLENIKILLMFITNCTKNDSIVLRTSVSYALVLFNLRKRSLV
AlucOR36	MAEDDYTLTDMAGVYLLPHSRAYMFWTGHWVGAVPGPTPVPIMVARALGGTLTYVAVFVVCWGSLNGIM<br>HGSGTNDMPMNLIIISCSISSVHKYCVYTNQEQGLGRLGRWMKRVSNREKKNKIPKTITDHILKVCLLIFYYS<br>GTLAAFMLLTKLALTGTTYNVLVPGLENVLLLKLAIALSFMSLGFEVVVDALILMNSLFTFRRELMRSFEEWR<br>KLNFDSENPNQYKEELKERVQKHIELLTIFQDLREFNNSMFGYQVFAIVFTTCALLYGMAKETENSHKVFVQ<br>TMPTATASFLEFFILCWCGEDIKFGFEQIHRSIYDTNWYEASLEDKKSMTIVLEFSKNPIILTGFTVFKANLETF<br>VESMRQSFSLYTILSEMV
AlucOR37	MAEFIDPFLMLRALVSVKFNDYTSLKLCNILLITIYSLIHCLLIHYMFKNLDINLAVRYVPMIMFLTLVIVGAIFS<br>VAIEKDILEAQVFLFKANWSLEMIRKDAQLKLERKCRIINICILCVLLLIFATITINAPLFGSQRELFICIQVFEEY<br>FGKWSFIPYYFYFAAFPFLYYDFLKLWMSFVYAVLEVQLQLTLVEEYLFETYQINSSKEWKNLQDTHYQQQIK<br>KSLRLCITHHIALKKFVKMTVDLTIKVMPFYLTIGVLILISFFSFIINFADSMSNILKIRIFMFSASIVSITVLLSWIG

	QQLVDVTSGIFWSLVGAPWYFWNLENVKTLLIFLMNCTKNESIVLAGICIDYSLGISILRLSVSYALGLFNLRK<br>SSLD
AlucOR39	MAEFKDPFIMLRTIVFINMNSYKVLKVCNILLIVLYSLIHCLLIYYMFKNVNINLVIRYSPAMLLSILAIIGAVFS<br>VFKEKDVLEIDKVFHKTRWSLNMITEDAQNMLKRKCLIVNACILFLLLVIVAMFIINAPCFGNQREIFICIQVF<br>EEYFGKWSSIPYYFYFFGYPFLYYNFFKGWMAFVYAILETQLQFLMLEAYLCEIYHTDNVKNWKYLHDTHY<br>QEEIGKSLRLCIAHHNVLKKMFKMVVNITDTAMPFFLLLGSLILISAFAFIINFVDTMTTILKIRIFISAIVMVSIT<br>MLLSWIGQQLINVTSDIFFTLGGAPWYYWNLKNVKILLIFLTNCTKNESIILAGICLDYQMCISLFQLAVSYAL<br>VLLNLRKSSLV
AlucOR4	MAEVRVDSLEFFKSHWTAWRYLGVAHFRVENWKNLYVFYSIVSNLLVTLCYPVHLGISLFRNRTITEDILNLT<br>TFATCTACSVKCLLYAYNIKDVLEMERLLRLLDERVVGPEQRSIYGQVRVQLRNVLYVFIGIYMPCALFAELSF<br>LFKEERGLMYPAWFPFDWLHSTRNYYIANAYQIVGISFQLLQNYVSDCFPAVVLCLISSHIKMLYNRFEEVGL<br>DPARDAEKDLEACITDHKHILELFRRIEAFISLPMLIQFTVTALNVCIGLAALVFFVSEPMARMYFIFYSLAMP<br>LQIFPSCFFGTDNEYWFGRLHYAAFSCNWHTQNRSFKRKMMLFVEQSLKKSTAVAGGMMRIHLDTFFSTLK<br>GAYSLFTIIIRMRK
AlucOR40	MAFDASKNNYLEMCLILYDLSGLRASSKPFLKFIALYTLYPLMLIVCAMIHVNIWFKHGNIFEITEVFTSICIIA<br>SITIRKTVLIYYGPLYEDVIQQHSQFWDYGLFGKTTELRLRKNMKFCVFLIKCFLTSGIASIVVRSISPLFVKDILL<br>PQECWIPGNNPVALKVIYVLEILFYLESTTYFPLFDGLYLIMTGNLKSQLILLQKAVESVDLVRDDDEISWRKL<br>KKCCQHHKLLLSVLKKINKIYSVFFLCTYVLTIIGICLPLFVIFNKSSTFTQVVESVLVANIMNTLLIMICIPGSEI<br>EIEAERLITQIYNINWHETSNLKIRKFVLFWLMQAQVPLRITGGGMLIVNRSLMFQIQRIAYSVSTLLTGLTS
AlucOR41	MAFDASQHNYLHLCLILYDLSGLRVSSNSFLKFLSLYVLYPLLLIMFVMVHLNVWFKHANIFEITEVFTSICIV<br>ASMCIRKTVLIQYGSTFEDVIQKHSQFWDYGLFGTKTESRLRKNMEFCFLLLKCFIISGIASIIVRCFSPLFMKEL<br>LLPQDCWIPGNQPVAKKIIYVLQIIFYIESMTYTPLFDGLYIIMTGNLKSQLILLQKAIESIDLKRQDDETSWRR<br>VKECCQHHKFLLSILKKINKMYSNFFVCTYLLTIIGICIPLFVIFDKSSNLTQIVESILVAIVMNTLLIMICIPGSEI<br>EIEADRLITQIYNLNWYETRSLKIRKFILFWLMQVQVPVQIKGGGVLAVNRALILQIQRIAYSASTLLTGLTS
AlucOR42	MAFFIRNKMLGLTITLNTLSWAGLIMRDQYTKTQRIIVRVYGWLVFLYLFVAATYVQIADLIDIWGDLDLMA<br>ETSLLLFMELAVISKILTLIFKYDKIMEIINGTEDILCSENRLEGQKIIASIDKETTRFFQYYTSSVIFTTFFWFLGE<br>HSSTFFIRAKYPFNELKSPGYEFALIHQCMMMVFTGYFEFNINIFFASVVAGCRCRLKLVALSLRNICINIPVNK<br>KNLITPEEEKLITERLHCAISQHKYALDAAEDVKHCLSKVLLVQLTVSIVIICTTAYQMAVNKSTDTIQKLSMA<br>GYLLGASFEVFLFCFQGQSLSNASEDIADAVYECPWYTLTQPLKRTLLIIMMRAQSPAILTAGGFVTLDITEYM<br>AVLTGHGGFGDFLHRTGAEPMAECHHCGCDLDTVQHTLLVCPAWKGWRRDLVVKIGNDLSSVLWHRCS<br>AATSRGRRCLTSASAPSRRRRRGA
AlucOR43	MAFGLEQMDCLTKEEIGIKAHFMRLMNLSGTFSRRKQTRLRSAIIWSVIYVPIILTLVATCIHFRKNFDLSSYAL<br>HHAALITIGFIVNVMTVCMYWKEFHDIMDGSTMSYNYDSGLVKNFAQQTIHERFKLSGLLVKLVSYGSVGVI<br>IEVQIFFAIEAFYLQTYKTIFPMYVPMDLDDPFVFTSVVIWQELVVIYTTYLPLMLAVLYYNAWSHLDIEIKILTF<br>AVANIQRIVEEESQNFRHEGIHRETLEAALYETYSYHFAKHHAHITSYFELFSKCVKLITLLLFTMGPVCLVTV<br>GLSLLSDNIGIRLKLFWFLVIQLIMTYAICWIGQYIADVSTGISEVLVTAPWWLMPKSCRSTFLLIMTRCRKPLQ<br>MTTDYGVPANMESFMDLLKGVYQIISVVIQMRDG
AlucOR44	MAFIMRLIDKCAAMENDDLDRLLDNYYGPMFKLGLVFPSWKRSALVFTIPWFILNVSTFTWNLILLGITVYK<br>AFLCDNDMDLFSLSTHYFLLLLCGSLIIFFMNWNRKLNGLNHTRISVDVGKYKDSRLYSHKDCILMEKQIRV<br>ESYRYLCLPLLIVLICGAVLIVPYASKLFRGVGTMYTTCGVDMFLPIPLYHPFPTHEGLNHFLALISQVLVVCCL<br>ANVIVAIMLNFTQYSLRVKLEYQVLGYSLDTLFARSKKVYLKNYPNEKASFHIRNPDYQRIVGSLLRDSIVHH<br>QTLVDMMDKYHGLITYPVVFAYLTGSGAIGLGLLSIVRALQKGDTETLLLFSLLMLGEVISMLTMSLIGESVTE<br>ATIMLRYKLYDIRWYDMDIPNRRSLLNFQTFITEPLVLTAGKGLVNLTMETFSSIMNSAYSFFNLVNIQQSE

AlucOR45	MAFNESQDNFKLCFIAFNLSGLGPSSKPFLKILSYVLYPWLCLLFVLVCVNIVFKHSNIWDIGEVTSSISIAVM<br>MVVRKTILIKYSSVFAEIIELHSRFWDYGLFGKATETKIRKKVDFFKFILKCYIVSGITATSTRSIVPIFDKNLTMP<br>QDCWIPGNNSIVKHIIYAFQVIFYAESISYFTFFDGFLLIVTANLQAQFILLQKATGSINFETDSEETAWKKLVK<br>CCEHHKFLISVHKKLNTLYSYFYLVTYFLVITMGCVSLFVIFDKSSTFAQLLESAITMVVLNVMIAMICICSSEIE<br>IEAEKLLTQIYEVNWYETPNLKIRKFILFWLMQAQVSVETKGAGLLVVNRSLMLQVQRFSYSVSTLLKGMNE
AlucOR47	MAGFKDPFIVMRTIFLDIINYKIVKVCYVFLFMIYLLVHCLQLYYMIKNFDLNLLIKYGFMTALFSYILVVAVLG<br>LVVEEKIRKTLKILDGVGWSLNIVGKDAEMKLEKKCKMINISVYAIMLFLIITLLVNLPVFGSQRELFISIQVIEE<br>YFGKWSEMLNRLYFTFAIFLSYHGVRLSFACIYGILEVQLQFNIIEEYLCEIYETDSSKSWQYLQDTGYQRKTG<br>KSLRLCIEHHVALKKVIEMMLEISVICLPFLAVVGLANLISCLTFIMNFWDTMDNILKLRIFMWAGWIVLITVL<br>FCRSGQQLIDVTSNIFFTLGGAPWYYWNLENIKILLTFMTNCTNNDSIALAGFCLNYPQFVSIANTTISYALVL<br>YNIRKSSDSDY
AlucOR48	MAGNIPWTDTVLWMNARVLALGGMWRPPWFQPKWYLLYRAWVLFTQFSFLFAQVQALWFFWGNIDKIT<br>HDTCLLITTILGLVKFFTFVLRQEDFFRMVQKIDDSRAEQSKSGDSEIVSILDASYRSARTITLYMTFLGGSSPGV<br>WAIIPTILRRLGVFPPERELPATAWYSRRDTETPYYQMLCTLQYFSMQYSFFMAMCLDLFFVCIIIHAAGQLEV<br>LNARFRRVGQIAGNHSADSHKQQKALEEFSGDVFSPEEIWEDLCDCIKQHQDIIELIKEIERLLSKIVLLQFLG<br>ATVIICVTLFQSSKNTDNIAALLLLQAYLGVVIYEIFMYCWYADDILYQSSRLAMSAYACNWPGAPPQLQRAL<br>VFIIRRTQRPLGLTAGKFYYVSRETFVRLMSASYSYYALLNQVNDK
AlucOR49	MAGYGRLEDGDIVDGLSIWYLKASGLWEMFNHHRETGGRSKVLKFWMAGMIIAYSPVFVVSVVGPFFAEK<br>DLEGMSLVVLNPMSTVQMVVKFGILWFHMEKQSRLLDLMKKNFLACVPPDKEAEVSRILGDAVKEANIYT<br>FFGTRINIITVLLWSILPVLRSEYFRITLGITIFGTPLRHNKLLGFSYPFDYDASPGNEIVFVYEFLLVLSAGLIITV<br>MECLVAQLVVLLTAYLKVFQYFMEELKSTHDPKFDKEQLLLYVKEHQKLMRVGDEVCDLYNFLITVQLSTGL<br>FILIIAIFNFFLSSGNGDVVVMIKFVVYTLYTLVEICVYCYAGSNLETTSEDVCFAAYSCEWYEMNPDFRKTLQ<br>MMMVRSRSPVVLKAGKLYPLNLITLTNIVQMAYSTSMLMYQQTHN
AlucOR5	MAHAQRPRGLPPANVLSANIAILRWSGLWPPERRGGWARLFAAYSAVAFLSQAVAAGMTLHLIYHSWGDI<br>YEITLTMMVTMTLVGGVLKMLHFFRHAGAYHLLVRELPAVAAGMTLHLIYHSWGDIYEITLTMMVTMTLV<br>GGVLKMLHFFRHAGAYHLLVRELRRAALFTRGALGYLNILAPTWFLMPVISGAADDPAGRKLPFTQLKGLR<br>ADDLVGYSVAYFVQCHAIFYWNFISVGLDVFFATAMLHAAGQLKILSHRLSRLGKGPAARQHWNPQDDTT<br>QIRQIMPQEGTNDLYTELRSCIKNHQEILRLVLLLESVMGPVAFIQFLCSVVAACVALFQATFNAEGNGVLKC<br>TMYLPTPAFQIFIYCWCGHEIMEEFSRYSLATRRACXYSSGWVGAGRRVSRGLRVLMCRAQRPLLLTAGKLY<br>PVNRLTFVSLINASYTFYALLRQMRDR
AlucOR50	MAHNPNLFELIKMFKIRDPFAILKIILKAEPFILLKWCFIDLSYHKFVKICNVLALIIHSVYFVFDVLYTIENFSL<br>DFLLKYSSSMLIYIYVITNMVFAMVMEKYTLEMVNLSESEFWPMDFAGKRMEAEIGRNYLMTRIVYYFLGGA<br>LLASMLILLPFFGDLNDWLLSSQMSVDYFGEWSVLVDLILFSTGPMVALSEIRAPAVFLYGIYKIDLQIFLLNKL<br>IVQLSHEKARDDAKYQRRIFAKLRQFTKHHANLKKCMRGISDIMNLSMPVFVFIGAPGVISIMYYFLYSLDSA<br>STITKIRSVYVAVFTSVIVATFAHAGQKISDNTSLIFDTLTTCPWNLWDKRNRKVLLIFMANSVKPMTFSVAGI<br>TVDYQFAIKMIKLSSSYALVLYQLKNQYNMN
AlucOR51	MAHTVADLFLRNMGWSEKHGYYTMCMVFFTYSELALTLLFIISTCLSIVYSRENLSMHLHGLLWLLVEIHVF<br>AVTANRLYHKSKLRDMHQRSQKVRIPENYRRTIANVLTYHMSLPNVLVAIPALYMILLDGVQMGDPFTFPFV<br>DVLPIKTTSVTVYVCKYIVYAMPVYIGQLETCFLNVSFMYYTGVVKRHFQILEEQVAEAMVNKDEQKLKIAI<br>KSHQEVLKYFKDMKTVSEIPILVNIEFCSFYVCLTCCYVIQAMQGFINQMILGIIIHTSIAGITTITIYCIYASNMY<br>DLHNGILNALFEHRSCYSRNKSFTQLILLMKTKATILLEFKAGFIFTLNLNLLVKIFRFAYTVFNVLLTSTNRQF<br>KESAI
AlucOR52	MAILERNIFILTLVGVWKPNHWKGFKAALYYFYICLVTIVNHSFLLSGVLDFELRNIDVVVIIDNLSLLICVFTI<br>RYKIITLLYYRKFIEEFVNCFERDPFRAKDNEEEQIYVQFDKRTKTLSILYAGLFTVAVSWYSVGHVLRMSPPN

	VMPYQGWFPYNYTVYKYYWPTVIYQLYAVCSGAWVNLAYDSLFCSILYYVCAQTHILKHRFSVLAENLQKINEENDGNDNKEIERKMIGDWVDYHNNILDLVKFVKSFFSTAIFVQYAASSLLICSIAYTLSHTETRSLNFAGNFFYLIAMTIQIFFQCIAADQVTVEFADITNALYSTNWYNLSNNAQKSLAILAEPLKPTLINSGYFVILSLDSFTKVIKLSYTIYNVLE
AlucOR53	MAILKESFCVLTGIGLWRPVEWQGIKGAFYNCYTLLVLLNSITFIISESMELIFFNDGIFDFFNNSSMLITVIGMCGKLITVVTNRETIIKMIERFHRSPFSPRDYEEEIIHKNFNQKIRFNTLAYIVFFEASVTVYTVGKIFEDRPPGVLPCRAWLPYDYSNNIIIYWMTASQQLLTVVMTANVDIAYDTLFPGMMMQVCIQINVLKHRFRLTLDALENISDDKMDPVMVKTVEKKFFSEMVDTISHVFGPMIFIQYSFSSVVLCSSVYALSQMVPFSPEFCACSVYILCMFFQILYICLSGNRVTLEFAKLGTAMYDTYWFALSNNAQKHIIMMMMSSVKPIIFASGHVVTLSLESFKRLLKLSYTIYNVFQQSS
AlucOR54	MAKARYLQDSDDPFIFIRKLFVDYGYSRIINSYNRFIFIFHTCSLLLEGYYIIKNFSIDFFTQYGGATNLILYFLVTQFIVIIKQDFVHQIVEESKSLYWSMDFLDVNKQAQILQEMTKLKRKIYVLWMSTVIFAIMMLPVWGDYSEAHLFPQIYQTYFGNWSPIFYYFYVSTFPFAAYTGLRIAAMALYFTLIIHFQIILLNQKILQIAQERDTSQEEIAKNLRSCVCQHVTLKRFVAKVLKSIENAVPVYFCLAILCLITVLFVILNNLDTSTSNHLKARFLFAGIYGTVILYTFTEAGQLLSEINDQVFNTLMQCPWYNWNTKNKKTLMIFMLGSLKPVQIVWGGVNVDYKFGGSVIQTCCSYALVLYQMRNAK
AlucOR55	MAKFNDPFKFVRTIIFVDMNSYKVIKTCNVLLNIIYSLIHCLLIYYLCKNLEINLLIRYAPAILLFILVIFGAVFSIYMDEDILEVRSVFRENRWSLSVLKENSQTKLGRKCQFINIFILLVLLLIVSTLAINAPCFGNQRELLICIQVFEEYFGEWSFIPYYFFFLGFPLLYYNFFRLWMTFVYGLLEGQLQFFILEEYLCGIYETEDSKSWKYLQDSRYQQEIEKSLRLCISHHIGLKKFLKMVENQTLKVMPFYLVFGVLILICYFSFIINFADTVTTIGKIRMFMTAICMMGVAILLSWIGQQLIDVTSDIYFTLGGAPWYYWSQKNAKLLLMFLTNCTKNESVTLAGISLDFTLFVSIVHTTLSYALVLYNLRESSLVSSSQK
AlucOR56	MAKLEYLTGATFTLKCAVLYPIDSNNPKIKKILYAVWAIFFILTFVTGFIQCFVFVCINPFDLVQEAMIIMSLVFYSTTFFYFIVFYKNWQNMVALVTNINKNFHRATDNVIEKISMDQASELSDKLAYVWTSSLAVGSVVPVVLAIATGNLEMPMPAWFPYDYNKSPVFEITYLWQVFCLITLAIIYGASDMFFPCITIIIGQQFKILASNFKNNFYTSLIKLGAEESIVQNFSKDIKTHEFRSFYIKYGNIFKILNNAKFQTLNRAFLKRNIKHHKLLLRFCEDLNKILNTFLLIRVSAIVFNLIFIGFNIIISTDRTLMLGFCNYFCFGSTELFIHTYSGQILTENADFLWTLYECPWYLCDVTYQKMLILVQMRVKRMVSTKAGNFFTMIAPSFIAFQRAVFSYITLLKEVTDLGKD
AlucOR57	MAKLFGFHIINADSRKCLSYRMSFPMTTLLMAFNLCMLSGNAIGCLIAALLDSNFDRRLMNVKGMMLLIMIILLAINESFISRNRVNRILDYINRIKSITRYGFKEEEDIMNKAIDDCYKSTKYSTTFFFTNSMLMVTLPPTMAIITGESWKQLPYPWVITQTNDWLYYSSLVLQVVATALCHGVGAAGFSLLTTMKPLAAAFDKVILGINRIEERAARKMSEEGITYQESMLSCLKESIAHHQEIVDELLMEKPHLEIMFFAQVTFISIVMACEAYPIIMGIVDVSGLIRGVLFLFIQVMCCGFLNLEFDTIANKNVEVSEALYGTPWYALGVEYRHVVLNSMTFSQNPIWICGMGFFGLRASRATFYSAMVSACNMLNMFRKFA
AlucOR58	MAKQKEKSSRLQDLDSGDGEDLSWDEVSHSVLKNSVRVLYCMGLWPLRSSRAYHCFTAFSLASSAVVIAMDIVAACYSLGDIDQMTGALSTILPMSGGLANGLLMILHRPDLCRVVRAVDRLVVHQQRYLRQDSHLAAVVARVRRQTLLVTIGVSCYLITIASYWIVIAFGKPTGLRVLPFVQLPWVQSSGLAHYWSTFAVQFYTAPFCSYATLSVEFFFLAVMLQLSAQFEILGSRFASLGRNPPPKAVVTTKSDMGTADSDAVYEELCLCVKTHQELLRFVRFLDDVMSPFAMLQFVAGTLAVCVVLFQAANNQDLNTNLKAAGWLPAPSLELYIYCGGAHEVVYEAEALVQAAYDCLWYNTAPRVSRAIRLVITRAQVPPVLTAGHLYPITRPTFVSLVNAAYSYYALLCQMQNK
AlucOR59	MAKTGDPFIMLRWILLMDVSNNKITKYCNIFLTTVYSLVLCLQIYYILKNYDINLLIKYGPITTLLLFMITVAVISLLLQKEIFETDTFIRETCWPLNIIQKSGQIKAERKCRTINFYIGSTFLLFLSVLILNYPCFGSQRDFFICIEMFEEYFGEWSSVFYYLYFIGSHFLYYRLFQTSYLFVYGMLEANLQFFLIEEYLLQTYQTDCLKRCKYLQDTRYQQEIGKSLRFCIKHHIALKKLVKMVVNLGVLAMPFFLVFGVLLLISCFTFITNFADTMSNILKIRIFMFVSSTVAIALLLCW

	IGQQLIDVTSDIFFTLGGAPWYNWNLDNIKLLLMFITNCTKNESMVLAGIRADYDLFVSLLRISASYALVLLK<br>LRKCTFV
AlucOR60	MAKYSVRGGGGGAPALDLRLQLRLLRLAGAWGPSPSASSTSLPYAGYSAAVVLLLLAFVGSQVSAMLHFW<br>GDILSVTTNACVTFTYTMAMFKLLVVLIMRPSAEYLIQELNRCMQEYGRDLSSEKAAVLARCGRLSRHVAA<br>AHVAIGAVAYVCGVALPAARGRLCPSAACRAADGFPVLVWYPFPFTVSPAYELVFAAVSLDFFYGYILSTTLD<br>GFFVTLIIYVSGQLRLLNLMAQNVCAGAGGGGSEASEQRVRDRLAQCVRYHTDVDRCVQRLSALLGPILLG<br>QVLADVVTISATAFVTTTGKTDSGWVFKYGSYLAAIAEQLLLYCWFGNDVLTESERLQLSAYSSQWVSAPAR<br>FRKGLLVFLCRAHRPLRLTASKFYTISRETFLLLMNASFSYYAVLRQLNSD
AlucOR61	MALKKMLALTKGLEDPTHPLLGPTLKALSVFGLWQTGSQKSTVIYNTFHFLTFLFVITEYIDLYTVRKELSKM<br>LNNLSVTVLSTICMIKTLSYVCRQSHLKVLVREISELELELMKTTDKNIVKRLRQYTVYTRAVTYVYWFLVVGI<br>NVVLLTSPLLKYASSEIYRSEIKNGTEPPPLILCSWFPFDSARMPGYFWATMVHIIMSIQGCGVVATYDMNAVA<br>VMSYLKGQTSILKDKCKAIFDETASSRDVLNRIRDCHRHHNILLRHYYMFNSLLSPIMFVYMLICSFTICCSIIQ<br>LDSSETTISQRIWIIQYSIGQISQLFLYCWHSNEFAAKVKKKHFPLFPINLF
AlucOR62	MALNQEDAICSKSCFYLRYSFLWPEEAPTRSFYAKFILVLILSFLTAFLPLFIHFLILVERGLDPSEDLFVIISYTGF<br>ALIMIIYVIHVKKTSYLIVQLSDFEKFGKPRGFDYWDKKFRLISSGVYYYVLIASSGLNLGRWVGMAECRKER<br>DFQVCGIVIPYWLPWKVDSWLFFILLDLYVLKMTLVVNCALFLIIIQILEITTHLKLRIDHLKEMLVKCFDSDS<br>QTNRKQLVNCIRYHTYIINCSKLFKKCFTHAMFSLIVTMALSCGCLESQVVKFDLWALPPISAWIFILFIACMA<br>GQILMNASLSIGDAGYHSKWYQTDANFRKYLILVLMRSHKALVLSAGPFNILCFELFVAIMKFSYSVFMLLN<br>QN
AlucOR63	MAMKQYPFLYKIFLDFAYAKIGKMVTYSCIIIQSLALQLQVYFIVTHFSKELIVKYGPGVLVVTYLVTSLVVELM<br>IENKTRKIIDFARLTFWPTDFCGLEAKNRLIKNSSKVSIVIYLILMWFAAQGIVMFPVWGDTSEWRLHVEIFD<br>QWKLFYYIYVSTFTIIVFSAVRLPGILLYSIFQTHMQIVLINQKITQISQNDPNDIRMMNQTGYQKRIYKEMCL<br>CVSQHIAIKRFIKKLLEIVRPVQPIFMVLGLLGVISIFFFALYNLENTSNILKIRLVMVVISCILILCLFAEAGQAVS<br>DETSRVFDTLLTCPWYLWDQRNKKALAIFLSNSLQPISFSMAGFTLNYGFGISMLRNSASYALILYKMKN
AlucOR64	MANFNWTKIIETNFVVLKVIGLWPEKTFFACKLYNIYTHFMVTCLLVIHLLLQTIQLALIIDEFQLFLTALPLLL<br>QQYHLLIKLFYFMVKFPILRYILHSLNNHQVFQPQNQDQKIQMEDRLSFMKKIYFSFYSMAGVAISFLVAFPIL<br>DILNGGERQILFVCWFPYDYMTSPFYEFTYFYQSASIIYAGVIVLQIDTLVTLLMTYLGFQCDLLCENLTQVGY<br>NNSKENNTELEFVKCIKHHQELIKLKNHCVDFSSGLIFVQVATSSIAIGLTLFQMTLNVSTFNVIFLVLYGLSVT<br>FQMFQYCWFGSEVIHKSDKIAYSAFEMNFVDAPLSVKENLVIFMACTQKPIKMPVLKVTHLSLQTFTKVLRT<br>AWSYFALLVQVSK
AlucOR65	MAPAAASSSAAAAAAAASASDLGHLLRPLHWAAVLRHPHSAAASPLFFRLCTVAMASFAFTSTCSEVTVLFR<br>DGTADLDAFTLTLSVVDTNTIWLFRMAHTVACERAYHKLAHQVRNDFGEFLTLEDLPLLRGQSRVVRRFAL<br>AYIWFGVGACAYYLVSPVSAEGLPFILALPFDATRPLSFAATWLFCTVTCLHVVVMTMVLDSLNVSLIAQLRI<br>QLTLLSGKIVGIAKEMSEKPVRSSETSLYSELHYRLEKCIRHHQTIIKNADLLERSLGAMLLAQSVAIGAAVCF<br>QMFQIATSANGLQQTGKFCCYLFAMLAELYVYCWFGDDLITESENVAQAAYDAVTSLQECPVSIKRSLLLLM<br>HRAQRPLRITAGGFFPLSRESFVSVVNVSYSFFAILRNFKDEEE
AlucOR67	MAPKQIDCFEINWKFWKFLGIWSENKPHRYYKYYSKIFITFFVILYDVLYTINFYFVPRQLDLIIGEMLFYLTEL<br>SVLSKVFTFIIMRHKLKIIFEILESDAFQTDTEEELKILHRAKVFIKRYWKIVALVSITANLTHISSPLLKNLIFKVE<br>LVLPVCSYSFLSESFLKTFEYPLYFYQIVGIHFHMLYNLNIDTYFLGLMILIIAQLDILNVKFRNLKSGKDHTQL<br>NESIMGLNKNLDHYNEIERFCSLVQNIFSFTLFVQFSMASCIICVCLFSFTLSVPVEYYIFLATYMFIMIIQIMVP<br>CWFGSRIMDKSILLSSAIYNCDWTSNSKDFKINMRLFVERANKPLSITGGKMFSLSLATFTSIMNSAYSFFTLL<br>RYIQTRE
AlucOR68	MAPQVNLFKAWMIWMKIAGADPPSVNFPYALALLWKLIMLYGSVHYVIIMFLAIVVGDSAFHLKLEAGLFL<br>LAGIPCSYKHFVFVIRKNKLHEVIDRLNTLLEEVEDVYGTETLAGWQRICNMVMYFYSTQFTMLVVPVFSFF

	YYIYYWEGVEATPYEVYIPFEKENHIHRVMLYELLSFLGPAAGLITGNIFFGSLTVAVSGVLRKIQEQFSQVSPS<br>NAQFLLHRTIRWHSEIISIVGETNRLLGTVFVVEYLLAMVYICFSGYMLLKVGSATEDVNLNKNIILCIVCIVM<br>PLFYCLCGHVIVLEYDKMSDSIFQNDWVSLQPVDRKKLILPALLAKRGLSLHYKKLLKFDMTTYLKIVKQSY<br>SFLTMLKLMENT
AlucOR69	MAQQSNLEPSDSLKTLYLMTFGVMRLRLVKAILIIIFIFHSTIGVIQIIELLVTFDGNQLLQYGPFFLVSCSCLVTI<br>SSVFTAEKILTKLFIKLAVVRFWTLTRFPPEISKKIAFESKIVTGASIIALTVMFLFSTVNVPFFGDKNVIYGIMLIQ<br>KSFGRTAKLFCFLYYGTLPILAYVAFISGGVLIYTLMHLRFYIFVINHYLERIVLQYDLVISDSHKLRDEEYQKSI<br>YDQLKICIGRHQFFKKIEKKLNAKLLPYLLLLLYLGTAAVLSMLFNILVDASKFGYFVAIGGMVVSVIVPLLLV<br>HVGQCLKDEYEKMFANTLKFPWIVWNSRNRKILFIFILNLQKSLCIGQPGLVLCEYSFIPSVLSFVWTFSTFIV<br>QVASKSR
AlucOR7	MARITDVFRLNFIFWKFLGIWGKSAPSKYNMAYTALYLSASLFVYDIFLTLNLIHTPRKLETLLRETMFYFNHL<br>VAMTKILKMFIRRKKILVIFDLLDCEEFKPSDEDSQEIMKRKNEFYYIYWRIVAVTSNLSCFMQVVGPLIKMLI<br>WKSELGLPVCKYYFMSDEFRNKYFVIWYIYQSFGIYNQMVNNLNLDTFNCGMLWMAVGQLQILKTKFVNF<br>KLNDIENSLDLKTRDDMQTERLRKYLTHYEIILKYCATVQDILNITIFVQLGMSSIVICVGLCGFVAMPSNTET<br>AIFMSSYLITMTMQIFVPSWMGTQISFECGELMSAAYCCEWIPRSKLFKRSLILFVERAKTPVRITGLKIFTLSL<br>DTFTSIMKTTYSFFTLIRQLQVDEVN
AlucOR70	MARTDLSDVIQLLQFTGHYFSFKGSRREKTYETLQKLRVVFMVICNPFTLSSLFIGGLKKSMGVELFFGLMGF<br>LTAMQHVYAYRHRKTTEDIIRSILEIRRKYQQGSDIEFQQNTRAIWKVVYIYFSAMTSLLVFYITIPKFVDILYGI<br>LWDDPVALRLPQSMDAYLDEHQHRNLKYATVALVSSSWSFVSTYSHFGLDTLLSLVGFYYSSLVKTFCNRLK<br>LNTHLTSKELEGHIKILAAHHHELFKLSLKMRSIFGCPYAMQNNFGAFCIVSLVYALLSDDSSGLLIKVANLF<br>NLMILAGMLTSTSYIGQHVTNEISAIFDALYDLPWYELSPSNRKYLVTMICVARDPFTIHFHGRAPLNLANFM<br>AILNTSYSYFMFMRSTL
AlucOR71	MASPDALLRHNVRLLRLGGAWPPEHGRGLRRLFPLYTASVYFCQSATIAMGALLTYELWGDVDAIMLTYV<br>NTFTLLGGFVKLVCFSGDVRGYRALVAQLRAVARHQWPHCQADAHLMAIFGAAQRAALWLTFGPLAYLN<br>VLGPTWFFMPLIVRATTGSHQRLLPFVNMRDSVTEIFPLYVAIYVVQVYCMFYWNIISVGLDMFFVSCMIHV<br>AAQLRILNERLSNLGRARADDDDDSCRAEANHKNIPGFGSSQKRSFSREGRVGNMYEELRNCIKTHQHILS<br>LLKTLQRMMSPVAMTQFMCSASGACITLFQATFNPEGNSTLKCLMFLPMPAFQIFIYCWAGHEIVYQEELLS<br>LSGYRSGWVGCGRRASALLHILMCNAQKPLQLTAGKFYPVNRDTFVTLINASYTFYTLMRQTRDQGSIVQT
AlucOR72	MAVDAPWSDTALWLNARLLALGGMWRPPWCPARCYLLYRAWVFFTLFSFFVAQIQALWHFWGDMDKIT<br>HDVCLMISIILSITKFFIFNFKEREVFRLVRRIDDTRAEQIETGDSEITSILDASYRSARGVALMMTCLGGSIPGV<br>WAVIPILMRRLGIFPPERELPGTSWYTGRDGETPIYETLYVLQYFSMQNSFFTAVGPDLLFVAFIIHAAGQLEVL<br>NARLRRVGGASDARKLQKAREEEEESGEAGCGELAWRELCGCIRHHQHVIGLIKEIERMVSKIVLLQFLGAT<br>VIICVTLYQSSKHTENMAALLMLQGYLGLIMYEVFMYCWYAEDILYQNSRLAVSAYSSGWVGAVPQLQRAL<br>VFVICRTQRPLGLTAGKFYYVSRESFVSLMSASYSYYALLRQVNDK
AlucOR73	MAVFKLIKPAPLTEKVQSRQGNIYLYRAMWLIGWIPPKEGVLRYVYLFWTCVPFAFGVFYLPVGFIISYVQEF<br>KNFTPGEFLTSLQVCINVYGASVKSTITYLFLWRLRKTEILLDSLDKRLANDSDRERIHNMVARCNYAFLIYSFI<br>YCGYAGSTFLSYALSGRPPWSVYNPFIDWRDGMGSLWIQAIFEYITMSFAVLQDQLSDTYPLMFTIMFRAHM<br>EVLKDHVRSLRMDPERSEADNYQDLVNCVLDHKTILKCCDMIRPMISRTIFVQFALIGSVLGLTLVNVFFFSN<br>FWKGVASLLFVITILLQTFPFCYTCNMLIDDAQDLSNEIFQSNWVDAEPRYKATLVLFMHHVQQPIIFIAGGIF<br>PISMNSNITVAKFAFSIITIVRQMNLAEQFQ
AlucOR74	MAVLEASGRQTLGEATELWGDVLRRNVKVLRFGGAWRPAALVGWRPRLFPLYFGSVCGSLLNIITLDMVRS<br>WLLWGDMTAVTFALVSAMTNLNGVVKMVHCFRHHGTYGRLVSELNGLVALQRPYCEADGALLAAFRRAS<br>RRAARLTVGCLAYMNVLGQMWCVVPLLTPEAPDSRESALPLVSLPGLRSRNRLWYSFAYLVECHAVFYWNF<br>ASLGMDMFFASAMIHVTGQLDILNIRLAQLRREGSTEDQFRSFASDAGRSDRQRERGDGDSSKMYSELCEC

	VKHHQAILKYLEFLESVMSPVALTQFLCSVVAVCVTLYQITFNPEGSGVIKCAMFLPIPALQMFVYCWCGHDIMEAGLSVSLAAYSGAWVGVGRRVTGALRVLMCRAQRPLQLTAGKVYPVNRDTFLSLINASYTFYTLLRQMRNR
AlucOR78	MAVSTRVATKQEVPESRRAFRNLFNCFYALGMQAPDGSRPTTSSTWQRIYACFSVVMYVWQLLLVPTFFVISYRYMGGMEITQVLTSAQVAIDAVILPAKIVALAWNLPLLRRAEHHLAALDARCREQEEFQLILDAVRFCNYLVWFYQICYAIYSSSTFVCAFLLGQPPYALYLPGLDWQRSQMQFCIQAWIEFLIMNWTCLHQASDDVYAVIYLYVVRIQVQLLARRVEKLGTDDSGQVEIYPDERRQEEHCAELQRCIVDHQTMLQLLDCISPVISRTIFVQFLITAAIMGTTMINIFIFANTNTKIASIIYLLAVTLQTAPCCYQATSLMLDNERLALAIFQCQWLGQSARFRKMLLYYLHRAQQPITLTAMKLFPINLATYFSIAKFSFSLYTLIKGMNLGERFNRTN
AlucOR79	MCFLKIKQQIIDIQKHFKDYSLNGSLWIVNLLPRLMGFNLRADKVGVFFWTIYILLLVYVFGIGIFVYLWKHVDTMSGLMKSYLNLSLILVIVNNSCWFLSKRSLLNKVLKKIHLIEDLSCESEHALAKYRRVFKIVTHLLLASYVLFYFTEIYFMFLFRNYDLLEDYSLAPCVGLEPLSSSPNSEICLIIVLIHEFISTTVMMSFAALFLVLIAHTAVMFLVLAEDMTKLTDLINLADHRKMIRESLRSLIHRHSLLLQIVYELRLLYSVPLGINFISNAMSILVLLCLPIHEWPSFLHIIGYCFFAFFLYCFLGQNVINASEKFIDAIYCCGWEHFGVAEKKLVHVMLRQAQKPVEIIALGMISVNMNTYVEALQLIYKFVTVLKI
AlucOR80	MCNLHDRYSQFGCFIVITFRQDSKVFFLTNMYSKLFGERGEFSMSVVYFVATGLFQIKLIRLMVYLLLIANTLGFLAILYQFILDAELVYIIQYGPIISGSTYALGSLYGIIFLRDAEEFQHGFQFWNEHEGSKETQNRIKQHINSLTVSVILNTALAFVTGTSLILPNKDEIHYHYFIKILMDLETVPRRLSQALYYLYKLNFVMMFPIMTINSYRVLYFSRKFKFQVMLLLEHIETLTKNYNVDDINLFYNTRYQDYIKQKLIFFIRRHSYIAQYVAKINNSIGPFVVLYAISATLLGVSVLLIVATGTIYYNTYQIILCGAIYLSTIFCAVDGTETVEMESVEIYNKLLGQPWYTWNNENKRIFVIFLMNCKKPLQITKFSDTFYVNYDWGIAVLKKVYSLGSVFFNLRRYIIDK
AlucOR81	MCRKYMHGSGLGPTSPKIKRFISLWLLFPVSLLLDVLVIYDFHFLDNDIFKTAELLESVSSFGQLPIRKFILTYHSKLIQNLLEDRKKFWSYEMFGETYGKFLRRKMVLATRLIQTMIFFGASVATLMFVSTLADDRKTVPLECWIPEFKHSTHVVLVMQFCSLCEIIYLVGAVDCLYVLTCVDIKIQFLLLQKKLKTIQVGVKPMEECLNELTICVKHHNLLLRSHKSLNRIFSEYFFVQYFVSVLAACVQLYILMYITASLEDIMKSIVYLSAVVFQVAIFFMPASDIEEEAEQFAVEIYNVNWECTSGTKFRKQLLFMLMKAQKPLYMLGGGMIHANRNEYIVLFRLAFSISTLLGGMNENGRTDK
AlucOR84	MCVRVPWRDSALWANARLLSVAGVWPPPSSGGWYLLYTCWLFGSQLFMVAGQLAGLWRFRGDLDKLTLDVCLTVTVVMGVIKAGAIVARRRRFFGIVRRLDAATAAQLLAGDPEEAAVVASAASLARTITVWAPVLGSLSPVVWGLAPLLLRLLGNAPRPRELPVVCWYGGWDAASPYYELLYLVQFVTIQGGYLVVMGSDLFFVSLMIHAAAQLRILNMRLVKIARNEVENDKTFGGMKCVPGYRNSNLKIIDKEWTSSATKTQDLTDETSSYDELRSWVEQHKDVIRLVQQLEQLLNVIILFQFLGGTIIICVTLYQSSAKTGEVTTLFKLQLYLGTMLSEIFMYCWYADGIVQQSARLATSAYSCGWPDAPQPFRRSLLIIMRRCQRPLSLTAGKFYTISRATFVRLVNASYSYYALLRQMNDH
AlucOR85	MDAREGKISSVQSQCKPGGIIPVVELVIPDAMKRTGCGLLQADALCDYFRLVTELDAFVSEQRSKYYNNEKVIEMLDASSKRTASITKAVMAYLICWTFIVVPALFLIESPYSVLPLMAWYPFTANIWPRYEIIIMLHFLTIGYCFFTSWGMDLFFGCLMYHLSLQLRLLNYHLANIRYRCKSECVLQEGFEGKTDFSTPRVELEVIRNGREKQQVAEYTRSAEDAIYVDLLQCIKHHQRIIRYADNVENVANPVILSQFVLSVLVLCVVLFQTSSELGTLTALVRFLVYLLELLLQIFIYCWVAHQIFEECDSLLQLSFVVKTLDGRRAIRTRSVKCLLQESFWSVMIMPRPVTFSAGKIYAIDRTTFVSIVNASYSYYAVLRQINN
AlucOR86	MDASYFAVQRRALEIVGFDPSTPQLSLKHPIWAGILILSLISHNWPMVVYALQDLSDLTRLTDNFAVFMQGSQSTFKFLVMMAKRRRIGSLIHRLHKLNQAASATPNHLEKIERENQLDRYVARSFRNAAYGVICASAIAPMLLGLWGYVETGVFTPTTPMEFNFWLDERKPHFYWPIYVWGVLGVAAAAWLAIATDTLFSWLTHNVVIQFQLLELVLEEKDLNGGDSRLTGFVSRHRIALDLAKELSSIFGEIVFVKYMLSYLQLCMLAFRFSRSGWSAQVPFRATFLVAIIIQLSSYCYGGEYIKQQSLAIAQAVYGQINWPEMTPKKRRLWQMVIMRAQRPAKIFGFMFVVDLPLLLW

	VIRTAGSFLAMLRTFER
AlucOR87	MDAVVGPLLPLMRLLGLWPCSGGSGRLPAAARCALTQLPVALMVAGSALKLCVDTPDQFEDVALCAFITNVVAAILVKAVMLVARGQRLRRLARLLADARARFPAHRSCTRGRYQALADRMERLFQVGGLVPLACWLSAPLVPQLTAAPGQGRGRPRQLPVPTWLPADLAASPTYQLVYTLQVLGCIGACASTVCADSLFVRLMLLIAAELQVLKENISSLRKTDSVRGGGYACRCRETVSFLASACKDCHDIVTPLSEKTTDEMHQLLVKIIRHHHMIMRMVSLLQEVMDVSIFILLFANMVNLCSSLFTAAILLQGGGSVVKVLKGLSPLPVVLYQTSLFCVFGHIITDKSGELTDAAVSCQWVDCDTRFKRSLLILMTVALKPLKITVGRVCTLSREMLLQVFHGSYALMNMFYYYHHKTK
AlucOR88	MDDFNWISTVKTNLLLLHIGGIWPRGDGTHKLNLYTIYAIFITFTFTTYHCFSQIINFFFVDDLQALTESIFISLIQSMALVKAFYILKNMRILKNILKNLETNKMLQPRNLKQIKMVQPSLTQWRLLSQMFWISAVFAMCLFGAFPIVESTYKEFRLPYLAWYPFDTKSSPFYEIMYLHQFVSSYTIAIVDIGADTLIAALNVFVATQCEILCDNIRNINGSVEEMDSKWKECFTHHKEILKVARHCQKFFNWIVLMQFCASVICIGLTMFQLTLVVSFSSEFFSSLFYFGAITVQIFMYCWFGNEVELKSSKILYATFEANWVEAPHQVKKNILIFAIRCQNPIKMSSLNVFYLTLETFMAIFRTSWSYFAVLRQIQNRISEE
AlucOR89	MDDGALPQPVVDLWRPCLFLRAIGLWAPARLRWLYSAYTVWSLLQLVVSVAGQLAGLQGHWDHLPTVATSVCLIVTSICTLFKASSFALRRGRVDSLVSRISHNLSTFCAHRPRTRAAVVWAARRRATRMFDTFLGIGGVALVFFYLGPIIQNAKDARVAASAAGNETLPPLLGRNLAMLLWWPSGQPVETPAYQLTYVGVCYWLMLLYLSTSTLDAFYVTVIIYLSSQLKVLNVDFLSITEGDDDSPAEETDPLSKRGGKHELEAKLPKGYEEATDQRTQERLLECIIFHQEIIKTVDEMESILSASILVQFLASTLVICFTAFVITTAENKQDLPTYITYLATMFYELFLYCWYGNELLAESERLQTSAYSCGWVGRSAGLQRSLRVVMVRLQRPVCLTAGKFYQISRETFLLLLNGSYSYFALLHQMNDH
AlucOR9	MDDPFIFIKKIFIDYGYSKPLNYYNRLVFIFHTCSLTLESYFMLKNFSLDFFTKYGCALILSHYLLLSQFVTLLNKELIKELIDKRGRFFWTIDSSAPSVKNQILKKSLKFNRKYYFVLFWFVALEVTLLPIWGDLNETHLSPQVYKTYFETWSPFFYYFYVFSFPLLAYHGIGFPATVFYFILQLDLQKILLTDMVLRIPDSGQEAIYENMCLCISQDKKLKKWIARIQYLLKKLMALYICVAFLCLISVIFFILSNLTNSRSILAKSRFFCAGMAGGVVLYTFCESGQLLMDYTEDVFSTLTQCPWYGWNTKNRKLYVMFMQNTQKPLIINWGSVTLNYSFGGSVIKNCCSYALVFYKLRNGQ
AlucOR90	MDEHSHFETSLNKIKVLFKYSGMNLENTVTNTYEFLNHRWVYILNHAWTLAAVTFICIGISNGQNFIEMTCIAPCVAMTVLAVSKSFFHYINENAVKSLLENLIELERTDFERTKSVQRTEIVATEKQLLNMVINVLYVLNCSMILVFDMTPLIIIAIKYWTTNKFVRLLPYLDIFVFVPYKFEYWVMAYILQIWAECIVLLFIGAADCLFFTCCTYIRIHFRLLQYDFERLTSSRRESDGLRDDEDFRETYTNLVKRHQGLIESSSILEMIYSKSTLSNFVLSSLVICLSAFNVTVVNDVTIVMTYLIFLAMSLMQVYFLCFFDMLMSASEEVGNAVYNCSWYTEKASTGKDLLFTITRAQKPCELTAAHFAYVNLKAFMRVSFTSASITTLPTI
AlucOR93	MDFFDSHYYKGMKILLCLIGRWPYQTLKERVLITIILSILWSYTMFHRMLVYSNYDNYSSKHEVILETISPLIVDTITFAKYITTICKMDTIINLLESIKQDWKIYTNKEEKKILEYYANLGKILSLGYVGAVYMVTVLFMTEPIVEQTFFKLFQNETIPKRFSIPIYWKTPDIEKYYYYLISFQTLNTNFIISITCASDAMFINLLQHVTGLFSVTGYLLENVPIEENSEENGQKKIKDVAYEHYVRCMRSHKRALEFAENLESIYVWCFGIVITLNMPVMSVTAMQLTTGTSNVIQSMKYGTFAGVQLLHLFFYCFMSQKLLTSSSVIPECAMNGKWYLCSVKAQRLVTLVIMRSQISCQLTAGKILVLSMETFTSIVKTSGSYFTMLVQMRNV
AlucOR94	MDFTIRDFDLRNSFSLERKLLLVLGFYPIRDKEKHRILHQLSAFLNLLLYYGQLLTIIIQMVIDRNDLSKLTDSTLYFLTLFTFLCKLFNFQYYGKDLIEVEKSLTDPIFYGYSFHKLQIIKAKVRSCTLVCLAFRISCTCSCFIYSVVPFIDRSGQKTLSIPGWFPYDTAKHFYITFFLQSLSLFISAHCNSATDTLPCKLISLATAQFELLKDNLRTIDYENSFEETKHALVKCITHHRKIVNYTKRVETIFSKGIFLQLFASVLVICTTGFQLVIVPFGSLKFAIHGIYLCAMTAQIAIYCYYGHDVMITSDEIGTSLYMSNWYASHIKIRKIMVIFLEKTKKPTIVLAGNFITLSLVTLTQILRSAYSYFAVLRRLYADD
AlucOR96	MDFWGNSDWRFLRFQLCTLGVWPFQKSNFKRVVGFFVILSVQSITLPEVIKFTYIWHDMEEFADCFPLIGIHF

	VCTIKWMCCVVNMDKIIALLNMIKSDNLSKELTEEEHQILRDTGKINRLFVLVYSIWIYVIAILFLVFLPLIPVT<br>LDIILPLNESRPKIELYHTDYLFGPVKYSWVISLHQCIISPFPTIIIIATGSLYCNCCQHACGMFEVIGYRLKNLDI<br>TIEAAMNKKNLGYYADTEIFYKSLVTCEQMHQRMLGYVEKFQDIYSLTLCFSMTTSIITLCITGLEAIIKKDQF<br>FEVIRYVTCGLAEIADVFILCWYGQKLIDSSDYLYLCACQGNWYKYSAKSQKMFISMIVRFSTPCSITLGKLYR<br>VSLECFSTIMKTTMSYFTVINSLREEE
AlucOR97	MDGNIERHFDHMEKFLKYYWQWYGYPDTFKGRLITIFNIFRIFLLMVLVGIAASQAYFYGMSYLVDGSAIFLP<br>LGIMSLVLNSHQSWNFGSIVKTAKQFELFLSSFQDEEERNLIASRMKERKRTVLQIVLIIEFYLMAPLMCHAIS<br>MTLHYYGFLKKPVLIPLLFEMFLEGNYELGPKLIATAVVSVFYVHLVANIVTMILLNVHFLGLVVACLEVLTE<br>RLKGFAEKTQEGYGKLKEELDLRDTIQQHADLLHIINSFNSWNGFLVTFCLAACSITFCLDALTTKRALEQEI<br>YSGACLWGSFLLVMMVLSYLICDSGSQIETKSEELLRAVYNLPWYRGSSETRKAVWMMLTQGNRLIILNYKE<br>LMDLNMVTYLEMLKRAYSYFMILSSIE
AlucOR99	MDHPDIKPMTDDPLKLIKFMASDILQPLPVKIILLVTLLALPVGSNVLMIYFVLYVIDIREFIDYAPVLFGGFYP<br>SLAILIAVFKGKLIHNLQDEIKLWAIDSAGEKIHSKIKLKVRMVTIFAICNTLLLIVATVHNLIPLPRDLHIYFVL<br>RLIYDYFPNHKTYLLILMKLMSPVTTYMLLVHAYQILYYTQHINIQIQLYNKFVADVDFWETPLCEPELFYNEL<br>YQKRVEKRLKFCIQRSQHFVYVHVAKIKEIGILIALFAVCGVLMGIGISFYLFSGNLTPEYYIRIFIIALVGATTFS<br>SIIWGGQSTETIVTEMIATISQVRWYNFSQTNKKLYLILLTNMMKERKIKFTENYSINYQLGLAIVRGIYSVMSV<br>LVKMYSINT
AlucOrco	MDIFDAPYYRIMKNSAKLIGQWPYQSREKRIIIIIIVWAFFFMQFVPQIIAIVVHIDDPDILFEACSNMAVDFVT<br>AIKYINTICKTNLIKKLHDRVIIDWSLMLNDEEKSTLEKHTNLGYLFSSGWAGFAYMSATIFVLEPVFPRILNV<br>FISVNATDPFKLALPLEYIIIDREKHYWIMLFVSTVFVYNIIIVLVSCDIMYITFVQHVCGLFAVVGCRLVNTPIN<br>ENYSERHKAGDYLSNSKDIPYKHLVSCIRSHRRALEFAELLEDAYCISFGLTVGLNLPVISVTGFQIITQFNTIQ<br>QLLKYASFTITEILHLFFECFMSQRLTDMSLEMQKSIAEVQWFDNSIKSRKLLIIMTMRCQVPCKLTAAKIMDL<br>TIENFGMMVKTSGSYFTMLLSMQ
BmorOR1	MDIPKFEELLKQIKMNFWLMGIPFDNPKIQIRYYVLLLPLSLMLIEEIAFFGSRMSSENFLELTQLAPCICIGVLS<br>VLKILALTAKRQKIYELTQNLECLHKIILNDTRKTELVRKNLVLIKFITKYFFVLNAVLIFVYNFSSPVIIAYNYIV<br>SNEVQFVLPYAVLLPFKTDSWIPWLIVYVYSIFCGFTCVLYYATVDVLYCVMTSLVCNNFSLISFKLQKVNRNT<br>AHLLKEVVKEQQYVLKLAEDLENIFTAPNLFNVLIGSVEICALGFNLMIGDLTQIPGCILFLSSVLLQILIMSVF<br>GENLISESSRIAEAAFLCKWYEMDQKSKKTILTIMIRSHKPKKLTAYKFSVISYGSFSKIISTSWSYFTILRTMYTP<br>PGTKFQDDL
BmorOR10	MDIQNEKNQVLNIKLTKLIGLYQILDPGTLKCRGRNVYHIVLAFIVVYMFLISMMFNISGAYYWTDNKLMSV<br>DYFWKAENTLFLIYKMCIIVYHSDDIWDVCMSITRYDFTSFSHRDRHILDRWRERSVWLTTMFTIMYSSTTVF<br>YLGISLAFRNYTLPVKNHDGSIGFYCQNVMNFYFIASDETYNTHYFTFYFAEALFVSLSFMSYVVISTLLITLCF<br>AMCCQMQMIYNAFESVGQKLICDPHSPIDNTDKKINVPNEHDLVYVELKTIVMDHQAVMEKYEHFLNMF<br>RLVILSQIFVSSVSIITLWFLFIMSFSNDDKYKDSDSVFKKCVFITLGILCEIFILCYLFGKLHDQKDSIIFGLYSSN<br>WTEMDMKCKKLILLTMKMNNANQKKLKFTRTKIVNLEMFFKTMSDCYSIISVLINCIYRNVK
BmorOR11	MDIQRFLKFYKVGWKTYRDPLMEASHSSIYYWREQMKAMALFTTTEERLLPYRSKWHTLVYIQMVIFFASM<br>SFGLTESMGDHVQMGRDLAFILGAFFIIFKTYYFCWYGDELDQVISDLDALHPWAQKGPNPVEYQTGKRWY<br>FVMAFFLATSWSFFLCILLLLLITSPMWVHQQNLPFHAAFPFQWHEKSLHPISHAIIYLFQSYFAVYCLTWLL<br>CIEGLSICIYAEITFGIEVLCLELRQIHRHNYGLQELRMETNRLVKLHQKIVEILDRTNDVFHGTLIMQMGVNF<br>SLVSLSVLEAVEARKDPKVVAQFAVLMLLALGHLSMWSYCGDQLSQKSLQISEAAYEAYDPTKGSKDVYRDL<br>CVIIRRGQDPLIMRASPFPSFNLINYSAILNQCYGILTFLLKTLD
BmorOR12	MDIRGNVHRFVKFYIDGWKHFRDPTMESSYSAVYYWREQMKAMFLYTTSKERQMPYRSSWHTLVIIQATVC<br>FLTMCYGVTESLGDKVQMGRDIAFIIGFFYIAFKIYYFQWYGDELDEVVEALETFHPWAQKGPGAVDYRTAK<br>RWYFTLAFFLASSWLVFLCIFILLLITSPLWVHQQILPLHAAFPFQWHEKSIHPISHAFIYLFQTWNVMYFLTW

	LVCIEGLSVSIYVEITFAIEVLCLELRHLHQRCHGYEQLRLETNRLVQFHQKIVHILDHTNKVFHGTLIMQMG<br>VNFFLVSLSVLEAMEARKDPKVVAQFAVLMLLALGHLSMWSYFGDLLSQKSLTISEAAYEAYDPIKGSKDVY<br>RDLCLIIRRGQEPLIMRASPFPSFNFINYSAILNQCYGILTFLLKTLD
BmorOR13	MDIRNEKNNIFNLKLANLTGLYQMIDPGTAQFRGRNIYHIGMVCVLLYVCPITIILILSGLYYWTVNIPVSIDFL<br>WKSLITLFIIYKGWFVVRYSDDIWNSLSITRVEFSSLGNRNTHIVNHWRERFVWLSTIFLRINFMVLFSVAIGPL<br>AFSGYVQIENHDGSIGYYRECVANLYVTVSDEMYNAHYYTFYFIELLFFVLNLAIYFMFDILLFLVCLGMCCQ<br>MEIICSAFELVGHKPHRDTHSPIDNQEENKKITLNEHNILIYDELKKIIIQHQAIMEKYEDFLTRYRPVMLLQIF<br>VSSFLAIMLWITFIMKDSIVFALYSSNWTEMDMKCKKLILLAMKLNNANYKKLKFTRTKIVNLEMFFKPLVIG<br>KSKKPRCFKNMDISSLPVIWKFNKKAWMTTEIMEQWLRYFNADMRSQNRNVLIFLDNAACHPKIELSNTKI<br>LMLPPNTTSITQPMDQGVIYTFKSYYRKFLLQSLLCKMDNCSSAHQLAKSISVLDAVNWIALACDNSHECM<br>MDMDYGYGLSSLAIINIEMHQTLEIVED
BmorOR14	MDIRNEKNYVFNIKLAKFTGLYQILDPGTVKCQGRNIYHIVIAFFLVYMFAISMILNFSGLYYWTVNIPISIDYF<br>WKSETTLYVIYKIWIVVHHSNDIWNCLSITRYCFTSSSNRNRHIMILDRWQKRSVSLTALFAIMYSISTITYMVI<br>TLAFSEDISPVKNHDGSVGYYRQNLMNFYLIVTDETYNAHYYMFYFAEALYLIFFAMSFLIFDILLVTLCFGM<br>HCQLELICCAFESVGHRSISDSNSPIDYTDEYNKIPNEHDIIYDELKTVIMDHQAVMEKYEKFITLFRRVMLSQ<br>IFVSSLSVIMLWFIFIMSFSSDDRFQASDVVIKKMFCSIPSLLFQIFMVCYLFGNLHSQQDSIIFALYSSNWTEMD<br>MKCKKLILLTMKLNNAYYKKLKFTRTKIVNLEMFFKTMGDCYSIISVLVNYIQRKVE
BmorOR15	MDIRNENNHVFNIGLAKLSGLYQMLDPGTVKFRGQNVYQIFVGFFVLFSFVGAMTLIAGSLYYWTDNTSVTI<br>WYFWSTTNSLYACYKMCTVFYRSNDIWNCLSITRYGFTSLSSRKRNGHDILDRWRARSVWYTSLLSGAYCWS<br>LVFYAGCLLAFGDATIPIKNHDGSIGNYRPNVLNLYFIASDETYNEYYNTFFVVETLFVASITIIYLLFDVLLLTL<br>CLAICCQMQIICSAFESVNRKSLSDSHSNAVDNTHEKQNVSNEHDLIYYDELIKIIIDHQAVIKNFVLFSTTFER<br>VMLSHIFVSSISLIIIWFNLIMSFSDDGKFEISGATTVKTIVAIPSFSFQIFMTCYLFENLHNQKDSIIFALYSSNWT<br>EMDMKSKKLILIAMQLNNANQKKLRFTRTRIVNLEMFFKTMGHCYTVVSVMIHYINAKND
BmorOR16	MDISKVDSTRALVNHWRIFRIMGIHPPGKRTFWGRHYTAYSMVWNVTFHICIWVSFSVNLLQSNSLETFCES<br>LCVTMPHTLYMLKLINVRRMRGEMISSHWLLRLLDKRLGCADERQIIMAGIERAEFIFRTIFRGLACTVVLGII<br>YISASSEPTLMYPTWIPWNWKDSTSAYLATAMLHTTALMANATLVLNLSSYPGTYLILVSVHTKALALRVSK<br>LGYGAPLPAVRMQAILVGYIHDHQIILRLFKSLERSLSMTCFLQFFSTACAQCTICYFLLFGNVGIMRFMNML<br>FLLVILTTETLLLCYTAELPCKEGESLLTAVYSCNWLSQSVNFRRLLLLMLARCQIPMILVSGVIVPISMKTFTV<br>MIKGAYTMLTLLNEIRKTSLE
BmorOR17	MDISKVDSTRALVNHWRIFRIMGIHPPGKRTFWGRHYTAYSMVWNVTFHICIWVSFSVNLLQSNSLETFCES<br>LCVTMPHTLYMLKLINVRRMRGQMISSHWLLRLLDKRLGCDDERQIIMAGIERAEFIFRTIFRGLACTVVLGI<br>IYISASSEPTLMYPTWIPWNWRDSTSAYLATAMLHTTALMANATLVLNLSSYPGTYLILVSVHTKALALRVSK<br>LGYGAPLPAVRMQAILVGYIHDHQIILRLFKSLERSLSMTCFLQFFSTACAQCTICYFLLFGNVGIMRFMNML<br>FLLVILTTETLLLCYTAELPCKEGESLLTAVYSCNWLSQSVNFRRLLLLMLARCQIPMILVSGVIVPISMKTFTV<br>MIKGAYTMLTLLNEIRKTSLE
BmorOR18	MDITEEELEAVVPCMARSGLLGYWRSSASREGAGSYLRGFLSCCLITCISLSAAERLLTDTPSDLAELTMTAFE<br>LTVPLTVVSKGLFFILQRDTIHELVDLLVDMRRRYAERDDGPNRRRACYLYVLAVQRVLLVMALLIIGGWLA<br>GPMLPHVFSFASQNESSVPWQTPLPLWLPVDLQRSPLYEALYLFQGLCVLTSLTSASALDACFCNMMLMIAA<br>ELQVLNDNISSPSGNETVVDKGESESITLEVHSELESVVPQFKSGATGDAGLSKTGRSRPRNQTSLRL
BmorOR19	MDIWDQKNHVFNVRLAKVTGLFQILDPRTTKFRGRNLYHIVMAIIMLYVCVISVILAISSLYYWPYSIIVSVDY<br>GWKGLMTLFLVHKMWNVVYHSNGIWNCLSITRYDFTSHSLRNRHVLDSWRDRSVRITTIMTVAYSTSSIVFA<br>ASSLIFRDDIMTVKNPNGSVGNYRQNLFNLYLIVTDQTYNAHYDTFYIVEVLYTVFLSILFFMFDFILVTLCLA<br>VTCQMQMVNVSFESAGHKSVYDPTIDNTDEKICLSNEHDLIYDELISIIMDHQAVMKKYGELLTLFKPLMVL<br>QVFWSSLSLIMVWFSFIMSFFGQDRFVASEVTTIKLICLIPSISFQIFLVCSLFTNLHNQKDSIIFALYSSNWTEMN

	IKCRKLILLTMRMNNTNQKKLKFTGTKIVNLELFYKIMSHCYSVVSVLINCIKAKNE
BmorOR2	MDIYERPYYKISKNFASFIGQWPYQSRLHSFMCGSVLWTLFIIQVIPQIIAAVVNSDDQELLLESVSPFITDGIYI<br>AKYVNTIRKAKMIRRLFEKVREDWKVPKNNDEKLVLESYLKMGRFLSIGYAAFVNMGVIIYIMDPVLSAIVN<br>IISKSNDSMPLKFSVPMRFIMFDEEKYYWLLLILSNTCVIFIINVIICCDVIFITVVQHVCGIFAVVGFRLEHSPSD<br>TVSPDLIEGTRFSMNSQDISYKHFVSCIRDHRRALEFSELIESTFAISFGISVGLNLPLMSITGVQLLTQSESMRA<br>TLKYIMFTGGQILHLFFDCYMSQKLTDMSSRIQHSVARANWYENSVKSRKLLILMTLRSQVPCKLTAGKIME<br>LSIENFGMMMKTAGSYFTVFLSMR
BmorOR20	MDKHKDRIESMRLILQVMQLFGLWPWSLKSEEEWTFTGFVKRNYRFLLHLPITFTFIGLMWLEAFISSNLEQ<br>AGQVLYMSITEMALVVKILSIWHYRTEAWRLMYELQHAPDYQLHNQEEVDFWRREQRFFKWFFYIYILISLG<br>VVYSGCTGVLFLEGYELPFAYYVPFEWQNERRYWFAYGYDMAGMTLTCISNITLDTLGCYFLFHISLLYRLLG<br>LRLRETKNMKNDTIFGQQLRAIFIMHQRIRSLTLTCQRIVSPYILSQIILSALIICFSGYRLQHVGIRDNPGQFIS<br>MLQFVSVMILQIYLPCYYGNEITVYANQLTNEVYHTNWLECRPPIRKLLNAYMEHLKKPVTIRAGNFFAVGL<br>PIFVKTINNAYSFLALLLNVSN
BmorOR21	MDKLRVEEFSINLELMKRFRFYHIFNPNVTTIFNFNAYRLLLFLCGSIMIMIIVYSTLGFFVEMDDTLSYIDFFV<br>VIFVMIVLFLCYWRICIFLYNADAIHDLFSISRIDFLNSKHCCKNVNVLYDNRDKSIKISNYFFLLSTTVMSQWI<br>IFPLVVIAFTKPEDENTRFQNILNLRYPVSTHTFNQYYYIFYLIEVAVAVFTMYVMIMPDILLMSFCWVIMAQQ<br>EVLIRAFKNIGYEENSQIVYYEDFKSILGDQLQLNLKIKLFYSVVRSIILTYVAIISTTFIMVTYVLILVCLSKESHP<br>VLNIIKLGSSAIYMCLLLFLYCYLFDSMNIKRQSINSGIYSCDWTKMDVSFKKLLLLTMQMNNANNLVIKASP<br>TKIVDLQLFANIITMSYNIVSVMLKAVETTS
BmorOR22	MDKRDDPFIILRKMIFIEAKNCKIAKFCDAFLILFYSLVQLLDIYYMSKNFSISLLIRYSPITIMYLLIIIAAVISVGL<br>DKEIIEAYTVCCKIRWPMNVVKKQTQIKLKKKCQIINAGLSCTVPLFLVTIISTFPYFGSERDLFICVEVFEAYFG<br>EWSFIPYYFCFAASPFFYYHFFRITFVLVYAFLHAQLQYLLIEEYLFETYETDEAKGWKYLQDTRYQQEIGKSL<br>QLCISQHIALKQFVKKTVDLVLIGMPFFLVFGVLLLTSLLAFITNFEDITSNILKIRILLAAGCSLCITIVFCWIGQ<br>QLINVTSDIFFSLGGASWYFWNRDNMKTLLMFLINCTENESVVFAGICLNYELFLSVVRLTVSYTLVLYNLQK<br>Q
BmorOR23	MDKVEFSDPLFFLNVIGMHPFKADKFSKFRLAFSIAVYFAVIFSGVLELIVNSQGLETYARASDTLIPQCQLVC<br>KIFVLAKYKKQIARLLNGSQRFWDLGQFGARYGNSFGKTHKYLKSFFLLYKVMLTFTCLQFLAVKIIFKIPKPI<br>AISFGETKGLEPLYDHLYLVLHAMITLVTINLVNGFDGLFFYFIGHVLTELKMVKVAFGDSPIETNWSEEKRFK<br>FAVRHHRFVLDFIEQFNIVYCTMLLVQHLTCLFGICFGVFLMTKDGVPPDLDRASKYLPYIVTFIFQTFTFCFA<br>GNLLLSWSLEIPNEIFYHDWAKKTTYENKLAKIISMKRGQRAARLTLGGFANLDLDSFRMVLKNALSFFTFV<br>NAMMNKKAVTSV
BmorOR24	MDKYDWRSHIKINILVLRFLGLWPKNTYHGFYIFHLIFMMGTFLLGHLFFQAANIYFIRTNLEAVTGTIYVLL<br>VESLVVFKVYHLVKNMAMFKQLLEILDTEMFQPKNKKQIVAIDETIHVWKTIYKSFLYTCFGTNAFWAIYPLL<br>DKSEGGKRLPFLAWYPYNTTITPLYEITYVYQIVSVSFITTVHVNVDVLVAALNIFNGSQFEILCDNLKNLHN<br>GPVKENLIECIKHHKEILKFAERCNNFLNWILLVQFFIFAVSIGITMFQLTLVIPFSTEFYSLLTYGMAIILQIYMY<br>CWFGNEVEIKSNKIPYAAFECNWVDFSPEVKKNLIFFIMRAQKPVKLSALNLFYLTLDTFMMIIKTSWSYFAL<br>LHQVSSRK
BmorOR25	MDLKPRVIRSEDIYRTYWLYWHLLGLESNFFLNRLLDLVITIFVTIWYPIHLILGLFMERSLGDVCKGLPITAAC<br>FFASFKFICFRFKLSEIKEIEILFKELDQRALSREECEFFNQNTRREANFIWKSFIVAYGLSNISAIASVLFGGGHK<br>LLYPAWFPYDVQATELIFWLSVTYQIAGVSLAILQNLANDSYPPMTFCVVAGHVRLLAMRLSRIGQGPEETIY<br>LTGKQLIESIEDHRKLMKIVELLRSTMNISQLGQFISSGVNISITLVNILFFADNNFAITYYGVYFLSMVLELFPC<br>CYYGTLISVEMNQLTYAIYSSNWMSMNRSYSRILLIFMQLTLAEVQIKAGGMIGIGMNAFFATVRLAYSFFTL<br>AMSLR

BmorOR27	MDLRRWFPTLYTQSKDSPVRSRDATLYLLRCVFLMGVRKPPAKFFVAYVLWSFALNFCSTFYQPIGFLTGYISH<br>LSEFSPGEFLTSLQVAFNAWSCSTKVLIVWALVKRFDEANNLLDEMDRRITDPGERLQIHRAVSLSNRIFFFFM<br>AVYMVYATNTFLSAIFIGRPPYQNYYPFLDWRSSTLHLALQAGLEYFAMAGACFQDVCVDCYPVNFVLVLR<br>AHMSIFAERLRRLGTYPYESQEQKYERLVQCIQDHKVILRFVDCLRPVISGTIFVQFLVVGLVLGFTLINIVLFA<br>NLGSAIAALSFMAAVLLETTPFCILCNYLTEDCYKLADALFQSNWIDEEKRYQKTLMYFLQKLQQPITFMAM<br>NVFPISVGTNISVTKFSFSVFTLVKQMNISEKLAKSEMEE
BmorOR29	MDNSLDSFLRINRLFLSSLGQWPQQEQISKIFTLINAVFFLITQAYFQTGGMIAAKCDQPIFMESIAPVLISFMC<br>LVKFVNFNYNADKMRKLLEIIQADWNSINDLEELKILNSWAKDSRKNTIMYAGALYGTMAPFMLGPLVPIF<br>CKLMPAGVLPANSSIVLEKPVLFHVEYFYDLEKYYYPLLIHSYFGTMAYMTVAVAIDSMFMVYVQHACAIFA<br>VIGNRLEHLADDSSINFYYNPHILNDEPYKRMIECIVQHSKALQYAQMIQSANSLSFFFNWDSICSLLLSVDF<br>NWPSQRLADESARISETTTRCAWYLTSMRSRKLLQLFIMRSSVPCQLTAGSFYTLNMQNFSAVVRTSMSYFTV<br>LTSVQ
BmorOR3	MDNVAEMPEEKYVEVDDFLRLAVKFYNTLGIDPYETGRKRTIWFQIYFALNMFNMVFSFYAEVATLVDRLR<br>DNENFLESCILLSYVSFVVMGLSKIGAVMKKKPKMTALVRQLETCFPSPSAKVQEEYAVKSWLKRCHIYTKGF<br>GGLFMIMYFAHALIPLFIYFIQRVLLHYPDAKQIMPFYQLEPWEFRDSWLFYPSYFHQSSAGYTATCGSIAGD<br>LMIFAVVLQVIMHYERLAKVLREFKIQAHNAPNGAKEDIRKLQSLVANHIDILRLTDLMNEVFGIPLLLNFIA<br>SALLVCLVGVQLTIALSPEYFCKQMLFLISVLLEVYLLCSFSQRLIDASENVGHAAYDMDWLGSDKRFKKILIF<br>ISMRSQKPVCLKATVVLDLSMPTMSIFLGMSYKFFCAVRTMYQ
BmorOR30	MDQRATSQSEEEEWAGQSVVRTNTHLLRLLGLWRPARSRLYDAYTAAVLAVGVADLALASAGLWLRPGGL<br>AEVTLGLANLFVILTALSKSVLLLGRRPLFYELVRRVDGATAAQRPFCGEDPLLARLSADARARADRLSRAM<br>HWYVVFAALSWSAVPLLAPPGDRVWPFQQLPPRPWARSPLYEASYALQVAGTTYFALINMDSDCFFMAVM<br>THVSLQFRILASRFAKLNSTEESLADKKASDVRVTSSESTLPVDDTDRELRACIQTHQKLLRLVNFLNDVMSP<br>MAMMQLALGVINSCMVLFPATYSEDSSDVMKCWGALPLLAIQVFLYCSGAQRLADQLVQATYSYYTLLQH<br>FNSH
BmorOR33	MDQVLEKFPENDWLRGVKFISSDIFQRKLVKAVLFMVLLVHLTASVITIRAILIKDITAKEFTFYGPVFFGCFYG<br>MLAIYIILFEKNFIANLSGELKMWSFRSAGAEITRQIRFESRVVTIYAIINFVMVVIASCLHITPLESDYETFYMIR<br>FFEDKIPDYANVCKTSYRSTFLVMGYVMMVHVYQIIYATQHGKFQIMLYLEYVKRVTQFNEKIGEKCLFYNE<br>SFQKMVARKLKNCVIRHNEFLKYHRKNTREMSHWIVAFSLCGCLLGISVFFYILSGVIYREQYFRVAVLLTTA<br>ASTFVAFIVAGQSLESRVDNGYSVVSRIEWYNFSETNKKTYFLLLVMLMQPWKIKFSDKYSINYELGLSIVRGI<br>YSIISVMVNIRFDS
BmorOR34	MDRHEGPLSTHFRRLFRLVGVYNGKYITPHSCLFFFSGFVNIIYLSYILIFTDCNMTKVAHFIILQYFYIGTLWT<br>VVYKGNDIIWIANECDKFVGLDGHHFERLYDEVREQEKTSPATKGKTIIDRIASIICTWTCIEPFINAWTGKAE<br>LEFPFTGTNADTTKFIFIYLMQCSLMFIVAIVCSVIFKSLMGIALDLVIKYKVIGLVLSSLNEQMMNHRNVYKS<br>DLHQTIRKCVQSHHHVLRIFEKYRDICTYGFRYSYVGLIGATSLSRSLLSSDDPDLGTIPHIIAELSYMGFFCYIL<br>NQVEQEHDKLKDAVFAADWPWLPKPATSSLRLIMMRTAKTPRVILVKGGGPANLETFYKLLNGTCGYLIFG<br>LVLDQAAF
BmorOR35	MDSFLQVQKSTIALLGFDLFSENREMWKRPYRAMNVFSIAAIFPFILAAVLHNWKNVLLLADAMVALLITIL<br>GLFKFSMILYLRRDFKRLIDKFRLLMSNEAEQGEEYAEILNAANKQDQRMCTLFRTCFLLAWALNSVLPLVR<br>MGLSYWLAGHAEPELPFPCLFPWNIHIIRNYVLSFIWSAFASTGVVLPAVSLDTIFCSFTSNLCAFFKIAQYKV<br>VRFKGGSLKESQATLNKVFALYQTSLDMCNDLNQCYQPIICAQFFISSLQLCMLGYLFSITFAQTEGVYYASFI<br>ATIIIQAYIYCYCGENLKTESASFEWAIYDSPWHESLGAGGASTSICRSLLISMMRAHRGFRITGYFFEANMEA<br>FSSIVRTAMSYITMLRSFS
BmorOR36	MDSKQEKQYIFNMKLARIICLYQILIPNSTSIFGYNIYHIVIVIFGSFMFAISMLFPIGLLYLRNDIIAIMYYMGCIS<br>NFLLSSFKMGNILYHAKDIWKCIDVTSFNYISYKHYDRNVFKNWQTRSIRITYIYIVIALSAFFCWIFSPSVMN

	KSIITIRNIDGSYSKYRMNIFNIYLIASHETYNKYFYIFYAIEIIISICYVYFTIVFDVLMLLLCFAISYQLETISNTIRSLGHEICTRDNFRTLHEKHGIIYNDLITIMTDHQNVLKKLNDFYNIFRSITLTQIFIASSSHVFIWFIAAMSIDEGDNADSILSFKLFIVLPLINFQLFMTCSLFGTIHEKKDSIIFALYSSNWTDMDLKSKKMILFNLTLNNANQLKMKYTNTKIVNLEMFSHTMRFCYSIFSMLINYNKNKMK
BmorOR37	MDSRRKVRSENLYKTYWLYWRLLGVEGDYPFRRLVDFTITSFITILFPVHLILGMYKKPQIQVFRSLHFTSECLFCSYKFFCFRWKLKEIKTIEGLLQDLDSRVESEEERNYFNQNPSRVARMLSKSYLVAAISAIITATVAGLFSTGRNLMYLGWFPYDFQATAAIYWISFSYQAIGSSLLILENLANDSYPPITFCVVSGHVRLLIMRLSRIGHDVKLSSSENTRKLIEGIQDHRKLMKIIRLLRSTLHLSQLGQFLSSGINISITLINILFFAENNFAMLYYAVFFAAMLIELFPSCYYGILMTMEFDKLPYAIFSSNWLKMDKRYNRSLIILMQLTLVPVNIKAGGIVGIDMSAFFATVRMAYSFYTLALSFRV
BmorOR38	MDTFCGGFMGKVYYLKKHNIYINVYLSCRVNRDNFSSFYRDFRYFLSQFYHLMAALSAALLGGSVCIVFIAIAEHLLAQLEILCISFRNAIGFIPAPGDRVGEKLAYQRVKSCLQHHNIILKFFDEFQKYYSIPLFCMLAGTTVAMCTIAFVVTDPSSTFGVSAAFLSLMAPEVAFCICYCTYGQKITDMSDILRDTVYNAPWYYQPKPVKMALLMALNKTRTPMTLSAAGLKDCSIKSIGEITQTTYTYFNALQLFRGKPAYHRE
BmorOR39	MDTNPSAAGDSVAPHLRRLRQVGFCQLDPTSQSRRPILALMHRVYHRLVLAATVLYIFEQLTYAYQARNDMERLSRVLFLMLCHLTCIAKQFVFHSDADKINQLVVGLDDALCNQPVETHRLLLLETSRRAARLLMLYSGCAVSTCILWAVFPLLDQLRGRTVEFAFWIPIDYRHNAFQFAVVLAYAFYSTSLVAVANTTMDAFIATVLYQCTTQLRILRMNFESLPERAYALSRKTRQDYHTVTHELLVDCLLHYKKITETCNLLEQIFGKAILVQFGVGGWILCMAAYQIVDMEILSIEFASTALFMGCILTELFLYCYYGNEVTVQSGLVSESVYAMSWLSLCPRERRALVVVLERARRPLRPAAGRVVPLTLNTYLKILKSSYSFYAVLRQTK
BmorOR4	MDVFDSMHWRTTKKLLSAIGLWPFQPVIQRIVVGAIVYFIIQSIFICVFLKLIVAWGNLAETLYSVPILVYFSMIQVKITNCHLNLHKAKFLLLRVKRDWESKLEDSEFEILRNDGRIHKIIMDVYFSGLICVATVYIVLPLMSPLLDIIIPLNETRERILPYPAEYFIDIQKNFFMLYPHGAIVTPIALTVLVGFDSLYAGFVQHACSMFTIIGRRLENLTVDRNNIDEEKNSLNERHGLQSFITCVKMHKDILQFVKLVEKYYSNYFFVLLGVIVVGLSAAGFQFVVLLSGVGEKVRCLWYAMGQVTHLFFLSYVGQKLIDHSQVINASLSAAKWYDYPQKMKPLIILMLMRGKRVSTVSAGKIYVMSIENFSSVIKASMSYFAVLTSMES
BmorOR40	MDVFEEPFYKMIKNFSHLIGQWPYQSSRKKFTIVTLIWIAFFMQFIPQIIAIVIHFDDRDVLFEAFSSMVIDFAFIIKYLNAIYRAGLMKELWESIRRDWTLLLNDVEKRTLQHHANLGNFFSMGYAGLAYMSTTIFVTEPIFPRIVNIFVETNETIPLKLALPLEYIIIDIDKHYWLILTITNIFVFNIIIVIISCDIVLITFVQHVCGLFAVVGCRLESTPFDENYLEGQKGEDFLSNSNDIPYKHLVSCIKGHKRALEYAERLERAYTLSNGIVSGLNAPVMSITGFLMITESSTIEQLLKYATFAISQMSHLFFLCFMSQRLADMSLRIQENIGNATWYNNSLKSQKLLVLMLMRSQVPCKLTAAKLMDLFIENFAVVVKTFASYITMLLSM
BmorOR41	MDVLQENFSVLFYLGVWKPLDCTGIKSFLYNLYTLFITSISYTFLLSQILDLIISTKTVSDFTNNIFIVSAILTGCLKIFRFIRSRNTFINIINNFKRGLFKPANNDEIIIWNKYARITRLVTIGATTSLIIGLIVMSYALCSFNIPQRQLLYRAWLPYNYSSLPIIYWLSSMEQLATVHILAGINFSFDLIFFGTMLNICAQINILKLRYKVALSHIYSINDSINNNDLGELRDVSKLIREYTDSHDSIIKLFNSAHHLFSTIVTIQYCTSSVAICTSAFNVTKMKFFSFQFFSTALYINNVMIELFILCVSCNEVTLEFADLGNTFYDCQWYAINNANKKSVAIMMTNTIKPIYFTCGYVIHLSLDSFTSVLKLSYSIYNVLQSAD
BmorOR42	MDVRNEKNYVFNIKLAKFIGLYRILDPGTVKCRGRNVYHIIMACILVYMFAISMILNLNGLYYWTVNIPISIDYFWKSETTLYVIYKIWIVVRHSNDIWNCLSITRYCFTSSSNQNRHIILDRWRERSVSLTTIYAIMYSMSTITYMVITLAFSEDVSPVKNHDGSVGYYRHNIMNFYLIVSDETYNTHFYIFYIAEALYLIFLTISFLIFDILLVTLCFGMGCQLQLICCASESIGHKKLSDSNSPIDYTDEYNKIPNEHDIIYDDLKTVIMDHQAVMEKYEKFILLFRRVMLLQIVVSSLSVITLWFIFIMSFSNDDRFKASEVVIKKMFCSIPPLLFQIFMVCYLFGNLHNQQDSIIFALYSSNWTEMDMKCKKLISLTMKLNNAYYKKLKFTRTKIVNLEMFFKTMGDCYSIISVLVNYIERKVE

BmorOR44	MDVSFAAVSVMRAGLPAEESGGNRRARRVTQWSLALWLGTTCSWMLAALLRLDLPFFAWFPFDTTHHYAE<br>AFIYQLVTANLVVVIISGLDCFCLELMMHLSERLQTLNKLFRSFATDNARQQPQLRMQPSTPASHRGKSPYGI<br>ITKKSFMKWVVAPLDVHRKPAKAINSLYSRHDSPNVGNANGSFKHCIQYHWELIELKKETEKFCGVVLFFQI<br>LASMFIICFVTFQATVNTMDAGSLTKCVMYLSVALLQLGLFCNEGTNIVTQSEELMLAVYSSEWPDCDAALK<br>QSVIVTMMRLQYPLQIRAASYCTLSFETFSKILHTSYTFFTLLRQVSETQ
BmorOR45	MDWDPKETEPLTWQYTTHSVLKYDLRILHLLCLWPLPGSLFFRLLTAFLIALCLGHFVEGLVNLCTLSGDME<br>DYTLALSNISVVTIGTVKTAFFLRNERKYFRLVRWLDALVAAEKKSVSSRPLSEAIFPAAQKRSARVAAFLLLY<br>NCFLLFIWLTAPLAARPEARILPLQQLPLTDSNAYPLYELSYAMQALSIFFIGLINVHLDCFFTVAMIQTAALLK<br>SLASRLADLQVRNAPSRRNVDEGRKNIVTADDMYRELCLCIRTHQEITRFVQHLENVMNPIAMMQLALGV<br>FDGCMLIFPAAYSSETSALVKCFGAAPTVCMQLLLYCLGAHSVREQGESVSVAAYSSGWADASARFRRAVQV<br>VITRAQKPLVLTAGGIYPIQRATFLSLLNAGYSYYALLQNFNGR
BmorOR46	MDYDRIRPVRFLTGVLKWWRLWPRKESVSTPDWTNWQAYALHVPFTFLFVLLLWLEAIKSRDIQHTADVLL<br>ICLTTTALGGKVINIWKYAHVAQGILSEWSTWDLFELRSKQEVDMWRFEHRRFNRVFMFYCLCSAGVIPFIVI<br>QPLFDIPNRLPFWMWTPFDWQQPVLFWYAFIYQATTIPIACACNVTMDAVNWYLMLHLSLCLRMLGQRLS<br>KLQHDDKDLREKFLELIHLHQRLKQQALSIEIFISKSTFTQILVSSLIICFTIYSMQMSPVLQDLPGFAAMMQYL<br>VAMIMQVMLPTIYGNAVIDSANMLTDSMYNSDWPDMNCRMRRLVLMFMVYLNRPVTLKAGGFFHIGLPL<br>FTKTMNQAYSLLALLLNMNQ
BmorOR47	MDYLSSPYCRLNKILLSCLGEWPYQTSTQRRFIRSTIYFFSASIIIPKIIKLIKVWGNLDMIIECIPMLLLDAVNFV<br>KVVNGFINFRKMRELFDRIQDDWGLNYSKREFEIMQNYAEDGKKLSQFYASYMYATMLIYFCMPIIPKVLDI<br>VLPLNTTRPELYLFEAEYFVDQHKFYYPILIHAYITCAVAVSMLVAFDTEYAIQALHGSGIFSALRYKLENLVIK<br>DDEADYKNDEKIKQSTYNMVVQCAVLHKRALDYADLLESSRVTCFFFVLLVNIAAISITGVQTVMKLDQPTE<br>AIRFGVYTLAQITHIFYNSYPAQMLFDNSWKTSDAIFAGNWYRAGSKSKNLLHMMIMRSRIPCKLTAGKIYL<br>MSLENFTGVVKTSMSYFTVLLSFR
BmorOR5	MDYQLGDHGCGYPLRCITDRSACDGVRSDEPGCTNGKTSFFSDESRFCLRHHDGGIRVWRHSGKRTLQACI<br>RHRHTCVSPGVMVWVAIGYTSRSPLIRIDGTKRQEELCLATDSSYIVDNGIETLAVLWGFEKFRYFLYGCKTRE<br>YTAHSLDLSPTENVLSMVVEQFARHNPPVTTLDELWYRVEAAWASVPVHAIKVLFDSMPRRIKAVITARGAV<br>LTRLCPVVPQYPIIALYPWPVQSGPAYALTFSLQVLCGGLFTMTHLACDTFLLSLLIYICSQIDVLCASLRQLGR<br>RLLRIVSLVSYRYVGVLQQVLSPVALAQFMCSMVIICLSGFGIAISNDFGSLCRYSVYFTGAAIQLLLFCWYGE<br>VLITKSEHVSEAAMACGWPAVRRGRFQSSALLLMVRAQRPLALTGSKFYVVSLKTFVQVTNAIITYFKIAPTL<br>GK
BmorOR53	MDYSEKSLMQGDCLKLLKVISSDIFQPKLVKLILLIVFGVHLIVDLLTLRALLVNELDFKEFVFYGPVFFGSFYG<br>MMALLTLVLKDDFISNLKQEFRLWPLDCAGDEIYSQIKFENKIIKIFVVFNCIVTFIGSYLYFLPLDSDNETFYA<br>VRFIEENYPDHRNLLHGLYRSTFLIFGYAMTVHVYQVIYNSQHLRYQIIIFTEYVASIGNPDKRKENELFYDKG<br>FQKVVYERLKFCIMRHQEFLVISNKKVGDMRVFIVGYSLCGCLLGISLTFYIFSGKFYREHFPRVAVACVGAVA<br>TFWAVITAGQAIESEYDSALSTLLGKIEWYYFNDSNKKNYLVMLINLMQPWKIKFSEEYAVNYELGLGIVRAI<br>YSIVSVVASMHFEV
BmorOR54	MEAFRLHLFFLSILGVWKPQGWHGIKAFLYSIYGSTVVIFNHIFILSGILNLTKFKHVSLDVFIDNFSQMLALIV<br>VRQRIICVIENRNSISQIIESTDKYPFKLRDRQEKLIFSKFSKLAKNILIYYPIVHMCIILVHTVGHISVMDPPYAL<br>PFQGWFPYNYTRKTKIYWATATYQLYAIFSEGSIDLILDLLLPCILCYMCGHIHILRHRFGVMTEKLQIMSENN<br>EPREKIDSAERKMTAEWVEYHIDILRLVELVKKIFERMIFVQYTVSSLLLCTLAYLLSHTKCTTMTFAANFSFF<br>MAMFIQILLPCYCADKLTFEFLDISTGIYNSNWYQLSNNIRRSVVVILRNTYQPVTITSGFFIILSLESFTKIIKLA<br>YTIYNLLE
BmorOR55	MEALEKGEPTNGELHLINGTFTGPQMAGRSCSSPASYSKYFHDAPVSGHLGFVKTLDWKGFYRSVNHYEAS<br>AANGAESAGRQEAVPRQRSRHPGKIRQDCLHRVETVDGESPRTGVTVTVVLWMGDPLLQMLTSPAGNSSR

	PLIFWVPLEVRHSPAYEITYAVQALGIAAIGQTSILMDIFFVVLLLQAASEIAVLNENIAGMGFKKLRDRENES<br>EEHTSIQRVESSTYVVRTSYVLSECTPHPEISSDNNGNPFAFDRGHKYCKMYSTLVRNIRHHQHIIAYVKDLE<br>VVMSTSLYLLLLANALNVCLHSFGFVALFQEGATRSTVIKEVLSFPSFLGQTALYCFFGQVVIDQADRLHYSA<br>FSCDWPHADEPFRRSLRIFMMQAARPLNVKVGKLVTLSRKTFLQALNTSYTIFNMLFNVERRS
BmorOR56	MEALWWPVSPVRRGLRLLGLWVAPPGRRALHRLALSWVLASHAFLLLVGAASLVMDTPEDLPQLSFTAYTT<br>LTCFGLIAKLVSFSLDGARLTRLLQLLAECRARFPDPGGRRGQHHLMAVRLHRFLQVSYRVNSVVWMFAPV<br>VSAALAARSGGEEPVKRMYVLPLWLPVDTQASPAYEAVYVAQLATGWMLSETTVLLDVALLALMLHAAAE<br>LAVLNDRLRSQPAAAAAGPALASPVAYPKDGDTDPKHDHMYRHMVENIQHHQIIIVYTGLLQSVVRRAISV<br>LLACNTVSICFHIIATVALLQKDIELVGMTKMVVGSTLYAYQTAILCLLGQRITTQSERLSASAYSSAWWEGDG<br>RYQKLVVVFCERASGALSIRVCGLYSLSKETLLQVLKAAYSLFNFMYQAMETSNH
BmorOR57	MEDAAGQSATLLSPCSVALAWLGIWRPPGGRRSGLGLPGAVFIAALDITISSLALVQMLIDRPEDPADFREVF<br>FICSCGVSWSVKVVAFLLQGDRLERMVLSLLDAKTRFPDNGSRVREKYTAMAYTVWRMWQAMPAVTVLL<br>WMADPLLQTIISPPADNATRPLIFWLPVQVHDSPAYEVTYAVEAFFIGTVSETSILMDIFLIILLVYAAGEIAVLN<br>ENVARMGLTMQRETAKAQPVESSSEKASMSKTRYVPSGDSPAADVGAGGKGQLPLLDGDDALWDMYSAL<br>VTNIRHHQAIIAYINDLEVVLSTSIYILLLTNALNVCLHSFGLVAFVIIVFLQLLAEGATSSTVFKEVISFASFLAQ<br>TALFCFFGQLIIDQADRLQFSAFCCDWPDADESFRRSLRIFMARATCPIKVTVGKLVELSRNTFLQALNASYTI<br>FNMLFNLQTSDE
BmorOR58	MEDFRDEEVVINLKLLKQYRFYHMLKFNETKILNCNVYRLILFLYGSIMTCMVVYGSIVLFVEMDDIISEADL<br>FIVIFLTINFFFCVWRICTVLSKSNTICDLINVSRFNYLTSKHCCKHLNVLYDYRERTIKITNYFFVFSMIVLMQ<br>WIIFPILAITFKKSDFENIRSENVMNFRFPVSTHTYNQYFFIFYIMEVAIVTFPIYLIIVMDTLVLSFCCVIIAQQEV<br>LSLAFRNIGHEENSQLEYYEDFKSVLGDQIQLNLKIKSYYSLMRNIILVQVAMSSTFFIMVAYVLIVVCFSKDSN<br>QILTIIKLGSSVIFIGSEIFLYCYLFGSMNLKRESVNFSLYSCDWTKMDNKFKKLLLLTMRMNNANNLMIKASP<br>KKVVDLQMFANVISIAYNVISVMLKSMDSNN
BmorOR59	MEDKLSWTFSGNSALKLNIRHLWLLGTWKLGESRLFKMQSTVAFGLSIWSTVECILAVYFIWGDLEQTTLVL<br>LITCTCSSGVVKMFIFVYDRRRYDSLTLRLDALLSLQTGPCSEDPALAAISDWSRKKASRLTMGLLLFMLSQS<br>MVWYFVPLIAHPEERSLPFVQHQWDNNSLYELSYGVQCLSAVWISQISFSVDCLFASVMILVAAQLEILGQRL<br>INLKNGRDSAEKEEKKQLDSKTGESMYDDLCLCIETHQEILRFVTQLQDTMSPIAMTQFALSVVIACMALFQ<br>ATFSEDFSAVLKCASFLPIPGGQVYLYCWAATNVTEQAEAVSAAAYSCSWVDASERFKRSLRIIISRSQKPLVLT<br>AGHLYPINREAFLTLVNASYSYYALLSQMNNR
BmorOR6	MEDKLSWTFSGNSALKLNIRHLWLLGTWKLGESRLFKMQSTVAFGLSIWSTVECVLAVYFIWGDLEQTTLVL<br>LITFTCGAGVVKMFIFVTTGRRYDSLTLRLDALLSLQTGPCSEDPALPAIADWSRRKASRLTMGLLLFMLSQS<br>MVWYFVPLIAHPEERSLPFVQHPWDDGGLFGLAYGVQCLSAAYVSQISFGVDCLFAAVMILVATQLEILGQR<br>LVNLRNGRRVAGRREKEQLARKTGESMYDDLRLCVETHQEILRFMTQLQDTMSPMAMTQFAVSVVIVCMA<br>LFQATFSEDISAVLKCVLFLPIPTGQVYLYCWAANNVTEQAEAVSAAAYSCNWVDASERFKQSMRIILSRSQK<br>PLVLRAGRLYPINREAFLSLVNASYSYYTLLSQMNDR
BmorOR60	MEDLTQEQKDLNEGAKMFDWGKWISKGIGVWPLAPNDYLFTTTFLYFTAVMTLEWVDLYTCLGDFEKVV<br>DNLTENLAFVHIYVRTLMLRVHIDKLRDVMTESLKDYRTSAFKNSTEIKLFMTHINKGKVFAKVVITFIAMTE<br>VTWYLQPLTTPSPPVDNRDNETISILLPYHFYVFYEINDFKTYVLTYLSHGPHVVISGFGHATSDCFLIILVFHL<br>SGRLAVLAERINALKNKPEMNGIQIKSIIAEHIRLLKMGENIRSAFATALLAYLFNGTILLCMIGYQILVNFMT<br>GPNSDLMQYFIFILATYFIITVFCIVSERLIFESTKVCEAYWNCGWYNMPREHINDIMYCIVRSQKPLALQAGK<br>FAYFGNSTLTDVTRTAMGYLSVLRNFLIVN
BmorOR61	MEEAVEAARGRAPRLLQTLRGDVAAPAHYLAFNVWLCRLVGLLPRPGQPLWATVQPLVWSCLLLHLMCEL<br>VDIALNIADVQQLGKNLPISSLVGGSWYRLSYFTMRRDAYWRLVSKVGESFHRGAPGRMRRWLRRSRGFTL<br>AYFVYGTIVCLFWLGHPLLLQQTTHTMFTSSNSTNRSRMSETAEFPSGAWYPFDVRERRVYGAVYGFQCLAL

	YFAAMLIMVTDIMFITLMLLACGQFEELGDKLRHCWEIATTRALSRAGTTPERELQKVLAHCVRYHDMLLG<br>IVGDIEDLHWTSMLVNFVLQLIILSFLAFEATASADLTNPLKGTNLLMYLVMAIFQLFLLCSCGDRLMEAEEM<br>VARAAYESQWFDAPQGAKRSLSIIVMRARLPQRVTVGKVVGLNLVTFSETLSRAFSYFTVLRQIRTSN
BmorOR63	MEEEEASELLGATAVALQLMGLWRGGGGGVAAQAAVAAPTLLVMGSAVLLSGAKLCAEPPAVYEELIAVIFI<br>LVASVSWTFKATAFVGQRRRLQALAALLVAGSQNYGDGSGTRAHYRALARRVFIYTQAITAVPIAMWALEP<br>LLSGGQNTPLPAWLPLDLHATPAYELLCTFQAVAVTLSVEASVCLDMFFIVLMIAVAAELHILNDNLESIRLQ<br>PVHSLPLKPVDDSAMQSYRNASSLTEKDIPQQYEFYRDSHKSMNAIHGTADAHEVMYCLLVKNIQHHQLIL<br>KCIKELETAMTYSIFVLLFLNMVTICTLIISTTVLLQSDSDPTSLYKMVSSLPIVMFQTGLFCIFGQMIIDQSERLP<br>AAAFSSGWLDGDIRLRRALLLLMRRAASPLCIIVGRMYPLSRHTYLQLLNGSYTIFNMMYQVRGRSD
BmorOR64	MEEERSRDERDLESSVRAFYWGKWMSKWIGVWPLAPNFYLFNVTFAYFTAVMLLEYVDLFFCLPNFEKVLD<br>NLTENLSFTIIYVRTLMLRVHNYKLGTAIRECLKDSSVSAFRNSKEIDIFVQYTKEGKFFAKFVIAFAAMTETS<br>WYLRPITSHTAIRVIADNETLNNVTLKFSLPFHFYVFYEINSIKTYALTYLSHGPFVPINGFGTASANCFLIALSF<br>HISGRLAVLAERIKTLKDNPDSYKRELKLIIDEHIRLLRMGEDVKISYGVNLLVYLLNGTILLCIIGYQILLTLTV<br>GPRTNIMPFIVYIMTMYMVISIFCILSENLIAESNKVCEAFWACGWYDMPPDCISDVYYCIARSQKPLALTAG<br>KFLTFGYGTITDVTRTAVGYLSVLRNFLLLEE
BmorOR65	MEESWLVRYYGGGLGQAEYERVRDFAVSEFTPLVLFLGIFPPTDKMALMSIVVSLSIAVYAFYIVLFTITCSFAT<br>DDFVLWSELIHHTSLMYLGIFIRSVLILEAKEMIKLARDYLDGIYHYEEGYVDPIFQQLQDKSRKLQRKLFMLP<br>LFIVLVTGIALGLKPLLDDVNEVEPHPKLLENGITYRSLIPIFYPFNNENTYQVLLMNGALLYFAFLVVVTVIA<br>ADLLFIRVSCRISLEIAILVESLNLIDKRAKRLYARKYGLNKKNESWPLYQDCIEECIKENVKHHQKIIIFYEQFS<br>AVAAPAIGGGFFTCTIVLGLGMIVVNMDNVNISDIIAFVGTVFAEMMNAFMISWMSEKIGEQNYELYNAVY<br>NLKWFKWRQSNKKLVITFLDGTRQPLFLNAFGMATINMEAFGSVVNTAYSFLNLVNASETLEEKK
BmorOR7	MEFESMMGPGLPLMRLTGLWQMGRQGGGVSRGLRLATIVLSVLLVVAGSTLHLVFDTPDQFEDITLCGFNI<br>DIVSLDLLKGVLFVVQGAPLRELVQLLCDARAGFTFADINHAIRGRYEAVADRMRILLQATVVLPLVGWLSA<br>PLMSRLAAGAGGSRAPRQLPVPAWLPVDIHATPTYELLYALQAFGCTAAGAFSICVDAFFIRLMLLISAEIEV<br>LCENISAIGVPHPAQGSGGCICRCQPNAADLACTCKGCVKAFTSSPEEASDEMYQLLVKAVRHHQTIIRMV<br>ALLQQTMDALVFIVLFANMANLCCSLFATAILLQRGGSLTKTLKGLSAVPVVLYQTSLYCLFGHIVTDQSEKL<br>YNAAISCGWVNCDARFKRSLLIFMVEAMKPLEITVGKFCKLSRQMLLQVFHSSYALMNLLYYYHYNTE
BmorOR8	MEFEVKTFMTRDYLKVVKFLASDIFLAKPMKILLLLIFIVQASVQAMTGYFMATAFNAKFFNNYAPIFFGTFF<br>PLLAISILLLKNKIFHNLKNELKIWSLDNAGEKIHSGITTEIKVVTYFVIVNSVFVLLANSTLAYPLSQDVNVFF<br>GCYLIHKYILTYGRTFEFFYKATYLVIGHTNTGHVYQLLYYTQHINYQLQLYIEFIKFLDEGKTISKNEDDLFN<br>NPTYQTLINQRLTFLIKRGQEIVKFHIKKTNEIRTLIPAFSVCTCTMGIGVVFFIISDNFIREYYFRMGMVSLVTV<br>STFAAGIWSGQSMETNLNEITTALNEVKWYNFNKSNRKLYLIFLTTSMRERKIKITENYSLNYQLGLTIVRGIY<br>SVISVIINMK
BmorOR9	MEFGNYKLMTDDYLKTIKFMSSDIFQPIPVKILLGFIFALHSAVNLVTAYYMLTTFDAKLFINYSSVFFGDFYPL<br>LATFALISKNNTVRNLKDELEIWTIDSAGEKLRSEIKLKIKFLNIFVVCNSLLVLVTGLTFIQPLPKDSDIFFAYRL<br>IHEHFPKHGQALEFLYRTTYVLISYIVAVQPFQIFYYCQHINFQLQISIETLKKISDWKTLSEDGENLIDNVKYQ<br>TEIKRRLKFCIQRSQNFICLHTEKIKEVSTFIAGFAVCACLLGIGVIFYLISGNFTPEYYVRMGFTSVVGIIIFAATI<br>WAGQSTESAIDEMVTSLNEVEWYNFDQSNKKLYLIFLINSMRERTIKFTENYSFNYQLGLAIVRGIYSVISIVL
DmelOR10a	MEGIVRNSFTLNLTIMKLMGIYPLENHSRLYKVFGYALYIFSIIPGSVLGFLQLFFKGDITGVGYKDLSSVVVIFL<br>SPKLCSMVFAADNVKKCIDYLDEGYFTIKNQNQEQIVTECVQICRRNSVIFLGGCTVSFITWSGTLLYRDDIK<br>QLPLIAWLPFNSQDYSLLHYVLYCSHGFGVAYVAFAAGTVDPLIPGLICHASGQVQILKDNLQHLDDYIDRS<br>DLVYEKIKECIDHYGAIINFVFKYERSFSVVILCQLLESAIVIGICCLQISKLEAYDINLIIMGNYLVFLLIQVYFYC<br>YYGTALVEENNSLINAIYMNRWYEYKKESQKALIILMECSKKPLLITAGRVVDLSLETFTLILKRSYSLLAVLK<br>NY

DmelOR13a	MEGRLSARHRESVLKWNVWVLSSGGLWPAGPPRLFAAFTSFVFIVKWTHVLMAVRTLYLSWGDLNEITLTL<br>LSMITMLGGSVKMTLFLKNKSAYYQLVQRLDEVVRYQEQYYLGNETMVSTFQKARKKALRLTFITLGYLNV<br>LGPLWFVMPLLENSSEKHLPFIPMHGLNVTSLPLYELAYVTQCTATFFWHLVSVGLDMFYASVMIYVTAQLTI<br>LNLRFMNLGLETKDFIGRPSLGSITASFDNRMFADAHDKMYKELCDLVRSHQKIIEFTNYLEQVMNSTVLVQ<br>FLSSVLVACVTLFQATINSQGNTVVKCWLYLPMPAFQIFVYCWCAHDLMDQGLEVSTSAFLSAWVEGSRGL<br>RRGLLLVMVRSGRPLEH
DmelOR19a	MEHIVDIFLRKMGCSDDRSYDTMCVIYFTYCELAVTLFFTISTYLSIVYSTEDLSMRLYGLLCFLIEIHIFGFIAAR<br>FYHQSQFRDMYHRSQEVGIPENYRQKIAMVIKHYFIMSNVFVAVSVLYTISLDWVQMGDPFTFPFVDVLPIK<br>TINLSVYVCKYIVYALPVYFAHLEICFLNVTFMYSTGVVKRHFQILDEQVEEAILNSEEQQLKIAIKHHQEVLK<br>FFEDMKTIYEKPILMTIEFCGLYVGLTSCFVIQVIQGFIHQIILGLCIVSSVACIMTIIIYCIYASNMYDLHNGILNS<br>LFEHRSCYSRNKSFKHLILMMMTRASIPLEFKVGSIFTVNLNLLVKILKFAYTVFNVLLTSINRQFK
DmelOR19b	MEHPYLAAVTPCRVMKESGGGQRAGADDTASLTWRQTAGSVLKVNVRGLALFGSWPLPESWLYHAFFAV<br>VFASNLGNMAEAAVGLWLGRGGLEEITLVLPNTLTTAAGVCKMVFFYRDRGRYYRLVRRTDLLAGSQLAAS<br>GRHGADAVRQADRQSLQLTYTVFAFISLQIIVWFPMPLYAYRDQRKLPFVQLPWNEDKDIPVYELSYALQCF<br>SSFTIIFITLGMDCLFAVIMIHVAAQFEILIVRIRNLRLDLQTTGVTQSKASLGQLSRNDVSSINVQTDYKEVSH<br>HQQQINEAHDKLYSELCHCVESHQEIIRFVRHLETMMSPIAMTQFVFSVLVACVALYQATYSDDLSAVFRCA<br>GFLPVPGAQVYLYCWAAHHVMEQSEAVSAAAYACPWIEA
DmelOR1a	MEILTTNFFVLSLVGLWKPRGWSGIKAILYHFYSSIVIFANVSFLISGIMDLEFTNIDIAAFIDNVSLLLSLVTIRQ<br>KTACAIGNRGDIKEIIDSLGRSPFKPQDEEEENIVKRFDDLTGYILKYYPVLFTVAITWYSIGHMFIMDPPYVLP<br>YRGWFPYNYTTTGVYWLTAVYQLYAICSAASINLAFDPLLPCIICHMCAQVHILKYRFGVMLKKLEVISDNE<br>PRYVIASAERKLMGEWVDYHISILNLIKYVNSTYSKVIFVQYTASSLILCTVAYVLSHMDALSMNFAGNFFYFI<br>AMNFQIFFQCFCANQVTLEFLDITTALYDENWFNLSNNVRRSMTIILCEPFRPVLFTSSFFIVLSLESFTKVIKC<br>AYTVYNVLQ
DmelOR22a	MEKALKLVNILGLDPRKNDTFSKFRSIFCFTILISASFSSHLEFFLNFKGLETCERAAESIIPQYQTMCKMATFLL<br>YKTEMLDLIKKSERFWKLDRFGDLQAKNLHSTYPIFQIFFYVYVVILFLTCAMFALVNWIFDTGKPISLCYGES<br>EGLETPWVEFYIVLQSVEVTIIFLGITGYDMVFLYYAGSVCIQFQMLKMAFAERKMNERQFLKAVKHHEFLL<br>QYVEQLGDIYSMWFLLQYFSSLFGICFGLFLISKEGLPTEPERLSKYFPYIFSFTMQSFTFCMTGTMLSDWSSEIS<br>DEIFHSDWSDDQVYKNKTARLIVMNRAQRPAKISIGKFLDLNLRSFILLMRSVFSFLAFVNNILNRIN
DmelOR22b	MEKFDWRCPIRINLLLLRSVGLWPRGYGVYKRNYYLFYSIFTTITIVGGHNLSQVINIFYVYSDLEALTGTIFVA<br>TTNILALVKRYVFVRNLPLIKEILQTLNTYQFHPKTRQQLKIIQAPLRRWKLAYLCFSIIVYFNVAMWTLEPLL<br>DKMIKNRRLPFEAWYPFNSKQSPNYEIAYCYQFICIWNITIANLNLDTLIFAFMMFISAQCEILCDDLRSLDVG<br>FGPKLIQCIKHHKEILRLAKVTNNIFNFIILGQIATSTAALALTMFQLSLISSINTTALTHFAYMMGMLSEILLYC<br>WFGNEIEVKSYLIPNAAYESQWMHQDRSVAKNLLILGCRCRKPIKITAINLFTLSLPTFIAILRSAWSYFALLSTI<br>NGK
DmelOR22c	MEKKTINDHTSIKLMRLCMNGIGMWSIEKRRDEIISNIVICYTIATLTVGLIVETTDIYYCLGDLREMSYVAPCL<br>LNVIVELFLMGTFVINRSEVIAFSDYTTREFWSIPYIESERKLLDDCNRKSVKIIIAFIVVIQLVVWQYITIPIIESY<br>GKNASERTLPYNLWFTFIPFKETPYYEICFFLQSAATLTTGVCATAFATFLFTINLYATGQFKILQQRLESSCQVY<br>NIEKIKSVEQINLIAEESYANLRKCVELHNVLLKYITRLENLYCQIMLVETLACVFLICTTGFQIVLGVDSSILRT<br>SRSALYFCCLVTQLLLYSWSCHEIIIESLEVAEAAYRAYWYSLSWSKYGKSFRQALLIIITRSRRPCVLTVGKFVP<br>MSLETFTAVFNSALSYFTILRQMTEEMENS
DmelOR23a	MEKLDDPFITLRKMVFIEAKNCKIARFCDVLLIVLYSLAQCLHLYYMCQNFNLNLLIRYGPILISCLLVIVTAVI<br>SVGLDKEIFEVYTVCWKISWPLNFLRKDAQTKLRRKCQIINRGILCSALLFLTTVISTFPCFGSVRDFFICVEVY<br>EKYFGEWSFIPYYFYFAAAPFLYYHFFRVCYVFAYAFLHAQLQYFLIEEYLLETYQTNDLKGWKYLQDTRYQQ<br>EIGKSLLLCITHHIALKKYVKISQNLVLIGMPFFLVLGVLLLINSFGFITNFGDTMSNILKIRILIFVACGVSITIV

	MCWIGQQLIDVTSEIFVTLGGAPWYFWNRDNNNILLMFLTNCTKNESFILAGICVNYQLFFSIVRLTVSYTLVLYNLRESGFI
DmelOR24a	MEKLMKYASFFYTAVGIRPYTNGEESKMNKLIFHIVFWSNVINLSFVGLFESIYVYSAFMDNKFLEAVTALSYIGFVTVGMSKMFFIRWKKTAITELINELKEIYPNGLIREERYNLPMYLGTCSRISLIYSLLYSVLIWTFNLFCVMEYWVYDKWLNIRVVGKQLPYLMYIPWKWQDNWSYYPLLFSQNFAGYTSAAGQISTDVLLCAVATQLVMHFDFLSNSMERHELSGDWKKDSRFLVDIVRYHERILRLSDAVNDIFGIPLLLNFMVSSFVICFVGFQMTVGVPPDIVVKLFLFLVSSMSQVYLICHYGQLVADASYGFSVATYNQKWYKADVRYKRALVIIIARSQKVTFLKATIFLDITRSTMTDLLQISYKFFALLRTMYTQ
DmelOR2a	MEKLRSYEDFIFMANMMFKTLGYDLFHTPKPWWRYLLVRGYFVLCTISNFYEASMVTTRIIEWESLAGSPSKIMRQGLHFFYMLSSQLKFITFMINRKRLLQLSHRLKELYPHKEQNQRKYEVNKYYLSCSTRNVLYVYYFVMVVMALEPLVQSCIMYLIGFGKADFTYKRIFPTRLTFDSEKPLGYVLAYVIDFTYSQFIVNVSLGTDLWMMCVSSQISMHLGYLANMLASIRPSPETEQQDCDFLASIIKRHQLMIRLQKDVNYVFGLLLASNLFTTSCLLCCMAYYTVVEGFNWEGISYMMLFASVAAQFYVVSSHGQMLIDLSTNLAKAAFESKWYEGSLRYKKEILILMAQAQRPLEISARGVIIISLDTFKILMTITYRFFAVIRQTVEK
DmelOR30a	MEKMFPQIRTEDMKKFPYYYLLKICIVFGYSKIVKLLNVVCIIITSSTIVLQVYYLKQNFSKELILKYGCGISLTIYTIASMLVEFLIEQKTKKLLNEAGTILWPVNFCGVKVEKLILKRVTVMNIIYYFMSAWFALMGIIMLPIWGDHSEWLLCDVISNEYFETRWKILYFACSCFSFPVIAFSSIRLPVILLCTILQTHMQIILINQKLNQISEQMGNLNNIKLVDDKCYQKRIFEDLRLCVSHHGKIKKWLNKVLKLVQSIMPLYIILGCLNFISLLFFASDGLQNASNILKARLCVVLIVCCLVLSMFAEAGQALSDETSGVFDTLLTCPWYLWDKNNKKVLSIFLSNSFQPDSISVAGITLNYDFAVALLKTSSSYALVLYNMKN
DmelOR33a	MEKQEDFKLNTHSAVYYHWRVWELTGLMRPPGVSSLLYVVYSITVNLVVTVLFPLSLLARLLFTTNMAGLCENLTITITDIVANLKFANVYMVRKQLHEIRSLLRLMDARARLVGDPEEISALRKEVNIAQGTFRTFASIFVFGTTLSCVRVVVRPDRELLYPAWFGVDWMHSTRNYVLINIYQLFGLIVQAIQNCASDSYPPAFLCLLTGHMRALELRVRRIGCRTEKSNKGQTYEAWREEVYQELIECIRDLARVHRLREIIQRVLSVPCMAQFVCSAAVQCTVAMHFLYVADDHDHTAMIISIVFFSAVTLEVFVICYFGDRMRTQSEALCDAFYDCNWIEQLPKFKRELLFTLARTQRPSLIYAGNYIALSLETFEQVMRFTYSVFTLLLRAK
DmelOR33b	MEKYRGKGMVESLSWAESGRSVLKLNIRHLWLMGVWPLGRSPVFKVYTGFTFAIGIWSVVECLLAVYYIWGQLAETTVVLMFTFTCSGCIIKMFFFVCNERSYSLMVREVASVMAAQSEACRDPALAAILRDSRSRAFRLSLGMLLFMFAQNFIWFPIPVVAHAGERRLPFSQHGWDNNSHFYGLSYTLQCLSGLYMSQISFGLDCLFASVMILVAAQLEILSGRILKLNQEVILEQRNESVLWKNKMTMDENRDTFYETLCFCIDSHQKILRFVTLLQDTMSPVAMTQFANSVVIACLALFQATYGEDMSAAFKCACYLPIPGGQLYLYCWAAHSVTENGESVSVAAYSCRWVEGSARARHALRTLMARAQRPLALTAGRLYPINRAAFLSMVNASYSYYALLGQMNNR
DmelOR33c	MEKYRGKGTVESLSWAESGRSVLKLNIRHLWLMGVWPLGHSPVFKVYTGLTFLMGIWSVVECLLAVYYTWGQLEETTMVLIFTSTCSCGIVKLFFFVRNESSYSLMVREVASVMAAQSEACRDPALAAILRDSRSRAFRLSLGMLLFMFAQNFIWFPIPVVAHAGERRLPFSQHGWDNNSHFYGLSYTLQCLSGLYMSQISFGLDCLFASVMILVAAQLEILSGRILKLNQEVILEQRNESVLWKNKMTMDENRDTFYETLCFCIDSHQKILRFVTLLQDTMSPVAMTQFANSVVIACLALFQATYGEDMSAAFKCACYLPIPGGQLYLYCWAAHSVTENGESVSVAAYSCRWVEGSARARHALRTLMARAQRPLALTAGRLYPINRAAFLSMVNASYSYYALLGQMNNR
DmelOR35a	MELAHLLLRLLHWSGALRHPSKPCGCYLLYSAAVVTIIGLFVVTQVAAVVDRQAGSDLDQATLALCVASTMCVGMCKILNILRSEHLFLKLADEVSRQPDGLSAWEAGAWLWSRGRVRRLSAVYLSLAASMAVTWPAAPLAVGGGALPFVARFPFDVAAPAGYAAAFAFQVLVVAVVGVVVPCTDLFTVSCVEHLNSLLHILVHRVERLNALETGARPQHATADDKAAAVADPLHRDLADCVALHQRIISEAECLNQAIGGVLLLQVVASSAGICFLLFQVAKKTTFHLVETGKLLGYLTFMLSQLLFYCWFGDDMLSKSESVSLASYRCRWPDAPTRFQRSLLMVSMRAHRPLTLRAGKFFVFSRQAFVQVMNVSYSYFTVLRSLSEA

DmelOR42a	MELDNIKLRHLEPYYNIKVSLTLTKYIGTWPPVLEPYRSIYLLYTCVSFIFILGIYLTVQTVNLFVIWGNIELMIATGFLLMTNSIHAYKVFVILGNQKRIQVLLDKLSTTNYYHNDDKYERVFTYYAWQGLYHHIAYQSFGTVAVLCWGLTPLADAVAGNTRRLPMEAWYPYNTKKNPAFEITSGHQAVAVLIACVHNIGMDTLVTGLINAACCQLEIIKQNLKNVDLDFEYQIDKCDYEDFMNKQINKIIKHSNEIYK
DmelOR42b	MELGCSRHLKLPCSLHPIGISKHGNTLSELLIYFPAIPKITYAILAVLLTVYYYIYLCSITWFVFVRCPQTGDLAAASIVFSLGVSSEIGAIKLFIIAKLRDITGEYLQCEADMAPGRLRARVGRSLRTVRRRAFVYWLVLVVNAFAYDLMPAFLPGRHLSEDVFVIYGFEPMFESPNFEIASTLMGVSVVFICYTAGSISAFLIVIVGYSEATMLALSDEISCVWDDACASECQQPNDFIRARLGKIVAIHTKQIRLIREVEVVFRGALAGGFACVAFGLIAALLGGLENTFLQLPFCVIQISVDCFVGQRLRDANVAFETAVYNCKWEYFDKSNMKTVLLILQNSQKTMGLTAGGVAALDFTSLMTIFKSVYSGVHHSQTDD
DmelOR43a	MELKSMDPVEMPIFGSTLKLMKFWSYLFVHNWRRYVAMTPYIIINCTQYVDIYLSTESLDFIIRNVYLAVLFTNTVVRGVLLCVQRFSYERFINILKSFYIELLQSDDPIINILVKETTRLSVLISRINLLMGCCTCIGFVTYPIFGSERVLPYGMYLPTIDEYKYASPYYEIFFVIQAIMAPMGCCMYIPYTNMVVTFTLFAILMCRVLQHKLRSLEKLKNEQVRGEIIWCIKYQLKLSGFVDSMNALNTHLHLVEFLCFGAMLCVLLFSLIIAQTIAQTVIVIAYMVMIFANSVVLYYVANELYFQSFDIAIAAYESNWMDFDVDTQKTLKFLIMRSQKPLAILVGGTYPMNLKMLQSLLNAIYSFFTLLRRVYG
DmelOR43b	MEMYQGKGMLKTLSWTESGRSVLKLNIRHLWLLGVWPLGHSQVFKVYTSFTFAMGVWSVVECLLAVYFTWGHLEETTLVLIFTSTCSCAIIKLVFFLRDERSYSLMVREVASVMAAQSEACRDPALAAILRDSRSRTFRLSLGMLLFMFSQCFIWFPIPIVANAGERRLPFSQHGWDNNCHFYELSYTLQCLSGLYMSQISFGLDCLFASIMILVAAQLKILSGRVLKLNQEVIPPERNDSVLLRNQLAGDKYCDKFYEGLCFCIDSHQRILSGGVQTEECLRVAILDVWRVALFCWNWFVTLLQDTMSPVAMAQFASSVVIACMALFQATYTLHSALYHIHEQLLSVDSKNVLSAIFLWSPSPWHSPPDVQLGTIPRSFANGEGESVSVAAYSCSWVEGSAAGRLYPINRAAFLSLLNASYSYYALLGQMNKRSMKDLASHN
DmelOR45a	MENDQKELDEGVKAFYWAERMSRCIGLWPVTPNYYLFNICLLYFSVLMVLELIDLYNSVYDIDKLIDNFTENLASTHMYARILMLRVHNYRIGEMITQAMKDYRISAFKNSYEIKVFMEFVNKGKFLIKGLFIFIMSTEISWFLKPLTTPSSSDNSIVNANKTFPQFILPYNVYIFYEVNSIKRYVLTYLSFMPMVYVSGIGHSAVDCILVLLVFYISGKLSVLTMRIDALKNNQYDCRKELKEIIAEHSRLLKMGDEVKDVYSTGLLVYLVNGNLLICIIGYQILINYMTGPNSDLLQYFVYIGATYFMIANFCIISEHLTAESNKVCEAYWNCEWYNMPQDCVKDIIYCIVRSQRPLALQAGKFSTFSIVTLTDVTKTALSYLSVLRNFLIAE
DmelOR45b	MENIPIDIHRKFLNINITILRYSGVWPLLPTAKIGWKVFNFIYRIFNLTVFIFYLITLGADAVTNYKDLTIFGSDGCYFFGTCMCVFKACKFWASYHKIIKLIVDVYDPIDVLVRSADPGILMNIKKSYYQESIAFWGFSTLCSFFHFSVIFLIPREKGILPIRAIYPFDTKISPNYELAIIYQAYCLAYALCVTIALDITTIGFIRWSTLQIAALTSNYKNSNPNVTKRASLVTSSSDARKIIEKLNKIKITDDDVEIETFLPLDYHETKYFINDLFLSRFTTCIKNHQRLIKIIRDLNAVLSPLMLVQFATSTCIICLNGYQMILAETMTYNQWMSGWECAYGKVKSNELRNLVTIAMMPAIKPFAFNAVGLFALSMPTLLAVVKSSYFMLILLTTVTED
DmelOR46a	MENPERVDYDSNGVTGNPAEKAEGGEMDNPPKNDTEGGTILDVELFKIIGVYQLLRPDEFGLNARLCRTTAIVVVCLTLGLQSMQVCRLYLARHDLQMFANVGVMIINGLMCLLKGYMVAANADRMSATLNAARYAFTGCGNRDQSKLRLCRARLSTILRTFVRLSFGTLIVWVVMPWFMASEYDDTPSIWATVYVIESIILTVNVFCWTSFDCYLVTMCFVLEALFCTMSTGYETLGRHRAAAKSSAGQQLAIAGTISDVSISDVNYDDLTSHILDNQNIIEQYDEFFDVVRPMVLVQIANGMYSIITLIFLTLLTYLSGYSIVSAPFLKFVCGLASLTIELYIYCYGFNHIEDGKSTVNFGLYSSNWTEMDLKFKNTLLMAMIMNSAHKRVMKVSPNSIVNLEMFTGVMNMSYSIVSVLLN
DmelOR47a	MENPLKLLHIIGLDPRQSDKYSTIKKVISFLIVLAVLLSALIEFFLHHNESQVYDTAPQSTVPNLQALLKMFALIIYKKELIDLFTKGNHFWKLDKFGDCHKQKLTKLHKYVDLFFYVYAVIITGAFLQLALLILIFEPGKPIFLCYGGLYGLESPQFEFYAVLDFLAIGVIAISVTAYDSIFFYFALYIYTEFKMIKIAFKRENCAQFIEAVKHHDFLLQYISKV

	NEVFSVIFLTQFFSGLLGICFNLFMISTQGTRDMKSFSTYFVGLVGYTAQSFTFCLIGELISELSEDISNEIFYTDWLDDEVYRNTTARLIVMNRAQESPKLTIGKFADMNLRTFIIILRNAYSFLAFINEVLD
DmelOR47b	MENTSIESRQSVLSMAIAGLRLCGIWSLDSSSPIFLKLLHNISNIFGIITLIIFVGTLTIDLLLNSNDLLIATDDGCYLAGISVIVFKVYQFHRHHKRIKNLTDATYQPIYVFWKSTDIGVKTVLRTNKFYEDLGFTFFVSLGGFLVIALIFFVPTEEGALPIRGAYPFNTTISPMHEVAFCLQIYAVTYGLMVIVLMDGMGLGIMRWLNVQCIILASNYRNCRTNQNNSFYLESRDDLSKIASIEDDNNNVTDIYDEPDSNITTFCPFDEQDHAGMSDCFIGRFKKCIKNHQRLLNTIDELNACFSSCMLMQLFASFSMICLTGFQAVLGATTKTSLIKFVLYLGAAFSQLLYWCWFGNELLYEVFILPHD
DmelOR49a	MEPVQYSYEDFARLPTTVFWIMGYDMLGVPKTRSRRILYWIYRFLCLASHGVCVGVMVFRMVEAKTIDNVSLIMRYATLVTYIINSDTKFATVLQRSAIQSLNSKLAELYPKTTLDRIYHRVNDHYWTKSFVYLVIIYIGSSIMVVIGPIITSIIAYFTHNVFTYMHCYPYFLYDPEKDPVWIYISIYALEWLHSTQMVISNIGADIWLLYFQVQINLHFRGIIRSLADHKPSVKHDQEDRKFIAKIVDKQVHLVSLQNDLNGIFGKSLLLSLLTTAAVICTVAVYTLIQGPTLEGFTYVIFIGTSVMQVYLVCYYGQQVLDLSGEVAHAVYNHDFHDASIAYKRYLLIIIIRAQQPVELNAMGYLSISLDTFKQLMSVSYRVITMLMQMIQ
DmelOR49b	MEQLPKNDPLLVLRALPEILMQHKIIKYVVLFIICYMTVTMILCSYVLATVRGLWDLFWSQYSLLAFGSSIGFSCYFVAFWKGSEFIKLRRRVFANYWPLTSLGEESFQKIKKLSIFANVFMVATILASLATSTAGLPWVGDEYDIMFPVRVYTDYFGERAVPLLVPFYLAMYCTGFVMISTGFIFVHFALHLKFQFFLLNKRLDGLRTEPLVNDFLYQNHVKEELTCCIEYHQKLLKVAKEMNDIVYYPIFIVVSCGIMFSVCLVFYMKNFKNSFVRGTTMAMTGTLTTFGFGFTGQLMENESGRLFDTSVMLPWHLWCLSNRKLYHIFLTKCQYHVSFSSSGIINLNHTLFISLYTKITSILSFLLNVSKKNHTK
DmelOR56a	MEQLPKNDPLLVLRVLPELLLLHKIVRHFVVFIVCYLTATTIFCLYVLATVRGLWDLFWSQYSLLTFGSVIGFSCYFVAFWKGFKFLELRRRVFADYWALTSLGEESFQKIKKLSKSANIFTVGTILASIATSSTCMPWVGDEYDIMFPVRVYTDYFGERAVPLLVPFYLAMYCTGFVMIATGFIFVHFALHLKFQFFLLNRRLDGLQTEPLVNDFSYQNRVKEELTCCIEYHQKLLKVAKEMNEIVYYPIFIVVSSGIICSVCLIFYMKTFENSIVRGTAMAISGGLITFGFGFTGQLMENESGRLFDTSVMLPWHLWCLSNRKLYHIFLTKSQYHVSFSSSGIINLNHTLFISLYRKVTSIFSFLMNVSNKNST
DmelOR59a	MEQQRAIIETSLTFFKVLGIHPLKTLRKIPVLCLISSHLILFALVCLRFFYEKITFGLISDTFESTFTIVHVLAKFITLVIIKPTIKKLFETTNTFWLGKTFQKEFRHEVDADLRTLGLLLRLFLFFVSIVIVTFAIRPIFDDTLAIHCYVPEFIPRAIFIVFNNAVFAVSAFAVGSFDMFVCVMVVLITVQFKILNYSILGLDLGKVVTENDEKLCQKKIKIIVDYHDFLDRYLAKVSSIVSPALLVYFTIAPIVLCFELFFMSKSSNFAEIAKSCVYILGIVIELFFFFCLPATYLMVQTQLMITTVYNSGWENCYFLSVRKSLIIMMQRMQKETLLTAGKMIKINLETCTNAFRMALSFYTSLQILDDKSRE
DmelOR59b	MESEAKSLVGPSGWALRQLGLWRPPGAAPAGPRLLAAFFVVATDALVSTSSAVQLAVDTPTNPETLRDVFFQTTCSGAWAIRTVLFMQQRDRLQRLVMTLLDTRKRYAENVPGTRSSYDRGAAIVFFAWQMLPLTAISLWALEPATVPAEPVLVGNTTVVLRREPLVLWLPIDTQRSPTYEVVFVMQVIGIATVSEVSVLLDIFFVSLMIHVTAEVEVLSGNVLNIHLSTLDGQLTQSREYGDGRLSYKGGGQLAAIDEPTDLSAAGSGYLPNKTLFYSQDMDDAQRRLYACLKTNIQHHQTIIHCVNELEEAMSNSTYLILLVNALTICLHAFGFVELFQGGGKGPAVVKRLLACPIYMGQTALFCVIGQSLIDHSERLLDSAFSCGWPSADRRFCSALLIFMRQASQPLKIRVGKIVTLSRNSFLQIMNVSYTIFNMLLNTQ
DmelOR59c	MESGAAEDVEGPMGWSESGGSVLRLNVRHLWACGVWPLPGGWLFEAYAALGLALGAWNAAESLLALSFCWGDMEETTLLLTSTFTIGCGSAKMALLLARRGRYRALARRVQALASLQTGHCLADPALDDIRRGSQRRAFRLTLSMLLFMFSQCFVWFPMPVLAHWEERRLPFAQHAWDNNTRLYALSYAVQCVIGAWTSQLSFGVDLLFVAVMILAAAQLRILTIRIASLKTESWKVKPEGGARCEDVRPENGRDVMYENLCLCIDSHQKILRFLKHLENTMSSVVMTQFCFSVLVACVALFQATYSTDFTAVLKCASFLPVPGGQVFLYCWAAHNVTEQAEAVTMAAYSCSWVEASVRFKRALRILISRAQKPLVLTAGHLYPINREAFVSLVNASYSYYALLGQMNRR

DmelOR63a	MESSARHDDSVGAKRGAVRLTLWKGPEQTTSILRLNVRCLLLGGIWPKSRGALYLAYSAFIQLCSISYIVMCM<br>LSIFSPDGDMNDITLTLLHTFEVVCGVVKAAIFYLKRHQYYQIVRDLDQLVSSQRQYLTASKDDHLLAMLDA<br>AHKKANFLTLVLTGYIYGLVFVWLPFPLILSPSDRLLPFVPLPGRYYKDSLLRYITVYAIQSFVPLALIMVVDGL<br>DCLFVASVVHAEALLKVLSERLASLGHSHYTDFRLQRSSGREQEPRKGNVGDNSNITAQLRSCIIHHQQIIEF<br>LQNLEKAMNIMVLIQLSFAMFNLCMALYQQTKIPDFTSALKYVMYLPFPTMKIFFYCWAAHNVKEQGEEVS<br>WAAYSCAWPDADQEFQKSLAIIMCRAQRPLLMTAGRIYPINKDAFVSLLKGSYSYYTLLRQFESK
DmelOR65a	MESTNRLSAIQTLLVIQRWIGLLKWENEGEDGVLTWLKRIYPFVLHLPLTFTYIALMWYEAITSSDFEEAGQV<br>LYMSITELALVTKLLNIWYRRHEAASLIHELQHDPAFNLRNSEEIKFWQQNQRNFKRIFYWYIWGSLFVAVM<br>GYISVFFQEDYELPFGYYVPFEWRTRERYFYAWGYNVVAMTLCCLSNILLDTLGCYFMFHIASLFRLLGMRLE<br>ALKNAAEEKARPELRRIFQLHTKVRRLTRECEVLVSPYVLSQVVFSAFIICFSAYRLVHMGFKQRPGLFVTTVQ<br>FVAVMIVQIFLPCYYGNELTFHANALTNSVFGTNWLEYSVGTRKLLNCYMEFLKRPVKVRAGVFFEIGLPIFV<br>KTINNAYSFFALLLKISK
DmelOR65b	MESVAMGEGEKRKTNRSEGCVDYRKFVFCRLMRTDDGLVKRGITGSLLIIMVTTVAMETCSFVSIFMSKQLQ<br>SCLDCVRSFLLGNLVTMVLFNEFANRKRLARLHDFLNSSMSSPRTDLPEAQEILNKAREEASSSLRMYIIIFSG<br>NIAPMILAQPLAEWISGHSWKKLPIPWAFPPSDSDVKFGLVFTFQCIGVCIANCLGMVFMSFSSITIQMTAMF<br>DVLLLSLRRIENRATIRTQREEMDYKTSLLYCLREDVIFYQQLVREMTSATPHLRNTFLAFSATVPMIMACEAY<br>PIISGNFTIGDLIRSFVFLAIQFTCWAQTCTKLETMTDQHEAVFREIYQTPWSDSGPVYKRLVYTTLLFSTQPKH<br>IKARLSNEISATTSTFYSFVVSSFNWLSIIRKMN
DmelOR65c	METAKDNTARTFMELMRVPVQFYRTIGEDIYAHRSTNPLKSLLFKIYLYAGFINFNLLVIGELVFFYNSIQDFET<br>IRLAIAVAPCIGFSLVADFKQAAMIRGKKTLIMLLDDLENMHPKTLAKQMEYKLPDFEKTMKRVINIFTFLCL<br>AYTTTFSFYPAIKASVKFNFLGYDTFDRNFGFLIWFPFDATRNNLIYWIMYWDIAHGAYLAGIAFLCADLLLV<br>VVITQICMHFNYISMRLEDHPCNSNEDKENIEFLIGIIRYHDKCLKLCEHVNDLYSFSLLLNFLMASMQICFIA<br>FQVTESTVEVIIIYCIFLMTSMVQVFMVCYYGDTLIAASLKVGDAAYNQKWFQCSKSYCTMLKLLIMRSQKP<br>ASIRPPTFPPISLVTYMKVISMSYQFFALLRTTYSNN
DmelOR67a	METKEDEYMKPLVQLLKFGGFWFDFSGHKHAMALKWCNIARNVIAFSVWAYQMAYFMGGVSYLLTEAGV<br>FVPICFDEGAVAVIVLCNQSTIRETIRIYRKRFEVFGSTPWAKNIIDSEMNKFNRIFQLPKLMLSVFFLFYSVVPL<br>VYDAVLAYSGKSPYVVPLPLNFLLETPMQRTPAFYMTIYLSYLYFMIIVPRFIAFEALMMYIVAFVVIDVKILVQ<br>KMKNLSEKDDGSLFLQEEWNLKDVIDHHSTVVRVVEEHFWLVGLAMMVQNLTFSISSCLVIYLTKTSFNNG<br>DIVLALFCGNFVVLLMILNLMFNGAGVLIENQGEMVLTAIYDTEWYKQPPKVRKEINAMLRQGLHVLKISY<br>WSNTVNFETAMVVLNRAYSFFTLINTGE
DmelOR67b	METTATKNDNTNQNKNELQLTKTNKYENVDYKSDADYAVVVARTLLTPLGIYPLHGSDTSLSKFLIAIQIIIV<br>FGLMLFLLVPHFIWTFFDAEDLKKLMKIIAAQIFNSLALIKFWTMIIHKKELRNCLIQLENNWKNVLCEEDR<br>VIMIKNAKIGRFFTIAYLSLSYGGALPYHILLPLTAERIVKEDNSTQIPLPYPTDYVFFVPEDSPGYEMLFVTHIII<br>STMILSTNCGIYSLIATYIMHACCLFEVVCRHLDEFSKNNTNNFKTELTWIVENHNRAIQFAETLESSLNIVFL<br>CEMVGCTVIICFLEYGVIVDWEDGKLLGLVTYVILMTSIFVNCFIISFAGERLKEQSIKIGESAYFAEWYLLPKGL<br>VYDFMLIMIRSSKPASLSTGKVSDLSLAGFAGLVKTSAAYLNFIRAVV
DmelOR67c	MEVHSRDQRDLDKGARLFTWARFISKGIGVWPLEPNYYLFNICIFYFTYIMITEYIDLYYCLPNLKKVINNLTE<br>SLAFTQMYVRAVMIRVHIKKLHHLMSEALKDYHVSAYKNSDEVYEFMSYVKRGRFFVKSVTIFILSTTTSWFL<br>RPITSSTPSTSMSAPDNETAAKFTYILPYKFHVFYEINNYRTYVLTYISHGPFPYVSVLGAITSAVFLIILSFHVSG<br>RLAILARRINALNCKNEGFRTDLTDIIAEHTRLLEMGEEIKISYAVALLVYLVVGTTQLCIIGYQILVLITMGKQ<br>HSLMPFFVFILTTYLLISIYCILSEHLLAESKKVSEAFYSCEWYDMPQDCIKDISFCILRSQKTLGLTAGAFLTFS<br>NSTLTDVTKTSMGYLSILRNFLLNEQ
DmelOR67d	MEVHSRDQRDLDKGVRVFTWARLMSKCIGVWPLEPNYHLFNICFFYFTYIMITEYINLYYCLPNFKKVLGNL<br>VENLAFTHIYVRTLMLRIHIDKLRDIISESLKDYHASAYKNSDEVNEFLIYVRKGKFFVKAAGIFVISTATSWYI

	RPITSPSLPNNETAAFTYILPYKFHIFYKISNYRTYVLTYLSHAPFAIISGVGAVTSAWLLIMLSFHVTGRLAILA<br>KRINSLKDKNGGYRSHLDEIISEHSRLLEMGEGIKSSYAIALLIYFVNGTILLCIIGYQILVTITLGVKHNLMPYF<br>VFILTVYLVISIFCILSENLLAQSNKVSEAFWSCEWYKMPQDCVKDITFCILRSQKTLGLTAGAFLTFSNSTLTD<br>VTKTAMGYLSILRNFLIVE
DmelOR69a	MEVLTFNFFLLSIMGVWKPRGWRGIKAILYNINRSIVVIVNHIFLLSGILDLEFKNVDLDAFVDNLALILAMV<br>VVRQKIVCVIQNRTGVKHILDSLAKGPFKLRTHQEKLIFSRFDDFARNIFTYYPLVFMSSLLTYSSGHMAVMD<br>PPYVLPYKGWFPYNYTRTTKIYWTTAIYQLYAVFTTATINLILDLLLPCIMCYMCGHIHILKHRFKEMIEKLLV<br>MSENNVPQEKIISTERKLLGEWIEYHIDILRLVKFTNELFSSVVFVQYTVSALLLCTIAYLMSHTDTMTMSFAG<br>NLAFFTAMFIQILLPCYCADKLSYEFLDISTGIYDTNWHYLSNNIRKSVVIILRKSYRPVIITSSFFIVLSLESFTKV<br>VKLAYTIYNVLE
DmelOR71a	MEVTGKKVTSGDPFITLKKLYIDLGYHKVTKLVNVFFIVFYGFVYLLQIYYLIVHFNFEIIAKYSTILLLSTYLFN<br>VMIFSIIYEKCILDAYKTFSQIAWPCDNASKPLQIIILQRSKTIKYLNYFFLGFIFFMACINWPWLGDQNDFLLCI<br>QVFKKYFGSWSPLFLLFYYLGFPIIGYSAARIFFIILYGVLHLELQIRLITELFCKISRNATLEDVRNAKYQRDVY<br>WTLREGIRHDTALKKVLFDLNKEVKHGIPVFLIVTLLCSVSIFFFAITYLESMGFGIQIRTIAFVGAVIFVLFSYSL<br>LGQHLINQTSLLFDQLYECPWYTWNVKNRAIYLNFMLNTVRPIKITYAGICIDSRFFLSITRIILSNAFMLYQLR<br>NS
DmelOR74a	MEYLKYCQSYIIGSGLASNSPVFRQFLARYLFFPLSLILVSLSIYIIKDANNDIYFLTEVMESLASYTQLLIRKYMI<br>FTQSKLMVEIINDCENLWSLELFGPELGKKFKQQMKNCWTFVNVLVTSGFSTVLLICITTLTDKEKSLPFVCW<br>IPGFPHATELIFLTQFVLLMNGLYYIKLTDAFYLLVCMDIQIQFKMMGKMLKTIHFGLLSEKESWEKLVELAK<br>HHNKILHKKLNKVYSKYYVVQYVMSVTAMTAQAYTLKYIEVNIQLALKSIMYTCSLLLQGALYFFPASNIEIE<br>AENFSTEIYFLNWQDHGDVKIRKHILFMLLKSQENLEMMGEGMMHINRNEYLMMFRLSFTIATLLGGLNQ<br>L
DmelOR7a	MEYLKYCQTYIKGSGLAPDSPPIRKFLAKFFILPCFLIIALSIYKLRDSNSDIFLVIDVLECVASYTQLTIRKYIIYN<br>HGKLMEEIISDCENLWSFDMFGPELGKNFDQKMRNTWTVVKSLITCGFVTFILMCVSATTSKENPLPFTCW<br>VPNFAFATELLFLLQFLLLMELLYDVMAMDGFFLLVCMDIQIQFKMMKKMLHSIQFGVTSEEESWDKLVELV<br>KQHNKMLKLHQKLNRVFSRYFLVQYAMTVGSMTVQAYTLKYSEANIETTIKSIVYTISLMLQGASFFFSASNI<br>EIEAGNFSKEIYFLNWEDQQDVKIKKHILFMLMKSQENLELTGEGMVHVNRRDYLKMFRLAFTIATLLDGL<br>SHF
DmelOR82a	MEYLKYCQTYIKGTGLASNSSLFRNILAKYFFLPPCFLIIAFSIHELWDTNNNDVSLVTEVLECVASYTQLIIRK<br>YIVFTQSDLMVEIINDCGKLWPFDMFGSELGKKFKQQMKTCWTLVKFLVVCGFATFFLMCISARAAERDNSL<br>PFLCWVPDFPYATELLFFLQFMLLLELLYYVLATDAFYILICMDIQIQFEMMGKMLKSIKFGEISEKECWDKLV<br>ELAKQHDRMLHQKLNQVYSKYYVVQYFMTVGSMTVQAYNLKYRMVNIQTALKSIVYTFSLMFQCGYYFFP<br>ASNIEIEAENFSTEIYFLNWQNIGNIKIRKHILFMLMKSQENLAMMGEGMVHVNRNECLMMFRLAFTIATL<br>LDGLNQV
DmelOR83a	MEYLKYCQTYIRGCGLASNSSPFRKILAIYFFLPPCFLIIAFSVYELWDANNNDIFLVIEVLECISSYTQLTIRKY<br>MIFTQNELMVEIINDCDQLWSFDTCGPELGKKFKQRMKNCWIMVKALVTCGFTTFILMCISARADRDNLLP<br>FLCWIPDFPYATEVLFLSQFMLLMELLYYVMATDGFYLLVCMDIHIQFEMMQEMLKTIQFDVISEKESWEKLT<br>ELAKHHNRMLHQKLNQVFSKYYVVQYFMTVAAMTVQTYTLKYRMVNIQTALKSIMYTFSLMFQSAYYLFP<br>ASNIEIEAENFSTEIYFLNWQDHEDVKIRKHILFMLMKSQENLELMGEGMVHINRNEYLLMFRLAFTIATLL<br>DGLNQL
DmelOR83c	MEYLRYCQSFVVGTGLVTNSPSFRKFLGWCFLLPLSLIVFAFSIYKIRDTDNDIFLMIEVMESISSFTQLMIRKYII<br>FIQGELMLEIFNECENLWSFDLFGPQLSEKFKQQMKNCWTLAKVLITSGFITIVLMCISALTDKTKSLPFICWV<br>PNFSYAHELIFLSQFILLIELLYYVVATDGFYLLICMDIQIQYKMMGKMLKSVQFGVISEEESWEKLVELANHH<br>NKMLHEKLNKVFSKYYIIQYAISVSAMSAQVYTLMYSKVVIETALKSICYTISLLLQVAYYFFPASNMEIEAEKF

	STKIYFLNWQDNADAKIRKHILFMLLKSQKSLEMWGEGMLHINRNEYLLIFRLGFTIATLLSGFK
DmelOR85a	MEYNQDFKYAVAWNRTSLRFVGLWPEPNDGFFTKLKGWLGAWSIFMTIYLPQSTLAYVNWGDMNAVIESL<br>SINGPILIAIIKIIIFRHYRDVLKLAIVTMTKDWNELRSKEEYKVMLKTAKISRIISVTSTIITNTLFIAFVFFKIWIG<br>MQLMKRTDLDPRLSVGLLYPGYLPFDSRIMTYFIPTWIAQCFATCFSMTAYAAFDTFVSCMVLHICGQLAVIG<br>VSLKNLINDDVKVDSKVFWIKFSEIIKHHEEINKLGLMIENSFNSILLPQMFVCTVTFCLQGFAMITSFIDPSAG<br>EISIFEMLFSIVYVFYTVMHLFVYCYVGDYLSFESTLIGQSYYKSKWYELPVIKSRSLMFIGHRARRPLLLTAGK<br>FCAFSRNLFLAVLKTSFGYLSMLLAVKQEKISDT
DmelOR85b	MFDFLQNLEDSERPLLGPNFWLINKTGLLLPKTNFGKLAYILVHEIVTFFVVTQYVELYVIRSDLDLVLTNLKI<br>SMLSIVCIVKVNTFVFWQTSWREVLEYVNEADKFERNQTDETRGKMIETYTKYCRRLTYFYWSLVFTTFLTTT<br>NTPLMRYWSSPIFRENLRNGTEDFPHIFSSWMPFDKNHSPGSYCTIVWHVLLCAYGAAIMAAYDTCIVVIMV<br>FFGEKLNLLRERCKKMLANDLYNHAFVIGQLHDIHVQLIKQSRLFNSLLSPVMFLYILMCSLMLCASAYQLT<br>SATSTAQKLLMAEYLIFGIAQLFVFCWHGNDVLFKNANVSLGPYESNWWSSSPRVRADVLLLCGQLRVRH<br>VFTAGPFADLTLSTFIKILKGAYSYYTLLRK
DmelOR85c	MFEDIQLIYMNIKILRFWALLYDKNLRRYVCIGLASFHIFTQIVYMMSTNEGLTGIIRNSYMLVLWINTVLRAY<br>LLLADHDRYLALIQKLTEAYYDLLNLNDSYISEILDQVNKVGKLMARGNLFFGMLTSMGFGLYPLSSSERVLP<br>FGSKIPGLNEYESPYYEMWYIFQMLITPMGCCMYIPYTSLIVGLIMFGIVRCKALQHRLRQVALKHPYGDRDP<br>RELREEIIACIRYQQSIIEYMDHINELTTMMFLFELMAFSALLCALLFMLIIVSGTSQLIIVCMYINMILAQILAL<br>YWYANELREQNLAVATAAYETEWFTFDVPLRKNILFMMMRAQRPAAILLGNIRPITLELFQNLLNTTYTFFT<br>VLKRVYG
DmelOR85d	MFEKALRSANFYMRVIGIPTDIRDGNRTLMERLRNRWFYCINFLWLNTDVAGEITWFVKGLLNGSSTLIENT<br>YLIPCLTLCILGNVKTFFTIKYANHIIDLVAILKDLEIKNNAARKNETEIVKERLKFLTTSNKFLLFVIGTGIIAFG<br>IGPLMLTASIYFSSGDMKLKLPFLIWYPFDSSDIRYWPFVYVHQVWSACIACCAVYGPDCFYFTSCTFIHIHFIH<br>LQNDITNVIVESSRARKNGLYRGCHQAFLELTNRHKDLIRCVNLLEIIYSKSTLVNVVSSSLLICVTGFNVMVT<br>FCWFAAPFASFLALGLVQTYLLCYYGDTIMCSSTEVSDAVYNSTWYGTNISQMRDYLFVMKRAQKPCKLTA<br>YGFSDVNLRTFSRILSTAWSYFALLITIYRGNGQQ
DmelOR85f	MFEPSEEILRELKISRETYLENDFFLARLSGLCRWNRLFSLFYFLSMTYAVASAVGFVLAAFSEKDRDQVLENI<br>HFVPLIFNMASQAASYHYTQKEYLQLFRAVDSGFFNYDGDLDIATELEITEVKSVAKNRKKKFGHFYSMLML<br>VAGLGQILKKPLLYVLRGGGTKPVDGENNLVWEAPYGMYVPYADHWISYLTGMFLGYSACTFVSITAVGSV<br>LSYQYMSEELLAEFKVVEITFSKCLRRAQTMYENRKNVLKANGKTSQITMKDCIIHCMNLSVKHHQHTLR<br>MMNVFKDLMFFPLFMVIFDGALVLCISAYLTISDDVSLNLRITMPSVITAEATLAFIFCYYGEKLTEATEDVGD<br>SIYNSDGWVQHSDIIRPYALIVKSFCNIPNELSAAGFSSVNHNTFGNVRT
DmelOR88a	MFGHFKLVYPAPISEPIQSRDSNAYMMETLRNSGLNLKNDFGIGRKIWRVFSFTYNMVILPVSFPINYVIHLAE<br>FPPELLLQSLQLCLNTWCFALKFFTLIVYTHRLELANKHFDELDKYCVKPAEKRKVRDMVATITRLYLTFVVV<br>YVLYATSTLLDGLLHHRVPYNTYYPFINWRVDRTQMYIQSFLEYFTVGYAIYVATATDSYPVIYVAALRTHILL<br>LKDRIIYLGDPSNEGSSDPSYMFKSLVDCIKAHRTMLNFCDAIQPIISGTIFAQFIICGSILGIIMINMVLFADQST<br>RFGIVIYVMAVLLQTFPLCFYCNAIVDDCKELAHALFHSAWWVQDKRYQRTVIQFLQKLQQPMTFTAMNIF<br>NINLATNINVAKFAFTVYAIASGMNLDQKLSIKE
DmelOR92a	MFKIIKNIIVENDALKQVEKPQEFQYMKWVQYHLKYIDGWPNMDMNKKNVSKIRFHKRHLLVVEQTITFL<br>SQMFYIVKNYGKLSFFEIGHSYITALMTIVIFSRSVVTALGRYRKIARYFVSSLHLYHYKDISEYALQTHLLVHR<br>LSHYYTVYLISLVVTGMLLFNITPLYNNISSGVFNSPRPENMTFQHAVYLGLPFDYTTDIKGYFVVFILNWHLS<br>HIAASYFCTFDLFLSLLILHLWGHLRIILNNLKTFPKPYTNNSMYTEEENQVVLLKLQECIRYHNFIISFTVMM<br>SNVYDVVIIVYYLFHQVTGCLLLLQCSTLDWESLSRYGPLTLIIFQQLIQVSMIFEILGFLSDKLPNAVYSIPWEA<br>MNVTNRKLVQVLLQKSQKPIQFKAMNMMSVGVQTMASIIKTSISYFIMLRTIARD

DmelOR94a	MFKINPEFAIAYTKLTVTLVCSWPPGRNSSRLDFLLFRIKWWISWLMGIFLVIPLIYAAYIDRRNVLEFTKSLCLAVSCGQCAVKMFFCKLQHHRIKFLLDEMEEYVKVAEPFEREIFLGYIKNCGLVHVTLNVCSLVASVGVILGPLVLPQSLPTEAKYPFSVENHPNYEIIYIHQAFAGILCSSIGSIDCQIAMLLWFSIARLELLSLEMKNITNVYQFHNCVRKHQFLLWFVDEVIKAGRNLVATTVIMTTFAVILGGVHIVGNEPMLVKLQFVIIVGGFSMLLYVTAWPSEILTRMCQNIGWTIYNSEWIRNSKELNKGIEFVIQRSNKPAVIYISGIFPAISLNYYATFLSKTFSYFTTFRIILAKLE
DmelOR94b	MFKKRKFDDRFIVFKKIFFEFAYSKEMKIYNMICLVFHSFSFVLQVYFIVQNFSVELITRYGCILAVFLYLIAAMSFAIFIEKQVKMLEVETTSFFWPIDCCGPQVKKLIYDRSARINILNYFTLAWFTLFGIIMLPVWGDQSEWFLCIQVFQQYFGSCWKLFYYFYFSTCPMIAFTAFRLPGLMLYGILHIDLQLVLIYQKIAQLSARRIFSENIVDNAHYQKTVFRKLKLCISHHVKLKTCLRKLIELIQMAMPVFIFVGAVCSIAVLFFLLYVFSSSSHILKIRLAISVVSNVLIVYTFSAAGQAIADETSHVFDTLMTCPWNAWNNKNRKVLLIIMSNTLRPLTFTLAGITLNYKFGLTMIRISYTYALILYNLN
DmelOR98a	MFKSVVLFALLAVAFAAPAPKPIANPKAKPTFYATAYTVPAAVAAPVIAYAEDLDGHVVYADEYYEPAVYTAYEILQAFYILMNMRALKGTLNNLQKNEMFQPKNLKQIKMVQPSLNEWTTLFRMFWFSGFAAMFLLSLFPLVDGTYKEFRLPFLAWYPYDTKSSPFYELMYLHQVISTYTVGVVDISADTLIAALNMYVGTQCDILCDNIRDIDGPVQEMDAKWKKCFTHHKEILKFAEHCQKFFNWIVLTQFCASVISIGLSMFQLTLVVPLSSEFFMFIFYLGAITVEIFMYCWFGNEVELKSSNILYATFEVNWVDAPQEVKKSILIFAIRCQNPIKMSSLNLFYLTLETFKAILRTSWSYFAVLRQVNARE
DmelOR9a	MFKVKDLLLSPTTFEDPIFGTHLRYFQWYGYVASKDQNRPLLSLIRCTILTASIWLSCALMLARVFRGYENLNDGATSYATAVQYFAVSIAMFNAYVQRDKVISLLRVAHSDIQNLMHEADNREMELLVATQAYTRTITLLIWIPSVIAGLMAYSDCIYRSLFLPKSVFNVPAVRRGEEHPILLFQLFPFGELCDNFVVGYLGPWYALGLGITAIPLWHTFITCLMKYVNLKLQILNKRVEEMDITRLNSKLVIGRLTASELTFWQMQLFKEFVKEQLRIRKFVQELQYLICVPVMADFIIFSVLICFLFFALTVGVPSKMDYFFMFIYLFVMAGILWIYHWHATLIVECHDELSLAYFSCGWYNFEMPLQKMLVFMMMHAQRPMKMRALLVDLNLRTFIDIGRGAYSYFNLLRSSHLY
DmelORco-receptor	MFLVFMVTVGILFQGAKLLWNKNETTFDLLFSLVHGRENHCTYTGTPYDEFLKSRRHTFLQYGPTLFTYTNITVMSMYVWLPPLVDCIMGVTHHPNDPHNGIATTLGLPMWTPLDADHSWTSYFIVIYMQITFFRIVTVSWAFGIFYTSIFQLTLLDEMRLLRKSLQEIDTRATQLFTLKYQRQPVNRKTKEYDDCYYECLKENIIHHQFIRKIYGEYQKRVGWTIAIPIYLSSVVLALSSSYIMLDNRNALKRSMDVLRIIGDSLLTFLTCHAGEMIAYENDELRNELYNTEWYDRSKKVKQAISICLHITYMPMRLYGGYMVFNHELLSTIVNSAYSVFNFFRVIQKSAK
LmigOR1	MFREQSLMNIPNDDCLWPLRFIFQNLYKIKVTNFIIKKMLTNLTILLIIQTYLFLRENSGSYFLKFLPAFAGSFFFLASYYSTPIIGKCVFDAIENAQLWQLNTSSPKLTKQVRREAFFTNLVGIVASILAILSATAIGLPSGDKKDFMFFLAIYESFTPIWKRLVLWNHTFDIAFVALVAIAPCYNVIYLLTHVRLQCLMLLHHVRNLNCDYKFSNLFDLFHDDKYQRTIEERLKFCIIRHSILLSILNKVRNELQHFIVIFATTGAVLFISIAVFCYSFEGNLRNEYKSLLLLVLIGALTCGHVVVSGQLIENVTFDHYEILRTVEWDCWNTRNKNLFVIYLQNSREIFKIRFTENLSVNFLLGISIVRALFTAFSLMRQLQLSRKE
LmigOR10	MFRERVYDDRFIVLKTIFLEFAYCKEMKIYNMFCLVFHLFSFSLQVHFIVLNFSVELITRYGCMLTVFLYLIAAKSFSIIIEKQVRMLEMEATSFFWPIDCCGPQVKKNIYDRAARQNIQNYFTLAWFALFGIIMLPVWGDQSEWFLCIQVFQQYFGCWKLFYYFYFSTFPMIAFTAFRLPALMLYGILHEHLQLILVNQKIVQLSVRRSLKENIVDNANYQKTVLKKLKLCISHHVKLRDSLGKLIGVIQLAMPVFLFIGALGSIAVLYFVLYIFLSSSNILKIRLVVITICNGLIVYTFSAAGQALADETGRVFDTLMTCPWNTWNIKNRKVLLIVMSNTIQPLTFTLAGITLDYKFGLTMLRISCSYALILYNLH
LmigOR101	MFRGKEKERPYVIKDPKDFDTDRLVFLWLGFVYDGSFFSKVRLTVFVLLLYSAPIHHMLPVILDKSTTTDEVLIALSINMLYVLLCIAWPFMIYRSPEIIRLWSTVRQGFFHYSDPLTSYERTILSKANDLIIKSTRMSLIAYFCAGFGTYLKEMSPNSMRLYNPPYPGWFPWTINSNFRFAMALLYQLSICLNTTFALEGIFLLFLFHTISFEGQISLLKQHFEDTFPPGLPTEMTHVPAFKERTLKRLKECVRHHLVIMDFHKRILSYFGICLLVYRAICTIMLCILCYLTTTGIALN

	KFLQLACLAALILYLLFIFCLKGQKVSKMSEIWRETLYEVDWWNHPVEVQKAVLLMLVGAGKTMTVYGVW<br>TPAMYSHEGISAIGQETFSFFNMLRAMK
LmigOR102	MFSFEVKLDENLFKNDVLWLSRKLCLDYYNTKLVKIILFVLSIGVAILTIIQTFLFLQRFDGRYFIKYAPVYAGSF<br>LIFLSVEHIPFALMLINSFKTITFWRIDSCGPEIEQKIKKHAMWTNICLISCTVVGLVSAIFHAMPLEDDDELFY<br>PLAMFEEFMPQWKNLLSWIYRSSFLIVPFSMPIPVYIAIYVITKSYFQILLFLSCLENLNTGFDTTSNHLLIYNNR<br>YQNTVKKRLVFCIKRHAYFSRAMNVHIKKMYVTIATFSIMGVILSVSVIAFLFSFQGNFENRYIRITTLVFTMVT<br>VSTHILYVGQLIEDAAFQVYTTLKTVDWNNWNLENRKLYLIYLQNAQIIFSIKFTQDVSINYRLGFSMAKAIY<br>SMISVMSKLRNVDYSKI
LmigOR103	MFTDSDKAYETLEIAVGSAEGVLKGIIFRTKFQKITESWQQIQQPEFQPRNEKQKSVLRRYIEVTKTFFKVYFSL<br>VYVGCVTGIVVSSWLRHKDLPTDHWLPFDFRRPFLYPYVYVHVTVGLYLNSFTNCVLDSCFYLSLLHITAQC<br>DVLADTLKNIHDLDKLNGKNVPERENVDEVMNKILTECMKHFKLIQKFTNVITDSFKEILTLQFVPTIAMICI<br>SMYKISTLHPSNTQFWFFIFTDIGATTQIFIYCFVGNLVTTTSEKLFYAAFESQWYNASQKFKKNVITVMMAV<br>QQPIIFYGWNVFAINYETFKSIMRTSWSICVALKSTQDL
LmigOR104	MFTTEFSYLSLIAFECLMSAMMCLNGLYYCINNIDESVLYFGFVVNMLYSSYKMYIVLNQSKLIWDCLTITQF<br>DFTSYGLQYRRTLDVWRNRAIKSSNIFAIVNAVVCICFVACPLVFSNTFTIMKNHDGSTSAYRLNVLNLYLFAS<br>EDTYNTHFNVFYIIEAFGTVIFVLFMVIFDIVVSTLGFALCGQLQIISNAFESVGHESHHSPNNNIENKIKLRNE<br>HIIINNISVYEDLKTIIIDHQSILKKYDEFLSIFRPTMLLQSFYEEDFTESMAVITLKAGFAIPFCIYQMYISCHMFE<br>TLHIKKDSIIFALYSCNWTEMDMKCKKLILLTMRMNNAHHQKLQYTRTRIINMEIFYHTMRVCYTIINVLQN<br>CKKAKLF
LmigOR105	MFVFIREKELKRDPNAMIGRRLVQARFAIFAGIYPDFYGWRHYYFVIFLWIIHPGLYSYFVMVYVLSFVEGLRY<br>MDVELLGQVLCLGTITVIYCLVSIYYIAKKTDVDELMMMAGKGLSNYSRPTTQEEKTILDSKEKSTYKYAIGSS<br>IMFVSVSLLHMGFLPIRRGLKGQYTSITNDTAPINKYTPLPVWTPYVCDDLTTFLISYFTQLIPGCMEISIINAC<br>CILYIGLCQQLTGNLEILVNSLRQLPERGLHMFEAERGIVEKFTPELYQNEYFLRCLNTCIGENIEHQYNIIKFY<br>KKIQSVVGFSILAIFSGTGLIISTAAYSMLLIAERERDTEIIITNSFVWSFNLFVYTVLLTLYCYYGQKVTDKNEEV<br>LEALYDTPWIEADMAFRKSVIIAMSYSQLDMTLSAMGLISASLATLLDIIKTSFSYLNMLLAAR
LmigOR106	MFYSYPYKALSFPIQCVWLKLNGSWPLTESSRPWRSQSLLATAYIVWAWYVIASVGITISYQTAFLLNNLSDIII<br>TTENCCTTFMGVLNFVRLIHLRLNQRKFRQLIENFSYEIWIPNSSKNNVAAECRRRMVTFSIMTSLLACLIIMY<br>CVLPLVEIFFGPAFDAQNKPFPYKMIFPYDAQSSWIRYVMTYIFTSYAGICVVTTLFAEDTILGFFITYTCGQFH<br>LLHQRIAGLFAGSNAELAESIQLERLKRIVEKHNNIISFAKRLEDFFNPILLANLMISSVLICMVGFQIVTGKN<br>MFIGDYVKFIIYISSALSQLYVLCENGDALIKQSTLTAQILYECQWEGSDRIEIQSFTPTTKRIRNQIWFMILCSQ<br>QPVRITAFKFSTLSLQSFTAILSTSISYFTLLRSVYFDDEKKLD
LmigOR107	MGCLQAKIEEWSDAEDEEMMNSIRRRFGLFCQLSLAYPSWKPGMRRWTLLLFFIHTVLLTTHSVLLGISGVL<br>MVLEWNMELASLTIHFSVILFFAIFIVYWMNSQRPLYTRQNMLMVTDVGSYKSGRIYDDDFCVEERRKNKRE<br>LLYYITCPVFISLTAGYVLTVPYIQHWLYSSGESPYTANMVNKHLPLPCWYPFPTHEGVLHLMVLLLQLAAA<br>LCGAIVLVAILLLLIFNTQRIRYEMRVVGYSLTSIFHRAKKMFLEQNPHRKGDNLRDDPGYQKVIGICLKDTII<br>HHHAVSEILSLFTKQADMPAALAYTIGTGVIAMCLFNILMALRDENYTSVVLFSMLVFVETLVMFVMSLCGE<br>SITSESVNLRHELYFTKWYNLDIENRKTLLNIQTNLVEPVIVSALGLIELNMN
LmigOR108	MGDGCEALGASVSVLRLLGLWVPTESSGAGGKAAYVPGALSCVAIGLLSLSCASKLFIDTPTELTELTVCAYL<br>FVIITANFVKAFCLLLQQGTLHELVTLLVEAKKKNVIDVQHNEDIRSLYGVMSARLYRYLQVMIVVSSVAWLF<br>VTVVFRVITAGSTNIEWPTPLPIWLPLDTQRSPAYELVYVAQVSCAVVTAATMLGADTLFFHLTLMIVAELQV<br>LNDNVSVLGRPAPPSADTRQVVCTMNGAGERQTHPPGGVAERQSDSDGAVSLSDHQGTAAAESKYLVLIEI<br>IQHHQIIIKMVSLLQTIMDYSVSVQLLTNVLDMCFLIFTMSELLHHEKSLHAVLQTILSLPCLLCESGAFCMFG<br>QMIIDQSENLVHAAFSCEWLEADGRFRKPLYTFMLMATHPLQIKLGGTAKLSRSTFLQALNGSYSLINLLYHS<br>RRPVG

LmigOR109	MGDRMVTRGHFFDFNIKYLFYVGLWPSNEAKRIEKIAYKIYEYQLHVLSLIFLVTTGIGTYKNHKDIIALLTNL<br>DKTLVAYNFVFKVIVFVYKREELRKLIEQIVQSGDQITEDRKALMAKLVIVLTGISTVIITAFSCLALFEGEMTID<br>AWMPFDPMKSKMNLFAASQILAATFVVPCGYRAFAMLGIVCSLILYLRDQLVDLQNKIRDLRFATGNVEKL<br>RDDFKLIVKKHVRLLGYSKVIEMIFKEYFFIQNMAVTAELCLNAMMVSVVGLEQKTLAASFLAFLSVALLNA<br>YIYCYLGNELIVQSEGIAMAAYESSWILWPVDMQKDLLIVITAAQKPMKLSAGGMAVLSVQTYSQTLYNGYS<br>IFAVLNDIVN
LmigOR11	MGDTTVMVNAIDVKVLRTMDIYDKPYYKITKISASLIGRWPYQSSRQSLVIVTVIWSAFILQAIPQIIAIVTHFD<br>DREVLLEALAPFIIDIMFVAKYMNSIYNAELMVTLFERMKKDWKLLSSAKEKRILEYHANIGRLISTGYAGFA<br>YTTTAIFLSEPILPRIINYFSKSNESVPLKFALPLEYIIFEKENHYWLMLAITNMFAINMIIVTISCDIMFITFVQHV<br>CGLFAVVGFRIENSPTGKITDSNHRAVSLRKNSQDFSYKHLVSCIRSHRRALEFVKLLEETFTGTFGVVVALNL<br>PMISITGLQLITQSNTVEQTLKYLMFALAQVLHLFFDCFLSQNLTNMSSRIPQCIANMKWYNISKNSQKLTLL<br>MTMRSQTPCKLTAGKIMELSIENFGMMMKTSGSYFTMLLSMQ
LmigOR110	MGDYDFRAAFAFEKAIFSLSGYYQRQAGFSSLIICAIASLITIAQFLSMVMQIIVAGNDLTVLSETLLFFMTHFT<br>YMCKLVNLLFYKSKLLHIEDLLSRPRFYGFSQNELTIIKDGIEATNTVANLFRIFCVLACIAYGLVPYLDHTKA<br>MALPLPGWLPYDTTKYYYPTYFFQMVAVSITASVNSTIDILTWKLITIASVQFDILKRKLKDLDYKLETTSLQIQ<br>FKTCVKHHKEIVNYVKNVEKTFSKGIFIQFFASVIVICFAGFLIIITPVLSMQFLYLTLYFMCMISQVAIYCWYG<br>HYVMTTSDEIGQDFYMSNWYESDVAFRKDIIIFMERVKKPVTFTAGNFITLSLVTLTRILRSSYSYVAVLQHLY<br>NEV
LmigOR111	MGEHDDPFSVLRMLLYINIDSKITNVLTVLLVIIYTLEHCLEINYMITNFNLNLLIRYGPVTTFSLLMTVGALIP<br>VALGKEIFEVVAFHRNTCWPLNMIREDAQTKLKRQCYVVNGCLFVIVLLLSTLIIYLPFFGNQRELLLCIQVF<br>EKYFGKWSFIPYYFYFVGFAFLYYQFFRISFGFVYIFLETQLQYFLIEEYLFETYHIDNLKHWKYLQDTQYQEEI<br>GKSLRLCIAHHNDLKKLVKTSVKLTITAMPVFLLLGVFLYISSFAHIINFADTMTNILKLRMFLFLASTMSITIM<br>FCWTGQQLINVTSNIFFTLSGAPWYYWNLENIKILLTFLTNCTKNESIVLAGICLDYKMFVTMCRISFSYAVVL<br>FNFRKQSLI
LmigOR112	MGEKPYTMDKNGEMEIDWLTQEDKNHINFFDQFHCWSGMWRSKKSIQWTYFWLSQMIAFSLVYFYSFYFL<br>LSELELLAHLIHHMMVAADDIMYIYLLNIHRIRVETIHDYASKTYNYDSGIVREKHKKLMTEQLKLYPKMSK<br>FIFLTTVATAMSLEVNYLLEATYLKSYVTMYPMYLPIDLNHPVTYTIVVFLQHLQVFISMILCGGLMSILFVVW<br>SHLKVELDTLTFAITHVDELVEEKLRHFSYTNPADKEKAKAEFYNGFCYHFARHHAAIKRYFGAFQISCKVT<br>MTFVLISGLICFACVGITSVTENIGIKLKFFVVMVIQTLIIYSWSWVGQDISDKNAALQNIIGGTHWWKMPKT<br>CHSTLKLMLVGTSRPMLLYTLIGQPNNIDSFMDMTASSYKIFNMVYQVKFSS
LmigOR113	MGERGEAPAGLLGPEAAVLRLLGLWRPRERQGQGLTPPAVVAAATIAAVAFIPAGVVLRLCGDFPEEIEETA<br>HCSYICIVCFGCIVKAVLFVMEGDTVRELVHLLQATRAEYGSDEGSDRIRSGYQGTVDRMYRYFQVMALLPT<br>LYWICWPLVAAAVSPGEASGVSGARQLPLPFWLPSGASGTPTYHLLYAVQALSLSLTVASAVCLDVFFIRLMM<br>MLAAELQVLNENIAAIDGCRASGSAYGREEEEFDSLVPSDDRALEPTKKSAANFSDDDLFSRLLNNILHHQA<br>ILRCIWLLQTAMNVSIFILLFINMANLCFNMFVTAGLLQDGRNVTKAVTAFSPVPGLLLQTAMYCLFGQITT<br>DQSEKLLHSAFGCGWDDCDTRFKHNLLIFMLMVGRPVEITVGKTYKLSKEMLLQVLNGTYVLLNMLFHV<br>HSDDHI
LmigOR114	MGFFTSVDMTDVQRLRVVEDSKRRSGLSDLIGRCGGYRGPLYNEHYSKNILFRIYVHLTDLAVWINYITMIA<br>AAMKSQSVLEFAMVGFPISAESLSLFLSYYSGYKNAEMTEVLLGFDDCFDDDPYPQHLEMEIRKSADYYHHF<br>SRSLLWLQVFTMQIYCFLFPVTNELMHDFFRPRALPLPSLYPCDWKESRSCFIMIIFIHFLGATYVNWKIIGFGE<br>VFFSMVSRQVALFRHLNHNLNQILTSVHVSTDGTVIYRVDKEIDHIYIKSALRKWIKHHQNVMTQYDRLQTL<br>YSWPLFVHFGLVSGALCCSAYATSDETLDFDANLLCGGFLVGQMLELFYLCRMGDWITIETNELTLALTGSY<br>TFVLDRIESQMLRIILSRVHRPSVMRAVGLYPLNTSTLKMLIQSTYSYYTMLKKVNRG

LmigOR115	MGFNFQSRTNKILRRLRYVGTWFENDSYDLYFLYAVLLNIYYNLHNIAQTMNVFYHLDDIEEWSSSGLLTLTTLLTNFKAYCVLTNKKRILKLNQILTRSVFQPRSDHQVKMATDKFKIFDTMYSLHSSGPTLTVVFFSLYSLAELENRKLPFNAWYPYDFKKTPNFELTYLFQFTACMVQALIHVNTDSLAFNFIAILVIQLDFLADNLRNMCQKAESMEQSLLDCIRHHKEILACRNELYHILNVNLFGQFILSTTALCMTFLQMTVVNPTSTHFIAILVYGMALLVELLMFCWWGNELIIKSQLIPQAAFESNWMEGSIFFQKNLVFFICRAQKEMMLYAVGFFRISLNTFILVSTSVSENVVY
LmigOR116	MGFSSWIAQQVVTEDEFQIRLKKYGWLHYLFQFSLVNSCYRSMTSLLMYYISLMFSLSIVIFHLFCYIKTALNAYSFGRADMSVANVHSVVLGIFIISVLTSYAIDKTKTSAIEQLYLESFLSYENEYPAPVTLFQKLMTLAGKMGIASGGMGLFFNVYVAAPMIDFRFWKESCVVKGINFCLALPHYYPYNSEDGWSFYLTELFQLMFGVYRISVFCAVQVTLTVWPLHLIQELTRLKTSIEHLEERIKKRYHRITMRNVEKVNLITMCMDKTFNECAKFCINENISHHHNILKYHERIDGIMALPSFLAYTTGTLTMGIAMVKLLSVEGDTTLGGNLAYVTVLAAEIGFMLLISTMGEAVTIQAEEIFNEVSHIPIENYDLEFRRNVIIFMEGTIKPIALSSSKFNKCNMEAFGNVLNAAYSFYNVTSASAKLEK
LmigOR117	MGHAIMTEILTYLTLMGFWPRSPKSSKASAFLIILSTSFLFFGILFYLIVNRQFGSSEIDSIETITSQFGVLYYLILFTWKRNDIVEIVELLSDFSKFGKPPFFDQRSTRLNYRLSCIVLILIVANIVVAALPVIYIDSCHKANEQLNLTKTCGLIAPVWLPFDYNEYPRKHLVFAWEVYCCVMNYVGSGIGALTMVGTMEHVIIRIEQLKYIFPKILDQPNPRIREQMLKNWVRYHLALFEIGRLMNDAYKWSLSVIVLCVGALFACIGISMLQSTASQINSICLFFGWFPSIAFLCMWGQRLLDSSLSVGTAVYSSRWYDMDVAFQKSVLMILIRSQKPIRISVGPFTHLSMLLLLGVFQSAYSYINLLNATS
LmigOR118	MGHLLTPLHWTGVLRHPRYSHQSPLLFRLYTAAVSSFALCFICSEAAALVHDDTGDMDVIILLISTINTASIWIIRMVHIAVFERDFHKLAVQVGHDFAEFLTWEDIPVMRAKSRRVRRFTLGVVWFGVSACSYFLVSPVSPEGLPFILALPFDATTPLGFAVSWLFCTITCMHAVVMTMALDSFNVSLIAQLRIQLTLLSNKIVSLAREMSQRPIYSPETSSYHELHSRLEKCVRHHQTIIRNVDLLERRLGSILLAQSISIGAVACFQMFQIATSANGVQQVGKFGCYLTTMLTELFVYCWFGDDLITESEKLALAAYEALASLQGCPMPITRSLLLLMQRAQRPLCITAGGFFPLSRESYVAVLNVSYSFFAILRNFKEEEQQPD
LmigOR119	MGHSSEERESLEGPGVVLRRLLGLWRPRGRLARGLDMLLAGVTLVAISFLVVCVALKLYADPPEELEQIALCGLVASLCIGFFFKALLFMVLGGTLRQTVRLLEDTRLEFFSGDKNETTRRRYQQLSRNIYNYGQMVAVPAAIGWITCPLLSRLLTHTGDDQHEVKRQFPVPVWFPVDVYTSPTFELLYAVQSFCVLVVAECCIATDIFFVHTMLMVAAELEVLNSNLCAMGDAKLQMKRVKEEEAVSRYKTKGRRWAFLNSDQPVGEHGLPENAVHEWLHEQLVKNVRHHQAILRSVSLLQSTMDVSIFILLFVNMANLCASLFVAGVLVHKEGNVGRALNALVSIPALLYETTMYCIFGHVMTDHSERLMYSAFSSGWINSDARFKRSMLIFMMVTMQPMNITVGKTYTLSKQMLLQVLNGTYGLLNMLYHMHGSE
LmigOR12	MGHVIMNEILTYLTFLGLWPRSRKSTKTVAYLIISSTSFLFFGSLFYLIAHRKFGSNEIDSIETVTSQFGILYYWVLFTLKREGTVEIVERLSDFSKFGKPRFFDQRNRRLNYLLSYFVLVLMVAIGGVVALPVVYIDSCHKANERLNLTKTCGLIAPVWLPFDYNEYPRKNFVFAWEVYCCIMTYACCGIAALVLVGTMEHLIIRFEQLKLMFPEILDEPDRHTRQQKLKNWIEYHLTLFDIGKLMTSNYTYCLSVIVLCVGILFGCIGVSTMQSASSHNSVFLFFGWFQSIGVLCIWGQRLLDTCLSVGIAVYSSRWYDMDVSFQKSVLMILIRSQKPILIYAGPFSYLSHLLILSVFQTAYSYINLLGAKG
LmigOR120	MGHVIMNEILTYVTLLGLWPRSRKSTKTISYLIILSSSFLFFGSLLYLVVHRKFGSNEIDSIETVTSQFAVLYYMTFFTLKREGTVRIIDQMSDFSKFGKPPLFDQHNKRLNYLLSYFVICLFVAIVGVVALPAIYTGSCHKANEQLNLTKTCGLVAPVWLPFDYNGYPLKFLVFAWEGYCCIITYACSGISSLVLVGTMEHLIIRIEQLKLMFPEILNEANRHIREQKLKNWVQYHLALFGIGKLMTATYTYCLSVIVLCVGILFGCIGVSTMQSASSNNSVFLFLGWFQSLIVLSVCGQRLIDTCLSVGIAVYNSRWYDMDVSFQKSVHMILIRSQKPILIYTGPFSYLSHLLILSVLQTAYSYINLLSARG

LmigOR121	MGKMNDPLQFICKMTLGAIDYKFVQLCLLLTFLLHSFVSLAFSWELILTFDANLLVAYGPIIFTFGSGIGGFLVI<br>YKIRGTLKLVLKKCSLPALDSNPIIFERIQSDSKIILKAIYFNNILMVVTVYLHWPVSGQNNNIYYAIVLFERIEA<br>KFSSTFCFLYYMTFFFVVYLVAINPYLLLYMSTYARYHLCYINELLTNVDAAWSKYGDYQLIHNNVFQNIVSK<br>QMKKCIERHCLFQSLVKEINDVIYTPLLILMTLAILGIVSILFSIIVKRQETSYCSLSSTLLFAFLTVSFVIISGQLM<br>QNECEKVIERAMCCRWTSWNYENRQTLLIFLLNAQRPSKIENVFIRCEHPFILTAGRFVCSLCGFFFQLARIRE<br>QIVQN
LmigOR122	MGLLFGRTPFYIKSGWLRFSYKSLPFVYALILAFANWFCLLYYIDHLHNTWNQTLGSNVSFSGVLFAILVFSQP<br>FSTIFMVYSWAFEVPAIVRGYNSTAVLEEKISIVFPLYKQKPSKRNLVTFLIAFLFVVDLIIAYFLLKTRFTETPLILI<br>LLIIVNVVVTFSYCVLWCFNCYFISDLAVKLNKYMLQCLQVRENCAFKIKTCRKIWISVWKQSQMNSQSIAV<br>SLSFALVLYGMIFVVGCYGILTSIRNQNILETLEMSPYVVVTFTIIACVFETSYQASHKLGATFLDTMIILDKDRV<br>DHECVEEIDKFVDTINRTRIAAITLKDYMTMDRTLVVSFLSYSITYLIVLIQFQDKNEEPSVNISTPMRNNTL
LmigOR123	MGLNTIKEFFVNVKRRFQDVSIDSLLWIVNIVPSLAGFSIRSDRVSAPFWIVHWSLLVYVYAVGNAVYQWKFA<br>NEAIDYITSFINVSLLILIGNNSWWFLANRRLLKSVLHKIEVNDELSRRSEQSRLKHKKLLKIIKRIVLVFYMSN<br>YVNASFIYLPNRVDVLNNYAMTPCVGMEPLTVSPNRELCLTILCMQEFSIMTVVLNFQALLLCFIAHTAVMF<br>QILADEIMALNNYENLEEHQAYVKEMLPIFVKRHSLTLSAVDNYKSLYSVPLGVNFGSNALTILLILYLPVLE<br>WFKFIPIFVFCFMLFFLYCFLCQKLVNASEAFETAIYCCGWENFALREMKMIYVMLHQAQKPVELLAADIVP<br>VNMNTFATTLQAMYKFVTVVKF
LmigOR124	MGLQNEHSIINLQYMKITGFYQLLIPSDGVKLFNINIYKTAFIVQILILSVTTIMGFYSIYAFINDVNQIFNYTIIIF<br>AANFAIFKYYFIIKNAKTIWNFMHNMMSTNFLCYKGHTKEIYKIGRNRSSTLILISFGLWSNIVLYWSLSAILS<br>KNSYFKLKFKDGVYNYRSNAIDLVYPVTDTFYNKYFLVFYLLETILLIFWSQMMWVFDILMISVCISIEHQLKT<br>IAASYSLIGLNHNELTRNNKSTKNVEAILDLEVLIQDQQNILEKTKNMYQILKPATFIQLAAESFQIILQPCMI<br>LKLYFDGSLSPTLFFKLSFPEITYLCHLFLTCYLFSIVNEQKESMNFALYSSNWTAMSVKFKKLLLFTMRMNDA<br>ENLKMQISIKRMVNMEMFADVRITNIMIQVILL
LmigOR125	MGLWQPRGRAAQRVNALLASLTLGSLCFMALCVTLKLCADTPQEIEQLTLCTLVASICVGFICKTALFVIQG<br>DTLQQTVRLLEDTSEQFCTGDHNRLTRRRYLRLSNNVYYYCQMVAVPAAILTNTDDEEQQLPWQLPLPAWF<br>PGDIYETPHFQILYVVHSFCVLVAVQSCLSIDIFFVHMMLMVAAELDVLNCNLAAMDHITVQTTRNEEERFIP<br>RYKRNGRRLPLLNSGQSLAEQTLSQDTAHKDLNQQLLNNVLHHQAILRSVSLLQSAMNVSTFTLLFVNMA<br>NLCSSLFVAGVLLQKEGNVGKALNALFSIPALLYETIIYCIYGHIMTDQSERLVYSAFSSGWVNSDPGFKRSML<br>IFMMVTVRPMAITVGKTCRLSKQMLLQVLNGTYALLNMLYHVHRSE
LmigOR126	MGNFDDPFLVLRMLLFIDIINYKITKFCSVLLITFYTIEHCLEIYYIANNFNINLLIRYGPVTTFSLLLIVAAVFSG<br>ALGNEIFKIAAFHCKISWPLDMIRNGAQTKLKKKCQTINGCLLGIVLILVSGLSINLPYFGNQRELLICIQVFEE<br>FFGEWSFIPYYFYFVGYPFLYYYFFRMCFGFFYIFQEAQLQFLLIEEYLLKTYQTDDLKHWQYLQDTQYQEEIG<br>KSLRLCIAHHSNLKKLVKMGVNITVTAMPLFLLLGILLLISSFAFIINFAGTMTNILKIRVFLFFASTISITILFCW<br>TGQQLINVTSNIYFTLGGAPWYYWSLENIKILLMFLINCTKNESIVLAGIRLDYQMFVTMCRISFSNALVLFNL<br>RKRSLV
LmigOR127	MGNKVRVYLNEGNLPREHGSSLGEGGRGLKLKGGMGGEGDLLEDMGTLEGIDVLENSGHKADYMLDGG<br>RGSEGVAGVQRRVAEMDGDGRSSGSVHLRWCCRQVERDFAEFLSPEDVPLLRASGRRLRRVVRAYLWFGA<br>AGCMWWLLYPVACFGLTVQGVPYQMLLPYDVARPAVFAANWLFCTLPTLHVAVMTMASDSYSVSLMVQL<br>RLQLQVLGKNLVALARGAEANHQKKCVKHGPSAEGLVRRSQLEDAIRQNIRHHQTIIRNTELLEKSMGAIL<br>LAQCLSIGATVCIQLYQIAVHAQGLVDAGKFGCYLFIMLAQLFVYCWFGDDFITESLKVSTAAYDAVTSLEGS<br>SCSTKRSLVLMMLRAQRPLRITAAGFFPLSRESFVAVVNMSYSFFAILRNFKDEMNS
LmigOR128	MGRASAEVSNSRPLLGPGAAQLRAMGLWRPGRSLLHSLAAALMLACLAWVSATAALRLLIHPPAELEEVA<br>LCSFIATICSGFTIKHSERIIVAKVDRIPTPTTVVQVYMSTSTADDEDIEEMYEEIKEIQKVKGDENLIVMGDWN<br>SVVGKGCVVPALIGWTFFPLVSRALNDSGEESPGAVADWQFPVPHWVPVDMQRSPTYHLLYVLQSFCLLVA

	SQSTIAVDLFFIHMMLMVAAEIEVLSENVSAMGKIDSGLVALDDEDCGPSTKYLLSYRDGNGRISEISRKDDIS<br>EDQVRALLVKNVQHHQTILQIGEKIQFCRISSKRRHRLVGHVLRLEGIVNLFLKGSGVGRRYFPCLIDLTYQV<br>EHFCSRLALLMISVLLQFYSRGFVSTQILQCSVKFLSHANSKERNVDHGDEGFVSHVVFHVRRALLLFLTEAV<br>RPVEITVGKTFKLSKQMLLQVLNGSYALLNLLYSIR
LmigOR129	MGRLFQLQEYCLRAMGHKDDMDSTDSTALSLKHISSLIFVISAQYPLISYVAYNRNDMEKVTACLSVVFTNM<br>LTVIKISTFLANRKDFWEMIHRFRKMHEQSASHIPRYREGLDYVAEANKLASFLGRAYCVSCGLTGLYFMLG<br>PIVKIGVCRWHGTTCDKELPMPMKFPFNDLESPGYEVCFLYTVLVTVVVVAYASAVDGLFISFAINLRAHFQT<br>LQRQIENWEFPSSEPDTQIRLKSIVEYHVLLLSLSRKLRSIYTPTVMGQFVITSLQVGVIIYQLVTNMDSVMDLL<br>LYASFFGSIMLQLFIYCYGGEIIKAESLQVDTAVRLSNWHLASPKTRTSLSLIILQSQKEVLIRAGFFVASLANFV<br>GICRTALSLITLIKSIE
LmigOR130	MGSFYQGITSPSENALKKLQISADTYVENGFFIARFSGMYRWSLLYSISYFSCMTFGIVAAVGYILNVSTTDEW<br>DKFLENIHITLLIVNMEAQGVAYHYDQNGYIEIWRAIDKGFFDYEGTLDEETDEEIAIMKSELRNFKKVFQH<br>NYTMLMCITTVLQFSKKPITRYLIGGGTVDGKNNLIWEAPFGLYFPFADYWIPYLLGLFLGNACGLLILITAL<br>GSVLQYIYMSEALIQEFAVVKKTMSKCIERAEQIYRNRSNSNQVEHQQWTMDDCIIHCINQSVKHHQITLR<br>MMNVFKKLMYFSLFAIIFDGGLILCISSYILINDEVGITFRLPMPCVIAVEASLALVFCYYGGKLTDANTDVGN<br>GIYECKRWMDHSKILCPYALIVKSYCNVPNELSAAGFTNVDVRTWGNLLSTAYSYIGFLLST
LmigOR131	MGSVTAKMDKWEKDEQEEVMKLFKEKYGPLIQLALIFPSWKSSSRSSTIMIFVLHSVVLLFHWMMIMISIKRS<br>LESEWNFEMLTIWIHFAFIVFFVFIVIFCVNNQRSTYFRQYQIMSNDIGHHRGADGSIYETDGCVSEAKNIKRE<br>MLLYMIIPVLIFLFASTIYGLPYISKWLEGMENPYTLAMVNMNLPVPAWYPFPTHAGLGHFTAMAGQALVA<br>LSVGVVLITIILLFLTNALRIKFEFRVICYALQTLFTRSTTLFLQMQHDMKDIGNSEHSYQRVIGSCLVDIVVHH<br>RAVSELISIFEKQVFFTCALGYMVGTLGVGLSLVNILEAMKVGNYVSVLIFSMMASMETLLMFTISQIGETITE<br>ESVKLRHQVYDIEWHKLDTQNRKILLIFQTAITEPIIVKAGGVINMCLDTFSNIMNLSYSFFNLMTNTN
LmigOR132	MGTSTLGARPTMRPNRNLFSLSLLLMKIQGLETPSHATLKLVSFVWKYWMLYTALHFVMVCLLGVTIGDNP<br>YYLKLETCSGMIAGMSMVYRHFVLAFNRKEVHRLMDRINALVDDVVSVYGEETIAPREKMCCGIMILSTCV<br>VSFTTIPAYAYSYLKFYTDGEVTAPYEVYMPFERDAAHIHHVVIFQMLSFLDQAITLIVSNTFIGTIVVIVSGVTE<br>KIAKRYKEINRNNFHTLKVTTNWHSEIIKIVEDTNALLGSAIMVDCLLSVVHISVSGYLLVKVGFESGTNLHK<br>YIFLNLLCVTIPSYFCLCGHIISVGRDKLHEAVYQNEWYELTPSDRKTLILPTWMADKGLSLHFKKAVEFNLP<br>TYLAIIKQSYSLIAMLKLMDG
LmigOR133	MGVSNGRGTVKPFLYPLVDELDYNLIVGVHLPFEYKTPSRYPLAYITVVIAFIYVSYFVMVTDLIMQAHLLHL<br>LCQFNVLADCFENMLNDCVKGFEGPLVSLHEYIHPLIDEFEYNLMVGLRLPFSFDTPLRYLFTYVIVLIAFNY<br>TAHYVMVTDLIMQSYLIPLICQYAVLADCFENILIDCSNDYGDHARRNDIVYSRSMELRAILSRPMLGQLASS<br>GLLICFVGYQATTSISVNIVKCLMSLFYLGYNMFTLFVVCRWCEEITNKSLNIGNAVYCSGWESGMTVVPTV<br>RSTILLVILRANKPIVFTAGGMYNLSLTSYTSLVKGSYSALTFLLRIQHE
LmigOR134	MGWDSKEDQPLTWQYTADSVLKYDVRILHMIGVWPLSGSQLYRCVVTVIIALCLGHFVEAVINLYTLHGDL<br>EDFTLALSNVSVVIVGILKVTFFLRHERGYCHLVRWLDTIVASQREYTRGRPHLEEAFAGAQTLAVRITRGFC<br>MYNATVVLAWVLAPLAAPPEAKRLPFQQLPFGEGSPFSLYALSYAMQGVSMLLIALISVQMDCFFTAAMIH<br>AASQLRILNSRLSDLQLGKAGLQLQGGTTLDSMYDELRLCIHTHQEITRFVEHLENVMNPIAMMQLAVGVF<br>NGCMLIFPATYSAENDALVKCLAAAPTISAQLLLYCLGAHSVREQGEAVPLSAYSCGWADASAAFRRSLLVV<br>MARAQKPLALTAGRIYPIQRATFLSLLNAGYSYYAVLRNFNSR
LmigOR135	MGWNPREEKPLTWKETANSILRFDVRILYVVGVWAMPVTKLFRAYTAFTLVLAVGYSVEAVIHIWLVRNSME<br>EVTLAVSTYAVVVTSACKLVSFLQHEPGYWRLVRWMDAVVADQRHFCEERPELRAIFDEARKRAKRYPNAL<br>RVYNTSLIISWVFIPLLAPPGLRPLPFQQIPLSETEDFPLFLFSYLLQTFGMLFMCLVSGCLDSFFTAVMIYTAAQ<br>FRILGLRIAALRQDNDEIKRQARSDVYEKPRKGAATDHVYEELRLCIRTHQEITSFVTHLESVMNPIAALQLIT<br>GVINGCLMIFPTAASSESGALLKCIACVPTISAQVLIYCLGAHAVMEQSEAVSAAAYGCAWPDTSPRCRRSLL

	VLMTRAMKPLTLTAGGIYTIERSTFLSLLNAGYSYYALLKNFNSR
LmigOR136	MGWRKYLTEMREFEDEDVRKAIRDNYSILPRLNNTFSYIDEGWVPLAIIHSIAFIIFVDLYLYLFFVTCYLLKDD<br>FVLVGVQFHYLLLALFGLVFQFHLYNSRKEVCVLHKIMAQEFFAYENNEILAEEKTKLKKHMIKQRLQLIPF<br>MILIGMIGLFIVGVGPLIDNMVGAGHDSDYLNGVYMKTPIPMYFPFEIVDFTTHYVATGFQIITVAMLALTISG<br>VVS
LmigOR137	MGYRVYPQQDLSDPSHMFSFQLNALKSTTMWKPDNQKYYIPFMILFAVNVFVLAICTVGLLLKGCSTKDLV<br>DRSEAMDIFTLTGSALYKMVFFLYHYEQLVDMVTCGLALVRNLPEGWTKNCGLLSRIHYTAGFLVLLIWGLA<br>PILKVMYGETTWAEMKLPINTYDPFDSTGFLFFLFYITGQYVLVLSAVIYMAADCYLFTSIYVAVGALQYIVDQ<br>FENMRDLNNNNKHTVADTMHDCLQECIEIHVHVLDYLRKTDKLFKSMILADVVHAVISLSFAMLQTSESK<br>GIFEGVKMVLFVQVCFVHQFLNSHFGQELIDKQDNLAKQIITDIPWTDASRKFKKSYYIMLTCVREPFKLSA<br>WNVYFLQYATFLEFSKTMIQYYMVLQEVQDEAEVS
LmigOR139	MGYVWSKLQLKIKLWDSWEDEFSIDVMRHRYRGFHRIGFIVLDLSSKYAKLSIITALLGTSFLLMATLSLLATC<br>AKMSDDFEECSGVCNLGFLSTLALAFLVNHNYFRKTILSAHHMLGKGFHDYQEPEYSSTEFEKYKTMLRKQ<br>NVALIILACYVALIGILVVVVCPLIDESLGFGWTEPYDEYGVNRQLPVTLWLPFYSHEGINHWFTFLFIEGLGG<br>AMICLSIGGSALLFTSLTIGTMLEQKRLVLSIRDIEQRARHRFQTQYKGKPGVDDEGKNVAL
LmigOR14	MHEFVINVQNETTKLYDQLNIILYILGLQGIWVDEIKLSRRFHVFFKVVTFILHIMCGMFAGLQFFAIFTQNSL<br>NSQQKSDVIVIGISNPMAYIFCINFIRNRNEIKDLFYHLAVVLKIYYNDVEIEKSMVNKIKSYLSTYVFASITILVS<br>NGIIAFFQTINSDEPFLGIITAWPDKTDTSKTASYARIGFYLFWCIHFFRISTVFAVIVCILISIKYQYKILCSYFESL<br>NKIFDDETSSHEVKEAEFENAFCNGIKIHTQIIWCVRRCQIMCRTVFSANIMLDTFVLVILMLAMVNSENDFY<br>GLCSQMSSVLVTVVLMAFFMWTAGDINVQASQLPDAIYGSGWYNCRGKSSARIRSLVTISMNKAQQPILM<br>WALGFVELSHKNFVAIIKSAYSVFSVFY
LmigOR140	MHHKLINILSKILRYNGIWPVESTTAISFKLLNLILRFFNFCIFVFLTSIIMADAIANHSDLSLITDNLCFLIGCFE<br>TMSKAFKFYTEYNNIIKLINDIYEPIDKLKTINNVEIMTRVNKLSRFECRQFYILCGVVALLISARVFGADRAN<br>REFPVRAIFPFDKSKSPNYQLIFILISYGVAFIDVSLFTLDLMIVVIMRYLTLQLEILISNYKHCRVGLIRNIARNIP<br>SNGSETVESFYEIATVTDNDDDDNGDDGIKNFVIFEIHRKDINNINTFDWRLKQCIKHHQKIVQMMVVLND<br>CFSFCVIVQIMTSTILICLNGFQILLGNDDRHLLVRRIIAINAVLLQLFFWCWYGNKMSTVADSLTYNQWMC<br>GWESEFKRGVSNSVTTSMILSLRSLELRAIGLVPLSLQTFVSAIKKSYSVLILLLTVVED
LmigOR141	MHHKNIQPMTDDYLKFIKFVSSDIFQLLPVKIFLAVVFLTHAVLDLLTIYFVLFVIEPHDFITYISVFLGEFYPLF<br>AIVMLLFRGKITDSLKHKLAMWTITSTDEKTQSDIKRQIVFFNGFVVLNSVIISIASWFYAARLSDDVNAFFAL<br>RLIHEYFPKSIFEVIYRVTNFVLGQMMCVHVHQTLYYTQHINIQVQMFKKIIRDLENESKIEQQLKFCIERHAE<br>FIKIITLTTKELRGAFVGFAFGGLLLGVAVAFYIFSGLLTPEYYLRVGAIGLASVVNFAVTIWFGQSTESHLDEL<br>MLAVGEVQWYNFSQRNKKVYLILLMNVMKGRKWRVSEEYSVNYRLGLAIVRGVYSIISVTSSYKKS
LmigOR15	MIFKATPEFAIAFTKFSTLVGTCWPNYKNAPKWKFVLFQIRWWLTFCLSVSAFLPMCYAAYNHWRNILSFTK<br>SLFDAANTSQTFIKMILSKIHYKRLQYLLYEMENYVTNAREDERELFIVYIKRCGKLHLFVMIFGFMAILIIIVA<br>PIGLPQPFPNIADYPFPVDESPAFELVYAHQSAATLHCLSIPVFDMQIALLLWYSGARLELLAREFKTVTDNK<br>HFVECVKKHQYLLWYIQEIIISSRYILATTSVTCVIAVITSGVHIAGNEPVGFKIPFAGASSIIAIILYISAWPSEHLI<br>HMCEGVGTALYESEWVQNSKALNNSMLIVMHRAQKPSTIEVIGVMPILSLPYYATFLSKTFSYFTTLRVLLSK<br>VEMD
LmigOR16	MIFKYIQEPVLGSLFRSRDSLIYLNRSIDQMGWRLPPRTKPYWWLYYIWTLVVIVLVFIFIPYGLIMTGIKEFKN<br>FTTTDLFTYVQVPVNTNASIMKGIIVLFMRRRFSRAQKMMDAMDIRCTKMEEKVQVHRAAALCNRVVVIY<br>HCIYFGYLSMALTGALVIGKTPFCLYNPLVNPDDHFYLATAIESVTMAGIILANLILDVYPIIYVVVLRIHMELL<br>SERIKTLRTDVEKGDDQHYAELVECVKDHKLIVEYGNTLRPMISATMFIQLLSVGLLLGLAAVSMQFYNTVM<br>ERVVSGVYTIAILSQTFPFCYVCEQLSSDCESLTNTLFHSKWIGAERRYRTTMLYFIHNVQQSILFTAGGIFPICL

	NTNIKMAKFAFSVVTIVNEMDLAEKLRRE
LmigOR17	MIFVDDAVIGIKDPREYRHLRVLRTSLRLLGAWPGHYLGEETGSKYECAPMFLLMFIKIACLYLTIVYLRNNADVLGFFELGHVYLTIFMTFVTLSRGFSLTWNPNYHKVVKKFITEMHLLYFKDNSEYAMKTHRRVHKISHFYTVFLKVQMIAGLTLFNVIPMYNNYRQGNYASDRPANITYDLSIYYETFDILNTPNGYIFICVFNWFASYICCSFFCSFDLILSLMISTVSGHFRILIHNLLTFPLPEAITASKKFVDKHRCNGNRSEFVLEEAKLYSPAEMWQVTDRLRQCIDYHRKLVEFTGDISEAFGPMLFVYYLFHQVSGCLLLLECSQLNTAALVRYGVLTVVLYQQLIQLSVIVESVGTVTGRLKDAVYEVPWEYMDTSNRKTVAIFLMNVQEPLHVNALGLAKVGVQSMAAILKTSFSYFTFLRTVSE
LmigOR18	MIGLTNGDYSPRPSMEGDCLKILKFFAVDIFNPKIVRFFLWIMLLYHVVFTLVTAYFMLYVLSNSEIIGYTPAFLGNFYPMLCVWSVLFISRLIYVKEDMPLWAIDTAGAKVQASIKRKIFLYTAFGIFNLVLSLSAGSFYLKNVSEDVNVFLALRIFRDYFPNYYQVLDLIYRLIYFCFSYLMVAPSYLLIYYILHVRIQAIIFAAYVAHIDGHSDYGTDIDLFDNEEFQSEVERRFKFCIKRQIEFLLMESKKLSQISNLIAAFSLAGCLFGISIIFHLFTGQLIQEYYFRIGLTSLAAIATFSAFIYTGQSTEVQIELVDNAIDNLCWYNFNRSNKLLYLIAKADLARVRKIKFSGQWAVNYDLGFAIVKGIYSIISVVVSMW
LmigOR19	MIIQSVIKRNDLMVLSETLYFLTTHLTFVCKLANLEYHKKLLLDIEDMLKTTRFQKTLSLDLIEKTGMNEKIRKFNLVAKTFRIVCVWCVVLYVLVPYFDPGKSKTLPTPGWFPFNWTDKYYYGTYFFEVAGISITAHMDSSIDILSWLLVTIASFQCDILKENLKNIYYNYDKEHDIRETFKDCIRHHEEIIKFTTKVEQSFSQGILLQFLCSALVICFTGFLMLVVPVLTFQFANTIMYFCCMMIQLGMYCWYGHEIMTTSDEIGQYFYLANWYDSSLTLRKDFAIFLERAKRPITLTAGGFVVLSLNTFTRILRSSYSYFAVLKHLYNKS
LmigOR2	MIISVTGFVTKPFLFEERGFPVDVWLPTSLKDRLDVYWGFYIYVSIGVAYPVIASGVLDPLIPSLLCLATGHLKVLNDNLEHLDEYSSEENGSKDSNLYKNIQKCIKHHIEILNFVYNHQKCFSLMVFSQFLGSPMILCFTCWNVSMREPFSLEWFQSLAYFLGLLLQLFFYCYYGTRLSEEFEHVTTAVYMGKWYKYDVKSRKALIILMERSKKPTIVTAGKILDLSLETFTIILKRSYSLLAVLKNQN
LmigOR20	MIKRPIETHRKYLDKCIHCIRFCGIWKFDSSASTYHKLMNLFGKIFNPTLLIFHFLTLFADIVANYNDLIIIADDGCFLAGSFTVCFKAYEFHLLNNIYMKIINDVHDSVDVLQKSCDLGVLTIIKQYIFFETLDFFLLIYVVTVLGVGLIVLLPLTRGGLPVRAIFPFDVTKPLMHKIAFFIQAYNISFGLVTIVALEYLSWGLMRWTIVQLKVLSSNYRNCNSDKVPIASFNVTKNTYNKIKNFNILKVDDEDIEIHNFIVFEEKELNSINDCFNWRFRTCIRHHQRLIKIIYDLNDIFTVSLLIQLGVSTFLMCLNGYLAFMFPHDNQRLIRSVLYLVAGFVQLLYWCGFGNELKFQANDLTTSQWMSGWEDKFDGGIKNLVTTSMIRTMQPLEVRAGGLFILSMETFLSILKTSYSVFVLLTTVSDEE
LmigOR21	MILFCSTLAKMSNVTFDEPFMFFKKVFFDFGYNKIIKFYNLFCFTLHLCCIITEHYFFLTKYLSADFVTLYGCATILIAYIIVCQYFVMRFEKPIKQLFEERETIFWKIDSDQKAKTQIIKFAAKITRIYKFFFVWVVVLVIVMLPFWGEIDKSFLIIRVQETLFGKWSIIFYCIYVSTFPFLIYSSVRLPMITFYLILQAHLQILILNQKIVQIPQNDNLDLDAGLQKKIYTSLKLCMSRHVSLKRWMSEILQIVRRAIPIFFCLSIICSVTVIFFILNYLENSNTSNLLKIRLVLVGICVAVVLYTYSEAGQLLSDDTSQVFDTLATCSWYEWDTKNRKMLLMFLLHSLKPIKFYWGGFALDYRFGGSVVRTTFSYALVLYNLRKSS
LmigOR22	MIPFVFKRDDSVDHEVVKGYQSTYNYIMRFCGLYPDFRGFWYYISGAHLNTVHLAYIWFLAAYMISTYYAFAYRDMELLSYELCYGLVTLIWFTVTHYTIYKRDQLDSLFRKVGRGFFTYEKPIDSEEEAIIDECNTNCRKTFQKTLALTTILAFWTCIIPPLPKAVMGDYSSIVEGGVPVNKHLALPTWNPYPTDTHLTYWTMWMYQALAGCTEAYIIGATCILYCNFCTIINRELKLLRFSLGNIKNRAIHAFKMRGYSLQLGQKYENSQLYQVCLVHCIDESIKHHIELKQFHGAIQNLLGFPIFAIFSGSALTISSPMFMFLQMIGEHEESSFTLVMNIFQYTIIIFGFTYFLANYCLFGQSITDESALLHFAFYDTPWPEAGLNFRRKVLMGMIHSRKPFVLTAHGLASASSETLVDMLKTVYSYFNLLAAT
LmigOR23	MIQASKYPNSKTKELFRKIAHIAYICGLPNFWIEELNLPKSFIRVYDKIVRIFNVATYFFLGIEIAAHFTQHHLTNKQKFDLLLYSISHPILNGYGVIVSRQVGNVKKVLLDLIVNLKVKYNDPVIEEAMIKISMTYSVSFITNCVLSM

	LTYTFDALLMVYKKGVTFNVIITAWPDVEDTTTEASIGRIGFHIFWWLFVTRPFAVYVLVINLTTCLSHQYMN<br>LQSYFFHLEDIFKENLSQNEKEAKYEAEYKIGVMLHANTLRCTRRCHMVWNGVMSGQIIFNISLIVIIMAQM<br>MNSDRTLVNTFGTVLTASAILISTGFFMWNAGDVTVQASRLATAMYCSGWQNCRGKSSVSIRNMVMNTIA<br>VAQRPLVLRGLGVIDLSYQSYLSIVKASYTVFSVIY
LmigOR24	MIRLRLPPTINQDTSQKNIKTIYEELDSQFHTGIRFLLMGASPEKFLGKLSLTYVLIYLSIILFFMLYALFEIIVPFF<br>FPGDFLEFMNHIYFGSYCLAFGYQWIYLLMNLNNIYLNRLNIESLYSTQAVSGIAEAALRKNLRPFKIAIKCSI<br>ALWSITILAFGLGSLIEIAVEYLLTGEVESYAIMSTFPFPPWGQMLVTALNFVTLNMGFAEVVAMAYISSLLALE<br>IETQCEILCAAMLQDRDDWFKFRGYISDHARIIKNAKWLIGILESLNAPTVFSAYILMAIEMVVLTLVEPDGLF<br>FVAMASDLMGVVIILIFQGWISSKITLSLQSISFAAYQTSWHEQDKNKALDLMIVTQMAQRTYVQKILLGTVV<br>IERGTVLQIARSAYSFYTLLMVLQSNKTL
LmigOR25	MIRNWQEEEEGTTLLERMGQKFLNGHSIYLGGWVLRFPVRLPPFFYLTCAFGLIVKMILNYDNLTLIIDCAH<br>MMIHMIVGLQTTLLALKQKGNIMRLKTQLDNFGYVKDLETAGEKIKEECEQEAMELYKPFSRCILITINVYIL<br>FPICKLFTEAGRAKLSRVLVWQMWMWVPDDTWWGFTIIFLFELVTSLFLLLSVMYTVPYLACLGKMTVGHC<br>KLLALQLESISKKATQAAFTSGSFKAALNHEIDECARRLHETHMLANEVAEVYKYYLSSFYYGGMFALCMS<br>GLQAVSATENIEESLKFMGVLTGELIAIGLATYVSEGMIQAFADVRSSI
LmigOR26	MISFEEKHLTNVRESDVLWLGRIMSLEIVQYKPMRFILNIIAVSIIALTLIQTYLFLQKFDGLYLIKYASVYTASLF<br>ILFSIIAAPFLTKFSTEALNNLEYWPIESAGAQIEKQIQREAIYINTFFVVNMVVSLISGVAHMIPLDDDKELFYP<br>LAIFEEFAPKWKNWLEWGYRLSFLVVPVVMLNSSYVGIYTLSNFRFQISLFNHLLKNINFPLNDNEQTIELMD<br>DQKYQNEINKRLKFCIKRQTHLYKVAHYVTGKVKHLSFFVAILTILLLIAVIVFLFSFQGTFENRYFRIITLVLTA<br>GNTFIHVIIMGNRIEEETEKIFENLKSLNWSSWNLQNRQVYLIFLHNNEEHFKVPISENASVNYELGISMAKTI<br>CSMVSVMSQLKNIDYSKN
LmigOR27	MISFEENIDHDIYKDDVLWIMRKISIDYFHYKVVKILLTLLSIGITILTVIQTFLFLERFEGRYFVKYAPAYIATFL<br>MVVAMQYISFSIRLAALIKRITFWTINSARVETERKIKKHAMYTNIFFLGTVIMGVISALFHIMPLDDDNELFF<br>PLILFEEFVPNWKNFFSWMYRLNFLAVPFTLPIPIYITTYHLIKSYYQILLYLDFLKNINTGFDTTSSNNIESAEY<br>QQVTRDRLVFCIKRHSYFYTQMREVNRKMSKFIAIFALISVLLGGSVLTFLFSFQGTFENRYPRIVTLILTAGCIF<br>AHVIYAGQLIEEAATQVCENLKVLDWYHWNCHNRKLYLIFLQNTQKPYKTQFSQNVSINYELGLSIIKTVYS<br>LISVLRNLQDIN
LmigOR28	MISFHKKAALNLDQEDKLWFLRKIYIDIFTSKISKILLSLLLINGIFLMLVQAVLFLEKFETTYLLKYVSSYSAAF<br>FVLACLYALPLVAKTIKLKFIQQFQLWPIDSASDEIKNQIKKTAFFVNFYTVVSLAVGVMCATVFAIPTDDDTD<br>FIFPIALMEEFIPEWKNFLLWCYRLAVFCFIVILTGPFFIVVYVIHNIRFQVLMFLHYLNNLNSGYTQRTINDKK<br>YQNVIKKRLQFCIKRHVHITSIINNNRKELYDFIVIFAVTGVLVLISVLIFVFSFQGSFESQNIRVAVFTLACMLTF<br>LHVIAVGQWIENVTSEVFEILKTIDWTCWNLANQKTYLIFMQNTKNHVKVQFSENISLNYELGVAMLKSVA<br>SMISVLHQLKNIDYSKSN
LmigOR29	MISFNEKLLLDSNQKDELWFLRKVYIDIYTTKIAKILLRLLFINCIFLMSVQAVLFLQKFEASYLLKYVAGYSGS<br>FFVLACFYAVPIMVKTMKVKFIAGIKKWRVDSASEKIKNEIKTTAFIINIYTGVTVVLGVFCSLLFSIPTEDDTD<br>FIFSIALMDRFVPEWKSVLMWPYRFSLFPLAVILTTPCFVVIYVVHHARFQMLLLLHYLKNVNSGHMTQSIG<br>NARFQKEIRKRLKFCIKRHNHIITSLNQCRIDLYNLIIVFAVTGGLMLISILIFLFSFQGSFKSQYVRVTAFTIEYIL<br>TFSHVIVVGQMSENVTFEFFQILKTVDWNRWNLSNQKTYLVFLENTQNHFKIQFSENIALNYELGVSILKTV<br>ASMLSVLNQIKSIDYSKEKM
LmigOR3	MISLTIGVRKQLQSTTNIMDNMFSFFAATPSFLGMGKIICLLRQRRALRRIWKSLDDLLENVLKRDVDEQLEK<br>ELRWRLKRCWAMYSIFLTVGTCITLHWLLRPIVYALYGERTSIVSTWPTYLESWIQWFTTYIFQAMNISSIGHA<br>LYIYDNVYFCICENLLIHFAVIKHHLHQMDISKGKPGGVTMKFCISHHVKLMDICMELRECSKYVIMQQVF<br>WTIFILCPGVFELVSGRQTDTTILFNLMEITTIMTCILFFYSWYSNEVTLQSSQVFNTCYMSNWVEGTPNQRRT<br>MMTMMTRSMKPMIFGGLVNVDLGTFISCFFRC

LmigOR30	MIVYEEKIDQDVNGNDILWVMRKVCIDCFQYKIVKILLLLLAIFIAILTLVQGFRFLERFDGPYFIKYAPAFIRTF<br>VILVAIAFISFGMETAIYVKDTTFWLIDSAGLESEKRIKKHAMYTNIFFVSYIVVGVISAIFHIIPLDDDNDVLYP<br>LALFEEYVPDWKNLFSSIYRFTFLTVPFTLGIPLYTAIYIITTVYYQILLFVVYVKNINTDLDVENVKYQETIEKR<br>LIFCIQRHSSFLKRMKESNRKMSATVLVFSIVGILLGASVLMFLFSFHMSFKIWYYRIITMVLPTGAIFIHIIFIGQ<br>SLENAISQLEANLKMVEWYHWNIPNRKLYLIFLINTQEQKGVKFSQNVSVNYKLGVSIAKAVYSLISLMSNL<br>RSID
LmigOR31	MIYYRKCKMELNFDKIFKIAIISQKFSGTYPYTKRDKKWATHFILMHGELTIICMLFIYNIIEFDLKAADYSQM<br>CRNMCLSFVYMVITLLYINMLYYQSKLKMLIETMKAEYELAKTMSEEEQNVILEYAKKGRWLCRAWAILTTC<br>GMAQFFLKSIVCTIYSAIQGNFRIVQYYEVICPEVIERHRNNPVIFITLYFCTFFYSLYTSALYTSVLPLGPIFLLH<br>GCAKLEIVRLNIKNLFDNDDYVVQERLKKTVLQMQDIYCYSHEINECFQILYEFLLKATSLVLPITIFAVIQAL<br>GRGQFIPEFFAFIFGAFMVGTTPCYYSNMLMEKSEDVRMTLYSCGWETRFDLNTRKCIILMLCRALRPVSIRTI<br>FRSVSLTTLTDVFQQAYALFNLLNAVWN
LmigOR32	MIYYRKSKMELNFDKIFRIAIISQKFSGTYPYTKRDKKWATHFILMHGELTIICMLFIYNIIEFDLKAADYSQMC<br>RNMCLSFVYLVITLLYINMLYYQSKLKMLIETMKAEYEIAKTMSEEEQNVILEYAKKGRWLCRAWAILTTCG<br>MAQFFLKSIIVCTIYSAIQGNFRIVQYYEVIYPEVIERHRNNPVIFITMYFCTFFYSLYTSALYTSVLPLGPIFLLH<br>GCAKLEIVRLNIKNLFDNDDYVVQERLKKTVLQMQEIYCYSNEINECFQVIYEFLLKSSSLVLPITIFAVIQALG<br>RGQFIPEFFAFIFGAFVVGTTPCYYSNMLMEKSEDVCMTLYSCGWETRFDLNTRKCIILMLCRALRPVSIRTIF<br>RSVSLTTLTGVFQQAYALFNLLNAVWN
LmigOR33	MKAQDSDNPYIVLRRVFVDFAFTSHMIIYTKITFVFHFLTLLLETYYMITNFNVELFSRYGCMMCLMTYIVLA<br>KLLEILFARHIKFLEEERLSHFWKLEESSEETQKVVNAESSKIRKKTFFVLSWFVALGFVLFPIFGDLNDLFMFG<br>RVYRNYFGSWAIIPFCIYVSTFPSIAYNSICLPAVVSYFIFHLNLQISLINDKLGKISEKSRQSEIYQKLCSCVAHH<br>VRLRRWTNIFQNELESALPFYLFLGAINSIAVSFFILYNLQNMTLIFEIRLVVISVCNVLILWIFAEAGQEFSDNS<br>DSIFDAVVACPWYSWNAQNRKIMLIFMLNCLKPMTFSWGGVKLDYQFTVTIVKMSYSYALVLYNWRYEK
LmigOR34	MKDKPDWDPTEEIAIIIAIYGTYYIVLAYVKNQRKAAGILRDLSNFDKFGVPPGFEEEEQRLRVYIICVFIYGFI<br>TITFYNFYKMSQKKSCERFNIEHNLHENCGLIDKFPRYELVFLYLLTCCHLLMKLPLIVSYNALEMVHHIILRI<br>NHLKIMITECFDDPDYEISRRKLTQCILYHTEILEFATRVDDCFSNCMFAHLTLTGTICACLEKQIVAGFSRFGA<br>ILHFFGWILALFIACLGGQQFINASDTIPEALWASKWYNADLRLRGDLLLMMMRSQRDLHITAGPFGVVSY<br>ALFVSVLKASYSILCVLTS
LmigOR35	MKEEYYLQHPRTQLFYKVLAHVSTIESTIDLTWWGYTFPKYVGWFYHLQCNVVRLFGKCVVVSQILFIILNY<br>QTIDKSVFIIAITITPLGALVGIKAESAKAECYVNLMKNFMDKVHIHSIYRKNENNEFVKKKVIQIERVSRFTA<br>YFLVILIAINCLSWMLKPTLHNIKHFEEIMNKSMEFQYYIYFWTPLDYKYNLRDYIIIHTLCIYLGATAVTVIVT<br>FDIFNFIAVFHVVAHIQILKNNVKSNWSDDFNESEKKGYLVSILEYHAYIIRIFGEVQSAFGLNVASNYLQNLI<br>EDGLFLYQIMNGEKENVLMYGLMIILYLGGLIFLSIVLEEIRRQNYDLCEYVVYALPWEGMSLENQKIFVVFLQ<br>RTQPDLEFETVCGMKAGVKPAFSIVKSMFSYYVMINSRF
LmigOR36	MKEIRNLTISNANNSMKVRHAYRLGINSEGDRSDDGLGRDRNQSFDNRAFIISVQPSESSVGAFLRKRFIVKY<br>LPKVLSTVHRLARSITLAMLVFGAASSIGWILNSLLLGGNSMLPMVAWYGIDQASSPTYEMLYVSQSLVIFYC<br>FLTSWGLDLLLASVMIHIAALLKTVCIHFSLVTAVSSNRTPGMQMLQRNKTLPTAGNNLETVVLEMTSTDYK<br>YLQCVKCIQDHQRVIAMVKDLEDLANPVILTQFVAGIVVICVNLYHTTTDTHGFLWASKFASYLLMLVFQIFI<br>YCWCAHNIMEQNLLLAEWTWENGWRLWRAAVDLSPGAGGGGRVNGYACTLLFVSVAGIGLFSSSLSSFRK<br>LINTSYSFYAVLRQLNSR
LmigOR37	MKEKDHSKRKDMLSVDYRKYMYCKLIWIDDGLADRGLTTPLLFIMIWMVFMVFFGLVSFVMSTQNKVRLD<br>NLRSFLLESMVTMSIFNEYMSRKSLARLHHFMDESMKTPRTGLPKEEEILKDAKSLAKKHLTAYTVIFTFNLA<br>AMILSQPVAEWIQGNSWKKLPYPWVIPPSHNEFLFWIVFMFQSIGLYFSHCLGVVIMSFSSITIQITALFEVLLL<br>SLSHIEDRAKVMSEREGADYTTCITECLKEDIIHHQRLARELISATPHLRRTFFALSVTISMIMACEAYPLIMGN

	FTLGELIKGLLFLVVQFMCWGQMCTRMETMADQNTAVFNALYGTPWYDSGVKYKKLMLTSFTFSREPMYIISPLFIEMKATMSTFYSFVVSSFNILNLIRKMN
LmigOR38	MKFAEPQIQEEDYLKLIKICFIDVFETNLAKLALIVAFVIATGLTILQSYYSINNFTANQFLKYAPVYFGRFYVLVCLVFIFYYTKVIFKIFDTVPKWKYETVSKKYNQHVRRDALYMNYFCLFSVLAGLCTAVLYVIPSEEDHEIFFIVSWFEDNVLEWADILSLCLRLSFLFVSYLMQAPCFQMCYLLRHIQYQMGILKFYMRNIHHGFENLDKLIFDDSFHKEIKRRLEFCIKRHVTIVLIGTTMVREGRIFILLFAISGAMISVSLMMFLFLVERSLPFTYLRLSALIFTATLTGIQYIAQGQSIQNTSEQWFRTLVRIKWYYWNRENQKSYFIILNAYKPFSIKFSQKLAVNYKLGITIAKAVYSIMSFMRYITDHVGN
LmigOR39	MKFFEQSYFTLPRNFARSIGRWPYQSSLQSFLIGIVIISAFILQVGPKILADIVHSDDQELILETLAPTITNVMAFAKYINTFVNARMLKILFERIKDDWESVTDKKEKIILESYAGFGKLMATGYAGFVYAATVQFITEPVLPIILNNILRTNLSAPHKFADPMEWIIIDKEKYYWILLSNSSVCIMVILTVLISYDVIFITFVYHACGLFAITGHRIENLPHDENFKIINRNTNSLKNSRDVHYKHLVSCIRIHRMALKYVDLIESTFAGCFGVVVGLNLPLMSITGVQGMKFFRLLHNRMTLQQKIKYVMFTGAQMLHLFFECFLSQQLTDMSLRVQQHIANGNWYDISTKSQKLLILMTMRSQVPCILTAGKIMELSVESFGMMMKTSGSYFTVLLSMQ
LmigOR4	MKFFNHPDWRIGKLMLCSFGGWPYQSQHSRRILNFISIFTVQSIFIPEIFRLTRIWNSMEMIVECVPMISLHIVANIKMFNCIINLNKVKVLLEDIERYYQSDLCKSELRILYKDRHSHKKVISVYVFYIYSIAVVFASIPVLSKVLDLIAPLNESRPKVYLYPAEYFVDQDKYSTYIYIHGYMALPITMTLCTAYDFLYSACGHHVCSMFKIAGSRLKNFIDNKIAWSKNESLDRNYRQDEVYKSLVECVKMHKFILNYVDCYQETFSDSLFLVIGVNMLALCITSLQSLITMNQFHDAVSYIVFAFGQLTHLFLLNYQGQNIINHSENFYNDAYQTNWHEFSRKSRKLYILIIMRSSEFSVIRAGKIYVMSSDSFSNVLKTAMSYLMVLNSLR
LmigOR40	MKFIDKLAEEEDDELIEILKGNYWHFLFYSMTFIRWKRPRIAIALISAYAIWIIVHLVIGIYSIYLAADERNWAVVGLVTHHMVLGALAIYLPIFCNTGGFREVMADMHRTFTTDIGQYSGGNMYAEQACIDIKKDVRRQTFVYYINPALVAAAGSLALAGPFLTKWFSGMENPYSPNGLSLKLPTALYYPFPTDSGVVFYAIVLTQVISGTILGYLILAPQLVFINLSQNLKRELRFVGYSMETLVRRAMRMTFENNVWKRKVTELDVDDTEFQQNVELSIKETIIHHQKASKLLSTAQVSVKGPLAASYIFGLVTIAISLYNITLALKTNDIGSLTTFLLLLSSEVIGTFINCGLIGSELTEQSEDVTEKLYFIEWYNFSVKNRKMFFTFQTAITQPYEIKAGGVTPMNMETFSDIMNSAYSFFNILQTIE
LmigOR41	MKFMKYAVFFYTSVGIEPYTIDSRSKKASLWSHLLFWANVINLSVIVFGEILYLGVAYSDGKFIDAVTVLSYIGFVIVGMSKMFFIWWKKTDLSDLVKELEHIYPNGKAEEEMYRLDRYLRSCSRISITYALLYSVLIWTFNLFSIMQFLVYEKLLKIRVVGQTLPYLMYFPWNWHENWTYYVLLFCQNFAGHTSASGQISTDLLLCAVATQVVMHFDYLARVVEKQVLDRDWSENSRFLAKTVQYHQRILRLMDVLNDIFGIPLLLNFMVSTFVICFVGFQMTVGVPPDIMIKLFLFLFSSLSQVYLICHYGQLIADASSLSISAYKQNWQNADIRYRRALVFFIARPQRTTYLKATIFMNITRATMTDLLQVSYKFFALLRTMYIK
LmigOR42	MKHVIMDELLIFLTFLGLWPRTPTSPKIISYLMIYSTSFLFFGSSIYLILHRKFGSDEIDTIEIITSQFGVLYYLTLLVVKRDGITKIVNLLSDFSKFGKPPLFDQRSRRLNLLLRLFVTVLLAATVAIVSVPVVFINSCNKQNLQLNATKICGLAAPVWLPFDYTQNPRKYFVSAMEIYCATMNYAGSGSGAFLVIGTMEHLVIRIEHLKNMFPEILNEPDKQIREKRLKKWIEYHLSIFEIGELMNETYKWPLSVIVLCVGILFGCIGVSTMQSVSFQNSSVFLFFGWFQSIFVLCFWGQRLLDSCLSIRKAVYNSKWHEMDVSFQKSVLMILIRSERPVLIHAGPFSYLSNLLVLGVLQTAYSYINLLNARS
LmigOR43	MKIVTSDSNVQPVGSTSESKMVERKPQNLTKQLSLSRNPVRTRKERQKRTKTIYDQLDTLFHVGVRCLLLGISPDKFRGNKGYERFLIYFVVILFFFAYSLFQLCAPYFFPGDFLQGMRHFYFGSYCLTFGYQWIYLLRNFDKINQNRVNLKCFNSSRAVTEIAEVILEGKLKPFTRRIVCIAMIFSIASFSHGLGELIQFGEQYFIMGEVKTMLTYFPFPIWGQLIIVLLNTITISLLLAGTVSALIITGLLTLEIETQCAVLCAAMVRGTDDGSQSFTGLIADHVQIIKNARWLIDLMESLNSPAFSCFYFHIAIGLVIMKLVETNGYLFYVSMAFGFMSVILQTGAQGWFSSAITHSLESIAFAAYETNWYERDKNYARDVLMVIQMGQLRFVQKIFFRSIEIDRATVLRIVRSSYSFYTILMIIQ

LmigOR44	MKKASAQYSQESLSARRGLDVSTCERILHWFSCWRGGSAWYVVYVVVARASVAGVVVSQLGLIPHVWGDL<br>YSTSLCVYLTLAVLSAWFKMVQFDLARPKVDALLQELRGELLLHAEDLSEGTEDVFRLFQKRSGAFRRVFLV<br>LDHLTILSWAAKPFLDRLLGSDGPLSLPQDAWYPFDTTRSPYYEIAYLHQVVSLYVAATCVVTVDVFFTTLFM<br>YVTAQLRVLNLHLRAMDCRPQLTQSEKITRCDKGVVAEKSGDANRLPSADPPDAYAQLVHNVQHHQVIIR<br>TVKILEDAMGPCVFVQFLFNVIVICIYAFVLAGVHSMNIGSLILCLTNLISTCVENFGYCWFGNEIMSQSEQLA<br>FSAYSSGWVGGGGRFQKALVVLTMRAQRPLCLTVGSLYTLSRETFLALLNGSYSFLAVLLQMNNSN
LmigOR45	MKKEKERRVQLEDLGSGEDLSRAEVESSVLRQNILLLHYMGLWPMGGSRAYRCFTAFNLSSSATIIVMNVVG<br>VCFSLSDIDQVTGALSTILPMSGGLLNGLFLLHQRPTLCRMVRTVDRLVTSQAQFVERDARLVAIVGGARRR<br>TLVVTLGVSGYLFAIASYWVVIAFTLPPSLRVLPFVQLPWMPSSDPGLFWSTFGAQLYTAPFCSYTTLFVEFFFL<br>AVMLHLSAQFRVLGSRFASLGRSSASKVAADSGAVYEELRLCVETHQELLRFVRFVDNVMSPFAMLQFVAG<br>TLAVCVVLFQAANNQDLNTNLKCAGWLPGPSLELYIYCGGAHEVVHAGEALVQAAYDCLWYNVAPRIGR<br>AIRLVITRAQVPPVLTAGHLYPITRPTFVSLVNAAYSYYALLSQMQNK
LmigOR46	MKKSGMEDLGRTVSWSESGRSVLRLNIRHLWLFGVWPLWDSPLFILYTGYGLSLGVWNVVEGALAASCTW<br>GDMEQTTLALMATSTNCNGLVKMAFFLRDRRRYQALVRRVAALVALQGESCAADPLLGRVQRGSRRRAFR<br>LTAAMLLFMFSQCFVWFPMPAVAHADEGPLPFAPDNTTAYGLSYLAQCAAGLWMTQVSFGMDCLFASVM<br>VLLAAQLQIVAGRIARLGEQNYDFAFSFVGHLQDTMSPIAMTQFAFSVLVVCFGLFQSTYAEAVTMAAYSCS<br>WVEASGRFKRALRILISRAQKPLILSAGHLYPIDREAFLSVRS
LmigOR47	MKLLEKLEDPDRPLLGPNVKALKFWGLLLPESRSKKYFYLFMHFAVTVFTATEYIDVWFVKSDLALLLNNLK<br>ITMLATVSVLKVTTFLLWQNAWRDLIGYVSRADLEQRATSDSRKLALINGFTGYCRKITYYYWFLMYTTVAI<br>VTVQPIFKFFSSAAYRLDVQSGNGTYLQVVSSWIPWDKNTLPGYLLASIYQTYAAIYGGGWITSFDTNAIVIM<br>VFFRAELELLRIDCAALFDDEKSFGDMAFMRRLKECHRRHTELVKHSRLFDSCLSPIMLLYMFVCSVMLCVT<br>AYQITIETNPMERFLMTEYLVFGVAQLFMYCWHSNDVLYASQDLSRGPYESAWWSRDVKYRKNLYILVAQF<br>NKVIVFSAGPFTKLTVATFIRILKGAYSYYTLLSQSQMNKT
LmigOR48	MKLPAHTKGYKVTPEGAVNFIRVTVYLTCISFPLTEKTKVRINYEIILWLSIFLSISLFAPLLASIIKYSDDTFIVM<br>KSFILMSAISNYVIKVIIVRIYHKELQQLGSALDEFIKKANESEKVILQRYVDNTWKFHGFMTCSYYLTATAVL<br>LGPLILPQKFPTDAVYPFPVDNQIISYIVYLHQCIVGYQCSAGMALDCQAALFLWYLSARFEILISEAKNVETF<br>DELRNYIKKHQIILLYAKELIRPTRLIAFVTVVMTKIGMIFGGIVLISDEPVVIKIQFAILVISTTINIYVCAWAAD<br>NLITVSSTAMSNAIFEISWMHAPKLRNFLQTVIHRTQKPVVIKIPGLLETLSNEYYAQFLSAAFSCFAAARVVV<br>SS
LmigOR49	MKLSETLKIDYFRVQLNAWRICGALDLSEGRYWSWSMLLCILVYLPTPMLLRGVYSFEDPVENNFSLSLTVTS<br>LSNLMKFCMYVAQLTKMVEVQSLIGQLDARVSGESQSERHRNMTEHLLRMSKLFQITYAVVFIIAAVPFVFE<br>TELSLPMPMWFPFDWKNSMVAYIGALVFQEIGYVFQIMQCFAADSFPPLVLYLISEQCQLLILRISEIGYGYKTL<br>EENEQDLVNCIRDQNALYRLLDVTKSLVSYPMMVQFMVIGINIAITLFVLIFYVETLYDRIYYLCFLLGITVQTY<br>PLCYYGTMVQESFAELHYAVFCSNWVDQSASYRGHMLILAERTKRMQLLLAGNLVPIHLSTYVACWKGAYS<br>FFTLMADRDGLGS
LmigOR5	MKLVFDNFIFALKVTLNWCRYFGIFIPDELTGRRQKLLVQAYSVFMFMLFIGFFIITQIILFILVWGDLSLMTDV<br>GLVLGTNLALSAKIAVFFFKREELASILKKNDDTLRFETREEGKKIISEIDRETNAFMKVFFCFGVGTVIAWFLS<br>TPKGELHIATWYPCDTKRSPAYEIIMIHQLAITADLLMLSMIAVCRCRVKLVGLYLQTICDDLPCNVKNKLTS<br>DEEVIVAKRIREYVIEHQAILDCISELQNHFSPALLVQLLTSVVIICVTAYQLAVEKSSDLLRKFTMASFLFAMST<br>EMFTFGYQGGHLSHDSMEVATAAYSCPWYTFPTSLKRSLLVIMIRAQQPALLTAGGFTTLSLETFVTIMKASYS<br>FFTVLQEATD
LmigOR50	MKLVFDNFIFALKVTLNWCRYFGIFIPDELTGRRQKLLVQAYSVFMFMLFIGFFIITQIILFILVWGDLSLMTDV<br>GLVLGTNLALSAKIAVFFFKREELASILKKNDDTLRFETREEGKKIISEYPCDTKRSPAYEIIMIHQTIAVAVIASL<br>AITADLLMLSMIAVCRCRVKLVGLYLQTICDDLPCNVKNKLTSDEEVIVAKRIREYVIEHQAVLDCISELQNH

	FSPALLVQLLTSVVIICVTAYQLAVEKSSDMLRKFTMASFLFGMSTEMFMFGYQGGHLSHDSMEVATAAYSCP<br>WYTFPTSLKRSLLVIMIRAQQPALLTAGGFTTLSLETFVTVS
LmigOR51	MKLWIRNANFTISLSLTLLRCLGFWSPDGLAGNKRLLYNCYSFVFFMFLLGIYILIQVVDMIKIWGDLPLMTG<br>TAFLLFTNFAHATKVINIVIRKNRIQRVIQQANAVLMGVQSEEARRIVKSCDFETSIQLCLYFLLTFVTTVGWA<br>TSAEKHQLPLRAWYPYDTSKSPAYELTYIHQVAALLIAAYINVAKDSLVSSLIAQCRCRLRLVGLALASLGQDL<br>KIDYQSQLSPAQENILNLRLKTCVLEHQTVLAAVTELQACFSKPTFAQFTVSLIIICVTAFQLVSQTGNLVRLLS<br>MGTYLMNMIFQVFIYCYQGNKLSVESSEIAGSVYFSPWYLGSVKLRRALLIVMVRSRRVAKLTAGGFTTLSLA<br>SFMAIIKASYSLFTLLQQVKQKK
LmigOR52	MKLWQSIREFGLEYCDLPTTLQNVASLLRAITLNIDSRHTARIPFICYVMTVVITLSYFYVFLVSMAWFVFVRS<br>AETRDYLAAMVVLSLGISSEIGTLKFFYTFIYIKKVQRIVREYLECDHMVVPESRFADNVLKTMRNVKKRAIL<br>YWVVVIGNGVVYVTKPLFMSGRHHMEDRYIVYGLEPMFESPNYEVAYFLMMFGLCFICYPPANVTVFLIVV<br>VGYTEAQMIALGEEMLRIWEDAVAHYNNKYHTVGALTNSSEKNKIINQYVKFRLTEIIKMHTTNIQLLRQV<br>EFVFRSAIAMGYVFLVLGLIAELLGGLENTYLQIPFALIQVLVDCYTGQKVMDASSLFEQAVYDCKWENFDK<br>SNMKTVLLILQNSQKSMRLSVGGITVLGFSCMMSVMKSIYSAYATLRTTMS
LmigOR53	MKNQEIKICRATLTVLKYSLIWPSEADEMNPGKWYYIRVVTFILFTCPWVLSVFMHLIVSIRNNADIHLSEDV<br>ALMVAFTGVYYMTIIYVKKQPKVAFLLRDLSYFQFGKPPGFDETERILGFLSKLTFCYSVMAVVIYNYIKYRQK<br>PECERMNKLKGLKENCGMLTPTWWPFEINYSPAFQLIFLYIFTSTQVMMKLSLMISFNVLEMAHHIILRINHL<br>KTMILESLDEQDYEASKRKIKTCILYHLEILGFAERMDDCFSNGMFAHLTITAAICGCLEKQFVDGDNQLGSL<br>LHIFGWILALFLACLGGQHLINASETISDAIWSSKWYDADLRLRKDLIFMMARSQVGLYLNVGGFGILSYALF<br>LSVIKMSYSILAMLTS
LmigOR54	MKNQMHSDEKVWDTTKDVEAMLGPSAVVMSAIGLWQPPGGRPAGAKVLVTSAVLLMMLTIFLGGLVQLV<br>LEPLPLEEILDAVFTCACSFTWGIRVMVIRLRQRRVQQLVVDVLNMRKRFTENAAALRKKYHRRGLMVCLA<br>WVVFPALAVPMWFVEPAMTKKLVTTSENTTMVIRKTPFIMWMPMETQTHPNYEITYVAHLSLLVCSVSPTLI<br>IDLFFACLMTAITADIDILNNNIANMRLYKEDGFTENSEKGVEVTATWETTYQSKKSLAEKSERVDDNTQVL<br>ATYSTDPYEQLHRTLAKNIQHHQTLMSIVGDLESIMSESSVLMLIVNSINICLQALGFVDAFRPGAKRSTVLK<br>KVLTFPAYINQTAHFCWLGQAIIDQSERLQDSAFSCGWADADQRFCSSLRIFMLQASRPLKLQIGKIFTLSRN<br>LFLQILNTSYTIFNMMINF
LmigOR55	MKPALKLANVLGLDPLRNDNYTQLKKMFCALCIVSLFVSAYLEFFSNFTTFETYETAPESLIPHFQTMFKMYS<br>LIFSRTEIVELIQMAEQFYKFSQCDERKKLTKLYKRVDLFFYVYASLVAAACVLFAIVTLIFKPGKPIFLCYGGL<br>HGLESPEFEIYLVVDLIGIVIISVTVPAFDGLFFYFALYIYTEFKLLKIAFKTMSGQELREAVKHHDFLLKYIKKL<br>NSVYSPIFLYQFFCNLLAICFCLFMLSRSGIPPEMVSFSKYFLCLLAFLVQSYTFCSIGDLITELSEDVSNAIFYTD<br>WLDDEAYENKTARLIIMSRAQNPVMLTIGKFANMNLRTFILIVRNAYSFLAFVNHALN
LmigOR56	MKPTEIKKPYRMEEFLRPQMFQEVAQMVHFQWRRNPVDNSMVNASMVPFCLSAFLNVLFFGCNGWDIIG<br>HFWLGHPANQNPPVLSITIYFSIRGLMLYLKRKEIVEFVNDLDRECPRDLVSQLDMQMDETYRNFWQRYRFI<br>RIYSHLGGPMFCVVPLALFLLTHEGKDTPVAQHEQLLGGWLPCGVRKDPNFYLLVWSFDLMCTTCGVSFFV<br>TFDNLFNVMQGHLVMHLGHLARQFSAIDPRQSLTDEKRFFVDLRLLVQRQQLLNGLCRKYNDIFKVAFLVS<br>NFVGAGSLCFYLFMLSETSDVLIIAQYILPTLVLVGFTFEICLRGTQLEKASEGLESSLRSQEWYLGSRRYRKFY<br>LLWTQYCQRTQQLGAFGLIQVNMVHFTEIMQLAYRLFTFLKSH
LmigOR57	MKPVGGGCGGLLGPGLTIRRLMGLWWPQGGRGRIAAAAAATLTVASLTMLVAFPALKLIMDTPSELEEITL<br>CCFVIFLCSGFIIKSALFIYQGDILKELLQLFSDNRRIYSNDRNSEGIRQSYIKLSERVYYVYMQVSVLPAVAGWVS<br>APMLARFFLTAESTQQFPVPLWFPGDIYQTPTYEILYAVQSFCVLVTGQCTVVIDVFFIHLMLMVAAELHVLN<br>ENISLMQKLNVKTRVSEAEEWQFRIRRNDEELTFPIHDHRARVGYFCSEDVSDENMCLKLVKNIQHHQQIL<br>RSVLLLKSVMNVSIFILLLLNMADLCSCMFITAVLLQRGGDVTKALKPLLTIPPLLYETGMYCFFGQILTDQSE<br>NLIDSAFSSGWVDCDSRFKRDLLIFLMAANRPLEVTVGKISKLSKQMLVQVLNGTYGLLNLLYHFHGSQ

LmigOR58	MKQKAVGAKKQLKTDNRDVLVTVKLLANDVFHAKAAKIVLKLLVLIHGSISLIQIYTILFKPNIEEFTLKAPIF FGLIYPISGALTLLLKPELIDNFPNYTKISLTDNIDPKTYQSIKKSAKIAFYYTVATISLAIISGINYIQFLKHEDEIF FAYKFFNDYFPKYSLILKIIYKATLPILGYIAVAIPIQTVYGIEHMKYQIMLVKSWVKKIDSDADNKKDQKMVR SQLVLCIQRHSSLITFIHKIKKEIDSLFLMSLLNAVLCFMSIFLFIFSATFYREYYLRIGLTCITSGFIGLIVVFGQQF ENEVENLATVTCNLNWYFFDLKNKSIYLIFVGQIIRPLRMQYLDFGLNYRLPLSFLKLVYTVLSLMLKVRPTF HEQN
LmigOR59	MKQSASLEDCSPLTWEYTQQSLLKLNIRLLWALGVWPLPGSWVFSLLKVWLAALAVGNAVENVLGVWKN WGDLTEVTYSLLNAFTIGAGVAKTWHLSRYQPRYCLLVRRVDRLTRSQRRYCDGDAAMRAVTLGCRRTAR RVTLSAFAYLTALCLIWMFMPLVAHPGERLLPFNHIPWEPRRFPLFYELSYAVQSASSVVYVFISFALDCFFAVV MIFLTEQLMVLNLRIRQLYARRDGDGSVLVAKQHSDKTVLDKHEEMYKELCLCIDTHQDIIRLLSFLDAVMN PIVLTQFMLSVMAACVTLFLESYSPDSSSVLNSISYLPTPGIQVYLYCWSAHNVLEEGFAVSEAAYGCAWYEG GGRFKRALRIVMCRAQKPLVVTAGRLYPVSRATFVSLVNASYTYYALLSRVHNRG
LmigOR6	MKQTATKEGLGRKGFSVTPETAISFTRITVYLTCIWSPSTSSTKLTFILFEIFLWFSIFLSLGLLFPLIVSIIKFIDDTF VVMKSFILISGIVNFVIKVIVCRIYRQELQELGASLNDFVRNSNENEKFYLQKYVDKCWKFHGYMTCSYYLTT SAVLMGPLVLPEKFPSDAVYPFPVDHPVVATIVYLHQCIVGYQCAAGMALDCQAALFLWYLSARFEILISETV NIASHKEIQDFIKTHQDILKFGKQVIRPIRLIVLTTVTMTKVGMIFGAIVLISDEPITVKVQFAILVVSATVNIYV CTWAADNLLTVSSSTISNEIFHVSAIHPPAMKKLWLTVIHRALKPITIEVPGFLETLSNEFYSNFLSTAFSYFAA MHAVVNS
LmigOR60	MKRELKAEAVRHFTRLVVKKPMDLLPRLHRGRGVFNLEETSSGRSDSESKLPRAQHNVFYQQFRPMILVLTA FGRLAIQRGSDGEYRWKWFSWLSLFCLLNYAVQTYFAVAICRQRIKAVFESSNYDEFIFAIHILAYIQMHFHVP VSYWVQGPLYAQYLNQWSQFQNEWFLVTGEELKFRHKKAALTFVLVMLPFLALVLVMEKYSTLHDPFEYLL PHFFTIGSTVCVLGTWYIACLEIAFISKDLTKHLIKKLHSNPDPTFLQKWRALWMNLGSLVTNLGTNHFAIMS SIFLTFSVTFLLGLYNALSKIIIGDFSLKTIGYLTASGMSIIIIYIVCDSGHQATSRVESYTSQCILRAHLPAARDDV KYEVDLILRVVQTDPPQIQLAGFVTLNRPLFISFVANTITYLIVILQFKG
LmigOR61	MKRGDTAVEEDEKDGEVLTWQETGGSVLKYNIRQLHVFGVWPLPGSAPFHAYTVLVSAIGLAGLAQDLAG VCACWGDLQEVTMALIHILSVSSGFVKLAFFVRRRRHFNALVRRTDRLVAAQGHFCDADATLRATFRASHR KAVYVTLLAYGYLSVQGVIWLPLPLIAHPGERRLPFMQMPAAATASVYVYALLYSLQCLSSMFVTFVGVTVD CFFAVVMIHTAVQFRILNTRISALRADAAVPVDTGTAVGGQTSEHDSLYKQLCQCIQTHQRLLRFVTYLDSV MNPIAMTQFTFGVIVVGITLFQASYSPSSSTMFKCVTWLPMPSTQIFLYCWGAHDIMDEGQSVSRALYSCGW VDAPPGFKRALRLVMSCAQRPISLTAGRVYAINRATFISLMNAAYSYYTLLRQFNSR
LmigOR62	MKRKEKKNSQTKKKINLTVFDDIEYTQQLLSILAIWPLVTKSSTIKKILSWIHLLVIIGSLIWNILFRCIFIYIYVK KFDDQIMLIGPTFFRVIILLKYLAIIYHRKTIKKIFNHIQTDRSGVECQEQQNIMMRNVQINRHVTLVFAIFMYS SGTFYNFVMPHILPLLTHRGSRNTSQRLTIFPGYNVLSDVQKSPIYEITFFFHIFSVFAGFSVLVLACNIAVVLVT HACGQIQIIIGQLNSLIDDFTNNEKTLYIKFSSIISRHIRVIQFSNNIIKDALYETCLVEIGASSVLICIVEFLLLKMI ENQNYANMIPYAMLLASLTLSILIICYFSELLESQFIEAGIQAYSINWYQLPPKARKYLILIIIISQRSYKITAGGIID LSYIAFVQILKTGFAYLQLLRATK
LmigOR63	MKRSGMEDLGRTVSWSESGRSVLRLNIRHLWLCGAWPLPGSWLYRWYAILGLVVGAVNAVESAVSLYFYW GDMEETTLLLTSSVSNGCGTVKLAVVMRNQRQYHALARRVQALMALQGDLVAADPALSAVVEAARRRAY RITLGMLLLMFSQYFVWYPMPFYVDPGARRLPFAQHAWDNNTRLYALSYFLQCAASARGTQLSWSLDLLF MSVMLLAAAQLQVLTRRITALQEEKREAKLGNAANSEKAALADGGGNMYDNLCLCIETHQKILSFIKYLD DTMSSVAMIQFCVSVLLVCVALFQATYSTDSTAVLRCALYLPMPGTQVFLYCWAAHNVTEQAEAVSLSAYSS SWVGTGRRIKQALRIIISRAQKPLVLTAGRIYPIDREAFVSLVNASYSYYALLASMSKRE
LmigOR64	MKSKVFYFTDDAPKSTWDKSTRIYHRLRPVLLVVLLIENAIGLYFLEGFGVMDGSIIYLPICLLLFVINSTAYFSR KAQDRLIATLNKHFTESNEPWMLTVSHKYTTKVWKYIKLILIYDKACKCMYLAAPLICDTVLHYVFDMLEK

	PFYLPTFITPWLPKDVTWGPHYYLILVFGWWGMLECLNGIMGFMIGYTLLVTYVLIQVVIFKEKVKSAKIDGS<br>QEEVDAEFRTLLSWHVDNLQLNRDLKAYFGFTCAFQSIFLSVGLTLTVFTAVSSKVIAVKIAYGVGFFFYFGTG<br>LLYCSLGQLLENEVQSKHSEKFEYDYATVSHTAHTRLPRSMQNELRKFHADFETVLFIFYLTTISNIVGLKFFL<br>PSSPTFITVGTKVVGTKVVGTKLAS
LmigOR65	MKSTFKEERIKDDSKRRDLFVFVRQTMCIAAMYPFGYYVNGSGVLAVLVRFCDLTYELFNYFVSVHIAGLYIC<br>TIYINYGQGDLDFFVNCLIQTIIYLWTIAMKLYFRRFRPGLLNTILSNINDEYETRSAVGFSFVTMAGSYRMSKL<br>WIKTYVYCCYIGTIFWLALPIAYRDRSLPLACWYPFDYTQPGVYEVVFLLQAMGQIQVAASFASSSGLHMVL<br>CVLISGQYDVLFCSLKNVLASSYVLMGANMTELNQLQAEQSAADVEPGQYAYSVEEETPLQELLKVGSSMD<br>FSSAFRLSFVRCIQHHRYIVAALKKIESFYSPIWFVKIGEVTFLMCLVAFVSTKSTAANSFMRMVSLGQYLLLVL<br>YELFIICYFADIVFQNSQRCGEALWRSPWQRHLKDVRSDYMFFMLNSRRQFQLTAGKISNLNVDRFRGTITT<br>AFSFLTLLQKMDARE
LmigOR66	MKTLPYSFMILEYFGGWKPLKWGTSIKGKLYNMYTLTVAVTLVTFCLSCIIDLFYTANIEDIVHNMSMSFTLIV<br>VCSKLTLVTVKRNEIIRIIKLLDINICRVRCAKEANIQLKYDEKAKNIVKKYGGLVCGAVFAVTGASILENIPTK<br>TLPFNGWFPYRHDNSTGFWVAYFHQNIAHFYVAMIGFSFDTVVYGVLLQNCSQLQILKNRLENFVEIINEEK<br>LKDKINGLSRTIRECEHKFIKQCTHHHWIILQFSQESNDLFAPIIFLQYLSSSLILCLCVQLLTKLAFMSPEFIFIV<br>VYLGCMLTQIFLFCWYGNEVIMESSEISSAIYKMNWQVLTNKTKKDLLFMKTQSILPIKFTSGHLIELSLDSFT<br>KLIKFSYSAYNLLHQK
LmigOR67	MKTSENITARKMSVYWLTLLLLNGINVYPGHGNRVGRIATAAYPWMICAFWAFITTSVTTSLALRATNYEEV<br>VEMLTYITGSSCTLALFAIGVHKRPRLHRMLDAVRRDFWDDGRRPAADVLFSRFVRTYGAILPVANVMMC<br>MTPVIWAARNGDIESPAALIFRMWTPWTRMTTTRYAAVYAAQFVVSLSVLTSIAGMVFAMVLFVTEMQVQ<br>VDTLVDAVQDLQVDMWYGDGHDSPVTHPDRRRRAAFDGLVKCVKHHQALITYFNHFKSYFNLLFIVDILY<br>IMVMTCLCASSVLMANGFSAFHIKMMSLLIIVVSQFFFYCLIGEQFSTMNQQIGDCVYFKLVKCKDPKLSRA<br>GLLIILRTQKPLQLTAMGITKYSASLFTFTVTMRSAYAGLNVLYNSS
LmigOR68	MKVLDNVNHAVKVTMNCCRLYGLFVSDDLTKRQLIIMRAFSLMLYLFFVGFFITTQSALIITMWGDLNLMT<br>NVGLVLGTHLTLSAKVFTLHYKEKEITNVIYKNEVRLRAETREQGKYIISEMNRETTLFMRLFIPFGMGTVTA<br>WLLCTPKGELYTPAWYPCNTTKSPAHEIILAHQGIAVILTATLEIAIVLLMTSIVAVCRCRLKLVGLSFETICDDL<br>PSNIMNKLTADEQVIVAKRVRENVIEHQAVLECINDIQDCFSSAMLVHIAISTMIICATAYQLAVEKSLDLTQR<br>MTMASFLGGMSTEIFLFCYQGGHLSIDSMEVATAVYSCPWYTFPTSLKRSLLVIMIRAQQPALLTAGGFAPLLL<br>DTFVSIMKASYSFFTVLQNASE
LmigOR69	MKVNPSDQNSTVIQNQSSEPENIVNNEQDFEWAIHYHRKVLKFCGIWIYSNSNRWYLKLITDLHSLFIIGGV<br>LICLTTPEAMALVKIWGNLTLIVDNLLSSAALISTQIKLFVLWTRRKAIARIVEAVKSDWLEPKTEAERKIMRR<br>YARIARIMMVCGLSNIAYNLITFHGSVLFGFVYRTVNNITDIEGYLIPTQSVFPFDITIGYRCWIIRIVQALQCFG<br>AGITYTAIDVFCGMSVLHNCGQLEILADKIKDLVNPDEPRVFQELLKTIVLRHYRIIGLIEEIRNIFATVLLLLVL<br>CFGILFSVIGFLIASSFESDGTKVPVSQMNFYIGYILFFVGLLFVYSWVGENLLSHSEEIHVAVYSCNWTDLEPH<br>QIAQLIIILVRAQRPLEITIGKFAPATLNTFAQILKTSAGYISVLLARNG
LmigOR7	MKWLQDHEVAINLALFKRYQFYHIFDPNGSKLLSYDTYKLTNVMFIVAVTTYNIFSAMCFFTDTVNSIDSVD<br>LLLMIFIYSIIVISLLKITVLLYNADQIWELFDLARLDFLTSKRCRKNIGILIKYRDTSITITNLYQNYSTIVFIIWM<br>VVPLILNTFVLVEGPNQRYHNIFNMQYPVSASIYNRYYYLFYLMEIAMGIFVLNYSMIIDNFLISLCWAIIAQY<br>EVITTAFENIGNDCELENLQNRRENKSFEAYEDLKSIIMDQEKVYIKLKSFYHVVWIIVIFLIIIDSVLLIILTYSFV<br>MICSSAESFSIFNILKISTAFFVFVIQLYLYCYLFDVLNDKKESVNFGIYSCDWTKMDIKFKKLLLLTMKLNNAD<br>KLKIKATPNKIVNLQLFSSVMTTTFNIVTVMLKTMKGKN
LmigOR70	MKYILMKKTIAFLSVTGFWPKTKESTKTRAFCILFSSSFLLFGSLGYLIVYRKFGSDDIDSIETATSHFGVLYFMF<br>FWILKRDGLVHIVNLLSDFSKFGEPRFFNDRNRQLDYLLQYCIFVLSVATGGVFLCPIIFVKNCEMVKQEKNL<br>TKVCGLVSNVWAPFDYSEYPMKRVVSLWESYCCFINFGCGGIMSFTMIKTMEHLHIRVEQLKDMFPDVVNE

	KNLAVRKQKLEKWVKYHLHLYDIGELMNNTYRYCLSVIVLCVGILFGCIGISTMQPGSSHNSLFLFMGWFQSICILCMVGQRLLDVFLSVGVMAYDSAWYEKDVDFQKAVLMIMIRARRPVLIYAGPFTNLSHLLILGVLQTSYSYINLLNAK
LmigOR71	MKYIREVENIKAGIFSSCGIVAEEQRVEMSEKNQQDYFKIPDKCYNWSGVRVSSNALTKFVSLYILYPLMLILYFMIIYNIRFKKNISDITEVFISISTFTIITFRKTLLIRNGSIYEDILKKQSHYWKYYMFGKPTEMKLRKSMEFCVMVIKFLIASTTGSIIFHSIISPIIEGKIVLPQPCWVPNNDPVANDIIFALENIFYMEGTNYLVVFDGLYLLMTANLKTQMILLRKAVASINFREDEKTTWAKLKEYCEYHKFLLRIHGKINKIYSAFFLMTYVFTIMGTCTPLFVIFYEEADMVLLGKSVFIALILNTLLVMTFIPAGELEIEAEKLSFEIYSINWYETKNLKIRKFVLFWIMQTQIPVQMSGGGMLIVNRPLVLQVQRIAYSLTSFLAGLS
LmigOR72	MLAFVLYMLFVVSVPILEVLNLVLQEKITFKQIIDNAFMIAELGCLIPKYWPFVRNNDRLVKCIHYFDSPAFQPTKKKHREILQNCVKVCRAITIFFVAAVSSGYVSWSSRPISWKNHIFPTDLWLPYDPKVAPKLYNFLVYTYLIKILKDNLQHLGEDTEAEFNQQSVINRLPKSEIMYRKIVKCVEHHNLILDFVKEFQYCFAQCAFSQIAGSVVVLCVSCLQLTIVDLLSFDCLAMILFLVSMLSEVYFFCHFGTLLYEESSTISDAIYMGSWYDYDKKSKQALTILMERTKRPVIVIAGKLVQLSLITFSMILRRSYSLLAVLENYNIEIN
LmigOR73	MLALDDPLQNVDNVEDFKYVKWLRNHLKTVDAWPVYSKSKRKIQKRYVLPIFSAACFISQTVYLKNGIGTLSFVVLVHSYICFLINGSCLCRGILIATERCKRLATCYLKTVHLFHHKNRSEHAMKIHVIVHRLSHYYTIYLISLVFVGMVLFNFMPIYNNINSGAFKSPRPESVTFQHAMYLALPFDYTTNIKGYFVVFILNWYISLVTTSHFCTFDLFISLMIIHLWGHIKILMCSLEDIEGFVLGGSFKFTIEQNRKINSILQECIRHHQFTIDFTNEMSSTFGLVILFYYFFYQVSGCLLLLACSQMDIESLSRFGPMTFILFQQLIQLSIVFELISSLSENLPNAVYNVPWESMDKNNRKMIQVLLLQSQKLTRFKATSMMNVGVQAMATILKTSVSYFIMLRTMYQEH
LmigOR75	MLAVLNTPQFLPKSDTQVDLVKPSLKLWKLSYTVFSVIVAMNVFLWSIAPFFNPEQRFFPFVAWYPFQTSTTLNYCIVYLYQVVCIWVITIANMNLDTVTMALMVYIGTQCDILCDDLSNLYGNSDYFHKHLISCIKHHKKIVSYARKSNNIFNMIILGQFATSTMVLALTMFQLSLVNPLSGIAAIHLNYILGITTEILLYCYYGNEVEVKSSRMSYSIYKSDWYEQPINIRRTILILCERCKRPIKFTAINLFALSLSTFMTIIRSAYSYFALLYNVNNR
LmigOR76	MLDLNDPFAVLKKFYIDFGYHKIVKSCNTFFITLYSLAYALQIYYLIYHFTPDIIQKYSLALLMSIYLLSTLIFSAVYEKTILQTYDMFIRVAWPHTNVSQKLETIIQKRVTIIKILNNFFVSAICVMCVFNMPLFGDQSDFLLSIQVFGEYFGDWSFIPSFFYYLGFSILGYNAARMLFILLYGVLHLEVQILLINELVSKFSEINTLDDFGNEEYQSCVYTILCDCITHDVVLKKIMSDLSRKVQNGIPIFLIVLLFCLISLFFFTLTYMGTLSFIMQFRMVAFLLTIIVVIFSYSMVGQHLLNQASLLFDELLKCPWYVWSTNNHKIYQIFLLNSIRPIKIIYAGVCLDGGFFLSMTRITFCNAYMLKQLRDS
LmigOR77	MLFGAGSKELGFRKETIEEEDVGFMQLAGLYPLTRGYTAYYFISFILCTTLMEGMIVGSYLEGEVDTSLETAHVLLISLNMFTQICTHRYYYDIVNKLLRAIDDNFFSYGDTMDEDTKQVIRKLAKEKTARKKMFGKVFKIQVSSAAIAITFQRPILYVLNGRGVKDVDGENWLIYQSPFGILVPFSNYWVPYLFGMVLVVNQVITTSITAMATATSFVRFSEELLHQLEIVKLGLGNFMFRARHLHSLRYNQSKESGEQDKHLDKCIITCLSKSVEHHAIIIKLFGDFKNMMYIPLFTVIFDGSVLICMSAVQLITSKNPIVRMSMPPFIVAELYYTYLYCSYAEKLTNMVLRTAYSLLNFLATRK
LmigOR78	MLFKATPEFAIAFTKLTSILGSSWPHYKNATKCQLIVFNIKWWFFWFMSITAFLPMCYAAYNNTKNILSFTKSLCDAANCSQAFIKMLLCKIHYRKLQFLFYEMEKYVEQARANERELFISYIKRCGRLHVSIMISAVMAAVIIIIAPIGMPQPFPNVAEYPFPVDGHPTFEIIYLQQSIATIHCMSIPVFDCQIALLLWYAGARLELLGDEFRKVTDNQQFVACVKKHQYLLWFIQEIIMSSRHILATTVVMCTIAVITSGVHIVGKEPLADKVTSVILSTGLSAVLYLCAWPAEHLAQMCENVGAALYCSTWIKNSKESNKNIFIVIQRSQKPETIQVPGILPILSLTYYATFLSKTFSYFTTLRVVLDKMED
LmigOR79	MLFKATPEFALKFTKLIALLGTSWPNYEGTPKWKLVVFQIRWWSTFFLAITACLTMCYAACNQYQNILNLTKSLFDISNTSQTFVKMFFCKVHYKRMQYLLCDMEKYVTKAKPHERDLFIKYIKRCGKLHLTVMGSGLLIIHIIILAPIALPQPFPNIAEYPFPIDGHPTYELLYLHQSCATIHCLSIPAFDCQIAMLLWYAGARLELLSEECKTITDNKQFVECIKQHQYLLWYIQEITTSSRHILATTGCTCILTAISSGVHIVSNEPVAFKVPFMISWVIVSSTLYITSWPAENV

	LQMCEQVGMALYESPWVQNSKELNSSILFVVQRSQKPSTIEVPGILPVLSLRYFAMFLSRTFSYFTTLRVLLDKI<br>NLDMEIPAED
LmigOR8	MLFNYLRKPNPTNLLTSPDSFRYFEYGMFCMGWHTPATHKIIYYITSCLIFAWCAVYLPIGIIISFKTDINTFTPN<br>ELLTVMQLFFNSVGMPFKVLFFNLYISGFYKAKKLLSEMDKRCTTLKERVEVHQGVVRCNKAYLIYQFIYTAY<br>TISTFLSAALSGKLPWRIYNPFVDFRESRSSFWKAALNETALMLFAVTQTLMSDIYPLLYGLILRVHLKLLRLR<br>VESLCTDSGKSDAENEQDLIKCIKDHNLIIDYAAAIRPAVTRTIFVQFLLIGICLGLSMINLLFFADIWTGLATV<br>AYINGLMVQTFPFCFVCDLLKKDCELLVSAIFHSNWINSSRSYKSSLRYFLKNAQKSIAFTAGSIFPISTGSNIKV<br>AKLAFSVVTFVNQLNIADRLTKN
LmigOR80	MLFRKRKPKSDDEVITFDELTRFPMTFYKTIGEDLYSDRDPNVIRRYLLRFYLVLGFLNFNAYVVGEIAYFIVHI<br>MSTTTLLEATAVAPCIGFSFMADFKQFGLTVNRKRLVRLLDDLKEIFPLDLEAQRKYNVSFYRKHMNRVMTL<br>FTILCMTYTSSFSFYPAIKSTIKYYLMGSEIFERNYGFHILFPYDAETDLTVYWFSYWGLAHCAYVAGVSYVCV<br>DLLLIATITQLTMHFNFIANDLEAYEGGDHTDEENIKYLHNLVVYHARALDLSEEVNNIFSFLILWNFIAASL<br>VICFAGFQITASNVEDIVLYFIFFSASLVQVFVVCYYGDEMISSSSRIGHSAFNQNWLPCSTKYKRILQFIIARSQ<br>KPASIRPPTFPPISFNTFMKVISMSYQFFALLRTTYYG
LmigOR81	MLFRNTEIRRFQTKAKDPLHFLYASTFGLFRVKLVRLVSIFTLVFHSVISCAFFCEMLYSFDPNLLIEYGPMIFIF<br>GSGVMAIAVFFYIEQNGRLLLRQSVGRLWNFDSSSNLYKKMKLESKYILQVVHFNNFLGVCMVFFHLPAGG<br>QSDKVFYGINLFRRILPQHLNFLKQIYYSTFPILTYMVTVNPYLLLYASSHMKFQVCYVNELLVKMTKDYKDI<br>DYNLLRNEKYQKTVTLGLKNCIHRHAILKFMDKKLNKLIHWPLLILMALSVVAIISLLFIAIVTRQDVHVPSF<br>GATMIFGIVTNSLAVYSGQEVINESMKSLSCAGTSRWTSWNFFNRKMLVIFMTNAQKPFILQSPFFVCEFAFV<br>IKALKFVSSVCGLFFEVARRRDLGEL
LmigOR83	MLGQGQAGPGGRGQHLPRVDLLAHLSPILRCLAVFGMWPPSVYTSVSQKVAALTTGAATLSLFLMQLVAE<br>VMALAASPVSGAAELYRFIYNFSVVDLHLQGMGKWAVMVARRRRYTALVHRLQHCVRLSGLADADYKNQ<br>QAAVSRLRQQLDDCRRWGVRANAVWLSVCVYGVTHWCLVPLLIGDNSLPFDALYLFSTDQPPLRQVAHCI<br>QYVAGLQNVLLSVFFDLFVFWLHLLLCAQLRYLAGNLRRLRHLTDAAQYRRMLAACVAHHSHLLSTMQE<br>LNSCAGPSFFLQCFENTIRMCMIAFMATTTVADQMQVWSSAQFFLAAVAQLFLYCWCGQQLSHLAESISEA<br>VYDSGWEDHDVSTQKTVAFIMWRAQKILVFKGGWFYTLTTETFVELIRLSFSYYTVLRNINDT
LmigOR84	MLHEWTLFQKTGSSNSSDKEHYDIKETKHFATCRKVLVMMGFVNDGSVIYKLRQPVFVFLLYSAPVHHLVP<br>AFIDKTAPSDQILMSWSISMLYVLLCIAWPFMIIRSSEIFQLWATVRKGFYHYSDPFTAAERTILSRTNDIVIKST<br>RVSIIAYFCAGFGTFLKEMQPESLRQYKAPYPGWFPWTINSNFRFALALLYQCAICLNTTFALEAVFVLFAYHT<br>IHFEGQLRLLTRHFGDTFPPGLPATVTYSAEYKKRTLRRLYECVSHHLIITGFHKQILSYFGICLLVYRVIVTIML<br>CILCYLTTTGISLDKFVQLLCFALALLYLCFIFCLKGEKVTQLSDGWRLTVYEVDWWNHPVEVQKTILMMQ<br>MGASKALKVYGVWKPAMYSHEGISVIGQETFSFFNMLRAM
LmigOR85	MLHVYLGTCFTASPLRQWVSAPACRQVATAAQEVRRLVGPAAPALEWLGLWHPPGSPPTGFKALRGATVL<br>LIDLLLFVFCSLLLLLDPPADAEGQRETFFYIMASFTWTVRGVFFMLERTRHEKLVSILLSLRQRFPDDGCDIR<br>GTHLKNAALLSLAWQVAPVLVLPAWIIEPMLETHYVTYGNITEVYRRTMLYMWTPGDMQQSPNYEISYVSQ<br>VVVSLIAVEASVLQDIFFVNIMVQVTSELDVLNANISSMRLPAAAKSQSVANGPEYSDTKLEQYEATYFSHKS<br>KTSAEDELQRSVTIYKNTNDENEELYKKLVKNVRHHQMIMVCIDELEAAMSKSIAVVLVISTLNICVHAFGF<br>VGMFQEDAPRVTVFKRTVAFVIYMTHNALFCFLGQSITDQSERLLHSTFSCGWADADRPFKRSLMIVMRQTS<br>RPLVINVGKFFTLSRNTYMQIVNTSYTIFNMLLSVQ
LmigOR86	MLIHSDIDKYIKFMKGYCVWYGKSVTWDDTRFDLCRKYYTESKCYLSAVLLCVVSFALYSTDDFGLQDGTFI<br>YWPICLMMLVLTSIATATRHQQDVLTMSLNDNFLENTESWMRAIKDQNINRLWKMLRFYTVYNNTISALY<br>MLVPLVVDSILHYGFDYLQTPFTLALPLTPLLKYSNTWNAQYYVLTAFNFWSCAEMVFMLEWFLGNYLLLT<br>TFFLTELIILKHQVKSLDFGKNEEWDQQVESIVNKHSKIMKLNVELRDYLGLPGAFISFFSSILLTFTAFVSFTST<br>SIPLRVSYGSGFVLYFGAALLLTTIGQKLENESDELFKAFYSLRWYQYSPNARKSLNMMMRQARTPLIIDFHG

	RYKMNLANFMQILRSSYSYFTLLREVAKE
LmigOR87	MLINCYIFTILISFSEMLNPQHDRDALKIVKFLASDIFHNKSAKLFLLIVALIHGSVTLIQIYCTLANPDIKDFILKAPIFFGMFFPLTGVITLLLRPELIDDVPQYMKNWSMENVDAKIHTWVQITRIYGVGTFILAFICGLSYSQFLINENQFFFFYQFLDDFVPKYEFRIFCKIIYKATFPVMFYAAVLHAIQTAYGTQHMKFQLIMLREKIEKIDCNEKHQKKIRQKLIYCIRRHDSIITFGRNKMEQMSELIGIFLTIATLCFISILIFMFAGPFYKEYYVRLCLTCATAVAAFGSLLVFGQKLEDEIEQLATVTYNLKWYNFDVENRKIYLLFLLNVMKTFQVKYLDFAINYRLGLSIVRTVYSVLSVMLKMDSKLYDKQN
LmigOR88	MLIYNRFSKNYFSMKKLNDPLRFIYKMTLDAIDYKIMQFFLTLTFVLHSFVSVAFILELTLTFDADLLVTYGPIIFTFGSGIGGFLVVYKIRRTLKLLLQKCKLPAPDSKSQMLERIKRESKIILNAVYFDNILMIVTVLFHWPIIGENNNIYYATVLFEKVVPSFSSKLCVLYYLSLFFIVYLVAMNPYLLLYTSTYIKFQLCYINELLTNIDAKWSKFGDYQLMRNNVYQEMISEELKKCIDRHRLFQSLVKELNNLIYIPLLILMSLAILAIVSILFNIIVKRQETFYCRVCSTLLFALLTLSFAIISGQLIENEWKKLIERAFCCRWISWNFENRRTLLIFFMNAQRPSRIGNAFIRCEYSFLLTAGRFVCSLCAFFFQLARIREEEMLQI
LmigOR89	MLKFEPKPTTKDELLWVVRTIYVDLFRNKLIQFALKMLFYGSIIMAIYQGVLFLYEFEIHYFVKYSSMYCFTCFILLAAYSVPIIAEVATTAFTTIKCWKIDSGGALVENKIKQEAHFTNIITAINCIFGLMVLVLFIVPFEDDNDFYFLFIAFEKYFPQWQQLLKWGFKAFFPCITILLQAPFYIVIYACLRIKFELYMWMEFLKNLNIVYEKSDICELVHDSEYQTEISKRLRFCIERQEHIYRSLLLYGKKYVQQLDIYIFAYAILGSLGGISIIFVCISFEGNFFQGTYLRLSALTFVTLTTFMHVIWAGQSVETTSSDSYDILKQCDWFLWNLKNRKTYLMCLNYTQRPLKAQFTQNVSINYVLGFSVVRTVYSTLTALNSLRKASK
LmigOR9	MLKMAKVEPVERYCKVIRMIRFCVGFCGNDVADPNFRMWWLTYAVMAAIAFFFACTGYTIYVGVVINGDLTIILQALAMVGSAVQGLTKLLVTANNASHMREVQNTYEDIYREYGSKGDEYAKCLEKRIRITWTLLIGFMLVYIILLGLVITFPIFYLLILHQKVLVMQFLIPFLDHTTDGGHLILTAAHVILITFGGFGNYGGDMYLFLFVTHVPLIKDIFCVKLTEFNELVMKRNDFPKVRAMLCDLLVWHQLYTRMLQTTKKIYSIVLFVQLSTTCVGLLCTISCIFMKAWPAAPLYLLYAAITLYTFCGLGTLVENSNEDFLSVIYTNCLWYELPVKEEKLIIMMLAKAQNEVVLTAADMAPLSMNTALQLTKGIYSFSMMLMNYLG
LmigOR90	MLLCWKKKKEKQKPVIKEQHDFANNYRLFKYIGMIQDGSLFSRIRVFLATFLLFYAWFHHLIPLVNSTEEYSFDELMDLIHLEMVYFLWCIVWPSYIIRAPLFTSLASKIQNGLYTYSDPLTLEEKTILSTANDAVVRMTKISVYVYVCGGIGIFLKGMNKERMRKLQLPNIGWFPFAINSLSRYAIGCLCQAIMGINAISIAIGTFMSFAIFLIHYEAQFKLLRTHLKRSFPKNVPLRIAQTDKYKKVSLRRLKDCYRHHLAILGFHQEIMKYYGILLLVFRVAIVMWMCTLAYVTVMVDVNAHNLLKMLSFASTELLYVFLFSFRGQDVTDWNYQWREELYSIQWWEQPKEVKTNIGIMVLGTTQPLLLYGVWKIALYSHEKLSDIGNESFSFFNMLRAIN
LmigOR91	MLLKWSSVIEFNLFLLKWIGLWPGEDYQLNMYSFYGFSVIILILCGHTLSTGLTLILDSGDIDTFTETMFILNIEFMTAWKALNFALNRKKFMQLLDAIDKTTFQPRNGKQVTLVLRNIDGWKVMFKMFGISLGLSFIFTGLLPIFSKTYKDRKLPMEAWYPFDSTKSPFYQLCYVYQMAAVAVAVMVILNVDTLVAAMNICIGLQCDLLCDNLRNLHTNTSKMQVFIYCWFGNEVIVKSSKIPYALFESDWTQDSLEMKKNMIVFILRTQKTLKITVCHVFDLSLPTFLTILKTGWSYFAFMNRVTSPH
LmigOR92	MLLSFKDDSRSPDIQKPQNFQYMKILRFNLKIICAWPEKQLNEIRSLGHSIHRVILPIQSVVCLACGILYIHFHFNEIPFFILASTFITVMMNLVTCSRTALVMLFERYLVLTGRFITVMHLFNFQKNSDYAYKLCTFVNRMSHFYTLYVLFSMFMGLGLFNLLPLYNNYVSGAFSDPYGPNVTFFHSVYFAFPFDYSHNFRGYIIMALFNSYVSVTCSIGLVMFDLLMCLMVMHVWGHLKILSHNLINFPRPKASHVITTPNGPTNVETYTEEESKEVFARLRECIKHYGTVDDFANDMSETFGVILLVYYGFHQVSLCMLLLECSDLSTKAMLRYGPLTLIMIQQLIQISIIFELLGSVADRIPDAVYQLPWECMDVKNRRVVYGFLRRTQNPVRFKAMGMLDVGVQTMASILKTSISYFVMLRTVAT
LmigOR93	MLLYHPNTQVEEKVNNVEEFTYMKFLKSFCKIMDFWPEREEKNSKTRIFRLRYILVLQFCFTLVAGVLYLKN

	NFGKKTFYDLGHTIITVVMNVVSVSRLILRCFKKYDVVGQQFINKIHLYHFRNDSEYSMKTYKAVHKISNNM TYIFSFSIFVCVVTFNLNPVFNNIGSGAYKNPRPDNVTLQQCVYYALPFDYTGDFKWYMLVAIFNVQKTFFCT SLFILFDLLLSMMIIHLWGHIRIFIHNLNHIPAPRNSLEYTREERQEVDNTLKKCIQHHTLIIGFVRIMSETYGL AVLIYYAFQQVVGCLLLLQCSRLDLKTITRFGFLTTMVNQQLIQISVIFELLGYMNDKLQEAVYCVPWEYMD TSHRKMVYMMFRQSQIPLQLKAMNMLSIGVKTMASILKTSVTYYLMLKTITANEA
LmigOR94	MLLYYPNTQVKEKVNNVEEFTYIKFLKSFCKIMDFWPEREEKNSKTRIFRLRYILVLQFCFTLVAGVLYLTNSV GKQTFYDLGHTIITVLMNVVSLSRLILRCFKKYDVVGQQFINKIHLYHYRNDSEYAMKIHTVVHKISHNMTY IFSFCIIFGTVTFNLTPIFNNIGSDAYKNPRPDNVTLQQCVYYALPFDYTGNFKWYLLVAIFNVQKTFFCTSLFI LFELSLSLMIICLWGHLRIFIHNLNHIPAPRNSFEYTKEERQEVDDTLKKCIQHHTLIIGFVRIMSETYGLAVLIY YAFQQVVGCLLLLQCSQMELKTVTRFGFLTLVLNQQLIQISVIFELLGYMSDKLQDAVYCVPWEYMDTSHRK MVYMMFRQSQIPLQLKAMNMLSIGVKTMVSILKTSVTYYLILKTVTTD
LmigOR95	MLNYSKDEDCIMSSLMAKCTGLHYVIDPKSPKLGGHNVFHLTIMAMIAFTVACVSLCPFGLYHWANDMTQ CLIQLMIIGNFSFGCFKAYMIVRHSDDIRRSLDVTRFDFVSSAIADPDSARFFRRCRNVSATFTGWFAAINHFV LMLWTLLPFVIDSNKVEVKNRDGSFSYYHFNPYNLYFLVSSETYNEWHLVFHLIEWMFGLIFILVMILFDTW MVTLCIAITCQMKGIADAYRKLGHCRSRTAPNAWFGDEIESADSSNNEYVRDLKLVIKHHQAVLGKMNDF YKIVGPVILPQLIVASFTIIFVSFIITRNYFNGMLLTSTISLKMCCCPTFFFQIYYTCLVFGNIDHQKNVMNFALYS CDWTHMEIKFKKLLLLAMEMHDAYKVDMKLTAEMIVNLELFTSVINLCYSIFSVLVNSQLKIVDRL
LmigOR96	MLPGFILLVANCVIMVPYISKWVVGMKNPYTPRGVNLNLPVACWLPFHSHTGFWHVVAVSNQLIAVSCLA VIIITLLFMFLKFSQKVRYELKVLHYGIETLFKRSKRLYFKMYPERKAIRFHWTDPVYQRVVGICLKDSILHHK TIVNILDVFMTMVSIPAALAYVIGTAVIGLSLLSILNALNQGNYPNVILFALLCVGEILNMLVASLIGETLTHETI ILREELYFIEWHKLNLSNRKTMLNFQTAITEPLSMKAAGLVDMNMDTFSSIMNSAYSFFNLVNAQ
LmigOR97	MLPRFLTASYPMERHYFMVPKFALSLIGFYPEQKRTVLVKLWSFFNFFILTYGCYAEAYYGIHYIPINIATALDA LCPVASSILSLVKMVAIWWYQDELRSLIERVRFLTEQQKSKRKLGYKKRFYTLATQLTFLLLCCGFCTSTSYSVR HLIDNILRRTHGKDWIYETPFKMMFPDLLLRLPLYPITYILVHWHGYITVVCFVGADGFFLGFCLYFTVLLLCL QDDVCDLLEVENIEKSPSEAEEARIVREMEKLVDRHNEVAELTERLSGVMVEITLAHFVTSSLIIGTSVVDILLF SGLGIIVYVVYTCAVGVEIFLYCLGGSHIMEACSNLARSTFSSHWYGHSVRVQKMTLLMVARAQRVLTIKIPF FSPSLETLTSILRFTGSLIALAKSVI
LmigOR98	MLPSADDEKRLLGLMRFLLGRSVASRDGSFRTQLANGATTATLLSTAAANLIIMVCSGLKLYLDPPEETEKAS QVAFLMTVSVANAMKGFSMVQQRARLQQVVAGLLAMRRAVCDGSGARHRYACSATLIGNIWMVMSVIL GIVWGVDPVFNQPPQLNGTSPDPVLPLPIWLPLDASVPLTYWLMFALEAVVCGWTIFFVMIVDLLYVTLILN FAAELHVLNHNIQITCNAVDVTAHPKRKIGVSRHAGVSLYKGHNDDTAAIPNFSLAHFTAANPLMPAYIPD HFRVELSEDYDTYRLLVKSIQHHQLIVKCVNEFGKATGLPVLMVVSINVVNLCSNIISLAVLVEEDPHASAIA KSLIFTIALGSQTALYCLPGQMIIDQSERLAHSAFCCRWPDAGVRFKRSLLVFMACAGRPLRLRVGKLVTLSR ETFQELLKLSYQLFNLVYQLQSS
LmigOR99	MLRLGRHHPTGSIWWTIFFIPVNTFFCSLLIILSIVGIVRYHEDDVFLAVDCLGTCTLMLHAISKQIFLHAQKN AINVLLKMKSQFWNLDDFDGEISKECEMILTSGKIAVRIYFSFTCAAATFYFLQPFTAHHLPSDCYVPEGWFP FLAISYMYLIPTLVPSVVGLDALFWALGLSLAVQFKLLAQKFKLLGTCHENETAILWNQLKELINYHRFLIDF CKKLNKLFSFIFFVQSFITITSASVAVFIVMQPGNLSTRVKCLLTFVSYILEMAFYCLPAEMSVNAAIDVADSVY NSKWFRIKSTEFKKCLILIIGRAQIPFTFSGFGLIHINIRMFQLVCKTTFTFYTYLNTVQNRQ
LmigORco	MLRLLGLYQQKGGGGRVKPKLLSALSLIFLLYHPVFAAMKLYMEPPEDLVEFALCSFSFIISDGVFVKTAIFIAD RGMLHQMLQVLSDSRRLYGGEETSKKIRNRYENLAERVLLYMQVSTMLASVGWLAAPLVFRALAMASGDG GEVPRKLPLPVWLPVDVQETPTYEILYVIEAYCVTLTGLVTLCIDVLFIRLMLMVTAELEVLNYNVATMAKRR EKISDERRSLEYQQGTESQDFYRGDKALPMASESYEDAMDNELYQQLVTNVRHHLIILRTVDLLEAAMSKSII ILLFINMGALCSNLLVVGVLLQAGEGVTRPLTLTAMIPFLLYQTGMFCVFGQMVTDQSEMLTTSAFSCGWNE

	SDARFRRSLLIFMAMVNRPLEITVGKTCKLSREMFLQVLNGSYTLFNMFYQVHSTS
MmedOR1	MLRQVYEALRDADEPSRYLEFNVLMVRFMGVLVSRSLTGALLTWSLFVLLATHCLAGVFDLVNNSGDIADI<br>TANLPVTTIIFSSTYRLFFFTLHRDRYQAIVDTVGARFVASSDSIDMAPWLRRSRIISILYFTYGFFVASTWQLHP<br>LISAQLTAAQMAKESNGTFEGLPRELWEFPTRAEYPFDARQPYVYTVVFILQGAAIFVSGCMILVLDMMFITL<br>TSLICGQFEILKDKLRNMRKIATNQRQNEDIGLVGKALQKQALERRKRILDSNIEITKQDENFNEDLITRKINL<br>LLGECVEHHNMLLSLISEIEFMHWSAYLVNFCVLLIIFSFSAFEVTSGTPTSPAKVVNLAEYLLVSVLQMFLLC<br>DCGDKLVDQELSVSQAAYESEWYHCSESVKRTLQIIVLRSRQPEQITVGKIAGLNLDTFSDMLSRSFSYFTVLR<br>QIRDDS
MmedOR10	MLSKFFPHIKEKPLSERVKSRDAFIYLDRVMWSFGWTEPENKRWILPYKLWLAFVNIVMLILLPISISIEYLHRF<br>KTFSAGEFLSSLEIGVNMYGSSFKCAFTLIGFKKRQEAKVLLDQLDKRCLSDKERSTVHRYVAMGNFFDILYH<br>IFYSTFVVMNFPYFLLERRHAWRMYFPYIDSDEQFYISSIAECFLMTEAIYMDLCTDVCPLISMLMARCHISLL<br>KQRLRNLRSKPGRTEDEYLEELTECIRDHRLLLDYVDALRPVFSGTIFVQFLLIGTVLGLSMINLMFFSTFWTG<br>VATCLFMFDVSMETFPFCYLCNMIIDDCQEMSNCLFQSDWTSADRRYKSTLVYFLHNLQQPITLTAGGVFPIS<br>MQTNLAMVKLAFSVVTVIKQFNLAERFQ
MmedOR11	MLSQFFPHIKEKPLSERVKSRDAFVYLDRVMWSFGWTVPENKRWDLHYKLWSTFVTLLIFILLPISVSVEYIQR<br>FKTFSAGEFLSSIQIGVNMYGSSFKSYLTMMGYKKRQEAKMSLDELDKRCVCDEERTIVHRHVALGNFCYIF<br>YHIAYTSFLISNFLSFIMKRIHAWRMYFPYVDPEKQFYISSIAEVILRGWAVFMDLCTDVCPLISMVIARCHITL<br>LKQRLRNLRSEPGRTEDEYLKELADCVRDHRLILDYVDALRSVFSGTIFVQFLLIGIVLGLSMINIMFFSTLSTG<br>VAVVLFMSCVSMQTFPFCYLCNMIMDDCQEMADSLFQSDWTSADRRYKSTLVYFLHNLQQPIILTAGGVFPI<br>SMQTNLNMVKLAFTVVTIVKQFNLAEKFQ
MmedOR12	MLTEYNLRFLKVLHYYGFWITFVGWKHDKIKKICLPVRGVVIGLSSILAIYKLGQEGIQCIINGTVVYFPFMVY<br>TFTIPILLFKNRKHFLSLLLDFEDCWKFFCEEKERKVLERHYKRIWKVANYVHICFFFEIAFYAVMQLALDSVL<br>HYVFDYLSKPHVLTYPHMGYLPTNRTWDGLYYVVAVVGCYNLVETLSVQLGWVILFVVIVAYCYPILLLTEK<br>AFKNLLQYDGDPDSAALKKAVQSHQLLVKLNKDLKAFLGLPCAFETLFVSIILTLTAFTSVTSTDALVVGAYS<br>SGFILHFLAALLYFSLGQLLENKSEELFTILYDLRWYTFSPAVRKDLNMMIRQARKPFVIDFHGNYKFNLENF<br>MQILSTSYSYFTIIQTLTDNH
MmedOR13	MLTKKDTQSAKEQEKLKAIPLHSFLKYANVFYLSIGMMAYDHKYSQKWKEVLLHWTFIAQMVNLNTVLISE<br>LIYVFLAIGKGSNFLEATMNLSFIGFVIVGDFKIWNISRQRKRLTQVVSRLEELHPQGLAQQEPYNIGHHLSG<br>YSRYSKFYFGMHMVLIWTYNLYWAVYYLVCDFWLGMRQFERMLPYYCWVPWDWSTGYSYYFMYISQNIG<br>GQACLSGQLAADMLMCALVTLVVMHFIRLSAHIESHVAGIGSFQHDLEFLQATVAYHQSLIHLCQDINEIFG<br>VSLLSNFVSSSFIICFVGFQMTIGSKIDNLVMLVLFLFCAMVQVFMIATHAQRLVDASEQIGQAVYNHDWFR<br>ADLRYRKMLILIIKRAQQPSRLKATMFLNISLVTVSDLLQLSYKFFALLRTMYVN
MmedOR14	MLTVGSSAHNSLSRILEVIGITRYKEASFFSENSFKLFRVVQWALNVLMIVSCLNFIFSESVDDSPDKLQSLALC<br>TADIQFLTTQLILISRQSLVDDLVAHLRSVYHGIKEIPGSMDILAEGDRQARLFIMSYGVVIFSNVPSSMFFAGIK<br>MILTGETAYPFPMSIFGLPTAVGWALQMLMIANAANILWGFYCVLKTVIYILGAYSNVMAHMLRERPIDVEA<br>KNDRKMLKLHCDINSLSLKLVNVYGLISFLEVTVASGRCCFVAYHILLAVQEGDYKNLGVALSTLLTSVAITY<br>VLCSCGEEISMQSVTIRDGVRDSKWYAVSPPARKTLLPVLLFTQRPIQFHYRRFVYFNLETFRNVLKTAYTMTT<br>ALAQV
MmedOR15	MLVKWSSVIKINIFLLKWVGLWPGEKYQLNVYSFYAFTVIILILCGQTLSTGLTLILGSGDVDTFTETLFVVNIE<br>FMTAWKALNFALNRKKFIQLLNAIDKPMFQPRNDKQVTLVLRNIDGWRVMFKMFAISLALSLIFTGLLPIFT<br>KTYKQRKFPYEAWYPFDSSKFPIYQLCYMYQMASASTLVVVILNVDTLVAAMNICIGLQCDLLCDNLRNLH<br>FDTSKSMNQKLIECIKHHKSIISFAEKFRQAFNWSIFLQFFISSTSLAIVMFKISRTTNYGSEYYRFISFACSVLVQ<br>VFIYCWFGNEVIVKSEKIPYALFECDWTPEPLEVKRSMIIFIIRTQRILKITVSYMFDLSLPTFLSILKTGWSYFAF

	MNQVTEVNTSK
MmedOR16	MLWSVFSYFTRADDVLAGIVIFSLGVSSEIGLVKLCFMYANIDKIQKITEGYLKSDAASARNSRFSKNILHTMQ<br>SVKKRGVIFWLVIISNGVVYLVKPIVTPGRHFMEDQFIILGLEPKYETPNYEIGFFMMAVGVCVTCYLPANITA<br>YLITVAGYSEAQFLALGHELANLWPDAQLHCRAMNLSQSVNEQANEYVKMRLRELVKIHSTNVNLLRDIE<br>GAFRGAIAVEFLLLIVGLIAELLGGLENTYMQVPFALIQVSVDCLTGQRVMDANLALERAVYDCRWEEFDAS<br>NRRVVLLLLQNAQKVATLSAGGIATLNFSCLMAVIKSIYSAYTTLRTTMK
MmedOR17	MMCEFDDPFIVLRTLLFINVSYKVTIFWSIFIIIFYTIEICLEIYYMLTNFNINLLIRYGPITTFFLLMIVTAVLPVIL<br>GKEIFEVLAFQCNTRWPLNMIRKDAQTRLKRKCQTINGCLLCILVILLSALGVNIPYFGTQRELLICVRVFEEY<br>FGEWAFIPYYLYLAGFPFLYYHFLRISFGFAYVFLEAQLQFFLIEEYLFETYQTDHEKHWKYLQDTRYQQQIGK<br>SLRDCIAHHNDLTKLVKMSVNVTVTAMPIFLLIGIILYISSFAFIINFADTMTNILKIRVFLFLASTMSITILFCWT<br>GQQLINVTSNIFFTLSGAPWYYWNLENVKILLTFLTNCTKNESIVLAGIRLDYRMFVSMCRISFSYALVLFKLR<br>KRSLVY
MmedOR18	MMDETYLQFFVKSFTYLNMLPEKTTFCTTIQQYYVSVIITITTFPILADLVSQFYEESISFTSVNENFVALSALFA<br>VIYVSVCFINRKHKIRALIADLALFETFSSKAVITETDKSVKFYTKLFIVYGIVGNLCYGLLPILGYKKCHESKSV<br>HMTRYGIPCGLVVRFLFPFKFDYSPLAELVALYEILVCILGTSVVIVVTTLICGVLIHITVQLQCLRKIILDLSQVN<br>DLEILEHKMKFCVKYHTAILDYGIRTDLAFNQMMLLHITWTGFIISVLGFEISTTDDYVEAFRFFMHLLGWL<br>GMLFVVCYYGQKILDESLAIADAVYTFLWYKKSVIVQRYVLLILLRSQKPLTLRACGVKVMSLATFLGVNIDI<br>NSVFFL
MmedOR19	MMDKLNYAPFITLRKIIFIEAKNCKLARFCDVLLIVLYSLAQCLNMYYMYQHFNLSLVIRYGPVLVLSLLVIVT<br>SVISVALEKEIFELHMVFRKIFWPLNCVGKNAQTKLTRKCQITNCWISCSLLLFLVTVISSFPCFGSQREFFICIE<br>VFEKYFGEWSFIPYYFYFAASPFLYYYFFRICYLFVYAILDAQVQYFLIEEYLFETFQTDDLKGWKYLQDAHYQ<br>QGIGKSLRLCIENHTALKKFMKMTLKFVLIGMSFFLVLGVLLLVSSFAFITNFADTMSNILKMRMLIFATSTVCI<br>TMVLCWTGQQLINVSSEIFLSLGGAPWYFWNRDNSKILLMFLTNCMICLNYELFLALVQLTVSYTLVLYNLH<br>KSGIA
MmedOR2	MMESTVTRLKRMYLWPTASVTSRKPAFFLITFSCFLLYGSVMHLIVNDISMEEVHVIETTAGQFGVLYYLTLFT<br>IYRKGILEIYADLSNFTKFGKPYNFDKRNKQLNQWSRWFSVVLYFFVISVFAWPGIFTQSCEDLNVALNKTEV<br>CGVVSPVWLPFRFDYKPMKQFVYFWQSFCCLYSNGGAGTISFAMSETIEHLILRVEDLKILFPKIVAERSPEVR<br>RKMLAKWVDYHLWLLSIGKLMNDTYRYSFSVIVLCAGTLFGCIGYTVMKNASTNFNSSFIFFGWMESVFVIC<br>VCGQRLMDAFHSVGTTVYNSEWCDTDVDFQKGVILITIRAQKPVRIYAGPFSYVSHLLILTVFQTSYSYINLL<br>NASS
MmedOR20	MMGNSTDLFLDRTKRILNFFAMWRSFEKPIPLKVYMAFIMTTQYLFLIFEIIYIVNVWGDMAEVSEASILLFTQ<br>ASVCYKMTAFISKTNNFVILLGLIESEIFSAQTELHEKILILKARKIKRLCMFFLVNAVTTCSLWAVIPLLDISSK<br>MLPFKIWMPVSTGESPHYELGYLYQMITIYISAXLFISVDSVPLSMIMFGCAQLEIIMDKIGKVKSWSLDQQPM<br>QKQEVLNSNYELLVECVRRYQSVVRFIELTEKTYHANIFFQLSGSVFIICNIGFRIAIVDSNSLQFYSMLTYLVT<br>MLSQLFQYCWCGHELTIRGEELRETLYQSPWHEQDIRFRKVLIITMERMKRPIIFKAGHYIPLSRPTFVAILRCS<br>YSYFAVLNRVRNE
MmedOR21	MMKFKVTGLVADLMPNIRLIQASGHFMLNYHADNSGALHTLRLGYCCMHLVFVLVQYGCNFVNLVLERG<br>DVNDLAANTITVLFFTHCVTKFVYFAVRSKLFYRTLGIWNQPNSHPLFVESNNRYHGIALKKMRRLLYIIIIW<br>TSFSAIAWTGITFVGDSVHNIKDPENENLTITEPIPRLLVKAWYPWDAMSGMPYYITLVFQIYYVFFSLAHANL<br>LDSLFCSWLIFACEQLQHLKEIMKPLMELSATLDTYVPKSADLFRAPSATSQDQLIENDYNEKNEDLKGVYS<br>TRQELGGHFRGGALQNFGSGGVGPNGLTKKQELMVRSAIKYWVERHKHVVRLVTAIGDAYGVALLLHML<br>TSTIMLTLLAYQATKITGVDKYAATVLGYLLFALAQVFHFCIFGNRLIEESSSVMEAAYSCHWYDGSEEAKTF<br>VQIVCQQCQKAMSISGAKFFTISLDLFASVLGAVVTYFMVLVQLK

MmedOR22	MMKTKHQGLVADLMPNIRLMQISGHFMFNYYGEGKKLMHKIYCSVHLFLILLQFGFVAINLVKEKEDVDD<br>LTANTITILFFLHTLIKIVYFAARSKLFYRTLAIWNNPNSHPLFAESNARYHSIALTKVRRLLFCVGAATVATTIS<br>WTTLTFFEDPHVERLNKETNETYIEEIPRLLVRSWYPFDARHGVAHIGMLIYQIYWLFICTVDANSIDVLFCS<br>WLLFACEQLQHLKAIMKPLMELSATLDTVVPNSGELFKAGSADHLRDNDGVPAEPAMNGDNMLDMDLR<br>GIYSNRQDFTATFRPTAGTQYNGGVGPNQLTKKQEMLVRSAIKYWVERHKHIVRLVTAIGDAYGVALLFHM<br>LITTITLTLLAYQATKVNGVNVYAASTIGYLLYSLGQVFLFCIFGNRLIEESSSVMEAAYSCHWYDGSEEAKTF<br>VQIVCQQCQKAMSISGAKFFTVSLDLFASVLGAVVTYFMVLVQLK
MmedOR23	MMPEFNDPFLVWRMIFTINFKKFKITKFCEIVLIVIYALVHCLLLYYTFTNFSANLLIRYGPVMIFYIFMIAATTF<br>SIALEAELSEVITFLDEICWPLNMIAEDAQVKLQRKCRIINMCIAFLVLIILSAIIVNYPFFGDQRDFFICVRVFEE<br>YFGEWSFIPYYFYFAASPFFYYNYFKLCFTFVYAVLEAGLQFFLIEGYLLQTYKVDYLKRWKCLKDNRYQQEL<br>GKSLRLCIVHHIALKKLVKMIVNLTVNGMPIFLLLGSLLYISCFTFTINLVNSLTNILKTRIILMGASCVGVTVLL<br>CWNGQQIIDVTSSIFTTLVGAPWYFWNLDNIKILLMFITNCTKNDKIVLAGICLDYKLFASILRISFSYALVLFN<br>LRKASVS
MmedOR24	MMPEFSDPFIMLRKMIFIKNHKIAKFCDFLIIAIYSSAFCLQIYYLCKNFSISLLIQYSPTLLCYIFVIDAAVLFFYV<br>EKNILEAITYYDEIGWSLSMIPKDAQTKLRKKCLIINICVSFILLLILSTLTINLPYFGSQRELFICIQIYEEYFGNW<br>AFVPHHFYFGVFPFIYYNSVKMWISFVYTILEAQLQFILVEEYLLESNIINDFKGWKHLHDIRYQQEIGKSLRL<br>CITQHIALKKLVKMIVNITMAAMPSFLVLGVLLLISSFAFILNFADTMTNILKIRVLMFVACIVCITTLLCWTGQ<br>QVIETTSDLFDSLVGAPWYLWNRENIQIFLMFLVNCTKNESLVLAGICLDYRLFVSMLRISVSYALVLFNLRKS<br>SIT
MmedOR25	MMRNEGETNYFQMCLTCYNLSGLRSSSKPFLKIISQFILYPVMWVLFGMMTYNIRFKHNNLSEITEVFIAVCT<br>TAYILLRKTILIKNSALYEDLIKEQSRFWKYDLFGEPTESKLRKNMQFCVSLIKLIVISGIMSTVVHCSSPFLVENI<br>DLPQSCWIPGNNSIVKNIIYVAESAIHIECLNYLAVFDGLYLLTTTNLKAQFVLLQKAIESITFKSGDEETYAWT<br>QLKACSQYHIYLLRIHKKINKIYSEFFLCTYILTIWGTCVPLFVIFNNISNFAEVVESMFIAFLINALLVMMFIPA<br>SEIEIEAEKLALQIYFINWYETKNLKIRKFILFWLMQAQVPVRMTGGGMLIINRSLMLQIQRIGFSLTTLLTGLT<br>T
MmedOR26	MMSDEYVKDVFIANRWMLRCAGLWTPSTRSKLVQIPYKIYAIVVFLFVNVYFTSTEFLSLFYTHKNLYNFIKN<br>VNFFLTHFMGAVKVIFWFFKGHVLRDLMRTLESPEFHYEPCEGFQPGLIWRKYRRIGFKYSLGFLALAHMTL<br>SSSYIPPLLTVTLNPPPYQNGTLSPFYQKLPYFSWMPFSYSTPRSYLLALGYQAGPMFSYAYSIVGMDTLFMNI<br>MNFIAAHLVILQGAFASSKMRVLDPGQMNNEMKRNCRHLQTILRVSEDLERVHRYLTLGQLTATLFILCTSL<br>YLISTTPASSKQFYAELVYMVAMGFQLYLYCWFGNEVTLMASEIPVNVWKADWYDCDQSFKKSMIFTMTR<br>MQKPIYMTVGKFAPLTLQTFVYILRTSYSIFAVIKNTSI
MmedOR27	MMSEFNDPFIVWRMIFMINFKKHKITKFCEIVLIVIFSLIHCLLLYYMFTNFSVNLLIRYGPTTLFGIFIIAVTIFSV<br>ALEKELSGGIDILDEICWPFNMIGKEAQLKLERKCRMRNMCTAFVVLIILTTIIVSYPCFGDQRDFFICIKVFEE<br>YFGEWWSIPYYFYFITIPFFCYNYHKLCFTFVYAVLETELQFFLIEEYLLETFKMGYLKRWKYLENTQYQQELG<br>KSLRFTIAHHNALKKMVKAIVNVTVNGMPLFLLLGFLLYISCFTFVINLADSMTNILKIRIFVCGASCVSVTVL<br>LCWNGQQIIDVTNSIFSTLTGAPWYFWDVDNVKILLIFITNCTKNDSITMAGICLDYKLFASLLRISFSYALVLF<br>NLRKSSLS
MmedOR28	MMSEFNDPIIVWRMIFMINFKKHKITKFCEIVLIEIFSLIHCLLLYYIFTNFSVNLLIRYGPTTLFGIFIIAVTIFSVA<br>LKKKLSGAIDILDEICWPLNMIGKEAQLKLERKCRMRNMCTAFVVLINLTVIIVNYPCFGDQRDFFICIKVFE<br>EYFGEWWSIPYYFYFITIPFFCYNYHKLCFTFVYAVLETELQFFLIEEYLLETFKMGYLKRWKYLENTQYQQEL<br>GKSLRFTIAHHIALKKMVKVIVNVTVNGMPLFLLLGFLLYISCFTFVINLADSMTNILKIRIFVCGASCVSVTV<br>LLCWNGQQIIDVTNSIFSTLIGAPWYFWNLDNIKILLMFITNCTKNDCIVLAGICLDYKLFVSIVQISISNALVL<br>FKIRNASAS

MmedOR29	MMTKVKTQGLVTDLMPCIRLLQAAGHFLFNYHADTSGMNMLLRKIYSSAHAVLIVVHYICMGINMAQYKDEVNELTANTITVLFFAHSIIKLAFFAFNSKSFYRTLAVWNQSNSHPLFTESDARYHQISLSKMRRLLYFICGMTVFSVISWVTLTFFGESVRMIASKETNETLTEPAPRLPLKAWYPFKTMSGGGYVFAFIYQIYFLLFSMALANLLDVIFCSWLIFACEQLQHLKAIMKPLMELSAALDTYRPNTAELFRVSSTDKTEKVPDAVDMDIRGIYSTQQDFGMTLRGAGGKLQNFNAENNPNGLTAKQEMLARSAIKYWVERHKHVVRLVASIGDTYGTALLFHMLVSTITLTLLAYQATKINGINVYAFSTIGYLVYTLGQVFHFCIFGNRLIEESSSVMEAAYSCQWYDGSEEAKTFVQIVCQQCQKAMTISGAKFFNVSLDLFASVLGAVVTYFMVLIQLK
MmedOR3	MMTLVYQTDIFKPNVFFWKMFGIWADRKSSKTYKYYSFVFLFITLIMYNSLLAINLLYTPLKIELLIREVIFCFTEITVSTKVLMILFKRNKILDAFDLLNKNEFRGNSEESSAIIQKNNSAYKTYWKLYAILSNFAYSSQVLGPLIVKLIWKTKLELPICNYYFLNEELRHDFFSGWYIYQSFGMYGHMMYNVNIDTFISGLLMMAVTQLKIIQTKLLSLKLNPRERKMDRGLMNITEVLKLNEILKHYELVLKYCSTVQSILDVAMFVQFGVASAIICVAMCGLIMVRSSTETLLFMVTYLFAMTLQIFVPAWMGTQLHFQSQELVFAAYNSEWIPRCQSFKRSIIIFVERAKIPITITGLKMFPLSLATFTSIMKTAYSFFTLIRNMQALQEE
MmedOR30	MNAYVCAMGALLSVKVAAFLWHRERLWQLACQLVSCWRQFEDADGGVRDMYRSQAARVVRYMQVMAAIPAMMWILEPLFSGGDEQSQGRSLPLPTWLPLTLQQSPTYEILYVLQVLIIIIAVAVSVYVNIFFAVLMLSIAAELHVLNNNMMAMGQCRDCEVPVRYEREEGRSRDQVTSSFLRTHRRQRVPKAAANKSLPSPVVVHMHKGSYDRMYHQLVKNIRHHQVILRSVEELQKAMTHSIFVLLFLNIFNICVVIFAGTTLLQKQADQVAMYKMLCSIPIYMYETGFFCVVGQTIIDQGERLSMSAFASTWLDGPRRLHRLLLVFMLRCARPPTITVGKTYTLSKRTFVRILNGSYTMFNMLYQFQRNK
MmedOR31	MNCENQFAKDDYLKTLKIMASEVFQSKAVKVILIFVFLVHAIANLLTIYFVLYVSDTKLFVNYASVFFSEFYPMLAILTVIFKGQIVQHLTDEFKIWAIDSASKKLQSEIKLKIKIITAFVITNSLIAVWGGFLYVQPLSEDENLYFALSFIHQYFPNQSSTLEFFYRMTYPILGYLMTVHAYQCLYYTQHINFQLRMFTEVVAEFAPVKRFLLFEHHLFYNKKYQTEIEQRLKFCIKRSQEFVQICVIKNSEIGSFIPEFAICGLLFGIGVTFFLSTGKFTSEYYLRMGVTSFGGVMTFSALIWSGQTTETMTSELVKALNEVRWYNFNQSNKKLYLTLVMNIMKERKIKFTENYSMNYRLGLAIVRNIYSVISVVVSKRRH
MmedOR32	MNDIGGLALAKSGLNNMMSILGGFRGPREVRFKGTIYEHIFIAYSYFGLLVSHYHVICCYLTPIFMPDMSFKDAMFFAVPCITTTFSHLRIYYMAWNRSKFIQLLEMNEEASKDDYYEDELQKEIDGWAKQVRILQPILYFAVSAPIVPWGVTPIVNEVLGNPWGPRKAPIISWYPYNVQETHFWVFTIFIQTMAGCHATLSNVMFDAVFICISTRQLALLIHLKNSFSKIFQVIHVDPKGISWYTNYRAEAVEKEEIENDLTQRLKYGIRKHQTTLRLSKTIVFFLATRFWIICLIYELHMYLFFMEVVQVRKS
MmedOR33	MNDSGYQSNLSLLRVFLDEFRSVLRQESPGLIPRLAFYYVRAFLSLLCQYPNKKLASLPLYRWINLFIMCNVMTIFWTMFVALPESKNVIEMGDDLVWISGMALVFTKIFYMHLRCDEIDELISDFEYYNRELRPHNIDEEVLGWQRLCYVIESGLYINCFCLVNFFSAAIFLQPLLGEGKLPFHSVYPFQWHRLDLHPYTFWFLYIWQSLTSQHNLMSILMVDMVGISTFLQTALNLKLLCIEIRKLGDMEVSDKRFHEEFCRVVRFHQHIIKLVGKANRAFNGAFNAQLMASFSLISISTFETMAAAAVDPKMAAKFVLLMLVAFIQLSLWCVSGTLVYTQSVEVAQAAFDINDWHTKSPGIQRDISFVILRAQKPLMYVAEPFLPFTLGTYMLVLKNCYRLLALMQESM
MmedOR34	MNFFQKKLAKGDFFKTLKFIASDVFQSKAVKMVLILLFLIHAIIYLLTIYFLLYVLEPKQFVNYATVFFAEFYPMLAILTVILKGKIIENLTDEIKIWAIENASKNLQSEINLKIKIITTFVIVNTLIAVSGGFLYMHPLPEDVNLFFALRLIRDYFPNHYTSLEFFYRMSFPIFAYLMTTHANQFLYYTQHINFQIKMFREVCLEVKAWKTVSPFENHLFYNKKYQTEIEQRLKFCIKRSQEFVKISVYKNKEIASFIPGFAICGLLLGVGLVFFLSNGKITWEYYLRMGFTSLGGVTTFLALVWTGQTTENITSDIERAINEIRWYNFNQSNKKMYLILVMNTMRERKIKFTEKYSVNYRLGLAIVRGIYSVISVVLSKYQH
MmedOR35	MNFNDEKNYIFNLRLMKITGFYQLIYPSAPKCFGFNAYKVAAAIEVMTGVLSVSFLFSSSYYYLDNTNELMSHFMLVVAIFFSTFKIFWVSRNSKTIWNNLDMTSINFLSYTGHKKEILQNARAKSISTTILFVILWSSVTVAWCIS

	PFFVKDVYLNVKFKDDEIRRFRYNSLNYVYPISGESYNEHFLYFYVVEMLQVVFWGHGTVAYDTFVISICISIA<br>FQLKTIAVSYISLNDRKGDVKNLKDNDLEAIFNLKLLIQDQQNMFKKIKEIYKIFQPVTYVQLAAQSMLIILQ<br>AYMIFINHYNGFSLLSVPIIKLVVTVAPNIIHLFITCYLYSDINYQKDSMNFALYSSDWTAMSISYKKMLLFTMR<br>MNDAEKLKLKISLRKIVNLEMFASVMHLTYSIISVLAKSYGNTNTK
MmedOR36	MNFNFFDKSDDFSMPVVYFVAEGIFHIKFIRFLAYLALISNALGLLLMLYQFVLDAESLYIIKYGPVLSGAIFAL<br>VSLHAILFMRDLETFKQEFDCWSEHDASQETQNRIKQHINSVTIFVIFNSVLAFVAGVSLVLPSKDEVHYHYF<br>IKILLELEVVPRGVTRTVYYLYKIDYVVMYPILTINSVRMLYFSRKFKFQVKLLVDRIVAMTKDNVDDLSLFYST<br>PYQNDMARKLKTFVRRHSYIAQYVAKINKSIGPFVVTFSLSATLMGISVLLIAAAGTFYYNKYQIILCGAMYL<br>CTLCSAIDATETVEMESIEIYNALLAQPWYIWNNENKKVLIIFLMNCEKPIQITKFSDVFYYNYDWGISVLRK<br>AYSLGSVFFNLRQYIGK
MmedOR37	MNGNGAVLNGVTESSMPIKRPRKLKKVPNKRSIQFETDRNEKKVEKFLSDEEALKKGFDENQGIYLVLGTLY<br>RTSLWSWVHTVLFSLTALFMMVCLGRVAVLISDDFSLLFETIHYITIIGGVLVILPPMMRDEFRFEKIFKTFGRN<br>VYSYDMLDEETAQSVQKLRAQGNREKQLLTKAFTVMLLGTFAGFSVLLPGMYIINGKFFERQRDDGIIMGIP<br>CVLWFPARVGDDWIIFVRVFLLLIEEYAAFTVVAFIIGQQTSAICIGHTLLYEFKVLSLTMEKFVRRATKLAEGK<br>KFEGIRINSEKQLYEKLTACLKDSVKHHDILLDVSEQYKSIFYVPELVILLSSTMVICLSAISLTSDDIPLEAKALS<br>LILTGAEMVNVFVNCYYGQVLLDAHDELGDAIYGSGWTSCSTTVRQHILIILSRVQRPLSLSAGGFAAVNLD<br>TFAQVVKSSFSYFSLLQALKE
MmedOR38	MNIKFLDKSGGFSMAVVYFVAEGVFHIKFIRLYTYSALISNSLGFLFMLYQFIVGAESLYIIKYGPVLTGCTYVLV<br>SLWGILFLRKTEEFKQEFHFWSEHNASKEIQNRIKQHINSVTVYYVILNIVLAFTAGTSLILPNKDEIHYHYFIKK<br>LSELDTIPRGINETCYYFYKINFVLMFPIMTVNSNRLLYFSRKFNFQVKLLVERIETMAKDYNVNDPNLFYNV<br>RYQNDVKQKLKIFIRRQAYIAQYVAKMNKFLAPFIIMFAISATLLGISVLLLLVTATIYYNKYQLILCGAIYISTL<br>FCAIEATETVEMESVEIYNALLAQPWYSWNNANRKTFIIFLKNCEKPIQITKFSDIFYFNYDWGISVFKKVYSL<br>GSVFFNLRQYIDK
MmedOR39	MNKFHDPFIMLRKLIFIEARNCKLARFCEILLIVLYSLAHCLQIYYMYQHFNLNLLITCGPIMGTVLLTIVTAV<br>MSVGLEKKIFEALTVLSNISWPLNFLKKDSHTKLTRKCQVIKWCISCSVLLSVITLISTFPCFGSQRDFFLYVEVF<br>EEYFGEWSFIPYYFYFAASPFLCYHFLRVTFVFVYAFLHVQLQYLLIEEFLFETYRTDDLKGWRYLQDIRYQQK<br>IGRSLRLCITHHVALKMFVKKTVDLVMMAMPFFLVLGVLLLISVFTFIINFAVTMSIISKIRILLFAATGVCITMT<br>FCWSGQQLINVTDEIFWVLARAPFYFWNRENSVILLTLLTNCTNNDSVVLAGICLDYKLFLSVVKLAVSYSLV<br>LFKLRKSSLV
MmedOR4	MNKNHYILKTYCDKIFLVGSGNFWHQKTESRNDKTLLYKIYSCVLFFTYGFMTVLEIMAATMGDFPEDEKR<br>DSVTFATSHTVVMIKFISIIKNKELLKTLNRKMMMICEAHEEQTLMDEMYRTVKINVVAYCVAVYGSATFYV<br>FEGLRKFYNGSHFVTIVTYYPSNDDDTMLASIVRIATTLVLLMMLLSMIISVDTYTMAYLIMYKYKFITLRHYF<br>KRLRENVDELVAAGKARLAAEKLAQGLVEGIKMHNELLSLSKDIHKAFGTVMALQLCQSSGSAVSLLLQIA<br>LSDQLTFTMGMKIFFFLAAMYLLLALFLCNAGEITYQASLLSDEIFYCGWHKCNSPVLSTQRNIRDIVLIAILR<br>AQSPLVMKAFKMVELTYATFILVVRSTYSVFALFYAQNK
MmedOR40	MNKNMNKNHYILKTYCDKIFLVGSGNFWYQKTESRNDKTLLYKIYSCVLFFTYGFMTVLEIMAAMMGDFP<br>EDEKRDSVTFATSHTVVMIKFISIIKNKELLKTLNRKMMMICEAHEEQTLMDEMYRTVKINVVAYCVAVYGS<br>ATFYVFEGLRKFYNGSHFVTIVTYYPSNDDDTLAATIVRIATTLVLLMMLLTMIISVDTYTMAYLIMYKYKFIT<br>LRHYFKRLRENVDELVAAGKARLAAEKLAQGLVEGIKMHNELLSLSKDIDKAFGTVMALQLCQSSGSAVSL<br>LLQIAVTMYLLLALFLCNAGEITYQASLLSDEIFYCGWHKCNSPVLSTQRNIRDIVLIAILRAQSPLVMKAFK<br>MVVRSTYSVFALFYAQNK
MmedOR41	MNKQKIYDVNFTLFKLIGVYQMVDPNTQKIFGFNVYHFVNIVFITFTTIMTILGLSGFFYKVQNNNYNTSEV<br>DIIFIIFSTVCITIGNLKIIIIIFKARQLWNMMEITDKSFLSNTFYRRNYHKILKCGDLLSKFFNLYFSFVLITLTSYA<br>IVPIVLNARSIDATQNTETIQKMNIINLRYPFTTEIYNTFFKIFYASECIILFYTGFGVFALDLFSMTLLMVISYQY

	KLLASAFEVLEYRMDNKDDSILSDEKLLETFISIVSDSQIIHKKLKMLYDIIRPIGLIQLMADALGMICMPYLIV<br>VYFVKYGSLFNPETLKFVFTLGIAGVQSYMYCSLFQRVNDRRDGVNFGLYCCDWPGMNIHMKKMILFTMQ<br>MNSSNKLNMNITTHKAINLPLLSTIIRLSYRISSVLINSNIN
MmedOR42	MNLENLFNGGPVALNLSMYKQLGYYQLLDPKGPHIYGYHLYRTILKIFLLIVQFIAIFGVMGFFIKIEDTDTDP<br>GKSNSFELIIILTNCSLSSLKMYTLVSNSKIIWDLFDLTRIDFLRCSRHSKLIKANFMKRCKKSTTITKWIARSFLV<br>GLILWVMGPFIANEEYTEPNVVRRHQNIINIKFPVTVKTYNNYYFVFYFMEVAVGFCIVYGSVLIDAFLMSFC<br>WIISAQYQSVTKAFATFGYNKQGSPKEIYKDFKSIIIDHQNVYLKMKSFYAVVRPITLIHVFAYSCSLIMYAYVIV<br>TIFHSKESFIIAEIMKIVMTVSNVTIEVFIFCYLFELIDNKKEDVNFGLYSCNWTGMDIKFKQLLLMSMKMNN<br>ANRLKLKATPDVTINRPFFANV
MmedOR43	MNLIGLPPREDDRKAVDYRKCTFFKILYTENETGGKVSMARGVFVIVTLSIMATCCLVSVTRSQTAEQLLDNL<br>KGMHLEVMVLMVAINECVSRKRMRRFMSYVERFRANPRYDLPGEEAILVQARSNAIRDITFLVIIFGANFPL<br>MMLTKPVTEALGGGSWKQLPFPWTVLPDDEDTTFVAFLLLHTLGVFFSHCLGVVGMCFSTITTQITALFDVL<br>LLSIERIEERAARKMKQLGLSYQESMLICLKESVAHHQELVQEVRSEKPHLESQFFSEIVNISTIMACEAFPLVR<br>PNLTISIAIKGLVFLVVQVLCTAVLCDRMEIMADQNTEVFAALYNTPWYKCGVEYRRILLNGMTFCRHPLTIR<br>GKSFLGLIATRATFYTAMVNTFNLLSMIRKMG
MmedOR44	MNLQKLDPLEGFKPTISMLKIFSVWNSSNMFYKIYKNVTTLSLAITYTCVMICVVVNFNVSEINENFYYIPALS<br>TAPFKLVIFQKSFKKIQNLLFLLQSQYTKIRSEKQAKMVEDSVVLSKRVVKVFAVLVVPTCVGLFGMPLLKDEI<br>KLPLIIWIPFDYHEPVVFGLVYFVISFSGSFTAYINIGTDTFFYNCLIQIETQCNILSDTLRNLHEFGRFEAEIHTIL<br>IECIEQYKTILKFTKILSKTYQGILSVQFICSLLSLCLTMYRMSLADPGSEEFLRYFVFQWGVLPEIFLYCYFGHR<br>VLDSTKNLYYSTYELQWYNTSAKFKTNLLIFMGQIQNPIVIYVAGIFSLDLETFKKIMQKAWSFFTALRNIHEQ
MmedOR45	MNLSQSVNEQANEYVKMRLERISKIHSPMLPFEDIQDFRELCCIPLAVYAVTGSITASYVYAFLISLLWFLFARC<br>TDPEDFQVAMVVFSLGISSEIGSTKFFNSIIYIKELRKLFKDYLLYDATCPAQGRLRLHLLTTLRYVKRRAIIYWL<br>VIIGNGFIFAIKPLLVEGRHLAQDDLVLIGLEPMRQSPNYEIAYAIMTMGVCFICYPPAHVTMFLIIIVGYTEAQ<br>MLALSEELKHLWNDAIEHYEKHSRTEREADAAMKSKILNSFVNFRLVQIIKSHSTNVNLIGRVENVFRGSLA<br>VGYVFLIVGLIAELLGGLENTYLQVPFALIQVAIDCFIGQRVNDANIDFEKAVYDCKWENFDKRNMKIVLLL<br>LQNAQKTVSLSAGGIAKLNFSCFMSVIKSIYSAYTTLRTTMK
MmedOR46	MNPSEKNYAFDLILFKTIGYYQMVDPNTKKIFGFNIYNVINITLVIFTSIMTLIGLSGFLYKVDSIAYEENSFQNI<br>QMLFYLSCISLGNLKIAITVYNADAIWKLLNVAHESFISNKYCKQDKYKLNISGKQFVRIFPWYFFLFFMTAV<br>SWSIVPIVVNNQVESKETQNNENIYMTNVANLRYPITVKTYNTYYKSFYALEFILVFYCAYGLVVFDLFIVALL<br>QLLATHYEIISSAYENFKYKAENKNGKLRNTEIQKELISIILDCQTIYKKLETLYGISRPIVLVYMVGDAIGMITM<br>PFLIVMSYVQSGSSILNTNVFAFSWTLFVVGIQSYMYCSLLQNVNEKKEDVNFGLYCCDWTSLDIEIKKLILLA<br>MRVNSSNNLTMKVTYTKFIDLPMFATIVRSSYSVTSVLINSNIHKISK
MmedOR47	MNQPQESFLKNDYLKVLKLISSDVFEPRLVRAILFVVFAVQLTASIITVRALLIKELTAKEFVLYGPVFFGCFYG<br>MLAIYIIIFQSSFITNMSQELEMWSYSSGGEEINRRVKFQSRVITIYALVNFLLAIVASYLYFSPLDSDNETFYMV<br>RFIEEKIPDYAKICKIAYRTTFLAMGYVMIVHSYQVIYASQHVRFQIIFFTEYVKKVVEFDEKISEECLFYNERFQ<br>TIVGKRLQNCVIRHIQFLKFDRIKIKEMSNLIAAFSLCGCLLGISISFYVLSGIFYREHFLRVALISVTAVSTFFALI<br>LAGQSMESKANSAHIIMNNIKWYNFNQSNKKAYLLLLMMSMKQYKIKFSENYSINYELGLTIVRGIYSIISVM<br>ANMHFDN
MmedOR48	MNSLESNEVAINLKLFKLFRFYHIFYPYSEKLCNFNVYHLAWYIINCVIGGIIIYGLLGHFTEMEDDIDIIFYIQI<br>LFCYLLYSLSLLKIITFLYKANDIWDLLSVTRINFLTSTQCQAHIGILHKHRSKSIQITNVISGFGIMTTLEWILFP<br>LVLWLLPKTNANGSNQRFENIFNFRFPVTISEYNNNYFIFYIMESSIAVFMLYVYVVIDVFFISVCYVIIAQYEIIK<br>RAYESVNCEQSPKNNNENKNNCNLNDCYDDLISILRDQQKHYAKLKLFYSTYKLIIVSTVVINSGSIIILTYAS<br>VVIFISSETIPIFSVAKLISAFAYMFFVLFFLCYLMERVNNKIESVQLGMYSCNWTAMSIKTKKLLLLSMRMHN<br>ANKLMIKTTPNKIINLQLFNSVIFDSIL

MmedOR49	MNTEVTERFEYASKAYWGALGFTGLDAFLYEKPPKHNVLRWYAFRIIYIYFVFVHYPIFITMQFWGIVTAESH<br>TLMQISFDISLMGYNIQNIIKLLIWMIRIKTVRSLRLNFSKFNVNKYRPKLSSWIIKRDAENALKFTGRCYWISY<br>ANLLFWVVLPTATAIINYSTYLAGYADWQENDFPRYSNTRFPFDLSQHRSQVIVSFLEVILFTLGFMSFQSMD<br>MFFSVIIRMAQAQFSVLNSALFALDGELDKFWGTEVPVNDCKPPMKIHLIVQDHQRMIRYGVRLRKFLSPIL<br>GLETLNCITIICNMTIVASSQVSGGGEFLEVALAAFASSLVVITCLVVFFTFTSMTGQLKDAEESVFYAMYSSK<br>WYERDVSHRKSIILMQKQAMTSRRIKMFGLGDMGRSTFIDGLRMVYTYYNFMQRFK
MmedOR5	MNTIDEAVRANRLRWWGHVTRMGEAMLSKRLMSSAAEGPWLETSCQHWCRSRLFGAYTLFAFSLGLWQT<br>AESLLSCYFSRGRMEQATMVLMTTFNIGGSTVKMALLALQRRRYFSLVRRTDLLVTEGAPPADCSKERPRSLP<br>GPVAAAQXAAHGRHAAADRGAHPWDNNRRFYALSYALQCMGAAWTTQASFGVDCLFVTVLMLVVAQL<br>EALASRVRAIRVEPEVGGGAADKAADQMYAELCACVDLHRKILSFVKQLESTMSPIVITQFAISVLVACVSLF<br>PATYSTNFTDVLLCAGFLPVPLGELYLYCWAAHNLTQQAEAVSAAAYSCSWVEASERFKRALRIIISRAQKPL<br>VLTAGRLYPINRAAFVSLVNASYSYYALLGQINSRSTETTH
MmedOR50	MNYKKQFAKDDRLKTLKLMASDVFQSKTVKIILTVVFLVHFIANSLTIYFVLYVFETKLFINYASVFFSEFYPML<br>AILTIIFKGDVVQNLTDEITFWTIDSASKNLQHEIKLKIKFLTAFVIINSFTVVMGSFSYVQQLSDDVNLFLAIRL<br>IRDYFPNYSTILEFFYRMTYPICGYLMAVHAYQCLYYTQHINFQLQMFTEVITELNNSKTSSLLENHLFYNRTY<br>QTNTEQRLKFCIKRSQEFIKICVTKNKEIGSLIPGFAICGLFLGIGITFFLSTGTFTTEYYLRMGVTSICGATTFSA<br>LIWSAQTTETMTSDLVMVINEVNWYNFNQTNKKLYLTFLMNTMKERKIKFTENYSVNYQLGLAIVRGIYSVI<br>SVVASKRQH
MmedOR51	MPAAAPWSESALWLNARVLALGAMWRPPGCRGPALSACWALYTGWMLFTQLSFLVAQARALWHFWGD<br>VGKVTHDVCLMVTVVLGLIKFGVFSLRKDDFFRIVRQIDSARSEQSRSDDAEIASILRASYRSARNVTLYMTLL<br>GGSSPAVWAVTPALMRKLRVGPPERELPATAWYSGRDTDSPRYELLCVLQLFSMQYSFFAAIGLDLFFVSIIIHI<br>AAQLQVLGVRLRGIGKLGHKQSSSLSNSPILLRDVEDGFYNEKSLWMEFCSCIQQHHSIIELVKEVESLLNIIIL<br>LQFLGATVVICVTLFQSSTNTSNFMTLLKLQAYLMVIIYEIFIYCWYADDILYQVNSFTSGIYFTVRLQDLDASQ<br>VLQGIREWDAVVEDKRGGGEPDTSSRLSAAAAGKFYYISRATFVRLISASYSYYALLNQMNDK
MmedOR52	MPEELFLDRSIKKIESYFRWMGINIRSGDNNNKKDVFKIRCIYFINFVLLNTDVLGAIFWFRSGLEQGKTFTEV<br>TYNAPCLTFSFLANFKMLSLIFYEKTVHELIAALQKLEIKHFLRQNCAEELKMLKDEKNFLHAVFKGSKIVNY<br>ASILTFGCSPLVLIASNYYKTGRMDYLLPLIVLYPFDVDNITVWPIIYVRQIWSVITAVIGVCATDYLFYTFCVYI<br>STQFRLLGHSIERVVPNNGLSVRTRLNGNLRMKFVENLKWHQELIRAASLLEQIYTKSTLYNFVTSSVIICLTG<br>FNVAVVEDFAVILSFLFFLFMSLLQIILLCFFGDKLMKSSTNISDAVYNSKWYLTEKNVGKVLLMVQIRSQRAC<br>RLTAYGFAEVNLRAFMKILSTAWSYFALLQSLYSSHE
MmedOR53	MPESIKLFSEESSTFRTMAEFHDPFIMMRKILYNKSKNYNLAKLCHVLLIIIYSSVQCLQIYYVFKNFSINLLLRY<br>APTILFFIFIITGAVLSLIFMENDILEIVTFLDKICWSFNMVRKDAQMKLKRKCQVINMCILFVLLLLLSTLTINA<br>PCFGSQREVFIFIQIFEEYFGEWSFIPYYFYFTAFPFLYYNFLKLWMSFVYMVLEAQLQFILVEEFLFETNQVNRL<br>KGWKYLHDTGYQKKIEKSLRLCIIHHNALKKYVRMTLNVTLKAMPFFLILGILLLISMFAFLINFADTITTMS<br>NILKMRIIMTVTVTMCIAALLCWLGQQLIDTTSDIFASLVGAPWYFWNLGNIRILLMFLTNCTKNESVVLAGI<br>CVDYKLLVSMLRTSVSYALVLFNLRKSSVV
MmedOR6	MPFEWTIRKNKIKPILQNDVLLNLMLVPNTIISNKFLVILNYFYFGFIILQSVFVAVIIITKDEWKLLNGQYAGY<br>TSGCAIVWSSYITMYTYVDKFLNLYKEIFPHLWSLDVVGQDHFNKFSKMAKVLKLGKNILLVVGFLSATVGL<br>PWYRDEYEIIITVRVYKDYVDKWTTLLYFVLFSSLYHIALTVIFCVLCLVYMVLHLHNQCVMLNKRLEALDD<br>EQLFLDNDNYQDFVTKELKFCIQQHQFLLKFAKRLNDILYYPTFYYVLSGVVTGVSLLLFPKNDIKNLLRCVL<br>IIVLGGGFAISFCFLGQILENASEELLFSAYSARWYLWNIKNRKLLSVFLLKTQDNIVLSSSGIITINFRLLISLYQS<br>IYSCLTFLLNIK
MmedOR7	MPHIDTINMFLQMTGCTDNKKMLYLTYFEFLITFYYLIAAYISILHFEESVTIQLFTLLCMLIECVILLNIVFRLY<br>HQNHIREMLQYSRRSGIPDSYQSIINLITNYHLIASNMFVIFPATYTILHDSVRVGDPFTFPFLDVLPIQTGNLAI

	YACKYMVYAISVYIAHIELCFINTTFIYYVGVLKQRLDTIVQTIQEAVVDDDEQKFKYAIIKHQKLLTYFNTMK<br>IVFAKPILLSMSFNAIYFGLTTSFVIQAIRGYINQAILSICIASSAAAVINITIYTFYGSVLLDLQDEILHVLFDNAY<br>FYVNKSFKSSILIMMTRVTIPLNFTVGYIFTINLNLLLKIVKMSYTVLNVLLSSETIKPHKLS
MmedOR8	MPIWSPVDIYSSPTYEFIYLLQSFASLITSQCCLSIDIFFVHMMLMVAAELDVLNYNLSAMKHYDSQTPISDGE<br>EFISNVKTSGRRLELPSSDKSFGEQALKGDIEGNGLHQLLLKNVLHHQAILRSVSLLQSAMNVSIFVLLFINM<br>ANLCSSLFVAAVLLQRDGNAAKALHALLCVPALTYETTIYCTYAHIMTDQSERLMYSAFSCGWVNSDARFK<br>RSLVIFMMVTVRPIEITVGKMCTLSKQMLLQVLNGTYALLNMLYHFH
MmedOR9	MPKKFRSDDISADSLRLLWLGHMHPFFPFRRSFAFLIINLTACFLMIALAIKGITISYNNDIFFVAECLQTCNLM<br>LHGVGKFLNLYFHRNGLQALLENRSKFWKIDDFKCENIYEDLSGITSTVKRGLRYYYCGALVVIFLFDLQPFA<br>TGLLPTGCYVPEGWFKGLTLTLWLLSISFFLIIQSTDGFFCSLSVAIVIQFKLLSHRFKNMHLLYAESERKMWKE<br>LKGLVDYHNFLTNYCKQLNAAFAPIFLLQFLVSIVSASVSIFIFMQPGAWSNRIKFVLYYLAIMVETSFYCVPA<br>EIIVNAASEIGNAVSDLDWYKIKINKVKKCFIIILARTQKTMVFTGYGLVNMNLQTFVIYVMTVFSFYTYLNSV<br>RKI
MmedORco	MPNVTNKRQKRLFSKTRTKSEDPFVMIKDVFVDGGYHPVTKMLNYICLVIHSCSLLLELNYFVHNYHFDLM<br>MKYCCAMSLMGYIIATMLFAIFQEHSAIDLTKDILSLFWPIDYCGPRVKEEIVKKATKINRIHYIVLLFAGALGI<br>TMFPIWGDQKEWFLCVQVYQHYFGKWSKIPYYVYFFTYPMLAFSSVRLPFMTMYAIVQIRMQVYLLHQHIS<br>EISGEYVYDMKNLQILCDQNYQNEIYDKMRLIISHHIMLKRWMRKLVHTVQISMPVFVLLGTMTSISVLFYAI<br>YSFHNINFILKVRLISVSVCTVLVVYMFSEAGQALSTETTGVFDLLMTCPWYVWNIKNRRILLIFMANSLEPM<br>TFSLAGVTLDYRFALGMLRTSCSYSLILYKLKTGI
MperOR10	MPPSLPPEAAAASSDLGYLLTLLHWTAVLRHPRFIGTSPFWFRVYTLTLLTIDASFVFSVFIVLFREGTEDLDVF<br>TLTLSVADTNGTWLFRLAHTVACEAAFHKLSQQVGHDFAEFLTWDDIPLLTSQSRVVRRFSKLYMWGGVG<br>ACAYFLVSPVCPEGLPYILALPFDAMQPLGFAVTWVFCSVVTLHAVVMTMVLDSFNVSVIAQLRIQLKLLST<br>KIVNLSKEILNTDMESSEANVYQELYYRLEKCIRHHEAIIKNADLLERSLGTMLLAQSISIGASTCFQMFQLAT<br>RANGLQQAGKFGCYLFAMLAELFVYCWFGDDLITESENVALAAYEAVTSLQGCPLSIKRSLLLLMHRAQRP<br>LRITARGFFPLCRESFVSVVNVSYSFFAILRNFKEEEQQPD
MperOR17	MPPSLPPEAAAASSDLGYLLTLLHWTAVLRHPRFIGTSPFWFRVYTLTLLTIDASFVFSVFIVLFREGTEDLDVF<br>TLTLSVADTNGTWLFRLAHTVACEAAFHKLSQQVGHDFAEFLTWEDIPVMRAKSRRVRRFTLGVVWFGVS<br>ACSYFLVSPVSPEGLPFILALPFDATTPLGFAVSWLFCTITCMHAVVMTMALDSFNVSLISQLRVQLMLLNSKL<br>VTLAKEESENSKLSSKTTDYRELHYRLVECIRHHQAIIKNADLLESSLGAMLLGQSISIGASACFQMFQCVTSG<br>NGLQQTGKYGCYLALMLAELFVYCYFGDDLITESENLALAAYDAATRLQGCPLSIQRLLLLLMQRAQRPLRI<br>TAGGFFSLSRESFVSVVNVSYSFSAILRNFKE
MperOR2	MPSIIDISFKFNLKVLRISGLYLPEKFKSLYQIYSYLAYFLLVIPVPILECTNLLVQEKITFRQIADSAFLIAELGCFI<br>PKNWPFVRHADRLKRCIHYFSAPIFKTKRKEHEEILQDCIKVCHRSTAFYFASVTVGFFSWAIRPISWKNHIFP<br>TDIWLPFDPHTASKVQVAGVYFYLVLGKGFSSKVLKFWFNFSCGIKILKNNLQFLGEYVDEELATQMHSLSPS<br>EKTQLTYQKIRQCVIHHKHILAFVKEYEECFSQVAFSQFIGSVVIFCVSCLQLTIVDVVSLDFLAMMMYLIATL<br>SEVYLYCHFGTVLYHESNTISEAIYLSKWYEFDIKSKKALSILMERLKRPMIIKCGKILDMSFITFTMILRRSYSLL<br>AVMENYNIELN
MperOR20	MPSIIDISFKININVLCLAGLYLPDKFKSLYRVYTYLVYVFIVIPVPTLGCVYLLAQEKITFRQIADNLFLIAELGC<br>FIPKYWPLVRHAERIKRCIHYFSAPIFKTDRKEHQEILDDCIKVCHQWSAFYFASVTAGFVSWSIRPISWENHI<br>LPTDIWLPFDPHTASSAKVASVYFYLVLGKGFGLGIKILKNNLQHLGEYVDEELASLEPCRKAQLTYQKIRQC<br>VIHHEHILAFVEEYEECFSQVALSQFVGAVVIFCVSCLQLTIVEVVSLDFLAMMMYFIAMLCQVYLYCHFGTIL<br>YDESDTISDAIYLSKWYEFDKRSKKALCILMERLKRPMTVTCGKIFTMSLVTFTMILRRAYSLLAVLENYNIEL<br>N

MperOR21	MPSLLKTESLALTLTLNTLSWAGLILRDDYTKTQRIIMKVYGGLVFLYLFVFTAYVQIADLVVIWGNIDFMTETSLILFMQLAVSAKVLTLMLKSKKIMEVTNEADAILNSEKKVEGQRIIASIDKNTTLFLKYYGFFVAFTIICWFMGENTSTFFIRSKYPFNELKSPGREFAFVHQCIVVIFTGSFDFNVDIIISLVAVCRCRLKLVALSLRNLCLDIPMNKRNLITSDEEKVITERLRNIISQHKRALDAAEAIKHYLSGALLVQLMVSIVVICTTAYQLAVKKSTTMQSLTMAGYLFGTSLEVFLFCYQGEFLRESSEEIADAAYECPWYTLTRPLKKTLLIIMTRAQRPATLTAGGFVTLDITEYMAIMKASYSFFTVLQQVSE
MperOR22	MPSSFFLPNLENPDYPSLGPTLKGLKYWGMWQSGGIKRILYNSIHAFATFFVITQYVELWIIRNNVELALRNLSVTMLSTVCVVKAGTFVCWQKYWSGIIGFVSNLEKEQLSKNDAATQAAIVKYIKYSRRVTYFYWSLVTATVFTVILAPLVGFLSSPERELIANGTLPYPEIMSSWVPFDRSRGFGYWVTALVHTLICFYGGGVVANYDSNAVVLMSFFAGQMKLLSINCSRLFDDGNEVISNNEAMKRIKECHYHHVFSTIFNSLMSPVLFLYVIICSLMLCASAVQLTTDGTSNMQRIWISEYLMALIAQLFLYCWHSNQVLYMALEDRLGGLFEACLESGRFPSKWKTGRLVLLRKDGRPADSPAGYRPIVLLDEAGKMLERIVAARIVRHLTETAPDLSAE
MperOR23	MPTPTATETMTWSDSGNSILKVNIRELCLSGVWPLTGRKLFRVYSVIIWILGLENIVEAMVGIYLSNGDLEQITLVLPNTFTTAGGVFKMAFFLRDPYSYNALVRLMDELISDSSRYSTGNQQMLSIVRESRRSARRLSVFIYAFISTQIVIWFPMPLIAYAGEGKLPFIQHPWINSTTFPAYDTMYALQCLSSGFHIFISLGMDCFFAVVMIHTAACLRILSLRISALRSGDAGSSEVPVTSACSWGRESVAHDEMYKNLRACICSHQKIIGFISYLETVMNPIAMTQFAFSVLVACVALYQATYSEDMSAAYRCASFLPTPGAQVFLYCWAAHNIMEQGLAVSAAAYDCSWVSGDARFKRALRILMCRAERPLVLTAGHLYPVNRPAFLSLVNASYSYYALLGRVQSR
MperOR24	MPVSQSLWNMIEGKMEITEEFEQKVTETPVERTAEDVFSTQIKALQIVAMWPNFKQNSNMELFTRALLKINTFVLAYCTLALFVKGLLTQDLVDRSEAMDIFTLTTSALYKMIFFYTHHKEMDDMVNWGAALAHQVPPKWMQYTTFFSCFHNFMGIFSITFWGLCPIFKWIFGETDLDGMTLPINVYDPIGVTGAMYSVFYIVCDYGLLSAVQIYMASDAYLFTAIHLAIGGFETLNNKLRKMGQINFNKGPTVNDSMNEYLKDCVKLHTHILIYIRKIDRLFRSMIMADVLHAIISLSFAMLQASESKGIFENMKMAMFVSYCIVHQYLNNYFGQNLIDQQEILNKELLISVPWNDGSKEMKKSYQIMMAGCLKSVRLSAWSVYTLQYATFLEFVKSMISYSMVLRQVQDQTVKQP
MperOR25	MPYVQKDDPFWVLKKLSIDIVSHTVMKKLNFAICSFNSTLLLLQTIHIITSTNKILLMSAYGPMMGFSCFVIFQALAQTVYEKTVLELLCEFYSLYWPLDNVNPGKFLKQFRFLYVISFVTGGMFITSVLLVSPVFKNEKDIFLIREMFHNWGQILEVVFWAGLFFQTVWAILIACVLAYAIFGIKFQLSLLLYQIKGMKGLRHQSMVKEKLHSVIRRHVCLTGFVRKVVKTYWGLLQVEVCMFLVVNISMLFFFINSFSRSDWQHNLRPLCITVTSFMLTTCLIVLCLQIPDMTGRIFDTTLELPWHLWNSKNRRTLLIFMTNSVQPIYINILGLGRLNSSSVSEYVKMIYSTTTVLCSLREGKK
MperOR29	MQCQPLFTRRFVLRLQILNLRNLHNIIQRIGHMAMGYKKDGLIKDLWPNIRLIQLSGLFISEYYDDYSGLAVLLRKIYSWITTIIIYSQFIFIVMFMVTKSNDSDQLAAGVVTTLFFTHSMIKFMYFSTGTKSFYRTLSCWNNTSPHPLFTESHSRFHAKSLSRMRQLLIIVSIVTIFTTISWTTITFFGESVWKVPNPETFNQTMYVPVPRLMLHSWYPWDASHGLGYIVAFVLQFYWIFITLSHSNLMELLFSSFLVHACEQLQHLKEILNPLIELSATLDSSVHNPAEIFRATSAKNQAINGIDRDYNGSYVNEITEYGTKGENESNRKGPNNLTSNQEVLVRSAIKYWVERHKHVVKYVSLITDCYGSALLFHMLVSTVILTILAYQATKINGVNVFAFSTIGYLMYSFAQIFMFCIHGNELIEESSSVMEAAYGCHWYDGSEEAKTFVQIVCQQCQKPLIVSGAKFFNVSLDLFASVLGAVVTYFMVLVQLK
MperOR3	MQDQLDHELERIDKLPKLGLLWVEYSAYALGVNIAPRKRSSKYCRLTRILVLIVNLSIIYSLVAFIMENYMISFETYVEAVLLTFQLSVGVVKMFHFQNKVESCSQLVFSTETGEVLKSLGLFQLDLPRKKELLSSVSLILLNNWMIIDRQVMFFFKIVCMPVLYYCVRPYFQYIFDCYIKDKDTCEMTLTYPAIVPYLQLGNYEFPSYVIRFFLLQSGPLWCFFAVFGFNSLFVVLTRYESGLIKVLRFLVQNSTSDILVPKDQRVKYLQCCVRLFARISSHHNQIENLFKYIILVQCSVSSILICMLLYKISTVLEVGWVWMGMIMVYFVTIALEITLYNVSAQKVESQSELLFHDWYNCSWYNESREFKFMIKMMLLFSRRTFVLSVGGFTSLSHKFLVQVFRLSANFFLLLRNMNNK
MperOR35	MQKPHGLVADLWPLIRMVQYSGHWMLEYSGGKALRAIYSSAVSLLVVTQFALMAVNLIQRSGDVNELAANTITVLFFLHPVTKFGYFAVRSKAFYRTLATWNQSNSHPLFAESQARFHQLSVVRMRRLVMYVVSVTALSVVS

	WTSITFMGDSTREVTDPDNANETITEEVPRLMISTWYPFDASSGMGYMLAFVYQLYWLTATLMHSNLMDV<br>MFCCWLIYACEQLVHLKEIMKPLMELSATLDTVVPHTSELFRAASTLPTNEPLYDAGNGAADGLTIRGIYSSQ<br>RDFSGFNRRSAALSTVREADAGGAVSSAGGIGPNGLSKRQEMLVRSAIKYWVERHKHVVRFVGNIGDAYG<br>AALLLHMLTTTVTLTLLAYQATKIDSVDVYAASVLGYLFYTLGQVFLFCVFGNRLIEESSSVMEAAYSCHWY<br>DGSEEAKTFVQIVCQQCQKSLMISGAKFFTVSLDLFASVLGAVVTYFMVLVQLK
MperOR36	MQKSERFILTPFQKFCIRWSVLFDSSSDRLSRTETVLRSVQFSTIMITSGMTMTSVLIADNKKALESFTYFVICVF<br>MLAIITLAIRTKRFNRTMLLMVEDEFPSYNRPMPDALKRKITDIRTSYGDFTMKVIVSYLTLVLFEIPATGMVP<br>LAAATLTEVKLGSQSTQMVVLWFPADTSQVGMYALSYVIQFLIVVTVKFIITGIMCSFSFFVNQMISEFQILSAY<br>IEHAVEIVEFDQSVDKTTEQKLLEHVKSCTMLHDRLIHFKDQLNESYGYIILLELMFSTLYFCLSAFNMIFVGN<br>RFVMVKGLLTLSNYLAELFIFCMYGSMVEDAHMGLLRASYSAQWYAQPVRFRQSLMMVMSRTQTPLQLTV<br>GKVFIANLPLFLSVLKVSYSGVNALRAANAK
MperOR37	MQKVKMHGLVGDLWPNIRLMQLTGHWLLEYHEENGGMLRLLRMAYCWMTTFSIYIQYAFLVCFLILETYN<br>ADEMAAVTITTLFFLHSVTKFTYFAFRSSYFYRTLGAWNQVNSHPLFAESNARHRATALSRMRKLLMIIGTVT<br>ILAVFGWTTVTFLDEPVWDKTDPDNVNETISVEIPQLMVYAWYPWDARYGMTYFMTFVFQLYWLFITLAHS<br>NLLDVLFCCFVIFACEQLKHLKEILQPLMELSAALDSVVPNSGDLFKAGSAGSDIALIGNGENGNDFDVRGI<br>YSSQRDFSGFQGGVVNGGTVGPNGLTKRQELLVRSAIKYWVERHKHVVKFVSSIGDTYGSALLLHMLTSTV<br>TLTLLAYQATKIEAVDVYAASTIGYLVYTLGQVFVFCIHGNELIEESSSVMEAAYSCHWYDGSEEAKTFVQIVC<br>QQCQKSLTVSGAKFFTVSLDLFASVFGAVVTYFMVLVQLK
MperOR38	MQLEDFMRYPDLVCQAAQLPRYTWNGRRSLEVKRNLAKRIIFWLGAVNLVYHNIGCVMYGYFGDGRTKD<br>PIAYLAELASVASMLGFTIVGTLNLWKMLSLKTHFENLLNEFEELFQLIKHRAYRIHHYQEKYTRHIRNTFIFH<br>TSAVVYYNSLPILLMIREHFSNSQQLGYRIQSNTWYPWQVQGSIPGFFAAVACQIFSCQTNMCVNMFIQFLIN<br>FFGIQLEIHFDGLARQLETIDARNPHAKDQLKYLIVYHTKLLNLADRVNRSFNFTFLISLSVSMISNCFLAFSM<br>TMFDFGTSLKHLLGLLLFITYNFSMCRSGTHLILTSGKVLPAAFYNNWYEGDLVYRRMLLILMMRATKPYM<br>WKTYKLAPVSITTYMATLKFSYQMFTCVRSLK
MperOR39	MQNEKEELPSCEKLFSLELAALKFVGLSSLRNGFRKENMNIPVKFYEIFLFICAASILSGFFISSALTVHMLLQS<br>DFILACEIATFIFAGAVTVSKVLRIWSYRIELIEILRELNELWEKIVKHRLNLKENILNMLNESRPIRYGYCFIASS<br>LNLSYALRPYFHMLVYFVKQSENKTIDLTVTTYPLLYPIERGTWLGYLLCVTYEQSILYFGGIYWIMCDTLFILL<br>TSHICVHFMIISNDFNNLHINYDKNNNESFQLIEDLSRRHQKMFVLCQRIETLFSPIILLTVVFNGIDLCLCIFA<br>LDKDLSDGNWAKVASSVTHALTLFFQIVIYCEFSHVATEETSKVGEAIYNSSWIYFDKKMKKMLLIIMMRASK<br>EYKFSVFGILILDREQLTQIVKTTMSYFTMLRSFS
MperOR4	MQNNNPDYLSFSYNILRSIGVFSASDSTKWTRWAFNFYRVIIFIVITLVTTLMMVQMFVATDLTLLARTIDIWT<br>MFSTGLYKWFYMTMFRGEFSQLKTALTQIQAQGSVAYGSSADAFTADYLKQTRKISFWYMFSGMVASFFIIVS<br>PLLTYSKGDQSDFQYYNDPKSYPLSCWIPFTLNENWMFLAVFVCYSVALVLIVIVYLGIDTYLFVAIYAIGGQIE<br>LLNTSLNNNENTLEQFENSSCTNRLSIYTEKQQILFYSTLRECVKHHILILNYITNIRKLFSTLILMDYLHGITSV<br>TFALFQLTISASVIETISVVCFICLSIWHQYLNNFFGEFIIQKQLSVCTVVYDVPWWRCGERVRQLLTLMILRSI<br>KPTFITGFYMYKLSYESFISFIKALYTYYMVLRRVNVED
MperOR40	MQPNLQKNDILWLIRKLTFDLFQLKITKMFLIITSVSIILLTIIQTFLFLKKFNGYYFIMYSAVYTGSLFILVSSLSV<br>LPISKLIKTAWVKFSFWEINSATPKIERKIRKEIFYINCVVFFNTIVAIISGIFHAIPLQDDEELFYPLAIFETYTPE<br>WKDWFSGIYRASFLPMPIIMVAPAYTVVYLCAHMRFQFCLLLHFLENINPDNENISDKKYQAQIKERLHFCI<br>KRHIHLFSKSRPVLEDLKKFVFVLTLCGTIFCISIIIFHFSFQGTYEGRYPRIITIIIAASITFFLSILPGQLIENTSSEIF<br>EVLRNTNWVSWNEQNKKLFIILLLNTRQIYKIKITENVSLNYELGVTMAKAMYSMISVMKQL
MperOR41	MQQADPLWPLCSVVGELGLLGLWLPPPGRRLRHRLVLAFVVASHLGCFFGALASITMDTPSDLPQLSFIAYN<br>CLTDAGLTCKMLSFSLDGCRLTELLRLLSESRRRFPDRAGHRASQHATAVRIHRFLQMMYRINTAYWLLGPV<br>VRNIVAAVSRQPSISVRDIPVPLWLPFDARRSPVYEALYGLVLAFGWAISETSVLVDSSLIALLLQVVAELAVLN

	DNLASGTPPAGKLTTRVTADAAGSRVQIPLTTAPSVLEGAVCDLVHRGEVYQHILDNIQHHQTIISCVRLLQK<br>VLSRATCVLLFCNTISICFQVIATAVLLQEDGETMQTLKILMGSTLYGYQVALFCLLGQRVINQSERLIRSAFSG<br>DWPEGDVRSWRLVHMLCMSTRQSLSLRICGIYTLSRMTLLQILNVSYSLLNFIYQTKTEQSSGKQEL
MperOR42	MQSKDPVSKSVSFRPPDGGSQNKDGDSAQPTESQDSFLTWKGSSESVLKYNVRCLCILGVWSLTRSRLYYLLS<br>GTAFLLGVMHIVVAIFGSYLYRDNMEEMTLIVANMFVVCAGVTKLVIFVVYRNNYRQLVTVTDGLTDRQRS<br>YCQGDPALKSILEDSERLAVRLTLFVPAYIATLSVVWVPMPLIAYNERRLPFVQLPFVNEVSASTYALLYVMQT<br>VPSLLFFNVGFAVDAFFASVMIHAATQLRILFHRIKDLRLDGRGTLKLLSGDRHDIMYGELCTCIQLHQQLVR<br>YLSFIGKVMDPIAMTQVVFSVLIACTTLFQANYSADTNTAFRCLAFLPTPGTQVFLYCWGAHNLMEQSAAV<br>SEAAYSCSWVEASRRFKRALCLLMCRAQRPLVLTAGGLLQINRPTFISLLKASYSYYTLLGRVNNR
MperOR43	MQSLIFFRYLSRHKDNYILVESFQHSQMAEFNDPFILLRTILFTNMNSYKILKQCNVLLNIIYLLIHCLLIYYMF<br>NNLNLNLLIRYGPGMLFFIVVTAAAVLFLHLEKKILEIVTFCDKICWSLSLIRKSAQTKLKRKCQIVNICVSFVL<br>LLLVSTVTINAPYFGNQRELFICIQVFEEYFGEWSFVPYYFYFVGFPFLFYNFMRLWISFVYAILEAQLQFFLIEE<br>VLYETYQIRYLKGWKYLQDTRYQQDIGKSLRLCIAHHNALKKLLKMVVNVTMTTMPFFLVFGVLLLISSFAFI<br>INFADTMTNILKIRMFMFALSIMCVAVVLCWIGQQVIDVTSKIFFALVGAPWYFWNAANIKILLMFITNCTQ<br>NDSIVLAGICLDYNLFVTMVRISVSYALVLFNLRKNSVA
MperOR44	MQTGEVEDADGHLSWEETARSVLRLNIRHLHLFGLWPLRASRLFPLYTAYAAALGVWNTAEGFLAVYFSW<br>GDLEQTTLVLMNTFTNASGLAKICFFARDRRRYASLARGADRLARLQAQACARDRALAQVLRRARRAARR<br>LTAAMLLFMFSQCFVWFPMPAVAHADERRLPFAQHPWDNNTAHYELAYAVQCVAGLWLSQISYGVDCLFA<br>SVMLLAAAQLEILAGRVAALGERRNGRGGEPLPADAPDSMYADLCACVEAHQKILRFVSDLENTMSPIAM<br>TQFVCSVLVACSSLFQATYSKDLSAAFSSMSFLPIPGGQVYLYCWAAHEVTEKAQMVSAAAYGCSWVGASE<br>RFKRALRIIVSRAQKPLVLTAGHLYPIDREAFLSLVNASYSYYALLGQMNKR
MperOR45	MQVLTFNFFLLSIMGVWKPRGWHGIKAALYYIYQTITIILNHLCLLSLLLDLQFKNIELGDLIDNLALVFTIIIIR<br>QKIVCIIGNRPGITHILDSLKNSPFKLEDSKEEFIFSRFEKLARNIITYYPLIYLSTLSVHSTGFISVMDPPYTLPYK<br>GWFPYNYTRTTKTYWVTAVYQIYIVLTMGSINAISDILLPCIICYMCGHIHILRYRFQVMAEKLRIMSKNNEPK<br>DKIISTERKLMGDWVKYHIDILNLVKFTNEIFSSVIFIQYTVSSLLLCTIAYLLSHMEPTTMRFAGNSAFLTAMF<br>FEILLPCYCADKLTFEFLDISTGIYDTNWYHLSNNIRKSIVIILRKSYRPVTMTSGFFIVLSLESFTKVIKLAYTIYN<br>VLE
MperOR47	MQVTRHERRFCPQPGTRTVSFLIRYRESIRTIGAIFQKHPFPELFNVVMQVTSHERRFCPQPETASMREEDIPW<br>SDTVLWLNARVLALGGSWRPPGARGFALYRLWVLFTQFSFLIGQLQGLYYFWGDTNRILQDVCLLITTILGLF<br>KFFTFVARQEQVFRIVRSVDDRRREQSKLGDARVTSVLEASRRAARTITVWMAGVGGLAPAVWASMPLVMR<br>GLGLAPPERELPARARYTDRDTATPVYELLYLLQFFSMQYSYFAAMCIDLFFACLILHVAAQLEVLNVRFGQI<br>REDYYRNGQTREARDDAKVEDVEEDAAWKELCECVEHHKGAIKLVDDLETLVNPIILSQFMGSTIIICVTLFL<br>ITTNKQHFAALVRLKAYLAVVVYEIFMYCWFGDDVMYQNSRLVESVYACGWPGAPPRLQKALVVVLQRAH<br>RPLGVTAGKFYRVSRETFVSLMKASYSFYALLNQMND
MperOR5	MRAKTEFEKTIKLTKTALFLSGINIFLGEWNHWTRTFVDSIAYYLNIVGLYFVLIGEMYWLIDGTITGKSFVELS<br>LIVPCLTISVLATAKVHYLYHNKESLLDVVDKLREIYPDEIEETANDNDQCLNDKKETVYDNDVTEVGIVNE<br>ANELLKFVNFLLSTVSFVVTMTFCTMPLFGMAGEFMETGKFVVLYPFAVKYPFDVYNTSFWVIVYVNQFWA<br>TIIVCTNIFGVDTLFYALCSYIGMNFRLLSYKFEHLEIKRNDRIINEIIVLIKRHQELIELVNKTQSLYSLSTLFNIV<br>TSSLLICLSGFNITILSRSWSYFALLKTIYS
MperOR51	MRCYNHDVLWLLRFWCCDIFQYKLLKFVIFVMLVANTVLMVLQGYHFLINFNSLYFISYSPYWFGSFFIILSLT<br>TCLTISNIGPEGIKSATLWQIKSTDPKLINRIKFQVKLITAYIIVNTVIALIAGLAHTFPSKNAEEICYVYKIIEIYV<br>PKWKTELCWMYKASYIVMALALPATCNQVVYGATHIRFQFYLVLDWIRNNIIESGCDDLKLPNDKDFQHKI<br>TKNIIVIVKRYTEFHRTFQAVNQRIAIYILLYAVIGGLLGISILMFYFKFSDTLVISDYLHAGTLTVAAITTFMATV<br>TSGQKLEDIFEELLYTWCSVPWYLFNKWNKQIYLMIMVNIKPINFRFTENSSVNYNLGAAIAKTIYSMLSLLT

	QMSDKDVSTL
MperOR53	MREDKMEINNSQKFYTKMIFRYLYSVGLGDWWYQHEDRSDSHRKLYCLWAVISNAYIFLNICNELLANFRK DLTDVEKNDAIQFSFAHPLIFAKIASFFFNRKKIREVFGRLLEENRSVYSCGELEKESMKQIKRYSLAFIGVSYM TLVMSTIDGLRAHFKEGIPIRTEVTYYPSPSNSGVIVNILRFLVEFHWWYIVSVMVAIDSLAVASFVFVTFKFKL LQRYFKDMGLTVRRDQSNMTDEALADKFRRDFIVGVKLHENALWCAENVQKAFGWVYSVQVFETVALLV MCLVKLVTTNHNMIFLLANFAFMLCVIILNGSYMMPAGDVTYEASEVPTSIFLCGWELVRQTDLRFLVVVAI QRSQVPVIMKAFGIMTLSYSNFIAVSLFKFYVQFQINLF
MperOR64	MREKPLHLSHFPYYLLKIMLCDTEQYRLGRFLSYSCAVIHSISLLLQMYYLIDNFNKETVSRYGCVVIVTTYCV VALIYEILYAQPSVSMMSQQISTLWPMDACGEKVKQMILKRAFFTSVVTYSILFSFPIFGIIMFPLWGDQSDMF LCVRVFNEYFTKWSKVPIYLYFCSFPVLTFSGIRLPGMLLYAILITNIQIILLNQKIAHISDLGDQRLVFGTLCSCV SLQIKLRQMLNKVLQFVYLVMPVFLLLGALTAISVLFFLFYSLENPSDYLMIRLACFLGGNILVVFTFCESGQA LSNDTGRIFDILLTCPWYKWDKKNKNILLMFLVNSLKPMSITIAGITLDYKLAVTLIRTCCSYALVLYQMKN
MperOR67	MRELEEEWERQRLRGLRLVVRALQLMGAWPPGADAGAGGGGGGGGVRRRLYRAYVAAATALMASYPAC ALCVLAGTRADIQVTAVLVGVTSSYVGAIFKMFTLCYKRSQVSGLVRAVQREFPRSPLLAGRAQRAVAASAG QHGARLTALFVSCFSSVFNWSLGPIALHLLGGGGGALALCLPWFPDHQPAGAAFRLVYGYQVATLVLVALW VGALDAFLLVLLVYAAGQLRVLNCTLLQMGARSDDGTEGQREKFASNASAMLRECAKHHLEVCRFVQDV EQVAAPALTLQLLVSTFLLCMSAFTATQIPVGSPLLARVVVYMLTGASELLIFCKYCDDVISESERVQQALYGS GWSAQGGAFSRGVLIMLARAQRPLCLRAAHVHPVSLQTFTKVLNASYTVFTLMRQIKD
MperOR69	MRGKTIESTTNPYSSLKKVFIDFAYSKLVISYTKASLTFHVLSLLLEVYYLVTNFSVELICRYGCMMCLMTYVVA TKLVGTMYSKKLKLLEKPCLLDFWKVYNSSTATQRLISEKSSKTNRRLYCALTCCFFLAIILFPIWGDLNEFFIF SQVYEKYFTSWAPAFCYFYVSTLLWCCFYCFHLPGIIMYLTLHLDLQFKLIKDKITEIDKNCSQKEIYQILRLCIS HHVALKKWMDKLADLLVTIMPFFFLFGALNSIATSFFVLYTLQNTTMILKIRLGTLTLCNFIIVSTFAEVGQIFS GQNNSLFEQLMDCSWYLWNIKNRKTLLMFMLNCMKPKTFSWGGITLNYSFVLFILKTSLSYASVLFKLRGET F
MperOR78	MRGRARLFALYTAALYACFAAVLLMALQLAYLSQRDINELTYALIVVMSHVGVLFKMTHFLTSRGAYLQLVE RLNRLVGQSLADDSSGPVLAACHRKAMRLTFCSFAYLALTGAIWYLVPIVDAIRSGSEGRRLPVANPHWIDT SKTALYAFLYVVQFPSIFYFVGISVGLDGFFATTMIHVATQLRLLSLRLSGLNHGGSRISSVTKFSEDNKSDLPSP LRVREIERLDESEGMYQQLVQEIKRHQEIVSFVKFLEAVMSPVAFVQFLFSVGSICVTLFQSTFNPRPDVVLKC AMYLPTPAFQIYIYCWCGHDIMEEGARVSLAAYSCAWTGASKRSKDALRMLACSTQRPLLLSAGKIYPVSKA TFLSMINASYSLFAVLQQMRSR
MperOR9	MRGTMELDDEEVMQTLQESGLRTWIGVIAGFRFAQQPRFKGTLKGRIYWWYEILTDLSVAINGISQFAALLT PEFRMIDRCLMCFPAASCLLCLFMSNYPRFKRKNFRSLVEEYENSFSDSQYRHHLEEQIRKGAKHTRSVIMCL VLLEFISMFIFCLILPVLNEATGFAFGPRRLAVPSLWLWDPLAGFWNYMAVVFVQLCGSVFVSLKKIGFLESFF VYASRQICMFTHLRYNLGKITDPLIVNDDGKVDVKEFTGSNRYLMKRKLIGWVKNHQNCLRLFEDLVKLYE WPLLVYFGATILILCTATYVTSDNSIDAQTCVICGVFNLGIFFELLFICRTGDRIKHESEKLLGALNGKNTFLLK SDEYKYLKMILTRCQSESVINASGGFPLTITTFIAIIKSSYSYYTLLKKVNGQTD
MperORco	MRLEIEALKNFPYYYLLKICIDFGYSKIVKCINVVCIIINSSTLFIQVYYVQQHFNKELIFKYGCGMALTIYTIASI SVEFLIEKNAKNLVNDATAFVWPVDFCGEKVKKLILKRATVMNKICYFMSAWFALMGIIMLPVWGDHSEW LLCDLLSKEYFETRWKILYFACSCFSFPVVAFSSIRIPGILLCTILQTHMQIILINQKLNQISEQMGNLNNIKLVD DKCYQKRIFEDLRLCVSHHGKIKKWLNKFLKLVQSIMPLYIILGCLNFISLLFFASDGLQNASNILKARLCVVL IVCCLVLSMFAEAGQALSDETSGVFDTLLTCPWYLWDKNNKKVLSIFLSNSFQPDSISVAGITLNYDFAVALL KTSSSYALVLYNMKN
TcasOR1	MRLGFEVSISEYLYRNIFYIYTLFHILLHFYYILHMIKLDLEAIFDDIDESVALLPHRDTRRIEVQKILNGRMKRV

	VTWHISVFKAVEAVSSIYGPPLAYQVMFTSIAICLIAIQITQKLENGILDIRFTMLGVAACLQMWIPCYLGTLLRNKAFGVGEACWNSGWHQTPLGRMIRQDIIIVLLRAQQPVTIKFPGLQSIQLETFSSVIFNLYGYYYFLLLRWVDELTAHLVLSGYWSP
TcasOR100	MRLRAALLAAAHLVLPVTLSVNFLSCRFDSLDLLATNIFLLSGMAGVSSKALLFVLDRERFELLLLRLQRTRMRFPDNSGARERRRRMATRVYYAQHGSAQLVVLLWVSVPGVTALVSGEGRELPMPLCPPATAADVHLSPCFELIYALQAACLFVAVEGMISLDSSYLTLMLNIATELEVLNDNLVSIRSGRLPEDKKCSQLSDQSPSNPAHEDDMYFQLVENIKHHQHIISRAQELESVMSGPTFVHLFYSLVCISLSIVSVTVLLQTEGYTPKILKITFVIVVFVSQLGFFCILGNNVIEQSERLLVSAYSSYWPGAQPRFQRALLVLMLRARHPLHISVAKLYPLSKETYLQILNASYTLFNLVFQTNGRN
TcasOR102	MRMTENGKNSDPDSLEIAEEKATKYLYQRFFVLPVVGGVFGFHQTRWWSIFTKTVFTAYYVSVLSTIVTLSYSSYLNRDNMAVASGCIHILITAFVVLGISLTLQRLRKDVVQLLSLDEIICEYQCSEYLSNLIRKSDKKLRFLLIFWVALYGSSAWIAVIFPFIDVYLGYELSLSNVTDVYWKGLPFASWWPMDADNSNLAWTTCFMSQGLYAFFAASSATSGMLCFAIFSEDIFNHIKLLVNSIERLEKRAKLMFKMLHPGKSLRNSLDEYDECYYNCILQNVKHHQKIVIKKDLLMKIANIPVAVPFFGGAMLLGLAGINLLSDGDIRIAPKVLFTCLGVTEAAQMFLLCHYGEQFRTQSELLFNATFYTKCYRRSMKCKRAMMIFRLGVSRPMAISAAKLIVLNMGTFANLVNSAYSIFNLQSITTQED
TcasOR105	MRNFQDSDDPFIFIRKVFVGFGCSTIIMYYSRLIFIFHTLSLLLESYHVITNFSLDIITQYGSAMSLMLYSITSQFLLICEQNLITEVVEECKSFFWTMDFLSFIKQTQILKDMTKIKRKMYLSWIWFVVFGIALLPVWGDYNEMFLFPFIYQTYFGNWSPLFYYFHASSFPFLAYIAIRIPAFILYLTLALHFQTLLLNQKILQIPQNKSGNQEDIFRNLCSCISHHVALKKFVTKTQQSIQKMIPVYFVLAILCLVAVMYSCLNSLAMSTSNHFKVRGFFGGVCGVVVLYTFAEAGQLQADTTGEVFNTLMQCSWYNWNNRNQKILLLFMVNSLKPSYIDWGGVIVGYSFGSSVIKTCYSYALVLYKLKISKEQNVTF
TcasOR107	MRQTRSVESVLGSSAALLRLLGLWSPRIEDFSRTHRVLRGGLMLALSFGLMVTTLLKLVMDCPRELEELSACIFSATMLCEVFFKMVFFVLKVPTLHKLVQLLSEIRTEDSIGERNDEIRRRYQIVVDKMFLFLMATAVVTETMWAAIPLMHQLLNMDGEVTRLLPLPLWLPLDVYASPTYEVIYGAQVLLMPLTTTSLFFDFVFIDLMMRIAAELEILNYNISSVHENRKTVSTMSKDIHKLCQTVSDNKTNVQLVKNVRHHQAILRAVVLLEEAMNTGVFILFLATTIAVSSNIFTATALLQAHDGRIKALKMLSAMPPVLFEVGLYCVFGQIVINQSEKLMHSAYSCEWVDCDTRFRRSLHMFCVGAIRPLEFTVGRMYKLSRETLLQVLHGSYVMFNMLYTIQNRK
TcasOR108	MRRLKKLLLVDIRQKLFKPRNRQQVVMVQSRVNFWKKIYFMFTGMGVATMFFWALFPIMDGTVKEHRLPFLAWYPFSVNKSPFYEITYIYQIVSVFFIVIVNMNSDMLLVALMNILGVQCDLLCDNLKNIQFRERINEEFLRCVNHHMQILSYASDCNKFFNTIVLAQFFTTVVSLGLTMYQLTIVTPFTSEFYSFIVYGGAVLMEIFLYCWFGNEVEFKSLNIPFASFGFDWTIGSVGLQKNLIIFIAKSQRPIRMSALNLFHLSLETFVKILRTAYSYFALLNNVNSLN
TcasOR110	MRRSKIASSAAPTDNGGDEATKNQEISSDEGDYNCKDSGTIVDVDLFKAIGVYQLLHPVECGLDSGLCRMAVKIIVGLTLGLQSIQVCRLYLARYDIPMFANMGVLVVYGLMCLFKGYTLATHADRICTTLEVARYSFTSCGGRYPSLMRQCRARLSTILRTFVGLSFGTLFVWLIIPWFLTSEYDDKPIVWAVVYVVESIIFTVNVFCWTSFDCYLVTMCFVFEAVFRTMSYGYENVGRVHRLYPHAVQPFLDYRSKDSDIQSDVPLKFPDHYDDLINHIKDNQKIVEKYETFFDVVQPVVLLQIADGSYSVITLIFLISISYLNGDSIISPAILKFFCGLASVIIELFIFCYGFNHIEVGRSTVNFGLYCCDWTEKDLKFKKTVLLAMSMNSAHKQVMKLSPNSIVNLEMFARVMNMSYTIVSTLLS
TcasOR111	MRSSSATAVDVMLFKTIGLYQLLCPADRGGYSVRFRRLLMTALGLSFALHSFQVPCLYYALNDLQRFAYMAAVIIYGMMCSFKGYVLVTNADRLWLVLDAAGYAFTGCGHRDPSKLRRCRATLSALLRTFVALSYGTLVVWIALPFFVDEYTGVTNLDGTVTRYRTTIHNMQFPVPLTVYNSRPFWALIYFTEVFVCIVNVFIWSLFDCYLVTMCFVLNAQFHTMSAGYCTLGSRRRRESSQPNTSRTGVRRIKFDDMESNHYVDLIGHIQDNQKLIKVFDVFFEVVWPVVLVQIANGSYSVISLIFLTALMYLIGVPVLSAPFFKFVCGLISLTIELFIFCYGFNHIETAKSILNFGLYNSNWTEMDLTFKKTMLLAMKMNSSHKRAMKVSPNSAVGLEMFARVMNMSYSIVSVLLNSRS

TcasOR121	MRTNAKSFLFVPSKVLTLCGVWPVEKTSIFSLIYRSIMLSSQFCFLVFNGIYIGLMWGDLKAVSDALYMFFTQT<br>TCCSKAIGFYFNFMKIKRIVASMDDVLFTAMSIEDQATIFSHSRTVNKLYKGVLGFTGFTLVQWTVLSLIGSGR<br>TLPFNEMWVPTDISKSPNYEITFVVELWMMVISAALFMSVDTITVATMMFSCAQLDIIMKKTQQIQEIPLSPD<br>LSSRNRSELHEKNNGILIDCIKQHQAIVRFSELCEGTFQVHSFFHLGGIVFMICVIGFRMAGESPVSAQFWAA<br>LSYLVIILGQLYLYCWCANELTTKSEQLRDKLYLTPWYDQDVKFKRNLCIAMECMAKALTFRAGSYIPLSRA<br>MFVSILRSSYSYFAFLNQANEQ
TcasOR127	MSAEPAQTWRSSASSVVSYNVRLLFLCGLWPLRRGRAFSAFTAAVLVAAALHAAGAFVGLCTEPGGLQEVT<br>LALANLFVVCSAIVKSCFFLADRTRFCTLVSTLDRLVQVSGQQSAVGAGLRSRLSASARRAVRLTLAFHLYVL<br>SALVGWCLMPALKRQRRLPFQQLRWLDTSSAAVYGASYALQCFATFFCSFINTHLDVFFMAVMIHVADQFA<br>ILAARFADLRLDADADPEGRLPQGERAVLAEDAYQQLRLCVRSHQELVRLVQLLDDVMSPIAMTQFVVGAI<br>NACMVLFPATYSTDVGAVLKCWAALPMVGIQIYLYCSGAHDIMEEAGAVSGAAYSCSWLGADRRRRRALL<br>LVTCRAQRPLQLTA
TcasOR128	MSAQTIQSDGGRALLNRDDVKGLNMGLNTFGAKTFWHTLEHFHATGKRHWVMATYIVLLHLVGFTYCLL<br>GFSAVFFIKMDIKRGTAAIMNPICGLQTVFKCWTFSWSTAEYLKLFEILKKDFLTCVPPQKEGGANDVLAKN<br>VVATNEFVKNAMRWNFLTLCMVSTMPYLRSQAFREFFRLGEGAIVPNKICENEYPFEWNSTPIYEIIWIYEQI<br>AVILAIVTSSAYQAILLFLVMALVGHLRVLGYVMENLRASDFRGETYQLMDKSAKANAYQQLIRCIRDHQKI<br>NAAGDALAERYNFFLTFHLGTAIIVGIIAIFNCTAADELADKIKFAIMCGYGLLEVAIYCYCGQLLENASEDV<br>LRQVYQCEWEEMEPKFRKAAQLMMVRANNPIALRAGRLYRVNLETLGAIQQLVYSALTMLSSMIDGS
TcasOR129	MSARPLHLRNFPYYFLKVLVFDFEQYSAGKVLSYFCAIVHSISIFLQMHYLVKNFTKETMFQYGCVLTVLTYC<br>VVALFFAIASGNFVEKLESEISSFVWPLDICGEDVKAAILKRAFYTSLVAYITIIAFPIFSVIMFPVLGDQSDMFLC<br>VRVFNEYFTKWSQIPISLYFYSFPVIAFSGIRLPGMLLYAILITHIQMFLLNRRIEQISELSNQRRVFETLCSCIELQ<br>AKLKRLIRNVFQLVYIAMPIFILLGAVSSVFVLFFVVNSLETASYFLVLRMGCFFGANVLVVFIFSQSGQSFSDE<br>TGRIFDTLVMCSWYNWDKRNKKVLLMFLANSLEPMSITIAGITLDYKFALAMLRTSCSYALVLYQMKN
TcasOR130	MSDHFRYVSTIFKLSGYLPSLKTLKTKILKYVFSPIMFTLITFIIYNFRYMHHDIFEIARTCEALSTHGHVFGRKL<br>AVLKHANLIEQVINDRNFFWSYEKFGEKLGQRFRQKLFFRDYVMKSLSAMSVLMLAYFYLTPVFVRSVNLP<br>QASWVPQFSHATALVYFCQVVCLSESIPVAVIIDGTFLLMGAELEIQFNLLKKTLKAVEIGQNSGQKHEEKCL<br>KQLKICASYHDFLLKEHVKMKTIFSEFFLLQYLLSIEGLCIELFVVNKAQTWGQFALGAIYVVGIIMQSSFTFLS<br>ASNLEIEVSITKFKKLTNKI
TcasOR131	MSDKVKGKKQEEKDQSLRVQILVYRCMGIDLWSPTMANDRPWLTFVTMGPLFLFMVPMFLAAHEYITQVS<br>LLSDTLGSTFASMLTLVKFLLFCYHRKEFVGLIYHIRAILAKEIEVWPDAREIIEVENQSDQMLSLTYTRCFGLA<br>GIFAALKPFVGIILSSIRGDEIHLELPHNGVYPYDLQVVMFYVPTYLWNVMASYSAVTMALCVDSLLFFFTYN<br>VCAIFKIAKHRMIHLPAVGGKEELEGLVQVLLLHQKGLQIADHIADKYRPLIFLQFFLSALQICFIGFQVADLF<br>PNPQSLYFIAFVGSLLIALFIYSKCGENIKSASLDFGNGLYETNWTDFSPPTKRALLIAAMRAQRPCQMKGYF<br>FEASMATFSTIVRSAVSYIMMLRSFN
TcasOR133	MSDNTKKATTKSLDLTNPYSSLKKVFINFAYSKIMIVYTSATLIFHILSLMLEIYYLATNFSVELICRYGCMMCLI<br>TYMVTAKFFGMLFSNQFKFLEEQCLLDFWKAFNSGPTTQRLILKESSKMNRKIHLALTFYVILAIIMLPIWED<br>VNDFFMFSQVYENYFANWAPVLYYFYISTFVWCSYYSFHFAGVIMYLTLLLDLQFRLINDKITEIDQNSTQNEI<br>CGTLRLCISHHIALKRWMNKLANSVDTAMPVFILLGALSTIAVSFFVLNTLQSTSVILKIRLATITVCNLIVVAT<br>FAELGQIFSDQNNSLLEHLMDSPWYLWDVENRKTLLMFMANCMKPKTFSWGGITLDYSFALSIFKTSFSYAL<br>VLYQLRGNTF
TcasOR135	MSDRHPDIAKYIKLMQATRNWYFAEDSTSHPAVDLLKRCYYHVRPSLFVFALLANGYGLYYREGFGALDGN<br>LALFPQALASLVTSSTIYFNRRHHRKLTMLLNQRFLDKNEPWMVEIKNKYVSAVWKFIKAVILYQEFVKIFYV<br>LAPVIVDSILHHIFDYLETPFFFPLTFSTFLTDDDKWTGRYYAVMFLNIWSGFEIVANLQGFIICYTVMTVFSVV<br>ELVILTEQIKSLDFYRSNGEINEQIRMVVKSHNDNIALNRELKAFLGPACAFLSLFTSLVLTLIVFTTTVTNDLM

	VILAYAVGAYFYFVAGLLYCSLGQLLDNQSSEVFDELCNLPWYRSSPDVRKSLNMMIRQAHNSLIIDYHGHYMMNLANFMNIMKSAYSYFTILQSVTGSD
TcasOR136	MSEKATNSIITSKIVNSKYALDANRWILKILGVWHFAIDSSYFHKVIALCHIIICTFLLSFVVIPGILFIFVIVKDVTTRLRISGVFSFCVMGVIKYYYLIKSNKQIGNCVKQFNSDWAQINDIKDKTIMVKYARFGRNSSIICAAFMYGSCMFYACILPHVSGVFKNKNDPTERTFAYPCHFIVFNQYESPAYEIVFSIHCCCAFVLASISNAACNLITVLITHACGQLEILMVWLNDLDSCQQNEVYAEKYSKIIKQHVKTIRFIVKIENLFQQICFVEVVGCTLIICLVGYYVLLDWNQKDTGGMTTYVMLLISFVYNIFLYCYVCELLTAKCKLISESTYLTRWYQIPENFARGLVLTIAISQNSNPIKAGKLIPLSINTFGTVMRTSVVYLNFLRKLME
TcasOR139	MSESVAVFKSLNLALKAVGFVDSASGIKSYLWTTWNFIIIFIGVEFIAITIPLNIIQDNDIYLKLECVLCIYLNLSMMFRFTVLTIRKKRLFSLINRIESLLQQQMEREFDKAFITALARRCGNWAYLFTFAMTATCIEPVVMAYVKYYFRGVEDPVPFEVNLPFEKKNNIHAVVWYEVFQFLGCSMIVISTNILFSTLSETTSELVKKIAENFEKIDETNADYLLKQTIKWHTEVIGITKETNDILGITIFADAFFALQYISIAGFLLIRVGLSNTTSFSKYFITYICVLTNPIYYCYSGHRVSLMGDVLYDSIYNNKWYRLAPKTSKNLILPLMVARRGLSWNYKALNFDMALYLEIVKQSYSLITFLKMMK
TcasOR14	MSEWLRFLKRDQQLDVYFFAVPRLSLDIMGYWPGKTGDTWPWRSLIHFAILAIGVATELHAGMCFLDRQQITLALETLCPAGTSAVTLLKMFLMLRFRQDLSIMWNRLRGLLFDPNWERPEQRDIRLKHSAMAARINFWPLSAGFFTCTTYNLKPILIAMILYLQNRYEDFVWFTPFNMTMPKVLLNYPFFPLTYIFIAYTGYVTIFMFGGCDGFYFEFCAHLSALFEVLQAEIESMFRPYTDHLELSPVQLYILEQKMRSVIIRHNAIIDLTRFFRDRYTIITLAHFVSAAMVIGFSMVNLLTLGNNGLGAMLYVAYTVAALSQLLVYCYGGTLVAESSTGLCRAMFSCPWQLFKPKQRRLVQLLILRSQRPVSMAVPFFSPSLATFAAILQTSGSIIALVKSFQ
TcasOR141	MSFENEHLGIKLTHFLCVDLIKKRPVKFLKNLIFFQYFVVQIIQTYYFLKIFEIRFFVKYAPVYFGTYFLLFVIIVSWFSENIDNYTTAQFDKWELENMTQTFDEKLYKKIKTPSMIVSIVIITNFALALVSGYFHILPDDDDKEIFFIFIFVEEHFPKWQSVISWAIRSTYIFTAYFMIYPINTLSYFMWRLKFHMYFYLENIKKINEGRPQNEKNITTCIPFQKEVRKRLIRCIKRHTEIAHLYTLTNGIFGTMILVCAVLGGLLMVSTAFFVLAFEGSFFKKIWRVGTLILCAAITSAGSTHSGESLETSGNNIFLYLKEQDWYIWDRENQKIYLIFLTNVKPLRIEFSADVGINYKLAVSTLRTVYSIVSVLSQLIK
TcasOR144	MSFHRYRPRLPGGELAPMPWPVSLYRVLNHVAWPLEAESGRWTVFLDRLMIFLGFLVFCEHNEVDFHYLIANRQDMDNMLTGLPTYLILVEMQIRCFQLAWHKDRFRALLQRFYAEIYVSEEMEPHLFASIQRQMLATRVNSTVYLLALLNFFLVPVTNVIYHRREMLYKQVYPFDNTQLHFFIPLLVLNFWVGFIITSMLFGELNVMGELMMHLNARYIQLGQDLRRSAQMLLKKSSSLNVAIAYRLNLTHILRRNAALRDFGQRVEKEFTLRIFVMFAFSAGLLCALFFKAFTNPWGNVAYIVWFLAKFMELLALGMLGSILLKTTDELGMMYYTADWEQVIHQSDNVGENVKLMKLVTLAIQLNSRPFFITGLNYFRVSLTAVLKIIQGAFSYFTFLNSMR
TcasOR146	MSFIVGYFEKILFHFDKLEEPRSEEITKLHRKHFSILLLVSSVDLNLGRKYLFWTLLHAVFNYVVLAAQTCVLMYSTFLLRNDFEIGSGVLNYGLLMIVAIGILMNMQYFRHEVLHISGIMCTGLFRYSDKTMETEDMIKFRKHMKFQRQLLIALAVYVATIGGIVVLGPIIDEKLGMGFDGTFDENGVNRRLPVPLNYPGIDTSKIFGFLLALGMIFQSGVETTLIYGGATLLFATACQFILTEMKTLSVSIQTIPHRAAKKYCRIHNVSKKSLDLKTIFDDSEFQDCITDCLKENIQHYLEIYKFTKVLETYVKVPLLLAVLVITLAIGLTMMKLNEDIVRIGATISFTSVALGELCIMFLIAVYGEYYLTMSQEVNWEIYFTPWYKFSVKNQKLIRQFLISTRNELCIFAWIVRMDMEMFASVMNSAYSFFNFLNISKTLEEDELN
TcasOR149	MSFLFKFFAYLAEGEDDELWDTYYNFYGPFLEISFVFPSWRRSRIPVSLTVLGFFAFTFPVHLWLLTLGIEGVRDDFNLASMEFHYWMLLMFSMISIFLMNSHRQNMIDFHRRLSRDVGFHGPGRIYDEDYPMKLEHNKRKQLLQFLFLPGLVLVLAGATLILPYLSKMDGTVLYNSREVNMKLPIPLWYPFPTHEGILSIMAVLGQFMAAGGLSAVIMTLDLIIFRATQAMIFEYKVLRYAIDTLVPRAKRLYAQEYPMMDLESVKMSDDAFQICIGKCLRACVIHHQDINRLLNLYKVMLKWPGFMAYGFGTAVIGLSLINILSAKQNGDYENIVLFFGLSMAEVLNMLMMSVFGELISTESKELRQELYFIEWHNLNTFNRKLMLGFQMGLNNPVIVKVGGLVTVSLETFSSIMNTSYSFFNLVNAQ

TcasOR15	MSFLLKFIDELAEEEDDELIEVLKNEYGHFLFLAMIFPRWKKPLASFGLVLFYLSTIVLHHAMLSYAVYLSLLE<br>HNWEQVSFLTHLVILLSFAIFQPVNFNWVRRVVAHVHRTLAKDVGIYCSGRIYDDPVCIAFREKIRLEKKFY<br>MTMSAFCLMMGGILWSGLYISKSFSDIEQSYSSSGLSLKLPLALYYPFPTDRGVLHYVILGSQLLVCLVIGFLYLI<br>CEVLLINLFLKIKYELQVIGYAIDSLVSRSINAVGVNENNPQKDVLVIEDTKLQRSVEKCLKETIVHYQKILGLL<br>LIARANLDAPLAIVMMLGLLVIGISLLNMLAALKANYIGMFLTFGMLVCAEIQAQLLVCLLGSSITEQADILR<br>EKLYSIEWYCFDMKNRRILLNFQACFTKSFVVTAGGIAEINMVTFSWILRAAYRFFNLMRSTS
TcasOR150	MSFLMKFIDSLAENEDDALWDQLHKFYGPILEIAFIFPSWRRSKLPISLAVMSFYAFIFPVHIWLLTVGIQCVR<br>DDFNLASLEFHYWLIFMFSLVALLMMNGNRNFMISFHRTLTSDVGKYRAGRIYDEKRPLEWEHNKKKQLL<br>KFLSLPTLVLVLAGFSLLVPYLQKMDGTVEYNERGANMKLPIPAWYPFPTHEGILSLLAVLGQFMAAGGLAT<br>TVATLDIVVFRLTQSLLFEYQVLRYALETLMPRAKRLYTLKYPAEDMRKLRTNDEAFQRCIGKCLEDCVIHH<br>QDIIKLIKDYKTLVKWPGFMAYGFGTGVIGLSLVNMLSAKEQGRYEDIVLFFLLALAEVLNMFMLSTFGESIT<br>TESKELREQLYFIDWHLLNTSNRKLVLNFQIGVTHPVIIKVGGLVNVSLDTFSSIMNTSYSFFNLMNAQ
TcasOR152	MSFNSEDLYLNRAKFVMKYLGVWVPPENENFARKFYKIFMMSLQHLFLFFQIIYIVEVWGDLEAVSQASYLL<br>FTQACLCFKITVFQINMNKLKELLKQMNGYVFQPKNINQQNIIKVQATRIKRLLFAFMISSQLTCGMWALKP<br>LFDDVGSRKFPFDMWMPVSPERSPHYHLGYSFQLVTICMSAYMYFGVDSVAFSSVIFGCAQIGVIKDKIMSIK<br>PLGIYRNHKTYTKISRYNRKTLIECVKHHQAVISFTELVEDTYNSYLLFQLVGSVGIICMSALRILVVDWRSVQ<br>FFSILCYLSVMISQLFVCCWCGHELSATSEELHTILYNCAWYDQDVKFKRDLNFMMARARRPILLRAGYYISL<br>SRQSFVSILRMSYSYFAVLDQTNK
TcasOR153	MSFQALKHLLKMCAEKTPDLDNPYLTLRRVFIDFPYSKSMKIHTCITLLFHFLSLILEIHYLVTNFSFELSSRYG<br>CMMCLMTYVISVKIFVIMFAKPLKILEEQRELHFWKIGDSSHAMQQSVATEALQVKKQTYFALSCFVLLAVIL<br>YPVWGHVNDLFMFSQVYEKYFGDWSVIPYYFYVFTFMSSSFNSFQLPGVILYFTLHLNLQISLINEKITKISGE<br>NYCQDEVFKQLRDCISYHVALERWMARLIDLTKTAMPVFILLGALSSIAVSFFVLYSLENTRFILKIRLTVVAIC<br>NVLIVATFAKAGQRFSDKTGLIFDAIATCPWYSWNVPNRKIVLIFMANCLKPKTFSWAGITLNYQFAIKIVRT<br>SCSYALVLYKLRNGNY
TcasOR154	MSFSFVEKYQLSPETEKTMVTEYSYLLYVGGLLINYRPKVWIISIAQTSIFIGLITSYTIIFIISTAKSSNFVAFSQNL<br>NYASLCCICLGLYFAGLSHRSAFVRLMEIIHDDFYDYGDSFDNAEVAMWKSSLRTFKIIIVVGIPTYLIIIAVSIV<br>LGDYIDTALGYDSTDEDYLGEIYQKAPLNLWYPFVVTNMFLRVAVTLSQMTTAAILATTLATGDVMMLFLG<br>QTVALQLRILCLAATKMDQRANLMYEKGLARSSSGDKEDLDGCYKLCIKQLVQHHLIIKEFYKTYYTIAKW<br>PTAIAFMNGSLMIAMSIIVAMNGNEETPSTYISTYLLLVAEVLSMWLLCETGQNVNTWSEKLFMDTYEFNW<br>NGLSVPNKKMLLIFKENIKKPLLMMAGGLTPINRDTFATIMNTSYSYVNLLRASERRSND
TcasOR159	MSGDISRFKAKSTTTNESLMREEYIRKGIDENNGLFLIIGGMYTGYLPISILHAILTAVHIPLLLAAVIIGRNDYV<br>VVSETIHFIILLSLAFVISMRYLSVRKKLDNIFEAMGRGYYNYEGTLDPGTEKEFAIHLKESEKRKSVLKYVFVG<br>GCIGALICVSILRPVLQYYLKKYIKSKKLPHGLNGAKNTFIYYPWDSSNTWLNFIGYFLQDAYTLMTANVVF<br>GFVLMFVSTAESVVVQLDKLKLSLKRIKIRAAFIASINHEDPATNNNKDFRRALHICIKHSIKHHQLISRIFDD<br>FKSINYLLLFYLIGSLTFLLCMSAVLFAADDVSLISKATFVFFITSELVATFLVCMYGEHIAGMSSSLPMDLYNTE<br>WYHFSNELLIYYRMLAMRCTRPCQLTAGGFSQINRNTFLEVLKTAFSYANLLQASKQK
TcasOR16	MSGFHGFWDKWWYGDPPMDDKIFDAIHQEFNQILYFCGLFPDPRPIRRLLTFGILILNIVICVTYMYLLGATA<br>VLQNEDFFTASQTAHFASCDLLSLICIISLLLNRNRMIEMFRASAHNYYDYDDDAKISEMREEYMTTKKQKTI<br>ILIGLPSYLSLIGMVCLMSRTIDEYLGGGSNETNIDGVYQLTPVPMWYPFSIQSEPMHWIAVSSQAIAMFSTAT<br>SIGGTAAILVIFSQSISLQFKIIIYRIRKAEKHAYQLYRMNGGKKLKNVQLYSDPSFLGFYNSNLNKLAEHHSILI<br>RQFDSLYQVVKWPAGCALLL
TcasOR160	MSGKTKRITTKTIHLSNPYSSFKKVFSDFAYSKIMIFYTIATLAFHMLSLFLQIYYVATNYSVELICRYGPMMCL<br>AIYVVTAKVVGVFYYKTFTMLENQCLFVLWKTCNSSPTTQRLILNKSLKMNQKLHLALMSYFLLAIVMLPT<br>WGDLNELFIFSQVYERYFKFWAPVLYYFYISTFLWCSYYSFHLPGCILYLTLLLDVQIKLINDKITEIDQNFSQN

	EISETLRLCISHHIALKRWMSTLAKMVNSVMPVFVLLGALSTVAVSFFVLNTLQNTTMILKIRLAILTVCNFVIVSTFAELGQIFSDQNNSLFEHLIDCPWYLWNVKNRKILLMFMANCMKPKTFSWGGITLDYSFAISILKTSFSYALILFKLRGETIRN
TcasOR164	MSGKTKRTTTTRKINLANPYSSLKKVFIDFAYSKIMMFYTKATLAFHVLSLLLELYYVATNFSVDLICRYGCMICLMTYVVTAKVVGIMFSKPFKLLEKQCLFVFWKTYNSGPTTQRLILDDSLKMNRKLYLALMFYLLLAIVLLPVWGDLNEIFIFNQVYETYFKFWAPVLYYFYISTFLWCCYYSFHLPGSIFYLTLHLDLQIRLINDKITEIDQNFCQNEISETLRMCISHHIALKSWMSKLAKLVDAVMPVFVLLGALSTVAVSFFVLNTLENTSLILKIRLTTLTVCNVFIVSTFAELGQIFSNQNNTVFEHLMNCPWYLWNITNRKTLLMFMLNCMKPKTFSWGGITLDYRFALTILKTSFSYALVLYQLRGETN
TcasOR165	MSHSNPLEAFKLNTFFLKALTVWHVENPTYRLYKIFVVFSFAVTFFSAWICALVNYNVSEISENFYYLPAMSTGPLKYAIFQKNFTNIVNLTHLLETQYAKIRTENQKKIFDESVIFERKVMKNFAILIIPTCVAMFIVPYFQDRREMPLIVWFPFDYKQPVVFDLVYFILAFACISIAYTNVSTDAFFYTCLIQIETQCEIVSDTLRNLDKIVTNGFRNVAESRKIFIECIEQYNVILRYTKIVSDTYQGILVVQFFCSLVALCLTMYKLSLADPGSQDFIKYFVFKLGVISEIFMYCYFGHRVLEKTEDLYFAIYEMHWYDASKQIQNEVFIFMGQLEKPIVFYVANIFSLDLDTFKKIMQKAWSFFTALKNMHDIRNN
TcasOR17	MSILQINNNRIFNLQVMATLFGFRQILDTETVTLPGRHNVYHGTLLVFLIVYECIISAMMCLNGLYYCIYNMAESILYFCYAVNILYASYKMYIVIKHSKLLRDCLSTTQFDFTSYGLRGRRTLDVARNRSIKSTYIFVMISFLVFICYAACPLVFRDTLIVMRNHDGSTSSYRLNVLNLYLFVSEQAYDAYFNVFYIIETFGSSILFFFVINYYTAVKTLGFTLSSQLRMISTALKSVGHKSFHSPNIDNINNKIELHNKNNMDVYNELKKIIIDHQNILKKYNEFLSIFRPSMLQEVFVLSYSIIVLWFIFLKSFIERDFTESLGTMAVTSMKASFSIPYCIYQMYMYCYIFDTLHNEKDSIIFGLYSCNWTEMDMKCKKLILLTMRMNNAHQPKLQYTRTRIINMEIFYQTMRVCYTIVNVLINWKKE
TcasOR171	MSKAKKSAVGTSSYKELEGLERAEALKKGYDENGGAYIRLGGQYVVSRSEIWRPVLFYADTALAMFELVTASYFSVLQGDMEAASECFHFIQMIFNMMVISANLQYYRKNIDELFTAIGAGFFDYGDTIDPQTKEKMDKHMMDMRANKKFRFKVFVLVVWVLGGCMFIKVIVAHFRFGDTIDGEGGSVSRKHIVAIWLPGIDEWPSYIAMVVAAYLCQVLIMNSIWGFVLPVICFAEELNAQLHIVGIGLRHTTARARHILYRKYGEQKSGNLKFKYEESLREALKSSVQHHNVILEACKCASTLLNLPLMTVMFGTAVLLCMSGFVMVEDSVPIIANIISLLFIGGEVIYAYLFCYYGEMITATSLEIGDELYNDDWWEGRDVFRPYMAMISLRSNRPIKLSAGGFTDVNNAAFSNIISTSYSYFNLMFTSKS
TcasOR172	MSKELFSLPGPNSVINVNIGVLKVAGLWPTRPYGLFTIYTVWIYLTQWAVFALDFMSLFYYWGNLNMITAVFCNLTSITAGIIKMTHFFVYKPKYYMLVNKLDALVDSQQKITHPNVDSKSILLQTSKLNKYSTYIIVTYGNLVGVPWIVLPFVVDSGDTERTLPVVEWYGITQDKSPVFQIGYVLQCLTIMYWFFASWGLDLFFGALMIHLAGQFRILNNRIANVGREVDPRLDVSSTLKIEMKEINIDAATRTTLVQRKDAELYSELRKCIMDHQEMISFMNDLEETVNFVVLVQFMAGTLVICVNLFQAALNVQDFSSVLKVCMYMFELILQLFIYCWCAHDVMVESERLSTSAYFSEWAGAPRRFTTALHILMARAQKPLTVSAGRIYTINRSTFVSLINASYSYYAILRQMSDR
TcasOR174	MSKFNEYWDNWWFGDPPMNDKVYDAIYEEFNIILYVSGLFPDPRPIRRLITLGILIFNVVNCAAYVYFLGVTATIQINDFVTASQTVHFASVDLVAVTCMISIVASRRHMIDMFRTIANKYFDYGDDFEIPEMVEEYRTMKRQKIIILVVLPSYLALNAFVCMMGRTIDGYFGRASNETYENGVYMLTPEPMWYPFTIHNELMHWMIVMSQATGAFALASAVSGSAAIMVLLCQSINLQFKIIIYRIRKAEECAYHLHRKNGGEKLKKSELYSDPSFMGYFNSNLNKLAEHHSILIRQFDSLYQVVKWPAGCALLLGSLLIAMSLLALLSGDGKPSILLLAALLIVAEVMNMALLCGQSESVQELGQSLHEELYNMNWIELNPAAKKTMMMMILQSKRPLVLMAGGLQPLNWEAFSGIMNTAYSYVNLLLAADV
TcasOR175	MSKGVEIFYKGQKAFLNILSLWPQIERRWRIIHQVNYVHVIVFWVLLFDLLLVLHVMANLSYMSEVVKAIFILATSAGHTTKLLSIKANNVQMEELFRRLDNEEFRPRGANEELIFAAACERSRKLRDFYGALSFAALSMILIPQFALDWSHLPLKTYNPLGENTGSPAYWLLYCYQCLALSVSCITNIGFDSLCSSLFIFLKCQLDILAVRLDKIGRLIT

	TSGGTVEQQLKENIRYHMTIVELSKTVERLLCKPISVQIFCSVLVLTANFYAIAVLSDERLELFKYVTYQACMLI<br>QIFILCYYAGEVTQRSLDLPHELYKTSWVDWDYRSRRIALLFMQRLHSTLRIRTLNPSLGFDLMLFSSIVNCSY<br>SYFALLKRVNS
TcasOR177	MSKLIEVFLGNLWTQRFTFARMGLDLQPDKKGNVLRSPLLYCIMCLTTSFELCTVCAFMVQNRNQIVLCSEA<br>LMHGLQMVSSLLKMAIFLAKSHDLVDLIQQIQSPFTEEDLVGTEWRSQNQRGQLMAAIYFMMCAGTSVSFL<br>LMPVALTMLKYHSTGEFAPVSSFRVLLPYDVTQPHVYAMDCCLMVFVLSFFCCSTTGVDTLYGWCALGVSL<br>QYRRLGQQLKRIPSCFNPSRSDFGLSGIFVEHARLLKIVQHFNYSFMEIAFVEVVIICGLYCSVICQYIMPHTN<br>QNFAFLGFFSLVVTTQLCIYLFGAEQVRLEAERFSRLLYEVIPWQNLPPKHRKLFLFPIERAQRETVLGAYFFEL<br>GRPLLVWIFRTAGSFTTLMNALYAKYETH
TcasOR178	MSKLKNLPFITILWREFKFLSVMGGTYGFYHTKAWTAVTVINYIVMYSAMLFTMSVLVYTTYLLRDNIGYMS<br>QALHLLIVGCVTTTASFTITINRYKVEKFVVFFEDPWSLCEYSRNDFFEELMKETQKKKTRLIVTWILIYGICGV<br>IGLLQSGINTVFGTQSELTDVNGAWVILPFIMWWPEDITASTGAWMRAFLVQSLYLYFCVIMVISGVVFAFSA<br>VERILDQVKLLIYGIKTLDRRAKDMFQQKFPGSDMKYMEKEYDDCYYECLVQNVKHHHKMIKWIETFLDM<br>ASLPIAVPFYGGAVLLGMALITITEKDDPRVGPKCLAACLAFSEAYNMYLLCDVGQRLENLSQELYDTLYFSR<br>WHTRSPKVKRAIQIMKIGCQKPIVFTAAKLLVLNMSLFADLVNSAYSIFNLKAASEKFEDK
TcasOR183	MSKNLKEIPPVYLKVHLTVLQILGIDILPNERIPQTLFYTYSVLLIATMVVFTTAECLDLVLNYEDIYKLTFGLC<br>CCVTHVLGAAKMFLMLYLRKKLWGYFTTLENGIFKPNPCRGGAEEFEIVTSAINMCKRQGYVFYVLTVGVT<br>GGQGLYAALANLPYDKHNYFDGNVTVVVNTKQMPYATWTPFDYNDSPLYEIMFAFQIFSTTLYGFYIGAAD<br>AVICGFLMLIKAQFLIVKRELETLVERAQRAGNPDRGDFGGGINRIEMLDDGTQVFVEKCANECVYHHQEL<br>IALCEHAEEDFCYLMLLQFISSLLIVCFQLFQLSTLSPGTFEFFSMACFLLFILFQLLCYCWHGNEVQFVSGELS<br>RYAFSINWIIMRESPKKTLLLLMMRAQRPCYFTAGKFSLLSLQTFMTVVRGAGSYFMFLKQMNT
TcasOR184	MSKSRVSDYKAYRGLTKTLLTVGGLWPYSNSNIFYRTLPYIQIILNLGMALAILGFVRDHFSNIALVTRGMGV<br>MTSFLTTILKVTCLVINKKDLMELHGNLDPYLNDLLKNSPSTEVILKDINSFKFLSWGLTLFAFIILHCHQHEIE<br>LINYPLIYPSVYPWKIISNGWIYKMNYVFETLASLILFFVTASVDSLFTLYVFQMVGLLREISYSIRRLDEKNVN<br>RDSVICKCITQYEKLMRCREILEKIYGPVILWIMTTNAVVLCTLLFQISQMKSISVTRGLLFTTYITLKMIQTFMY<br>AWSGSCLTDESENYKDAVYAAHWYGNKRFMTSVIIMLAQRPLTLTACNFSTVSLKIFVMVLNTTVSYFFLLQ<br>TLDYQE
TcasOR185	MSLSTRCLLKDFCKYVYYAGAGNFWYEDIYKETVPYKMYVVISFFTYTVMIFLENLAALFGKLPEVEKNSAV<br>MFAAIHNIVLTKMFLLLYHKRSISKLNCEMAAVGENLEEASIMRRQFRKMRLGTALYFISVYLSLVAYGVESA<br>RRTIVEGAPFYTVVTYLPDYDNTTVLASFLRIFFYITWLYMMLPMMSADCMPIAHLITMTYKFVTLCRHFDQI<br>REKFQINVKIMAKTEATEILKLGFIEGIKMHQKLMYLADEIHRVFGIIMALQVCESSAVAVLLLLRLALSPHLD<br>LTNAFMTYTFVCSLFLLLALNLWNAGELTYQASLLSNAMFYSGWYFCDFEKDWCRDIRRLVLIGCAQAQKP<br>LILKAFGVLDLSYETFVSVARMTYSVFAVFYKRGD
TcasOR186	MSMPDSELRTLLGSGASIRQLMGLWWPRGRRGRGRACSAAAAAVSLASLAWLPTFSGLKLLIDPPPEIEEIA<br>MCYLLIFACTGFFSKAAFLIYKGETVWKLLDLLSETRRLHRNGESNDNIRLSYQQQSRRVYLYMQGAICVAF<br>VFWVSTPLLVRAFLASDEDSPESYRLFPVPLWFPGNMYLSPTYEILYSVQSFSVLVAAQSTVCVDIFFFHLMLM<br>ISAEVQVLNENIALMEKVNLKSEKQEDHELRLNIKEKAEDLSFRSVGYSTGKALTEEVSDENMCVQLVKNIQ<br>HHQLILRSVVLLQDIMNLSVFILLFVNMVDLCSCIFVGAVLLQRDGNVTKALKPLSTVPPLLYETGMYCIFGQ<br>ILSDQSEKLTDSAISCGWVDCNDRFKRDFMFLLISAKKPLEITVGKTSKLSKQMLVQVLNGSYGLLNLLYHFQ<br>SIQ
TcasOR189	MSMQYSQDFKYALAWNRTSLKIVGLWPEDDDGIITKSLGWFCACLIIIIIYLPQSASVYVYWGNMDAVIECLS<br>VNGPVFITIVKIIIFRYYRRVLKRVIDTMAEDWSSSRSNEEYAVMLKTAKISRAISVTSTIITNSLFVAYIFFKIWEG<br>LEMSKRTDLDPRLSVGLLHPAYFPYDTKKIKFFVPTWIAQLVATLFSMTAYAVFDTFVSCMVLHICGQLAVVG<br>ISLQNLINEDTNSDPNYFWSKFSKTVERHEKLNELANVIEDSFSSILLPQMIICTVTFCFQGFAMITSFIDPLAG

	KVSILEMLFSIVYVFYTVLHLFVYCYVGDYLSFESSIIGQSYYKSEWYKLSQDKSRSLMFIGHRARRPLKITAGK FCAFSRNLFIRILKTSFGYLSMLLAVKRDKSA
TcasOR191	MSMSVWALTIGGLRSSLGIEMTYACLTIMTTVQHFHMYRNRTRTEAIIATFQEIRNTYQKGTDIEFKNYTRFM WKVVKVYIVMIVGICSAMSLPFFADLVVWFVWETPTAFRIPIGMDSMVDKEPVRDATYFAVVLFSNCWTILG GVTQMGVDTFLFVSCYSLSSMVKTFCKQLKVPPNSTPEETTVHIRLLAAHQQALYKLQTEIRRTFGFPFFVQN LLGSFCICSLLYVMSENGATLLSQFIYVFNMIAVLMILASTAHVAQHVKNTTSEVFEALYEMNWYTLRPSDRK YLVTMLGVARNPLCIHFYGLLPLDMENFMSMLNTSYTYFMFLKSIG
TcasOR194	MSMTRSKYFQDSDDPFSFIRKIFIDYGYSKKINYYNRVTFTFNTCSILLESYYMITNFSLDLFVRYGGALSLMLY HVVTQFLVIAKQKSLEQLLEESKSYFWKADIFNSSVKNQILKSCNHMQRKFCLLWTPFVACGIVLLPVWGDF TESHIFPQVYKAYFGHWSPIFYYFCISSYPFAVYTSIRLPAIALYLFLQAHFQIVLLNQKILQISKNNDLDETTIFE NMEYQKTIYRNLRSCISQHVALQKYITRILVSIQKAIPVYFCLAVLCLIAVIFFVLNNLNMSASNHFKARIFVSG VCGSLILYTFTEAGQLLADTTGDIFNTLMQCPWYYWNIKNRTVFMIFMLHSLNPLKIDWGGFTLGYSFGGA VIRTCCSYAVGLYNLRESKY
TcasOR195	MSNGNEGIYFGTIINLMHCFKIWSPDDSKRTISFSVYILVPAYVFFFALSCIEIYHNWGDMLSTTDAVNTFVIYL ATSHKYFRLIYHEKDLKKLMKMVENNFSVPVWQNDALRNSIVKSYVQEVKKLTILWTTLCFTTLCGFMILPL VDGLFHYYTTNVTTEIEWKLPYRTWTPFNDYGAIVTVPLYVYHMFMGFVLIAEIPAFDTIYFSLINHSCAQLK IIQNSLINIVSISAQNVLSNEKNGVTFDTMLDEHYTAEENKDQSSLENKMNNGLNSYTPSGSYDSMLNLLHD SELDKKIRKNVGELVNHHEKILEFIDGVEAIVNAVFLTQFLCSATLFCLTGFQLTVILKEQQLARFLNMMELL GAAIFEMGMFCYYANRVMDEGINVGKAAYDSQWYYASKDYGNSVSIIMARCTRPPKITFGKFADLTMENFA SVLQISYSYFTLLTRINE
TcasOR197	MSNNDETDYFKIPLKFYNWSGVRITSKKIPKIISVYILYPMMIVLYGLMIINIRFNIDSLAKFIEVGISVSTMTIIA LRKTLLIKNGSVYEDLLETQKLYWAYNRFGQAFESTQRKRMYFCLLVTKFIIGYSCVSLSYHSIIAPIIMHEMIL PQPCWNPGNTTIGRNIIFFVENIFYIEGTSNFVIFDCLYFLLATNLRIQFALLRKELNSIDFKEDSEEKCFARLVK CSQYHKLLLSVHRKINTIYSAFFLLTYVFTITSTCTLMFVVFYMEADAALLGKSIFIVIILNTLLVMTFIPAGELEI EGEKLAMDIYFMNWYETSSLKIRKFILFWLMRAQIPLQMTGGGMLVVNRPLVLQAERVSYSLASFLANL
TcasOR198	MSNQDDVGDDVSVRRIPRSAQKRPQITVKNEKEFDGGCTDYRKYIFCRLMYIDDGLVSRGLTLPLIIIMVHTV TMEVCSFLSIFGSSQIKDGLDCVRSFLLGNLVTMVLFNEFANRRRLGRLHVFLDKSMRTLRTGLPEEEEILKRA RDQASSNLKMYIIIFSNNIAPMVFAQPLGEWFAGRSWKRLPIPWSFPPSDTDLAFLLIFLFQFIGVIMANFLAM VFMSFSSITIQITALFDVLLLSLRHIETRAALRSKLEGTDYRQSLYNSLREDITFYEQLVREVGSATPHLRNTFLA FSATVPMIMACEAYPIMLGNFAIADLVKSSVFLAIQFLCWAQTCMRLETMTDQHAAVFQALYNTPWYEADL KYRKLIFMSLTYAAPSKYIKARLSNEITATTATFYSFVVSSFNWLNLIRKMS
TcasOR199	MSNYIFKPFHETYRIITFTMIAAMIYPNPATEKRRLIYIGLMLLSVIPLAFMIVTEMYEFFMASDLNNTIRHSTVI GPFIGGFVKVALMYYKRRQANELVSEINRDHLAYNGLKGEDREIAASSIRNCQIYCELGWTLIVMSCGLSFPV IAILLKIHSFTFKLDSTKHMIHDINNPFTDDPEDRFESPFFEIMFVYTFFSSFIYIINYVGYDGFFGLCINHACLK MKLYCRALEDAMRSDSRRHEKIVAVIEEQRRTYEYIALIQDTFNIWLGLIYVATMIQMCTCMYHIVQSFNIDV RYIIFVISIIHIYLPCRYAANLKCMAAETPTLIYCCGWESVSDLRIKRMMPFMVARSQVIVEITAFNMFAFDME LFVWIMKTSYSMFTLMRS
TcasOR202	MSPHDSGTFARIRKIFSILVYTSTVVLSMAELFFNYKDLETVIRATESFFTQYGLAWKIAVFVVYKTELAQIIRLC DNLWPLDEFGTGHNFQFLHKFLRRFFLLYTGNLALLCTQFAVTAFFDDQFKSVMVYYGEKESRSQIYDNFVF TLQVIYLYVGCFVVAGFDCFFFYLLGHAVTELKMLTISFSCKEIGRNWGYEERFKCSVKHHIHVLELLDKINK VYSVMLLNQHLCSLFGICFGIFLMTKDGIPPNVDHFSKWSTYIFTFILQVWTYCFAGDQIMHWSLKIPDEIFY DNYWNKYSLKNGLNKIIAIQRGQKAAGVSLGGFAMLDIESFNVVIKNAVNFFMFMDKMYKRE
TcasOR204	MSQIDLKEAFKQNIVLLKAMGLWFFQNERFYKLFKCFVQGSLVFDSTSLIIYVALNIRIKNVTDTIYSLPGSLEV VLQAILFRKNFHLIRKSLNNLKQKEFQPKNDTQEKILKDSIALSRRVFYSFFWLVFVMIGMWMVLPLTKKGK

	YLPTKYWIPFDYRLPVVYELLYVFECSCIIFHAFSNVALDTFFSIAMIQIGAQCDVLCDTIRNMDEQEKTNTM<br>DRILIECVHHYRLIEDFAKSIATSFKEILMVQFVCSSLMLCVSMYELSLSEPMSGHFFQVLLFQISATNEIFLYC<br>WFGNEVIIKSERLFYAMFESKWYDSAATHRKNLMIFAHQVQKPISLLVWNIFPVDLKTFGGLLQKCWSFFVA<br>MKNIQEIQE
TcasOR205	MSQLKARTFFGELDDPFLLIKKIFIDYGYSKPVNYYNRLGFILHTCTLILENYFVVKNFSLDYFTKYGCVVILM<br>HYFLISQFLTILNEKLFKELIGERGSLFWKSDSSSPRVKNQIVKHSVKFNRKFCFVLFWFVALGIIVSPVWGDLS<br>ETHLFPQVYKTYFGIWSPVFYYFYVSTLPLVAYTAIRIPAIALYLILQLDLQKILLCEQISLIPSSDQVRIYKKMRL<br>CISQDRELKKWIAKIRLSLKRNMSLYICIAFLCLITVMFFILNNLTTTTDNVTKMGFFVAGMCGSLILYTFSEAG<br>QLLFDYTEDVSNTLIQCPWYCWNTKNRKLYLMLMQNCQKPLKINWGSVTLNYCFGGSVIKTCCSDASIFFK<br>LRNDK
TcasOR208	MSQRPDPEGPPSKRTYLSKGLKLKSSVRLDAWRHIGYRLLLLGMTPENFMEKFSVRHALVYLIFFLTHSLYSA<br>HELTVALFFSRSLLERVTHGYILTYIFTFNLQWYYMLRHVGNFHRHEMSLENFASTQAHLDFAEEVFNKNIN<br>AFLKYLFISMLWWATNTTTHVFGPLVETVLMYVRSGDFKLATVLPQVFSLPMWAQVIMYIHNATATILLFVY<br>CLASYLVLGTRVLKVKTQCDILNEALRRDYDEESNIRSYIKDHINIIKSAKLLNGHMQMLNGIIFTACYLEFAT<br>QMFALTLFEPSGAYFFALAFDLMSIMLILVMQCWFASIVTISLQSVTDAVYETNWYRRDKDNSLNVLMMLQ<br>MAQQDYIQRIWFKSFKIERAAILNLIRSSYAVYTALLIFQE
TcasOR209	MSQVQHLKFNVLTLKLIMLWPPNGHMTLNYHLKSLSLLIIVYLSAWTCIYGILRPLFTESDGYDVLIQRAIATI<br>DFIGCIYMRYCFLDKIQNVTSLIRELPTFEKFCGKVEIETTEKKVQKYSKAVTVYWFFGNLMNCLAPLHERTK<br>CENLRKSQLYIERDPCGLMARCFYPFDVSKKLFPMAYFIQVYTCVVISYYVIVLTMTLVGIMMHILTQLKNCR<br>NLIHNLHEEITERESLKKKIQRIIVYHIKIINLTMTRKHIFLNFTVTILKLSFLWPSNDNYDQWRLVKDASLIVSL<br>MPCALPILAHFVLQITGDVYNMVTITENLIALICIIGMIYMTICFVKNRKLVKTLVKNLPAFTKYSKTTDIILTD<br>KKANLYTKIFVFYGVIGNVVYMIMPYLNIEKCQQRQNNDVPCGLVTRCWFPFKFDYSPVFEIVFVHQFYTCL<br>MVSVIILDLTMLICGFLMHITNQLKHLRGFIKRFDCSSQKIAEDVIYCVKFHTAIITYSEKTNEAFGTMMMLHI<br>TLTSLVISALGFEILIVDNFNDSLRFTLHLLGWLVLLLLICYYGQLLIDESIAVAEDIYYVPWHLAPVDVQKDIY<br>MILMRSQKPLTLNAANIGVMSFPTFLRVISSAYSYFTLLLNIKS
TcasOR211	MSRAYIAHVGNSLTESFSPKLLRKRTVLFFLSLRSRGRALEMPEQKVQLQRGARICDVLRHNVLLLVATGAW<br>PPTTRRWWRPLYPLYTASIYFSMLATIAMGFQFAYQSWGDWDSIMLTFVNTFTLIGGAVKLAHFSSHVDAYR<br>RLVTALRDVIGTQWAHCERDAALMAAFAGSHRKALWLTWAPVVYLNILGPVWFMMPVVAWASGAPGRQ<br>FPFANVRGVLKTNFPLYVAVYFVQCHSVFYWNFLSFGLDIFFVTCMIYVSAQLHILGKRLSNVGRGPNVDQN<br>GIVDEKQTKLQQFGQKPYKSLEVDRESNEMYAELVDCVKAHQHILSFVAVLQGVMSPVAMAQFVCSATAA<br>CITPFQATFNPEGNSIFKCLMYLPMPAFQIYIYCWGGHEIIDEGAALSASAYSCAWMGAPRRVTSAMHVLMC<br>RAQKPLTLTAGKLYPVNRDTFVSLINGSYSFYALLRQMRGH
TcasOR212	MSRYGKIEDDELVNSIDIWYLKRSGLWEVFNHYREHGVRNRRFTLWKIITLILFVPIGFFSLCGPFFTETDLEG<br>MTLVILNPMTSSQTVIKFAILWYGIETQCRVLELFKRDFLTCVPPSMQAKASEILTKAAKKANKLANLGILTD<br>VITVSFWNILPLLRSEYFRIELGITAFGTPLRHNKILGFWYPVDYDETPYVQFVYCYEFLSCVWAGFVIALLEGL<br>VIHLVILLTANIKVMHHLLEELKTSNGTLNSETLLTYIKDHQKLVKISNDMRNLYNMMITMELSTGLIILIITIF<br>NFFLSSGNGDLVIMFKFMVYLMYTLVEVTVYCYIGSDLETTSEDLGFAAYSSQWYKVGKKFRKTLQMLMVRT<br>RYSLALKFGRMYPINLMALTNILQTAYSTSMLLYRATSQDEQKEEAQILM
TcasOR213	MSSKNYLSFPTGFLLGTGLLPESKFSRKIVSIGVFTPMTLFLVYLIVKKARNGENKDVLLWAELFESLTTCAHIL<br>SRKYVMYVHGGLISQIIKERGCYFWNYDIFGPKFGQTLERKMNICTKIVKIVVSGGVVTIVLFCLTTVFDKSKV<br>VPLVCWTPEDSLQTGIIYAMEVLIMFEIMWALLSIDCFYLLICTDLRIQFILLQRMIKSIKFGSNHDEKSFAKMV<br>HCTQHHKFLLHFHAKLNTIFSSYFIVLYLVTVACASMHTYIILFKQVANLDISDWLKVRFRSPGFGDSIKSACY<br>LSGMLFQVGLYFVITSNVEIEVVEIYNLNWENTGSVKIRKHVLFMLMKSQEELSVTGGGMLHVKRNEYVGL<br>VRLAYTIATILGGMT

TcasOR214	MSSLIQESLHINLRVLELLWLYTPKEQSNFHKMRSCILFFLLMFHVPVLGGIHLIFGINDNPTKLADNALTIAG<br>VSCYALKLWPLIGNRSKIKDCINYLDKPIVELRENQKVIFQSCSKICRRNSKIFQIYMITALIGFVIKPFFFEDKSF<br>PVDIWLPKALRDRLDVYWSFYFYVSLGVAYPVIASGALDPLIPSLLCLATGHLKVLNDNLEHLDEYSNEISES<br>KNCVLYKNVQTCIKHHIEIMNFVENHQKCFSSMVFCQFLGSPLILCFTCWNMSILQPFTFKWFESLAYFLILL<br>VQLYFYCYYGTNLSDECANVTTSIYMGKWYKYDVKSRKALILLMERSKKPTIVTAAKILDLSLETFTIILKRSY<br>SLLAVLKNQN
TcasOR216	MSTENTQLDADIEYNLKLTKWILKPLGVWSIVTKDYRFISKLSSVFIIILNSFIMFITFMPCCVHMIYRETDPAV<br>KIILMGPFGFCLTNCCKYYFIIFRSNIITHCLENLKSDWARPKTKQDRLEMIKNVNIGHNITKLCAIFMFSGGV<br>SYHTIMPIWSGSTVNEANETIRPLVYPGNEIFVNCQETPIYEFIFILHLTCGMVMQTITTAACHLAAVFASHVC<br>GQVDILKSQLQNLVDKESLKIDGTIENRIASIIQSHVRILDFSTNIEKMLREICLVEVGASTLIICLLEYYCMTEW<br>SNSETINILTYLMLLVALSFNIFIFCYIGELLKKQCNSIGESTYMINWYKIPRNKGKQLMLIIASSNNQRKLTAG<br>GMMELSLRSFGNIIKTSVAYLNMLRTVTE
TcasOR217	MSTSESPSSRFRELSKYINSLTNLLGVDFLSPKLKFNYRTWTTIFAIANYTGFTVFTILNNGGDWRVGLKASLM<br>TGGLFHGLGKFLTCLLKHQDMRRLVLYSQSIYDEYETRGDSYHRTLNSNIDRLLGIMKIIRNGYVFAFCLMEL<br>LPLAMLMYDGTRVTAMQYLIPGLPLENNYCYVVTYMIQTVTMLVQGVGFYSGDLFVFLGLTQILTFADMLQ<br>VKVKELNDALEQKAEYRALVRVGASIDGAENRQRLLLDVIRWHQLFTDYCRAINALYYELIATQVLSMALA<br>MMLSFCINLSSFHMPSAIFFVVSAYSMSIYCILGTILEFAYDQVYESICNVTWYELSGEQRKLFGFLLRESQYPH<br>NIQILGVMSLSVRTALQIVKLIYSVSMMMMNR
TcasOR218	MSVKINLLEVFDNVKFLLKFLGCWTFPEENALYKIHKYFIIITCFLYATTCNIYGLKHAFIDPNKAYETLPIAVG<br>TTEGVLKSYLFRRNFAKVTESWNQIQQQEFQPRNEEQKTLLKNYIAMTKFLFKIYVVGVYICCVSAIVISSWM<br>RHNELPTDHWIPFDYHRPFLYQYIYVHLTVGLYLCSFLNCALDSCFYLSLMHITAQCDMLANTLRNIHDLEK<br>LNAAHRVTHCKNKNQVMNEILTECMEHYSLIKKYTNLVTDCFSGILTFQFIPSVGMICICMYKISTLDISSSLF<br>FQFIFVEAGSITQIFFFCFFGNLVTVTSEKLFYSTFESQWYNSSLKFKKNLITLMMTFQEPITFFGWNIFAINYET<br>FKSIIRVSWSICVALKSTQDI
TcasOR219	MSVNWNRKLNSLNHTRIAFDVGKYKDGRIYSHKACIRMEKELQKETILYLCIPLLIIILGGAILIVPYGSKLVR<br>GYGMMYTACGVDLFLPIPLYHPFPTHEGIHHFLALISQVLLVFCLMNGIIAGVLNFLQYSQRVKLEYRVLSYS<br>LDTLFARSKRVYLRHYPDKKANFTIRDPEFQHILGSLLRDSIIHHQTLVDMMNNYHGLITYPVAVGYMTGAG<br>GIGLGLLSILRALQKR
TcasOR220	MSVQGEVIASKAFKDNFGRYMVWSGMYSESKIYSGAIHVFFLVHTVFLAYTVILSLDDEKLMGESAHFTAFR<br>VSAFMLMINAVLNKDNLEILFIKLGQNQVHDYQNTLSDQCKKEIQAVRKNCDERKDFYGTNFLRTVSAAL<br>VIFWVRSLMEYYRGHMDNPKSDNGVNKNLPVPTYLPYESHDWPGYQFALLSEVALVMMSYFLVLGHDCSF<br>ICFSEEVLRELEIIIITLREVEQRSDHLRKEMLYKISQKESVHICLKHSVIHHQKLIKIFGSFKRYCFYSLFFMLSG<br>GAFLICLSSLMFTSEKISHQDKSVFMMFLANELFHIFIFCYYGEHIMDRSIEVGNSLYNSSWIRIAQYVKPAFIM<br>VKLRCQVPLSLSAGGFITAGFDTYGNVLRTAYSYLNLLQATN
TcasOR221	MSVQKVSPDNLWLSAKICLHIFQYKAIKIILKILSLSIIILTCIQTFLYLKRFDSAYFMKYLPVYAGSLFILASIFCIE<br>HISHVILSTVEEFEFWDYADSKPEIRNWIKWEALYINTFMVVDAFVAYLSGIFHAIPLDEDYEIFYPLPIFQEFFP<br>DWTNVLGWLYRSSFLIVPVVMTAPSLMIIYFTSRLRFQMFLFMDILENISEGYDISEANDLIENSTYQKEIKERL<br>KTCIKRHNEFLSAGGQVMKNGQLFILIMSAAGVILGVSIIFFLFSFEGSFEKRYPRLVTLVISTGLTFTHVIIAGQ<br>LVENIATRLYEILHFMDWHSWNQENRKILLIFMHNAQQELQIKFLDEVAVNYQLGISIGKAVYSMISVLSSFK<br>NLEESYN
TcasOR222	MSVSNLKFEALFKPTTMSLHMNRSHPSIKRNKIWLLQFISLMTLTAFCATGLITSLLFHDLKFGKYMEASKNG<br>TIAMLSFTTTFKYSLLLYLQKSLNRLIAKIDMDYEIAKGLPPQEKATVLNYAKKGVIVSKFWLFTAFAITFCFPL<br>KAFIIMGYRFFIKNEFRLEPMFDMTYPEPIESYKTSFPVYFILFVVFFLFGCYASSLFVAFDPLVPIFVLHACGQL<br>DLLSLRITKLFSDTKNPRIIAKELKVIISKLQELYGFVNFIKVNFSILYEYNMKITTISMPLSAFQVVESLRRGEFN

	IEFTYFFFGCILHFVMPCYYSNLLMERSENFRFAIYSCGWENHHDKNIRQMLLFMLTRATEPLGIATVFTNNS<br>LDTFAEMCRQSYTIFNLMNAAWA
TcasOR223	MSWGSEEVEQLTWKYSAKSVLKWNIRILNLVGLWPLTESLLFRSSTAVIVMLCVAHIAEAAVNLGTLRGGLQ<br>DFTLALSNVSVVCVGVLKLTFFLRHERSYCRLVRSLDVLVDSQREFVQGQPPLAALFEATQKRTVRVTVGFLV<br>YAITQLVAWSFAPMIAAPGTWRLPFQQLPLTDETAFLIYELSYAMQVVSIIFIALINCQMDCFFMATMLHTAA<br>QLRILSARIMCLKLQNEDIPAVFRSNEGEEVQASATHDSTYRNLCLCIKTHQELVRFVRHLDQVMSPIAMMQ<br>LGLGVFNGCMLIFPAAYSAESDALVKCLAAVPTISTQLLLY
TcasOR225	MSYNIKLTKDDRLKLLKIMASDVFQSKTVKIILIVVFLVHAIANSLTIYFALHVSDTKQFISYASVFFSEFYPMLA<br>ILTIIFKGEVVQHLTDDINIWTIDGASKKLQSEIKLKIKILTAFVIINSFSVVIGGFCFVQQLSDDVNLFFAIRLIRD<br>YFPNHSTILEFFYRMTYPICAYLMAVHAYQCLYYTQHINFQLQMFTEIITELTDLKTISLPENRLFYNKKYQTVI<br>EQRLKFCIKRSQEFIKVCVTKNKEIGSLIPGFAICGLFLGIGITFFLSTGKFTTEYYLRMGVTSICGLTTFSALIWS<br>AQTTETMINDLVMVINKVSWYNFNQSNKKLYLTFLLNTMKERKIKFTEKYSVNYQLGLAIVRGIYSVISVVAS<br>KRHH
TcasOR226	MTAGMIAARIDIDVFLEAIPTVLADVICGIKMFNFSVNAAKMKKLLLTMEEDWKIYASGPENKILNEYAHFG<br>RKVTIYYTGALYGTLAPLVLVPITPLILDVIAPMNESYPKHLMFQQIEFLVDADKYFFPLFIHNYMGTVAFLTIII<br>AIDTMLMVYIQHGCAKFAILGLCLERIATNADQNIDRHTSEFDDIDYREIVKCIVIHNRAIEFANLIEEANHLS<br>FLIVIGINIIMMTTSALVAVFKLAMNETEIAGRFAFFTLGEICHIFYSSWQGELILKHSESIFYFVYQANWNNTS<br>TRSQKLMVPLLLRSAIPCRITAGKMFEMSLKSFSMIVKTSFSYLTVFASMRV
TcasOR227	MTAMLNFEEKPDENIFKNDVLWLSRKLCFDYYNNKIVKILLRVLSIGVAILTILQTCLFLYRFDGRYFIKYAPLY<br>AGSFFICVSVEYIPLSLTITRYFQSITFWEIDSGGPDIAKKIQKHAMCTNVCLVSCTIVGLVSAIFHIIPLEDDDEL<br>FYPLAMFEEFVPQWKNLWSWMYRLSFLTVPFSMPIPVYIAIYVITKSYFQVLLYLPFLENLNTGFDTTSNHLLI<br>HDHNYQNTIKQRLVFCIKRHSYFSREMNSLNHRMYVTIATFSVMIIVIGVSVFAFLFSFQGTFENRYIRITTLVF<br>TIATISIHVLYIGQLIEDACFRVYQTLVMVDWYYWNIENRKLYLVFLQNAQIIFKIKFSQNISISYQLGVSILKTIY<br>SMISVMSNLRNVDYYKN
TcasOR228	MTDFKDPFIMLRRIFIDVNSYKITKLCDFTVITFHSLVLCLQLYYMIRHFDVNLSIKYGPITAFFLFMTVSAVLSG<br>ALSQDIFRAVAFFEKISWSLDVIRKEARIKLERKCQVINTCISCILLFSSTTMVINLPFCENQRYFFISNQVFEEYF<br>GKWSVLLNVFYYSGVPYLGYHSVKPCFVFVYAILEIQLQFSLIEEYLLQTYETDYLESGEHLEDTQYQREIGEA<br>LRRCITHHVQLKKLIDMMVDIVLMYMPFFLVLGVFLLITCFAFIINFADTTTNTVKVQAFMFVVTALCNTVLF<br>CWNGQQLIDVTNSIFLTLGGAPWYHWNVENIKILLMFITNCTKNDSIVLAGICLDYKMFVFRISVSYALVLF<br>NLRKRSLV
TcasOR229	MTDSGQPAIADHFYRIPRISGLIVGLWPQRIRGGGGRPWHAHLLFVFAFAMVVVGAVGEVSYGCVHLDNLV<br>VALEAFCPGTTKAVCVLKLWVFFRSNRRWAELVQRLRAILWESRRQEAQRMLVGLATTANRLSLLLLSSGTA<br>TNAAFTLQPLIMGLYRWIVQLPGQTELPFNIILPSFAVQPGVFPLTYVLLTASGACTVFAFSFVDGFFICSCLYIC<br>GAFRLVQQDIRRIFADLHGDSVDVFTEEMNAEVRHRLAQVVERHNAIIDFCTDLTRQFTVIVLMHFLSAAFV<br>LCSTILDIMLNTSSLSGLTYICYIIAALTQLFLYCFGGNHVSESSAAVADVLYDMEWYKCDARTRKVILMILRRS<br>QRAKTIAVPFFTPSLPALRSILSTAGSYITLLKTFL
TcasOR23	MTDSKILLFPLKLYFLFGYHPDCSPKFRKLALLAGLWMYAFPFYLSIKGILFYRKTDFLLMLECLEAAFLFGEP<br>LFRHLAIHYHVSNLKNVLNLRQKAQIDGLEGESLFYYNLGKTFTFNYMIAGFVVLFGFCVQPFISGKLPATTY<br>LPEGYFVTFCIYYTLSGCYVVVTVVTTDALFCSLCTTAIVNFKILKRKIRNIKRTNLRHEVKKIVDQQNFLLRY<br>CEALGEMYSDIFVVYFCFSIGAICMQTYISTNDQLESAIRIKTSIYAVGLFAQSILYSISAENVLSAASEIGDAAYD<br>SPWYRFSDAQYTKSLILVISKAQRKVIFSGCGLVTINFTTLTVIIKTAVSFYAYLNSLGSG
TcasOR230	MTDSRDPFIMLRRIFIDINSYKITKLCDFFVVTFHSLVLCLQLYYTIRNFDVNFLSRYGQTTVIFLFMTVSAFLSG<br>VLEKDIYRILTFSKTFLWSLDVIGKDARMKLERKCQMINMCITCLLLFLSTALVINMPFLGNPRQFFISIHVFEE<br>YFGEWSSLLNVLYFASMPYLAYHATKPCFLFVYATLHMQLQFSLIEEYLFQVYEIDYLKSWKYLQDVRYQREI

	GNALRRCIIHHIALKKLIKIIVDIVLVYMPFILILGVLLLITCFAFIINYANTTTNTIKIQMFMFMASALLIAILFCWNGQQLIDVTSNIFFTLGGAPWYYWNRENIRILLMSIMNCTKNESIVLAGICLDYKMFVSIFRTSVSYALVLFNLRKRSLV
TcasOR231	MTEAEVKDGTKKVDDKLGCIDYRKYTFARMIMIDDGLAARGLTFPLLLIMVVNVGMQTCSFISIFTSTQTSVSLDNIRACLLGTSVTMSLFNQFISRQAIARLHGFFDKSFRTISTNPNFPEEKIILDDARKAASSQLEMYVKMFSCNAVAMMFAQPLGELMSGHSWRKLPVQWTFPPSDDEFSFWLIFAFQFTGICIAHCVGIMIMSFTSITIQMTALFDLLIFSIQHIEERATARGRQSGGDRHACLLACLTDDVDFYQQLIREMDSVTPHLRNTFLIISAAVPMVLACEAYPIMQGNFSFADLVKSFLFLSIQILCWAQTCSRLGTMTDKHAAVFAALYDSPWYEAGMKYKKLVLNSLTYAVQPKYIKARLSNEVTASMATFYSFVMSAFNLLNMIRNIG
TcasOR232	MTEEKQLRICLSSCFFLKWSFMWPTKSEEFRTSKGLYFRLLAFVIISGLTFTAMIVMHLLKSVEAGDYDISEDIAILATNTGYILMMLLYIIRQKDLESLLVDLSSFKKYQKPPKFDEVNRKLEWCTRMVFGYCVFGSVFYNLVKILAIPSCKKSRRINEVCGVAIPYWVWFDTENWSIKLPLILHTFLVIIIVDKVTLLVSLQVLEIACNIKLRLDQLNCMLVSCFDGDVEASRRRLNECIKYHKEIISYSEIFSKCFSIEMFTHLTTTGIICGCLENQVVQEHRPEAILHIGGWITAIFVSSFGGQILIDSSLSVAEAAYSSAWYEADVSLRKDLILVILRAQKALFVSTGPFNVLSFALFVSIMKMSYSILTILQ
TcasOR236	MTELRSERKNGNWDRLFGPFFESWAVFKAPQAKSRHIIAYWTRDQLKALGFYMNSEQRRLPRIVAWQYFVSIQLATALASLFYGISESIGDIVNLGRDLVFIITIIFICFRLVFFAQYAGELDVIIDALEDIYHWSIKGPATKEVQETKRLHFLLFMALIITWFSFLILFMLIKISTPFWIESQTLPFHVSWPFQLHDPSKHPIAYIIIFVSQSTTMLYFLIWLGVVENMGVSLFFELTSALRVLCIELRNLQELCLGDEDMLYRELCRMTKFHQQIILLTDRCNHIFNGAFIMQMLINFLLVSLSLFEVLAAKKNPQVAVEYMIIMLMTLGHLSFWSKFGDMFSKESEQVALAVYEAYDPNVGSKSIHRQFCFFIQRAQKPLIMKASPFPPFNLENYMFILKQCYSILTILANTLE
TcasOR238	MTFHWITTPLEPILKDDPLFVLMALPNKLIGSKLQALVNYFFFVYMVILPVSCFLVIVATNQWQIFYSPYSGYASGVFIVWSCYVSFFIFGSKYRRVYRDVFPHLWSLDVAGEEHHNRLKKIGKQLRTFKLVLITLAFIGATSGLPWFGDDYDFYIPIKLIVDYCDQWKLFFSIFFYLSFYHIGVTVLSCFFSLMFLVLHLQNQFYLLKTRLQTFATDSGTSDVFLSMKVKDEEYNRSVTQEIVFCIRHHQSVLMYCDRLNDLLYLPIFYFTLSFIVTGVSVILFPKYDLQALIRSLFVIVLGMCMTLLFCSLGQLIENESENVLYSLIEAPWYLWNTTNRRLYYLFLLKAQDTVNLSSSGLITINFQLILTLYRGIYSALTFFLNFS
TcasOR239	MTGAGAGTFRTGAGPGRGDGVARRGESGETTTLGRDAFAALGCFGAADGSTARARFFPRVTVLNPSEVPGSGLAADSNSISDSESEPELDAAQDAIDAGAGVGGDIGESRARTVFGIQGHDASDSALRMHNNVAIYAKTTMSGNSQLTFATAATIFLKNASGPNGVAIGTDYAICVVSLSLFFCYRFTELVEDTYNSYLLFQLVGSVGIICMSALRILVVDWRSVQFFSILCYLSVMISQLFVCCWCGHELSATSEELHTILYNCAWYDKDVKFKRDLIFMMARARRPILLRAGYYIGLSRQSFVSVSIPRIRFNAILVI
TcasOR24	MTIEDIGLVGINVRMWRHLAVLYPTPGSSWRKFAFVLPVTAMNLMQFVYLLRMWGDLPAFILNMFFFSAIFNALMRTWLVIIKRRQFEEFLGQLATLFHSILDSTDEWGRGILRRAEREARNLAILNLSASFLDIVGALVSPLFREERAHPFGLALPGVSMTSSPVYEVIYLAQLPTPLLLSMMYMPFVSLFAGLAIFGKAMLQILVHRLGQIGGEEQSEEERFQRLASCIAYHTQVMRYVWQLNKLVANIVAVEAIIFGSIICSLLFCLNIITSPTQVISIVMYILTMLYVLFTYYNRANEICLENNRVAEAVYNVPWYEAGTRFRKTLLIFLMQTQHPMEIRVGNVYPMTLAMFQSLLNASYSYFTMLRGVTGK
TcasOR242	MTKFFFKRLQTAPLDQEVSSLDASDYYYRIAFFLGWTPPKGALLRWIYSLWTLTTMWLGIVYLPLGLSLTYVKHFDRFTPTEFLTSLQVDINCIGNVIKSCVTYSQMWRFRRMNELISSLDKRCVTTTQRRIFHKMVARVNLIVILFLSTYLGFCFLTLFTSVFAGKAPWQLYNPLVDWRKGHWQLWIASILEYCVVSIGTMQELMSDTYAIVFISLFRCHLAILRDRIANLRQDPKLSEMEHYEQMVACIQDHRTIIQCSQIIRPILSITIFAQFMLVGIDLGLAAISILFFPNTIWTIMANVSFIVAICTESFPCCMLCEHLIEDSVHVSNALFHSNWITADRSYKSAVLYFLHRAQQPIQFTAGSIFPISVQSNIAVAKFAFTIITIVNQMNLGEKFFSDRSNGDINP

TcasOR243	MTKFNDAFPMIRAIVSLNFNANTSFKLCNIMLITIYSLIHCLLIHYMFKNFDINLVVRYTPTIMFITLVIVGAIFSVAMEKDILEAYAILPKANWALEMIKEDAQLKLERKCRIMNICILCVLLLILSTITINAPFFGSQRELFICIQVFEEYFSKWSFILYHFYFIAFPFLYYGLLRLWMGFVYAVLEVQLQLTLVEEYLFETYQINSLKEWKNLQDTHYQQQIRKSLRLCITHHIALKKFVKMIVDLTIKVMPFYLTIGVLILISFFSFIINFADSMSNILKIRIFMFSASIVCITVLLSWIGQQLVDVTSGIFWPLVGAPWYFWNLENVKTLLIFLMNCTKNESIVLAGICIDYSLGISVLRLSVSYALGLYNLRKSLD
TcasOR244	MTKSGDPFIILRWILLMDVSNNKITKYCNIFLTIIYSLVLCLQIYYMFKNDDINLLIKYGPITILLLFMITVAVISLIMQKEFKTVTFIRETCWPLNMIKNNGQIKAERKFRTISFYTLCTVLLYLSVIIINYPCFGSQRDFIVCIEMFEEYFGEWSSVLYYLYLIGAHFLYYRLFQTCYMFVYGMLEAHLQFFLIGEYLLGTYQTDCLKRCKYLQDIRYQQEIGKSLRFCIKHHIALKKLVKMVVDLAVIGMPFFLVLGVLLLISCFTFIINFADTMSNILKIRIFMFVSSSVCNSILLCWIGQQLIDVTSDIFFTLGGAPWYNWNLDNIKLLLIFIMNCTKNESIVLAGIRADFQLFVSLLRVSASYALVLLKLRKCSFV
TcasOR245	MTLIESCWMVFTWLGLFRPIKWKGLKARIYDLYTAIVLFFNYSFFICGVMDIDFTHLNFFADIDLITLMLQYIENTPKILCMILNRNALIEIDFKLQHDHFKIKDEDEKKIQNKFDKFSRYVLLAYSALQATSLVYYTTGRILAMESPVILPYRSRIPFNYSSSGKIYMLTALDQLYSVSSLICINGAFNLVFTSTMYQICTKIRILKHRFKVIIQQLEHDGELGNNDNKNLRDVMRKNDATTDKFESQLIANWVESHIALINLYDYAKSVFAKAVFIHYVINSIVMCTLAYILSHCEIDNIFFGNVCYFSVKCTQQFLQCSSAHQITLEFEDLRDMIFSTNWFATKITIQKSIIIIMFKSIVPIEFVSGYFVTLSLDSFKRILKLSYTIYNVLEG
TcasOR247	MTLKSYIKKTLKWEEPLGLTTMIAVISGAWNTMAPPQSIQRFIYWQSWFQTSTYVLFMMSAGINIFVSTDFFGECLESLHFLVTAFHVFIKYMTMRFRERDFLELFDDIKRVWSGYRIHNEKFLSSTLASVNRTTVIISVCIINVMFVNIGAAVLKNILEPDKIHFPIQIWIPSFCRDSFMYGTIAQVVLFSWPLFIVAQSTTFLNSISVHVEALGLSLAKDIGRQKVWKGDSARRFYKKHQEVISIVSRVNALMAGNWGFEMLCSSLQLTLPAYRTLRALKRNEIEVFNHAVILSLNFMVIYIIFGSGNRILSMGEEINDRLYESDWYKLPVKEKKNVLFMLFRATKPVEYRYKMIHFDLPGFMKVVNTVFSYMALLRFLDGGNDGGNGGLL
TcasOR25	MTLLIEKKNYQRKFYQILMTVAFFLDTNKYRYISSFIMQFYIFDWMVLVFLASAYNIWDEKSEMLMVMELIQYMIVGVYFSLIFVVFITKKKDIVSNYDCIQTDFIQWSNKRALNPNVAYKKNIKTIKRLVIPLATLSLSIAFGPLVSAINDIGKLPLDNRAHFVLFWPKIVDTNKVSMYGIIYSLQVIFTVILYISVLSFNLGFMVFLNELTNQFEMLLDGINDAFKYKMDKQFQTLFIDCIRHHQIIIKFLDDLKSYFKWMILIEMIVVQVVLAILIYNLTKVHASLGYKVKIAGSLLFNLLPICFHCHIGEVVLGLHKRLSNHIYNMTWYDMPNKNKQLIVIMFQRTQRDLTLSSALFSSERASRSLISKVIKQVYTILNVLLKT
TcasOR259	MTLLSFFKDKFKWNEPLGITETTATLFGAFVNYAPSPGYRKFFRFFGWYIIFMFIIFNINVVLTIYFASDFFEESLEAVRLFVTAIHILAKLLTMRAMEKQYMELIEQIRRAWRTYEYSSGDMLNKTLAAANKGTVIVFVAIGNTIPINVIVAALKNLGNPPEIQFSMQCWVPPSLRTSFLAGSFYQLTPYFFPVMLYCMTISFLNSITLHVEALGLALAKEIRSQKEWRDEAARSLYIKHQEVVRIVGRVNDLMASNWGFEMMCATLQLTLVSYNALRTLKKNDVAFFNQANLMLVNFLVIYFIYGNGNRIIKMGEELHNSLYDTKWYTSTVKERKNVLFMMFRTSMPMEYRFKIAHFDLPSFAKLVNTVFSYITLLRSVDEPEEQGAF
TcasOR26	MTLMRKLQTAIRNLFEIQIKDDILAELLDWPTLVLFSKWPKNFAIFSTIYCVFDTLVCTLVYSTLDVEMLGKYAIFIAKSTIALCSFFSFFAKRKQYHKIINENFPHFWQLQSMGESTFDQMKKIATTVKFYSCLSVVAMLIGAVILILFTEDESEIYLSVKIYKDYVNKWTTGYIMFFYASFLYIGIVTAAVVFGLTYIVFHLIFQCFLLNQKLKLINSYIVKNGQKLVKLEERNQNFIYKELISCVKLHQRLIYFSNQINDLLYAPIFMYTFSGIVVGVALIYFLKTSIQYILTSLVLSIVSLIITTTFVINGQLLEDETENIIISLTNLPWYSLNVQNRRVVYVMLMQSQKIIHMSASGIVSLNYQLTIVLFRCIYTAMTFLVNMGL
TcasOR263	MTLVRKLQAAATNAFEIRIKDDILAELFNWPFLVLDSKWSTKFAVFLTVYCVFETLACALVYSTLDVNMMGTYAIVIARFATTFCSFFSFFTKRKQYFEIINENFPHFWPLQSLGKSTFNRIKMRASSVKFYSFLNVVVMLIGAVILIS

	FTQDESEVYLSVKIYKDYVNKWTTGFVMFFYVSFIYIGLVVAAISFVLTYTAFHLIFQCFLLNQKLKQINDSIVE<br>NEQKQAKFDEKYQSFIYKELISCVKLHQRLIFFGKRINHLVYAPLLVYIFGGIVVGVALIYYLKSSVQHIFTSLIL<br>LLIALINSTTFVINGQMLENEAENIYISLTNLPWYSLNVQNRRVVYVMLMQSQKIIHMSASGLVSLNYQLTIV<br>FFRCIYTGMTFLVNVGL
TcasOR264	MTMQFIVKRATRGIFHDLRVLKFISSDIFDIKIMKLCLFITFLIHLTACAITIHAFMFNNFSRREFISCAPVLFGCF<br>YGLLGLGTILFKPSMTRTLMLELKAWDITAADDAVSSRIKFEINVITVFCLVNYLLALVASFFYYMSFYGDEEIF<br>YLIRFLEDHCPNHKRVLIKLYKISFVLLGYVMVVHACQVLYATQHVRFQLILCAHFMANVTKQAKNIKDEH<br>LPDDNNYQNMIRERLKFCIIRHQEIRRFYFDKLEEMGNLIGGFALLGCFLGISFAMHMLTSEFLRYHFARTVS<br>SIIAGVTTFATVIAAGQSVETEVDISTRVVKEVKWYTFNESNKRSYMLMLLNSMQTYKIKFSENYSINYELGLS<br>IVRGVFSIVSVVVQLDY
TcasOR269	MTNFFSNFCSPLKNHWAKTKHLFSKFSLSSDQPFIMIKLVCVDIGYHPVAKTINYICLAIHISSFLLEMNYLRL<br>NFSTDLLIKYGCGISAVVYDISTLIVAPMIERPTIGLSEGITTSFWPIDFCGPKVKQLILEDTKKTSKIYYRTLVTIF<br>GFAAVIMLPIWGDQKEWFLCVQVYEHYFGKWAQIPYHIYFLSFMWFAFTSVRLPLMMSYAIKNIRVQVFLV<br>NQKIAKMSKEYEEAKIEDVNYQNRVYKNLRLCISHHVLLKWWLRKLQKIVRFCLPVFVVIGILTESSVVFYLI<br>YNFKKVNLLLKIRFLLLACTTGVIIYFFSEAGQSLYIEVFDSLISCPWYSWNVKNRKVLLIFLTNSLQPMFFSLV<br>GFTIDYRFALTMIRTSFSYAIILYNLSSGSQIASI
TcasOR272	MTNIFSNFSLYFKNTWTKTKQRFSKTLPSSNVPFMMIKLVFVDIGYHPVSKIINYICLAIYMSSFLLEMNFLRLR<br>FSTHLLIKYGCGSSLSVYFISSMTVAAMTELLAVDLSEGILSSFWPIDFCGPQVKQLILKQSRADKRMHYVVLL<br>VFSITGLAMLPIWGDQKEWFLCVQVYEYNFGEWSKIPYYIYFFTFPWVAFSSLRLPFMMNYAILNLRMQVFLI<br>NQKIAKMSNAYDQTTIEDVNSQKRIFKNLRLCISHHILIKWWLRKFVNHVKFCIPIFVIVGIATSISIVFYLIYSF<br>QQVNLVLKIRFLSIACCCWFVIYLFSEAGQSLYEYTEIFHSLISCRWYIWNVKNRRILLVFLANSLEPMTFSLAG<br>ITLNYRFALNMMKTSCSYALILYKLNCDSQIMD
TcasOR276	MTNKLSYAPFTLLRKLIFIESKHCKLARFCDFLLIVLYSLAQCLNMYYMYQHFNLSLVIRYGPVLVFSLLVIVTS<br>VISVAWEKEIFELHMVNRKIFWPLNCVGKNAQTKLTRKCQFINHWISCSLLLFLITVIINFPCFGSQRDFFICV<br>EVFEKYFGEWSFIPYYLYFAASPFLYYHFFSSSFLFVYTILDAQVQYFLIGAYLFETFQTDDLKGWKYLQDAHY<br>QQGIGKSLRLCIENHIALKKFMKMSLDFVLIGIPFFLVFGVLLLISSFAFITNFADTMSNILKIRMLIFATSSVCIT<br>MVLCWTGQQLINLTSEIFLSLGGAPWYFWNRDNRKILLMFLTNCTKNESVVLAGICINYAFFLSLVRLTVTYT<br>LVLYKLHRSGIV
TcasOR277	MTNLEIKICRATLKILKYSLIWPNEADEMNPGKWYYIRVATFLLITSLWVLSVFMHIVMSIIHDADVHLSEDV<br>AFCVAFCGLYYMTMIYVKNQPKVALLLRDLSKFQFGKPPGFEEKERILGFLSQFFFYYCVMAVMVYNLVKLL<br>QKPDCEKMNEIKGLKENCGLLTPTWLPFDINYFPAFHLTFLYVFISTQILMKLALIISFNALEMAYHVILRIDH<br>LKIMITECLDQRNYEVSRRKLKTCILYHLEILSLSNRLNDCFSNIMFAHLTITAAICGCLEKQFVDGDNRLGAL<br>LHVCGWISALFVACIGGQHLLNASLSIPDAIWSSKWYEADVRIRKDLLFMMAKSQVGLHLNVGSFGVLSFS<br>VFFSVLKMSYSILAMLTS
TcasOR278	MTPEKFMGTFSVQHAILFSALFIFHTMYAIYEVTITAFFARSLLETVSHFYIATYLFAFNFQWYFMLYSIRTFHE<br>NEIFLEDFKCTQTHADFATRELDENVYIFTRVLFVSCLCWTVNSSTHIIGPAIEALISLIKTGQIDNVLFILPPVFS<br>TPWWAQIIIYFCNAITMFGLLLYCLASYTIMGFKVLKLKTLCDILNEALRNDVEEESNIKAYIKDHQIIIKAAK<br>LLNAQLRTLNGFMFTACYLEFAVQLFALTLIDPSGSYYFALALDLSSIFLILVFQCWMGTMITHSLETVANGVY<br>ESLWYSRGNNNRSEVILMTQMAQKPFVQTIWLGTLKVERATSLSLVRSSYAMYTLLNFFQDK
TcasOR281	MTPKVGKSEYVIKDTKVFATCRIPLVSMGFVDDGTVMSKIRKLITVFLMYSAPVHHILPAFVDETINLDGVLI<br>GVSLLMLYILICVSWPVMFRRSNDIIKLWKTVQKGFYQYSDPLTKEERTLLSETDDLVIKTTRISIIAYFCAGFGT<br>FLKEITPSSLRAYKTPYPGWFPWTINSNFRFSMALLYQLALCLNTTFALEAIFVLFAYHVIHFECQLRLLSQHF<br>KDTFPTGLSATVTYGAGYRKNTLRRLNECVRHHLVIKRFHEQILSYFGICLLVYRVIVTIMLCVLCYLVTTGIS<br>ANKFLQLLGLALALLFLCFIFCLKGEKVTLMSDQWRQTIYEVDWWNHPVGVQRTVLMMQLGATKPLRIYG

	VWKPAMYSHEGISVIGQETFSFFNMLRAMK
TcasOR283	MTQTDVLHLIKFLTNDIFRSKIAKIFLLSSTIFSASVTLIHSYFMLFRPNLREFSLKAPMFFGFCYPFLAGVVLLF ENKLIENIPKQVKSGPIDQKLRKKIKTIKLYVIFVMISAVLAGLSYVQNVTNEAEIAFALQFFLDFVPNYYTFLA VCYKVSLFLMAVIAIIHPMQGIYAIEHMKIQVILLQKHVKMIEKKAKSGKDVETTLKFCIKRHINFLNFAKKL SARLSFLIAILVACGCVILMAISVFMLSGSFSPDYTFRITATSLETLATFVGVLKSGQELEDEIDKLGQILCTLDW YSFNCKNRKLYLIFLMNAMKPFKLKSLESYAINYQIGLSAVFTVISFTSQMHSKLYKNGH
TcasOR284	MTRITDVFSLNFIFWKFLGLWGKSAPSKYNMAYTVFYLFASLFVYDIFLTLNLIHTPRKLETLVRETMFYFNHL VAVTKILMMFIMRKKILVIFDLLDCEEFKPNDENSQEIMKRKTDFYYIYWRIVAVTSNLSCFMLVIGPLIKMLI WKIELGLPVCKFYFMSDELRNKYFVIWYIYQSFGIYNQMVNNLNLDTFNCGMLWMAVGQLQILKTKFVNL KLNDFENGLDLKSRDDMQIERLRKYLTHYEIILKYCATVQDILNITIFVQLGMSSIVICVGLCGFVAMPSNTET AIFMSSYLITMTMQIFVPSWMGTQISFECGELMSAAYCCEWIPRSKLFKRSLILFVERAKTPVRITGLKIFTLSL DTFTSIMKTTYSFFTLIRQLQVDEVN
TcasOR285	MTRSLKLQDQETFDQVAKVLKWNKWLLSTLGLWPQSPNTFIFTVNFSYFVYHMAMEYLDLFLFIDNLEHVI ENLTENMAFTQILVRIAMLKKYNRQLGEVVNEAFKDYDARIYRTDEERQVFIDYMKKAKLFIKLLCAFVTM TATSYYAKPITSPPPPPEGELDVEMENATMSFILPYRFHLFYQVNDSRTWALTYLSHFPFVFVSGFGQTAADCL MVTLVFHVSGKLASLAIRISEINTEPGVCKQELRSIIIEHDRLLKMGQSIEEAFSETLLAHLIGATSLVCILGYQL LVNYARGQGADLVTFFVFIFLVFLVLYAHCVVGESLITESFKVCEAYYDCLWYKMPKESSKTIVLCMARSQKP LGLTAGKFGAFCLSTLTDVVKTAMAYLSVLRTFLVIE
TcasOR286	MTRTPTDDTTFDLDNFMKNDSMKLVRVIAYDTLKFKITKLILFITFLVHFSTTLIQVYFVCVDFNVYFFVKYAP AMFGSLFVMVSIIALFVTAETDMVVRVFRKAQLRKLTVEDGPSFHVFQKECKIFTVFFVLNLIIALFSGYLHAL PDDDDREIFYAFAFFEDYCSEWKDFCSFLYRITFLPVAYVMYVPINVFVYAAIHLKSQIYYLKEHLIQINEGYDI SNNNDLFYDENYQRIIREKMIYLYKIHVKLFLAALDIRKLIRGFIALFAIVGCLLGISILYFVMLFQGNLFDKFG RLSTLTVVAFNSFAAVIISGQMIESSSSDDVDAIIYNCNWYDWNEENKRFFLLIRMATMHPFKLQFSQNYAVN YQLGVAILKAMYSAFSLLKAIKNDF
TcasOR287	MTTSMQPSKYTGLVADLMPNIRAMKYSGLFMHNFTGGSAFMKKVYSSVHLVFLLMQFTFILVNMALNAEE VNELSGNTITTLFFTHCITKFIYLAVNQKNFYRTLNIWNQVNTHPLFAESDARYHSIALAKMRKLFFLVMLTT VASATAWTTITFFGDSVKMVVDHETNSSIPVEIPRLPIKSFYPWNASHGMFYMISFAFQIYYVLFSMIHSNLCD VMFCSWLIFACEQLQHLKGIMKPLMELSASLDTYRPNSAALFRSLSANSKSELIHNEEKDPGTDMDMSGIYS SKADWGAQFRAPSTLQSFGGNGGGGNGLVNGANPNGLTKKQEMMVRSAIKYWVERHKHVVRLVAAIGD TYGAALLLHMLTSTIKLTLLAYQATKINGVNVYAFTVVGYLGYALAQVFHFCIFGNRLIEESSSVMEAAYSCH WYDGSEEAKTFVQIVCQQCQKAMSISGAKFFTVSLDLFASVLGAVVTYFMVLVQLK
TcasOR288	MTTTGATTFAPASEDLTIVDNKLFKAICLHQILDPTKGRNRYYRSALLAVMWLSLSMQITQLVGLYFAVNDL QRFAFTTTTVTNAFQCLSKGYIIMTHADRLRASLETARYDFTSCGARDQRIVRRSRNVLSTVLRTFIVLSWVT CFIWALTPLFGMDEYLQVTNADGTFTRYRVTIYNVWLPVPATVYNATAVWALVYSAEVIVCFVNVFSWLLF DSYVVTMCFTFNAQFRTVSASCATIGHGDCSGSPSPHATGTHNIIRDDNNILNCYDELINHIKDNQSIIKKCD DFFEIIKPAILFQIIGGSYSVITLIFLTLLTYLMGFSIISIPVLKVFFGFLSVTFELFMYCYVFNHIETEKCKMNFGM YSCNWTAMDLKFKKTLLFAMNNNSAHRRVMKVTPKSIINLEMFSNVMNMSYSIVSVLLNSRVQK
TcasOR290	MTVVQTNKRTAMTDVHHYHSLLLTMLEIAAVFKKREGSIFSPTGFKMFRVANLIVCFLFVTSCARYVFHEKG AQFFTVAIGTGSIEFCIINMILVSKSDIIDRMLATSAKIFYQLPQNEETRDVLETYRTKGYTFMRAFGMLIGVNE VLGLIKPFWMARLTGQLGLPFDISCLGIPTVPCWIFQIICTSHLIVTVAFHVIIVKTLMYLTWGHSIVITKIMNR RPVHDDEENDRKIIELYCDFSRFSTSFSSLFGLTTFIEVTFTSTRCCFLIYHAIKSLSNNDMEQAIVSVTALIASIAI SYVMCSCGEDLVEINQMMRDGFYNSKWYESSPQSRRRMLPMLVLSRVPIRFQYRYYMYFNYEILMKIMHST YSLSAALIQFL

TcasOR292	MVAIGSRAKPILVQQSKTSSKGQDGGEESVVSPSENVLRRNIRILRLAAVWRPPGRWRQRLYPLYFGTVCTSM<br>LHIGALAILRSYTIWGNMTEVTFALVSGLTCFNGAVKMIHHYTHSETYYRLVDELNLLIDRQRPYCEGDAELT<br>EALQTAYKKAKRLTWGVLLYMFVLGQMWCIVPLFMKFPPDDPSSPLPLVTITRVHKVHNHTLYSMAYLSEC<br>HTVLYWNWSSLGMDVFFGSIMIHVTGQLNILNIRLSRLSHEGVGDGLAQYSSFVKGSELHKGGIHDSASMY<br>DELCKCVKDHQEILRYLDFLESLMNPVPLAQFLLCVGGICLTLYQITFNPDDGGVIECILFLPIPALQIFIYCWA<br>GHGIMEESEYVSFAAYSCRWGGAERKVTNVLRIIMSRAQRASLLTAGKVHPINRDTFLSLLNASYSFYTLLRQ<br>MKNLEEENEASS
TcasOR293	MVASTSKNRKIKNEQAVRGFTRSEEKQIEDHLFSIFNILPIIGGIFGYHQSPKWSALTYTLNIGMYTSVSLTALN<br>LLYCSYLLRDNLSQVTMAFHCFLISCVVMTASISLTLQRNKLIEFLLKLQFRGPLAEYHDSDYFQALEGKTRQ<br>RIFRMLVIFLSCYGACGLIAVIFPFVDLYLKNADQVTNVPEIYWKGLPFAVWWPYDVHNSTSAWILCFLSQGI<br>WALFAPVIVTTAVVLCFYGAELILNHFKLLIFSVKNLDQRTKAMYERKYKENSRTQLENVYEDCFYECIVQN<br>VKHHHIILKIVEEFLALANYAIAVPFFGGALLLGLAGMNLLSTDDLRIGPKIFCASVGATEATNMFLLCVYGE<br>KFQHEGEELFNSIICTRWYKRSMKCRKALMIMQCGSFRPPKITAARMIELNMATFSNLVNSAYSIFNLNSVAS<br>ATEDK
TcasOR294	MVDRSVETPLTWRESAESVLRPNIRLLCSLGLWQPVDSMLFHAFTAAVLAVGVAHLAVAALGIWQRPADLA<br>EVAIGLSNAFVIFTALSKAVLFLTRRPLFYSLARLVDQMTAEQKAFRAGDPSLQEVFSAARRSAGRLSVFFHW<br>YVLVADVLWSLIPLVQASREKRWPFQQLPLDGWATSPAYQLSYGLQCASTLFFSLISVDVDCFFVAVMTHITA<br>QLKILTFRFAAIGNRMYISDTSLNNQTASTKDASHEKLRGCVQTHQNILRLVSFLNVVMSPVAMMQLAVGV<br>VSSCMVLFPAANSTDSAVVMKCWAALPVLGVQLFLYCSGAQRLIDQAEAVSGAVYSCAWPEAGGRVQRSL<br>LVVVSRAQRPPELTAGRMFPINRPTFLSLVNATYSYYTVLKQVNSH
TcasOR295	MVEDLTVKDLTGIYLLPHSVAYMHFTGHWIGAVPGPTPFRVKMYRAFGGTFTWLIVIATAIASLNGMLHGS<br>GMSDISMNLIIISTSISSLHKYSVFIHQEQGLGRLGRWMKRANEQNKISKNPDTTTDRILKKSLVSFYYSGIVAA<br>SLLLVKIVVTGYTYNALIPGLDQRYQPLILIFMEAFSFSSLSLEVIMDALILMNSLFVFRRELMKNVDEWRKM<br>NYKSDNPQQFRQQLKTNVQNHVELLTIFQDVKNYCNSMFGYQVFAIVF
TcasOR297	MVEFKDPFIVLRKIFFIKFNNCKLTLFLNISIIVFFSLVLCLQICYLMKNFNLNLLFRYGPVTVLFTLVTVTAVLSL<br>TLEREIFMAITFFFKFCWSLNIIRNDAQITLKRKCRCVNIGLLCILLIILIAIVIGFPCFGSQKDFFICLEVFEEYFG<br>EWSFIPYYFYFAASPFLCYHFLRICFTFVYAILEAQLQYLIIAEYLFEIYQTNPSKRWKYLQDTRYQQQIGKSLRL<br>SIVHHVVLKKFLKRTLHLTKIGMPFFLVLGILLLTSSFAFIMNLGDTMSNILKIRIFLFTTSVLCITILLCWIGQQL<br>IDVTSQIFVSLSGAPWYFWNLENIKILLMFLTNCTKNESIILAGICLDYKLFVSVARLTVSYAVVLFKLHKSSLV
TcasOR30	MVEFKDPVIMLKTIFLVNVKEMTKFSQVFLAIFTFYSLVHCVQMYYLYKNFDVNLLIKYAPATTATLFVSNTK<br>LLSSSLNIMPIFSVVSETKLLRITTFIDKTFWPLDSIRKEARIKLERKCRAINISIYCILLLLSVAVFSNFPCFGRQD<br>DFFLCIKIFKEYFGQWSSIPNYIYFTLFPIFCYPYFRIAFSFVYAILETQLQFSLIEEYLFEVYQMVDLNWKYLQDP<br>RYQQEIGKSLQLCIEHHTALKKLIHSIVNITLTGMPIFLLFGIGLFVSCFVFIINFGDTMTMILKLKTPLLLYVAT<br>MLSMTLLMCWNGQQVIDVTSRIFYTLVRAPFYFWNLYNMKVLLMFITNCTRNENIVLAGICLDYTLSVSILRI<br>SVFYTLGLLELRNHSFD
TcasOR300	MVEFNDPFIVWRLIFMINFKKYKIPKFCEIALIVIYSLIHCLLLYYIFTNFNVNLVIRYGPIMIFYIFMIAATIFSVA<br>LEEELSGGIAYLDEISWPLSTIGKEAQLKLKRKCRIINMCIAFVLLIILSEIIVNYPSFGDQKDFFICVKVFDDYFG<br>EWSSIPYYFYFTASPFFYYYYFKLCFTYVYAVLETQLQFFLIEGYLLQTYKIDNLKRWEYLKDTRYQQELGKSL<br>QFAIAHHVALKKMVNVIVSLSVNGMPLFLLLGFLLYISCFTFVINLADTMTNILKIRIFLLGASCVCVTVLLCW<br>NGQQIIDVTNSIFSTLTGAPWYFWDLDNVKILLIFITNCTKNDSITMAGICLDYKLFASLLRISFSYALVLFNLR<br>KSSLS
TcasOR301	MVEKSNFHVKRAQLFKAYNSIHWLTLTKWFYEDYPVEKLWSDKRLWIHLSIVIICQSSITMFKVFHLISEENFF<br>IFLTSLTSFLVIVLVAVRTYILYQFPTFRQLYFKPEVFNCNLHRPTRSLALLTEAITHSRKVGMWCLVLFITFDVA<br>FLVLPIVPPILEIIDGTNKTYDELIPQYPSINPVSLSWLSKELKYAFDLVMAVFNTIPWVGFVVVYYTVVQLFRA

	QHKIMMTAMLPGPPVPGDGREPLELKLWIQDHALIRKLVYKLRNTISPALAGTICVNVFTVGLNMLALVSSPIGSDAPMFTRYLYYFSFGTYSALSIFDIFIHCWLASEITNCGEDLSYALLKSDWQNDLKRSHHHYVLPLMLCKKQIRFTGLGLIPVTLTTFTETIRVSYSYFTLLRKTDD
TcasOR304	MVFELIRPAPLTEQKRSRDGCIYLYRAMKFIGWLPPKQGVLRYVYLTWTLMTFVWCTTYLPLGFLGSYMTQIKSFSPGEFLTSLQVCINAYGSSVKVAITYSMLWRLIKAKNILDQLDLRCTAMEEREKIHLVVARSNHAFLIFTFVYCGYAGSTYLSSVLSGRPPWQLYNPFIDWHDGTLKLWVASTLEYMVMSGAVLQDQLSDSYPLIYTLILRAHLDMLRERIRRLRSDENLSEAESYEELVKCVMDHKLILRYCAIIKPVIQGTIFTQFLLIGLVLGFTLINVFFFSDIWTGIASFMFVITILLQTFPFCYTCNLIMEDCESLTHAIFQSNWVDASRRYKTTLLYFLQNVQQPIVFIAGGIFQISMSSNISVAKFAFSVITITKQMNIADKFKTD
TcasOR305	MVFNSKKNIISLFSLLEDSRHPSVGPHLRLLSLTGIWYPNSKTNITLLKRACFYVIVLFFVSQYLKCIIKFKIDSLQLILEYAPFHMGIVKTCFFQKDYNVWQDLVSFISKTERDQIAKKDPKSIKTIQSYISRNRKITYSFWALAFIANIGVFSKPYQNNQSDVNGTVTYNHLFDGYTPFSEEPPGYYFSMGIETILGHVVSFYVLGWDTLVVSIMIFFAGQMQMSRLQCSRMINGSPERTHKNIIKCHKFHTDLIKYQKQFNSLISPVMFVYLFVSSINLSVCIVQIAEIEDDFATVLSSFIFLLACLIQLLLFYWHSNEVTVQSELVSYSTFESNWTSTQNKLQKEVALLGLTTSKTLVFTAGSFNHMTLATFISIIRASYSFYALLNSTKY
TcasOR309	MVHFPSQKRPLKDDPFQYLRRCLEPWGQKPSLLPLCLLILLIKIFFFTARTVFILKWMKEIDVLEISVTWPVPLLVVIKMCYTLYQRDKIGFLFRSVTEKFWDLGVGGPGLEKELEKRFKLINRFLFGHVSLGVFYVGLYAFFADVPIPKGRTRWLPVLASMPFDQDQSPQYEILYVLMYWNLVVSILGHGVFDMVFIYSSQHLVGQFILLKALLRKLDYGFEGLEIVAKARSRQFQKEIRKRIAICVQHHNLLLAYGNELKKIASMIFGVHVLSTSLTLILVGYILSKNLEKILQYSMLLSGVVSEALQFIIFAVQSGEIYHKSVSVAQAAYQSNWYVFNAKAKRDLTLLILNSQKGISMYGAGLVTINNEILVSMIQKIFSSITLLRSLGEQK
TcasOR310	MVIGTIIVSQNEDNLMINMRLMKKTGFYQLLDSRSLKVFGHNVFKCMSVVQISILLSIAVIFVLNIYYFSDDINTVMMYSMLITSDVLSILKLYYILQNSDTIWNCIQLTSIDDLSYKYHDRRILEEGRLKSKSYSLLIIFMWMYLIISWGMAPLFVTNYFLTVEVNNQIHRYRFNVLNYVFPATDQFYNDNFVFYYCVELLTLVLWGHCTMNFDILLLSMIITFKYQLKTVANSFKSISDYKSLIYDHQRVIENMRNIYRVFRPVVLTQLASESVIIMLLSCITMMNYFNGMSLLSAMNLRLFAAISTFTFHIYVICYLFDDVNEQKDSINLALYSSDWTPSNLQHKILLLHAMRMNNAENLRIQVTKSRIVNFKMFTDIMRTTYSILSVLEKMCANKT
TcasOR311	MVIIDSLSFYRPFWICMRLLVPTFFKDSSRPVQLYVVLLHILVTLWFPLHLLLHLLLLPSTAEFFKNLTMSLTCVACSLKHVAHLYHLPQIVEIESLIEQLDTFIASEQEHRYYRDHVHCHARRFTRCLYISFGMIYALFLFGVFVQVISGNWELLYPAYFPFDLESNRFLGAVALGYQVFSMLVEGFQGLGNDTYTPLTLCLLAGHVHLWSIRMGQLGYFDDETVVNHQRLLDYIEQHKLLVRFHNLVSRTISEVQLVQLGGCGATLCIIVSYMLFFVGDTISLVYYLVFFGVVCVQLFPSCYFASEVAEELERLPYAIFSSRWYDQSRDHRFDLLIFTQLTLGNRGWIIKAGGLIELNLNAFFATLKMAYSLFAVVVRAKGI
TcasOR313	MVKLFDELAEQEDEELMGVYEKLYGPALQLSLLFPSWKRENLYKTFGIILVYTVTLLVHFYVLSVSVVMLRDDFEAACLAFHYWLIFVMVFLSLALINMDRRTFSFAHRCLARDMGNYAAGRIYSESKPLALEARKKKELFRFLVLPGMVVILAAALLVVPYLKKINNPPHYNAYGVNMNLPLATHYPFPTDHGILHGVVVLGQLSAAFSLAVIVVSLELLLFRVSQAIIFEFKILQYALETLFERSERLFFQLHPDYYGKLSHMNSNYQKCITRCIQDCVKHHYKIQELLQAYEYVLKWPAALGYGIGTGVIGLGLVTLLMAKEKGNLENVVIFSLLIVAEVLNMYIVSVFGEDITTESAAVRDELYFIEWYKLNIPNRRMMLNFQVGITNPVIVKAGGLVALCMDTFSSIMNTSYSFFNLMNANPLDGNK
TcasOR314	MVLEKEPKLMANSKLLKTIGLYQILNSSSPHPKVYGYNSFKCIAVIEIFIATATIISCILNAFYCLNDINEATRYFSIGVMCAIIAFKFCYIIGYSDTIWNCLRITSIEYLTYKYHSRRMLEVGRKRSKFFLILFIVLWIAVYLNWMLIPIVVQNSYVRVEAEHLIYHYRTNIMSLVYPVTDKFYNENFIMFYSIECIVMIVGIHSTFMFDTLLISTCITITCYLKTIANSFSTLGNVENHFMTRYDETKILNDFKIIIQDQQKVIENMKNIYKVIRPVILFQIAAGSSVIILLSIITIMNYFNGFSLASPMNLNLLSTILTFTLEIYFICYLLNGVNEQKDSLNFALYSSDWLDKSLKYKKKILCAMRMNNANQLK

	LQVTLTRIVNLELFTGVMRTTYSVISVLSDQLAKQT
TcasOR315	MVLFLCTMLSEIFLYSYYGTILYEENQTLTNAVYMGKWYNYDTKSRKALVILMERSKRPMLVTAGKILDLSLE TFTTVLRRAYSLLAEHQEMKGLVEKSFRVNLLVMQVMGFYPPQKYKSLYKIYTYVVYCAFTTLIPVLATLELF LAENINLEQISDNAFIVCEAGCFIIKYLPFVRNADKIKKSLFLIERPMFHIYTKRQEHIIEECVAICRRNCRLFLT FCTITVINWSITPFFLPGNNLPVEIWSPFEHKASRKFYFLSFVYIVAGVGNAAVSSGVIDPLLAGLISHATSQLK VLKNNLQFLDEHAEERIASRNISFIERKRFKADFIYQQIKLCVNHHIAITEFIDVYEDTYSSSVFIQFAASVVVIC ISCLRLSMVEPFTFTFFVMALFLWTMLCEIFLYCYYGTILYEENHSLTNAIYMGKWYNYDIKSMKALVILMERS KRPMIVTAGKILDLSLETFTTVKRSFDC
TcasOR316	MVLVPYLKPQKERNAAVDRGYDITGMFYARLAGLYPDLEIGWRYWFFGSYQILVVVAYFYYVLAYVIANVIA IKYMDVELIGSTLCFGSYTYTYALIALTFYIKRSKIDKLLEIIGNELYIYQCPLSQKQLKIRNEETTRAKNFGRYSF FVPCLVALTHMSVVPAIHGFKGEYSSIVNGSAPINKYTPLPVWTPVQATSGMSFFVVFWCQLCPGFVEFLIFH GSCTFFVGVVCVLVSEIKILLESLNSITDRAKYLYHVKGGRGSDIDNLYDDPIYQQCMVDCLKENVKHHIKIK EFRNLFQDIISYCIFFIFGGAAVTISTPPYTILKIMESGDTDKLYSAGVVMMGHTFLSVYLLSRYCKFGQNFESE NSKLLEAFYCTPWYNTNMDYRKILIIAMSNSQKTLQIKGSVVGVSLSAAAFLDVIKSSYSLLNFLATAGS
TcasOR318	MVNVQKAGGGGRRLCTDLRLQQWLLFLVGAWAPHRGAPAICSLLYGVYSACVVLVLLLFVASLLFAMVHY WGHMLGVTMNACLMFVYLMNIVKIVSLLKMQPSAEEFIRELDRCMQEYGGQSLEMEKAAVFQWTALKSRIV SVARMLVALSGCLYWAVVPAARAHACGGTVQCRDQVGLPAHVWYPFHFTHTPVYEVVYTVVAGGLLSGA LISCIVDAFFVSLIIYQAAHLQLLNLMLAAVGTEKCYQPGPPTSPGSKRRLAEESAGAEQRMHRQLAECVSY HCHIDCCVQHLSSLVGPILLGQFLMDMVTISATAFVAIANNADSAWLLKYTSYLSSVVQQLLLYCWFGTDIIT QSERLQLSAYSSQWVSASPRFGRELLVFLCRAHRPLRLTASKFYTISRETFLLLMNASVSYFAVLREISSK
TcasOR319	MVRPCRYFAIHFILLRFLGLGWWHHPHENETRNYPGLYLYYSILTQLVWVVGLVGLETIDPFVGEKDMDRF MFSLSFVITHDLTLIKLYIFYFRNVEIQDIVRTIEIDLYRYYQNDDKIRATIRISRIFTAAFLFFGWVTIGNANIYGI VQDLRWKDIVKNLNETTSKPLRTLPQPIFIPWPYQEDKHYILTFILETMGLLWTGHIVMTIDTFIASVILHMST QFAILREAIVTAYDRTMIALSEGALQSGVLCENSNGNEENNQIFLESFYSKEHIESVLESTLLSCIRQHQLLIGC VEKFSKTYSYGFMTQLLSSMAGICVVMVQVSQGASSFKSVRLVTSLAFFFAMVIQLAIQCFTGNELTIQAERIA DAVMESKWEKMPVRLRRLLLVTMMRAQRPLHLTAAGFAYIDNTCFLSILKAAYSYYAVLSQKQG
TcasOR320	MVRYVPRFADGQKVKLAWPLAVFRLNHIFWPLDPSTGKWGRYLDKVLAVAMSLVFMQHNDAELRYLRFE ASNRNLDAFLTGMPTYLILVEAQFRSLHILLHFEKLQKFLEIFYANIYIDPRKEPEMFRKVDGKMIINRLVSAM YGAVISLYLIAPVFSIINQSKDFLYSMIFPFDSDPLYIFVPLLLTNVWVGIVIDTMMFGETNLLCELIVHLNGSYM LLKRDLQLAIEKILVARDRPHMAKQLKVLITKTLRKNVALNQFGQQLEAQYTVRVFIMFAFAAGLLCALSFK AYTNPMANYIYAIWFGAKTVELLSLGQIGSDLAFTTDSLSTMYYLTHWEQILQYSTNPSENLRLLKLINLAIE MNSKPFYVTGLKYFRVSLQAGLKILQASFSYFTFLTSMQRRQMSN
TcasOR321	MVTFVPTCEIVAKKMLVLCLGFIDFNQKCLDQTDNKIEKITMEKSSKLSDKFVFYWTFNCIASSLMPMIASLL GGNKNLPMVVWYPYDPNKTPYFHLTYIWEIFCISNLGLIYAVLDLVFPCIAIVLGQQFKILASNFKNNVYRAL VDSEVSEKIVQTFSKNLHNDSFNEEIFEIMNSAKFKKNNARYLRKNVKHHQQLLQYCADVSDILSIFLMGKV SAAIFNTLFMAFSLITTGNRAMIFGLGSYMVSTSIELLIYTYSGQVLTQNADIVGTLYESPWYMCDVHFQRTF HIVQMRASKIVNVKAGNYFTMSASSYITFMKSLGIVYSALEGVN
TcasOR322	MVTVAVLTLTYVFTHEVVTQTDFIIAINSYIVSMTFCAAAFKILIFLVLQKEFKKLFLMVEELGDLDMYAPSTK DHFFNCYMYVTLVLTNPCTWSLWHLIAHNDIPFKSQYPWGDDGVGYLLSFFFGIMAAVFCGLSHILVDTSF MMVIAGITLHVDKLSQSLSLLGKHRFKDSKIMSAGIDKHAQLLRVSQHLSTCYSNLFVGQSVYTVGHSCVLL FGAVHVESKVEVVMSLGTMLVTSYCQLLVYCYYGELLTSKFSDLVFDSYNNAWYDSDLQVKRALPKFSLMC HRHVSLRGFGKVIPSKSNWLHSLQESVSYFLFLKTISGEE
TcasOR323	MVTVYEYEEMMKPIKMVNRLISIWPLEENDNSILSRLRIFHRISMFILILIQTVAVTADIVHHWGSMKEVTECA

	LIATAFYLCVLRLTVYTIHDKDLQTSVQIMKNDWIKFSGEDELTLKEKCLPIIKLAKFFIMTVFSTIGLFMVAPILEVKILGMEEKKLPFRGYFFENQTITPAYGGLYLLGVTAGGFGGSMIAGATTLNLILVMHGAAKFMVVRKNIESLKSNSENSITFIDCVRGHQDAILFAERVENTINVLVLGQFVISTGLVCFAGFQITEMAEDRGQLMKYTSFLNSAIFELFLFSYSGNELLTESDAISQSCYASNWVGTSFAKSMQIVMTRSLSPCKITAVKFYDMSLANFSSIFSFSFSYLTVLRTMEAE
TcasOR324	MVVEKINLREPFENVTRLLKILGCWYFPNESLVYKMYKNFALITCCMYTVTSIIYSFKYMSIDYDKAYESLEIGVGTAEGVLKGIIFRMKFQKITESWQQIQQPEFQPRNEKQKMLLRRYIYVTKFLFKVYFFVVYIVCVTGLIVSSLLRHKDLPTDHWLPFDYRKPFLHQYIYLHLTAGLYLNSLTNCAVDSCFYLSLLHITAQCDVLADTLKNIHDLDKLNAKNAPERENKDQVMNKILVECMKHFNLIKKFTNQITDCFKEILTLQFVPTVAMICMGMYKISTLQASSSQFWFFVCTDLGATTQIFIYCFVGNLVTTTSEKLFYATFKSQWYNASQKFKKNLLTFMMAVQHPIIFYGWDVFAINYETFKSIMRTSWSICVALKSTQDL
TcasOR325	MVVEVNSRTYYGIEVVYFKFIGFWQFLTNGLGKDKLVISSIVYGLMFTFFIIVQILDMFIKDYDFSIFSEKLSVNLTCFESVIKIGYYCFKRSSLLELLPLYRLDLLLSAKHSPVISTEILMANRRFVNGATKSFVVMIFSTVGIWNCLPLLKCFTSGGCSTLQIMPTWYPGDVSYVPLNLFVYIFEFFIMIYCAALLYNVNCFFSSLALTASAQFELLSNNFANIESNAERRIEDHASSTDEDTKKATMYVLLRECLIDHQTLLGILQKMEDVFNPMFLFQMLTSTFTICLVLFQLNFHTASGDDLPIAMACKFVMYLVFGSMELLVYSWGGQIIYNKSEEIYWSLQKCGWEVGCDKFKTNVQIALQRSQFPVTLTAGKFYVVNLASFSQVIKASYSYFTFLHGSISNEE
TcasOR328	MVVFGRGVEAVLGPSAKLLRLLGLWSPQKGDTSHNGSALTGYLTLALIFGLMVTSALKLLMDRPRELDELGACIFIVTMLAEVFFKMLCFVVQRPTLHKLVQLLTEIRADGSTGERNDEIRRGYQILVDRMFLLIMVTATATQTLWAAAPVIYQPLNEDGEVTRLLPLSMWLPLDMNASPNYEVIYLVQVLLMPLASASLLFDFVFIDLMVRIAAELEILDYSFSGLSKNPKSVSATSKNEFKSVHTVSDGEINLQLAKNVKHHQEILRSVDLLEEAMNTGVFIQFLASTIAISCNIFAATSVTLYDSQFKQLIIILLIQSGLYCIFGQVVTDQSEKLMHSAYSCEWVDCDTRFRRSLLTFSVGATRPIEFTVGRMYKLSRETFLQVLQGSYAMFNMLYTFQSNR
TcasOR329	MVVKESEIKVSRVTRKILQYSLIWPKEGDEINPGKWYYIRIFTFLSFTSLWCIAICMHFIIVLKDKIDWDVTEEIAIIIAIYGTYYMVLAYVKNQKKAARILRDLSNFERFGVPPGFEEEEKRLKVYIIGIFIYAFLTITFYNFFKLSQKGACERFNEEHHLDENCGLLSPVWIPFKVDRFPQFELVFLYLFTCCHLLMKLPLVVSYNALEMVHHIILRINHLKIMITECFDEPEYEISRRKLTQCILYHIEILEFATRVDDCFSNCMFAHLTLTGAICACLEKQIVAGISRFGAILHFIGWILALFIGCLGGQHFINASDTIPESIWASKWYNANLRLRKDLLLMMMRSQRDLHITAGPFGVVSYALFLSVLKMSYSILCVLTS
TcasOR33	MVVLKDIFTILFYVGLWKPATWHGRKSILYTLYTSCIVIMASTFLITEVMDLIFVTSNIVEFTNNVFMMSAVISSFIKTIIIIRHRKIIADIIDVLKIYLSKISGNEEIIIIDRYTRLIKFMNRSFLCTALFGVSLMVYVASSQNISQHILFYRAWLPYNYSQPMAYWMTTTGQVLTIYVLTIIYTVFILLFSGIMFNICAHINIFKYHLQITFSDEYYHSRNDKRRCIS
TcasOR330	MVVQKIDLLEPFDNVTRLLKILGLWYSPNETIVYKIYKNFVMATCFLYTLTCTVYGFKFMSFETLEIAFGAVEGVLKSLMFRLKFQKIAESWQQIRQQEFQPRNEHQRTVLKWYIEVTKSLFLVYFFGVYIGCISALTVSSWLRHKDFPTDHWFPFNYRRPFLYQYIYVHITVGFYLTAFLNCASDSCFYLSLLHITAQCEILADTLKNVHDLHKLNAAKKNSGQKGEDEVMNQILIECMKHYNLIKKYTSLVADCFKEIITLQFVPTIVMICIAMYKISTLEPSNTQFWFFAFTELGAITQIFIYCFVGNLVTSTSQKLFYATFESQWYNASQKFKKNLITVMMAVQRPVIFYGWNIFAINYATFKSIVQTSWSMCVAFRSTQDL
TcasOR331	MVYLKDPFITLRVMFLNFNKYKIVKCCDFSFIIFYSLVFCLQIYYLISYFSANPLIRYATTILLVLWGIVGAILSVTLEKQILEATAFLDEMCWPLNMVRKEAQTKLERSCRIINIYITCSLLLILITVVFNMLCFSSQRDFFINIQIFEEYFGELSHVFNGLYFTGFPYLCYHGARLCYVFVYAILQIQLQFSLIEEYLLQVYEIDCLKSWRYLRDTRYQQEMGKSLRLCITHHNALKKFVKMINDMSLICMPFCLVLGVLILISCLAFVINFGDTLTIFVKLRILIFVVSCLCVLSVFCWSGQQLTDVSSYIFLTLARAPWYYWRLENIKILLTFSTNCTKNDSIVLAGIRLEYMLFVSMLRISCSYALVLFNLRK

TcasOR332	MWDMRQLRMMNLWGWWPKMIKDPKKRKIMRVYGYCSFGLDSITMIAEIISLYLAVVNGSFRGAIINIVTTTLGTMAAQKIYTMLVHHEFISHICDTLEDLDNRAIELMGEECQVTMKDRERRCLLTFVFVGSCMFTVCHYNVRPIIVYFLYGERTIAMDMWTPWDEQTSETGWIVVLIYEWIHIFAAMYGMTVFDSLFLSIFEMILAEFDVLKIALRKINFAAEKKEVTLEFCIKFHQDLLLLVARINEFLIPIQTIQCVMFTFTICFSGFELLSLSDGSLNKMANLVEVVGAATYITFGYCYQCHCITEECEEVVRAACDNNWYEGSVEDQKKLLIILERAKNPISFGNIIKFDLGCFIAIFKTAFSYYQVLQAFDI
TcasOR333	MWKCWKKSSAPPPSLKDEAWKWPHESMMNWFGWWAEELERPLVVKLLQVMRAILIPSHLIFYGSLLYQTSNEFRQGTIISTVKSAFVSGPSTVACFKLYVIVRHRKSLKEITNSMDVMMKGILSRHIPEDLEKEMRSRWTGCRKLYKCCVYFGCSVTTHASVTPLLQTIAGALLTDDPLPFDSWPYFLMGYYFWALNTFCIGHVLYMFDATWFAMADNLQIHFAVLKNYLENLDLTKRSDVDLNLCLKNHMELIRLCRIFRRISRTVIVTTRMCSMLLLCAGTFVLTSAGDEFTSNDRGNLLSTLIYIAAVFFNYCRCADNIAHQLDELTTDCYSAQWVNAEKSQKTSILNMMTITRMEPKFCGIASIDLDTFVNVMRGVYSYYNFLTAVDVGDESETSRTEVNEPL
TcasOR335	MWLKYPSDEELNRLGICRSTFDGNVFFKAQLGGLCWFSNSWSVFYFISTIFGIFSGCGFAYGVFMEKEWEALYEALHYIPLVVNITSTAASYHYTQDEYLQVFRSIDKEMFDYEGTLDEQAVEEIARMKTEARARKKKISMIYTKLMIVAFICQTLRKPLNYIIDGRGKKDVDGENNLIWDVCPFGIYMPYADYWAPYLIGHFLCWSCSAFSAITAVASALTYQAICEELLADYSALDLTLSTIVQRAERLFSNMNRGFTGNASGTVTFDYCLEKCLKVSIKHHHEIIRLFNIVKKLLYIPLFFTIFDTGIVMCFSGFIMISDDFSPKFKLLLSPVMLAESGIAFMFCYYGEELTEMNKNIGNRIYFSQDWMKHFKSIKPYALTVKSYCDIPNELSAGGFTKVNRLAFSNILSAAYSYVGLLLTTS
TcasOR339	MWLREWFKNRSSKTASKEKQVVPVNIDGTPDFASFATCLSYQKFVGLYLDGSVINYLKISIPILFLTTGCISFAMADILNYKNKNIIWLIENGHWCIVYIAAIFWDTQMGIKSPLLLRMSRSVKSGVYKYAKYDTINREDLEKTNSQVVTTSRFCVFVYVVAVLATLVKPTILEEYDPYRHFFNGWFPFEVNSLWRVSIVRVYELGCAWSAASGVCTFFVTFMAYSYHIEAHLKLLIQKIEKVFDPESSEYDYIPQLDKKIRECLGHHREILRVFNDFSEFCDPTIGCATLMATFMVCTLLYLMTNPDFDVSVIVTFSGVVAPEFSLLISFRLRGQRITDLSNKINEAIYKLKWLDQDVKVQKNVLMWLRLTSKPLELKSFGYRNVSNSGIKEVLQTSYTFFNMLKAST
TcasOR34	MWNNNPFIVIRTIFLDINNYKIVKFCYVSLTVFYSLVHCLQFYYIIKNFNLNLIIRYGFITSLLSYVLAAGILSLVVEKRIRKTQIFFDEIGWSLNIVGKDAEMKLEKKCKLINISIYAIMLFLVITLLVNLPFVGSQRDLFLSIQVFEEYFGKWSEILDRLYFTLAPFLSYHGARLSFTCIYAILQVQVQFSLIGEYLFETYQVDDSKSWKYLQDTRYQHDIGESLRLCVEHHVALKKSIKMMVDVALTCLPFLVLLGLSTLISCLAFIMNFWDTMDNILKLRIFMWAAWIVLITIMFCRSGQQLIDATSDIFFTLGGAPWYYWNLDNIKILLTFMANSTKNDSISLAGICLDYPLFVSVANTTVSYALVLYNLRESSLDSSNKK
TcasOR340	MWSLLPVLNGWTWQKKLPFPARYPLDVTKSPYYELAYVYQFICIWYITVANLNLDTIIIALMMYTSCQCDLLCDDLKNLTETRFFDKKLIECIKHHKAILVFAEKSNSLFNMIVLGQIATSTVVLALTMFQLSMVSPLSSEGLNHLFYIGGIIMQILLYCWFGNEVEAKSSNILYAIYESTWSEASKNSKKNLIIFSIRCQRPIKATAVKLFALSLRTFITIVRSGWSYFAVLYNVGSE
TcasOR40	MYEFEDPFIWLRNSFHINESNNTMTKKIYVLLILIYSFAQCFQIYYMFKNFNMNLLIRYGPAIILFILVILVATLFLYIEQDMFKALTFFDKISWSFSMIREDAQIRLKRKCLIINICILFILLLVVTTITIGAPYFGNQRELFICIQIFEEYFGKWSPIPYYFYFVGFPFLYYNFFKAWTVFVYGILEVQLQFNLVEEYLIESNKINDFKGWKHLQDTRYQQEIGKSLQLCITHHIALKKLVNKIINFTMTGMPFFLVLGVSLLICSLAFIINFAHTMSNILKIRILIFVATNVCLTILLCWIGQQLIDATSNIFSSLVGAPWYFWNLKNTKTLLMFLINCTKNDSIVLAGICLDYRLFVSISRITVSYALVLQKY
TcasOR46	MYHKLLNKLVRILRYNGIWPVESTVRSYKLLNLIFRLFNLSIIVIMMLLTIADAIANFNDISLITDNLCFFVGCSEALTKGIKYCIEYKNIVKLMNDIYGPIDIINKKNNTEVMKGINEIARFENRQFKIIFGIVSLLIVARVLGADFKNKGFPIRALFPFDATATPYYHLIYLLISYGVLLVDYTLLGVDLMVVVIMRYLTIQVDILRANCRHCDIESTRRNIVINGYDDKNNENTDIRNFVGFEIEHEDSDGKDSFDDRLKRCIIHHQKVIYMLNGLNDCFSFCVVVQILGTTVLLCLNGFQIIMGRDIHLFMRRVLASTAALLQLLLWCWYGNKLSAAADSLTINLWMCGWEDNYKHGLRNFIS

	IPMTLSLQTLELRAIGVVPLSLQTFVSAIKTSYSVLVLLLTVAKDE
TcasOR47	MYKQHKKQHFDKSDDPFINLKHVFITYGYHKLVKYYSGFAFIFHTCSMVLEIHYMIKHFSMDLVTKYGGAI MLMNYFLISQIVTVVIEKLYLPELVKIRDLVFWKIDSFGSSVKEQILNDSIKMKRKLYFVWILFAAFGIILLPICG DFDESHLFPKVYETYFGTFGTIFYYFYVSSFPFVLYTSLRIPVFALYGVLQLHVQIILLNHKIRQLSHGLVDIDNV RYQERVSQNLCLCFSQHVALKNWLSKCLKFMKNVMPVYFCLAIICIVIIMFFILNNLVLNTSTHMKLRFFICG ICSSVVLYIFSEAGQLLSDDTGGVFQVLMECPWYCWNTKNRKIYTMFLLSAVKPLTVDWGGLILNYNFGNH VFRTCSSYALILYKLRKAK
TcasOR48	MYLDLNKGIADNTVLFRIMGHWPFGNPKLYRVYTHFVLINMYLYNLTSLINMLKNLDDTEEVTATIYNLLST VAVIIKANIFHYHFNHVKTIVTMFESEAFQPKNKQQEKILKNGIFWARFIFYYFFLTADLTLVMWILFPIMDGE RRFPSNAWFPYDYLSGRNYTLTYIWQSIFIIYHALSNVCMDTFFAMLMVQTGAQCDVLNNQVSLLGKESVD SDTVRGELGKCIHHHKLILKLAETIGLVFRNIVLVQFATSVSVLCETMFLLSLVKTLNATFVMLLFYQVAIFTQI FLYCWFGNEVVLKSAKLYYSAYESRWYECPQSFKKDLLFFMQRTQKPIVLFVGKMFPITVITFTSILRSSWAYF MALRKVHDKS
TcasOR49	MYNSFQKYPLWKVDTVGTAYKKIVERDALYTKIYIVVIIVIGFCTGILYVIPTDEDHEMFFIMTWFEDNLLEWA DILSLCHRCSFLFVSLLMQAPCFQICYLLKHAEYQLGIIQKLLENIHYGFEDLDEMTCGDTKVQGEIRTRLLFC VKRHVSFIVIIKKHIREAGVFVFLFSVTGGMVAVSMLLFFFLNEENLTFNHFRFWALIVVAFLTGVQYVLQGQ SLEDITVEFYETLTRIEWYYWNKENKIIYQILLINASDPVTVKFSENVSVNYKLGIDVGKAVYSMMSFMTYIRD SVLTRKG
TcasOR50	MYPRFLSRNYPLAKHLFFVTRYSFGLLGLRFGKEQSWLHLLWLVFNFVNLAHCCQAEFVFGWSHLRTSPVD AMDAFCPLACSFTTLFKLGWMWWRRQEVADLMDRIRLLIGEQEKREDSRRKVAQRSYYLMVTRCGMLVFT LGSITTGAFVLRSLWEMWVRRHQEFKFDMPFRMLFHDFAHRMPWFPVFYLYSTWSGQVTVYAFAGTDGFF FGFTLYMAFLLQALRYDIQDALKPIRDPSLRESKICCQRLADIVDRHNEIEKIVKEFSGIMAAPTFVHFVSASL VIATSVIDILLYSGYNIIRYVVYTFTVSSAIFLYCYGGTEMSTESLSLGEAAYSSAWYTWDRETRRRVFLIILRAQR PITVRVPFFAPSLPVFTSVIKFTGSIVALAKTIL
TcasOR51	MYRQVPPHDPFGSLKFFFIDFGYNKIMSYYSHFGLIFHTCSLFLESYYMIENFSLDFFVKYGCAMTLMFYFIISQ FVTIAIETLVHELEEESKLFFWHIDSLDSKLQRKILKKSAKTNRKYCLLWSPFAVLVIVLLPVWENLNESHLFP QVYESYFRIWSPVFYYVYVSTFPLLGYTAIRNPAIILYAVLNLDLQIVLLNQKILQISGDNDFECVRFQEKVTRN LCLCISQHVALKKWTNKFLKPMQKTIPVYSCLGVFCLVTIMFFILNNVNTNVSNHLKIRLLVSGICCSLILYTF SETGQLLVDTTGQVYNALVECPWYCWNIKNRKLFQMFILDSLKPLKIYWGGLVLDYSFGGSIIKTCCSYALVL YKLKNAK
TcasOR52	MYSILPGSFVVLQAIGLWKPPEYNNSPILNYYYRLRTFITFFLIYSFTITGITGLILTTKDIADVTSDCFILLSIFAIC GKIANIIWSRNEIIWIIDTLNSEPCKPLNNDEIIIQQKVDRLIWHSTLFYGILTEITVFMVTFGTLLLQLPIGTLPY NTYLPWDYSHGYLYWVAYGYQIISVCLSANSDIGFDTLVPGLMLQITAKLEILKYRFINLVDTLKLTQWNGV NDKSYHNFRIENKLIADYVKCHLIILKLADTINKTFDKVILLQFFISSIVLCISVYNLAFLDVFTTEFTSIILYLCC MLMEIFILCAAGNQVTIVSSTLSDAIYHTDWINLDTSAVKSLMIIMNRGLKPIIFSSGHIIKISYDSFKTPIKLSYS SYNVLQRT
TcasOR53	MYSPEEAAELKRRNYRSIREMIRLSYTVGFNLLDPSRCGQVLRIWTIVLSVSSLASLYGHWQMLARYIHDIPRI GETAGTALQFLTSIAKMWYFLFAHRQIYELLRKARCHELLQKCELFERMSDLPVIKEIRQQVESTMNRYWAS TRRQILIYLYSCICITTNYFINSFVINLYRYFTKPKGSYDIMLPLPSLYPAWEHKGLEFPYYHIQMYLETCSLYICG MCAVSFDGVFIVLCLHSVGLMRSLNQMVEQATSELVPPDRRVEYLRCCIYQYQRVANFATEVNNCFRHITFT QFLLSLFNWGLALFQMSVGLGNNSSITMIRMTMYLVAAGYQIVVYCYNGQRFATASEEIANAFYQVRWYGE SREFRHLIRMMLMRTNRGFRLDVSWFMQMSLPTLMAMVRTSGQYFLLLQNVNQK
TcasOR54	MYSSNSTQVIADFFKQPCYRVNVLTLRICGLWPYQSKFEGKLLRFLWAFFVSSQMIPQVCVCITDFGMDTLTS

	TIPPFTVAIIAGAKMIISTLKVNQIIELLVTIQKDWSKLKSETECDIMKKHLDQGKKLTLFISAWYYNSMVVFLL<br>LPMRPKVMTWLGLSKGPADFDFPYPVNYGVDHDKYFYAIETHISFCSTLVITTIIAADTLFIVFVQHICGVFKII<br>SYRLENLVSSSSLDIDLHPNKINDRAFWVISDCIKKHNKAIQFAELLENSYCWIFFISVGFETLLMTFSGVQLVS<br>QMGNIDGLFRYGPFAFGQLVHVFIENAISQQLINYSGEINDAISMIKWYTLSMRSRKLLTFMIMRSQVICSVSA<br>GKMFIMSMETFGMILKTTMSYFTILSSMQDD
TcasOR57	MYTYFKVLVFWLNKDKVISLQKILHCKEFKPKEPEHKEIIRKSIRKARFVMTSYATMCVGAVSVGIILPLTENF<br>DILPTNVEYPFFDVYKNPTYAYLYLHHIYYKPATCIIDGVMDTILAAFVASAIGQIEILAFNLRNFDVLAERRR<br>KRAISGNKYIGKYTNLYFTKRILKECILLHNSIIRYVSVIESAFSLASALQFMLSVMVLCLIGIQFLSIENPTSHP<br>MQMVWMAIYLTCMLIEVFILCWFGNELIWKSNDLRQAAFDGPWRNLNRKTCMFIIIFMERCKRPMRLSAG<br>KIFTLSLDTYTVLINWAYKAFAVMRNMKK
TcasOR6	MYVFFIILVPTLTIYNYIIQENFDILQFNYTATFLAETVSWIAKLLPFITNANRMKKCITYFGTRYFEEMLQRYTS<br>AKIMKECISVCRRNSTVFLYGVICGMTSFITKPLFWKGYQLPLDMWLPFDATSGPGIYYTTYSFLAIAISYCAF<br>AGTLIDPLIGGLACHATGQLKVLKLSKIKTFGYYFIQLVFYFGVILAQIYFYCFYGSTLFEESSSIINAVYSSKWY<br>DFDVPCRKALLILMERAQTPITVAAGKIMDLSLVTFATILRRSYSLVAVLNNYQ
TcasOR60	MYYSPWMANNCIMDTQEYKTARKKDYWYLFEMSGMILDWRPGFYIINLAYVVVMVINAVYTCACLIASIF<br>KVDDLIDVCQYLNFVGLLFVSLSVLASLNFQRERTLETCAIGSTEFFDYGVSFSRSEEIEQYRKEGRRRMKILF<br>MVIPPWLTIIALSLMMSGPIDAAFSYPKINATYVNGIYQMAPLKMYYLHPIDNEFIRWLTVLSQAACSGNTAL<br>VIGCADLIMFNAGQNVIIQLEILNLAVLDTDKRASKLYELKFGREPPSDPVDKTNDRPLMNIYGFCLKQIVD<br>HHKIILRRAEVYHKIVNWPCGIVVVNGSIVVAMSLLSIMQGGGKPSVLVLSFFLIIAEVASIFMVCEIGQSITSQ<br>CERLFDSMYAFKWMDCSVEVNRAINIMKCRFRKPIIMTAGSLTPINRDTFGTMMNTAYSYINLVAASGKEDA<br>D
TcasOR61	PAAPRQLPVHVWLPADLNRSPTYEALFAAQSFSLMVLSQATVCMDIFFVHLMLLVAAELEVLNENLSAMER<br>GRLQHGRSEYSETTDIHGEDSDRSTFTNTDRRLDVTVGTSQREHDERMYAELVKNVRHHQAVLRSVSLLQK<br>AMDASIFILLFINMANLCGAVFVAAVLLQRDGNITKALKEVMLIPCVLYETGMYCLCGHMIISQSERLVTSAF<br>RCGWPDCDRRFKSSLLIFMMAAIRPLEITVGKMCKLSKQMLLQVLNGSYALLNMLYHFHHTL
TcasOR63	PGAVADWQFPVPHWVPVDMQRSPTYHLLYVLQSFCLLVASQSTIAVDLFFIHMMLMLAAEIEVLSENVSAM<br>GKIDSGLMALENEDCGLSTKDLLSYRDGNGLVSEIFQKEYISEDQMRALLVKNVQHHQTILQAVGLLQDAM<br>DISIVILLFTNMADLCSCMFASAILLQRGGNAAKALKPLMTIPPCFYETAIYCFFGNIVTEKSEQLVTAAWSCG<br>WPLCGSGFRRGLLLFLTEAARPVEITVGKTFKLSKQMLLQVLNGTYALLNMLYHVHRSE
TcasOR64	PGRRLRHRLVLAFVVASHLGCFFGALASITMDTPSDLPQLSFIAYNCLTDAGLTCKMLSFSLDGRRLTELLRLL<br>SESRRRFPDRAGHRARQHATAVRMHRFLQVTYRINTAYWLLAPVVRNIVAVISNKPYSRDIPIPIWLPFDIRSS<br>PAFEILYSLELAFGWAISETTVLVDGSLIAMILQLAAELAVLNDRLAAGTTPAVKQTPSDAATTEEGTLKDSRV<br>RMPTKSSSAFVETTCTDFLQSRSSIGSEDEMYQQMVGYIQHHQTIISCVRLLQKILSRATSVLLFCNTISICFQVI<br>ATAVLLQEDGEIVQTLKMLMGSTLYAYQVALFCLLGQRIINQSERLIRSAFCGDWPDGDVRSWRLVYMLCMS<br>TKKSLSLRICGIYTLSRMTLLQILNVSYSLLNFIYQTKTEQSSGKQEL
TcasOR66	PGSTSAAGLCRERVDDLEEMSEGIFICTTVIVSTARMLYFLAYRMRLQRLASLLLEARRCFPVQGAALRSRYQ<br>RHAANVCIGFQATAVLPVSLWVLDPLLTAAVTSQNTNNASADAAAEASRLPLSLWLPVDGRQSPSYEAVYA<br>FEGFLVVFTAQVLLFLDMLFIVLIIHITAELNVLNDSVAAIREAVAGGGETGNGTDASGKLDNTYGSVSRSDS<br>DMYGQLVEAIRHHQTVMRYVQELEDFMSQPLYILLFTNMMNMCLHMFTFVVLLQKDIERSSMVKMMLTFP<br>AYLYQTGTYCIFGQTIIDQLLKDTYTMFNMLYTLQGNK
TcasOR67	PLLQHKAADEFSGAEKVERQMPIPSWFPFDVQRSPAYEVVYAVQAVCGTAAVQLSMLLDASFYQLALLLTA<br>ELRVLNDNLALVGAATSATGGTGVAGRDSHHQHMPAPKLTTSAVTEDNDRSRTLLYFEFVENVRHHQAIM<br>KCFQLLESVLNYSISILLLTNILTMCFSIFFASVMLQADGGLRRAMKITSSIPNLLIETGMFCIFGQMVVDQSER<br>LPQSAYSCSWVDSDARFKRALLIFVLRTSQPLEFTVGKLIKLSRETFLKILNSSYTLISLLYQFQESND

TcasOR68	PPAAGLLYRAYTAAVVGLLCHITLPEAAGLVHFRGELRTATEVACLLFAFATTCYKLLAVLLRRRRILRFVHDLDARVAAMAEESAEARAAVSRRDRWTRRLAVLMVLQSTSTAFTWSMNSLRLSLSKGCSLRFLPIISWYPYDMTVWSNYAITYIIQFFTLIASAFSNRTCDILIITLMCQVGSLLEMLNLRFLEISRDSQSKADKRRTEWNQKHIMKRATSVLCKTIGANGLLDHVPTTSEEVPQDEMYAKLTRCIKAHQEIIRYAKELESLVNDIFLVDFLCCMIVICSTLYISTSASSNFGDLMAHFGYLVAMTYPLLFYCLFAHDIMEQSGRVAVSAYCLPWFLGNTKYRRAVCVALCRSQRPLTLTAGKFSVVSRATFLAIMNASYSYYQILREINEVKRSE
TcasOR69	PRLAAAFRESCRWSAKLTFLFFSWVLLALVAWSLMPLTLYPRVRLFPFQQLPWPVLTQSPTYWFLYLHQILATFFFCSIDMNTDCFFATVMTHMSTQFKILASRIADLRLRENTQKSKLCAEVDTSTPHDEMYKELCLCIETHKELIRLVGLLESLMNPVAMLQFLVGAVSSCVVLFSATYSPDSSSAMKCWGSLPLLLTQLFLYCSGAQHILDESE
TcasOR70	QAYIWFGVGACTFFLFSPASAEGLPYILALPFDASQLVGFAVAWLFCMVVTFHVVVMTMVLDSFNVSIIAQLRMQLALLNRKIVSLAKGVNEKLQQCSDTSEYSDLHSRLEKCVLHHQAIIKNADLLEKCLGTMLLGQSLSIGAAACFQMFQIATSANGLQQTGKFCCYLFAMLAELYVNCWFGDDLITESENLALAAYDAVTSLHGCPISIKRSLLLLMQRAQRPLCITAGGFFPLSRESFVAVLNVSYSFFAILRNFKNEDQ
TcasOR71	RGRLARLLGGVVRAATMFGLVFMWLGTVLKLCVDPPPQLEQLTLCSLVSSICTGFIIKAAFFLAFGGTLRQTVRLLADTRARFCTGDHNEATRRRYHKQSNNIYYFIQIVAAIAIVGWILCPLVTHILAKTDEDHPEARMQLPVPVWLPGDIHETPVFEMLYAFQSFTITFGAQFCLSIDIFFIHMMLMLAAELEVLNYNLSAMGHVNLPKLGSHGGKSISRYKSSGRQSALLSSGQQLGEQILTEDSGNEWLHQQLVKNVLHHKAILRSVSLLESSMTVSIFGLLFINMANLCSSMFVASKLLQKEGSIGKALNALLTVPSELYETCIYCIYGNVMTDQSERLLESAFHSDWVNGDTRFKRSLIIFMAVTRRPIVITVGKTCKLSKETLLQVLNGTYALLNMLFNIH
TcasOR72	RLLAVAAVSYANGCDVVAVCREGITDFDRFTITLSVFDTGNTWVYRRCHVAWHERDFQKLAQQVRDDFGEFMAPSDVPVLRGLAASLRRFVTAYVLMGAFDTVVWLTHPTRGEGLPLLVLLPFQTQRGLGWLSGWLFCAYITADCLTVNFMVDSLNVCLMEQLRMQLLILRKHIAELGNRSESTKYSLSEKESESAKEVGKPQQGKLAFVNETHKGSQVSSEAANSSDIHSRLRGIILHHQAILRNAEALQKCLGNMLLVQSLSLGSVICILLVQIALSAQGARETGKICGYLFAIFGELWLYCWFGDKLTSEGENLTLAVYDAVTSLQESPTSIKRSLLLLMLRSQKPLCITAAGFFPLSRESFVSILNISYSCFTVLRNFKEE
TcasOR73	RLPFAQHLWDDNGHWYGLSYAVQCVTGLWMAEVSFGVDCLFATVLMLAAAQLRVLALRLVRLKVDAGGAPGDQPGATDGAYRELCLCVESHQEILRFITHLNGTMSPVAMTQFVFSVLVACVALFQATYSTDITAVIKCVSFLPIPGGQVYLYCWAAHHVTEQAEAVSTAAYCSPWVDAGPRFKRALRILISRAQKPLVLTAGRLYPVNRETFLSLVNASYTYYALLGQMNKRSAN
TcasOR76	RQSVTVAERLPLAVYAYIALLCLIWFPMPLLVEPHNPKLPFIQLHYWIDHTRFPVYEASYLMQVVSSFFFIFISTGMDCFFAVVMIHVTVQLKLLIYRITEIRLRDAPAVAAESEARWNRDVGDAHAEMYKELCLCIESHQKILGFVKYLESVMNPIALTQFIFSVLAACVTLFQETYNPDISAVFKCASYLPTPGAQVFLYCWGAHS
TcasOR77	RRESLRSHVTATCLMNCGGGRRLRTDLRLQQWLLFFVGAWAPHRGAPAVCSLLYGVYSACVVLVLLLFVASLLFAMVHYWGHMLGVTMNACLMFTYVMNSIKIVAFLKMRPAIDQFIDELDNCMQEYGGEQQSERAALFGWTALKSRIVSVARLSVTAMGCVYWSVMPAVRARACGDTVRCRARVGLPAHVWYPFSYTQSPVYEVIYAGVAAGLMYGALLSSIMDGFLVSLFIYMAAHLQMLNLMLQNLCVDQPQDGSKGLPPGHHQHLCRWRLAQCVNYHCRIDRSVQRLSMLFGPILLGQFMMDIIAISATAFVAIAKNADSTWLVKYTSYLSAVIQQLLFYCWFGTDVLTESERLQTSAYSSQWVDASPLFRLELRVFLCLAHRPMRLTASKFYTISRETFLMLMNASLSYFAVLREINAK
TcasOR78	RRLTLAVTLLGCPSPGVWTAVPLLAPLVGDGAAPRNRSLPAAARYTARDTESPRFEALTALQFFSMQFSYFTTVGVDMLVVSIMIHASAQLELLNLSFGRLGQAAGSLPGNRERRRAEVCVREAADGPGRSGISRRADAEETPPREKFCQELRDCIRHHQDVIQLVADVERLLTSMILTQVLGATLIICVALFQFATNIENIGTILRVSVYMSFMVNEVFMYCWFAHNIIDQSSRIAESAYSCAWPGVPPSLQRSLLVVICRAQRPLALTAGKFYQVSRETFIQLINASYTYYALLRQMND

TcasOR79	SKAAALLDGAEELVCPNATFLRLMGLWRARGWTGAARGWFCWTLVLFVTATSAGKLCLDTPQELAAVAD<br>YGYAVFHLSAVTVKVACFILQRSTIEELVNLLDETRKTYGKTEANYQVRQIYQRRATNIYRVLQALAVAVICM<br>WISSLVIQRQGLKEGERPPPNPIWMPDNSPGYEIVYSVQSLCGSAAVQASMLIDTSFYKLTLMVTAELQILND<br>NLATLGRAAEA
TcasOR80	SRLYDAYSVWTILQLLMTGVGQMAGLQGHWDDLQTVFTSLCFDLTVTCTIIKGSIFVAQRGSLDALSRHLEA<br>NARQFCSHLPAERRALLERARNLSRVIVCSFQSVGGVTLVSFITGPLVQNGRDRELIASAPGNATLDRHLGH<br>NYPMLMWWFGGLPVSSPGYEAAYLIMCYWLVLMYICTNVPDAYYVGLINYISAQLRLLHIALRQIAHPGAD<br>DALAEKLGHIYGLDVKGGSPHKDGAQDASRDSVYDRLVECIRFHQEIIKCVDEMESLLSLTVLIQFFTSTLVIC<br>LTAITVINTEAAYLPTYAAYLATMFYQLFIYCWYGGEVYLESESLQFSAYSCNWPYTDARFRKTLKICLARMQ<br>RPISLTACKFYKLSRETFLLLLNGSYSYFTLLLQMNQKDD
TcasOR83	SSGLDLLQRLLHWSGTLRHPRAGRWSSLAFYPLNAAASAAIALFLCSQGAAIWREGARDLDRLTLVLSTFNT<br>IATWLFRLGHIAVHEHQFHYLSFQMERDFKDFLNPRDVPLLQASNQAHRRFVLSYLWFGVVLCMVWTLFP<br>VATFGAGPDGLPFIMALPYDVSPLHAFIPTWIFGAFITVHVTMMTIISDTFNVSLMAQLRFQLVVLNEKIINLT<br>TDIETPKSPLTKEEYITTKSAYESVKHTNIHHRLRQNLLHHQVIIRNTEMLENCIGGILLAQCLSIGAAVSFQLF<br>QVAVSTQSLVQAGKFSCYLTVVLVELFMYCWFGDDLITESENVALAAYTAVTSLQGFPAADRRSLLIAMTRA<br>HRPLRITAGGLFPFCRESFVSIVNMSYSYFAILRNFKDD
TcasOR86	SYAVQCVAGLWMAEISFGMDCLFASVMILAAAQLEILSGRILKLGQSPYVAKKGSPTPDEIYKELCRCVETHQ<br>KILRFVSRLQETMSPIAMTQFVCSVLVLCVTLFNATYNKDIITSLSSMTFLSNPCGQVYLYCWAAHNVAEKAN<br>AVSTAAYSCSWVEGSERFKRAVRILMSRAQKPLVLTAGSLYPIDRAAFLSLVNTSYSYYALLGQINNR
TcasOR87	VCCRQLSLHASRPRAGAGMEAHLSPAVRALGAVCLWKPSRAGCWYHAATAAGSLAALSLLASVAAGLPA<br>QWARGDITSFSMNAYVCFAIFAAQIKVSTFGYWGGPQRLVAQLGAERRAAGAAEAALPPGRAERLLGESGT<br>LLRRSAAAFYACGHLMMVAWYASPLIANARLAPDPDTNATLPRHLLFDAWFPFDPVPSPNYEAALLYQSVT<br>LYIAFITTAVIDVFYVSVMVYLGVELEILNEAVARSCRPFEEQENKDKDKEGEKKEEGRGQGDDCSLLVACVR<br>HHQHLNRCVGTLQEVMGISIFVQFVFNMLLICVYAFVITTTKSDFGTLAKFAMTLESYLFENLLYCWFGNNL<br>IEQSERLPFSAYSSAWPDAGRRFQRSLRILALRASRPLQVTVGSLYTLSRQTFLHLLNGSYSLFAVLHHLNSK
TcasOR88	VNIAANISLVITSGVELCADKPRQTERASVTAFLFSVSIINFVKAVSLLRHRSRLRRLVRRLVAVRAAFADPAGT<br>RGRYARHAALLASTWLVTAETNVAFWCLDPLISEAAGGAAAERQLPLPLWLPFNQSRPHSYGRLFALEAAV<br>LMSAVQIAILVDALFVTLIINVTAEIHVLNSNIRSMSKAAASGGGLQSGGTIENTTTSDESTLVANLHRSNVRS<br>SNSAISDISASGRNGKSADDEMYGLLVKNIQHHQLIIICVKELEKAVSTGTFALLSINILNLCSHIFSLVVMLEG<br>ENSVSAITKMLVAVPVFMCQSGLYCLTGQAIIDESARLSTSAFSCGWPDADQRFKRSLRLFMTRAAQPLHIRV<br>GTLISLSRATFQELLKGSYQLFNVVYQVHTN
TcasOR92	VQRPAAGMRVTAVHKQTALASEAGRDLMGPGEAVLRLLRVWPPPGPEARGLLHRLPYPLLALQAGMAGV<br>LASTAAHLHQGGAGGDEESEAQQTTVALFIVGTIIGMLVKIWSFMGQRGRMQVLLSLLLEMRRRYLRDHTG<br>ARPRAQDHGVTLFYILQGNAVLAALAWSVQPLLSGSARRLPLPAWLPFDATVSPYYEAVYAGQALSLLLVP<br>QISLCINICYFALMLHLAAELAVLCDNVAAVGWRRTSKEPAPQSQQQVLDREFAASSENRLLEDNVRHHQL<br>IIRAVSELQQIMSTSVYIHLFVNMINVCSHIFVISVVLLETGEMAVVVSQACSLAVFLSGIALYCVIGHTIIDQSE<br>RLPEAVYSSGWTGADLSFRRSVSILLVRASHPLSITVGKMRVLSKPTFVQVLNGSYTLFNFLYRTQSDKEQRER<br>LGLQDS
TcasOR93	VVQSARRRAYRITLGMLLLMFSQYFVWYPMPFYVDPGARRLPFAQHAWDNNTHLYGLSYFAQCAAGLWM<br>TQMSFGLDCLFASVMVLLAAQLDILARRILALGSGAHDEKAEYPEKKPAPRFGDQMYDDLCLCVQSHQKIL<br>SFVIHLQNTMSPVAMTQFAFSVLVICLGLFQATFSEDFSAVFKCASFLPIPCAHLFLYCWAANNVTVQAEAVS<br>AAAYGCSWVGASERFKRALRIIVSRAQKPLVLTAGHLYAIDREAFLTLVNASYSYYALLSQMNNR
TcasOR94	WRPRGGRAARLLNGLLTAFILASHAFLPVCVALKLYVDPPEELEQITLCSLVTSICMGFLFKAALFVAQGETL<br>RQTVRLLADIRAQFGDRQQNHSTRRRYRRLSDSVYRRYQMVAVPAVIGWVLCPMLSRSVRGSDQAPQVAQ

	RQLPVPVWFPVDVYASPTYEFLYVAHSFCALVAAESSVCVDIFFIHMMLMVAAELDVLNDNLTVMEDVNLY ATPNERRGLTSVNGSTGRQSAIHDSSQSTLGENAAREGIHEQLSKNVQHHQAILRSVSLLQSAMNVSIFSLLF FNMANLCSSLFVAAVLVQRDGNVGKALNALTSIPALLYETRMYCIYGHIMTEQRARC
TcasOR95	YSLLWPVENDELSPGIRYKLTILAFFSITGILVFSISVYSVLEIKQGYDIDVEDVAILIAVYGTYYMVSAYLNNQH QIALLERDLSQFYKFGKPPGFEQLNSQLNFAVKVLIIYSFLGTFVYNGTKMLLREECKKNSQEKGLSDNHCGL IATFMFPFRVDYFPVFYIVLVITFLLAHTLIKLCMHISFNAYEIVNHIVLRIEHLKEMILSCFNERNQTIVQKKLR VCILYHIEILDMAARLDKNFFNTMFGHFALTGAICACLEKQIVLGVNIVAGTLHFIGWIIALFVGCVAGQCLL NASEIIPNALWAAKWYHADLRTQKTLLFMLARSQKELTIKAGPFGILCFPLFVTVLKTSYSILCMLTS

Table S4.

The protein names and sequences of 298 ORs from 5 different species used in Figure 1.

Protein name	Protein sequence
AlucOR107	MEESWLVRYYGGGLGQAEYERVRDFAVSEFTPLVLFLGIFPPTDKMALMSIVVSLSIAVYAFYIVLFTITCSFATD<br>DFVLWSELIHHTSLMYLGIFIRSVLILEAKEMIKLARDYLDGIYHYEEGYVDPIFQQLQDKSRKLQRKLFMLPLF<br>IVLVTGIALGLKPLLDDVNEVEPHPKLLENGITYRSLIPIFYPFNNENTYQVLLMNGALLYFAFLVVVTVIAAD<br>LLFIRVSCRISLEIAILVESLNLIDKRAKRLYARKYGLNKKNESWPLYQDCIEECIKENVKHHQKIIIFYEQFSAVA<br>APAIGGGFFTCTIVLGLGMIVVNMDNVNISDIIAFVGTVFAEMMNAFMISWMSEKIGEQNYELYNAVYNLK<br>WFKWRQSNKKLVITFLDGTRQPLFLNAFGMATINMEAFGSVVNTAYSFLNLVNASETLEEKK
AlucOR106	MKEKDHSKRKDMLSVDYRKYMYCKLIWIDDGLADRGLTTPLLFIMIWMVFMVFFGLVSFVMSTQNKVRLD<br>NLRSFLLESMVTMSIFNEYMSRKSLARLHHFMDESMKTPRTGLPKEEEILKDAKSLAKKHLTAYTVIFTFNLA<br>AMILSQPVAEWIQGNSWKKLPYPWVIPPSHNEFLFWIVFMFQSIGLYFSHCLGVVIMSFSSITIQITALFEVLLLS<br>LSHIEDRAKVMSEREGADYTTCITECLKEDIIHHQRLARELISATPHLRRTFFALSVTISMIMACEAYPLIMGNF<br>TLGELIKGLLFLVVQFMCWGQMCTRMETMADQNTAVFNALYGTPWYDSGVKYKKLMLTSFTFSREPMYIISP<br>LFIEMKATMSTFYSFVVSSFNILNLIRKMN
AlucOR105	MGFFTSVDMTDVQRLRVVEDSKRRSGLSDLIGRCGGYRGPLYNEHYSKNILFRIYVHLTDLAVWINYITMIAA<br>AMKSQSVLEFAMVGFPISAESLSLFLSYYSGYKNAEMTEVLLGFDDCFDDDPYPQHLEMEIRKSADYYHHFSR<br>SLLWLQVFTMQIYCFLFPVTNELMHDFFRPRALPLPSLYPCDWKESRSCFIMIIFIHFLGATYVNWKIIGFGEVF<br>FSMVSRQVALFRHLNHNLNQILTSVHVSTDGTVIYRVDKEIDHIYIKSALRKWIKHHQNVMTQYDRLQTLYS<br>WPLFVHFGLVSGALCCSAYATSDETLDFDANLLCGGFLVGQMLELFYLCRMGDWITIETNELTLALTGSYTFV<br>LDRIESQMLRIILSRVHRPSVMRAVGLYPLNTSTLKMLIQSTYSYYTMLKKVNRG
AlucOR103	MGTSTLGARPTMRPNRNLFSLSLLLMKIQGLETPSHATLKLVSFVWKYWMLYTALHFVMVCLLGVTIGDNP<br>YYLKLETCSGMIAGMSMVYRHFVLAFNRKEVHRLMDRINALVDDVVSVYGEETIAPWEKMCCGIMILSTCV<br>VSFTTIPAYAYSYLKFYTDGEVTAPYEVYMPFERDAAHIHHVVIFQMLSFLDQAITLIVSNTFIGTIVVIVSGVTE<br>KIAKRYKEINRNNFHTLKVTTNWHSEIIKIVEDTNALLGSAIMVDCLLSVVHISVSGYLLVKVGFESGTNLHK<br>YIFLNLLCVTIPSYFCLCGHIISVGRDKLHEAVYQNEWYELTPSDRKTLILPTWMADKGLSLHFKKAVEFNLPT<br>YLAIIKQSYSLIAMLKLMDG
AlucOR101	MWDMRQLRMMNLWGWWPKMIKDPKKRKIMRVYGYCSFGLDSITMIAEIISLYLAVVNGSFRGAIINIVTTTL<br>GTMAAQKIYTMLVHHEFISHICDTLEDLDNRAIELMGEECQVTMKDRERRCLLTFVFVGSCMFTVCHYNVR<br>PIIVYFLYGERTIAMDMWTPWDEQTSETGWIVVLIYEWIHIFAAMYGMTVFDSLFLSIFEMILAEFDVLKIALR<br>KINFAAEKKEVTLEFCIKFHQDLLLLVARINEFLIPIQTIQCVMFTFTICFSGFELLSLSDGSLNKMANLVEVVGA<br>ATYITFGYCYQCHCITEECEEVVRAACDNNWYEGSVEDQKKLLIILERAKNPISFGNIIKFDLGCFIAIFKTAFS<br>YYQVLQAFDI
AlucOR100	MESVAMGEGEKRKTNRSEGCVDYRKFVFCRLMRTDDGLVKRGITGSLLIIMVTTVAMETCSFVSIFMSKQLQS<br>CLDCVRSFLLGNLVTMVLFNEFANRKRLARLHDFLNSSMSSPRTDLPEAQEILNKAREEASSSLRMYIIIFSGNI<br>APMILAQPLAEWISGHSWKKLPIPWAFPPSDSDVKFGLVFTFQCIGVCIANCLGMVFMSFSSITIQMTAMFDV<br>LLLSLRRIENRATIRTQREEMDYKTSLLYCLREDVIFYQQLVREMTSATPHLRNTFLAFSATVPMIMACEAYPIIS<br>GNFTIGDLIRSFVFLAIQFTCWAQTCTKLETMTDQHEAVFREIYQTPWSDSGPVYKRLVYTTLLFSTQPKHIKA<br>RLSNEISATTSTFYSFVVSSFNWLSIIRKMN
AlucOR99	METKEDEYMKPLVQLLKFGGFWFDFSGHKHAMALKWCNIARNVIAFSVWAYQMAYFMGGVSYLLTEAGV<br>FVPICFDEGAVAVIVLCNQSTIRETIRIYRKRFEVFGSTPWAKNIIDSEMNKFNRIFQLPKLMLSVFFLFYSVVPLV<br>YDAVLAYSGKSPYVVPLPLNFLLETPMQRTPAFYMTIYLSYLYFMIIVPRFIAFEALMMYIVAFVVIDVKILVQK<br>MKNLSEKDDGSLFLQEEWNLKDVIDHHSTVVRVVEEHFWLVGLAMMVQNLTFSISSCLVIYLTKTSFNNGDI<br>VLALFCGNFVVLLMILNLMFNGAGVLIENQGEMVLTAIYDTEWYKQPPKVRKEINAMLRQGLHVLKISYWS

	NTVNFETAMVVLNRAYSFFTLINTGE
AlucOR97	MVKLFDELAEQEDEELMGVYEKLYGPALQLSLLFPSWKRENLYKTFGIILVYTVTLLVHFYVLSVSVVMLRDD<br>FEAACLAFHYWLIFVMVFLSLALINMDRRTFSFAHRCLARDMGNYAAGRIYSESKPLALEARKKKELFRFLVL<br>PGMVVILAAALLVVPYLKKINNPPHYNAYGVNMNLPLATHYPFPTDHGILHGVVVLGQLSAAFSLAVIVVSL<br>ELLLFRVSQAIIFEFKILQYALETLFERSERLFFQLHPDYYGKLSHMNSNYQKCITRCIQDCVKHHYKIQELLQA<br>YEYVLKWPAALGYGIGTGVIGLGLVTLLMAKEKGNLENVVIFSLLIVAEVLNMYIVSVFGEDITTESAAVRDEL<br>YFIEWYKLNIPNRRMMLNFQVGITNPVIVKAGGLVALCMDTFSSIMNTSYSFFNLMNANPLDGNK
AlucOR96	MAKLFGFHIINADSRKCLSYRMSFPMTTLLMAFNLCMLSGNAIGCLIAALLDSNFDRRLMNVKGMMLLIMII<br>LLAINESFISRNRVNRILDYINRIKSITRYGFKEEEDIMNKAIDDCYKSTKYSTTFFFTNSMLMVTLPPTMAIITGE<br>SWKQLPYPWVITQTNDWLYYSSLVLQVVATALCHGVGAAGFSLLTTMKPLAAAFDKVILGINRIEERAARK<br>MSEEGITYQESMLSCLKESIAHHQEIVDELLMEKPHLEIMFFAQVTFISIVMACEAYPIIMGIVDVSGLIRGVLFL<br>FIQVMCCGFLNLEFDTIANKNVEVSEALYGTPWYALGVEYRHVVLNSMTFSQNPIWICGMGFFGLRASRATF<br>YSAMVSACNMLNMFRKFA
AlucOR94	MSNQDDVGDDVSVRRIPRSAQKRPQITVKNEKEFDGGCTDYRKYIFCRLMYIDDGLVSRGLTLPLIIIMVHTV<br>TMEVCSFLSIFGSSQIKDGLDCVRSFLLGNLVTMVLFNEFANRRRLGRLHVFLDKSMRTLRTGLPEEEEILKRA<br>RDQASSNLKMYIIIFSNNIAPMVFAQPLGEWFAGRSWKRLPIPWSFPPSDTDLAFLLIFLFQFIGVIMANFLAM<br>VFMSFSSITIQITALFDVLLLSLRHIETRAALRSKLEGTDYRQSLYNSLREDITFYEQLVREVGSATPHLRNTFLAF<br>SATVPMIMACEAYPIMLGNFAIADLVKSSVFLAIQFLCWAQTCMRLETMTDQHAAVFQALYNTPWYEADLK<br>YRKLIFMSLTYAAPSKYIKARLSNEITATTATFYSFVVSSFNWLNLIRKMS
AlucOR93	MTLKSYIKKTLKWEEPLGLTTMIAVISGAWNTMAPPQSIQRFIYWQSWFQTSTYVLFMMSAGINIFVSTDFFGE<br>CLESLHFLVTAFHVFIKYMTMRFRERDFLELFDDIKRVWSGYRIHNEKFLSSTLASVNRTTVIISVCIINVMFVNI<br>GAAVLKNILEPDKIHFPIQIWIPSFCRDSFMYGTIAQVVLFSWPLFIVAQSTTFLNSISVHVEALGLSLAKDIGRQ<br>KVWKGDSARRFYKKHQEVISIVSRVNALMAGNWGFEMLCSSLQLTLPAYRTLRALKRNEIEVFNHAVILSLN<br>FMVIYIIFGSGNRILSMGEEINDRLYESDWYKLPVKEKKNVLFMLFRATKPVEYRYKMIHFDLPGFMKVVNTV<br>FSYMALLRFLDGGNDGGNGGLL
AlucOR90	MVASTSKNRKIKNEQAVRGFTRSEEKQIEDHLFSIFNILPIIGGIFGYHQSPKWSALTYTLNIGMYTSVSLTALNL<br>LYCSYLLRDNLSQVTMAFHCFLISCVVMTASISLTLQRNKLIEFLLKLQFRGPLAEYHDSDYFQALEGKTRQRI<br>FRMLVIFLSCYGACGLIAVIFPFVDLYLKNADQVTNVPEIYWKGLPFAVWWPYDVHNSTSAWILCFLSQGIWA<br>LFAPVIVTTAVVLCFYGAELILNHFKLLIFSVKNLDQRTKAMYERKYKENSRTQLENVYEDCFYECIVQNVKH<br>HHIILKIVEEFLALANYAIAVPFFGGALLLGLAGMNLLSTDDLRIGPKIFCASVGATEATNMFLLCVYGEKFQH<br>EGEELFNSIICTRWYKRSMKCRKALMIMQCGSFRPPKITAARMIELNMATFSNLVNSAYSIFNLNSVASATEDK
AlucOR89	MAFGLEQMDCLTKEEIGIKAHFMRLMNLSGTFSRRKQTRLRSAIIWSVIYVPIILTLVATCIHFRKNFDLSSYAL<br>HHAALITIGFIVNVMTVCMYWKEFHDIMDGSTMSYNYDSGLVKNFAQQTIHERFKLSGLLVKLVSYGSVGVII<br>EVQIFFAIEAFYLQTYKTIFPMYVPMDLDDPFVFTSVVIWQELVVIYTTYLPLMLAVLYYNAWSHLDIEIKILTFA<br>VANIQRIVEEESQNFRHEGIHRETLEAALYETYSYHFAKHHAHITSYFELFSKCVKLITLLLFTMGPVCLVTVGL<br>SLLSDNIGIRLKLFWFLVIQLIMTYAICWIGQYIADVSTGISEVLVTAPWWLMPKSCRSTFLLIMTRCRKPLQMT<br>TDYGVPANMESFMDLLKGVYQIISVVIQMRDG
AlucOR88	MSFLMKFIDSLAENEDDALWDQLHKFYGPILEIAFIFPSWRRSKLPISLAVMSFYAFIFPVHIWLLTVGIQCVRD<br>DFNLASLEFHYWLIFMFSLVALLMMNGNRNFMISFHRTLTSDVGKYRAGRIYDEKRPLEWEHNKKKQLLKF<br>LSLPTLVLVLAGFSLLVPYLQKMDGTVEYNERGANMKLPIPAWYPFPTHEGILSLLAVLGQFMAAGGLATTVA<br>TLDIVVFRLTQSLLFEYQVLRYALETLMPRAKRLYTLKYPAEDMRKLRTNDEAFQRCIGKCLEDCVIHHQDIIK<br>LIKDYKTLVKWPGFMAYGFGTGVIGLSLVNMLSAKEQGRYEDIVLFFLLALAEVLNMFMLSTFGESITTESKEL<br>REQLYFIDWHLLNTSNRKLVLNFQIGVTHPVIIKVGGLVNVSLDTFSSIMNTSYSFFNLMNAQ
AlucOR87	MWKCWKKSSAPPPSLKDEAWKWPHESMMNWFGWWAEELERPLVVKLLQVMRAILIPSHLIFYGSLLYQTS

	NEFRQGTIISTVKSAFVSGPSTVACFKLYVIVRHRKSLKEITNSMDVMMKGILSRHIPEDLEKEMRSRWTGCRK<br>LYKCCVYFGCSVTTHASVTPLLQTIAGALLTDDPLPFDSWPYFLMGYYFWALNTFCIGHVLYMFDATWFAM<br>ADNLQIHFAVLKNYLENLDLTKRSDVDLNLCLKNHMELIRLCRIFRRISRTVIVTTRMCSMLLLCAGTFVLTS<br>AGDEFTSNDRGNLLSTLIYIAAVFFNYCRCADNIAHQLDELTTDCYSAQWVNAEKSQKTSILNMMTITRMEP<br>KFCGIASIDLDTFVNVMRGVYSYYNFLTAVDVGDESETSRTEVNEPL
AlucOR86	MWLREWFKNRSSKTASKEKQVVPVNIDGTPDFASFATCLSYQKFVGLYLDGSVINYLKISIPILFLTTGCISFAM<br>ADILNYKNKNIIWLIENGHWCIVYIAAIFWDTQMGIKSPLLLRMSRSVKSGVYKYAKYDTINREDLEKTNSQV<br>VTTSRFCVFVYVVAVLATLVKPTILEEYDPYRHFFNGWFPFEVNSLWRVSIVRVYELGCAWSAASGVCTFFVTF<br>MAYSYHIEAHLKLLIQKIEKVFDPESSEYDYIPQLDKKIRECLGHHREILRVFNDFSEFCDPTIGCATLMATFMV<br>CTLLYLMTNPDFDVSVIVTFSGVVAPEFSLLISFRLRGQRITDLSNKINEAIYKLKWLDQDVKVQKNVLMWLR<br>LTSKPLELKSFGYRNVSNSGIKEVLQTSYTFFNMLKAST
AlucOR85	MSKAKKSAVGTSSYKELEGLERAEALKKGYDENGGAYIRLGGQYVVSRSEIWRPVLFYADTALAMFELVTAS<br>YFSVLQGDMEAASECFHFIQMIFNMMVISANLQYYRKNIDELFTAIGAGFFDYGDTIDPQTKEKMDKHMMD<br>MRANKKFRFKVFVLVVWVLGGCMFIKVIVAHFRFGDTIDGEGGSVSRKHIVAIWLPGIDEWPSYIAMVVAAY<br>LCQVLIMNSIWGFVLPVICFAEELNAQLHIVGIGLRHTTARARHILYRKYGEQKSGNLKFKYEESLREALKSSV<br>QHHNVILEACKCASTLLNLPLMTVMFGTAVLLCMSGFVMVEDSVPIIANIISLLFIGGEVIYAYLFCYYGEMITA<br>TSLEIGDELYNDDWWEGRDVFRPYMAMISLRSNRPIKLSAGGFTDVNNAAFSNIISTSYSYFNLMFTSKS
AlucOR84	MYYSPWMANNCIMDTQEYKTARKKDYWYLFEMSGMILDWRPGFYIINLAYVVVMVINAVYTCACLIASIFK<br>VDDLIDVCQYLNFVGLLFVSLSVLASLNFQRERTLETCAIGSTEFFDYGVSFSRSEEIEQYRKEGRRRMKILFMV<br>IPPWLTIIALSLMMSGPIDAAFSYPKINATYVNGIYQMAPLKMYYLHPIDNEFIRWLTVLSQAACSGNTALVIG<br>CADLIMFNAGQNVIIQLEILNLAVLDTDKRASKLYELKFGREPPSDPVDKTNDRPLMNIYGFCLKQIVDHHKI<br>ILRRAEVYHKIVNWPCGIVVVNGSIVVAMSLLSIMQGGGKPSVLVLSFFLIIAEVASIFMVCEIGQSITSQCERLF<br>DSMYAFKWMDCSVEVNRAINIMKCRFRKPIIMTAGSLTPINRDTFGTMMNTAYSYINLVAASGKEDAD
AlucOR83	MMGEWWKDLKLPAGRHPESAPTLKKIYDDYIRRFDHLKMFKPIFCDTQFKWHTLLAYFGLFLHNTFIGFSYL<br>VTCILNMDDISQASFPANLVIIHLIVLAILYQLVRLSWTENVTIIDNLVTIVKGREEYCKYTGTQYDVYLINRRSK<br>ILRRVTQVFTYVFGFQISWLILPVINSVFGEKRVPNEVSNGVAVTLGVPIWTPLDADHSWFYYCIVCSMQLFFF<br>GSSGLFIGLGVFYNMMSQMSILDEMKLLIRSVGELDSRTSRLLTDKYPKLSIDKYSEKYDKCYFQCLRDNIIHH<br>SFIQRTFSEYQSLVSVTLAIPFFFSSIIIALFFADITSGSLTLVELSIEVIHMCMNIFLMAMMCYFGQLITLKNEE
AlucOR81	MGSFYQGITSPSENALKKLQISADTYVENGFFIARFSGMYRWSLLYSISYFSCMTFGIVAAVGYILNVSTTDEWD<br>KFLENIHITLLIVNMEAQGVAYHYDQNGYIEIWRAIDKGFFDYEGTLDEETDEEIAIMKSELRNFKKVFQHNY<br>TMLMCITTVLQFSKKPITRYLIGGGTVDGKNNLIWEAPFGLYFPFADYWIPYLLGLFLGNACGLLILITALGSV<br>LQYIYMSEALIQEFAVVKKTMSKCIERAEQIYRNRSNSNQVEHQQWTMDDCIIHCINQSVKHHQITLRMMN<br>VFKKLMYFSLFAIIFDGGLILCISSYILINDEVGITFRLPMPCVIAVEASLALVFCYYGGKLTDANTDVGNGIYEC<br>KRWMDHSKILCPYALIVKSYCNVPNELSAAGFTNVDVRTWGNLLSTAYSYIGFLLST
AlucOR80	MVVEVNSRTYYGIEVVYFKFIGFWQFLTNGLGKDKLVISSIVYGLMFTFFIIVQILDMFIKDYDFSIFSEKLSVNL<br>TCFESVIKIGYYCFKRSSLLELLPLYRLDLLLSAKHSPVISTEILMANRRFVNGATKSFVVMIFSTVGIWNCLPLL<br>KCFTSGGCSTLQIMPTWYPGDVSYVPLNLFVYIFEFFIMIYCAALLYNVNCFFSSLALTASAQFELLSNNFANIE<br>SNAERRIEDHASSTDEDTKKATMYVLLRECLIDHQTLLGILQKMEDVFNPMFLFQMLTSTFTICLVLFQLNFH<br>TASGDDLPIAMACKFVMYLVFGSMELLVYSWGGQIIYNKSEEIYWSLQKCGWEVGCDKFKTNVQIALQRSQF<br>PVTLTAGKFYVVNLASFSQVIKASYSYFTFLHGSISNEE
AlucOR79	MTVVQTNKRTAMTDVHHYHSLLLTMLEIAAVFKKREGSIFSPTGFKMFRVANLIVCFLFVTSCARYVFHEKG<br>AQFFTVAIGTGSIEFCIINMILVSKSDIIDRMLATSAKIFYQLPQNEETRDVLETYRTKGYTFMRAFGMLIGVNE<br>VLGLIKPFWMARLTGQLGLPFDISCLGIPTVPCWIFQIICTSHLIVTVAFHVIIVKTLMYLTWGHSIVITKIMNRR<br>PVHDDEENDRKIIELYCDFSRFSTSFSSLFGLTTFIEVTFTSTRCCFLIYHAIKSLSNNDMEQAIVSVTALIASIAIS

	YVMCSCGEDLVEINQMMRDGFYNSKWYESSPQSRRRMLPMLVLSRVPIRFQYRYYMYFNYEILMKIMHSTYS<br>LSAALIQFL
AlucOR78	MSQRPDPEGPPSKRTYLSKGLKLKSSVRLDAWRHIGYRLLLLGMTPENFMEKFSVRHALVYLIFFLTHSLYSA<br>HELTVALFFSRSLLERVTHGYILTYIFTFNLQWYYMLRHVGNFHRHEMSLENFASTQAHLDFAEEVFNKNIN<br>AFLKYLFISMLWWATNTTTHVFGPLVETVLMYVRSGDFKLATVLPQVFSLPMWAQVIMYIHNATATILLFVYC<br>LASYLVLGTRVLKVKTQCDILNEALRRDYDEESNIRSYIKDHINIIKSAKLLNGHMQMLNGIIFTACYLEFATQ<br>MFALTLFEPSGAYFFALAFDLMSIMLILVMQCWFASIVTISLQSVTDAVYETNWYRRDKDNSLNVLMMLQMA<br>QQDYIQRIWFKSFKIERAAILNLIRSSYAVYTALLIFQE
AlucOR75	MDILHYSDQTGFKFIPVYDHMLRSIGVHSERGETQATAIKRYFGNFLILVAIVQGWSSAVAAYDSLKEEDFRAV<br>TNVMSYLSVQLSSFSKFHIARTHMEVTHRLGNWIVEAKKNRPRDMKQPQLEFLVLKVNPAFFYFGLFATFFW<br>CSVPLTNNLQAFIPTQYPFLDKQTSNSISFPLIQVPLYIFFTVTITYTATSLLHFLAIFTTEVKLLSKKWEQVFYDK<br>RRPDNYMELMKDCIQQHIKLLDVMKDLNIIHDSMFAFQVFIFIVHFVSFNFCLVMTSGSNALSSVGPLTMSSL<br>MEFGLLCWMGEEITDALQQFHRSLYMTNWYEASLEDKKNMIVVLEVLKKRHALTGTKVFVASLETFVEAAR<br>QAFSAYTLMKGLTTTE
AlucOR73	MNTEVTERFEYASKAYWGALGFTGLDAFLYEKPPKHNVLRWYAFRIIYIYFVFVHYPIFITMQFWGIVTAESHT<br>LMQISFDISLMGYNIQNIIKLLIWMIRIKTVRSLRLNFSKFNVNKYRPKLSSWIIKRDAENALKFTGRCYWISYA<br>NLLFWVVLPTATAIINYSTYLAGYADWQENDFPRYSNTRFPFDLSQHRSQVIVSFLEVILFTLGFMSFQSMDMF<br>FSVIIRMAQAQFSVLNSALFALDGELDKFWGTEVPVNDCKPPMKIHLIVQDHQRMIRYGVRLRKFLSPILGLE<br>TLNCITIICNMTIVASSQVSGGGEFLEVALAAFASSLVVITCLVVFFTFTSMTGQLKDAEESVFYAMYSSKWYER<br>DVSHRKSIILMQKQAMTSRRIKMFGLGDMGRSTFIDGLRMVYTYYNFMQRFK
AlucOR71	MAPQVNLFKAWMIWMKIAGADPPSVNFPYALALLWKLIMLYGSVHYVIIMFLAIVVGDSAFHLKLEAGLFL<br>LAGIPCSYKHFVFVIRKNKLHEVIDRLNTLLEEVEDVYGTETLAGWQRICNMVMYFYSTQFTMLVVPVFSFFY<br>YIYYWEGVEATPYEVYIPFEKENHIHRVMLYELLSFLGPAAGLITGNIFFGSLTVAVSGVLRKIQEQFSQVSPSN<br>AQFLLHRTIRWHSEIISIVGETNRLLGTVFVVEYLLAMVYICFSGYMLLKVGSATEDVNLNKNIILCIVCIVMPL<br>FYCLCGHVIVLEYDKMSDSIFQNDWVSLQPVDRKKLILPALLAKRGLSLHYKKLLKFDMTTYLKIVKQSYSFL<br>TMLKLMENT
AlucOR70	MARTDLSDVIQLLQFTGHYFSFKGSRREKTYETLQKLRVVFMVICNPFTLSSLFIGGLKKSMGVELFFGLMGFL<br>TAMQHVYAYRHRKTTEDIIRSILEIRRKYQQGSDIEFQQNTRAIWKVVYIYFSAMTSLLVFYITIPKFVDILYGIL<br>WDDPVALRLPQSMDAYLDEHQHRNLKYATVALVSSSWSFVSTYSHFGLDTLLSLVGFYYSSLVKTFCNRLKLN<br>THLTSKELEGHIKILAAHHHELFKLSLKMRSIFGCPYAMQNNFGAFCIVSLVYALLSDDSSGLLIKVANLFNLM<br>ILAGMLTSTSYIGQHVTNEISAIFDALYDLPWYELSPSNRKYLVTMICVARDPFTIHFHGRAPLNLANFMAILN<br>TSYSYFMFMRSTL
AlucOR69	MGSVTAKMDKWEKDEQEEVMKLFKEKYGPLIQLALIFPSWKSSSRSSTIMIFVLHSVVLLFHWMMIMISIKRS<br>LESEWNFEMLTIWIHFAFIVFFVFIVIFCVNNQRSTYFRQYQIMSNDIGHHRGADGSIYETDGCVSEAKNIKRE<br>MLLYMIIPVLIFLFASTIYGLPYISKWLEGMENPYTLAMVNMNLPVPAWYPFPTHAGLGHFTAMAGQALVAL<br>SVGVVLITIILLFLTNALRIKFEFRVICYALQTLFTRSTTLFLQMQHDMKDIGNSEHSYQRVIGSCLVDIVVHHR<br>AVSELISIFEKQVFFTCALGYMVGTLGVGLSLVNILEAMKVGNYVSVLIFSMMASMETLLMFTISQIGETITEES<br>VKLRHQVYDIEWHKLDTQNRKILLIFQTAITEPIIVKAGGVINMCLDTFSNIMNLSYSFFNLMTNTN
AlucOR68	MSDRHPDIAKYIKLMQATRNWYFAEDSTSHPAVDLLKRCYYHVRPSLFVFALLANGYGLYYREGFGALDGN<br>LALFPQALASLVTSSTIYFNRRHHRKLTMLLNQRFLDKNEPWMVEIKNKYVSAVWKFIKAVILYQEFVKIFYV<br>LAPVIVDSILHHIFDYLETPFFFPLTFSTFLTDDDKWTGRYYAVMFLNIWSGFEIVANLQGFIICYTVMTVFSVVE<br>LVILTEQIKSLDFYRSNGEINEQIRMVVKSHNDNIALNRELKAFLGPACAFLSLFTSLVLTLIVFTTTVTNDLMVI<br>LAYAVGAYFYFVAGLLYCSLGQLLDNQSSEVFDELCNLPWYRSSPDVRKSLNMMIRQAHNSLIIDYHGHYM<br>MNLANFMNIMKSAYSYFTILQSVTGSD

AlucOR67	MPVSQSLWNMIEGKMEITEEFEQKVTETPVERTAEDVFSTQIKALQIVAMWPNFKQNSNMELFTRALLKINTFVLAYCTLALFVKGLLTQDLVDRSEAMDIFTLTTSALYKMIFFYTHHKEMDDMVNWGAALAHQVPPKWMQYTTFFSCFHNFMGIFSITFWGLCPIFKWIFGETDLDGMTLPINVYDPIGVTGAMYSVFYIVCDYGLLSAVQIYMASDAYLFTAIHLAIGGFETLNNKLRKMGQINFNKGPTVNDSMNEYLKDCVKLHTHILIYIRKIDRLFRSMIMADVLHAIISLSFAMLQASESKGIFENMKMAMFVSYCIVHQYLNNYFGQNLIDQQEILNKELLISVPWNDGSKEMKKSYQIMMAGCLKSVRLSAWSVYTLQYATFLEFVKSMISYSMVLRQVQDQTVKQP
AlucOR65	MSFIVGYFEKILFHFDKLEEPRSEEITKLHRKHFSILLLVSSVDLNLGRKYLFWTLLHAVFNYVVLAAQTCVLMYSTFLLRNDFEIGSGVLNYGLLMIVAIGILMNMQYFRHEVLHISGIMCTGLFRYSDKTMETEDMIKFRKHMKFQRQLLIALAVYVATIGGIVVLGPIIDEKLGMGFDGTFDENGVNRRLPVPLNYPGIDTSKIFGFLLALGMIFQSGVETTLIYGGATLLFATACQFILTEMKTLSVSIQTIPHRAAKKYCRIHNVSKKSLDLKTIFDDSEFQDCITDCLKENIQHYLEIYKFTKVLETYVKVPLLLAVLVITLAIGLTMMKLNEDIVRIGATISFTSVALGELCIMFLIAVYGEYYLTMSQEVNWEIYFTPWYKFSVKNQKLIRQFLISTRNELCIFAWIVRMDMEMFASVMNSAYSFFNFLNISKTLEEDELN
AlucOR63	MAEDDYTLTDMAGVYLLPHSRAYMFWTGHWVGAVPGPTPVPIMVARALGGTLTYVAVFVVCWGSLNGIMHGSGTNDMPMNLIIISCSISSVHKYCVYTNQEQGLGRLGRWMKRVSNREKKNKIPKTITDHILKVCLLIFYYSGTLAAFMLLTKLALTGTTYNVLVPGLENVLLLKLAIALSFMSLGFEVVVDALILMNSLFTFRRELMRSFEEWRKLNFDSENPNQYKEELKERVQKHIELLTIFQDLREFNNSMFGYQVFAIVFTTCALLYGMAKETENSHKVFVQTMPTATASFLEFFILCWCGEDIKFGFEQIHRSIYDTNWYEASLEDKKSMTIVLEFSKNPIILTGFTVFKANLETFVESMRQSFSLYTILSEMV
AlucOR61	MDTFCGGFMGKVYYLKKHNIYINVYLSCRVNRDNFSSFYRDFRYFLSQFYHLMAALSAALLGGSVCIVFIAIAEHLLAQLEILCISFRNAIGFIPAPGDRVGEKLAYQRVKSCLQHHNIILKFFDEFQKYYSIPLFCMLAGTTVAMCTIAFVVTDPSSTFGVSAAFLSLMAPEVAFCICYCTYGQKITDMSDILRDTVYNAPWYYQPKPVKMALLMALNKTRTPMTLSAAGLKDCSIKSIGEITQTTYTYFNALQLFRGKPAYHRE
AlucOR60	MNDIGGLALAKSGLNNMMSILGGFRGPREVRFKGTIYEHIFIAYSYFGLLVSHYHVICCYLTPIFMPDMSFKDAMFFAVPCITTTFSHLRIYYMAWNRSKFIQLLEMNEEASKDDYYEDELQKEIDGWAKQVRILQPILYFAVSAPIVPWGVTPIVNEVLGNPWGPRKAPIISWYPYNVQETHFWVFTIFIQTMAGCHATLSNVMFDAVFICISTRQLALLIHLKNSFSKIFQVIHVDPKGISWYTNYRAEAVEKEEIENDLTQRLKYGIRKHQTTLRLSKTIVFFLATRFWIICLIYELHMYLFFMEVVQVRKS
AlucOR59	MFVFIREKELKRDPNAMIGRRLVQARFAIFAGIYPDFYGWRHYYFVIFLWIIHPGLYSYFVMVYVLSFVEGLRYMDVELLGQVLCLGTITVIYCLVSIYYIAKKTDVDELMMMAGKGLSNYSRPTTQEEKTILDSKEKSTYKYAIGSSIMFVSVSLLHMGFLPIRRGLKGQYTSITNDTAPINKYTPLPVWTPYVCDDLTTFLISYFTQLIPGCMEISIINACCILYIGLCQQLTGNLEILVNSLRQLPERGLHMFEAERGIVEKFTPELYQNEYFLRCLNTCIGENIEHQYNIIKFYKKIQSVVGFSILAIFSGTGLIISTAAYSMLLIAERERDTEIIITNSFVWSFNLFVYTVLLTLYCYYGQKVTDKNEEVLEALYDTPWIEADMAFRKSVIIAMSYSQLDMTLSAMGLISASLATLLDIIKTSFSYLNMLLAAR
AlucOR58	MSFLLKFIDELAEEEDDELIEVLKNEYGHFLFLAMIFPRWKKPLASFGLVLFYLSTIVLHHAMLSYAVYLSLLEHNWEQVSFLTHLVILLSFAIFQPVNFNWVRRVVAHVHRTLAKDVGIYCSGRIYDDPVCIAFREKIRLEKKFYMTMSAFCLMMGGILWSGLYISKSFSDIEQSYSSSGLSLKLPLALYYPFPTDRGVLHYVILGSQLLVCLVIGFLYLICEVLLINLFLKIKYELQVIGYAIDSLVSRSINAVGVNENNPQKDVLVIEDTKLQRSVEKCLKETIVHYQKILGLLLIARANLDAPLAIVMMLGLLVIGISLLNMLAALKANYIGMFLTFGMLVCAEIQAQLLVCLLGSSITEQADILREKLYSIEWYCFDMKNRRILLNFQACFTKSFVVTAGGIAEINMVTFSWILRAAYRFFNLMRSTS
AlucOR57	MTPEKFMGTFSVQHAILFSALFIFHTMYAIYEVTITAFFARSLLETVSHFYIATYLFAFNFQWYFMLYSIRTFHENEIFLEDFKCTQTHADFATRELDENVYIFTRVLFVSCLCWTVNSSTHIIGPAIEALISLIKTGQIDNVLFILPPVFSTPWWAQIIIYFCNAITMFGLLLYCLASYTIMGFKVLKLKTLCDILNEALRNDVEEESNIKAYIKDHQIIIKAAKLLNAQLRTLNGFMFTACYLEFAVQLFALTLIDPSGSYYFALALDLSSIFLILVFQCWMGTMITHSLETVANGVYESL

	WYSRGNNNRSEVILMTQMAQKPFVQTIWLGTLKVERATSLSLVRSSYAMYTLLNFFQDK
AlucOR56	MSRYGKIEDDELVNSIDIWYLKRSGLWEVFNHYREHGVRNRRFTLWKIITLILFVPIGFFSLCGPFFTETDLEGM<br>TLVILNPMTSSQTVIKFAILWYGIETQCRVLELFKRDFLTCVPPSMQAKASEILTKAAKKANKLANLGILTDVIT<br>VSFWNILPLLRSEYFRIELGITAFGTPLRHNKILGFWYPVDYDETPYVQFVYCYEFLSCVWAGFVIALLEGLVIH<br>LVILLTANIKVMHHLLEELKTSNGTLNSETLLTYIKDHQKLVKISNDMRNLYNMMITMELSTGLIILIITIFNFFL<br>SSGNGDLVIMFKFMVYLMYTLVEVTVYCYIGSDLETTSEDLGFAAYSSQWYKVGKKFRKTLQMLMVRTRYSL<br>ALKFGRMYPINLMALTNILQTAYSTSMLLYRATSQDEQKEEAQILM
AlucOR55	MLHEWTLFQKTGSSNSSDKEHYDIKETKHFATCRKVLVMMGFVNDGSVIYKLRQPVFVFLLYSAPVHHLVPA<br>FIDKTAPSDQILMSWSISMLYVLLCIAWPFMIIRSSEIFQLWATVRKGFYHYSDPFTAAERTILSRTNDIVIKSTRV<br>SIIAYFCAGFGTFLKEMQPESLRQYKAPYPGWFPWTINSNFRFALALLYQCAICLNTTFALEAVFVLFAYHTIHF<br>EGQLRLLTRHFGDTFPPGLPATVTYSAEYKKRTLRRLYECVSHHLIITGFHKQILSYFGICLLVYRVIVTIMLCILC<br>YLTTTGISLDKFVQLLCFALALLYLCFIFCLKGEKVTQLSDGWRLTVYEVDWWNHPVEVQKTILMMQMGAS<br>KALKVYGVWKPAMYSHEGISVIGQETFSFFNMLRAM
AlucOR54	MFEPSEEILRELKISRETYLENDFFLARLSGLCRWNRLFSLFYFLSMTYAVASAVGFVLAAFSEKDRDQVLENIH<br>FVPLIFNMASQAASYHYTQKEYLQLFRAVDSGFFNYDGDLDIATELEITEVKSVAKNRKKKFGHFYSMLMLV<br>AGLGQILKKPLLYVLRGGGTKPVDGENNLVWEAPYGMYVPYADHWISYLTGMFLGYSACTFVSITAVGSVLS<br>YQYMSEELLAEFKVVEITFSKCLRRAQTMYENRKNVLKANGKTSQITMKDCIIHCMNLSVKHHQHTLRMM<br>NVFKDLMFFPLFMVIFDGALVLCISAYLTISDDVSLNLRITMPSVITAEATLAFIFCYYGEKLTEATEDVGDSIYN<br>SDGWVQHSDIIRPYALIVKSFCNIPNELSAAGFSSVNHNTFGNVRT
AlucOR53	MSKFNEYWDNWWFGDPPMNDKVYDAIYEEFNIILYVSGLFPDPRPIRRLITLGILIFNVVNCAAYVYFLGVTA<br>TIQINDFVTASQTVHFASVDLVAVTCMISIVASRRHMIDMFRTIANKYFDYGDDFEIPEMVEEYRTMKRQKIIIL<br>VVLPSYLALNAFVCMMGRTIDGYFGRASNETYENGVYMLTPEPMWYPFTIHNELMHWMIVMSQATGAFAL<br>ASAVSGSAAIMVLLCQSINLQFKIIIYRIRKAEECAYHLHRKNGGEKLKKSELYSDPSFMGYFNSNLNKLAEHH<br>SILIRQFDSLYQVVKWPAGCALLLGSLLIAMSLLALLSGDGKPSILLLAALLIVAEVMNMALLCGQSESVQELG<br>QSLHEELYNMNWIELNPAAKKTMMMMILQSKRPLVLMAGGLQPLNWEAFSGIMNTAYSYVNLLLAADV
AlucOR52	MGFSSWIAQQVVTEDEFQIRLKKYGWLHYLFQFSLVNSCYRSMTSLLMYYISLMFSLSIVIFHLFCYIKTALNAY<br>SFGRADMSVANVHSVVLGIFIISVLTSYAIDKTKTSAIEQLYLESFLSYENEYPAPVTLFQKLMTLAGKMGIASG<br>GMGLFFNVYVAAPMIDFRFWKESCVVKGINFCLALPHYYPYNSEDGWSFYLTELFQLMFGVYRISVFCAVQV<br>TLTVWPLHLIQELTRLKTSIEHLEERIKKRYHRITMRNVEKVNLITMCMDKTFNECAKFCINENISHHHNILK<br>YHERIDGIMALPSFLAYTTGTLTMGIAMVKLLSVEGDTTLGGNLAYVTVLAAEIGFMLLISTMGEAVTIQAEEI<br>FNEVSHIPIENYDLEFRRNVIIFMEGTIKPIALSSSKFNKCNMEAFGNVLNAAYSFYNVTSASAKLEK
AlucOR51	MSVQGEVIASKAFKDNFGRYMVWSGMYSESKIYSGAIHVFFLVHTVFLAYTVILSLDDEKLMGESAHFTAFRV<br>SAFMLMINAVLNKDNLEILFIKLGQNQVHDYQNTLSDQCKKEIQAVRKNCDERKDFYGTNFLRTVSAALVIF<br>WVRSLMEYYRGHMDNPKSDNGVNKNLPVPTYLPYESHDWPGYQFALLSEVALVMMSYFLVLGHDCSFICFS<br>EEVLRELEIIIITLREVEQRSDHLRKEMLYKISQKESVHICLKHSVIHHQKLIKIFGSFKRYCFYSLFFMLSGGAFLI<br>CLSSLMFTSEKISHQDKSVFMMFLANELFHIFIFCYYGEHIMDRSIEVGNSLYNSSWIRIAQYVKPAFIMVKLRC<br>QVPLSLSAGGFITAGFDTYGNVLRTAYSYLNLLQATN
AlucOR50	MGEKPYTMDKNGEMEIDWLTQEDKNHINFFDQFHCWSGMWRSKKSIQWTYFWLSQMIAFSLVYFYSFYFL<br>LSELELLAHLIHHMMVAADDIMYIYLLNIHRIRVETIHDYASKTYNYDSGIVREKHKKLMTEQLKLYPKMSKF<br>IFLTTVATAMSLEVNYLLEATYLKSYVTMYPMYLPIDLNHPVTYTIVVFLQHLQVFISMILCGGLMSILFVVWS<br>HLKVELDTLTFAITHVDELVEEKLRHFSYTNPADKEKAKAEFYNGFCYHFARHHAAIKRYFGAFQISCKVTM<br>TFVLISGLICFACVGITSVTENIGIKLKFFVVMVIQTLIIYSWSWVGQDISDKNAALQNIIGGTHWWKMPKTCH<br>STLKLMLVGTSRPMLLYTLIGQPNNIDSFMDMTASSYKIFNMVYQVKFSS
ALucOR49	MIRNWQEEEEGTTLLERMGQKFLNGHSIYLGGWVLRFPVRLPPFFYLTCAFGLIVKMILNYDNLTLIIDCAH

	MMIHMIVGLQTTLLALKQKGNIMRLKTQLDNFGYVKDLETAGEKIKEECEQEAMELYKPFSRCILITINVYILF<br>PICKLFTEAGRAKLSRVLVWQMWMWVPDDTWWGFTIIFLFELVTSLFLLLSVMYTVPYLACLGKMTVGHCK<br>LLALQLESISKKATQAAFTSGSFKAALNHEIDECARRLHETHMLANEVAEVYKYYLSSFYYGGMFALCMSGL<br>QAVSATENIEESLKFMGVLTGELIAIGLATYVSEGMIQAFADVRSSI
AlucOR48	MSESVAVFKSLNLALKAVGFVDSASGIKSYLWTTWNFIIIFIGVEFIAITIPLNIIQDNDIYLKLECVLCIYLNLSM<br>MFRFTVLTIRKKRLFSLINRIESLLQQQMEREFDKAFITALARRCGNWAYLFTFAMTATCIEPVVMAYVKYYFR<br>GVEDPVPFEVNLPFEKKNNIHAVVWYEVFQFLGCSMIVISTNILFSTLSETTSELVKKIAENFEKIDETNADYLL<br>KQTIKWHTEVIGITKETNDILGITIFADAFFALQYISIAGFLLIRVGLSNTTSFSKYFITYICVLTNPIYYCYSGHRV<br>SLMGDVLYDSIYNNKWYRLAPKTSKNLILPLMVARRGLSWNYKALNFDMALYLEIVKQSYSLITFLKMMK
AlucOR47	MSMSVWALTIGGLRSSLGIEMTYACLTIMTTVQHFHMYRNRTRTEAIIATFQEIRNTYQKGTDIEFKNYTRFM<br>WKVVKVYIVMIVGICSAMSLPFFADLVVWFVWETPTAFRIPIGMDSMVDKEPVRDATYFAVVLFSNCWTILG<br>GVTQMGVDTFLFVSCYSLSSMVKTFCKQLKVPPNSTPEETTVHIRLLAAHQQALYKLQTEIRRTFGFPFFVQN<br>LLGSFCICSLLYVMSENGATLLSQFIYVFNMIAVLMILASTAHVAQHVKNTTSEVFEALYEMNWYTLRPSDRK<br>YLVTMLGVARNPLCIHFYGLLPLDMENFMSMLNTSYTYFMFLKSIG
AlucOR45	MLIHSDIDKYIKFMKGYCVWYGKSVTWDDTRFDLCRKYYTESKCYLSAVLLCVVSFALYSTDDFGLQDGTFIY<br>WPICLMMLVLTSIATATRHQQDVLTMSLNDNFLENTESWMRAIKDQNINRLWKMLRFYTVYNNTISALYML<br>VPLVVDSILHYGFDYLQTPFTLALPLTPLLKYSNTWNAQYYVLTAFNFWSCAEMVFMLEWFLGNYLLLTTFFL<br>TELIILKHQVKSLDFGKNEEWDQQVESIVNKHSKIMKLNVELRDYLGLPGAFISFFSSILLTFTAFVSFTSTSIPLR<br>VSYGSGFVLYFGAALLLTTIGQKLENESDELFKAFYSLRWYQYSPNARKSLNMMMRQARTPLIIDFHGRYKM<br>NLANFMQILRSSYSYFTLLREVAKE
AlucOR43	MRGTMELDDEEVMQTLQESGLRTWIGVIAGFRFAQQPRFKGTLKGRIYWWYEILTDLSVAINGISQFAALLTP<br>EFRMIDRCLMCFPAASCLLCLFMSNYPRFKRKNFRSLVEEYENSFSDSQYRHHLEEQIRKGAKHTRSVIMCLV<br>LLEFISMFIFCLILPVLNEATGFAFGPRRLAVPSLWLWDPLAGFWNYMAVVFVQLCGSVFVSLKKIGFLESFFVY<br>ASRQICMFTHLRYNLGKITDPLIVNDDGKVDVKEFTGSNRYLMKRKLIGWVKNHQNCLRLFEDLVKLYEWP<br>LLVYFGATILILCTATYVTSDNSIDAQTCVICGVFNLGIFFELLFICRTGDRIKHESEKLLGALNGKNTFLLKSDE<br>YKYLKMILTRCQSESVINASGGFPLTITTFIAIIKSSYSYYTLLKKVNGQTD
AlucOR42	MRMTENGKNSDPDSLEIAEEKATKYLYQRFFVLPVVGGVFGFHQTRWWSIFTKTVFTAYYVSVLSTIVTLSYSS<br>YLNRDNMAVASGCIHILITAFVVLGISLTLQRLRKDVVQLLSLDEIICEYQCSEYLSNLIRKSDKKLRFLLIFWVA<br>LYGSSAWIAVIFPFIDVYLGYELSLSNVTDVYWKGLPFASWWPMDADNSNLAWTTCFMSQGLYAFFAASSAT<br>SGMLCFAIFSEDIFNHIKLLVNSIERLEKRAKLMFKMLHPGKSLRNSLDEYDECYYNCILQNVKHHQKIVIKK<br>DLLMKIANIPVAVPFFGGAMLLGLAGINLLSDGDIRIAPKVLFTCLGVTEAAQMFLLCHYGEQFRTQSELLFN<br>ATFYTKCYRRSMKCKRAMMIFRLGVSRPMAISAAKLIVLNMGTFANLVNSAYSIFNLQSITTQED
AlucOR41	MGCLQAKIEEWSDAEDEEMMNSIRRRFGLFCQLSLAYPSWKPGMRRWTLLLFFIHTVLLTTHSVLLGISGVL<br>MVLEWNMELASLTIHFSVILFFAIFIVYWMNSQRPLYTRQNMLMVTDVGSYKSGRIYDDDFCVEERRKNKRE<br>LLYYITCPVFISLTAGYVLTVPYIQHWLYSSGESPYTANMVNKHLPLPCWYPFPTHEGVLHLMVLLLQLAAAL<br>CGAIVLVAILLLLIFNTQRIRYEMRVVGYSLTSIFHRAKKMFLEQNPHRKGDNLRDDPGYQKVIGICLKDTIIH<br>HHAVSEILSLFTKQADMPAALAYTIGTGVIAMCLFNILMALRDENYTSVVLFSMLVFVETLVMFVMSLCGESI<br>TSESVNLRHELYFTKWYNLDIENRKTLLNIQTNLVEPVIVSALGLIELNMN
AlucOR40	MIPFVFKRDDSVDHEVVKGYQSTYNYIMRFCGLYPDFRGFWYYISGAHLNTVHLAYIWFLAAYMISTYYAFAY<br>RDMELLSYELCYGLVTLIWFTVTHYTIYKRDQLDSLFRKVGRGFFTYEKPIDSEEEAIIDECNTNCRKTFQKTLA<br>LTTILAFWTCIIPPLPKAVMGDYSSIVEGGVPVNKHLALPTWNPYPTDTHLTYWTMWMYQALAGCTEAYIIG<br>ATCILYCNFCTIINRELKLLRFSLGNIKNRAIHAFKMRGYSLQLGQKYENSQLYQVCLVHCIDESIKHHIELKQF<br>HGAIQNLLGFPIFAIFSGSALTISSPMFMFLQMIGEHEESSFTLVMNIFQYTIIIFGFTYFLANYCLFGQSITDESAL<br>LHFAFYDTPWPEAGLNFRRKVLMGMIHSRKPFVLTAHGLASASSETLVDMLKTVYSYFNLLAAT

AlucOR37	MTPKVGKSEYVIKDTKVFATCRIPLVSMGFVDDGTVMSKIRKLITVFLMYSAPVHHILPAFVDETINLDGVLIG<br>VSLLMLYILICVSWPVMFRRSNDIIKLWKTVQKGFYQYSDPLTKEERTLLSETDDLVIKTTRISIIAYFCAGFGTFL<br>KEITPSSLRAYKTPYPGWFPWTINSNFRFSMALLYQLALCLNTTFALEAIFVLFAYHVIHFECQLRLLSQHFKDT<br>FPTGLSATVTYGAGYRKNTLRRLNECVRHHLVIKRFHEQILSYFGICLLVYRVIVTIMLCVLCYLVTTGISANKF<br>LQLLGLALALLFLCFIFCLKGEKVTLMSDQWRQTIYEVDWWNHPVGVQRTVLMMQLGATKPLRIYGVWKP<br>AMYSHEGISVIGQETFSFFNMLRAMK
AlucOR36	MLTEYNLRFLKVLHYYGFWITFVGWKHDKIKKICLPVRGVVIGLSSILAIYKLGQEGIQCIINGTVVYFPFMVY<br>TFTIPILLFKNRKHFLSLLLDFEDCWKFFCEEKERKVLERHYKRIWKVANYVHICFFFEIAFYAVMQLALDSVL<br>HYVFDYLSKPHVLTYPHMGYLPTNRTWDGLYYVVAVVGCYNLVETLSVQLGWVILFVVIVAYCYPILLLTEKA<br>FKNLLQYDGDPDSAALKKAVQSHQLLVKLNKDLKAFLGLPCAFETLFVSIILTLTAFTSVTSTDALVVGAYSSG<br>FILHFLAALLYFSLGQLLENKSEELFTILYDLRWYTFSPAVRKDLNMMIRQARKPFVIDFHGNYKFNLENFMQI<br>LSTSYSYFTIIQTLTDNH
AlucOR35	MGLLFGRTPFYIKSGWLRFSYKSLPFVYALILAFANWFCLLYYIDHLHNTWNQTLGSNVSFSGVLFAILVFSQP<br>FSTIFMVYSWAFEVPAIVRGYNSTAVLEEKISIVFPLYKQKPSKRNLVTFLIAFLFVVDLIIAYFLLKTRFTETPLILI<br>LLIIVNVVVTFSYCVLWCFNCYFISDLAVKLNKYMLQCLQVRENCAFKIKTCRKIWISVWKQSQMNSQSIAVS<br>LSFALVLYGMIFVVGCYGILTSIRNQNILETLEMSPYVVVTFTIIACVFETSYQASHKLGATFLDTMIILDKDRVD<br>HECVEEIDKFVDTINRTRIAAITLKDYMTMDRTLVVSFLSYSITYLIVLIQFQDKNEEPSVNISTPMRNNTL
AlucOR34	MVTVAVLTLTYVFTHEVVTQTDFIIAINSYIVSMTFCAAAFKILIFLVLQKEFKKLFLMVEELGDLDMYAPSTKD<br>HFFNCYMYVTLVLTNPCTWSLWHLIAHNDIPFKSQYPWGDDGVGYLLSFFFGIMAAVFCGLSHILVDTSFMM<br>VIAGITLHVDKLSQSLSLLGKHRFKDSKIMSAGIDKHAQLLRVSQHLSTCYSNLFVGQSVYTVGHSCVLLFGA<br>VHVESKVEVVMSLGTMLVTSYCQLLVYCYYGELLTSKFSDLVFDSYNNAWYDSDLQVKRALPKFSLMCHRH<br>VSLRGFGKVIPSKSNWLHSLQESVSYFLFLKTISGEE
AlucOR32	MSGDISRFKAKSTTTNESLMREEYIRKGIDENNGLFLIIGGMYTGYLPISILHAILTAVHIPLLLAAVIIGRNDYV<br>VVSETIHFIILLSLAFVISMRYLSVRKKLDNIFEAMGRGYYNYEGTLDPGTEKEFAIHLKESEKRKSVLKYVFVG<br>GCIGALICVSILRPVLQYYLKKYIKSKKLPHGLNGAKNTFIYYPWDSSNTWLNFIGYFLQDAYTLMTANVVFG<br>FVLMFVSTAESVVVQLDKLKLSLKRIKIRAAFIASINHEDPATNNNKDFRRALHICIKHSIKHHQLISRIFDDFK<br>SINYLLLFYLIGSLTFLLCMSAVLFAADDVSLISKATFVFFITSELVATFLVCMYGEHIAGMSSSLPMDLYNTEWY<br>HFSNELLIYYRMLAMRCTRPCQLTAGGFSQINRNTFLEVLKTAFSYANLLQASKQK
AlucOR31	MNGNGAVLNGVTESSMPIKRPRKLKKVPNKRSIQFETDRNEKKVEKFLSDEEALKKGFDENQGIYLVLGTLY<br>RTSLWSWVHTVLFSLTALFMMVCLGRVAVLISDDFSLLFETIHYITIIGGVLVILPPMMRDEFRFEKIFKTFGRNV<br>YSYDMLDEETAQSVQKLRAQGNREKQLLTKAFTVMLLGTFAGFSVLLPGMYIINGKFFERQRDDGIIMGIPCV<br>LWFPARVGDDWIIFVRVFLLLIEEYAAFTVVAFIIGQQTSAICIGHTLLYEFKVLSLTMEKFVRRATKLAEGKKFE<br>GIRINSEKQLYEKLTACLKDSVKHHDILLDVSEQYKSIFYVPELVILLSSTMVICLSAISLTSDDIPLEAKALSLILT<br>GAEMVNVFVNCYYGQVLLDAHDELGDAIYGSGWTSCSTTVRQHILIILSRVQRPLSLSAGGFAAVNLDTFAQ<br>VVKSSFSYFSLLQALKE
AlucOR29	MTEAEVKDGTKKVDDKLGCIDYRKYTFARMIMIDDGLAARGLTFPLLLIMVVNVGMQTCSFISIFTSTQTSVS<br>LDNIRACLLGTSVTMSLFNQFISRQAIARLHGFFDKSFRTISTNPNFPEEKIILDDARKAASSQLEMYVKMFSC<br>NAVAMMFAQPLGELMSGHSWRKLPVQWTFPPSDDEFSFWLIFAFQFTGICIAHCVGIMIMSFTSITIQMTALF<br>DLLIFSIQHIEERATARGRQSGGDRHACLLACLTDDVDFYQQLIREMDSVTPHLRNTFLIISAAVPMVLACEAY<br>PIMQGNFSFADLVKSFLFLSIQILCWAQTCSRLGTMTDKHAAVFAALYDSPWYEAGMKYKKLVLNSLTYAVQ<br>PKYIKARLSNEVTASMATFYSFVMSAFNLLNMIRNIG
AlucOR27	MDGNIERHFDHMEKFLKYYWQWYGYPDTFKGRLITIFNIFRIFLLMVLVGIAASQAYFYGMSYLVDGSAIFLP<br>LGIMSLVLNSHQSWNFGSIVKTAKQFELFLSSFQDEEERNLIASRMKERKRTVLQIVLIIEFYLMAPLMCHAIS<br>MTLHYYGFLKKPVLIPLLFEMFLEGNYELGPKLIATAVVSVFYVHLVANIVTMILLNVHFLGLVVACLEVLTER

	LKGFAEKTQEGYGKLKEELDLRDTIQQHADLLHIINSFNSWNGFLVTFCLAACSITFCLDALTTKRALEQEIYS<br>GACLWGSFLLVMMVLSYLICDSGSQIETKSEELLRAVYNLPWYRGSSETRKAVWMMLTQGNRLIILNYKELM<br>DLNMVTYLEMLKRAYSYFMILSSIE
AlucOR25	MSFLFKFFAYLAEGEDDELWDTYYNFYGPFLEISFVFPSWRRSRIPVSLTVLGFFAFTFPVHLWLLTLGIEGVRD<br>DFNLASMEFHYWMLLMFSMISIFLMNSHRQNMIDFHRRLSRDVGFHGPGRIYDEDYPMKLEHNKRKQLLQ<br>FLFLPGLVLVLAGATLILPYLSKMDGTVLYNSREVNMKLPIPLWYPFPTHEGILSIMAVLGQFMAAGGLSAVIM<br>TLDLIIFRATQAMIFEYKVLRYAIDTLVPRAKRLYAQEYPMMDLESVKMSDDAFQICIGKCLRACVIHHQDIN<br>RLLNLYKVMLKWPGFMAYGFGTAVIGLSLINILSAKQNGDYENIVLFFGLSMAEVLNMLMMSVFGELISTESK<br>ELRQELYFIEWHNLNTFNRKLMLGFQMGLNNPVIVKVGGLVTVSLETFSSIMNTSYSFFNLVNAQ
AlucOR24	MISLTIGVRKQLQSTTNIMDNMFSFFAATPSFLGMGKIICLLRQRRALRRIWKSLDDLLENVLKRDVDEQLEK<br>ELRWRLKRCWAMYSIFLTVGTCITLHWLLRPIVYALYGERTSIVSTWPTYLESWIQWFTTYIFQAMNISSIGHAL<br>YIYDNVYFCICENLLIHFAVIKHHLHQMDISKGKPGGVTMKFCISHHVKLMDICMELRECSKYVIMQQVFWT<br>IFILCPGVFELVSGRQTDTTILFNLMEITTIMTCILFFYSWYSNEVTLQSSQVFNTCYMSNWVEGTPNQRRTMM<br>TMMTRSMKPMIFGGLVNVDLGTFISCFFRC
AlucOR23	MWLKYPSDEELNRLGICRSTFDGNVFFKAQLGGLCWFSNSWSVFYFISTIFGIFSGCGFAYGVFMEKEWEALY<br>EALHYIPLVVNITSTAASYHYTQDEYLQVFRSIDKEMFDYEGTLDEQAVEEIARMKTEARARKKKISMIYTKL<br>MIVAFICQTLRKPLNYIIDGRGKKDVDGENNLIWDVCPFGIYMPYADYWAPYLIGHFLCWSCSAFSAITAVAS<br>ALTYQAICEELLADYSALDLTLSTIVQRAERLFSNMNRGFTGNASGTVTFDYCLEKCLKVSIKHHHEIIRLFNI<br>VKKLLYIPLFFTIFDTGIVMCFSGFIMISDDFSPKFKLLLSPVMLAESGIAFMFCYYGEELTEMNKNIGNRIYFSQ<br>DWMKHFKSIKPYALTVKSYCDIPNELSAGGFTKVNRLAFSNILSAAYSYVGLLLTTS
AlucOR22	MIRLRLPPTINQDTSQKNIKTIYEELDSQFHTGIRFLLMGASPEKFLGKLSLTYVLIYLSIILFFMLYALFEIIVPFFF<br>PGDFLEFMNHIYFGSYCLAFGYQWIYLLMNLNNIYLNRLNIESLYSTQAVSGIAEAALRKNLRPFKIAIKCSIAL<br>WSITILAFGLGSLIEIAVEYLLTGEVESYAIMSTFPFPPWGQMLVTALNFVTLNMGFAEVVAMAYISSLLALEIET<br>QCEILCAAMLQDRDDWFKFRGYISDHARIIKNAKWLIGILESLNAPTVFSAYILMAIEMVVLTLVEPDGLFFVA<br>MASDLMGVVIILIFQGWISSKITLSLQSISFAAYQTSWHEQDKNKALDLMIVTQMAQRTYVQKILLGTVVIERG<br>TVLQIARSAYSFYTLLMVLQSNKTL
AlucOR21	MFLVFMVTVGILFQGAKLLWNKNETTFDLLFSLVHGRENHCTYTGTPYDEFLKSRRHTFLQYGPTLFTYTNIT<br>VMSMYVWLPPLVDCIMGVTHHPNDPHNGIATTLGLPMWTPLDADHSWTSYFIVIYMQITFFRIVTVSWAFGI<br>FYTSIFQLTLLDEMRLLRKSLQEIDTRATQLFTLKYQRQPVNRKTKEYDDCYYECLKENIIHHQFIRKIYGEYQK<br>RVGWTIAIPIYLSSVVLALSSSYIMLDNRNALKRSMDVLRIIGDSLLTFLTCHAGEMIAYENDELRNELYNTEW<br>YDRSKKVKQAISICLHITYMPMRLYGGYMVFNHELLSTIVNSAYSVFNFFRVIQKSAK
AlucOR20	MVIQVRNMEQGLKEITKQYYAAAGISGISAYLKNDKPFFLIRLWVWISTWIILPQVTLAHIYTALFTGIPLATRF<br>LSLSLGLDQLQTTIKCHYMLHNLNRFRSIMLDLETFNCVNHLGTEALTTMLKMCKLVKWLRSTYNFACYFTF<br>IAWTLVPVVSTPKALFYAEDGVSMMKVLPPEYPFSTDYFPMVQLIYALESFSTFVILTYFASTNLIMVTDILLICQ<br>LFRVLNESVAPSKGRKAMTLRMFAVDHQKLLKICAEVRDLLSPLLALQLGISVMTITLAVFEITMVNQASSEG<br>GINQVVLMSRKTSYTFIIFVELLLYCWLSTELELSCLSVRNGLYNSEWYKYEQLGTKDYRNYIMICMRALKPVR<br>LTAMNIVTLSLTTSMEVLRMAYSYYTYLKKLR
AlucOR109	MKRELKAEAVRHFTRLVVKKPMDLLPRLHRGRGVFNLEETSSGRSDSESKLPRAQHNVFYQQFRPMILVLTA<br>FGRLAIQRGSDGEYRWKWFSWLSLFCLLNYAVQTYFAVAICRQRIKAVFESSNYDEFIFAIHILAYIQMHFHVP<br>VSYWVQGPLYAQYLNQWSQFQNEWFLVTGEELKFRHKKAALTFVLVMLPFLALVLVMEKYSTLHDPFEYLL<br>PHFFTIGSTVCVLGTWYIACLEIAFISKDLTKHLIKKLHSNPDPTFLQKWRALWMNLGSLVTNLGTNHFAIMS<br>SIFLTFSVTFLLGLYNALSKIIIGDFSLKTIGYLTASGMSIIIIYIVCDSGHQATSRVESYTSQCILRAHLPAARDDVK<br>YEVDLILRVVQTDPPQIQLAGFVTLNRPLFISFVANTITYLIVILQFKG
AlucOR17	MSKLKNLPFITILWREFKFLSVMGGTYGFYHTKAWTAVTVINYIVMYSAMLFTMSVLVYTTYLLRDNIGYMSQ

	ALHLLIVGCVTTTASFTITINRYKVEKFVVFFEDPWSLCEYSRNDFFEELMKETQKKKTRLIVTWILIYGICGVIG<br>LLQSGINTVFGTQSELTDVNGAWVILPFIMWWPEDITASTGAWMRAFLVQSLYLYFCVIMVISGVVFAFSAVER<br>ILDQVKLLIYGIKTLDRRAKDMFQQKFPGSDMKYMEKEYDDCYYECLVQNVKHHHKMIKWIETFLDMASLP<br>IAVPFYGGAVLLGMALITITEKDDPRVGPKCLAACLAFSEAYNMYLLCDVGQRLENLSQELYDTLYFSRWHTR<br>SPKVKRAIQIMKIGCQKPIVFTAAKLLVLNMSLFADLVNSAYSIFNLKAASEKFEDK
AlucOR16	MTLLSFFKDKFKWNEPLGITETTATLFGAFVNYAPSPGYRKFFRFFGWYIIFMFIIFNINVVLTIYFASDFFEESLE<br>AVRLFVTAIHILAKLLTMRAMEKQYMELIEQIRRAWRTYEYSSGDMLNKTLAAANKGTVIVFVAIGNTIPINVI<br>VAALKNLGNPPEIQFSMQCWVPPSLRTSFLAGSFYQLTPYFFPVMLYCMTISFLNSITLHVEALGLALAKEIRS<br>QKEWRDEAARSLYIKHQEVVRIVGRVNDLMASNWGFEMMCATLQLTLVSYNALRTLKKNDVAFFNQANL<br>MLVNFLVIYFIYGNGNRIIKMGEELHNSLYDTKWYTSTVKERKNVLFMMFRTSMPMEYRFKIAHFDLPSFAKL<br>VNTVFSYITLLRSVDEPEEQGAF
AlucOR14	MLTVGSSAHNSLSRILEVIGITRYKEASFFSENSFKLFRVVQWALNVLMIVSCLNFIFSESVDDSPDKLQSLALCT<br>ADIQFLTTQLILISRQSLVDDLVAHLRSVYHGIKEIPGSMDILAEGDRQARLFIMSYGVVIFSNVPSSMFFAGIKM<br>ILTGETAYPFPMSIFGLPTAVGWALQMLMIANAANILWGFYCVLKTVIYILGAYSNVMAHMLRERPIDVEAK<br>NDRKMLKLHCDINSLSLKLVNVYGLISFLEVTVASGRCCFVAYHILLAVQEGDYKNLGVALSTLLTSVAITYVL<br>CSCGEEISMQSVTIRDGVRDSKWYAVSPPARKTLLPVLLFTQRPIQFHYRRFVYFNLETFRNVLKTAYTMTTAL<br>AQV
AlucOR13	MAFIMRLIDKCAAMENDDLDRLLDNYYGPMFKLGLVFPSWKRSALVFTIPWFILNVSTFTWNLILLGITVYK<br>AFLCDNDMDLFSLSTHYFLLLLCGSLIIFFMNWNRKLNGLNHTRISVDVGKYKDSRLYSHKDCILMEKQIRVE<br>SYRYLCLPLLIVLICGAVLIVPYASKLFRGVGTMYTTCGVDMFLPIPLYHPFPTHEGLNHFLALISQVLVVCCLA<br>NVIVAIMLNFTQYSLRVKLEYQVLGYSLDTLFARSKKVYLKNYPNEKASFHIRNPDYQRIVGSLLRDSIVHHQ<br>TLVDMMDKYHGLITYPVVFAYLTGSGAIGLGLLSIVRALQKGDTETLLLFSLLMLGEVISMLTMSLIGESVTEAT<br>IMLRYKLYDIRWYDMDIPNRRSLLNFQTFITEPLVLTAGKGLVNLTMETFSSIMNSAYSFFNLVNIQQSE
AlucOR10	MDRHEGPLSTHFRRLFRLVGVYNGKYITPHSCLFFFSGFVNIIYLSYILIFTDCNMTKVAHFIILQYFYIGTLWTV<br>VYKGNDIIWIANECDKFVGLDGHHFERLYDEVREQEKTSPATKGKTIIDRIASIICTWTCIEPFINAWTGKAELE<br>FPFTGTNADTTKFIFIYLMQCSLMFIVAIVCSVIFKSLMGIALDLVIKYKVIGLVLSSLNEQMMNHRNVYKSDL<br>HQTIRKCVQSHHHVLRIFEKYRDICTYGFRYSYVGLIGATSLSRSLLSSDDPDLGTIPHIIAELSYMGFFCYILNQ<br>VEQEHDKLKDAVFAADWPWLPKPATSSLRLIMMRTAKTPRVILVKGGGPANLETFYKLLNGTCGYLIFGLVL<br>DQAAF
AlucOR9	MNLIGLPPREDDRKAVDYRKCTFFKILYTENETGGKVSMARGVFVIVTLSIMATCCLVSVTRSQTAEQLLDNL<br>KGMHLEVMVLMVAINECVSRKRMRRFMSYVERFRANPRYDLPGEEAILVQARSNAIRDITFLVIIFGANFPLM<br>MLTKPVTEALGGGSWKQLPFPWTVLPDDEDTTFVAFLLLHTLGVFFSHCLGVVGMCFSTITTQITALFDVLLL<br>SIERIEERAARKMKQLGLSYQESMLICLKESVAHHQELVQEVRSEKPHLESQFFSEIVNISTIMACEAFPLVRPN<br>LTISIAIKGLVFLVVQVLCTAVLCDRMEIMADQNTEVFAALYNTPWYKCGVEYRRILLNGMTFCRHPLTIRGK<br>SFLGLIATRATFYTAMVNTFNLLSMIRKMG
AlucOR7	MLFGAGSKELGFRKETIEEEDVGFMQLAGLYPLTRGYTAYYFISFILCTTLMEGMIVGSYLEGEVDTSLETAHV<br>LLISLNMFTQICTHRYYYDIVNKLLRAIDDNFFSYGDTMDEDTKQVIRKLAKEKTARKKMFGKVFKIQVSSAA<br>IAITFQRPILYVLNGRGVKDVDGENWLIYQSPFGILVPFSNYWVPYLFGMVLVVNQVITTSITAMATATSFVRFS<br>EELLHQLEIVKLGLGNFMFRARHLHSLRYNQSKESGEQDKHLDKCIITCLSKSVEHHAIIIKLFGDFKNMMYI<br>PLFTVIFDGSVLICMSAVQLITSKNPIVRMSMPPFIVAELYYTYLYCSYAEKLTNMVLRTAYSLLNFLATRK
AlucOR5	MKIVTSDSNVQPVGSTSESKMVERKPQNLTKQLSLSRNPVRTRKERQKRTKTIYDQLDTLFHVGVRCLLLGIS<br>PDKFRGNKGYERFLIYFVVILFFFAYSLFQLCAPYFFPGDFLQGMRHFYFGSYCLTFGYQWIYLLRNFDKINQN<br>RVNLKCFNSSRAVTEIAEVILEGKLKPFTRRIVCIAMIFSIASFSHGLGELIQFGEQYFIMGEVKTMLTYFPFPIW<br>GQLIIVLLNTITISLLLAGTVSALIITGLLTLEIETQCAVLCAAMVRGTDDGSQSFTGLIADHVQIIKNARWLIDL

	MESLNSPAFSCFYFHIAIGLVIMKLVETNGYLFYVSMAFGFMSVILQTGAQGWFSSAITHSLESIAFAAYETNW<br>YERDKNYARDVLMVIQMGQLRFVQKIFFRSIEIDRATVLRIVRSSYSFYTILMIIQ
AlucOR4	MVLVPYLKPQKERNAAVDRGYDITGMFYARLAGLYPDLEIGWRYWFFGSYQILVVVAYFYYVLAYVIANVIAI<br>KYMDVELIGSTLCFGSYTYTYALIALTFYIKRSKIDKLLEIIGNELYIYQCPLSQKQLKIRNEETTRAKNFGRYSFF<br>VPCLVALTHMSVVPAIHGFKGEYSSIVNGSAPINKYTPLPVWTPVQATSGMSFFVVFWCQLCPGFVEFLIFHGS<br>CTFFVGVVCVLVSEIKILLESLNSITDRAKYLYHVKGGRGSDIDNLYDDPIYQQCMVDCLKENVKHHIKIKEFR<br>NLFQDIISYCIFFIFGGAAVTISTPPYTILKIMESGDTDKLYSAGVVMMGHTFLSVYLLSRYCKFGQNFESENSKL<br>LEAFYCTPWYNTNMDYRKILIIAMSNSQKTLQIKGSVVGVSLSAAAFLDVIKSSYSLLNFLATAGS
AlucOR3	MSAQTIQSDGGRALLNRDDVKGLNMGLNTFGAKTFWHTLEHFHATGKRHWVMATYIVLLHLVGFTYCLL<br>GFSAVFFIKMDIKRGTAAIMNPICGLQTVFKCWTFSWSTAEYLKLFEILKKDFLTCVPPQKEGGANDVLAKNV<br>VATNEFVKNAMRWNFLTLCMVSTMPYLRSQAFREFFRLGEGAIVPNKICENEYPFEWNSTPIYEIIWIYEQIAV<br>ILAIVTSSAYQAILLFLVMALVGHLRVLGYVMENLRASDFRGETYQLMDKSAKANAYQQLIRCIRDHQKINA<br>AGDALAERYNFFLTFHLGTAIIVGIIAIFNCTAADELADKIKFAIMCGYGLLEVAIYCYCGQLLENASEDVLRQ<br>VYQCEWEEMEPKFRKAAQLMMVRANNPIALRAGRLYRVNLETLGAIQQLVYSALTMLSSMIDGS
AlucOR2	MLLCWKKKKEKQKPVIKEQHDFANNYRLFKYIGMIQDGSLFSRIRVFLATFLLFYAWFHHLIPLVNSTEEYSFD<br>ELMDLIHLEMVYFLWCIVWPSYIIRAPLFTSLASKIQNGLYTYSDPLTLEEKTILSTANDAVVRMTKISVYVYVC<br>GGIGIFLKGMNKERMRKLQLPNIGWFPFAINSLSRYAIGCLCQAIMGINAISIAIGTFMSFAIFLIHYEAQFKLLR<br>THLKRSFPKNVPLRIAQTDKYKKVSLRRLKDCYRHHLAILGFHQEIMKYYGILLLVFRVAIVMWMCTLAYVT<br>VMVDVNAHNLLKMLSFASTELLYVFLFSFRGQDVTDWNYQWREELYSIQWWEQPKEVKTNIGIMVLGTTQP<br>LLLYGVWKIALYSHEKLSDIGNESFSFFNMLRAIN
AlucOR1	MLPGFILLVANCVIMVPYISKWVVGMKNPYTPRGVNLNLPVACWLPFHSHTGFWHVVAVSNQLIAVSCLAV<br>IIITLLFMFLKFSQKVRYELKVLHYGIETLFKRSKRLYFKMYPERKAIRFHWTDPVYQRVVGICLKDSILHHKTI<br>VNILDVFMTMVSIPAALAYVIGTAVIGLSLLSILNALNQGNYPNVILFALLCVGEILNMLVASLIGETLTHETIIL<br>REELYFIEWHKLNLSNRKTMLNFQTAITEPLSMKAAGLVDMNMDTFSSIMNSAYSFFNLVNAQ
AlucOR74	MVEDLTVKDLTGIYLLPHSVAYMHFTGHWIGAVPGPTPFRVKMYRAFGGTFTWLIVIATAIASLNGMLHGSG<br>MSDISMNLIIISTSISSLHKYSVFIHQEQGLGRLGRWMKRANEQNKISKNPDTTTDRILKKSLVSFYYSGIVAASL<br>LLVKIVVTGYTYNALIPGLDQRYQPLILIFMEAFSFSSLSLEVIMDALILMNSLFVFRRELMKNVDEWRKMNYK<br>SDNPQQFRQQLKTNVQNHVELLTIFQDVKNYCNSMFGYQVFAIVF
AlucOR72	MSVNWNRKLNSLNHTRIAFDVGKYKDGRIYSHKACIRMEKELQKETILYLCIPLLIIILGGAILIVPYGSKLVRG<br>YGMMYTACGVDLFLPIPLYHPFPTHEGIHHFLALISQVLLVFCLMNGIIAGVLNFLQYSQRVKLEYRVLSYSLD<br>TLFARSKRVYLRHYPDKKANFTIRDPEFQHILGSLLRDSIIHHQTLVDMMNNYHGLITYPVAVGYMTGAGGI<br>GLGLLSILRALQKR
AlucOR64	MSGFHGFWDKWWYGDPPMDDKIFDAIHQEFNQILYFCGLFPDPRPIRRLLTFGILILNIVICVTYMYLLGATA<br>VLQNEDFFTASQTAHFASCDLLSLICIISLLLNRNRMIEMFRASAHNYYDYDDDAKISEMREEYMTTKKQKTII<br>LIGLPSYLSLIGMVCLMSRTIDEYLGGGSNETNIDGVYQLTPVPMWYPFSIQSEPMHWIAVSSQAIAMFSTATSI<br>GGTAAILVIFSQSISLQFKIIIYRIRKAEKHAYQLYRMNGGKKLKNVQLYSDPSFLGFYNSNLNKLAEHHSILIRQ<br>FDSLYQVVKWPAGCALLLG
AlucOR44	MKSKVFYFTDDAPKSTWDKSTRIYHRLRPVLLVVLLIENAIGLYFLEGFGVMDGSIIYLPICLLLFVINSTAYFSR<br>KAQDRLIATLNKHFTESNEPWMLTVSHKYTTKVWKYIKLILIYDKACKCMYLAAPLICDTVLHYVFDMLEKP<br>FYLPTFITPWLPKDVTWGPHYYLILVFGWWGMLECLNGIMGFMIGYTLLVTYVLIQVVIFKEKVKSAKIDGSQ<br>EEVDAEFRTLLSWHVDNLQLNRDLKAYFGFTCAFQSIFLSVGLTLTVFTAVSSKVIAVKIAYGVGFFFYFGTGLL<br>YCSLGQLLENEVQSKHSEKFEYDYATVSHTAHTRLPRSMQNELRKFHADFETVLFIFYLTTISNIVGLKFFLPSS<br>PTFITVGTKVVGTKVVGTKLAS
AlucOR26	MGYVWSKLQLKIKLWDSWEDEFSIDVMRHRYRGFHRIGFIVLDLSSKYAKLSIITALLGTSFLLMATLSLLATC

	AKMSDDFEECSGVCNLGFLSTLALAFLVNHNYFRKTILSAHHMLGKGFHDYQEPEYSSTEFEKYKTMLRKQ NVALIILACYVALIGILVVVVCPLIDESLGFGWTEPYDEYGVNRQLPVTLWLPFYSHEGINHWFTFLFIEGLGG AMICLSIGGSALLFTSLTIGTMLEQKRLVLSIRDIEQRARHRFQTQYKGKPGVDDEGKNVAL
AlucOR11	MFRGKEKERPYVIKDPKDFDTDRLVFLWLGFVYDGSFFSKVRLTVFVLLLYSAPIHHMLPVILDKSTTTDEVLI ALSINMLYVLLCIAWPFMIYRSPEIIRLWSTVRQGFFHYSDPLTSYERTILSKANDLIIKSTRMSLIAYFCAGFGTY LKEMSPNSMRLYNPPYPGWFPWTINSNFRFAMALLYQLSICLNTTFALEGIFLLFLFHTISFEGQISLLKQHFED TFPPGLPTEMTHVPAFKERTLKRLKECVRHHLVIMDFHKRILSYFGICLLVYRAICTIMLCILCYLTTTGIALNK FLQLACLAALILYLLFIFCLKGQKVSKMSEIWRETLYEVDWWNHPVEVQKAVLLMLVGAGKTMTVYGVWTP AMYSHEGISAIGQETFSFFNMLRAMK
AlucOR39	MGYRVYPQQDLSDPSHMFSFQLNALKSTTMWKPDNQKYYIPFMILFAVNVFVLAICTVGLLLKGCSTKDLVD RSEAMDIFTLTGSALYKMVFFLYHYEQLVDMVTCGLALVRNLPEGWTKNCGLLSRIHYTAGFLVLLIWGLAPI LKVMYGETTWAEMKLPINTYDPFDSTGFLFFLFYITGQYVLVLSAVIYMAADCYLFTSIYVAVGALQYIVDQFE NMRDLNNNNKHTVADTMHDCLQECIEIHVHVLDYLRKTDKLFKSMILADVVHAVISLSFAMLQTSESKGIF EGVKMVLFVQVCFVHQFLNSHFGQELIDKQDNLAKQIITDIPWTDASRKFKKSYYIMLTCVREPFKLSAWNV YFLQYATFLEFSKTMIQYYMVLQEVQDEAEVS
AlucOR30	MVEKSNFHVKRAQLFKAYNSIHWLTLTKWFYEDYPVEKLWSDKRLWIHLSIVIICQSSITMFKVFHLISEENFFI FLTSLTSFLVIVLVAVRTYILYQFPTFRQLYFKPEVFNCNLHRPTRSLALLTEAITHSRKVGMWCLVLFITFDVAFL VLPIVPPILEIIDGTNKTYDELIPQYPSINPVSLSWLSKELKYAFDLVMAVFNTIPWVGFVVVYYTVVQLFRAQH KIMMTAMLPGPPVPGDGREPLELKLWIQDHALIRKLVYKLRNTISPALAGTICVNVFTVGLNMLALVSSPIGS DAPMFTRYLYYFSFGTYSALSIFDIFIHCWLASEITNCGEDLSYALLKSDWQNDLKRSHHHYVLPLMLCKKQI RFTGLGLIPVTLTTFTETIRVSYSYFTLLRKTDD
AlucOR28	MAGYGRLEDGDIVDGLSIWYLKASGLWEMFNHHRETGGRSKVLKFWMAGMIIAYSPVFVVSVVGPFFAEKD LEGMSLVVLNPMSTVQMVVKFGILWFHMEKQSRLLDLMKKNFLACVPPDKEAEVSRILGDAVKEANIYTFF GTRINIITVLLWSILPVLRSEYFRITLGITIFGTPLRHNKLLGFSYPFDYDASPGNEIVFVYEFLLVLSAGLIITVME CLVAQLVVLLTAYLKVFQYFMEELKSTHDPKFDKEQLLLYVKEHQKLMRVGDEVCDLYNFLITVQLSTGLFILI IAIFNFFLSSGNGDVVVMIKFVVYTLYTLVEICVYCYAGSNLETTSEDVCFAAYSCEWYEMNPDFRKTLQMMM VRSRSPVVLKAGKLYPLNLITLTNIVQMAYSTSMLMYQQTHN
AlucOR18	MSFSFVEKYQLSPETEKTMVTEYSYLLYVGGLLINYRPKVWIISIAQTSIFIGLITSYTIIFIISTAKSSNFVAFSQNL NYASLCCICLGLYFAGLSHRSAFVRLMEIIHDDFYDYGDSFDNAEVAMWKSSLRTFKIIIVVGIPTYLIIIAVSIVL GDYIDTALGYDSTDEDYLGEIYQKAPLNLWYPFVVTNMFLRVAVTLSQMTTAAILATTLATGDVMMLFLGQT VALQLRILCLAATKMDQRANLMYEKGLARSSSGDKEDLDGCYKLCIKQLVQHHLIIKEFYKTYYTIAKWPTAI AFMNGSLMIAMSIIVAMNGNEETPSTYISTYLLLVAEVLSMWLLCETGQNVNTWSEKLFMDTYEFNWNGLS VPNKKMLLIFKENIKKPLLMMAGGLTPINRDTFATIMNTSYSYVNLLRASERRSND
AlucOR12	MKFIDKLAEEEDDELIEILKGNYWHFLFYSMTFIRWKRPRIAIALISAYAIWIIVHLVIGIYSIYLAADERNWAVV GLVTHHMVLGALAIYLPIFCNTGGFREVMADMHRTFTTDIGQYSGGNMYAEQACIDIKKDVRRQTFVYYIN PALVAAAGSLALAGPFLTKWFSGMENPYSPNGLSLKLPTALYYPFPTDSGVVFYAIVLTQVISGTILGYLILAPQ LVFINLSQNLKRELRFVGYSMETLVRRAMRMTFENNVWKRKVTELDVDDTEFQQNVELSIKETIIHHQKASK LLSTAQVSVKGPLAASYIFGLVTIAISLYNITLALKTNDIGSLTTFLLLLSSEVIGTFINCGLIGSELTEQSEDVTEKL YFIEWYNFSVKNRKMFFTFQTAITQPYEIKAGGVTPMNMETFSDIMNSAYSFFNILQTIE
AlucOrco	MQKVKMHGLVGDLWPNIRLMQLTGHWLLEYHEENGGMLRLLRMAYCWMTTFSIYIQYAFLVCFLILETYN ADEMAAVTITTLFFLHSVTKFTYFAFRSSYFYRTLGAWNQVNSHPLFAESNARHRATALSRMRKLLMIIGTVTI LAVFGWTTVTFLDEPVWDKTDPDNVNETISVEIPQLMVYAWYPWDARYGMTYFMTFVFQLYWLFITLAHSN LLDVLFCCFVIFACEQLKHLKEILQPLMELSAALDSVVPNSGDLFKAGSAGSDIALIGNGENGNDFDVRGIYS SQRDFSGFQGGVVNGGTVGPNGLTKRQELLVRSAIKYWVERHKHVVKFVSSIGDTYGSALLLHMLTSTVTLT

	LLAYQATKIEAVDVYAASTIGYLVYTLGQVFVFCIHGNELIEESSSVMEAAYSCHWYDGSEEAKTFVQIVCQQCQKSLTVSGAKFFTVSLDLFASVFGAVVTYFMVLVQLK
AlinOR86	MKTVLVHEDIAKFIHFMKARTMWYGTTVPWDDSLFDRFRKSYTDLKWLFCLLFFFMCGFSLSFADNFGLFDGTFIYWPICFMMTLLTSIAKFTARKQDVLMTSLNDHFLRNTEPWMRSIKDDYIKSLWKFIHFFSSYQLLVSILYMVVPFVADLILHYGFDYLESPISMPTPLSPIIKYNNAWNVKHFAVTVVNLWAFVEVVLIVQWFIANFSLTTVFVLTELIIFKHQVKSLNFEDEDNWEQQVKNIVDKHNQMIRLCKDLKDYLGLSSALVCFFTSLVLTFTTFTMYASSDITLRLSYGCGFSLYFSSALLNSYLGQKLENESDEVFKALYGLRWYRFKPEARKSLNMMMRQARDPLIIDFHGRYKMNLSNFIS
AlinOR85	MEEENKARKNKGDNLGCVDYRKYTFARLIMIDDGLAERGLTLPLLTIMVINVCMESCSFISIFLSTQASVSLNNVRSFLLGSSVTLSLFNQYISRKEIARLHDFFEKSFRSFRTDFPEERTILDDARKAATAQLDLYVKIFACNTVAMIFAQPLGGLLSGNSWKKLPVDWTFPPSDNEFSFWLIFLFQVTGVCIAHCVGIMIMSFTSITIQMTALFDVLVFCIENVEKRATTRSQQTGRPYHPSLLACLKDDVAHYQQLIRELSSATPYLRNTVLIISAAVPMVMACEAYPLMQGNIILGDLVKSFLFLSIQFLCWAQTCSRLETMTDRHDAVFNALYDSPWFDTGFEYRKLVFNTMTFSTHPKYIKARLSNEVTATMATFYSFVMSSFNLLNMIRNIG
AlinOR84	MTKKIYEKPERIYWDSLGFSQLSVFLDLNPPIDSSKFLWHLKRKTAQFFTFIFSPIFISVHIWSILSASPYNLKQISFDLGFLSHNVQNLIKMFFWLTNLTSVRDLCLDFSQFHVNRYRPLLSSWVLQKESNVTRKYIERCFLISKGGLIIWVFIPTSIALINYFLYVSGLSAGQETYIPRLSVTQFPFNMSYLGNRLFVGTLEYGQMISVFIYYQPIDMFLIAAVNMVRTQYLILNFGLFWMPKDLEAFWGTEVSVKDTEPPMDMRLFVEDHQRLVRYGQKLREVLNPVLGIVTFDCIILMCNCAIFITKLLEDQLELLELVLYMTAYITIILASLVVFYTFSSMSGMLKEAEESVFDALYAQDWYRMSTQKKRSMIFIRKQTITARKIPMLNLGDMDRATFIEGLRAVYTFYHFAKQFK
AlinOR83	MSKQSGPLDNIVDYRKHLSYTMTFTESSQGKLSKPKVIGTAIMMFIIPLGCFVSVVKSEELKESLENFKGVMAEVMMISVLLNETIFGPEREERLFEYLSKCHMTTRYGLAEEHTIIRNAGDSARKETFVYALLFIVNCPLMLLTKPVMAAIAGQSWKQLPLPWTLPEGDDVVLNAVLLLQIVGLLISHVVAIVMMSVLAITTQLNAQFDVVILGLRRIEDLAAQKATEYGLSHSDSMLSCIKESVAHHQELIRELLLVKPHLETEFFCQIMTISIIMACEVYPLIKKDLELSDAVRGIWFLIVQVLCTALICNKMEVMSNKNVEVCDALYNTCWYDCDIRYRRVVLNAITFSQNPIPIRGKGFLGMKACRETFYSAMVSTYNMLNMLRNAQ
AlinOR82	MNWFGWWAEEMDRPLVVRLLQIMRAMLIPSHMIFYATLLYQTSHEFREGTIISTVKSAFVSGPSTVACFKLYVIVRHRKSLKEITNSMDVMMKGILSRHIPADLEEEMKSRWGSCRKLYKVCVYFGCSVTTHASVTPLLQTIAGALLTDDPLPFDSWPYFLLGYYCWALNTFCIGHVLYMFDATWFAMADNLQIHFVVLKNYLENLDLTKQGDVDLNLCLKNHIELIRLCRIFRRISRTVIVTTRMCSMLLLCAGTFVLTSAGSEFTPNDRGNLLSTLIYIAAVFFNYCRCADNIAHQLDELTTDCYSAKWVHADKSQKNSILNMMTITRMEPKFCGIASIDLDTFVNVMRGVYSYYNFLTAVDVGDESGEPNADSNTPL
AlinOR81	MDRPSDPTRSNGPKFKAFDRLEKWQRSGYKLLLYGMSAKRFMKSSSQRAVVYLVVVMTLGLYSLHELIAALFFSRSLLEGISHAYIMTYILTFLIQWFYMLQHVRSFHENEIYLEKFESTQAHSSFADHILDRNMKDFLKYIMFCAGWWATNNFTHLIGPLIEFSLAFIRTGEFIQISLLPQVFDLPKWGQVAMYIHNAVMTFSFFMYCGANSLILGTRVLKVKTQCDILNEALRNDHGVESNIKAFIKDHIVILRAAKMLNGELADLNLVIFTGSYMEIATQMFTLTLFEPSGVYFFAVAFDFGSIFLITATQCWLSSTLTIALDTVSDGVYDTEWYGRDKNHALNVLIMLQMAQQEKSHRIWFNSFKIDRSAALSLLQSSYAVYTFLMIVQSN
AlinOR80	MSVEKNLIKSKAFKDNFGQYMVWSGIYCGNNVYPGCVLIFYIVHTFFLLYTLILSISKDNEKLMGETAHFTAFRFSAILLLINGLWKKEDLEALFLKLCQPQVHDYGNTLSDQCRKDIEGARQNCKARKDFYGPNFMRTVTVALVIFWIRSLMEYFNGHLDNPKSDDGINSNLPVPTYLPYESHEWPGYHFALVCEVLLVMMSYFLVLGHDCSFICFAEEILRELDILIITLTEVERRIDHVRKSSNYKISQEESVRLCLKHSVMHHQKVIHIFEHFQHYCFHSLFFMLSGGAFLICLSSLMFTSDTISLRDKSVFLMFLGNELFHIFIFCYYGEHIMGKSDDVGNSLYNSSWVGISKHVKPTFMLLNLRCQVPLTLSAGGFMTASFDTYGNVLRTAYSYLNLLQATN

AlinOR79	MSGIGRIGDDEIVNGLDIWYLKCSGLWDVFNDYRDSGARNKLFRIWMVITVMFFAPLAFLSTFGPFFVEADLE<br>GLTLIILNPMSCSQTVIKFAVLWYGIETQCKLLDLFKNDFLTCVPPDKKLKASRILTASAKKANILAYLGIFMDA<br>ATVAVWNILPILRSEFFRVQLGITAFGTPMKHNKILGFWYPVDYDVAPYVQFVYCYEFFTCFWAGFIIALLEGLI<br>VQLILLLTANIRVLQYLLEEIKASNSNLNSETLLLYAKEYQKLLVVGDDMRHLYNSLITMQLSTGLIILIITIFNFF<br>LSSGNGDIVIMFKFVIYLMYTLVEVALYCYVGSDLETTGDEIGFATYCSEWYKVGVKFRKTLQMMMVRSRYS<br>MAIKFGRLYPVNLMALTNILQMAYSTSMLLYRITNKEEENRV
AlinOR78	MSRSGRLKEEDILDGLSLRYLKVSRLWGILNHHRETGGRSKVLKAWAIGMVIAFTPLLMVTVVGPIFTEKDLE<br>GMTVIFLNPIATVQMIFKFGIFWFHMEQQTKLLDLMRKDYLSCVPADQKAAVSEIMINAVKDANIYTFFGSR<br>MNIITVSLWNILPVLRSEFFRLTLGISIFGTPVDHNKILGFWYPFDFDGTPGNEIVYVYEFILCIWTGFIITLLECLI<br>AQLVVLLTGHLKAFQYLMENFRSSHDPRMDHRKLLSYVKEHQKLSRAGESICELYNFLITVQLATGLVILILG<br>AFNFILGSGKGDVVVMIKFVVYTMYTLVEVTVYCNAGQNLETTSEDVCFAAYSCDWYEMGVDFRKTLQMM<br>MVRSQSPIVMKAGKLYPVNLTALNNILHMAYSTSMLLYRIKVPEEKQVM
AlinOR77	MDTLIRKNNIDKYLPWHHPDLTKVWRWPIARYLNTFGWWSEDYTRPSVKFLFRVIKFTFLAVNVVYLISLTIG<br>VHRQLQSTTNFMDNMFAFFAATPSYLGAAKIFALIIQRRELRRIWKNLDDLLKDVLKRGVDDKLDRELRWR<br>MKRCWLMYSIFLTVGSCITLHWLLRPLVYAMYGERTSIVSTWPSYLENWPLWFATYVFQAMNISSVGHALYIY<br>DNVYFCICENILIHFAIVKHHLHEMDISTGKPGGITMNFCISHHIRLTSVCMDLRECSKYVIMQQVFWTIFIICP<br>AVFELISGRQTDTTIVVNLLEITTIMTCILFLYSWYSNEVTLQSSQIYNTCYMSDWVQGTASQRKTLMTMMTRS<br>MKPIIFGGLVNVDLGTFISVLKTTFSYYQFLDTMDKNKRKLDDTS
AlinOR76	MVNMQRVREVEDVKTRSGLSKLIGPCGGYRGPLYNEYYSKNIFFRAYVHFTDLAVIINYITMIAAAIHSKSVLE<br>FVMVGFPISAESLSLFLSYYSGYKNKEMTNVLLGFDDCFDDDPYPEHLESEINKAAKYYYHFSRTLLWLQVFT<br>MEIYCFIFPVTNELMQDYFRPRALPLPSLYPCDWKESRSCFIMIIFIHFLGATYVNWKIIAFGEVFFAMVSRQVA<br>LFRHLNHNLNKILTAIQVSENGVILYRLNKEADHMHIKKALRKWIKHHQSVMAQYDRLQALYSWPLFVHF<br>GLVSGALCCSAYATSDETLDFDANLLCGGFLMGQMLELFYLCRIGDWITIETNELTLALTGSFTSVLDRIESQM<br>LRIILSRVHRPSVMRAVGLYPLNTATLKMLIQSTYSYYTMLKKVNKG
AlinOR75	MGSLLDKFDEWAEVEDEDFWKFIWKSYGPLIQISCMFPSYRRSMLPITLLTFAVHLLLLVPHFLLLVITIYQTIVD<br>WDLELCSLAIHFSLMIFYAIFTLFYVQYIRYAYSSQAKAMSTDIGNYISGRIYEDKWCVRAKEENWQETLRLLIG<br>PAFLMLGSGAVLILPYTLKTIKGLDNPYGAGMVNANLPIPAWYPFPTHEGAVHWIAVLYQLVAALSVGVIMM<br>QVLLLFLSNAQRLRFELSVVGYSMTSILKRSMKLHLRANPGLNRDEVDMWDPKFQSVVESCLRDSLLHHQTI<br>LELLSLFTTQTDTLVLLGYSVGTASIGMSLFNILRALNTQSYESIMLFTIMIMGETLVMYILSYIGESITSQTSDLR<br>NQLYYSPWNNFDLRNRKIFLNFHTAITEPIVITAGGLIPISLDTFSSIMNTAYSCFNLLNTQPEI
AlinOR74	MGKLEISWPQLNYLAIYGLWPVSVGGIRFRKVQWLWGRLIVTLTVMVIVLEIVNLHELLMANEMDLNDFVIT<br>MNDILYATQSICILVNILVYKKQFAIMMDELGEMLDHSQLLMKSYAEHVFTKKLRVCRFAFIAFLSGTLLMAH<br>WGISPLLNRILYHRNTFVYHVWIPFDVEPWYIYFSVLTAQITIGVSWIMGQPMFASLFISISEHLLGHFDVLRQG<br>LETLDYSAPSASQEVNRYFEYHQKILKVGYILRSATKLVFLSQFLCVTSIMCLNLYEITYVDVETSRFLNMVEYT<br>LLELLIIALYCSYCNELTLRGSEIMSSAYFSGWESASIQDRKSLWIFMTSTKAPLNYGGMVKMDWTTFVNILKT<br>AFSFYNFLGAVRTSKEGQITNG
AlinOR73	MAISDHQIEWLTQEEVLVFDNLTKLLAIVGTTSRSKKWYRMFSLIFWLAAYIPMIVGLTVTAVHYIKDLDFLAY<br>TLHHLVLITVAVFVTHLVVPLFDTQILYVMNASKNTYNYESDFVENYAKKIIRKRIKLSRTITKMIYFGMVWIM<br>LEVQVFFMIETFFLKSYQTVFPLAIGLDLNRWLVFVPVVLWQELIVYYTTFLPTTLAVLCYTAWSHLDLEMRIL<br>TYAIANVQKIVNEKLSQDGGPGSVSEIYVSYCNHFAKHHADIISEYRLAVSWITPLVFFIGAILFITVGLSLMSD<br>NLGIKLKLFWFLVVQYIVIYFWCLISQKIGEKSEEISEVVVSTPWWLMPRSCQSTLLLIMTRCKKPLVLSTPLGA<br>DVNVESFMEMMKSVYQAISVVYQMKTTS
AlinOR72	MKEFKSKTHALLLTMLEVAAIHRNPERSMFSPNGFLIYRVGLCLSYITMITSSLLYLNEEGGNQLMKMVLSSV<br>ALQFFVCSMVLISKKESITKMLLASTEVFSQLTPNDENRIKMDLLDKKSLKFAKFFAFLISMNVISGVMKGLM

	NSWLSGETHYPFDLSCLGLSAVPCWFTQLFSTSGTIIYIYCYFAILKLILYQLWGYVDVLSALIRDRPVSASEKDDRDLLKFYCDYGNFAVSFTSIFGITAFIDMIFTSVRCGLISYYIVMSISENNWSDAAGSMVGLASSFILPYIVCSCGEDMDEMNNQIRNGFLESNWYQCSPQSRKRLLPILTLNNVPIKFQYRQCMHFNMERFMQVLRSSYSLTTALANFM
AlinOR71	MEADDGTVNEVLLNSGLINWIGIVGGIRFAQRTRFKKNFQSTIYWFYEIFTDLTVFINILSQIFALMASEKMTERCLIGFPLVSCSFCFFISNYPRFKRQEFATLVLRYDDVFPNTQYSDHLEEEIKKAAERVRLTSTILVFLELGPMFTFCLILPLVNEASGFALGPRKVAIPSLWPWDPLASFKNYIILVFIHAWASIFVNLKKIGFEESFFIFASRQVALLRHLRYNLEKLFDPLEVNVDGTVNKNEFTSPQRYWMEEKLTQWVKDHQHCLRLFYELEALYKWPICIYFGATILILCTSTFVTSDNSIDSQTCIICGVFTTGIFCELFFICRMGDHIQIETEELLLGLSGKNAFLSNWKEFKYLRMIMTRCQKESVIRAAGGFPLTISTFKSITTSSYSYYTLLKEVNGKLTE
AlinOR69	MKEEKEDNSSGCTDYRKYIFCRLMCIDDRLVDRGLTWPLLLIMAQTVAMETCSFVSIFKSTEIKNGMDCVRSFLLGNLVTMVLFNEFTHRRRLARLHEFLEKSMSTLRTGLPEEEEILKNARDQASSNLKMYMMVFFGNIAPMVFAQPLGEWLSGHSWKRLPIPWSFPPSDTDWAFIFIFLFQFVGVVMANCLGMVFMSFSSITIQVTALFDVLLLSLRHMEMRAKLKGQIEYLDYHQSLYSCLRDDVIFYEQLVRELESITPHLRNTFLAFSATVPMIMACEAYPIMLGNFAIADLIKSFVFLAIQFMCWAQTCMKLETMTDQHVAVFRTLYDTPWYEAGVRFKKLIFMSLTYASPPKYIKARMSNEITATTATFYSFVVSSFNWLNLIRKMS
AlinOR68	MSFVSNILSSWAKDEDDELWEVYHKLYGPALELSFTFPSWRRSRLPLTLLIFGYYAIIFPFHIALLSVSIDCVRDDFNMASMEFHFWMIFMFSSISILLMNSNRQSMMKLHRTLTIGVGKYKAGRIYNEDYPVMLERKKPKQTIKFLFLPGLVMVLAGLTLLIPYVKNMDGTVEYNHRGANMKLPIAACYPFPTHEGITYLLAVAGQFMAAGGLALVIATLDLLLFRMTQSVIFEYEVLIYALQTMSSRAKKLYRITYPNSNIKEVRTKDEAFQRCIGECIRDCIVHHHDIIKIVKEYMNLVKWPGLLAYGFGTGVIGLSLVNILSAKEAGNYENIILFMLLMIAEVLNMFMISSFGEAITTESKVLREQLYFIDWPKLDTQNRKMMLNFQVGINNPVILKVGGIVNVTLDTFSSIMNTSYSFFNLVNAQ
AlinOR67	MGYREYPKRDLSDPSHLFNFHLSALRVVTMWKLNDMKYHTPFMILFFWNVTVLSICTVGLFIKGCTTDDLVDRSEAMDIFTLTGSAMYKMVYFIYYHDDLVDMITCGLALTEKLPKGWTKHCTLFAKFHCCGGIFCMTFWGLVPMFKTLLGETTLEEMKLPINTYYPFEGRFAFSVTYSIAQYALMVSGQIYMAADIYLFTSIYVAVGALQYISDELEKMNENSNLKIADVGKVDQTHEHLKECMELHVNVLDYIRKTDKLFRSMILADVVHAIISLSFAMLQASEAKGLFEGLKMAVFVVVCFLHQFLNSHFGQTLIDKQDHLIEKIRIAVPWREASRSFKKSYHIMITSNTNAIKLSAWSAYYLQYATFLEFSKSMISYYMVLRELQDQEEVP
AlinOR66	MAETGRLKDEDLVEGLSVWYLKASGLWGIFNHYRETRGKSAVFTAWMVGSVIGFGPLFLTSAVGPFFTANDLEGMTLVILNPLSALQMAVKFTVLWFGLDTQMHLLDLMRNNYLTCVPASRQARAKDILKKAAKKANFMANMGIAANAITVSFWNIFPILRSDYVRLTLGITFFGEPKDHNNKILGFWYPVNHKETPWTQILYVYEFIICFWAGFIITLLEALIAQQVVLLAAYLEVIQYLMSELKKTQSAYLDNKTLLSFIIEHQRLMRVGDEMRQLYNFLITMTLSTGLIILIISIFNFFLGTGKGDIILTIKFVIYTMYTLVEVCVYCYAGVELETTSKEVGFAAYNCDWYVMGPDFRKTLQMMMIRTGSPVSLKAGKLYPVNLTTLTNILQVAYSTSTILFRITNKDNTGLDMV
AlinOR65	MKNLKKGTSSWRDLKGLAREEALRRGYAENGGTYVKMGAQYVATRNDIWMPVVFFSDLMLAVFQLTAAGYFSVIDGDMEAASECFHFITMISNMMIITANLIYYKNIFDDLFVATGSGFFDYGDSLDPQTKNEMEAYISNMKVKKRFRFRTFVLVVWTLGGSMLFKVALAYFRFGDRIDGEGGSVSRKHIVAQWFFGIDKWPNYIFMASVTYIAEVLVMTSVWGFVLPVICFAEESTAQLNVIGLGLKRTSARARYVFTCRFGEYKSVHKLKYEQCVREVLRASVQHHNAVLDVCNEMRTLLNLPLMTVMFNTAVLLCMSGFVMIEDSVPIIAKIISLLFMIGEVIYSYIFCLYGEMMTSTSEEIGNQLYQDNWKEVSVVIKPYLAMIKMRSSKPIRLSAGGFMEVNEAAFSGIISSSYSYFNLMLTSKS
AlinOR64	MFIFIREKELERNPDNMKGRELLEARFAIFSGIYPDFYGWRYYYFVLFLIFIHPGLYCYFLYAYGISFYYGMLYADVELLGQVLCLGTITVIYCIVSIYYIARKTDMDDLITMVGKGFLNYSRGLTEQEKTIIDRMEKVTHRYAFGSTAMLMAISLVHMGLLPMIRGLKGQFTSITNETAPINKFTPLPVWMPFECNSTRSFVLTFIWQIIPGCMEYAIINACCILYVGLAQQLSGNLEILANSIRDIHTRALIMFENDGGILSKITGELYENSHFLKCINACLNENIEHHVKLIEFFNKF

	QGVAGFSMLAIFSGTGLIISTAAYSLLLIAQTGGDSELLITNAFVWTFYLFVYTFLLTVYCYYGQEVTDKNDALL<br>PALYETPWLEADLPFRRSLLISMSYSQRTMELSAFGLIQSSYATLLDIIKTAFSYLNMLMAVQ
AlinOR63	MGRSVPDLPDLVRLLRFCGLYYTFNGPRAKVYENLQPIRPAFVVATSIMGIIALFVGGLRSSLGIEMCYALLGLV<br>TSLQCVHFYLRREDTESTIELFQEIREKFQKGSEEEFKNNTRGIWMVVKVYGFLLVGTCMAMSLPFCTDLVIW<br>AIWKTPKAFRIPMGMDSFLDKEPIRDAKYFFVMFAAGSWTIIGSIAQLGADAFLFVACFSYSSMVMTFCKSLTI<br>HSNLTPKETTAHLKRVAAHQQQLFKLSVKMRLLFGLPFFVQNLFGAICICSLLFVISTDDSINMLKMFAYLINL<br>LVTVSILGCTAYVGQHVKNKTSEVFEAIYEMNWYELRPSDRRYLVTMLSAAREPMSIDFYGLLPLDMENFMKI<br>LNTSYSYFMFLKSMI
AlinOR62	MSYFSFVEENILPQDLEATMFREYSYLLYIGGLIINYRPFIWIISVIQTVFFISLTIAHALIFSITTVKSSTFVSFSQNL<br>NFASLCCIALGLYFAGHSHRESLVRLLRIMTDDFYDYGDSFDNSEVAEWKKKFRTFKLVIVIGIPVYLSIIAASV<br>VLREYIDNAFGYRITDKPDYIGDIYQKTPVPVWYPFKITNSFTWLATTLLQMLVAAILATTLATGDILMLFLGQ<br>TVALQLRILCLATTKIDQRAQKMLKNGIGKLAPGRKADLDECYRICIKQMVEHHLIIKEFYSTYYVIAKWPTAI<br>AFMNGSLMIAMSIIVAMTGTEESPSIYISAYLLLTAEVMSMWLLCETGQNVNTMSEKLYMVTYDCDWMGAD<br>WSTSNKKLLLIFKENIKKPLLMMAGGLTPINRDTFATIMNTSYSYVNLLRASEQRTN
AlinOR61	MPSSMSFALIRMMLTGTKTFPFAMSVFGMPTFFGWVLQMLLVANAANILWGFYCVLKIVIYVLGSYSNVLAH<br>MLRERPVDVQPKEDRQMLKLFCDINSLSVKLGNIYGLIAFIETSMASGRCCFLAYHILLAVQNSDYNNLGVPL<br>STFLTSVAITFALCSCGEDISKQTDTIRKGVMDSKWYAVSPANRKTLLPLLMFTQKPIQFHYKRFMYFNMETFR<br>NVLKTTYTMTTALAQV
AlinOR60	MSTVVLHNDIAAYINRMKSFTVWYGSSVSYGDSPYSRYKKFYSRIKWVLFLVLLFVNGFCLYLLDDFGLFDGN<br>FIYWPICLELFVLTSIARFSGQQQEAITMSLNDCFLKNTEPWMRAIKDKYISGMWKFVNFFALYNNVTLFLYLF<br>GPLVADTILHYGFDYLQKPFALPMPLSPIFKYEDSWNTVHYVVTIINFWATAEVVFVFQWFFANFSLLTTFFLT<br>ELIIFKHQVKSLNFERDDSLDQQVEDIVNKHNQIIEMSKDLKGYLGLSAAFVCFITSLALTFTAYTMYSSSDLPL<br>RVTYGSGFLLYFLGALLFSTLGQKLENECDEVFKALYGLRWYRFTPNARKSLNMIMRQARYPITIDYHSRYKM<br>NLSNFMQILRSSYSYFTLLQSMASKSSASRL
AlinOR59	MDDPGGRALEKSGLNNVMSILAGFQGPREVRFKGTKYQHVFTAYSYFGLCVSHYILICCYLTPLFITDMSFKD<br>MMFFAVPTITSTFSTLRVYYMMWNRSKIIRLLIKNEEASEDDYYQDKQEEEIKKWANTAKVLQPILYFAVCAPI<br>APWGVSPIINEILGNPWGPRKATIISWYPYDVQKTHFWVLTVCLQTMAGCYATLSNVMFDAVFICIAARQLA<br>LLIHLKNSFSKIFQVFHVHLNGNVWYSNYNGEVVERKEIEDVVIQRLKYWIKKHQTVLRLLDESQSMYSFPLF<br>LHFCYVGMVLATGSAAVLKGTLTSMEYYFIGMHLVGLSFTLFIICRIGDYIKIETDEITEGLYGQNYFMLSKEQH<br>MLIKNILTAVKQPFVFTVAQAFPLSTETFKSVMTTTYSFFAMFTQMQHKN
AlinOR58	MWDWIQLRMFNLWGWWPKVIKDPRKRVMMRIYGYCMFGLDSATMIAEFVSLYLAVVNGSFRGAIVNIVTT<br>TLGATAAMKIYTLLVHHEFISHICNTLEDLNNRAIALMGEESQVTMGTRKRNCKLTFVFVGSCLFTVCHYNV<br>RPILVYTLYGERTIAMDMWTPWDEQTSLSGWVIVLIYEWIHIAAAMYGMVVFDSFVLCIFEMVLAEFDVLKIA<br>LKKVDFAAEKNAVPIEFCIQFHQDLLVLIAKINDFLIPIQTFQCIMLTLTICFSGFELVSLSDVSMNKAANLLEVL<br>GASTYITFGYCYQCHCITEECVEVITTACDNNWFEGSIKDQKSLSILLERAKNPISFGNIIKFDLGCFIAIIKTAFS<br>YYQVLEAFDIH
AlinOR57	MFARPNMEFVQTWHYYGYWNIFLGGKYDSYKKVALVVRLILFTAGAFTALAKISSDGVESVLNGALIYVPLC<br>VYTFVIPTILFFKRKNLFSLLKVFDECWASLNDEEDRAVFEDYYQKTWKFVRIIHVGLFCQLTYYITFPILYSLVF<br>HYIFDVLSQPYILSYPHMSYLDKNFTWNGEYYAVVFAGVYCIAEIEFALLGWVLMYVVIVGYCYPVLVVTRNA<br>VQAVGSNRSPSSGDRAMKKAVVAHSLLVKVNQDLKAFLGLPCAFQSIFTSLCLTLSVFTTIRSSDYFVEGAYGS<br>AIILYSLSCLLYCSLGQILENQSEKLFEDLYNLPWYQFSPLVRKDLNMMLRQAGKPFTIDFHGGYNMNLENYM<br>EILKSAYSFFTLLETLASNT
AlinOR56	MLCCRKKKEKQQPVVQEQNDFVNNYRLFKYIGMIQDGSIFSRIRVPIATFLLFYAWFHHLIPLIMNYDEYSFDE<br>LMDLIHLEMVYFLWCIVWPSYIIRSPLLTSLTSKIKSGLYTYSDPLTLEEKTILSTANDAVARITKISVYVYVCGGI

	GIFLKGMNKEKMRKLSLPNIGWFPFAINSLSRYAIGCLCQAIMGVNAVSIAIGTFMSFAMFMIHYEAQFKLLR<br>THLRRSFPKNIPLRIAQTDKYKEATLRKLKDCYNHHLAILEYHEELLKYYGILLLVFRVAIVLWLCTLAYVTVIV<br>DINAHNLLKMMSFASTELLFVFLFSFRGQDVTDWNYEWREELYSIEWWEQPKEVQTNIEVMILGTTEPLLLY<br>GVWKIALYSHEGLFAIGNESFSFFNMLRAMN
AlinOR55	MIRNWQDEEEGQSLLARMGCKFLNGHSIYVGSWVLRFPVRLPLFLYVTCAVGISIKMVLNYDNLVLIIDCAH<br>MMIHMVVGIQTTLICTKQKGRIMKLKDQLDKFDDVPGKEASSTRIKEDYELQVMNLYRTFSRCILVTINIYILF<br>PICKLFTEAGRAKLSKVLVWQMWLWVPEETWWGFTIIFLFELVTSLFLLLSVMYAVPYLACLGTITVAHCKVLI<br>LRLKSLQKRAEERSGKMSESYEAALNYEIDGCARRLHENLRIANEVADVYKYYLSSFYYGGMFALCMSGLQA<br>VAANENIEESLKFMGVLTGELVAIGLATYVSEGIIEGFADVRASIYDLPWYVYPKLSCKRLHLMSTMSSFRGLR<br>TMFGYELTLLHFGDVLNASYKYYNLLLLSMK
AlinOR54	MNFSKMKMLPLFAKKTRTCGNNRKSVNKKWDAIFKLGLRMLYLGASPDQVWRGERNVLRFGFFPLLILLYS<br>GFGLLIVVFPFIFESDFMESLENLYLGTYFVAAGFQWAYLQIHLDGIYEHRTKIESFNSTQAIPDVANSIARRNIK<br>NFIKLFWVLVILWNVGIGLFTIKITMKIITLLTGESDSSKVDSDSFQNSQSWRLTVDFMNAVMTLLSLPISLSLIIIS<br>RFLTLEVVTQCEIIRAAMALDTGKKVMFKGYILDHIRVIKNAKWLIKRLEAVNTANTLLSYLMFALNMFMLT<br>LVEPALYTYGVLALYFFIDFLQLAAQGWFSTSVKIALESLSDGSYETIWYERDRSNALDVLIVSQMAQQECIQK<br>VLFGSLRIERATAISIVRSSYSFYTLLMVLQT
AlinOR53	MAEGEAYFDYMVQILKNYNLWYGYSTASIQGIILTMYSVLQIFLMLGLLSAAISQTYFYGMSYIVHDSAVFLPI<br>GFMGLVIIIYMALNYSAIMKTASKFELFLNSFTVDWEEELIKNHMKDTRTVVTLMVNVISFYVLSTGAMHFNS<br>ISLHYFFGIFSKPVLLPLVLEKFMEGNFQLRPMFFVHVLLSLLYIKIAASIITVIALNIHFSGSAVGALQVLIKRLE<br>NVSKKTADGCHKLAADSDLRDTIQKHVELLNIVSDMMEWNGFIVSFTLTSCSILFCINAITVKKSIETKEYSST<br>CVWGSFLLVALAIGGTMCSRGQEIEKMSEELLRAMYNLPWYRESSKSRRNIVLMIAQGNRLISLDYKGLMRV<br>NMVTYSEMVQKAYSYFMILGSVE
AlinOR52	MMKNKRESSKPTQKGTMRLGEWFSFHFSPQKTSKKKPANTAYHDGVPDFANSVTCFTNVRYSLLYLDGSLV<br>NYLKICIPIFFLATGTISFAMADIVHMNEKNTIWIVENGHWCIIYTAVLFWDAHMGWFSPIILEMNNSVKYGV<br>YRYEKYEANNRMEFGKNNAWISKMNRVFGVIYIVAVIGTLVKSTILEEQYPFKHLFNGWFPFEINSFMRLNIV<br>RIYELGCAWSAASGAQTFFMTTMAYTYHVEAHLRLLMQKVAKVLDSQHPEKQIQECLAHHRAILRLFNNLS<br>AFFDPTVGFSTLTATFMVCTLLYLITNPDFDMNVIVTFSFLVAPELGLLVSVRIRGQKLTDLSSEVNKTIYDLNW<br>LEQDIKLQKDLLMWLRLTSKPLELKAFGYRNVSHAGVKEVLQTSYTFFNMLKAST
AlinOR51	MKDHLILDDMGEVDIEWLTEEERALIISFDKVHSWTGMWRNAKRIQWSYFWLFQMTMFMIIYFYSLYFFLEE<br>LEILTHVIHHIIMAGDDFMYIYLLNYNRRNLEIVHDLNLKTYGYGSDLVKNYHRKLMTERLKTYRLVYRFILLS<br>AAATILYLEAFFVLEATILKTYVTMYPIYLPIDLNHPVTYTSVVFLQHLQVYVTLVMGSGLVSILFSAWNHITLE<br>LAVLTFAMNNIEEIVKEQLSRFQFKSHGTKEAARNKIYRSCCYHLARHHGSIARYFNTFKSASRLTISCIFLTGI<br>VCFACVGITTVTDNMGIKLKFFLIMVVQTSVIYAWCAVGQYISDQNANIQWVISGIPWWKMPKPCHSTLRLI<br>MVGTSMPWFLTTPLGQDANNESFMDMVTSSYRIFNLVYQMMFSS
AlinOR50	MFRREKKERPYVIKDPKDFATDRKVFLWLGFVYDGSIFSKLRLTVFVLLLYSAPIHHMLPVILDKSTTTDEVLIA<br>LSINMLYVLLCIAWPFMIYRSSDIIGLWDTVRQGFYHYSDPLSEHERSILSKANDLIVKSTRMSLIAYFCAGFGT<br>YLKEMSPNSMRLYNPPYPGWFPWTINSNFRFAMALLYQLSICLNTTFALEGIFLLFLFHTISFEGQIGLLKQHFE<br>DTFPPGLPAELTHTPAFKQRTLNRIKECVRHHLVITGFHKHILKYFGLCLLVYRAICTIMLCILCYLTTTGIALN<br>KFLQLACLAALILYLLFIFCLKGQQVSKVSEGWRETLYEVDWWNHPVEVQKAILLMLVGAGKTLTIYGVWKP<br>AMYSHEGISAIGQETFSFFNMLRAMK
AlinOR49	MKNLQTVVPSRGWSGKYITRSMFRREYARNQLLFRSEYYYMSRPILWLQRSFGRMPYSVISGWLRHSNWSISF<br>IYAVFVALVNVGSHFYYHEHITDAWIQTMRDSVNFKSVLFSYLLVTQPPTCFVTIYSWLYELPRIVKCYNSTAIL<br>EHKISGVFPTSTRSRCTRLMVPFGLASLLIASLVVGSLLLILRFREQPKILLIIIAINLIANYSYNALWCFNFFFISD<br>LAAKLRKHMLLCLQDKKNCSFKLKTCRKIWISIWKQSQSYAQSIAVTVGYSLIINSILFIIGCYGAIASFRSNDIL

	DVVEKLPYVLVTFINAALIFESSYQATDKLGNAFLNTLVFLNKDSVDQESVEEIEQFVDTINHTRNAAITLRGY<br>MTMDRTLLVSFMSNSITYLIVLLQFQGKSQDSGMSTSNSTSPVT
AlinOR47	MVVEKKTTTDFEREVRQTVGEITAEDVFPVQLRVLQIVAMWPAFNGSPQKQMLTKVILNFNSFVLAFCTVGL<br>LLKGIITRDLVDRSEAMDIFTLTGSALYKMVFFYIYHNEMADMMNCGAALIHQVPPKWMQYVTFFTVLHCF<br>MGFICITFWALCPILKLIFGETELNEMTLPINLYDPMEAKGAMFALFYFICHYGLISSAHIYMAADCYLFTAIHL<br>ANGGFQTLNGKLEDMKQAIAHKGDTSHDSANEYIKDCIKLHVHILTFIRKTDALFRSLFVADVVHAIISLSFA<br>MLQASESKGIFENLKMTVFVSYCFVHQYLNSYFGQHLIDQQDTLFEKLLLSVPWNDCSRNLRKSYHIMTTGC<br>LKSVRLSAWSVYTLQFATFLEFVKTMISYFMVLRQVQDETEVQIEI
AlinOR46	MFYTKRMAELFAMDTPKRKTNRRKYYWYLLELGGLITNWDPRYYIWTIAYLIVQLILGFYFLICFLVSVFKAV<br>NLMGMCVYLNLFALLMLSTFMLIVTILLQEKLLIFTSVADDAFYEYGNSLTRTEEIRQMNENAAKMRKMMFII<br>APSWISLVALSIMLSDLVDVAFSYPASNETVINGIDQRLPCKMWIILPIDNIIVRLLTILAQGVCFGGAAVVIGTA<br>DLIMFFSGQTLVIQLKILNMAVLDTDKRAAKLYETNLGRKPPSEPSEKSKDLQLMGFYEFCLKQTVEHHCAIL<br>RYVVIYSELINWPGGLMVINGSIVVAMSMLSLMQGGGQPSVLITSCLLIVAEVASIFMVCEVGETVTDQCRELF<br>DSMYQFKWMDCSAEIGKSINIMKSYMIKRPIVLTAGSLTPIDRNTFGAMMNTAYSYMNLVMASGAVD
AlinOR45	MICSVYLFLFGVTAIKQIDDFVTATQTVHFALIISGSILAMVTVAIKRKHFIIIVRTMSVKYFDYGDSFIIPEMEEE<br>YKNTKKQRIIFLTVIPMYFAISTVVLGLGRTIDGYFGSPLNETYVNDVYMLTPEPVWYPMKVDSDILYWFLALS<br>VVSMSYAACITVAGGDWILFVLYLSITQQFKILIYRMRRINAYAYRLHRKAGGVKLRKSMMFSNSSFLKYFNLC<br>LSKHAEHHSIIIKQFEQLSIIMSWPAGFIFIFGSVIIAMSLLGVSVQGGGKPSILVLATFLTFSEIGEMALFCGLSESI<br>QTLGLSLHEELYRLNWADADISAKRTIMIMIEQSKRPNVLKAAGLQSLDWMAFSSIINTAYSYVNILMAVDA
AlinOR44	MSSNTVYISEKSDAAKKATEYVYERLFIFTIFGGFFGFHHTRWWSMFTYTVFTLYYISVISMIVTMSYSSFLNQS<br>NTSVMSGCLHMTITGLVVSCASITLQLARKDLFKLLVNFVLDENLCEYQSNEYFSHLLKKADEKLRSLLILWL<br>LLYGSAASIAVVFPFVDVYLGYELTLNNVTNVYWRGLPFTLWWPMDADNSTFAWMTCFTSQGLYAMFAASL<br>CTGCMVFYAMMCENIFNHIKLLVFSLEHLDERATLMFKKLYPGKSPKKMRDLYDECYYACIVQNVKHHHRI<br>VIFKDAVMKAANLPIAMPFFGGALLLGLAGINLLSHDDNRIAPKVFFSCLGMTEAGQMFLLCKYGERYQDL<br>SEVLFNASFYTSCFRRSMKCRRAMMIFRLGVSKPMRMTAAKIILLNMETFANLVNSAYSIFNLQSVTS
AlinOR43	MKSGGRSFNKRRAELLKAYNAIHWLTFTKWFYEDNPIEDNVWRDKRLWVHFAFAFICQSNIIIFKVFHLVSEE<br>NFFATLTSLTSGLVVLLVVVRTYVLYQIPTFKQLYFKPEIFNCNKHRPTSSLDVLIQTVKHSRKVGIWCMMLFLT<br>FDVSWLVLPIVPPIIEIIKGTNQTYDELIPQYPSINPVRLTWLTKEAKYAFDLFMGAVNTIPWVGFVVVYYAVVQ<br>LFLAQHKIMMLSMTRGPQVPGDAKEPLELRLWIQDHALIRKLVYQLRSTVSPALAGTICANVFTVGLNMLAL<br>ISSPIGPEAPLYTRYLFYFSFGTYSAISIFDIFIHCWLSSEISNSGKELNYAIYAGDWNSDLKRPPQDNVIPLMVCN<br>KEIRFTALGLIPVTMTTFTEVIRISYSYFTILKETGH
AlinOR42	MKDAKPAMEPKRNLFTLSLRLMALQGMARPSNRILRMISFVWKYWMLYTALHFVMICLLAVLIGDNPYYLN<br>LETCSGMFSGMSMVYRHFVLAFNRKEVLRLVARINALVEEIVDVYGVKTILPWENLCYGIMIFSTCIVTFTTIPA<br>YAYSYYVFYTTGEVTAPYEVYMPFERDEHHIHHVVIFQFLSFIDQAMTLVVSNTFIGTIVVIVSEITKIIAARYEMI<br>RKKTVDTLKDTIRWHSEVIMIVEDTNALLGSVIMMDCLLSIVHISVSGYLLVKVGIENGTNLHKYVFLNLLCV<br>TIPSYFCLCGHVIALGRDRLRQAVYQNEWYDLNPHDKKLIILPSWMADKGLALHFKRAVEFNLPTYLAIIKQS<br>YSFIAMLQLMDS
AlinOR41	MTQSELGMFNGLKLNSSVRIEKWISFGYRLLLLGMTPEKFLDKSPAGRVRVYLTLFMIQSFYCCHEIAAVVYFS<br>RSLLERITHGYIMTYVVAFNMEWYFLLYCVRSFHENELHLERFESTQAHFEFSERAFDRNTKVFLTCLAICVA<br>WWGTNNSIYIFGPLLETVMSFIRSGEFKQVSILPQVFSMPWWLQIIVYIHNALLIFSALVYCVSSFIILGSKILKVK<br>TQCDILSEALKNDTGEESNVRAYVKDHIQIIKAAKLLNEQLATLNAIIFTACYLQIAIQMFTLTLFEPSGAYFFAI<br>AFDSLSIFLILSIQCWFASIITLALESVSDAVYETDWYRRDKTDTLDVLLMLQMAQQEVSQKIWFKSLKVERAA<br>SLNMIRSSYAMYTALMIFQD
AlinOR40	MFGAASEKLGFNERTLEEEDIAFMQFCGLYPLNKFYTIYYFTSTTISLFTLGGMVVRSYLDEEMDVAFETAHVL

	LIATNMFTQNLISHYCHHIVEMLLRAIDKEFYSYGDTMDEETKKIINELNNEKIARKKMSVKLFKFQVACAGI<br>GVIVKRLLLVLFTDSASKKVDGENWGIYQAPLSIYIPYSNYWGPYLLGMFLSCNSVLTIALTAMGAATTFIRFSE<br>ELLQQLQIIKLGLRNVLARAHHLHTIKYGRPYPLVNGETKEDFDKCLSICLIKSVEHHTIVIKLFEEFKKMMKV<br>PLFTVIFDGGALICMSMALLITSESASMKLLMPSFIAAELYYTYIYCAYGEKLTNLFTEIGDQLFLADWLIHQKT<br>MKPYMLIMKAYSFYPKKLTAGGFTTPNLESFGNVLRTAYSLLNFLVTQQ
AlinOR39	MGLMEFFDKLAEPEDEKLMGVFKKLYGPFVQIALIYPSFRRENLPLTFFLFIFTFSVFLYHYYLLSMSIFKSLDNF<br>ELASLAFHYWMIFTFTLITLPLLIINRHKMGETHRYLQDNLGEYKSVKIYQEGKPTQYEKNKRLEFIRFLFLPG<br>MVVCLASGLLLIPYIRRFTNPPHYSENGVNLNLPIAAWYPYSTHEGINHGLAVLGQLMTGGNLALTLGTLEVI<br>LFRVAQSIIFEYKVLQYGIETVFSRAKKLYWKRNASLAKAHIHFKDPEYQKCVTECFKECVRHHYKIRVVLAG<br>FEYLIKWPGALAYGFGTGVIGLSLVNVLIAKESENYENVVLFLLLSVVESLNMFMLSVIGESITTETKVLRDELY<br>FIEWYKLDITNRRMMLNFQMGVTNPVIVYAGGLVALCMDTFSSIMNTSYSFFNLVNADMGNKDEK
AlinOR38	MSFLLRFIQNLAAEEDEEFLNLLRKDYWIFLHASMVLPTWKHPIVSFCLFFHHATCLFMHFVIFSYSMYLLLQ<br>EGNLEVFSLVLHYNVILSFAVFLVVYCNYMRKELVRLHKIFVTDIGIYRNGRIYSDKWCTDMYKIIQLEKYYFL<br>MIPGLMAAMGGLVCVVPYVFKSLVGIEHPYSSTGLSMTLPVPAWYPFPTHEGIWHLVVMLGQFQACGLTAFL<br>IVNCQYMLLNITSKLKYEIRVIGHSLDTLLTRSQTFHDRITSIHHGQHKPVTVAILQTVEHHGKKRDVLTSIDD<br>EGFQRFIGEEFKDSISHHQTIAAFLSEFQSFGSVPLAAALLLGVVVIAMSLYNILMGMRVDDMGIIVTFSLVIITE<br>TLAMYVVTALGASLTDELELLRSTAYFMSWEELDKGNRKIFLNFFTVITEPFALKAAGITDLNMETFSSLLNSA<br>YSFFNIMNSVD
AlinOR37	MNILGINPDRDDGRAVDYRIYPFFKILYSESEDGGKVSMARAVFVIITLAIMASGCLWSVTQSQTAEQMLDNL<br>KGMHLEVMVLMVAINECVSRPRMRRIMAYIDKSRANPRYGFPEEETIMLEASKKARADMTFLVIIFAANFPL<br>MMVTKPVTESVGGHSWKQLPFPWTILPDDDDMIYVAILLFHTLGVGFSHCLGIVGMCFSTITTQITALFDVLL<br>LGIERIEERAARKMKQLGLSYEESMLRCIEESVAHHQELIREVRSEKPHLESQFFAEIVNISMIMACEAFPLIRP<br>NLTVLIAIKGLVFLVVQVICTAVLCDRLEIMADQNTEVFNALYNSPWYKCGVDYGRIVSIGMTFSQHSLTIRGK<br>SFLGLIATRATFYTAMVNTFNLLSMIRKMS
AlinOR35	MSELHADIAKYVTLMKSTRYWYFPEDSTSSPAVDMLRRYYYHARPFLFFITLIVNAYGIYNTEGMGVLDGNLL<br>FIPLSLLSVVTTSTIYFNRAQHRKLTMLLNQRFLNNNEPWMVEIQTKYTSTMWKFLKIVIVYQRFCAATYIVVP<br>FIVDTILHYVFNYLDAPFFFPLTFSVFLPRHITWDVNYYAIMFLDAWAGFEIIANLQGFIICYTVITLFSLIEIQIFK<br>EKIKSLDFEVSMENRQQQFQMIVQWHNDIIGLNLDLKDFLGPTCAFQSLFTSLVLTLTVFTTTVTTSIPVILAYS<br>CGCFFYFSAGLLYCSLGQLLENESSEVFDALCNLPWYRSGSDVRKNLNMMIRQSHNPLIIDFHGHYKMNLEN<br>FMQILKSAYSYFTLLQSMTSSG
AlinOR34	MQTEGDKIVQPLIDALKFGGLWFDFSDHKYGEALKWCNIIRNAIAFLVWGIITGYFFIGGLSFLLTESGVFMPIS<br>FDEGCMSIIVICNLPAVRNVIQIYNKRFDSFSSIPWARSIIDEEMNKFNKIFQLPKTALVVFYCLYSIAPLMYDGY<br>RACVGNENPYVVSLPLNFLLELPMRRTPTFFLTVYLANIYFLIIVPRFIAFEALVLYMVAFVVIDVKIFIRKMEKL<br>SENDDGTEFIQKAWNLKDVTLHHSSIVCVVRDHFPLLGFAILLQNVSNSISSCLVIYLMKTSYNNGDIILAIFCG<br>NFFVILMVINLMFNGAGVIIENQGELLLAAIYNTGWYKQPPSVRKEVNFMLMQGLKLLKISYKLNSVNLEAA<br>MLVMNRAYSFFTLINTGE
AlinOR33	MIPFIFKKKDSNDPIVMRGYELTYSYAMRLGGLYPDFRGFRYYIFGFHLNCIHVAYLWFILAYIISAYYGFVYND<br>MELVSYNICYGLLTIIFFLVGHSVMYKRNHLDRLFETVGKGFFTYEKPLDADEQAIVDECDMKCKKTAKRNIL<br>LTMVLTTWTCVVPPLPKALKGEYSSVVAGGVPINKHLPLPVWSPYPIDTPLTYWSMYALEFTAGVTEAFIIATT<br>CTLFCNLCTIVSRELKLLRLALRRTRSRANYTFKMRGYSFLPGSNYAKYKLFQQCMVHCIEECIKHHIALKKF<br>QEEFQGFMGFPIFAIFSGTALTISSPMFMLLTMTEAEDSFLVLMKILQYSSIIFSFTCFLSSYCLFGQMITNESSLVH<br>FAFYETPWIDGDLDFRRKVIMGMIHSRKPFILTAKGLAAASSETLVDISKTIFSCFNLLAATQ
AlinOR32	MDVLHYWDLTGFKFFPIYDHMMRSIGVHSERGEKSAVAIKRYFGNFLIILAIIQGWASTVAALDSLKEEDYKA<br>VTNVMSYMSITFSCLSKVQIARSHMSVMHRLGVWIIQVKKNRPKDMKQPLLEYLVLKANPSFFYFGLFAAVF

	WVWVPIVTLTFQAFIPTKFPYLDKYTSNSISFPVMQLPLYVFFTVAITYTATSLLHFLAVFTTEVKLLSEKWAQVVYDKRRPHAYMESMKTCVQQHIKLLDVMKDLNIIHDSMFAFQVMIFIVHFVSFNFCLVMTSGSNAISSVFPLFSSSMIEFGLLCWMGEEITDALQQFHRSIYMTNWYEASLSDKKNMIVMLEFLKKRHVLTGTKVFVASLDTYVEAAKQAFSAYTLMKALTE
AlinOR31	MDEALKLANSTVLVISGDKKLMLFSIQKAFLNILGFDWFPGGVNLPLPAFVKHTLYRLLYPVLMIVVAILILVYVFTHEVVTENDFIIKINGYIVSMTFFAASFKVLIFIFQKNMFKELFLMVEMVGAIDTDAPSTKSHIYNCYIYITLVLMNPGTWAAWWVIVHNDTPFRAQYPWGNEGFGYMLSYFVGVNGAVFCGLCHILVDTSFMMVVAGITLHVDMLSASLSYLGKNRFKDNKILSAAIDKHAELLRVSQHLSQCYSNLFVAQSVYTVGHSCVLLFGAVHVASQVEVVMNQGTMLATSYSQLLVYCYYGELLTTKFSDLVFDSYNNKWYDCELSVQKALPNLTLMSMRHVSLRGFGNVHPSKSNWLHSLQESVSYFLFLKTISGEK
AlinOR30	MTKTDLSDIIQLLQFTGHYFTFEGRRRTSFETLQKLRVIFMVACNPFTLSSLFIGGLKKSMGVELFFGLMGFLTAMQHVYAFNHRKDTEDIIQAILRVRRKYQKGSDAEFKQDTRMIWKVVYIYFSAMTALMVFYITLPKILDVIYGIIWDDPIALRLPQSMDAFLEEHQHRNLKYVIVSLISSVWSIVSTYSHFGLDTFLCLIGFYYSSLVKTFCNSLKLDTSLSSEQLTIQIKTFAAHHHELYKLSLRMRSIFGCPYAMQNTFGAFCIVSLVYAILSDDSGGPLILLANIFNLMILAGMLTSTAYVGQHVTNESSAVFDALYGLPWDELSPTNRKYFVTMICAAREPFTIHFHGRAPLNLTNFMAILNTSYSYFMFMRSTL
AlinOR29	MDSDQFRYVREVYRESLGLTGMDVFLDEKPPDHRVIRWHVLRKFFLLFVFVYYPIFISTQIWGIAAGDSNTLKQISFDISLLGHNIQNFIKMGIWITRIQTVRSLCLDFPKFHINNYRPNLASWILEKESKDARNFTGRCYWISYVNLLFWVVLPTSTAGVIYLAYLAGYKEERDTYIPRYSPVRFPVDMTLRSSRLLVAGIEMFQFYFGFLLFQPIDMFFTAIIQMAHAQIRVLNSALFSLDGELQEYWGIQVPEGDPKQPMEVRLIIEDHQKIVKYGQRLREFLNPILGFESFNCITVICNMTIVAASEFSAEGEFLDLALPAFSSILVVFTSLTCFYTFTKMTAVLKDAEESIFHALYASNWYEKDVNYRKSIILMQKLTHTPRRIKMCGIGDMGRSTFIDGSRMVYTYYNFMQRFK
AlinOR28	MVIEVRNMEQGLKEISGQYYAASGISGIGAYFKKDKPFFLIRLWVQASTWIILPQVTLAHIYTALFTGIPLATRFLSLSLGLDQLQTTIKCHYMLHNLDRFRSIMLQLETFNCVNHLGFEAITTMLKMCKLVKWLRSTYNFACYFTFIAWTLVPVVSSPKALFYGEDGESMMKVLPPEYPFATDYFPMIQFIYGLESLSTFVILTYFASTNLIMVTDILLICQLFRVLNDSVAPNKNRNAMSLRMFAVDHQKLLKICAEVRDLLSPLLALQLGISVMTITLAVFEITMVNQASSDGGINQVVLMSRKTSYTFIIFVELLLYCWLSTELELSCLSVRNGIYNSEWYERLGTKDYRNFIMISMRALRPVKLNAMKIATLSLGTSLEVLRMAYSYYTYLKRLH
AlinOR27	MEVLHPNIVIFLKLMKSTFYWYDEDPPTSLWDRCRRIYQYSRVVLFVLLFIQNVVGLYNTDGFGIAEGSFLHFPVCIQLVVINGMVFYSRKALIKLTTTLNKHFIESNEPWMIAISNKYTVPLWKMIKIMKVYHFYANIAFFLSPFIADTILHYGFDALEKPFYMPTPTTAWMATNATWDLQYYSVVFLGLWSMQEVMAVVMGFIYNYSILLIFALIQLTIMNEKVKAMKLDGTQKEVDAEFKAIVDSHNDIIRLNADLKSFLGVQCAFQSLFSSFTITLCLFTATQRPEFVVRTSFAWGAVFYASTTFIYCSLGQLLENKSSELFYALYDLPWYRCSPKVRKDLNMIMRQSHNSLLVDYHGHFKMNFESYMQIIQQAYSYFTLLNSMAG
AlinOR26	MEDSWLIRYFSAGTGRQEYERIQAVAIKEFTPLVVFVGVFLPTDRTVLLCIFGFTSILVYSFYTTIFTYTCIVATDDFVLWSEIIHHTSLMYLGVFIRTVFMLKAQDMFILTQDYVDGVYNYEEGYVDPIFQELKDKSRALQRKLIMLPLFIVAVTGMAIGLKPYLDYVNEVEPHPDLMKNGVNFNSLVPVVYPFENANTYQVLVMNCVLLYFALLVILTVIAADILFIRVSCRISLEIAILVESLNLIGKRARRLYARKYGLKPPSKKNEDWPLYQDCISTCLKENIVHHQNIIKFYESFSAIAAPAIGGGFFTCTIVLGLGMIVVNMDNVNISDQIAFTGTVFAEMMNAFMISWMSEKIGEQNYELYNAVYGLKWFKWRRDNKKLVITILDGTREPLFLNAFGLAKINMEAFGSVVNTAYSFLNLVNASETLEEKK
AlinOR24	MTDIKNNSRAYYGIDVLYFKFIGFWQLLTEGVWANKITIFSIVYGLLFSAYVIMQFSIMFVQEYDFSIFTEKLSVNLTCLESVIKMLFYCLKRTSLLELLAIFRLDLLLSSKHSPSITDKILTSNISTVNGATKSFVIMIFTTVGIWNCMPLFKRYSSDESTMLQIMPSWYPIDVSYAPVNIIVYIFEFFVMIYCAALLYNVNCTFSALALTASAQFELLSVNMANIESNAENTIDKESIVDMNDEIKKDIMNILLRECLIDHQTLLRLLKQMEDVFNPIFLFQMLTSTFTICLVLFQLNFHL

	ASGNDLPIVVTFKFIMYLVFGSMELLVYSWGGQMIYNKSEEIYWSLQKCGWHKGSHEFQTNVMIAMIRSQYP<br>ITLTAGKFYAVNLASFAQVIKASYSYFTFLHGSISEK
AlinOR23	MYFLAIPAFIGLGTGIIMILPYATKWINGMDEPYIVGMVNENLPFPCWYPFPTHEGVTHWVVVLLQCGAAGS<br>LAFIALTLLLMLLENSQRIKYEYRVLGYSLEIILKRSMKLYLQMNPWKKSASVNVQEPEFQRMIELCLIDSVIH<br>HHKILEYVSLFGQQVSLLGFLTYSVGTGVIALSLFNIIDAINSEDISSIILFSIFIISEVLVQFAFCVLGEAITSESVAL<br>RNKLYCSKWHYFDRRNRKIVLNFHTAMTEPVVITAMGLINVSLETFATIMNSSYSFFNIVNST
AlinOR22	MSFFLDYCDKLEKKRDATVKKFIRDNYSILLQAGTIDMNLSRKYVFWSCLLAIFNYAVLANQDWMLLYSTFL<br>LKDDFETASGDLNYFLLISVSIGFLCNFHYYREDFLDGCKIMSGGLFQYADHMKETEDMIKFRKHMRFQRNL<br>LIALAIYVCNIGGIVVLGPVIDDYTGHGFNGTYDENGVNRRLPVPLFLPFESINGIGYLTALGMTTISGTVTCAV<br>IGGASFLFSTFSHQILTELKILSCSIKAISSRALKMYCRIHNVSKKSVDRKTLYSNPMFQDCITECIKENIKHYTNI<br>AECMEVVERFVKIPVFLSFLIVTLAIGLSMMKLNEDIVRIGSSVSFASVAIGEILNMLSVAVNGEHFLTLSHEVN<br>WEIYFTPWYKFNLKNKKMIRQFLQSTQNELYLSAWIVRFDMEMFASVVNSAYSFFNFLKLSKTINVEEM
AlinOR21	MAFGYLRIDWLTQDEVDVFDLFQKLMSNVGTMSDSKEKRRLTIIILSLLFIPMVIASLCASIIYRDDFDILAYSLH<br>HTVLMSLAWVLNNVVIPVFRNQYNFLMEGTKKTYYYDSDLVNNYAKDIIHKRIGISRFIGKSIVIGTIGILIEIQI<br>FFAAEVIWFQTYRTLFPVYTFGLDLDNIVVLVSVVLYQEVVMCWTANLPVVLLNIAYSTWSHLDLEMKILVFA<br>ITNVEKIVMEKSGKRSLDRTDEKHRSEIYESYCCHLAKHHHCIISYFEAYSQAVSLITTLMFTTGFIMFVLVGLST<br>MSDNLGVKLKLFWFLVFQVIITFGWCWVGQYIADKSAEISEAVMGTPWWLMPKSCHSTLLLIMVRCKKPLVI<br>TSSFGQQANMQTFMDMMKSVFQFVSVLYQVTKGENEE
AlinOR20	MNKENEKKRTSFSCVDYRKYMYCKLIWIDDGLAARGLTRPLLFIMVWMMGMVSFGLISFVMSTQNKARLD<br>NLRSFLLESMVTMSIFNEYMSRKSLAQLHQFMDESMRTSRTELREEEEILETAKSQARKHLAAYIVIFSFNLAA<br>MILSQPLAEWLQGNSWKKLPYPWVVPPSNTEFMFWIVFLYQSIGLYFSHCLGMVIMSFSSITIQVTALFDVLLL<br>SLRHIEARAKVRMEREGTDYITSITDCLKDDVVHHQRLVSELVSATPHLRRTFFALSVTISMIMACEAYPLIMG<br>NFTLGELIKGLLFLVVQFMCWGQMCTRMEIMADQNSEVFHALYNTPWYSSGLKYKKLMVTPLTFSRHSMYI<br>KSPLFTEMSATMSTFYSFVVSSFNILNLIRKMK
AlinOR19	MGWLRSSKKYLTKMRDLEDTDVRRVVNENYSIIPRLSNMVSSIDEGYIPLNIIHSIIFNVLVYLYLYLFVVTCYLL<br>RDDFVLVGVQFHYLLLALFGSVFQFHMYTSRLSFLQAHKIIALDFYTYEMDLLVDEKAKFKEYMLKQRRQLIP<br>FMFLIGVIGMFIVGFGPLIDNMVGAGHDGDYLNGVYMKTPIPMYFPFEIDDAVSHYAATGFQIVTVVMLALSI<br>SGVVFMYVVTTQNLALQFRVLIASLNKLKERSKARFDKLYPNDKINPKNLSNDDKFQQCIAFCLRENIKHHQ<br>VMIKYYLIHARLIGVPILSAFFMGTFIIALSMIILVEKTDRYGLLMTNTLAMIGEVGNLFIACYFGEEILTLVSR
AlinOR18	MNKKIEGDSASAQEAKVERHLQEQFSVLTVIGGVYGFYRSPRWSIISYSMYITMYTCSFLISADLLYSSIVLRNNI<br>SLFTVAFHAFLINCVVLTGSVSLTLQRRRTTEFLYNFDFTGYWTEYQESKFFVTLEAQSRRRILQLLILFLSGYCA<br>CGMIGVVGPFVDMRLGIDENVTNVTGIYWKGLPFAMWWPFDAHKSTISWISCFMCQGLWASFAPLMCTSAI<br>ILCFNSCEKLLNHMKLLIYAIEHLDLRAKNIFKKKFKYVTPDKQGIEYDDCYYECIVQNVKHHQKIVKAIDDF<br>MILANYAIAVPFFGGGLLLGLAGLNILSRDDPRVGPKLFCASLGMTEALNMLLLCVYGEKFQHEGEKLFTSIM<br>YTKWYTRSLKCRKALMILQCGTLRPVKITAAKLIVLNMATFANLTNSAYSIFNLNSVVSSGE
AlinOR17	MSIANECDRRVGLRGNRIERMYAEVTEIEKTSPQTEGMRLICIIGCIICSWTCVEPFVNAWAGGKELEFPFTGT<br>NENSKLFVAIYLLQCSLMFITANFCIVIFQTLMGTALNLVIKYKVIGMELARLNEKMVNDKDVYKTDLYQSIR<br>RCVQSHHHILRIFERYREVCTYGFRYSYVGLMGATTLSRTLLSGDEPDLGTIPHLIAELSYIGFFCYILNHLEEQ<br>NDNLKDAVYAGDWAWMPKKATSALRLIMLRTTKRPHVILVKGGGPANLETFYKLLNGTCGYIIFGLVLDQA<br>AF
AlinOR16	MGFVLDGSLVCKLRQPVFVFLLYSAPIHHLIPAFTDDTATSDQILMAWSISMLYVLLCIAWPVMIIRSSEIFGLW<br>DTVRKGFFHYSDPFTSAERMILSKTDDIIIKSTRMSIIAYFCAGFGTFLKEMQPQSLHNYKPPYPGWFPWTINSN<br>FRFALALLYQCGICLNTTFALEAIFVLFAYHTIHFEGQIRLLTRHYEDTFQPRLPASVTYAAEFKRRTLRRLKECV<br>RHHLVITGFHKQILSYFGICLLVYRVIVTIMLCILCYLATTGIALNKFVQLLCFALALLYLCFIFCLKGEKVTQMS

	DEWRHTVYEVDWWNHPVEVQKTILMMQLGSSKALSVYGIWKPAMYSHEGICVIAQETFSFFNMLRAMK
AlinOR15	MSGDIEQIKSNSLTRDWYIKKGRDENNGFFLIIGGMYIGYRWISVLHAVLTAWHMPVLLLAVYYGREDFVVVS<br>ETIHFIILLLLAFLISMTYLSFRETLDRVFEAMGNGYYDYDGTLDTKTEKMIQQLQFESDRRKTILKYMFIGGCI<br>GAMICVSGIRPILQYYLQKYIKIKKLPDGVNGVKNTFIYYPWDPANLWLNLIGYTLQDVYTIITANVIFGFVLIF<br>VSTAESLSVQMEKLKLSLKRVKIRAAYVISKKPKDSDLSTDRDFSKALHICLRHSIKHHQVVIRIFDDFKSINHT<br>ALLWLVGGLTFLLCMSSVLFTADDVSLISKATFVFFISSELLATFLMCWYGEHLGGMSYGLPGDLYDTEWYEFS<br>GDLLIYHRMLAMRSSKPCQLTAGGFSKIDRNTFLEVLKTAFSYANLLQASKQN
AlinOR14	MAKKSKEVFETDPQRFSTNKELLQALGLLWSKTDWNPLRSIFSLSLITSGMFHYSLRVAFTYTSMELLTIIKILH<br>LTNVYFLAGLCAYVVFLQSRRFHIIHSIIKDGFYNYPEGLTQEQMMIRSKANHKVSKITKYGLRFFVISTLGSCL<br>KEPVPYDFEEWTKEYNGWIPFVVDSWPKYFASQFYHLLAALSAALLGGSVCIVFIAVAEHLLAQLEILSISFRN<br>AIGCIPTRGDKVAEKLAYQRMKYCLQHHNVILRFFDEFQKYYSIPLFCMLAGTTVAMCTIAFVVTDPSSTFGV<br>SAAFLSLMAPEVAFCICYCTYGQKITDMGDVLRETVYNAPWYYQPRPVKMAMLMVLIKTRKPLTLSAAGLK<br>DCSIKSIGEITQTTYTYFNALQIFRGNPTSNK
AlinOR13	MVLVPFFIKPRIGRNAAIDRGYKLTSMFYARLAGLYPDLEAGWRYWFFGTYLNSVYVAYAYYVLAYVIANIIAV<br>KYKDFELIGSTFCFGSYTYVYSLITIMFLIKRKKIDRLLEIVGNDVYKYRRPPTKEETLIKETETMKVIVYGRYTFF<br>IPCSVALMQMAVVPAYHGLRGEYTSIVNGSTPINKYSPLPVWTPVEATTGLSFFVLYWCQLCPGFVEFLIFHGS<br>CTFYIGGSCALVSNLKLLHHSLGRLVDRAEYLYDIKNGKYDHQSKIPLNTELFDECMVECLKENVQHHIEIIKF<br>HHLFQDIVGYSNLFIFSGAAVTISTPPFTIIKIAELEDRHQLLCAGIVMIGHAFLSLFLLAQYCKYGQSIEDESEKI<br>LESYYFTPWFKARKSFRQLLVVAMSNSLNPLQIKSAVLGISASAATYMSIIKSAYTMLNFLVTAK
AlinOR12	MTLFSFAKKKLKWDEPLGLTTITATLTGSFVRIAPPPSYRRFCYFLSCYQIIMSALFMSIAATKAVTATDFFAESIE<br>ALRFLVTGAHILAKHLTMMVREKEFLELIDQIRTAWVTYQPSNSDLLSKTLSSSNKYTVIIFSALQFTLITSVLGA<br>YAKNTNDLDDIQFPLQVWVPASLRTSFVAGTIFQIIPYWAPLVIYCTTISFLNSITRHVEALGLALARDIRRQKK<br>WKTNRNNTLYKKHQEIVRIVHRVNALMASNWGFEMMCSTLQLTLVTYNFLRCLKRNDVEFVNQAFLVLVN<br>FGVIYLIYGNGNRIIQMSEDLHESLCASDWHEASVKERKNLLIMMFKTINPLEYKYKIIHFDLPGFAKVVNTVF<br>SYITILRSVDEMEDKEGS
AlinOR11	MKRSNMAGAGKDEYVIKDTKVFATCRRALVLMGFIDDGSLISKIRKATFVFLLYTAPFHHLIPAFVDDTISLDG<br>ILISVSLLMLYILLCVSWPFMITRSDDILHLWETVRKGFYHYSDPLTKEERTILSETDDLVIKTTRISIIAYFCAGFG<br>TYLKEMSPASMREYRPPYPGWMPFVINSNFRFILTLFYQLCICLNTTFALEAIFLLFAYHVFHFECQLRLLTQHF<br>KDTFPEGLSATVTYGAKYRKNTLRRLKECVRHHLVIKRFHEQILSYFGICLLVYRVIVTIMLCILCYLVTTGISVN<br>KFMQLLCLAMALLFLCFIFCIKGEQVAQMGDGWRKTVYEVEWWNHPVEVQKTIFMMQLGASKALRVYGV<br>WKPVMYSHEGISVIGQEAFSFFNMLRAMK
AlinOR10	MAFSTWIHAQLVTEEQFQMRLRKYGWLHYLFQFSLVNSSYRTVPTLFIYYLYLLFTSSVVVFHLFCYIKTALNA<br>YSIGRADMSVANVHSIILGIFILSVLSSYVVDKTTIVDIEELYLESLFDYDTVIPNSATLFEVAMTFCGKLGIVLGG<br>MGLFTNVYLAAPLMDLRFWKESCVIEGINFCLALPHYYPYDSEDGWKFHATEIFQVLFGVYRISVFCAVQVTL<br>TLWPLHLVRELSKLKASLEGLEERIKKRYYQKTKINLDKVNLITMNKDKVFNECASFCINENIQHHHNILRYH<br>GTIDAIMAIPSFLAYTTGTATMAIAMVKLLSVEGDTTLGGNIAYVTVLGAEIGFMILISVMGEAVTMKAEEIFD<br>EVAHIRIENYDLDFRRKVIIFMEGTIQPIALSSSKFNKCNMEAFGNVLNAAYSFYNVTSASAALDK
AlinOR9	MEHADKHAFERMLRREFWILTVIGGTYGFFHEKAWAVVSTINYVVLYSIMALTMSILAYTVYLLQGQLGYLSQ<br>ALNIFIVGMVVVTATLTLTLSRPKMQAFLAFYDDPWSFCEYSRNEYFENLMLHTVKKKNKLILTWAFLYGLC<br>GAVGVCQPVIDKIFGRSSEITNVNGAWLNLPIIFWWPFDPTESTLIWMVPFMLQSLFLGYSAIVVASAVSLCFA<br>TADLVMDQFKLVIYGINNLDNRAKEMYKKRFPGSDMKRMDNREYDDCYYDCLVQNVKHHVDTLKWMDK<br>FNDMASLPVAVPFFGGAVLIGMALITITEEDDPRLGPKCLAAMSAFSELYNMYLLCKLGQEWQQLSDDLLDA<br>LYGCRWSGRSERVKKAIRIMRLSCARPMKFTAAKLLVLNMQLFSDLINSAYSIFNLKAVSQEKES
AlinOR8	MSLRTNAADGKNHLLDLRDVEGLTMGLNTFGIKTFWHILDYFHTTGKRHWLMTTYIVMYHLIGGTYCLLG

	FAAVFFIDQEDIPRMAAAIMNPLIATQAIFKCWTFSYSTAEYLKLFVLLKKNFMSCVPPEKKLAVDEVTKKNIG<br>LTNQFVKYAMRWNCFTLSMVSFMPYLRSQAFREFFHLGVGPIVPNKVFENEYPFEWNSSPTYEIIWFYEQICA<br>FLAVVTSSAYQAILLYFVMAIVGHLKVLGFVMGNMKATDFTSDSNETMDETAKAKSYKQLVLCIRDHGKINE<br>AGDLLAERYNTFLTFHIGIAIVVGIIAIFNCTVATELADKIKFGIMCVYGLLEVAIYCFCGQLLENACDDVLRQ<br>VYSCEWERMDPKFRKAAQLMMVRANSPICLRAGRLYRVNLETLEAIQQLVYTSLTMLTSMVQ
AlinOR6	MTDEHLAGVSRIYRDALGFSQLDVFLDAKPPQNGRFSWHIKRIVAQFFVCFLAPSFISLQICGVLTAESQNLK<br>QLSFDLGFLSHNVQNFVKMTYWLTHLKSVRSLCIDVSTFNVNKYRPILSSWVLKKETDVTRKFMNRCFLISYG<br>NLIFWVALPTIVSICNYFRYVAGVTEGQDSYIPRLSPTRFPVDMSSLRNRLLVGFFEYGLITMGFVYFQPIDMFFS<br>SIVNMVRTQFFILNSSLFEMPADLEEFWGSRIPVQDTQPPMDLRLFVEDHQRLVRYGVQLRKFLNPVLGMVT<br>VDCFNIMCSLLIVITEILEGDMNFTALLELISGLLVILSSLVVFYTYTSTSGMLKEAEESVFEALYAHKWYGKND<br>EHKKNVIFMQIRTESANKIPMFHIGDVGRDTFIEGLRMCYTYYNFLKQFK
AlinOR5	MPPNQLLNTSKHEHDVYKRLDKLYYYGMRLLLLGITPHKFFGKCYFKGAILYVTVILLYILYGLGELLWAIIIPG<br>GILERLSHAYVCSYCASYGVIWVYLMVKLETIHQNRIDFKSFNCSRLLGNTEVESILQKNINYFVKTLFAASLL<br>AGANVTSYILGFVVELIVQYAETGTLEEICVLSCFPFPLWGQMIIAAANLVTLFMCFSIIMSMYIITGLLSLEIETQ<br>CEILTRTMAYDEVNDDFKTFVIDHIRLIKQTKWVVRLFENINNSLFFSSYVCLAMQMFSLSVIKPEGYYYLGVF<br>SDFCVEFIVMASQCWLSSAVTNSVLSISEGVYNTPWYRKNKSNAIDVILMTQMAQRPYIQRVFLGTMKIEKET<br>MIQVIQQSYSFYALLMILQSKK
AlinOR4	MGYVSSKFKSSVQEWHSWEDEYSVEAMRLRYRGFHRIGFLVLDLSPKYALLSVIMCVIAAAVLFIVSFCLTFSC<br>YQMSDDFEDCSGVCNLGFLCVLAFSFLLNHNFYRKKILDLHHMLGKGFHDYQEPQYFPDELEKFKKVVTKQ<br>NVALIILASYVALIGFLVVVVCPLIDESLGFGWTEPYDENGVNRQLPVPIWLPYPSHEGFLHWFSFLFLEGFGG<br>AMICLSIGGTALLFTCLSGGLMLEQKLLVLSIKSIEKRAKRRYRELHKGKPGIDEDGNKIALNDDNKYQECIGY<br>CLRQNILHHHKILTYTNHYLDLARSPLLFAFLVETMAIAMSMVKLNEGSNKWGANIAFACIAIAEVANMIML<br>CVLGELVTSGSIEINDELYYTKWYTFNKSNKKVLLQFLLETRNPVVLAALGLVVCNMDQFSSVMHTAYSFFN<br>MVKLSKLREETVTMGANT
AlinOR3	MTLKSYIKETLKWDEPLGLITTIAVVAGAFNTIAPPKRIRRFIYWLSWYQTISYILFLMSAGTNIFTSTDFFDECL<br>ESLHFLVTAFHVFMKYLTLRFRERDFLELFDHIKRVWSSYRIHNEHFLTSKLSSVNITSVLIFTSIFNVVFVNVGA<br>AYLKNILDPTKVHLPIQIWIPSFTKSSFLVGTTIQVVLFTWPLFIVAMSTTFLNSISSHVEALGLALAEDIGREKV<br>WSRDVARDFYKKHQDVISIVLRVNALMAGNWGFEMICASVQLTLPAYRTLRAFRMNDVEVFNHAVILCLN<br>MMVIYMIFSSGNRILSMGEKIHTKVYESNWFELPVKERKNVLFMLFRTTVPVEYRYKIIHFDLPGFTKVVNTVF<br>SYMALLRFLDSGGSEDEGALM
AlinOR2	MTVDELTLHDMVGFPLWIQNVLYMKITGHWVGAVPGPTPLRVNILRAVGGFPVFLVLLYVAGANINGMVH<br>NSDMTDISMNLIVLSTTVSALHKYSVFTNQQQALGRLGRWVKSVAAERKANNVPDTYADRVLKKALKAFYI<br>SGNVASPILIVKMMLTGNTFNVNPGVENFPKPFLMFLIAVSFQAIAWEAVVDGCILMNSLFVFRSELVRFALE<br>WEKLNFDPHNPEISRRQLKAMVKKHVMLLGVKKDLKEYNNSMFGYQVFAAVFTTCALIYGCAKDTKFLGQ<br>AVIQVLPTSTASLLTFSILCWSGEEVTYLFQQIHRNIYMTNWFEAPREDKKSIIVILEFAKNPIIFTGFTVFTCTLT<br>TFVETMKQSFSLYTILKAVL
AlinOR1	MSINDYKPGEIFQNNLKPMKKLRPLKKVSYQQPDKRGTYKKVAKRLSEEEALKKGFNDNQGLYLVLGTLYR<br>DSFGSWVHTIVFIIACLFMLFCLGRQTLLITDDLSLLFETIHYITIIGGVLVIVPPMMKNQFRFQKIFKIFAREVYC<br>YDYLDEETAQEILRLRAEGNKEKQLLTKAFTVMLLGTFAGFSVLLPGMYIINGQFFAPQREDGVIMGIPCVIW<br>FPFRVDDKWVVTVRILLLALEEYASFTVVAFIIGQQTTAICIGHTLLYEFKVLALTMNKFEQRAKLMDKKFIGD<br>GTLKPASQTRKYITSCLNESIKHHDVLLDVSEQYSSIFYVPELVILLSSTMVICLSAVSLTSDNIPLEAKAVSVIFT<br>GAEMMNVFVNCYYGQILLDAHNIIGDAMYESNWTSYSSIVHQHVLIILSRVQKPLSLTAGGFAAVNLDTFAQ<br>VVKSSFSYFSLLQALKE
AlinORCO	MQKVKMHGLVGDLWPNIRLMQLTGHWLLEYHEETGGMARLIRIAYCWMTTFVVYLQYAFLVCFLILETYNS

	DEMAAVTITTLFFLHSVTKFTYFAIRSKYFYRTLSAWNQVNSHPLFAESNARHRAAALSRMRKLLMIIGVVTIL<br>AVFGWTTVTFLDDPVWDKTDPDNVNETISVEIPQLMVYAWYPWDAKTGMTYFMTFALQLYWLFITLAHSN<br>LLDVLFCCFVIFSCEQLKHLKEILQPLMELSAALDSVVPNSGDLFKSGSAGSNIALISNGDGGNDFDVRGIYSS<br>QRDFSGFQGGMTNGTTVGPNGLTKRQELLVRSAIKYWVERHRHVVKFVTSIGDTYGTALLLHMLTSTVTLTL<br>LAYQATKIEGVDVYASTTIGYLVYTLGQVFVFCIHGNELIEESSSVMEAAYSCHWYDGSEEAKTFVQIVCQQC<br>QKSLTVSGAKFFTVSLDLFASVFGAVVTYFMVLVQLK
MperORco	MQCQPLFTRRFVLRLQILNLRNLHNIIQRIGHMAMGYKKDGLIKDLWPNIRLIQLSGLFISEYYDDYSGLAVL<br>LRKIYSWITTIIIYSQFIFIVMFMVTKSNDSDQLAAGVVTTLFFTHSMIKFMYFSTGTKSFYRTLSCWNNTSPHPL<br>FTESHSRFHAKSLSRMRQLLIIVSIVTIFTTISWTTITFFGESVWKVPNPETFNQTMYVPVPRLMLHSWYPWDAS<br>HGLGYIVAFVLQFYWIFITLSHSNLMELLFSSFLVHACEQLQHLKEILNPLIELSATLDSSVHNPAEIFRATSAK<br>NQAINGIDRDYNGSYVNEITEYGTKGENESNRKGPNNLTSNQEVLVRSAIKYWVERHKHVVKYVSLITDCYG<br>SALLFHMLVSTVILTILAYQATKINGVNVFAFSTIGYLMYSFAQIFMFCIHGNELIEESSSVMEAAYGCHWYDGS<br>EEAKTFVQIVCQQCQKPLIVSGAKFFNVSLDLFASVLGAVVTYFMVLVQLKc
MperOR2	MQKSERFILTPFQKFCIRWSVLFDSSSDRLSRTETVLRSVQFSTIMITSGMTMTSVLIADNKKALESFTYFVICVF<br>MLAIITLAIRTKRFNRTMLLMVEDEFPSYNRPMPDALKRKITDIRTSYGDFTMKVIVSYLTLVLFEIPATGMVPL<br>AAATLTEVKLGSQSTQMVVLWFPADTSQVGMYALSYVIQFLIVVTVKFIITGIMCSFSFFVNQMISEFQILSAYIE<br>HAVEIVEFDQSVDKTTEQKLLEHVKSCTMLHDRLIHFKDQLNESYGYIILLELMFSTLYFCLSAFNMIFVGNRF<br>VMVKGLLTLSNYLAELFIFCMYGSMVEDAHMGLLRASYSAQWYAQPVRFRQSLMMVMSRTQTPLQLTVGK<br>VFIANLPLFLSVLKVSYSGVNALRAANAK
MperOR3	MKTSENITARKMSVYWLTLLLLNGINVYPGHGNRVGRIATAAYPWMICAFWAFITTSVTTSLALRATNYEEV<br>VEMLTYITGSSCTLALFAIGVHKRPRLHRMLDAVRRDFWDDGRRPAADVLFSRFVRTYGAILPVANVMMCM<br>TPVIWAARNGDIESPAALIFRMWTPWTRMTTTRYAAVYAAQFVVSLSVLTSIAGMVFAMVLFVTEMQVQVD<br>TLVDAVQDLQVDMWYGDGHDSPVTHPDRRRRAAFDGLVKCVKHHQALITYFNHFKSYFNLLFIVDILYIMV<br>MTCLCASSVLMANGFSAFHIKMMSLLIIVVSQFFFYCLIGEQFSTMNQQIGDCVYFKLVKCKDPKLSRAGLLII<br>LRTQKPLQLTAMGITKYSASLFTFTVTMRSAYAGLNVLYNSS
MperOR4	MTLLIEKKNYQRKFYQILMTVAFFLDTNKYRYISSFIMQFYIFDWMVLVFLASAYNIWDEKSEMLMVMELIQY<br>MIVGVYFSLIFVVFITKKKDIVSNYDCIQTDFIQWSNKRALNPNVAYKKNIKTIKRLVIPLATLSLSIAFGPLVSAI<br>NDIGKLPLDNRAHFVLFWPKIVDTNKVSMYGIIYSLQVIFTVILYISVLSFNLGFMVFLNELTNQFEMLLDGIN<br>DAFKYKMDKQFQTLFIDCIRHHQIIIKFLDDLKSYFKWMILIEMIVVQVVLAILIYNLTKVHASLGYKVKIAGS<br>LLFNLLPICFHCHIGEVVLGLHKRLSNHIYNMTWYDMPNKNKQLIVIMFQRTQRDLTLSSALFSSERASRSLIS<br>KVIKQVYTILNVLLKT
MperOR5	MPHIDTINMFLQMTGCTDNKKMLYLTYFEFLITFYYLIAAYISILHFEESVTIQLFTLLCMLIECVILLNIVFRLYH<br>QNHIREMLQYSRRSGIPDSYQSIINLITNYHLIASNMFVIFPATYTILHDSVRVGDPFTFPFLDVLPIQTGNLAIY<br>ACKYMVYAISVYIAHIELCFINTTFIYYVGVLKQRLDTIVQTIQEAVVDDDEQKFKYAIIKHQKLLTYFNTMKIV<br>FAKPILLSMSFNAIYFGLTTSFVIQAIRGYINQAILSICIASSAAAVINITIYTFYGSVLLDLQDEILHVLFDNAYFY<br>VNKSFKSSILIMMTRVTIPLNFTVGYIFTINLNLLLKIVKMSYTVLNVLLSSETIKPHKLS
MperOR9	MEHIVDIFLRKMGCSDDRSYDTMCVIYFTYCELAVTLFFTISTYLSIVYSTEDLSMRLYGLLCFLIEIHIFGFIAAR<br>FYHQSQFRDMYHRSQEVGIPENYRQKIAMVIKHYFIMSNVFVAVSVLYTISLDWVQMGDPFTFPFVDVLPIKTI<br>NLSVYVCKYIVYALPVYFAHLEICFLNVTFMYSTGVVKRHFQILDEQVEEAILNSEEQQLKIAIKHHQEVLKFF<br>EDMKTIYEKPILMTIEFCGLYVGLTSCFVIQVIQGFIHQIILGLCIVSSVACIMTIIIYCIYASNMYDLHNGILNSLF<br>EHRSCYSRNKSFKHLILMMMTRASIPLEFKVGSIFTVNLNLLVKILKFAYTVFNVLLTSINRQFK
MperOR10	MAHTVADLFLRNMGWSEKHGYYTMCMVFFTYSELALTLLFIISTCLSIVYSRENLSMHLHGLLWLLVEIHVFA<br>VTANRLYHKSKLRDMHQRSQKVRIPENYRRTIANVLTYHMSLPNVLVAIPALYMILLDGVQMGDPFTFPFVD<br>VLPIKTTSVTVYVCKYIVYAMPVYIGQLETCFLNVSFMYYTGVVKRHFQILEEQVAEAMVNKDEQKLKIAIKS

	HQEVLKYFKDMKTVSEIPILVNIEFCSFYVCLTCCYVIQAMQGFINQMILGIIIHTSIAGITTITIYCIYASNMYDLHNGILNALFEHRSCYSRNKSFTQLILLMKTKATILLEFKAGFIFTLNLNLLVKIFRFAYTVFNVLLTSTNRQFKESAI
MperOR17	MTTTGATTFAPASEDLTIVDNKLFKAICLHQILDPTKGRNRYYRSALLAVMWLSLSMQITQLVGLYFAVNDLQRFAFTTTTVTNAFQCLSKGYIIMTHADRLRASLETARYDFTSCGARDQRIVRRSRNVLSTVLRTFIVLSWVTCFIWALTPLFGMDEYLQVTNADGTFTRYRVTIYNVWLPVPATVYNATAVWALVYSAEVIVCFVNVFSWLLFDSYVVTMCFTFNAQFRTVSASCATIGHGDCSGSPSPHATGTHNIIRDDNNILNCYDELINHIKDNQSIIKKCDDFFEIIKPAILFQIIGGSYSVITLIFLTLLTYLMGFSIISIPVLKVFFGFLSVTFELFMYCYVFNHIETEKCKMNFGMYSCNWTAMDLKFKKTLLFAMNNNSAHRRVMKVTPKSIINLEMFSNVMNMSYSIVSVLLNSRVQK
MperOR20	MRSSSATAVDVMLFKTIGLYQLLCPADRGGYSVRFRRLLMTALGLSFALHSFQVPCLYYALNDLQRFAYMAAVIIYGMMCSFKGYVLVTNADRLWLVLDAAGYAFTGCGHRDPSKLRRCRATLSALLRTFVALSYGTLVVWIALPFFVDEYTGVTNLDGTVTRYRTTIHNMQFPVPLTVYNSRPFWALIYFTEVFVCIVNVFIWSLFDCYLVTMCFVLNAQFHTMSAGYCTLGSRRRRESSQPNTSRTGVRRIKFDDMESNHYVDLIGHIQDNQKLIKVFDVFFEVVWPVVLVQIANGSYSVISLIFLTALMYLIGVPVLSAPFFKFVCGLISLTIELFIFCYGFNHIETAKSILNFGLYNSNWTEMDLTFKKTMLLAMKMNSSHKRAMKVSPNSAVGLEMFARVMNMSYSIVSVLLNSRS
MperOR21	MENPERVDYDSNGVTGNPAEKAEGGEMDNPPKNDTEGGTILDVELFKIIGVYQLLRPDEFGLNARLCRTTAIVVVCLTLGLQSMQVCRLYLARHDLQMFANVGVMIINGLMCLLKGYMVAANADRMSATLNAARYAFTGCGNRDQSKLRLCRARLSTILRTFVRLSFGTLIVWVVMPWFMASEYDDTPSIWATVYVIESIILTVNVFCWTSFDCYLVTMCFVLEALFCTMSTGYETLGRHRAAAKSSAGQQLAIAGTISDVSISDVNYDDLTSHILDNQNIIEQYDEFFDVVRPMVLVQIANGMYSIITLIFLTLLTYLSGYSIVSAPFLKFVCGLASLTIELYIYCYGFNHIEDGKSTVNFGLYSSNWTEMDLKFKNTLLMAMIMNSAHKRVMKVSPNSIVNLEMFTGVMNMSYSIVSVLLN
MperOR22	MRRSKIASSAAPTDNGGDEATKNQEISSDEGDYNCKDSGTIVDVDLFKAIGVYQLLHPVECGLDSGLCRMAVKIIVGLTLGLQSIQVCRLYLARYDIPMFANMGVLVVYGLMCLFKGYTLATHADRICTTLEVARYSFTSCGGRYPSLMRQCRARLSTILRTFVGLSFGTLFVWLIIPWFLTSEYDDKPIVWAVVYVVESIIFTVNVFCWTSFDCYLVTMCFVFEAVFRTMSYGYENVGRVHRLYPHAVQPFLDYRSKDSDIQSDVPLKFPDHYDDLINHIKDNQKIVEKYETFFDVVQPVVLLQIADGSYSVITLIFLISISYLNGDSIISPAILKFFCGLASVIIELFIFCYGFNHIEVGRSTVNFGLYCCDWTEKDLKFKKTVLLAMSMNSAHKQVMKLSPNSIVNLEMFARVMNMSYTIVSTLLS
MperOR23	MNFNDEKNYIFNLRLMKITGFYQLIYPSAPKCFGFNAYKVAAAIEVMTGVLSVSFLFSSSYYYLDNTNELMSHFMLVVAIFFSTFKIFWVSRNSKTIWNNLDMTSINFLSYTGHKKEILQNARAKSISTTILFVILWSSVTVAWCISPFFVKDVYLNVKFKDDEIRRFRYNSLNYVYPISGESYNEHFLYFYVVEMLQVVFWGHGTVAYDTFVISICISIAFQLKTIAVSYISLNDRKGDVKNLKDNDLEAIFNLKLLIQDQQNMFKKIKEIYKIFQPVTYVQLAAQSMLIILQAYMIFINHYNGFSLLSVPIIKLVVTVAPNIIHLFITCYLYSDINYQKDSMNFALYSSDWTAMSISYKKMLLFTMRMNDAEKLKLKISLRKIVNLEMFASVMHLTYSIISVLAKSYGNTNTK
MperOR24	MGLQNEHSIINLQYMKITGFYQLLIPSDGVKLFNINIYKTAFIVQILILSVTTIMGFYSIYAFINDVNQIFNYTIIIFAANFAIFKYYFIIKNAKTIWNFMHNMMSTNFLCYKGHTKEIYKIGRNRSSTLILISFGLWSNIVLYWSLSAILSKNSYFKLKFKDGVYNYRSNAIDLVYPVTDTFYNKYFLVFYLLETILLIFWSQMMWVFDILMISVCISIEHQLKTIAASYSLIGLNHNELTRNNKSTKNVEAILDLEVLIQDQQNILEKTKNMYQILKPATFIQLAAESFQIILQPCMILKLYFDGSLSPTLFFKLSFPEITYLCHLFLTCYLFSIVNEQKESMNFALYSSNWTAMSVKFKKLLLFTMRMNDAENLKMQISIKRMVNMEMFADVRITNIMIQVILL
MperOR25	MVIGTIIVSQNEDNLMINMRLMKKTGFYQLLDSRSLKVFGHNVFKCMSVVQISILLSIAVIFVLNIYYFSDDINTVMMYSMLITSDVLSILKLYYILQNSDTIWNCIQLTSIDDLSYKYHDRRILEEGRLKSKSYSLLIIFMWMYLIISWGMAPLFVTNYFLTVEVNNQIHRYRFNVLNYVFPATDQFYNDNFVFYYCVELLTLVLWGHCTMNFDILLLSMIITFKYQLKTVANSFKSISDYKSLIYDHQRVIENMRNIYRVFRPVVLTQLASESVIIMLLSCITMMNYFNGMSLLSAMNLRLFAAISTFTFHIYVICYLFDDVNEQKDSINLALYSSDWTPSNLQHKILLLHAMRMNNAENLRIQVTKSR

	IVNFKMFTDIMRTTYSILSVLEKMCANKT
MperOR29	MVLEKEPKLMANSKLLKTIGLYQILNSSSPHPKVYGYNSFKCIAVIEIFIATATIISCILNAFYCLNDINEATRYFSI GVMCAIIAFKFCYIIGYSDTIWNCLRITSIEYLTYKYHSRRMLEVGRKRSKFFLILFIVLWIAVYLNWMLIPIVVQ NSYVRVEAEHLIYHYRTNIMSLVYPVTDKFYNENFIMFYSIECIVMIVGIHSTFMFDTLLISTCITITCYLKTIANS FSTLGNVENHFMTRYDETKILNDFKIIIQDQQKVIENMKNIYKVIRPVILFQIAAGSSVIILLSIITIMNYFNGFSL ASPMNLNLLSTILTFTLEIYFICYLLNGVNEQKDSLNFALYSSDWLDKSLKYKKKILCAMRMNNANQLKLQV TLTRIVNLELFTGVMRTTYSVISVLSDQLAKQT
MperOR35	MDKLRVEEFSINLELMKRFRFYHIFNPNVTTIFNFNAYRLLLFLCGSIMIMIIVYSTLGFFVEMDDTLSYIDFFVV IFVMIVLFLCYWRICIFLYNADAIHDLFSISRIDFLNSKHCCKNVNVLYDNRDKSIKISNYFFLLSTTVMSQWIIF PLVVIAFTKPEDENTRFQNILNLRYPVSTHTFNQYYYIFYLIEVAVAVFTMYVMIMPDILLMSFCWVIMAQQEV LIRAFKNIGYEENSQIVYYEDFKSILGDQLQLNLKIKLFYSVVRSIILTYVAIISTTFIMVTYVLILVCLSKESHPVL NIIKLGSSAIYMCLLLFLYCYLFDSMNIKRQSINSGIYSCDWTKMDVSFKKLLLLTMQMNNANNLVIKASPTKI VDLQLFANIITMSYNIVSVMLKAVETTS
MperOR36	MEDFRDEEVVINLKLLKQYRFYHMLKFNETKILNCNVYRLILFLYGSIMTCMVVYGSIVLFVEMDDIISEADLF IVIFLTINFFFCVWRICTVLSKSNTICDLINVSRFNYLTSKHCCKHLNVLYDYRERTIKITNYFFVFSMIVLMQWII FPILAITFKKSDFENIRSENVMNFRFPVSTHTYNQYFFIFYIMEVAIVTFPIYLIIVMDTLVLSFCCVIIAQQEVLSL AFRNIGHEENSQLEYYEDFKSVLGDQIQLNLKIKSYYSLMRNIILVQVAMSSTFFIMVAYVLIVVCFSKDSNQIL TIIKLGSSVIFIGSEIFLYCYLFGSMNLKRESVNFSLYSCDWTKMDNKFKKLLLLTMRMNNANNLMIKASPKKV VDLQMFANVISIAYNVISVMLKSMDSNN
MperOR37	MKWLQDHEVAINLALFKRYQFYHIFDPNGSKLLSYDTYKLTNVMFIVAVTTYNIFSAMCFFTDTVNSIDSVDL LLMIFIYSIIVISLLKITVLLYNADQIWELFDLARLDFLTSKRCRKNIGILIKYRDTSITITNLYQNYSTIVFIIWMVV PLILNTFVLVEGPNQRYHNIFNMQYPVSASIYNRYYYLFYLMEIAMGIFVLNYSMIIDNFLISLCWAIIAQYEVIT TAFENIGNDCELENLQNRRENKSFEAYEDLKSIIMDQEKVYIKLKSFYHVVWIIVIFLIIIDSVLLIILTYSFVMICS SAESFSIFNILKISTAFFVFVIQLYLYCYLFDVLNDKKESVNFGIYSCDWTKMDIKFKKLLLLTMKLNNADKLKI KATPNKIVNLQLFSSVMTTTFNIVTVMLKTMKGKN
MperOR38	MNSLESNEVAINLKLFKLFRFYHIFYPYSEKLCNFNVYHLAWYIINCVIGGIIIYGLLGHFTEMEDDIDIIFYIQIL FCYLLYSLSLLKIITFLYKANDIWDLLSVTRINFLTSTQCQAHIGILHKHRSKSIQITNVISGFGIMTTLEWILFPLV LWLLPKTNANGSNQRFENIFNFRFPVTISEYNNNYFIFYIMESSIAVFMLYVYVVIDVFFISVCYVIIAQYEIIKRA YESVNCEQSPKNNNENKNNCNLNDCYDDLISILRDQQKHYAKLKLFYSTYKLIIVSTVVINSGSIIILTYASVVI FISSETIPIFSVAKLISAFAYMFFVLFFLCYLMERVNNKIESVQLGMYSCNWTAMSIKTKKLLLLSMRMHNANK LMIKTTPNKIINLQLFNSVIFDSIL
MperOR39	MNLENLFNGGPVALNLSMYKQLGYYQLLDPKGPHIYGYHLYRTILKIFLLIVQFIAIFGVMGFFIKIEDTDTDP GKSNSFELIIILTNCSLSSLKMYTLVSNSKIIWDLFDLTRIDFLRCSRHSKLIKANFMKRCKKSTTITKWIARSFLV GLILWVMGPFIANEEYTEPNVVRRHQNIINIKFPVTVKTYNNYYFVFYFMEVAVGFCIVYGSVLIDAFLMSFC WIISAQYQSVTKAFATFGYNKQGSPKEIYKDFKSIIIDHQNVYLKMKSFYAVVRPITLIHVFAYSCSLIMYAYVIV TIFHSKESFIIAEIMKIVMTVSNVTIEVFIFCYLFELIDNKKEDVNFGLYSCNWTGMDIKFKQLLLMSMKMNNA NRLKLKATPDVTINRPFFANV
MperOR40	MNPSEKNYAFDLILFKTIGYYQMVDPNTKKIFGFNIYNVINITLVIFTSIMTLIGLSGFLYKVDSIAYEENSFQNIQ MLFYLSCISLGNLKIAITVYNADAIWKLLNVAHESFISNKYCKQDKYKLNISGKQFVRIFPWYFFLFFMTAVSW SIVPIVVNNQVESKETQNNENIYMTNVANLRYPITVKTYNTYYKSFYALEFILVFYCAYGLVVFDLFIVALLQLL ATHYEIISSAYENFKYKAENKNGKLRNTEIQKELISIILDCQTIYKKLETLYGISRPIVLVYMVGDAIGMITMPFLI VMSYVQSGSSILNTNVFAFSWTLFVVGIQSYMYCSLLQNVNEKKEDVNFGLYCCDWTSLDIEIKKLILLAMRV NSSNNLTMKVTYTKFIDLPMFATIVRSSYSVTSVLINSNIHKISK
MperOR41	MNKQKIYDVNFTLFKLIGVYQMVDPNTQKIFGFNVYHFVNIVFITFTTIMTILGLSGFFYKVQNNNYNTSEVD

	IIFIIFSTVCITIGNLKIIIIIFKARQLWNMMEITDKSFLSNTFYRRNYHKILKCGDLLSKFFNLYFSFVLITLTSYAIV<br>PIVLNARSIDATQNTETIQKMNIINLRYPFTTEIYNTFFKIFYASECIILFYTGFGVFALDLFSMTLLMVISYQYKL<br>LASAFEVLEYRMDNKDDSILSDEKLLETFISIVSDSQIIHKKLKMLYDIIRPIGLIQLMADALGMICMPYLIVVYF<br>VKYGSLFNPETLKFVFTLGIAGVQSYMYCSLFQRVNDRRDGVNFGLYCCDWPGMNIHMKKMILFTMQMNS<br>SNKLNMNITTHKAINLPLLSTIIRLSYRISSVLINSNIN
MperOR42	MLNYSKDEDCIMSSLMAKCTGLHYVIDPKSPKLGGHNVFHLTIMAMIAFTVACVSLCPFGLYHWANDMTQ<br>CLIQLMIIGNFSFGCFKAYMIVRHSDDIRRSLDVTRFDFVSSAIADPDSARFFRRCRNVSATFTGWFAAINHFVL<br>MLWTLLPFVIDSNKVEVKNRDGSFSYYHFNPYNLYFLVSSETYNEWHLVFHLIEWMFGLIFILVMILFDTWMV<br>TLCIAITCQMKGIADAYRKLGHCRSRTAPNAWFGDEIESADSSNNEYVRDLKLVIKHHQAVLGKMNDFYKIV<br>GPVILPQLIVASFTIIFVSFIITRNYFNGMLLTSTISLKMCCCPTFFFQIYYTCLVFGNIDHQKNVMNFALYSCDW<br>THMEIKFKKLLLLAMEMHDAYKVDMKLTAEMIVNLELFTSVINLCYSIFSVLVNSQLKIVDRL
MperOR43	MDSKQEKQYIFNMKLARIICLYQILIPNSTSIFGYNIYHIVIVIFGSFMFAISMLFPIGLLYLRNDIIAIMYYMGCIS<br>NFLLSSFKMGNILYHAKDIWKCIDVTSFNYISYKHYDRNVFKNWQTRSIRITYIYIVIALSAFFCWIFSPSVMNK<br>SIITIRNIDGSYSKYRMNIFNIYLIASHETYNKYFYIFYAIEIIISICYVYFTIVFDVLMLLLCFAISYQLETISNTIRSL<br>GHEICTRDNFRTLHEKHGIIYNDLITIMTDHQNVLKKLNDFYNIFRSITLTQIFIASSSHVFIWFIAAMSIDEGD<br>NADSILSFKLFIVLPLINFQLFMTCSLFGTIHEKKDSIIFALYSSNWTDMDLKSKKMILFNLTLNNANQLKMKY<br>TNTKIVNLEMFSHTMRFCYSIFSMLINYNKNKMK
MperOR44	MFTTEFSYLSLIAFECLMSAMMCLNGLYYCINNIDESVLYFGFVVNMLYSSYKMYIVLNQSKLIWDCLTITQFD<br>FTSYGLQYRRTLDVWRNRAIKSSNIFAIVNAVVCICFVACPLVFSNTFTIMKNHDGSTSAYRLNVLNLYLFASE<br>DTYNTHFNVFYIIEAFGTVIFVLFMVIFDIVVSTLGFALCGQLQIISNAFESVGHESHHSPNNNIENKIKLRNEH<br>IIINNISVYEDLKTIIIDHQSILKKYDEFLSIFRPTMLLQSFYEEDFTESMAVITLKAGFAIPFCIYQMYISCHMFETL<br>HIKKDSIIFALYSCNWTEMDMKCKKLILLTMRMNNAHHQKLQYTRTRIINMEIFYHTMRVCYTIINVLQNCK<br>KAKLF
MperOR45	MSILQINNNRIFNLQVMATLFGFRQILDTETVTLPGRHNVYHGTLLVFLIVYECIISAMMCLNGLYYCIYNMA<br>ESILYFCYAVNILYASYKMYIVIKHSKLLRDCLSTTQFDFTSYGLRGRRTLDVARNRSIKSTYIFVMISFLVFICYAA<br>CPLVFRDTLIVMRNHDGSTSSYRLNVLNLYLFVSEQAYDAYFNVFYIIETFGSSILFFFVINYYTAVKTLGFTLSSQ<br>LRMISTALKSVGHKSFHSPNIDNINNKIELHNKNNMDVYNELKKIIIDHQNILKKYNEFLSIFRPSMLQEVFVL<br>SYSIIVLWFIFLKSFIERDFTESLGTMAVTSMKASFSIPYCIYQMYMYCYIFDTLHNEKDSIIFGLYSCNWTEMDM<br>KCKKLILLTMRMNNAHQPKLQYTRTRIINMEIFYQTMRVCYTIVNVLINWKKE
MperOR47	MDIRNENNHVFNIGLAKLSGLYQMLDPGTVKFRGQNVYQIFVGFFVLFSFVGAMTLIAGSLYYWTDNTSVTI<br>WYFWSTTNSLYACYKMCTVFYRSNDIWNCLSITRYGFTSLSSRKRNGHDILDRWRARSVWYTSLLSGAYCWS<br>LVFYAGCLLAFGDATIPIKNHDGSIGNYRPNVLNLYFIASDETYNEYYNTFFVVETLFVASITIIYLLFDVLLLTL<br>CLAICCQMQIICSAFESVNRKSLSDSHSNAVDNTHEKQNVSNEHDLIYYDELIKIIIDHQAVIKNFVLFSTTFER<br>VMLSHIFVSSISLIIIWFNLIMSFSDDGKFEISGATTVKTIVAIPSFSFQIFMTCYLFENLHNQKDSIIFALYSSNWTE<br>MDMKSKKLILIAMQLNNANQKKLRFTRTRIVNLEMFFKTMGHCYTVVSVMIHYINAKND
MperOR51	MDIRNEKNYVFNIKLAKFTGLYQILDPGTVKCQGRNIYHIVIAFFLVYMFAISMILNFSGLYYWTVNIPISIDYF<br>WKSETTLYVIYKIWIVVHHSNDIWNCLSITRYCFTSSSNRNRHIMILDRWQKRSVSLTALFAIMYSISTITYMVIT<br>LAFSEDISPVKNHDGSVGYYRQNLMNFYLIVTDETYNAHYYMFYFAEALYLIFFAMSFLIFDILLVTLCFGMHC<br>QLELICCAFESVGHRSISDSNSPIDYTDEYNKIPNEHDIIYDELKTVIMDHQAVMEKYEKFITLFRRVMLSQIFVS<br>SLSVIMLWFIFIMSFSSDDRFQASDVVIKKMFCSIPSLLFQIFMVCYLFGNLHSQQDSIIFALYSSNWTEMDMKC<br>KKLILLTMKLNNAYYKKLKFTRTKIVNLEMFFKTMGDCYSIISVLVNYIQRKVE
MperOR53	MDIWDQKNHVFNVRLAKVTGLFQILDPRTTKFRGRNLYHIVMAIIMLYVCVISVILAISSLYYWPYSIIVSVDY<br>GWKGLMTLFLVHKMWNVVYHSNGIWNCLSITRYDFTSHSLRNRHVLDSWRDRSVRITTIMTVAYSTSSIVFA<br>ASSLIFRDDIMTVKNPNGSVGNYRQNLFNLYLIVTDQTYNAHYDTFYIVEVLYTVFLSILFFMFDFILVTLCLAV

	TCQMQMVNVSFESAGHKSVYDPTIDNTDEKICLSNEHDLIYDELISIIMDHQAVMKKYGELLTLFKPLMVLQ<br>VFWSSLSLIMVWFSFIMSFFGQDRFVASEVTTIKLICLIPSISFQIFLVCSLFTNLHNQKDSIIFALYSSNWTEMNIK<br>CRKLILLTMRMNNTNQKKLKFTGTKIVNLELFYKIMSHCYSVVSVLINCIKAKNE
MperOR64	MDVRNEKNYVFNIKLAKFIGLYRILDPGTVKCRGRNVYHIIMACILVYMFAISMILNLNGLYYWTVNIPISIDY<br>FWKSETTLYVIYKIWIVVRHSNDIWNCLSITRYCFTSSSNQNRHIILDRWRERSVSLTTIYAIMYSMSTITYMVIT<br>LAFSEDVSPVKNHDGSVGYYRHNIMNFYLIVSDETYNTHFYIFYIAEALYLIFLTISFLIFDILLVTLCFGMGCQL<br>QLICCASESIGHKKLSDSNSPIDYTDEYNKIPNEHDIIYDDLKTVIMDHQAVMEKYEKFILLFRRVMLLQIVVSS<br>LSVITLWFIFIMSFSNDDRFKASEVVIKKMFCSIPPLLFQIFMVCYLFGNLHNQQDSIIFALYSSNWTEMDMKCK<br>KLISLTMKLNNAYYKKLKFTRTKIVNLEMFFKTMGDCYSIISVLVNYIERKVE
MperOR67	MQNNNPDYLSFSYNILRSIGVFSASDSTKWTRWAFNFYRVIIFIVITLVTTLMMVQMFVATDLTLLARTIDIWT<br>MFSTGLYKWFYMTMFRGEFSQLKTALTQIQAQGSVAYGSSADAFTADYLKQTRKISFWYMFSGMVASFFIIVS<br>PLLTYSKGDQSDFQYYNDPKSYPLSCWIPFTLNENWMFLAVFVCYSVALVLIVIVYLGIDTYLFVAIYAIGGQIE<br>LLNTSLNNNENTLEQFENSSCTNRLSIYTEKQQILFYSTLRECVKHHILILNYITNIRKLFSTLILMDYLHGITSV<br>TFALFQLTISASVIETISVVCFICLSIWHQYLNNFFGEFIIQKQLSVCTVVYDVPWWRCGERVRQLLTLMILRSIK<br>PTFITGFYMYKLSYESFISFIKALYTYYMVLRRVNVED
MperOR69	MDIRNEKNNIFNLKLANLTGLYQMIDPGTAQFRGRNIYHIGMVCVLLYVCPITIILILSGLYYWTVNIPVSIDFL<br>WKSLITLFIIYKGWFVVRYSDDIWNSLSITRVEFSSLGNRNTHIVNHWRERFVWLSTIFLRINFMVLFSVAIGPL<br>AFSGYVQIENHDGSIGYYRECVANLYVTVSDEMYNAHYYTFYFIELLFFVLNLAIYFMFDILLFLVCLGMCCQ<br>MEIICSAFELVGHKPHRDTHSPIDNQEENKKITLNEHNILIYDELKKIIIQHQAIMEKYEDFLTRYRPVMLLQIF<br>VSSFLAIMLWITFIMKDSIVFALYSSNWTEMDMKCKKLILLAMKLNNANYKKLKFTRTKIVNLEMFFKPLVIG<br>KSKKPRCFKNMDISSLPVIWKFNKKAWMTTEIMEQWLRYFNADMRSQNRNVLIFLDNAACHPKIELSNTKI<br>LMLPPNTTSITQPMDQGVIYTFKSYYRKFLLQSLLCKMDNCSSAHQLAKSISVLDAVNWIALACDNSHECM<br>MDMDYGYGLSSLAIINIEMHQTLEIVED
MperOR78	MDIQNEKNQVLNIKLTKLIGLYQILDPGTLKCRGRNVYHIVLAFIVVYMFLISMMFNISGAYYWTDNKLMSV<br>DYFWKAENTLFLIYKMCIIVYHSDDIWDVCMSITRYDFTSFSHRDRHILDRWRERSVWLTTMFTIMYSSTTVFY<br>LGISLAFRNYTLPVKNHDGSIGFYCQNVMNFYFIASDETYNTHYFTFYFAEALFVSLSFMSYVVISTLLITLCFA<br>MCCQMQMIYNAFESVGQKLICDPHSPIDNTDKKINVPNEHDLVYVELKTIVMDHQAVMEKYEHFLNMFRL<br>VILSQIFVSSVSIITLWFLFIMSFSNDDKYKDSDSVFKKCVFITLGILCEIFILCYLFGKLHDQKDSIIFGLYSSNWTE<br>MDMKCKKLILLTMKMNNANQKKLKFTRTKIVNLEMFFKTMSDCYSIISVLINCIYRNVK
AgosOR22	IGMYQLLYPAECGLNDGGHGYRTAVLAAMGLVLGLQSMQVCRLYLARHDIQMFANMGMLVVYGFMCLLK<br>GHTTATNASRICVTLDAARYAFTGCGGRDPSVMRRCRATLSTILRTFVYDDMPTVWAVVYVVESIIFTVNVFL<br>WTSFDCYLVTMCFVFDMCFVFDALFRTMSAGYEKLG
AgosOR2	PGDTTQQQPESFVLTPFQKFCIRWSVFFDSTSDRLSRIETVLRSVQLSTIMITSVLTMTSVLIADNKKALESFTYF<br>VICVFLLAIITFAIRTKRFNRAMLLMVVDEFPGYERPMPDDLKRKISAIRKSYGEFTMKVMVSYLTLVLFEIPAT<br>AMVPLTAARLTDVKLGSQSTQMVVLWFPGDTTQFLIVMIVKFIITGIMCSFSFFVSQMISEFQILSAYVEHAVEI<br>VEYDLSTGKTTDQKLLDHTTDQKLLDHVKSCVMLHHRLIDFKDQLNESYGYIILLELMFSTLYFCLSAFNMIF<br>VGNRFVIAKGLLTLSNYLAELFIFCMYGSMVEEAHMGLLRASYSAAWYSQPVRFRRSLMMVMSRTQTPLQLT<br>IGKVFIANLPLFLSVLKVSYSGVNALRAANAK
AgosOR38	SLLEPNEVSINLKLFKFIRFFHIFDPNIRKICNINVYHLAFHIINCVIGCIVIYGLLGYFTEMEDVFNIINQIQLMF<br>CILIYYSSLLKIITFLYKANNIWDLLLVSKINFLTSTQCKTHIDILHKYRNKSIKITNMISTIGVVTTLEWIMYPLLL<br>QLLQKEDANKSNQRFENIFNFRFPVTINYYNNNYAIFYFMESFITMYMLYIYVAVDVFFISACYIMIAHYEMIK<br>RAYENINIELISENNNKNKNYCNDCIDDLVSIMMDQQKHFAKLKLFYSTYKFIILSTVIINSGSIIILTYASVVIFM<br>SSETISILSVIKLISAFGYVFIVLFFLCYLIDRINNKMESVHFGMYSCNWTAMNLRSKKMLLLSMQLNNANKL<br>MIKITPKKIINLQFYNSVILKFIYIYFFII

AgosOR42	MLNLSDNGDCIVSSVLAKCTGLYYIINPKSIKLGGHNVFHIAIMVMITFTSVCLLLCPIGLYYWVNDVTQFIIQL<br>IVLGNFSFGCFKAFTIVHYSDDIRRCLDVTRFDFLLRLPRHGSDSVRFFKRCRNVSLKFTSWFAISSHSVLLVWT<br>LLPFVVVGNPVVIKNRDNSTSNYHFNPYNMYFLVSNETYNEWHLIFHLLEWMFGLCFVMFMVLFDTFMVS<br>MCIAITYQMRCVAAAYRRLGHNRRTTMKVYFDGAIESDNTNNECLKDLKIVIKHHQEVLGKMNDFYKIVRP<br>VILPQLIIASFTIIFVSFIITRNYFNGMLLTSTQSLKMCSFPIFFFQIYYTCLVFENLNNQKTAMNFALYSSNWTQM<br>EIQFKKLLLLAMQMHDANKLNIKLTAELIINLELFTKVMNLCYSIFSVLVNSQLKITDKL
AgosOR8	MELKSLDGTFTSFFYIFYALVGLCIGCYGIFTSFSTNCKFLSNIDYFLIFYITTQTYLSYWKLFKCLKDRNRFLDLF<br>KIGQLNFLTTKECAKYNKVLYQHHDKNLKFANYSFIFSFLIIMVFIFPLVINEIIHFENSNVRAENIVNFCFGVST<br>STFNEYYLIFYLLEITVTSLTVYILIMMDILIIFLCSAIIYHQEVLIYAFKNIGYEDNLTISKTMKIKLFYSIMKSTILL<br>TVGIDSFYLIFFTYLFILVFLTLIKIGSTVLYITARLFIYCYLFDSINKKRELVNYSIYCCNWTKMDLKFKKLLLLTL<br>QMNDANRMGIKASPKKIINLQLFAGVIIKF
AgosOR11	ISIYYSQCDFTAVVRYIMFIYATFFVIIKISFLIIKSDILWNFISFTSINFLSYSGHQKYFLMNARIISLIISNVFAILWV<br>AFIALWIFSPIVINDSYLNIKSKNSTYMQYRYNTLNLLFPVSTQFYNDNFTIFYLFETIILIIYGYSMIVFDCLIISFC<br>LTIAFQLRTIASSYSTLGYNHTNNQIKSFINNIIHIVNTINSNNGISLTSTESIKLLSAEIVNTGHLFSACYLFSLIDI<br>YNDTINFALYNCNWTEMNINFKKLLLFTMQMNNANNFKLNISTNIIVNLKLFTNVIHFTYKIISILKSVVN
AgosOR40	GKLNKKEIQKEFVSIILDCQTVVFTLEKIFSQLKTIYGIARPIILIYMVGDSIGMITMPFLIVMFYTQDKSIFNSNVI<br>AFSWTLLVVGIQLYMYCSLLQNVNERRENINFGLYSCDWTRLDIEIKKLILLAMRMNNSNNLKINVTFTKFID<br>LPMFASIIRSSYSVTSVLINSNIHKINK
AgosOR44	MKIHDNHKRNFNLALAKFIGFYQVVDTEKVTFLGRTSRSVLVIFVLLIVYECLIAAMMLLNGLYYSKNNITESI<br>LYTGFVINMFYASYKMYIVLTRSKDIWDCLSITQYDFTLYDHRDRRIILDLWRNRSIWLTSTFMIFSFVMVTFYV<br>TCPLAFNNTFIVMKNRDGSTSTYRMNVLNLYLFIPEEAYNNTYFNVFYIIEASGTYVLVLFVIIFDTIVMTLCLA<br>LSCQLHMNFAAFESVGHTSVVILKTNYFCLDNNDNIDNKIKLPNEYINGIAMYNNLKTIITDHQNVLKKYDE<br>FLSIFKPIMLLEIFVLSYAIIVLWIIFLTSFIVGEFTESMGVTSMQTGFAIPFCVIQLFMSCFVFDILHNKKDSMTFSL<br>YSCNWTELFDMKCKKLVFLTMGMNDAHHQKLQYTRTRIINLEMFYQVCFTLSS
AgosOR36	MDSFNGQNILINFKLFKQLQFYQIFHSSGLKIFGWSIHQLFYIVFGLVGLCIQCYGLSTSFFNNCKNISEIDSFLIV<br>FASTYIYLSLWKLFKCLKDRKIFLDLFKIAQLDFLTTKECTKYSKLLYKQREKNLKFANNFLIFSFVICLQWFIFP<br>LVINEIINFENLNIRAQNIMNLCFGVSTQTYNKYYFIFYLLETIPISISVFIIIFMDTLIISISLAIIYQQDLLIYAFKNI<br>GYEDNPTINYYNTFISILQDRLRLNLKIKLFYSIIKSTFLLSIGIESFFLILLTYLLILVYLTPKSDAVLTLIKIGSSAIY<br>MSARLFIYCYLFDSINIKRELVNYSIYYCCNWTKMDLKFKKLLLLTMLINDANRMVIKASPKKIINLQLFASVII
AgosOR27	ISFTSINFLSYNGHQKYFLLNARIISLIISNVFAILWVAFIALWIFSPIVINDSYLNIKSKNSTYMQYRYNTLNLLFP<br>VSTQFYNDNFTIFYLFETIILIIYGYSMIVFDCLIISFCLTIAFQLRTIASSYSTLGYNHTNNQIKKNQLINEKPMDL<br>SNLIVIIQDHQKLTKKIHDIFDEMRPIILFQLLSESILMSLIPLVLFLNSNNGISLTSTESIKLLSAEIVNTGHLFSAC<br>YLFSLIDIYNDTINFALYNCNWTEMNINFKKLLLFTMQMNNANNFKLNISTNIIVNLKLFTNVIHFTYKIISILK<br>SVVN
AgosOR14	TKIFGWNIHQLFYIVFGLVGLCIQCIGLSTSFFNNCKNISKIDYFLMVYASSQMYQSYWKIFKCLKDRNRLLDL<br>FKLAQLDFLKSEECAKYSKVLYKHHDKNLKFSNYFLIISFVVIIQWFVFPLVINEILNFENSNVRAQNIINFCFG<br>VSTQTYNKYIPIFYLLETTGAALTVYFLTMIDTLIISVCSAIIYQQNVLIYAFKNIGYENKSTINYYKTFISILQDQL<br>RLNLKIKLFYSIMKSTMLLNIVIDSFFSMIFTYLFILV
AgosOR18	NNNTLNCYDELVNHLQDNQRIIKKYDEFFEVIKPVILFQIIGGSYTVITLTFLTSLVSVLVFPRKIFHIYINTFLNV<br>YLFLFLLRRILWGFQLYPYLAVNFGLYSSNWTAMDLKFKKTLLLAMNMNSAHRRVMKVIPRSIINLELFAKV
AgosOR31	MAILNDHTAAIHLKILKQCGFYQIFDSNSKKIFGWNIYRLSFISLTIITQCLIGFGNCGFFFELEDAINYIDLFLIIF<br>SGSFNYLILYKVIILILNRKNILDLLDVTCLKFLKSKQCYNNIEILYKHRNRTLQLTQLYFNFCILVIIQWVFCPIII<br>NSFIVDKNDNRRLENVINRRYPITVNTYNQYYVLFYIIETIIAIKSLYLILMIDILLLSIGWAITVQYEVLAVAFKN<br>LGHDVNFQKGETTLFKKIYKKVKLYFSIVKPIVLMHVAISSGLFIMLSNSFIMIILSKESFTILIVNLFKIGVGIFYIC

	LQLFLYCHLFDNINLKREFVNLGVYSCNWTKMNLIFKKLLLLTMQMNNANQITMKATTNKIVNLQLFSNV<br>NIKFFVLNKSYNIISVMVKAISK
AgosOR9	MSIAVMVHYMAEMFFKKAICSDDDLHRREAMRMVFFTYGELAITLFFAVSTYLSIVHSTEDLSVHLYGVLCLII<br>QLLVFAFLSFRSYHRSHFRDMYQRSRGMEISENSNRKIAAVIKHHLIMPNVVHIGDPFTFPFMDVLPIETTSVA<br>VYVCKYVVVYALPVYLTQIEVCFLNVTYIVIFRLLEKQVEEAMVNKDEHKLKIAIKHHQELLKFFKEMKTVYEK<br>PIFLIIVSCGLYIGLTHDRILNALFQHQLLYSQNKSFKQLILIMMTRATIPLEFKAGSIFTVNMNLLVRILKFAYTV<br>FNVLITSINHQLIKTAV
AgosOR33	MSSFEVSNVAINIKLYKLLRFYHLFDPNNIFGYHFYRFTGIFITVFIQLFVLFGLLGCFMEMEDTINDIEQFIFIFV<br>NLSNFLSVMKLCVFTYKAKNTWDLFDVTCIHFLKSEKCCKYRNEILEKVRNKSIKLTNFIFYRKLKLYYSIIWY<br>VILTYVVLSSCSIITLTYSFIMVITCISSTKSLPVLSIIKIIAPFSIVSFQIFLHCYFFGLINFKKASVSYGMYSCNWTS<br>MDLKFKKLLLLSMQMNDADKLMIKATPTRIINLELFVKVDNNLLLSEAIYK
AgosOR10	MSSFEVSNVAINIKLYKLLRFYHLFDPNNIFGYHFYRFTGIFITVFIQLFVLFGLLGCFMEMEDTINDIEQFIFIFV<br>NLSNFLSVMKLCVFTYKAKNTWDLFDVTCIHFLKSEKCCKYRNEILEKVRNKSIKLTNFIFYRKLKLYYSIIWY<br>VILTYVVLSSCSIITLTYSFIMVITCISSTKSLPVLSIIKIIAPFSIVSFQIFLHCYFFGLINFKKASVSYGMYSCNWTS<br>MDLKFKKLLLLSMQMNDADKLMIKATPTRIINLELFVKVDNNLLLSEAIYK
AgosOR39	MNSENIFNGRPVALNLSTYKQLGYYQLLDPKGPYLYGCHFYRTILKIFLLVIEDSDANNGKSNSFELIIIILTNCT<br>LSSLKIYTLISNTKIIWDLFDLTCIDFLKCSRHSELITENFVTRCKKSTEITKWIARSFLIGLILWVMGPFIANEEYT<br>APNASHRYQNIINIKFPVTVKTYNNYYLVFYLMEVAVGFCIVYGSILVDAFLMSFCWIISAQYQSVTKAFATFG<br>HKNELSSPEEIYKDFKSIIIDHQNVYLKMKSFYAVVRPITLIHVFAYSCSLIMYAYVIVTIFHSKESFIIAEIMKIVM<br>TVSNVTIEVFIFCYLFELIDNKKEDVNFGLYSCNWTGMDIKFKRLLLMSMKMNNANRLKLKATPDVTINRPFF<br>ANV
AgosOR6	MDVREENKHVFNIWLAKRVGLYQMFDPGTARYRGKNVYHIALTFIVLYLGVIATMMNVSGVYYWKDNMPI<br>SIDYFWKAETWLFVFFKMWIVVYRSTDIWDCLSITRYGFTSFGYRNTRTLDRWRERSVRFTTAVTVIYLTSLVFY<br>IAGSLAFREDVILVKNHDGSVGYYHQNVMNFYFVVSDSTYNAHYNTFFFVEAATAVLLTMLFLIFDILLITMC<br>FATCCQMQLVGCEFESFGHDKPLGDDPRRSPIGEHEFSDYTDERKNVFKERVSMYYDELKTIVLDHQAKYEN<br>LLSLFELAMLLQIFVSSITLIILWFIFIMSFSNDDRFIVSDIIVKKMIFLIPSLSYQIYMECYLFGLLHNQKDSVIFAL<br>YSSNWTEMCMRCKKLILLTMEMNNANHIKLKFTRTKIVNLEMFFKTMSDCYTITSVLINHIKTKNK
AgosOR20	MAHSSSTTVVDVSLFKTIGLHQLLCPVNRGGYSVRSRRALMAHSSSTTVVDVSLFKTIGLHQLLCPVNRGGYS<br>VRSRRALMTALGLSFALHAFQVPWLYCALNDLQRFAYMAAVIIYGMMCAFKGYVLVTNSDRLWWVLDAA<br>GYGYTGCGGRDPSALRRCRVTLSALLRSFVALSYATLIVWIVLPFFVDEYTPITNLDGTVTRYRTTIHNMQFPV<br>PLSVYNSRPVWTLIYVTEVSVCIVNVFIWSIFDCYLVTMCFVLNAQFRTMSTGYVTLGRRRVKPLPQDTPVKG<br>VRIKFNDVKSNHYDDLIGHIEDNRKLIKAFDVFFDVVRPVVLVQIGNGSYSVISLIFLTSLMYLMGVPVLSAPF<br>LKFICGVISLTLELFIFCYGFNHIETAKSNINFGLYSSNWTEMDLKFKKTLLLAMKMNSSHKRVMKVSPKSSVG<br>LEMFARVMNMSYSIVSVLLNSRS
AgosOR21	MYQLLRPVECGLDVGRCRSAALAVVFMTLGLQSMQVARLYLARHDFQMFANMGVLVVNGLMCLLKGYM<br>VVANADRMCSTLDATRYAFTGCGGRDPSVLRRCRATLSTILRTFVALSFGVWAVVYVVESTILTVNVFCWTSF<br>DCMDQWYTKHKLRLFRTMSTGYETLGRSRSGDVKLSARQQFDTVAAGAAISKTSITDDNLDDLKSHIIDNK<br>NIIEQYDAFFDVVRPMVLIQIADGSYSIITLIFLTSLVYLKGYSIVSAPILKFVCGLASLTIELYIYCYGFNHIEDGKS<br>TVNFGLYSSNWTEMGF
AgosOR32	MATFNEHTAAIGLKVLKQFGFYQMFESNTKKIFGWNVYQFSYIILLMINQCLIVFGNSGFLFELDDTINNINLL<br>LIIFSNSFNYLTVYKVIILILNKSKIQQVLDVTDLKFLKSKQCRDNKVKLYFSMMKPIVLMHVSINAGLIMMLST<br>SFCMVLLSTESFTQAFVNLFKIGIGIVYLSLQLFLYCHLFDNIHLNIQSVNLGIYSCNWTNMDLKFKKLLLLTM<br>QMNKANEIMMKASMKKIINLQLFASVLTTSYNIVSVMVKTIGK
AgosOR5	MPRIDAINVFLQMTGCTDSKRMLYLTYFEFLITLYYFIASYVSIIYYEQSVSIQLFTLLCMLIESYILLNITFRIYHK

	NQFREMDQYSKQLGIPDDYQSKINIITMYHLIASNMFVIFPVYFNTMKIVFTKPILQSMSFNAIYFGLTTTLVIQAIRGYINQTIVSICIASGIAAIINITIYTFYGSVLLDLHDEILRVLFDNSFFYVNKSFKRSILIMMLSYTIIKMILSSEAIKPHKMS
AgosOR25	MATTTKMVYKNEDNLMINTRLMKITGLYQLLDSRTSKIFGQNMLKCMSLFQLSIMFITVVIFLANTYYFSDDINAVMQYSILFVCDVLSILKLYVTIVKSDTIWNCIQMTSIDDLSYKYHNRSILRNGQLKSKSYSILIMFMWMNLIILWALAPLFVTNYFLEVEVKKKIYRYRFNIMNFVFPATDQFYNDNFVIYYCIEFACLIIWCHCTMNFDVLLLSTNITFKYQLKTIANSFSAFNITHYIKNNFTKNIKHYKESELIFDFKSIIYDQQRVIENMRNIYRIFQPVVLTQLAFESIIIILLSCIIMMNYFNGISLLSAMNLRLFAAVLTFTFHIYVICYLFDNVNQDRKSVV
AgosOR30	MVTTTKMTTKNEDNLMINTGLMKITGLYHVLDSRSLKIFGHNVFKCLSVVQMSNLILLTIIFPANIYYFSNDINVVMQYLMLLTSDMISILKLYVTIVNSDTIWNCIQMTSIDNLSYKYHDRRMLRDGQLKSKSYSILIMFMWMNLMLSWGLAPLFVTNYFLETQVENKIYRYRFNILSFVFPVTDQFYNDNFMIYYYIEFVYLILWCHCTMNFDILLLSMNITFKYQLKTIANSFSTFNITHYVKNNLTKNVKHRKESELMSDFKSMIYDQQRIIENMRNIYRIFQPVVLTQLASESIIIILLSCIIMMNYFNGISLLSAMNLRLFAAVLTFTFHIYVICYLFDNVNQQKDSINFALYSHDWTQSNAQHKILLLHAMRMNNAENLRLQVTKNRIVNFKMFTDFWPVGVEFKRSDIIIGIILLTTSRLESCRIFTQNKIAFVVL
AgosOR29	MATRTKMATKNEDNLMINTRLMKLTGLYHLLDSRSSKSFGHNVLKYLSLVEMSILFTMMVIFTANLYYFSDDINAVMQYSMLFACGVISIHKLYVTLVKSDTLWNGIQMTSIEDLSYKYHDRSILSKGQLKSKTYSILIMFSWINLIISWSLAPLFVTNYFFETRVENKIYRYRFNIMNFAYPATDQFYNDNYMIYYYTEFVFLMLWCHCTMNFDILFLSMNITFKYQESELMFDFKSMIYDQQRVIENMRNIYRIFQPVVLTQLASDGTFFFIQNYFNGISLISAMNLRLFAAVLTVTFHIYIICYIFDDVNQQVSYIKDSINFALYSSDWTQSNPQHKHLLLHAMRMNNAENLRLQVTRKRIVNFKMFTDVHSINFALYSHDWTQSNAQHKLLLLHAMRMNNAENLRVHVTQNRIVNFKMFTGIMRKAYSILSVLGKMCAKKT
AgosOR26	MNNAENLRLQVTRKRIVNFKMFTDVRTIFLFFFILVPTTDIIFGHNVFKCLSVVQMSSLILLTVIFLSNIYYFLDDINAVMHYSTLLTSDMISIFKLYVTIVNSDTIWNCIQMTSIDDLNSDTIWNCIQMTSIDDLSYKYHDRRILINGQSKSKSYSILIMFMWMNLIISWSLAPLFVTNFFLELEVENEIYRYRLSIMNFVFPVTDQFYNDNYVIYYCIEFTGLILWCHCTMNFDILLLSMNITLKYQLKTIANSFIIYLYNYVLYAFNNLTKNVEHHKKSELLMFDFKSIIYDQQRVIENMRNIYRIFQPVVLTQLASESIIIILLSCILMMWHFFFFIQNYFNGISLISVMNLRLFLAVLTFTFHIYVICYLFDDVNQQKDSINFALYSHDWTQSNAQHKLLLLHAMRMNNAENLRVHVTQNRIVNFKMFTGVRIIFFFYFNAYLQICLKRYNFIFYI
AgosOR41	MTFDKSILESNEVSINLKLFKFIRFFHLFDPNIKKICNLNVYHLAWYIINCVIGCIVIYGLLGYFTEKEDILNIVNQIQLMFCGLLYYSSLLKIIIFLYKANSIWDLLRVSHMNFLTSSQCKTHIGILHTHRNQSIKITNFISGFGIVIALEWIMFPLLLQLLQKEDVNKLNQRFENIFNFPFPVTINYYNNNYVIFYIMESFIAMYMLYIYVVVDVFFISVCYVMIAHYEMIKRAYENVNTELVSENNNKNKNYCNDCIYDLVSIMKDQQKHFALLKLFYSTYKFIILSTVTINSGSIIILTYASVVIFTSSESIPILSVMKLISAFGYMFVVLFFLCYLMERINNKIESVHLGMYSCNWTAMNLRSKKMLLISMQLNNANKLMIKITPKKIINLQLYNSVIITCYNVLSAMLNTRSE
AgosOR28	MDNFNGKNILINFKLCKQFQFYQMICSSRMKIFGWNIHQLVLYKQRDKNSKFVNYFLIFSFVIILKWFIFPIVINQILYFENSNVRAQNIINLCFGFVLIFYLLEITVTSLTVYILIMMDTLIIFLCSAIIYHQEVLIYAFKNIGTVLILIKIGSKVIYLTARLFIYCYMFDSINIKRELVNYSIYFCIWTKMDFKFKKLLFLTMQMNDANRMIMSMSFNMVLVLLKITNSENHKSQLT
AgosOR7	MDGFNEQNILIDFNLFKELQFYQIFYSSGIKIFGLNIHQLFYISYALVALCIESYGISTLFSTNCKFLSYIDYFIIFYVVNQMYLSFWKLFKCLNDRNRLLDLFKIAQLNFLTSEECTKYSKVLYKHRDKNSKFVNYFLIFSFVVILQWFMFPIVINQILYFENSNVRAQNIINLCFGVATLTYNKFVLIFYLLEITVTSLTVYILIMMDTLIIFLCSAIIYHQEVLIYAFKNIGYEDNPKISKIIFFYSKYKKLIRINVYFFRKIKLFYSIMKSTILLTVGIDSFYLIFFTYLFILICLTPGSDTLLPLIKISMSVVYITGRLFIYCYLFDSIYIQRESVNFNIYSCNWTKMNLKFKKLLLLTMQMNDANRMGIKASPKKIINLQ

	LFAGIMSMSFNMVPVLLKITNSENNKP
AgosOR34	MDGFNEQNILINFKLFKQLQFYQIFNSYGMKIIGWNIYHLFYIIYALVGQFIGCYGMFTSFSINCKYLSDTDYFIFVYTAIQMYLSIWKIFKCLKDSKKFLDLFKIAQLNFLTSEECTEYSKVLYKQRDKNLKFARYFLIFSFTVSIQWFIFPLVINQIINFENSDVRAQNIINFCFGVSTSTFNEYYLIFYLIEIIVASLTMYILIIIDALIISLYSAIIYQQDVLIYAFKNIGYEDKPTICKIIFLVYIFKMNVYFFRKIKLFYSLMKSTILLSVAIDSLYLIFLMYFFILVCFLFKLIQL
AgosOR24	MELQNEHSITNLQFMKITGSYQLLMPSHGLTFFNINIYKIAFIVQILFLTIAAIMGVFSIYSCRNNVNQIIHYIIVIFAIYFAIYKYYFIIKNSKIIWDCMHMMSTNFLSYNDHTKEIFKIARTRSFNVDIDFPYLSAILIHSSYLKIKHEHGIYKYRSNALGLVFPVTDTFYNKYFIVFYTTESIFLLFWGQMMWVFDILMISICISIEYQLKTIADSYSLLGLKDKHLTRELSNSKYIRFFVFIQLAAESFQIILHACMILKLYFDGSMSPTIFLKLLFPEITYLCHLFLTCYLFSIVNEQESMNFALYSSNWTDMNIKFKKLLLFTMRVNNAENLKMKISINRIVNMEMFADVMHITYSIVSVMMKSYSK
AgosOR15	MTPDNTLKYIINLKLMKLTGLYQLLNPDNPKSFGCNIFKLGGTLAVVYLILVIIMCNLSIYYSLNDFTEVVKYIMLIIAALFASTKMCFVILYSNELWKFISFTSIDYLSYKGHKKYMHNKARKLSKSISNIFTLAWIAVISVWILSPIIIKDNFMNVKSKDDTYNQYRYNMLNLIFPVSAQFYNNNFKVFYFFESIALIVYGYSMMVFDCLVISMCITITYHKPLDERSNDLNNLILIIQDHQKCIKNLNKGLSLTSPESIKLILSALTNIIHLFSTCYIFSIINTHKDSINFALYDCNWTNKNIMFKKLLLLSMKINNSEKLKLKASSQIIVNLQLFTNVIHTTYKIISVLVNQYR
AgosOR23	MNPNDENYIINLKLMKITGFYQLINPHTSKYLGFNVYKVGAGLEVMFGIISMLLLFLSSYYYLDNTNELMSHFMLIVAIFFSTFKISWVSKKSEMIWNNLDMTSINFLSYTGHKQEILQTARAKSISTTIIFVILWSSVTVAWSISPFFIKDVYLNVKFNDEIRRFRYNSLNYVYPITEESYNENFLYFYVVEMLQVIFWGHGTVAYDTFVISICISIAFQLKTIAVSYTSLNDIKGDIKNLKHNDLEAILNLKLVIQDQQKMFKKIKEIYKIFQPVTFVQLAAQSMLIILQAYMIFIFLLLSVPIIKLIVTVAPNIIHLFITCYLYSNINDQVLNLIIRHIIIIMTIIYPQNILIILSIILFQKDSMNFALYSSDWTAMSIKYKNMLLFAMRMNDAEKLKLKISLRKIVNLEMFASVKPKIKFYEKNNN
AgosOR4	MTLIENKNYHRKFYTILMTVAFFLNTSQYDCIPKFLMHFYIFDWMMFVTLAAGYIFFYEKPGMSLGMELIQYMIVGILYTLIFLVFILKNEAIMSNYNFIQTKFIHWSNKKSLHPNAVYKKNIETVKSLAIPLATLSLSIAFGPLVSTINDIGKLPLDNRAHFVLFWPKIVDTNKISMYGIIYAVQVLFTVILYISSLSFNLGFMVFLNELTNQFEILLDGINDAFKYKRDKQFQPLFIDCIRHHQIIIKFLDDLKSYFKWVILIEIIVVQVVLAILIYNLTKVNASIGYKVKVAGSLLFNLLPICFHCHVGEVILSLHTRLSNHIYNMVWYDMPNKNKQLLVIMFQRTQRDLTLSSALFSNERASRSLISKVIKQVYTILNVLLKT
AgosOR3	MKNTAVNEMKFYQILIAIAFSVNTGHPYNYIFQYIIYLGIFNYILSLIIAPLYIIWEGQTMSMIMELILYIICGILHSSHLIVIALNKQSIVATYSFIQTHFFNWSVKRGMDPNGAYKKNIKKIKYFVTIWSFLGCTIMFSPLLSTIADLGELPLDHRSHWNTHWPIFFSINNLYTYGFIYLLQTILAVFLITATGAMKCGFIVLLSELNYQFEHLLHGLEHAFNHRMEQKFKIIFFDCVRHHQYVDNFKSYFKWITLINIFMLQGIISTSLYCIIKIDAPIGYKMKQCGIIITHIAEFLFHCYLGEIISRMHNHLEEKVYNMTWYDMPNPHKKFLMIMLQRTQKDLVPNAAIFSSHSLSRSLMTKFVKQIYTLLNVLLKT
AgosOR35	MKRSRFYHIFNPNGSKIFNYNAYRLLLIILLVLVNGIVVYSSLGFFVKMEDTLSYIDSSVIIFVMINIFLCNWRFSVFLYNAKIIYDVFNVSRFDFFKSKHCCKNINVLCGHRDRTIKITNYFFVFSSTVMSQWVLFPLVLIAFTAPEDENIRQQNIMNLRYPVSTHTYNQYYYLFYLMEVIVAIFTMYSMIIPDILLMSWCWAIIAQQEILIQTFKYFGHEDSSQTVHYEDFKSILVDQIQLNLKIKSFYSVVRPVVLTYVAIISTCFIIVTYVLIVVCLSKESNSVLNIIKLGSSALYMCLDLFLYCYLFDSMNIKLESVNTSIYSCNWTKMDVKFKKLLFLTMQMNNANNLMIKASPKKIVNQQLFANIISMSYNIVSVMLKTTS
AgosOR45	MKIQDNDKQIFNLALAKFIGFYQVVDTEKVTFLGRHNVRYKIFVFLIVYECLIAAILLLNCLYYSENNPTEFIRYTGFVVNMFYASYKMYIVLKRTKDIWDCLSITQFDFTSYDHRDRRIILDLWRNRSIWFTNTFVVFSLIMLIFYTACPLAFNDTFIVMKSRDGLSSNYRLNVVNLYLFVPEEVYNAYFNIFHIIETFGICIFVLFIVVFDTIVMTSCLALSCQLHMNSAAFESVGHTSVVDSPNNNIDNKMKLPYENINGIAMYNNLKTIIIDHQNVLKKYDEFLSIFKPIMLLEIFVLSYSIIILWLIFLTNFIVGEFTDSKGVASMQTCFAIPFCIMQMFMSCYVFDILHNKHNNNMTIDYRLGSNK

AgosOR43	MDSKHEKQYIFNMKLAKIMGLYQILTPDSTSIFGYNIYHIVIVFFGSFMFIISMLFPIGLLYLRNDMIALMYYMG<br>CISNFILSSYKMGNILYYSKDIWKCIDVTNFYFISYKHYDRNLFKNWQTRSIRITYIYIVIALFAFFCWIFSPCVM<br>NKSIITIRNIDGSYSRYRMNIFNIYLIASHETYNTNFYIFYIIEIIVSVCYVYFTIIFDVLMLLICFAISYQLETISNTIK<br>SLGYNLSIQDNIGTKCVIFTGTSNSIQLKEKCDIIYNDLITIITDHQHLNDFYNMFRLVTLTQIFIASSSHSIDEGD<br>NADSILSFKLFIVLPLINFQLFMTCSLFGTINEKKDSIIFALYSSNWTDMDLKSKTIILFGLTMNNANQLKMKFT<br>NTKIVNLEMFSHTMRFCYSIFSMLVNYNNKKKNN
AgosOR12	MDSKALKILRLTGLYQILDPNTAKIDDCNIYHIVIVFFASFTLVVSMLFPMGFIYLRNDIIALMFYVGCISNFML<br>CCFKVLNILYYSKDIWKFIDVTKYYLTLYKHYNTNVLKNWQTRSTRIMYLYIIIMFIGFCFWFFSPHILNESTVTI<br>RNIDGSYGKYRLNIFNLYLIVSDETYNMYFYVFYVIEIIMQICFLYFTIVCDIIMILISFEIISRMEIICIACGSLNYNII<br>CSKEGSNPIKSEKKFDVIYDDLKIIITDHKTVIRKLKEYYTIFRPVTIIQIFITSSSHIIIWFVVAMNFGEGDKGNSII<br>TIKLFAVHPLLSFQLYMMCYLFGSMNEKKDSIIFALYSSNWTEMDIKCKKMILLAMEMNNANHLKMKFTNT<br>KIVNKEMFTQVNITSHIYKVNYKMNVFYYL
AgosORco	MGYKKDGLIKDLWPNIRLIQLSGLFISEYYDDYSGLAVLLRKIYSWITTIIIYSQFIFIVIFMVTKSNDSDQLAAGV<br>VTTLFFTHSMIKFMYFSTGTKSFYRTLSCWNNTSPHPLFTESHSRFHAKSLSRMRQLLIIVSIVTIFTTISWTTITFF<br>GESVWKVPDPETFNQTMYIPVPRLMLHSWYPWDSSHGLGYIVAFALQFYWIFITLSHSNLLELLFSSFLVHAC<br>EQLQHLKEILNPLIELSATLDSAVHNPAEIFRANSAKNQPINGVDYNGSYVNEITEYGTKGETELNRKGPNNL<br>TSNQEVLVRSAIKYWVERHKHVVKYENLIRDCYGSALLFHMLVSTVILTILAYQATKINGVNVFAFSTIGYLMY<br>SFAQIFMFCIHGNELIEEVTVMEAAYGCQWYDGSEEAKTFVQIVCQQCQKPLIVSGAKFFNVSLDLFASVLGA<br>VVTYFMVLVQLK
AgosOR17	MATASPSEEESTIVDNRLFKAICLHQILNPTHGSNRYIRSQEPFSKHQSAIMATASPSEEESTIVDNRLFKAICLH<br>QILNPTHGSNRYYRIAILACIWMSIVVQITQLVGLYYAVNDLQRFAFTTTVVVNSFLSLAKAYVLMANVDRLR<br>DGLEAARYEFTSCGSRDQRTVRRARAALSTLVRTFTVFSYVTCFFWMLNPLSAIGEFLPLTNADGTVSHYRVTI<br>YNYWLPVSATVYNTTTVWALTYAIEMTVCFFNVNTWLLFDSYVLTMCFTFKAHFRTLSASYATIGHLDTFRSL<br>TPHASGFLNWKKGLNKTQNKFKFKHVLINIL
AgosOR37	MKWLQDHEVAINLALFKRYQFYHIFNPNSSKILNYDSYKFTNVLFIVVVTSYNIFSAMCFFTDTVDTIDSIDLL<br>LMIFIYSIIIISLLKICVLLFNADQIWDLFNLTRFDFLSSRRCRKNVGILYKYRDRSITITNLYQNYSTIVFIIWMIVP<br>LVLNTFAMTEGPNQRYHNIFNMQYPVSASIYNHYYYIFYLMEIAMGIFVLNYSMIIDNFLISLCWAIIAQYEVIT<br>TAFENIGNDCKLENLQKEKKNNSFEAYEDLKSIIMDQKKIYTLICSSAESFSIFNILKISTAFFVFVIQLYLYCYLF<br>DVLNDKKESVNLGIYSCDWTRMDLKLKKILLIAMKFNNANQLKIKATPNKIVNLQLFSSVMTTTFNIVMVM<br>LKTINEKN
AgosOR13	MDSKHEKQYIFNMKLAKIMGLYQILTPDSTSIFGYNIYHIVIVFFGSFMFIISMLFPIGLLYLRNDMIALMYYMG<br>CISNFILSSYKMGNILYYSKDIWKCIDVTNFYFISYKHYDRNLFKNWQTRSIRITYIYIVIALFAFFCWIFSPCVM<br>NKSIITIRNIDGSYSRYRMNIFNIYLIASHETYNTNFYIFYIIEIIVSVCYVYFTIIFDVLMLLICFAISYQLETISNTIK<br>SLGYNLSIQDNIGTKCVIFTGTSNSIQLKEKCDIIYNDLITIITDHQHVIKKLNDFYNMFRLVTLTQIFIASSSHVF<br>IWSIGAMSIDEGDNADSILSFKLFIVLPLINFQLFMTCSLFGTINEK
AgosOR16	MDSFNEQNILINFNLFKQLQFYIFYSDGTKIFGWNIHQLYIVFGLVGLCIQCIGLSTSFFNNCKNISKIDYFLMVY<br>ASSQMYQSYWKIFKCLKDRNRLLDLFKLAQLDFLKSEECAKYSKVLYKHHDKNLKFSNYFLIISFVVIIQWFVF<br>PLVINEILNFENSNVRAQNIINFCFGVSTQTYNKYIPIFYLLETTGAALTVYFLTMIDTLIISVCSAIIYQQNVLIYA<br>FKNIGYKIKLFYSIMKSTMLLNIVIDSFFSMIFTYLFILVC
AgosOR19	MGVLVVNGLMCLLKGYMVVANADRMCSTLDATRYAFTGCGGRDPSELRRCRATLSTILRTFVALSFGTLVV<br>WVIIPGKYELFFRIVRPVVLLQIANGSYSIITLIFLTSIAYLNGDSIVSPAIFKLVCALISLTIELYIYCYGFNHIEDGR<br>STVNFGLYSSNWTDKDLQFKKTLLLAMTINSAHKLKMKVSPNSIVNLEMFTRVMNMSYTIVSTLLS
ApisOR1	MYTFSTNMGYKKDGLIKDLWPNIRLIQLSGLFISEYYDDYSGLAVLFRKIYSWITAIIIYSQFIFIVIFMVTKSNDS<br>DQLAAGVVTTLFFTHSMIKFVYFSTGTKSFYRTLSCWNNTSPHPLFAESHSRFHAKSLSRMRQLLIIVSIVTIFTT

	ISWTTITFFGESVWKVPDPETFNQTMYVPVPRLMLHSWYPWDSSHGLGYIVAFVLQFYWIFITLSHSNLMELL<br>FSSFLVHACEQLQHLKEILNPLIELSATLDSSVHNPAEIFRANSAKNQSINGIDHDYNGSYVNEITEYGTKGEN<br>EPNRKGPNNLTSNQEVLVRSAIKYWVERHKHVVKYVSLITECYGSALLFHMLVSTVILTILAYQATKINGVNV<br>FAFSTIGYLMYSFAQIFMFCIHGNELIEESSSVMEAAYGCHWYDGSEEAKTFVQIVCQQCQKPLIVSGAKFFNV<br>SLDLFASVLGAVVTYFMVLVQLK
ApisOR2	MDVMQKPERFILTPFQKFCIRWSVFFDSSSDRLSRIETVLRTIQFSTIMITSGMTMTSVLIADNKKALESFTYFVI<br>CVFMLAIITFAIRTKRFNRAMLLMVVDEFPGYNRPMPDVLKRKMAAIRTSYGDFTMKVIVSYLTLVLFEIPATA<br>MVPLAAASLTDVKLGSQSTQMVVLWFPADTSQVGMYAVSYVIQFLIVVTVKFIITGIMCSFSFFVSQMISEFQIL<br>SAYVEHAVEIVEYDQSADKTTEQKLLDHVKNCVMLHDRLIYFKDQLNESYGYIILLELMFSTLYFCLSAFNMIF<br>VGNRFVMIKGLLTLSNYLAELFIFCMYGSMVEDAHMGLLRASYSVAWYAQPVRFRQSLTMVMSRTQTPLQL<br>TVGKVFIANLPLFLSVLKVSYSGVNALRAANAK
ApisOR3	MKTSENITARKMSVYWLTLLLLNGINVYPGHGNRVVRIAAAAYPWMICAFWAFITTSVTTSLALRATSYQEA<br>VEMLTYITGSSSTLALFAIGVHNRPGLHRMLDAVRRDFWDDGRRPAADVLFSRFVRTYGAILPIANVMMCM<br>TPVIWAARNGDIESPAALIFRMWTPWTRLTTARYAVVYAAQFVVSLSVLTSISGMVFAMVLFVTEMQVQVDT<br>LVDAVQDLHVDMWYGDGDGRPDAHPDRRRRAAFDGLVKCIKHHQALITYFNRFKSYFNLLFIVDILYIMVM<br>TCLCASSVLMANGFSAFHIKMMSLLIIVVSQFFFYCLIGEQFSTMNQQIGDCVYFKLVKCKDPMLSRAGLLVIL<br>RTQKPLQLTAMGITTYTASLFTFTVTMRSAYAGLNVLYNSS
ApisOR4	MTSIGKKNQRKFYQTLMTLAFFLDTSQYRYISRFVKQFYIFDWMVLVSVAAAFTILEGNYRMPFVMELIQYMI<br>VGFYFTSIFVVFIIKKEAIMSNYNCIQTKFIQWSNKRALHSNAAYKRNIKTVKSLSIPLAILSLSIALGPLISTINDI<br>GKLPLDNRAHFVLFWPTIVDTNKLSMYGIIYTLQVIFTIILYISVLSFNLGYMVFLNELITQFEMLLNGINDAFK<br>YKMDKQFQTLFIDCIRHHQIIIKFLDDLKSYFKWMILIEIIVVQVILAILIYNLTKVNASLGYKVKIAGSILFNLLP<br>ICFHCHIGEVVLSLHTRLSNHIYNMPWYDMPNKNKQLIVIMLQRTQRDLTLSSALFSSERASRSLISKVIKQVY<br>TILNVLLKT
ApisOR5	MQRIDTINMFLQMTGCTDSKAMLYLTYFEFLITFYYLIATYASIVHFEQSVTIQLFALLCMLIECVILLNITFRLY<br>HKNHIREMHQYSRRLGIPDSYRSVINVITKYHLIASNIFVVFPVTYAIFCDSVRVGDPFTFPFLDVLPMHTDNL<br>AIYACKYLVYAISVYIAHVELCFINTTFIYYVGVLKHRLETIVQTIGEAFADNDEQKFKYAIIQHQKLLSYFNTM<br>KKVFSKPILLSMSFNAIYFGLTTSFVIQAIRGYINQAIISICIASSAAAVINITIYTFYGSELMDLHDKILHVLFDNA<br>FFYVSKSFKSSILIMITRVTIPLKFTVGYIFTINLNLLLKILKMSYTVLNVLLSSETIKPHKLS
ApisOR7	MAHAVDNFFQKTGRSDVRRYATIMTCVEFFTYCDLVVTLFFAISAFLSIVYSEEDQLSNRVYGFLWLFVEIHVF<br>GLIVVRLYHQSQCRDMYDRSLLIEQGIPKNYRRTIAMVIAYYCIMSTVHVTVPMLYTISSDSAQVGDPFAFPFA<br>DVLPIKTTNPTAYVCKYIVYAFPVYLTHLECCFMNVTFMYFTGVVKRHFQILDQQVQEAVANEDEQKLKIAIE<br>YHQESLKFFKEMETVYEKPLIMTIEFCGLYIGLTGYIMIQCIQGIIHPIILGLCIASSTASLITISIYCICGSNMYDLH<br>DGILNSLFEHQSCYSRNKSLKHLILMMMKRATIPLELKAGSIFKINSNLLVKILKFTYTVFNLLLTSVNRQIKET<br>AI
ApisOR8	MTDIVEIFLQRLGCSDDGDGRGTRRAVFFTYCESAITLFFAVLTYLSLVYSSTGDNLSIRIYGLLCFVIEIYIFAYTA<br>VRVYHQSEHHDMYQRSRQMGIPENYRRKIATVIKYHLVMSNVVLAIPMLCTILLDELRMGDPFTFPFADVMP<br>IKTANVTVYVCKYILYALHTYFAHLELCFLNVTFMYSTGVVKRHFQVLDEQVEEAMVTEDEQKLKIAIIHHQ<br>QVLKFFKDMKTVYEKPLLLTIEGFGLYIGLCCCAIIQVIQGFVDQIILGLCMASCVAGFMTISLYCICASNMYDL<br>QNGILNSLYEHRACYSRNKSFKRLNLIMMTRATIPLEFNVYSLFIVNLNLLVKILKLTYSVLNVLLTTINLKFKE<br>TAT
ApisOR9	MAHIVDIFLQKMVCSHDRGYGMVFFNYCELAITLFFTVSTYPTIADPTENLSIRLYGVLCLLIEVHIFAFIAVRIY<br>HQSQHRDMYQRSQGVEIPGNYRRKIATVIKHYFIISNVFVAVSVLYTILLDWVRIGDPFTFPFIDVLPIKTTNVT<br>VYVCKYIVYALPVYFAHLETCFLNVTFMYSAGVVKRHFQILDEQVEEAVVNEDEQKLKIAIKHHQQVLKYFE<br>DMKTVNEKSILVTIEFCGLYVGLTSCFVIQVMQGFIHQIILGLCIVSSMACLMTIIIYCIYASNMYDLHNGILNAL

	FEHRSCYSRNKSFKRLILIMMTRATIPLEFKAGSVFTINLNLLVKILKFAYTVFNVLLSSINRQFKETRM
ApisOR10	MAHIVDIFFQNMGCSHDHGYGMVFFNCCELAITLFFTVSTYPTIADPTQNLSIRLYGVLCLLIEAHIFAFIAVRIYHQSQHRDMYQHLHGVEIPENYRRKIATVIKHHFIISNVFVAVSVLYTISLDWVRIGDPFTFPFIDVLPIKTTNVTVYVCKYIVYALPVYFAHLETCFLNVTFMFSVGIVKRHFQILNDQVEEAIVNEDEQKLKIAIKHHQQVLKYFEDMKTVYEKPILMTIEFCGLYVGLTSCFVIQVIQGFIHQIILGLCIVSSIACLMTIIIYCIYASNMYALHNGILNALFEHRSCYSRNKSFKRIILIMMTRATIPLEIKAGSVFTINLNLLVKILKFAYTVFNVLLSSINRQFKETAI
ApisOR11	MADIVEIFLQRLGCSDDGAGHDTMRVVFFSCCEFTVSLFLAILTYLSLVYSKEDLSMRFYDLLLLIVEIVVCSLIVIRVYHQSQHRDMYQRSQKVGIPENYRRKITTVIKYHLVMSNVIVVIPVLCTISLDWVRMGDPFTFPSIDVLPIKTTNVTVYVCKYILYALPTYFAHLEMCFMNVTFMYSTGAVKGHFQILEERVEEAMATQDEEKLKIAIKYHQQTLKFFKDMKTVYEKSLLIAIEVSMLYIGLSGCTMIQVMQGFVDPIILGLCMGSCVSTFMTISIYCICASNMYDLHDGILNAIFEQQSCFSRNKSFKQLVLMMMTRATVSLEFRVYSIFTINLNLLVKILKFTYTLLNVLLTSVNRQFKETAK
ApisOR12	MADIVEIFLQLLGCSDDGAGHGTMRVVFFSYCEFAVTQFLAISTYLSLVYSTEDPSIRIYGLLFFIVEIVVCSLIVVRVYHHSQHRDMYQRSQKVGIPENYRQKITMVIKYHLVMSNVLVVIPLLCTISLDWVRMGDPFTFPSIDVLPIKTTNVNVYICKYILYALPTYFAHLEMCFMNVTFMYSTGAVKGHFQILEERVVEAMATQDEEKLKIAIKYHQQTLKFFKDMKTVYEKPLLIAIEVSILYIGLSGCTMIQVVQGFVDPIILGLCIGSCVSTFMTISIYCICASNMYDLHDGILNAIFEQRSCFSRNKSFKRLVLMMMTRATVSLEFRVYSIFTINLNLLVKILKLTYTLLNVLLTSVNRQFKETAK
ApisOR13	MADIVEIFLQKMVCSDDGAGHGTMRLVFFAYFELAVTFFFAISTYLSLVYSTEDLSIRIYGLLFFIVEIVVCSLIVVRVYHQSQHRDMYQRSQKVGIPENYRRKIATVIKYHLVMPNVLLAIPVLYTISLDWAQMGDPFTFPFADVLPIKTTNVTVYVCKYIVYALPTYFGHLEICFLNVTFMYSTGVVKGHFQTLEEMVEEAMVTEDEEKLKIAIKYHQQALKFFKDMKTVYETPILITIEVSILYIGLSGCTMIQVIQGFVNPIILGLCIVSCVSTFITISIYCICASNMYDLHDGILNAVFEHRSCYSRNKSFKRLILMMMTRATVSLEFRVYSMFTINLNLLVKILKLTYTLLNVLLTSVNRQFKETAK
ApisOR17	MTTTPRVTELTAPASEDLTIVDNRLFKAICLHQILDPTKGGNRYYRLAFMVVMWVSLSVQIIQLVGLYFAVNDLQRFAFTTTVIFNALLCLSKGYVLVVNADRLRASLEVARYEFTSCGARNQRLVRRSRAVLSTILRTFAVLSWVTCFIWALTPLFAMDEYLQVTNADGTVSRYRVTIYNVWLPVPATVYNETTVWSLVYAVEVIACFVNVFSWLLFDSYVVTMCFTFNAQFRTVSASCTTIGHHSDSFRSPPPHAPEGTSDDNNTFNCYDELINRIKDNQSIIKIYDDFFEILQPAILFQIIGGSYSVITLIFLTSLTYLMGFSIISIPVLKVFFGFLSVTFELFLYCYVFNHIETEKCNMNFGLYSSNWTAMDLKFKKTLLFAMNTNSSHRRVMKVTPMSIINLEMFANVMNMSYSIVSVLLNSRVQK
ApisOR18	MTTPRVTAFTVPASSEDLTIVDNKLFKAICLHQILDPTNGGNRFCKLVLMAFMSVSLSVQIMQLVGLYFAVNDLQRFAFTTTTLSYAFLCMTKDYVLLAHADRLRDSLEVARFEFTSCGARDQRVVRRSRAVLSMVLRTFAMLSWSTCVIWALVPLFMMDEYLQVTNADDTVSRYRVTIFNMWLPVPVAVYNATPIWSLIYMVEVIACLFTSFSWLLFDSYVVTMCVTFNAQLRTVSASCATIGHRDCFASLSPHVCTGTHIIKIDDNSILSNCYDELIIHIKDNQNIIKKYDDFFEIIQPVVLFQIIAGSYSVITLIFLTALSYLMGWSIISGPVLKVFFGFLSLTFELFLYCYVFNHIETEKCKMNFGLYSSNWTAMDLKFKKTLLFAMNVNSAHRRVMKVTPTSIINLEMFANVMNMAYSIVSVLLNSRVQK
ApisOR20	MRSSSATVVDVMLFKAIGLYQLLCPADRGGYSVRSRRALMTALGLSFALHSFQVPYLYYALNDLQRFAYMAAVIIYGMMCSFKGYVLVTNADRLWLVLNAADYGYTGCGHRDPSRLRRCRATLSALLRTFVALSYGTLIVWIVLPFFVDEYTGITNSDGTVTRYRTTIHNMQYPIPLAVYNSRPVWALIYVTELYVCIVNVFIWSLFDCYLVTMCFVLNAQFHTMSAGYGTLGIRRTGSSPPDTTFAGVRRIKFDEIESNHYSDLISHIQDNQNLIKMFDVFFEVVRPVVLVQIANGSYSVISLIFLTALMYLMGVPVLSAAFLKFICGLISLTIELFIFCYGFNHIETAKSVLNFGIYSSNWTEMDLTFKKTMLLTMKMNSSHKRAMKVSPNSAVGLEMFARVMNMSYSTVSVLLNSRS
ApisOR21	MIGVHQLLRPDEYGQDNDLYRTAAKVIVGLTLVLQSMQVCRLYLARHDITMFAYIGVMIINGLMCLLKGYMVAAKADQMSATLTAANYAFTKCGGRDPSKLRLCRARLSAILRTFVGLSFGTLIVWLTMPWFMASDYDDQPFIWGVVYVIESIILTVNVFCWTSFDCYLVTMCFVFEAQFCTMSTGYETLGRRRTGAKSSAPQKLGNASTINNVKISDVNYDDLTSHIRDNQNIIKQYDAFFDVVRPMVLIQIANGMYSIIMLIFLTLVTHLSGYSIFSAPILKFVCGLASL

	TIELYIYCYGFNHIEDGKSTVNFGLYSSNWTEMDLKFKKTLLLAMTLNSAHKRVMKVSPNSIVNLEMFTGVMNMSYSIVSVLLK
ApisOR22	MDVKLFKAIGMYQLLHPVECGLNSDLCRKTAMMVVGLTVGLQLMQVFRLYLARHDIPMFANMAMLVVYGFMCLLKGYTLANHADRICITLEVARYAFTDCGRRDPSLMRRCRARLSTILRTFVGLSFGTLVVWLVMPWFLASEYDGKPLIWAVVYVVESIILTVNVFCWTSFDCYLVTMCFVFEAIFRTMSSGYEKVGQVKANVTLTFPSHYDDLISHIKDNQKIVEKYKTFFEIVTPTVLLQIADGSYTIITMIFLISIAYLNGNSILSPMILKYVCGLVSLTIELYIFCYAFNYIEDGRSTVNFGLYSCDWTDKDLKFKKTVLLAMSMNSANKQVMKLSPNSIVNLAMFSRVMNMSYTIVSTLLS
ApisOR23	MNLNDEQNYIVNLKLMKITGFYHLISPRAPKYFGFNVYKVTAAIEVMTGIFSIIMLFLSSYYYLDNTNELMSHFMLVVAIFFSTLKIFWVSRNSETIWNNMDMTCINFLLYTGHKKEILKKARAKSISTTILFVILWSSVTVAWSISPFFVKDVYLNIKFKDETRRFRYNSLNYVYPISEEFYNEHFLYFYVVEMLSVVFWGHGTVAYDTFVISICITIAFQLKTIAVSYISLNDKKGDIKNLKDNDLEAMFNLKLLIQDQQNMFKKIKEIYKIFEPVTFVQLAAQSMLIILQAYMIFINHYNGFSLLSVPIIKLIVTVAPNIIHLFITCYLYTNINHQQDSMNFALYSSDWTAMSINYKKMLLFTMRMNDAEKLKLKISLRKIVNLEMFASVMHLTYSIISVLAKSYGNTNTK
ApisOR25	MATGIKTVSKNEDNFMINMRLMKKTGFYQLLDSRSLKVFGHNVFKCMSVVQMSILSSVAFIFVANIYYFSDDINTVMMYSMLITSDVLSILKLYYILQNSDTIWNCIQMTSIDDLSYKYHDRRILEEGRSKSTSYSILIMFMWLNLIVSWSLGPLFVTNYFLIVEQNDEIYRYRFNIMNFAFPATDRFYNDNFMIYYGIEFITLVLWCHCTMNFDVLLLSMNITFKYQLKTISNSFKSMFDFKSLIYDQQRVIENMKNIYRVFRPVVLTQLASESLIIMLLSCIIMLNYFNGISLLSALNLRIFAAISTFLFHIYVICYLFDDVNEQKDSMNLALYSSDWTTSDLQHKILLLHAMRMNNAENLRLQVTRNKIVNFQMFTYIQSYNYSLVVRIAIYIVIKSRV
ApisOR29	MVFKNEPKLMENIQLLKTIGLYQILDSHSPKVFGYNVFKCVAVIEAFILTATVYASILNIFYCLSDINEATRYFTLCLIASVPTFKLSYIIGYSDTIWNCLHITSAEYLSYKYHSRCILEVGRKKLKQFLILFVILWIVVFIAWILTPFIVQNSYLRVEARNMTYHYRTNILNLVYPAPDKFYNANFIMYYNIELTISIVWAHSTIIFDILLISMCITIEYYLKTIANTFSTLGNVENQFMIRLDDTKIINDFKIIIQDQQKVIENMKNIYKVIRPVILLQIVAESSIIILLSSITIMNYFNGFSLVSPSNFRFITSIFIYILHIYFICYFLNDVNEQKDSMNFALYSGDWSGKSLKYKKMILYAMQMNSTDQMKLQVTKTRVVNLELFTSVMRTTYTVISVLSEQYAKKT
ApisOR30	MVLKNEPKLMSNIPLLKMIGFYQILNSRSQKVFGCNIFKCIATIEACILIAGVFALILNAFYFLSDINEATSYFTMGVMVSVATCKLFYIIGYSDTIWNCLHITSVEYLSYEYHSRCMIDVGRQKLKSILIISMVVWITTSIGWLLTPLIIQNSYLRVEVKNEIYHYRTNIMNLVYPATDTFYNDHFIMFYIIEFIVPIGFIHCTLIFDILLISMCSTIACYLKTIANSFSTLGNVENHCMGHDEMKTLNNFKIIIRDQQKVIQNMKNIYKVIRPVILLQISAATSIIILLSSMTIMNYFDGLSLISPLNFKFMSTMLTYAMHMYLVCYLLNDINEQIDSLNFALYSGDWTSKSLKYKKMILLAMCMNSAIKLKMQVTMTRIVNLELFAGVMRTTYSIISVFSDQYAKNKI
ApisOR31	METCNDHTCTINLNILKQCGFYQIFDPNSKKIFGWNVYRISFIALTVITQCLIGFGNCGFLFELEDTTDNIDLFLIIFSNSYFCLTEWKVVILIINRKKFLELLDVTDLIFLKSKQCRNNIKILCKHRIRTLQLTNLYFMFCIFVIIEWIIFPIMINSFIAHKTENRRLENVVNRRYPVDVNTYNKYYILFYVFEIIIGVKTVYLVLMVDILLLSIGWAIIIQYEVLAEAFKNIGYNENLQKDHDHDVDDYKYFKSILFDQQQLDSKVKLYFPIVKPIVLMHVAINSVLFIMLSNSFLMVFLSTESFTYKIVNLFKIGTGILYICLQLFLYCHLFDNINLKRKSVNLGIYSCNWTKMDLKFKKLLLLTMQINDANYITIKASTKTIVNLPIFANVLMTSYNIVSVMVKTMSKYRKT
ApisOR32	MNTFKDQDVAINLKLLKQCHFYQIFDSSNRKVFGWNVHQISFVMFAVVVQCFVCYGNAGSVFEKDDVVTNIDYFLIFYTNIHTYLSLWKLIVYLYNAKKILKVFNVTRINFLTSETCCNYSEILHKYRDKTIRFTNWYIIFSIVVIIQWLIFPLVLNMFMISGNSNVRYKNIMNLRYDVSTHTYNQYIIIFVLMETTTLSFAMYFMVMTDLILISFCSAIITQQEVLIHAFKNIGHEDKSQIKYYEQLKSILRDQQHLNLKTQSFYSAMKPIVLLAVAINSTFIIILTYLFILVCLTTESDTSDTALILIKLGSSAVYISLELFLYCYLFENMNIQRERVNLSLYSCDWTKMNLKFKKLLLLSMQMNNANQMLIRASPKKIINLQLFASIISMSYNVVSVMLKTTTPKSSR

ApisOR35	MGNLKVEEVTINLKLWMLYRFYHMLKTNNYIKIFNCNVYRLILFIYGAIVNCMVAYSIIGFFVEMDDIISNVDVFLVVFVMINFFFCSWRMCIILSKSNTICDVLTVSQLNFLTSKQCVKHSNVLYDYRDRTIKITNYFFVFSVIVLIQWIIFPIMLITFTESDIENIRLPNIMNLRFPVFTYTYNQYYFIFYLMEVTIATFPIYVIIVTDTLIMSFSLAIISQQEVLNRAFRSVGYEENSQSEYYEDLKSILEDQIQLNLKIKSYYSIVRPVILANVAMSSTCFIIVTYVFIVVCFSKEPNQILTIIKLGSSAIFICGQFFLYCYLLDSMNLKREYVNFALYACDWSKMDIKFKKLLLLTMRMNDANNFIIRASPSKVVNLQMFANVISMSYNIISVMLKSMDSNNQISE
ApisOR36	MLYRFYHMLKANNYTKIFNCNVYCLILFIYGAIVNCMVVYSSIGFFIEMDDIISDVDVFLVVFVMINFFFCSWRICIILSKSHIICDVLNVAKFNFLTSKQCFKHLNALYDYRDKTIKITNYFFVFSVIVLIQWIIFPIIVITFMESDVENSRSPNIMNLRFPVSTQTYNQYYFIFYLMEVAIAAFPIYVIMVTDTLILSFSLVTISQQEVINRAFKSIGYEENSQSKYYEDFKSILGDQIQLNLKIKSYYSIVKPIILANVAMSSTSFIIVTYVLIVVSFSKESNQILTIIKLGSSAIFICGQFFLYCYLLDSMNLKKESVNFALYSCDWTKMDIKFKKLLLLTMRMNDANNFMIRASPRKVVNLQMFANVISMSYNIISVMLKSMNSNNQSTE
ApisOR37	MKWLQDHEVAINLALFKRYQFYQIFNPNGSKLLNYDTYKLTNVMFIVAVTTYNIFSAMCFFTDTVDTIDSVDLLLMIFIYSIIIISLLKISVLLFNADQIWELFDLTRFDFLTSRQCRKNVGILCKYRDRSITITNLYQNYSTMVFIIWMITPLVLNTFVVVGGPNQRYHNIFNMQYPVSANIYNQYYYLFYLMEIAMGIFVLNYSMIVDNFLISLCWVIIAQYEVITTAFEKIGNDCELTTLQNEKNNNSFEAYEDLKSILMDQNKLYIKLKSFYRVVWIIVIFLIIIDSVLLIILTYSFVMICSSAESFSIFNILKISTAFFVFVIQLYLYCYLFDVLNDKKESVNFGLYCCDWTKMDLRFKKLLLLATKFNNANTLKIKSTPNKIVNLQLFSSVMTTAFNIVTVMLKTMNGKN
ApisOR38	MSSLKSNEVAINLKLFKVFRFYHIFDPNSGKLCKFNVYHLAWYIINCVIGCILIYGLLGYFTEMEDVIDSIFHIQIMFCYLLYSLSLLKIITFLYKANNIWDLLRVTRINFLTSTQCQAHIGILHKHRNKSIKITNLISGFAIVTTLEWILFPLVLRLLSKTDASHSNKRFENIFNFRFPVTVCEYNNNYYFIFYIMESFIAIFMLYAYVVTDVFFISVCYVIIAQYEIIKRAYEIVNCEQTSENNNENKNHNNIIVNDCCDDLISIVMDQQNHYAKLRLFYSTYKLIIVSTVVINSGSIIILTYASVVIFTSPETIPILSIVKLISAFTYMFFVLFFLCYLMECINNKIESVQLGMYSCNWTAMNIKSKKLLLFSMRMHNANKLMIKTTPNNIINLQLFNSVMMTSYNIVSAMVNTRSK
ApisOR39	MFSCDFINRTVNMNSENLFNGGSVAFNLSTYKQLGYYQLLDPKGPHIYGYHLYRTILKIFLLIVQFITIFGVMGFFIEMEDTDPGKSNSFELIIILTNCSLSSLKIYTLISNSKIIWDLFDLTRIDFLRCSRHSKLITKNFVKRCKKSTTITKWIARSFLVGLILWLMGPFIANEEHTEPNTVHRHKNIINIKFPVTMKTYNNYYFVFYLMEVAVGFCIVYGSVLIDAYLMSFCWIISAQYQSVTKAFATFGYNKQGSPKDIYKDFKSIIIDHQNIYLKMKSFYAVVRPITLIHVFAYSCSLIMYAYVIVTIFNSKELFIIAEIMKIVMTVSNVTMEVFIFCYLFELIDNKKEDVNFGLYSCNWTGMDIKFKQLLLMSMKMNNANRFKLKASPDVTINRPFFANVIHTCFKIVSVLIQTQSIDLLN
ApisOR40	MNTTDKKYAFNLTLFKTIGYYQMVDPNSKKIFGFNIYNVINITLVIFTSIMTVIGLSGFFYKTDNITSEENNFKDLQMLFYLSCIGLGNLKIAITVYNADAIWKLFNVAHESFLSNKYCKHDKYKLNNCGKQFARIFPWYFFMFIMTAFAWSVVPIVVNNHAASNETQNNENAYMTNIANMRYPITVKTYNTFYKGFYALEFIMVWYSAYGLVVFDLFIVALLQLLATHYEIISSAYENFKYKAENEDGKLRKEEIQKELVSIIFDCQTIYRKLETLYGFSRPIVLVYMVGDAIGMITMPFLIVMSYVQSGSSIFNTNVMAFSWTLFVVGIQSYMYCSLLQNLNERKEDVNFGLYGCDWTSLDIEIKKLILLAMRMNSSNNLKMKVTSTKFIDLPMFASIVRSSYSVTSVLINSNIDKITK
ApisOR41	MNKQKIYDSNFTLFKLIGVYQMVDPHSQKIFGFNVFHFVSMVFIIFTTSMTILGLSGFFYKVHNTNYNNSDVDTIFIMFYTVCITIGNLKVMIIIFKARQLRNMLEITDESFLSNTFYKRNYYKIVKCGGQLSKFFNLYFSFLLITLTSYAIVPIVLNAHFIDGTTQNTETIQKINIVNLKYPFTVETYNAFFKIFYASECIMLFYIGFGVFALNLFSMTILVIISAQYKLLASAFEVLEYRVNDEDDSLLSDEKLLETFISIVSDNQIIHKKIKMLYDIIRPVGLIQLMADALGMICMPYLIVVYFLEYGSLFNPETMKFVFTFGFAGVQSYMYCSLFQRVTDRREEVNFGLYCCDWTGMDIHMKKLILFTMQMNSSNKLKMNLTTNKCINLPLLSTIIRLSYRISSVMINYNINK
ApisOR42	MPNSSEECVMSSSMAKCTGLHYIIDPEGPTVGGHNVFHVTVMVMIGFTVVCLSMCPFGLYYWANDVTQCIFLLITIVNFSFGCFKAFTLVRHSDDICRCLDVTRFDFSSGAIMSDPDSARFFRKCRDASSTFTGWFAASSHFVLLV

	WTLLPFVVVGKGVEINNRDGSTSYYHFNPYNMYFLVSSETYNRLHLVFHLVEWAFGLCFVLIMVAFDTFMVTLCVAITCQMRGIGNAYSKLGHDRCATASNVCSDGGIESNKSNNEYLRDLKLIIKDHQAVLGKMNDFYKIVGPVILPQLIVASFTIIFVSFIITRNYFNGMLLTSTQSLKMCCFPIFFYQIYYTCHAFGNLSHQKNVMNFALYSSDWTQMEIKFKKLLLLAMQMHDANKLDMKLTDKLVINLELFTRVINMCYSIFSVLVNSQLKIADKQ
ApisOR43	MDSKQEKQYIFNMKLARIMGLYQILFPNSTSFFGYNIYHVVTVFFVSFTFAISMLFPIGLLYLRNDIIAIMYYMGCISNFLLSCFKMVNILYHSKDIWKCIDVTSFNYILYKHYDRNVFKNWQTRSIRITYIYIVIALFAFFCWIFSPCIMNKSVIAIRNIDGSYSKYRMNIFNLYLIASNETYNKNFYIFYVIEIIISICYVYFTIVFDVLMLLVCFAISYQLETISNTIKSLGHEIYTRDNIRSGNSIKLKEKHGILYNDLITIMTDHQNVLKKLNDFYNIFRSITLTQIFIASSSHVFIWFIAAMSIDEGDNADSILSFKLFIVLPLINFQLFMTCSLFGTINEKKDSIIFALYSSNWTNMDLKSKKMILFNLTINNASQLKMKFTNTKIVNLEMFSHTMRFCYSIFSMLINYNKNKMK
ApisOR44	MRFRYIFLQVTKRYITSTLIIMNIRGSNSDSIFNLKLAKIFGFYQILDTETVTFLGRHNVYYGIFVFLIVYQWLLSAIVFLNGLYYPVNNSNIIQDMFYFGFTVNMLYGNYKMYIILNRSKVIWDCLSITKFDFTSYGVQGRHTLNVWRNLSIKYTNIYVMFYITVSILCVGFPVVFSNSFIIIKNHDGLSSAYRLGLVNLFLFISEETYNTHFYVFYIVESLCLIINTLFIIIFDTIVNTLAIALTGQLQMISNAFESVGHKSLHFPNDNVDNKIKLPNENIKYMDHYKDLKTLIIDHQNILKKYDEFLSIFRPTMLLQVFVVSCSIIFLWFIFLTSFIEDDFTQYMALTSMEAIFGIPFCTFQMYMSCFVFNTLNIKKDSITFALYSSNWTEMDMKFKKLILLTMRMNDAHQQKLQYTKTKIINMEIFYHTMRVCYTIVNVMINCKKEKMVQQ
ApisOR45	MNIHGSNSDSIFNQKLAKIFGFYQILDTKTVTFLGRHNVYYRIFVFLIVYECLLSAIVILNGLYYPVNNNNIVQAMFYFGFVVNMLYGSYKMYIILSRSKVIWDCLSITKFDFTSYGVQGRHTLNVWRNLSIKYTNIYAIFYLTISILCVASPVVFSNSFIIIKNHDGLSNAYRLGLINLYLFVSEETYNAYFYVFHIVESLGLVINTLFIIIFDTIVNTLAFALIGQLQTISTAFESVGHKSLHFPNNNIDNKNKLPNENIKYMDHYKDLKTLIIDHQNILKKYDEFLSIFRPTMLLQVFVVSSSIIFLWFIFLTSFMEDDFTQYMALTFMEVAIFGIPFCTFQMYMSCFVFNTLNIKKDSITFALYSSNWTEMDMKFKKLILLTMRMNNAHHQKLQYTRTRIINMEIFFQ
ApisOR47	MDIQNEKHHVFNIRLANLIGLYQTLDPETVKFRGRNVYQIFVAFVALYLLVISLGLFAGCLHLWTYNTATSLLDLLITTNSFYASYKMWIVVYRSNEIWDCLSITRYGFTSLNNRKWNGHDILDRWRARSVRYTSLLAGAYFLTIVFYVGCPLVFGAAVIPIKNQDGSIGSYRLNVINLYLFVSDETYNEYYNTFFFIEALFIVGLIITCLLFDTLLLTLCLGICCQIQMICSAFESVNHNSPSDPHSSAIDNNDEKQIISNEHDLIHDELITIIINHQAVIKKFELFLTIFDRVMLSHIFVSSISLIILWFNLIMSFFNDGTFAISGDTTLKTIVAIPSFLFQIFMVCYLFEDIHNQKDSIVYALYSSNWTEMDMKCKRLILLTMQLNNANQKKLRFTRTKIVNLEMFFKTTGHCYTVVSVLMNYINAKNV
ApisOR48	MDIQNEKHHVFNIRLANLIGLYQTLDPETVKFRGRNVYQIFVAFVALYLLVISLGLFAGCLHLWTYNTATSLLDLLITTNSFYASYKMWIVVYRSNEIWDCLSITRYGFTSLNNRKWNGHDILDRWRARSVRYTSLLAGAYFLTMVFYVGCPLVFGAAVIPIKNQDGSIGSYRLNVINLYLFVSDETYNEYYNTFFFIEALFIAGLVITYLLFDTLLLTLCLGICCQIQMICSAFESVNHNSPSDPHSSAIDNKDEKQIISNEHDLIHDELITIIINHQAVIKKFELFLTIFDRVMLSHIFVSSISLIILWFNLIMSFINDGTFAISGDTTLKTIVAIPPFLFQIFMACYLFEDIHNQKDSIVYALYSSNWTEMDMKCKRLILLTMQLNNANQKKLRFTRTKIVNLEMFFKTMGHCYTVVSVLMNYINAKNV
ApisOR51	MDIRNEKNHVFNIRLAKLTGLYQMLDPGTTKFLGRNVYQMFVALFLLYLLVSAVALMVGCLHLWTYDTSMSILDFFLAINSFYACYKMCIIFYRSDDIWECLSITRYGFTSSSLRKRNGHGDVLDRWRARSVWYTSSMAGAYCFSFVFYIRCHLIFGDAIIPVKNLDGLIGNYRWNVLNLSFLTSDETYNEHYNTFFVIEALFIVVITIFYLIFDILFLTLCMAICCQMQMICDAFKSVNHKSLGDPHSSAIDNTDEKQIITSERDLIHDELITNIINHQAVIKKFELFLTIFERVMLSQIFVSSISLIILGFNLIMSFFNDGTFSISGDTTVKTIVAIPSFLFQIFMACYLFENIHDQKDSIKFALYSSNWTEMDMKCKQLILLTMQLNNANQKKLRFSRTKIVNMEMFFKTMGHCYSVLSVLINYMNAKND
ApisOR52	MDIWNENNHVFNIRLAKLIGLFQILNPGSIKFLGRNVYHIVVAINMLFVCIVAMVFFASGVYYWSDGVLVGVDYGWKGITALFLTYKMWKVVYHSNDIWDCLTITRYDFTSQNLRDRQILDRWRERSVWITNTMAIAYLMSLVILLSGSLMFRHDTLTVKNHDGSVGNYRQNIMNLYFIVTDETYNAHYKTFYFIEMLFTVGGGTLFTAFDVLLVT

	LCLAISCQFQVVNAKFESVGYKSLCDSRTKISDNKDEKQNISNEHDLIYDELISIIKDHQEVIKKYYELLTIFKRL MLLHVFYSSISLIVIWFIFIMSFTTEDRFFAWEVTTMKIICLIPSFSFHLYMTCSLFDNLHKQKDSIIFALYSSNWTE MNMKCKKLILLTMKMNNANQKKLKFTRTKIVNLELFYNIIRSCYNVVWFLINYIKVKYEL
ApisOR53	MDVWDKNNHVFNIRLAKLTGLFQVLSPESIKFLGQNVYHIVVTVILLYMGIISMILIVSGLYYWADNILLSVDY GWKGITALFSTYKMWNVVYHSNGIWDCLAITRYDFTSHGLRNRHILDLWRERSVWITNTLTIIYVSTTVLFAG SSLMFHDNISTVKNHDGSVGNYHQNLFNLYLIVTDETYNAHYETFYFIETLFAVGLATLFIAFDLLLVTLCLTV SCQMQVVNVAFESVGNKPLNDPHTPSIDNADEKKNISNEHDVIYDELISIIMDHQAVMKKYNDLLRRFKRP MLLQVFYSSTSLIVIWFCFLMSFSTEDRFAASEVTTIKIICSIPSISFQLFLVCSLFDNLHKQKDAIIFALYSSNWTE MNMKCKKLILLTMKMNNANQKKLKFTRTKIVNLELFCIIIHNCYSVVSVLIKCIKVKYE
ApisOR54	MDIRDDQNHVFNVTLAKLIGVYQTLDPKTTKYRGINVHRIVMAFIILYIGVTAVILTLSGAYYWTNNMPLSVD CYWKGIVSYTMCYSMWLIVHYSNDIWNCLSITCYGLTSNSLRDRHILDGWRELSVLITTILTFVYFMSAIIFYISS LALSNDLLPVKNHEGLVRNYRYNLFNLYLFVSEETYNVHYNIFYMVEALGVVSLLISFFVFDILLVTFCLAITC QMQMICAAFESVGHKSLANDLSSIDSRDEKKEITNKHDLIYDELKTIIMDHQEVMKKYDMFLTLFKRVLLIQ MVVFSVAFIITWFCFIMSFSNEERFKSPTIFIIKIFCGIPPNVFKLFATCYLFEKLHNQKNSIVFALYSSNWTEMDM KCKKLILLTIKMNNANYKKLKFTTTKIVSLEMFFKTMRDSYSVLSVLINYIKNKDESI
ApisOR64	MDIRNETNHVFNIKLAKLIGLYQMLDPGAAKCRGRNIYHIGMACVLLYMCLVLMILVISGLYYWTVNVPISM DYFWKSESTLYVIYKMWFVVHHSDDIWNCLSITRYDFTSFSNRNRHVLDRWRERVSWSTTIYAIIYFTTCVSYL AITLAFSEVKSPVKNHDGSIGYYRQNAMNLYLIVSDDTYNAHFYTFYFVEALFGNLIGLFFFIFDFLLVTLCFS MCCQMQIVCSAFESVGHISLRDHHPPIDYTDENIKISPDEHELIYNELKTIIKDHQAVMEKYKDFLSLFRRVML LHIFVSSLLVIAIWNTFIMSFSDDDRFQTSDVIVKKMFCVIPSILFQIYMVCYLFGNIHNQKDSIIFALYSSNWTE MDMKCKKLILLTMKMNNANQKKLKFTRTKIVNLEMFYKTMGDCYTVISVLVNYI
ApisOR67	MQNSDYLSFEYNVLQAIGVFPSTKWTKWTQRVFNFYRAIFFIFLALITFLMTVQMFIATDLTLLARTIDIWTMF FTGLYKWFYMTMFSGEFSKLKTALTQIQTQGSAAYGRSADAFTATYLKQTRKISSWYLFSGMVAASFIIVSPLLT YPKGDRSYFQYYNDPRSYPLTCWLPFELNENWIFLVVFVCQSIALVFVVNIYLGIDAYLFGAIYAVGGQIELLN TSLNNNEIILAQFENSSCTNQISMYTEKQQIRCYSTLRECVKHHILILDYITNIRKLFSTLIIMDYLHGITSVTFAL FQSTISASIIETISVICFICISIWHQYLNNFFGEFIIQKQLSVCTLSYNVPWWRCDKRIRQLLALMILRSIKPTFITGF YMYKLSYESFISFIKALYTYYMVLRRVNVKDKNA
ApisOR69	MDFRNEKNHFFNIKIAKLTALYQMLDPETIKFRGRNIYHIVTACVLVYMCLISMILLLSGVYYWTGNIPISMDY FWKSVSAFYIIYKTWIIIRNSNDIWNCLSITRYDFTSFSDRNRHILDRWRDRLTWFTTIYATMYFTAAVTYLAITL AFGENKSSVKSHDGSIGYYRQNVMNLYLIVSDETYNAHYYIFYFIEALFAAFIGLFFFIFDFLLVTLCFSMCCQM QIICSAFESVGHKSLRDQYSPIVENIKISPKEHDLIYDELKKIIMDHQLVMKKYEDFLKLFRRVMLLHIFVSSLSV ILLCFTLIMSFSNDERFKTSEIILKKLFCLIPSILFQIYMVCYLFGNIHDQKDEIIFALYSSNWTKMDMKCKKLILL TMKLNNANHKKLKFTRTKIVNLEMFFKTMGHCYTVISVLVNYISTKDE
ApisOR70	MIVDRLLKDILCCYLFLVFQQYIDIITIDIRNETNHVFNIKLAKLLGLYQILDPGALKCRGRNIYHIVTSCLLLYA CLISTILIISGLYYCTNIPVSIDYFWKSVTTIYVIYKTWIIIHYSNDVWNCLSITRYDLTSLTDRNRHILDRWRERLA WLTNIYVIMYCMTLVLYLVITLAFSEVKSTVKNRDGSVGYYRQNALNLYLIATDDTYNVHYYTFYFIEASFVAF ITLYFLIFDVLMVTLCFGMCCQMEIICSAFKSVGHKFVTDPHSPIDDIKNQTSNEHDLIYDELKTIIMDHQVVM KKYEDFLTIFRRVMLLHIFVSSFTVILLWFTFIMSFSNDDRFKTSDVIIIRMICEIPSILFQIYMMCYLFGNINDQK DEIIFALYSSNWTEMDMKCKKLILLTMQLNNANQIKLKFTRTKIVNLEMFFKTMGHCYTVISVLVNHIQTKN E
ApisOR72	MDIRNETNHVFNIKLAKLLGLYQILDPVALKFRGRNIYHIVTSCLLLYACLISTILILSGLYYCTNIPVIMDYFWK SVSTIYTIYKMWIIIHYLNDVWNCLSITRYDLTSLTDRNRHILDRWRERLAWLTNIYATTYFTTLVIYFVITLAFSE GKLTVKNRDGSVGYYRQNIMNLYLIASDDTYNAHYYTFYIIEASFIVFMTFYFLIFDILLLTLCFGMCCQMEIIC SAFKSVGHKSLCDHHSPINDIKNQTSNVHDLIYDELKTIIMDHQVVMKKYEDFLTLFRRVMLLQIFVSSFSVIL

	LWFTFIMSFFNDDRFKTSEVMVTRMFCLIPSTLFQIYIMCYLFGNLYDQKDEIIFALYSSNWTEMDMKCKKLILL<br>TMQLNNANQIKLKFTRTKIVNLEMFFKTMGHCYTVISVLVNYIKTKNE
ApisOR77	MDIQNMKNHIFNVKLARLTGLYQMLDPDTIKCRGRNIYHVVMSCVLLYMCLISMILMISGLYYWMVNVPISI<br>DYFWKSVSTFYIIYKTWIIIRHSNDIWNCLSITRHDFTALTDQNRHILERWRKRLAWLTTIYAIMYTMSVVSYLV<br>FTLAFNEGKTPVKNHDGSIGYYRQNVMNLYLIVSDETYNAHYYTFYFIEALFLGLIGLFYLIFDILLVTLCFGM<br>CCQMQIICSAFESVGHKSVRDPHFPIDYTNGNTNITPNEHDLIWDELRTIIMDHQAVMENFKYNFFYFFFSINI<br>NKMYKLFSCLNCIKFSTLIANSFSNDYRFKTSEVIVKKMFCSIPPILFQIFMVCYLFGNLHEQKDSIIFALYSSNW<br>TEMDMKCKKLILLTMKLNNANQKKLKFTRTKIVNLEMFFKTMGDCYTVISVLVNYIQTKSE
ApisOR78	MDIQNMKNHIFNIKLARLTGLYQMLDPDTIKCRGRNIYHVVMSCVLLYMCLISMILMISGVYYWTVNIPISID<br>YFWKSVSTFYTIYKTSIIIRHSNDIWNCLSITRLDFTTFSNRNRQVLDRWRERLSWLTTIYAIIYTMSVVSYLVFTL<br>VFNEGKTPVKNHDGSIGYYRQNVMNFYLMVSDETYNAHYYKFYFIEALFAAFMGFFFFIFDFLLVTLCFSMC<br>CQMRIVCSAFESVGHKSVRDPQSPIDEHDLIWDELRTIIMDHQAIMEKYKDFLSLFRRVMLAHIFISSLSVIAL<br>WFTFIMSFSNNDRFKTSELIVRKMFCAIPTILFQIFMVCYMFGKLHEQKDSIIFALYSSNWTEMDMKCKKLLLL<br>TMKLNNANQKKLKFTRTRIVNMEMFFKTMGNCYTVISVLVNQIIKQNE

Table S5.

A percent identity matrix of MperORs.

	Mper ORco	Mper OR2	Mper OR3	Mper OR4	Mper OR5	Mper OR9	Mper OR10	Mper OR17	Mper OR20	Mper OR21	Mper OR22	Mper OR23	Mper OR24	Mper OR25	Mper OR29	Mper OR35	Mper OR36	Mper OR37	Mper OR38	Mper OR39	Mper OR40	Mper OR41	Mper OR42	Mper OR43	Mper OR44	Mper OR45	Mper OR47	Mper OR51	Mper OR52	Mper OR64	Mper OR67	Mper OR69
MperOR2	14																															
MperOR3	14	16																														
MperOR4	13	11	15																													
MperOR5	10	16	15	14																												
MperOR9	10	17	15	11	46																											
MperOR10	11	14	15	13	43	68																										
MperOR17	12	15	13	14	11	13	13																									
MperOR20	11	15	12	12	10	10	12	50																								
MperOR21	10	15	11	13	12	11	10	49	57																							
MperOR22	11	14	9	11	13	11	11	47	54	61																						
MperOR23	11	14	11	9	12	12	11	25	26	26	26																					
MperOR24	11	12	10	8	10	11	10	26	25	24	24	43																				
MperOR25	10	13	9	8	12	11	12	25	24	25	23	39	37																			
MperOR29	13	13	11	11	12	11	12	26	26	26	25	37	36	49																		
MperOR35	11	15	15	12	12	11	11	21	24	23	23	26	25	25	26																	
MperOR36	12	14	13	11	14	13	13	20	21	22	22	29	26	23	26	60																
MperOR37	14	14	14	12	14	12	11	24	25	25	24	28	26	26	26	43	39															
MperOR38	12	14	14	10	14	14	11	22	19	20	22	26	24	23	24	41	38	42														
MperOR39	13	14	13	11	10	10	11	24	26	26	24	27	26	25	24	33	34	39	34													
MperOR40	10	12	12	12	14	14	13	24	24	24	26	26	24	22	24	26	26	29	28	30												
MperOR41	10	15	14	10	11	12	11	22	21	21	23	26	22	21	24	26	26	28	26	27	46											
MperOR42	12	10	12	12	12	13	14	29	26	26	26	30	28	28	30	21	24	24	21	26	22	20										
MperOR43	12	11	12	14	12	13	12	28	25	25	24	30	30	30	31	25	26	27	28	25	27	24	32									
MperOR44	12	11	14	12	14	13	13	27	22	24	24	27	25	28	28	24	23	24	23	26	23	22	29	38								
MperOR45	13	9	12	14	14	11	12	27	22	24	23	24	24	25	24	21	21	22	22	24	21	21	27	35	68							
MperOR47	14	12	14	12	15	16	14	26	24	23	23	28	23	28	27	21	22	25	26	26	23	21	28	36	38	39						
MperOR51	13	11	12	14	15	16	14	29	25	25	25	28	25	28	29	23	25	27	24	25	23	21	31	39	40	40	56					
MperOR52	11	11	14	14	17	17	16	26	22	23	23	28	25	28	27	25	26	25	25	25	24	22	28	36	39	40	53	58				
MperOR64	13	11	12	13	15	17	15	29	25	25	26	29	25	29	30	23	25	28	24	24	23	21	31	38	42	40	55	88	57			
MperOR67	14	10	13	14	11	9	8	13	9	13	12	11	9	12	12	13	13	12	12	12	10	11	14	13	14	12	13	13	13	14		
MperOR69	12	12	10	11	13	14	12	26	22	24	26	26	25	26	30	24	22	23	25	24	26	19	29	31	37	35	50	56	48	55	11	
MperOR78	12	13	12	14	15	17	15	28	26	27	26	29	24	29	30	24	26	26	26	28	24	23	29	37	41	41	56	66	54	65	11	52

Table S6.

A percent identity matrix between MperORs and DmelORs.

	Mper ORco	Mper OR2	Mper OR3	Mper OR4	Mper OR5	Mper OR9	Mper OR10	Mper OR17	Mper OR20	Mper OR21	Mper OR22	Mper OR23	Mper OR24	Mper OR25	Mper OR29	Mper OR35	Mper OR36	Mper OR37	Mper OR38	Mper OR39	Mper OR40	Mper OR41	Mper OR42	Mper OR43	Mper OR44	Mper OR45	Mper OR47	Mper OR51	Mper OR53	Mper OR64	Mper OR67	Mper OR69	Mper OR78
DmelORco	55	11	12	9	11	12	11	10	9	8	8	10	9	12	10	10	10	9	12	8	12	12	12	11	11	11	9	9	12	8	8	9	12
DmelOR1a	11	14	12	10	11	10	10	12	12	14	12	10	10	13	10	12	11	12	14	12	11	13	12	11	12	11	11	12	11	13	12	11	12
DmelOR2a	13	12	10	11	12	11	10	14	14	14	13	13	11	14	14	11	12	14	11	13	12	14	11	14	12	11	14	14	15	13	13	12	13
DmelOR7a	12	11	12	9	10	12	11	11	12	12	11	11	11	12	12	11	12	11	11	13	11	12	11	12	9	10	11	12	13	12	13	10	14
DmelOR9a	13	11	13	10	9	10	11	14	12	13	11	16	13	11	12	12	12	12	11	12	10	10	10	12	10	12	11	11	13	12	12	8	12
DmelOR10a	9	12	13	12	9	10	13	11	10	10	10	12	12	15	12	13	12	12	14	11	12	12	11	12	13	13	10	12	13	11	12	11	15
DmelOR13a	13	16	13	12	10	15	14	12	17	15	14	16	13	13	11	11	14	14	12	12	11	13	14	14	13	13	13	14	15	13	13	11	12
DmelOR19a	13	12	9	11	13	12	12	12	11	12	13	13	10	12	12	9	10	14	14	12	13	11	12	13	14	13	13	14	14	13	14	13	13
DmelOR19b	11	13	12	10	13	14	14	11	13	12	14	10	12	10	11	11	10	10	12	12	13	13	11	13	11	10	12	13	12	12	14	11	11
DmelOR22a	15	14	12	12	11	13	11	14	14	13	13	13	12	12	13	16	14	14	15	14	12	12	11	15	15	11	12	13	13	13	12	12	13
DmelOR22b	16	16	11	11	11	11	11	10	13	12	11	12	11	16	13	13	12	14	13	15	12	15	12	12	11	11	12	12	15	12	12	10	15
DmelOR22c	12	11	9	11	12	10	10	10	12	9	8	6	9	10	12	9	9	11	9	6	9	8	9	12	6	7	9	7	10	8	9	9	15
DmelOR23a	14	11	10	12	12	11	10	11	12	10	10	8	7	8	9	10	9	12	10	8	10	9	10	10	8	7	10	9	11	10	10	10	8
DmelOR24a	11	12	9	9	9	11	14	10	11	11	10	12	9	12	13	10	12	12	11	12	11	11	12	10	12	11	8	10	10	10	10	8	9
DmelOR30a	13	12	11	11	11	10	9	10	10	12	11	12	14	15	12	11	12	10	11	10	13	15	12	15	13	10	10	12	14	12	12	11	11
DmelOR33a	10	12	9	12	11	11	12	11	11	11	10	11	9	10	11	12	12	10	9	9	10	10	12	10	9	10	11	11	11	10	12	11	15
DmelOR33b	9	13	14	10	10	12	12	13	12	13	11	11	14	12	12	12	11	11	12	14	11	13	11	9	12	13	9	12	13	11	11	10	10
DmelOR33c	9	13	14	10	10	11	12	13	12	13	11	11	14	12	12	11	11	11	12	14	11	13	11	9	12	13	9	12	13	11	11	10	15
DmelOR35a	11	11	15	12	11	11	14	15	15	13	12	11	13	13	13	12	15	14	12	13	14	12	13	13	13	15	11	13	15	13	11	11	13
DmelOR42a	10	12	12	14	9	8	8	11	12	12	11	12	11	9	14	13	12	12	12	11	10	12	12	11	12	11	12	12	13	12	11	12	12
DmelOR42b	13	11	12	10	13	13	14	14	12	11	12	11	12	12	12	12	11	13	14	11	11	11	13	13	15	14	12	13	12	13	14	14	12
DmelOR43a	12	12	11	12	12	10	10	15	12	12	13	11	12	11	14	15	14	12	12	12	10	11	11	13	16	15	14	14	14	14	14	13	11
DmelOR43b	11	11	11	11	9	11	10	14	13	10	12	13	14	12	13	14	15	13	12	12	10	12	13	12	12	12	13	11	12	12	12	12	11
DmelOR45a	12	12	10	12	11	11	12	15	13	13	12	11	10	12	12	12	11	11	11	11	10	9	14	13	13	10	13	12	11	12	9	12	12
DmelOR45b	10	12	10	8	12	11	11	11	12	10	10	14	11	11	14	12	14	13	10	11	12	10	10	12	13	12	14	12	13	12	12	10	9
DmelOR46a	10	11	10	10	11	11	10	13	12	11	11	13	12	11	12	12	12	12	10	14	12	12	10	13	13	12	13	11	13	11	12	10	10
DmelOR47a	13	9	10	10	12	10	10	14	13	12	11	11	15	11	13	11	12	12	10	13	10	10	11	14	14	12	12	11	13	11	12	10	18
DmelOR47b	10	12	10	12	10	11	10	13	14	12	10	12	13	11	12	10	13	11	10	10	11	11	13	14	13	10	12	11	12	12	13	10	10
DmelOR49a	10	12	10	12	12	13	12	12	11	11	11	13	12	10	13	11	14	12	10	14	10	10	11	12	13	10	12	10	11	9	12	10	11
DmelOR49b	10	9	10	10	11	12	9	10	10	11	8	12	15	12	12	10	11	12	9	11	9	10	10	13	14	11	14	12	14	12	9	13	10
DmelOR56a	10	13	13	9	12	13	12	11	11	11	11	11	12	9	9	10	11	12	10	10	11	14	10	14	13	12	14	13	14	14	13	13	10
DmelOR59a	11	13	10	11	12	12	10	15	15	13	11	12	11	8	14	11	12	11	11	12	12	10	12	11	13	11	10	10	12	10	10	11	12
DmelOR59b	12	12	12	12	12	13	11	12	14	12	10	11	11	9	12	9	10	10	9	10	12	9	11	12	10	9	10	10	10	9	10	10	10

DmelOR59c	10	13	12	10	11	12	12	12	12	12	12	12	11	12	11	11	11	15	11	10	13	14	13	11	12	10	11	12	12	12	11	11	9
DmelOR63a	13	12	11	12	12	12	11	13	15	15	14	16	14	13	15	12	12	13	12	12	15	14	14	15	13	12	12	15	14	14	14	13	14
DmelOR65a	14	11	14	10	13	14	12	12	12	13	12	11	11	12	14	11	13	12	10	11	10	13	14	14	14	12	10	14	16	14	14	12	14
DmelOR65b	12	12	13	13	10	13	13	12	13	13	12	11	13	12	13	14	14	10	11	13	13	12	14	11	12	12	13	14	12	14	15	14	12
DmelOR65c	13	12	11	10	10	12	12	11	14	14	13	12	12	10	13	12	14	11	12	12	12	11	13	11	12	13	13	14	15	14	15	12	12
DmelOR67a	12	13	13	13	7	10	10	10	10	11	11	11	15	13	13	12	11	11	10	12	8	9	9	12	11	10	11	13	11	12	11	11	11
DmelOR67b	15	14	11	10	10	10	10	15	13	13	12	12	11	12	12	13	12	11	12	10	11	10	10	14	11	11	13	13	13	14	12	13	9
DmelOR67c	12	13	11	12	10	12	13	17	12	12	12	13	13	16	16	11	14	13	12	10	12	12	13	12	13	14	14	15	14	14	15	14	10
DmelOR67d	13	14	12	12	9	12	11	16	15	15	13	14	11	13	12	12	13	17	13	10	11	8	13	12	11	12	13	15	12	14	13	12	12
DmelOR69a	12	15	10	12	11	11	10	10	12	11	11	13	11	12	11	12	12	11	12	12	11	13	11	12	11	10	11	12	9	12	10	9	8
DmelOR71a	12	13	12	9	10	8	8	11	11	10	8	12	12	12	13	9	11	10	12	9	9	9	10	13	9	8	9	12	12	13	10	10	13
DmelOR74a	9	12	14	12	11	12	11	11	11	11	11	12	12	10	10	10	9	12	9	8	12	11	11	13	8	8	8	11	9	12	9	9	9
DmelOR82a	11	10	13	14	10	10	9	12	10	10	11	10	14	11	10	10	10	13	10	9	10	11	11	11	8	7	9	10	9	10	10	11	11
DmelOR83a	12	11	12	14	9	8	8	11	12	9	10	10	14	12	10	10	10	12	9	8	9	10	11	11	10	9	8	10	8	9	10	9	11
DmelOR83c	11	14	12	11	10	12	12	12	12	13	10	14	12	14	13	13	11	15	12	16	12	14	12	14	12	12	13	12	13	12	11	12	10
DmelOR85a	12	13	11	11	11	11	9	11	11	12	10	12	11	12	13	11	12	11	10	13	14	12	12	12	11	11	11	12	12	12	12	11	14
DmelOR85b	10	15	11	13	13	13	14	11	11	13	13	12	11	10	12	10	13	12	11	13	14	13	12	11	12	11	12	14	12	13	13	14	10
DmelOR85c	12	16	10	14	10	12	12	12	10	12	10	10	13	12	11	10	9	10	10	11	14	13	12	13	13	11	11	12	12	11	14	13	13
DmelOR85d	11	16	11	13	8	11	12	12	12	13	10	11	13	11	11	11	10	9	11	12	14	12	15	12	12	11	10	12	13	12	13	12	13
DmelOR85f	12	15	12	10	9	11	12	14	12	9	11	10	10	13	11	8	8	10	12	10	15	14	13	14	11	11	10	12	11	13	12	12	12
DmelOR88a	12	14	11	14	12	13	11	11	14	12	13	14	12	11	12	11	12	11	14	11	14	13	13	14	13	12	13	14	14	14	13	12	12
DmelOR92a	12	14	11	12	14	14	12	14	13	13	12	15	13	14	14	12	11	12	12	13	13	12	12	12	13	10	11	13	13	13	15	11	13
DmelOR94a	11	15	11	10	12	10	10	11	13	13	11	11	9	12	11	8	13	11	9	10	9	10	10	11	11	10	10	11	10	12	11	10	12
DmelOR94b	12	11	9	12	9	10	9	12	14	12	12	11	11	10	10	11	11	12	11	11	11	9	12	12	12	10	12	12	12	12	12	9	10
DmelOR98a	16	12	13	10	12	10	9	12	13	12	12	12	12	12	10	10	9	12	11	11	14	13	13	10	11	9	10	11	10	11	9	9	9

Table S7.

A percent identity matrix between MperORs and AgosORs.

	Mper ORco	Mper OR2	Mper OR3	Mper OR4	Mper OR5	Mper OR9	Mper OR10	Mper OR17	Mper OR20	Mper OR21	Mper OR22	Mper OR23	Mper OR24	Mper OR25	Mper OR29	Mper OR35	Mper OR36	Mper OR37	Mper OR38	Mper OR39	Mper OR40	Mper OR41	Mper OR42	Mper OR43	Mper OR44	Mper OR45	Mper OR47	Mper OR51	Mper OR53	Mper OR64	Mper OR67	Mper OR69	Mper OR78
AgosORco	96	29	27	14	37	41	38	30	27	34	39	31	28	36	28	28	29	32	40	29	28	31	39	31	28	36	28	28	29	32	40	29	28
AgosOR2	29	87	17	16	16	16	15	15	15	13	13	13	12	12	12	12	12	11	27	33	30	29	25	22	21	21	21	20	20	20	20	20	19
AgosOR3	27	44	44	15	14	14	14	13	13	13	13	13	13	12	12	12	12	12	27	33	30	29	25	22	21	21	21	20	20	20	20	20	19
AgosOR4	14	81	17	81	16	15	15	14	14	14	14	13	13	13	13	13	27	33	30	29	25	22	21	21	21	20	20	20	20	20	19	19	19
AgosOR5	37	41	36	15	78	14	14	14	14	13	13	12	12	12	12	12	27	33	30	29	25	22	21	21	21	20	20	20	20	20	19	19	19
AgosOR6	41	60	60	54	54	60	27	33	30	29	25	22	21	27	33	30	29	25	22	21	21	21	20	20	20	20	20	19	19	19	18	18	17
AgosOR7	38	39	34	33	29	25	43	24	23	23	23	23	22	22	22	22	21	21	20	20	20	13	13	12	12	12	12	12	11	27	33	30	29
AgosOR8	30	38	32	31	27	22	22	43	22	21	28	28	27	26	19	19	18	18	17	17	15	13	13	13	12	12	12	12	12	27	33	30	29
AgosOR9	27	59	16	15	14	14	14	14	63	13	33	28	27	26	26	12	12	12	12	11	11	14	13	13	13	13	13	27	33	30	29	25	22
AgosOR18	34	54	38	37	37	36	35	39	37	54	30	29	28	28	26	26	26	11	27	33	30	29	25	22	21	21	21	20	20	20	20	20	19
AgosOR19	27	57	42	41	41	40	39	36	39	36	61	30	29	28	28	26	26	12	27	33	30	29	25	22	21	21	21	20	20	20	20	20	19
AgosOR20	27	40	40	39	38	37	36	36	36	39	36	86	30	29	28	28	26	33	30	29	25	22	21	21	21	20	20	20	20	20	19	19	19
AgosOR21	28	31	31	31	30	30	30	30	30	36	36	28	71	30	29	28	28	33	30	29	25	22	21	21	21	20	20	20	20	20	19	19	19
AgosOR23	30	38	30	28	27	26	25	24	24	30	30	23	22	72	21	21	20	25	22	21	21	21	20	20	20	20	20	19	19	19	18	18	17
AgosOR24	35	75	49	36	34	31	31	30	30	30	30	28	27	27	78	26	26	21	20	20	20	13	13	12	12	12	12	12	11	27	33	30	29
AgosOR25	25	28	28	28	27	26	26	26	26	26	26	26	26	25	25	89	24	18	17	17	15	13	13	13	12	12	12	12	12	27	33	30	29
AgosOR26	28	40	33	28	27	26	26	26	25	24	24	24	23	22	22	21	71	12	12	11	11	14	13	13	13	13	13	27	33	30	29	25	22
AgosOR27	29	38	30	29	28	28	28	28	27	27	27	26	26	26	26	24	24	86	35	26	26	26	26	26	26	25	25	25	28	42	27	27	27
AgosOR28	30	41	33	32	30	27	23	23	23	22	22	22	22	21	21	20	20	20	45	19	35	26	26	26	26	26	26	25	25	25	42	27	27
AgosOR29	26	57	45	40	35	26	26	26	26	26	26	25	25	25	25	25	23	22	22	74	35	26	26	26	26	26	26	25	25	25	28	42	27
AgosOR30	27	33	30	29	25	22	21	21	21	20	20	20	20	20	19	19	19	18	18	17	39	35	26	26	26	26	26	26	25	25	25	45	36
AgosOR31	31	38	36	28	28	27	27	26	26	26	25	25	25	25	24	24	23	23	23	23	23	46	21	40	35	26	26	26	26	26	24	36	28
AgosOR32	29	36	32	31	29	26	25	25	24	24	23	29	28	28	28	28	27	27	20	27	27	22	37	21	40	35	26	26	26	26	26	42	31
AgosOR33	24	36	34	31	30	27	25	25	25	25	24	32	30	27	23	23	23	22	21	30	27	23	23	40	21	40	35	26	26	26	26	26	24
AgosOR34	26	46	26	26	26	26	25	25	24	23	21	40	35	26	26	26	26	26	26	26	26	26	26	22	72	21	40	35	26	26	26	26	26
AgosOR35	28	42	27	27	27	27	25	25	24	22	20	29	25	22	21	21	21	21	21	21	20	21	21	22	27	66	21	40	35	26	26	26	26
AgosOR36	31	45	36	31	31	30	28	28	27	26	25	28	28	27	27	26	26	27	26	26	26	27	26	22	22	22	48	21	40	35	26	26	26
AgosOR37	24	36	34	29	29	28	27	27	27	26	26	31	29	26	25	25	24	25	25	24	24	25	25	24	25	25	24	38	37	33	25	26	31
AgosOR38	25	42	25	24	24	24	24	24	23	22	22	31	30	27	25	25	25	25	25	25	25	25	25	25	25	25	25	18	65	37	33	64	24
AgosOR39	33	45	27	26	26	25	24	24	24	22	19	26	26	26	25	25	24	25	25	24	23	25	25	24	25	25	24	24	22	72	37	46	25
AgosOR40	37	32	31	28	28	26	26	26	26	25	25	27	27	27	25	25	24	25	25	24	22	25	25	24	25	17	17	14	14	13	34	37	33
AgosOR41	31	36	36	31	31	30	29	29	29	26	26	31	31	30	28	28	27	28	28	27	26	25	28	27	28	16	16	13	12	12	22	85	37
AgosOR42	24	51	25	25	24	24	23	23	23	23	23	29	29	28	27	27	27	26	18	17	17	17	17	14	14	13	12	11	11	10	9	22	74
AgosOR43	25	26	26	26	25	25	25	25	24	24	24	24	24	24	24	24	23	22	21	20	19	19	16	13	12	12	11	11	10	8	14	23	83

AgosOR44	33	64	34	34	33	32	32	32	32	32	31	26	26	25	24	24	24	22	27	27	27	25	24	19	15	15	15	14	14	12	23	23	23
AgosOR45	37	46	45	40	37	35	34	34	33	32	32	28	28	26	26	26	26	25	28	28	28	28	24	23	18	15	15	14	14	14	24	58	24

Table S8.

A percent identity matrix between MperORs and ApisORs..

	Mper ORco	Mper OR2	Mper OR3	Mper OR4	Mper OR5	Mper OR9	Mper OR10	Mper OR17	Mper OR20	Mper OR21	Mper OR22	Mper OR23	Mper OR24	Mper OR25	Mper OR29	Mper OR35	Mper OR36	Mper OR37	Mper OR38	Mper OR39	Mper OR40	Mper OR41	Mper OR42	Mper OR43	Mper OR44	Mper OR45	Mper OR47	Mper OR51	Mper OR53	Mper OR64	Mper OR67	Mper OR69	Mper OR78
ApisOR1	96	16	14	14	11	12	11	11	11	10	12	10	9	10	11	10	11	13	12	12	10	11	12	11	11	12	14	12	10	12	14	10	11
ApisOR2	16	93	15	14	17	22	17	12	12	13	13	13	11	11	16	13	13	14	12	12	10	11	11	9	10	10	10	12	12	12	11	12	12
ApisOR3	14	14	94	16	15	13	14	13	13	13	10	10	10	10	12	10	10	11	9	11	13	8	13	13	14	13	12	14	13	12	10	10	12
ApisOR4	15	16	17	84	15	16	13	13	12	11	11	10	13	11	14	13	15	12	11	12	12	13	10	12	12	14	13	14	12	13	13	10	12
ApisOR5	11	18	14	15	87	46	42	10	10	10	11	11	10	10	12	11	10	12	11	11	14	10	10	12	14	14	12	13	14	13	11	12	13
ApisOR7	13	17	12	14	43	70	59	7	9	9	9	11	10	10	10	11	11	11	9	11	11	11	10	9	12	12	12	13	15	12	9	13	12
ApisOR8	14	18	14	16	43	69	59	11	10	11	10	11	11	12	12	12	12	13	11	11	12	12	13	9	13	11	13	14	16	15	10	13	12
ApisOR9	12	20	14	16	46	82	68	10	10	10	12	12	10	11	12	12	12	11	10	11	13	11	13	10	14	12	14	14	15	15	10	12	13
ApisOR10	12	20	14	16	45	80	67	10	9	9	12	12	10	11	12	13	12	10	10	11	13	10	13	10	14	12	14	15	15	15	11	13	13
ApisOR11	12	20	14	18	40	67	58	10	10	11	11	10	10	10	11	12	11	12	9	10	12	11	13	9	15	12	15	14	15	14	10	13	13
ApisOR12	13	18	13	17	41	68	58	10	10	9	9	10	11	9	12	11	11	10	10	11	11	11	14	9	13	10	15	13	15	13	10	13	12
ApisOR13	12	18	13	17	42	71	63	11	10	11	11	11	11	10	12	12	12	11	10	11	10	12	14	9	13	11	15	13	15	13	11	13	12
ApisOR17	10	12	13	14	11	11	11	84	52	50	48	26	25	24	26	23	21	24	21	26	26	22	29	27	26	27	25	28	27	28	14	27	26
ApisOR18	10	11	11	12	10	11	11	77	51	47	44	28	25	26	26	23	21	25	22	24	26	21	29	28	26	28	25	28	26	28	12	26	26
ApisOR20	12	14	12	12	9	10	12	50	89	58	53	25	23	24	26	23	21	24	18	26	25	21	24	24	22	21	23	25	22	25	12	22	25
ApisOR21	13	14	13	15	10	11	11	49	56	84	65	26	25	26	27	24	24	27	22	25	24	24	26	26	26	26	23	26	23	25	15	24	26
ApisOR22	11	14	11	13	11	11	11	48	54	68	80	27	24	24	26	24	24	27	23	24	26	25	29	24	24	23	23	25	22	25	12	24	26
ApisOR23	11	12	9	10	11	13	12	26	25	25	25	90	43	39	37	28	30	29	29	28	26	27	29	27	27	24	28	28	28	29	12	26	29
ApisOR25	8	12	9	10	10	11	10	25	24	25	23	38	38	80	46	26	24	26	25	27	24	22	27	30	27	23	28	29	25	30	10	26	29
ApisOR29	12	13	10	11	15	13	13	26	26	26	26	39	35	50	72	24	24	26	22	24	24	25	27	30	27	25	27	29	26	29	14	28	28
ApisOR30	13	14	10	12	13	13	12	26	24	26	26	38	36	46	67	25	25	25	23	25	25	25	26	29	28	24	26	29	27	30	12	27	28
ApisOR31	14	12	10	12	9	11	11	23	22	23	24	26	27	25	25	44	42	42	34	35	29	28	22	25	23	24	24	26	24	24	13	24	24
ApisOR32	10	11	8	12	13	11	10	23	21	22	23	28	26	26	27	50	47	41	41	32	26	26	23	26	25	23	25	24	26	25	11	23	26
ApisOR35	12	12	11	15	10	10	11	19	22	21	22	27	27	22	26	59	73	39	39	32	26	28	24	25	26	23	24	23	25	22	11	22	26
ApisOR36	12	12	10	15	11	10	10	21	22	23	22	29	27	24	26	60	75	40	40	35	28	27	24	26	25	24	25	23	26	23	10	21	25
ApisOR37	12	14	11	11	12	10	11	24	24	26	26	28	27	26	28	43	39	88	42	38	29	28	23	27	24	23	24	26	24	26	9	26	25
ApisOR38	12	12	10	12	11	11	9	24	20	22	22	26	25	24	24	40	38	42	84	34	27	28	22	29	23	22	27	25	26	25	10	22	26
ApisOR39	11	14	10	14	10	10	12	24	27	26	23	27	27	25	26	34	33	38	34	93	27	26	25	25	26	25	26	25	25	24	11	25	26
ApisOR40	11	11	12	12	12	13	12	23	25	25	25	27	23	24	25	27	27	32	28	29	86	45	22	28	23	20	22	24	24	24	10	25	24
ApisOR41	10	10	8	13	10	10	10	24	21	23	24	25	22	22	24	25	24	27	26	27	44	84	22	25	23	23	23	23	22	22	10	21	22
ApisOR42	12	10	12	11	10	13	13	29	28	25	28	29	27	26	30	21	24	26	24	28	24	24	75	31	31	29	29	30	30	30	12	31	30
ApisOR43	11	11	13	12	11	11	11	26	24	24	23	29	29	30	31	25	26	26	26	25	28	26	31	92	36	35	37	39	37	39	12	33	38
ApisOR44	11	10	11	12	12	11	11	27	22	24	24	28	27	28	28	23	24	25	24	25	25	24	29	39	65	59	39	42	40	44	14	37	41
ApisOR45	10	9	10	12	12	9	10	27	22	25	25	27	28	28	28	22	24	25	24	27	23	23	29	38	66	58	39	43	40	44	13	40	40

ApisOR47	15	10	12	14	12	14	14	26	24	23	23	28	22	28	26	23	23	24	26	27	25	23	30	36	40	41	73	56	54	55	14	50	53
ApisOR48	15	10	12	13	12	14	14	26	24	24	24	28	22	27	26	23	23	24	26	28	25	23	29	36	40	41	74	56	53	55	14	50	54
ApisOR51	13	10	12	14	12	13	12	25	24	24	24	29	23	29	26	23	24	24	24	25	24	21	31	35	38	36	74	55	53	53	14	46	53
ApisOR52	10	11	11	12	11	12	13	26	24	24	23	27	23	28	27	25	24	26	26	29	25	25	30	38	39	38	58	56	66	54	13	47	54
ApisOR53	12	12	12	11	14	13	13	26	23	24	24	28	23	28	26	25	25	25	27	27	25	24	31	38	39	39	55	58	70	56	12	49	55
ApisOR54	13	11	14	16	14	12	12	25	23	22	24	29	23	28	27	24	24	26	26	26	24	23	29	35	39	35	52	56	58	58	12	48	53
ApisOR64	12	10	13	10	12	14	15	28	25	26	27	28	23	29	29	23	26	24	22	27	25	23	32	39	45	40	57	72	58	71	12	62	64
ApisOR67	12	12	12	12	12	15	15	28	24	24	25	27	23	27	29	24	25	26	26	27	27	23	33	38	42	40	55	70	57	69	13	58	60
ApisOR69	12	13	12	12	14	16	14	28	24	24	25	29	23	29	29	25	24	26	23	26	26	24	33	38	42	40	57	69	57	69	12	59	61
ApisOR70	12	10	13	11	13	16	15	27	24	22	24	28	23	28	29	23	24	25	24	25	26	24	32	38	43	39	58	70	56	68	11	58	61
ApisOR72	12	11	12	12	13	15	14	29	24	25	26	27	23	30	30	23	24	26	24	24	24	23	31	39	44	40	57	68	58	68	11	59	60
ApisOR77	12	11	12	10	12	14	13	25	22	22	22	27	24	27	27	22	23	24	23	24	26	23	30	36	41	36	53	67	53	67	12	57	60
ApisOR78	12	11	13	11	11	13	12	28	24	24	26	28	25	28	28	24	25	25	25	26	24	23	31	38	44	41	55	69	57	69	12	60	62

Figure S1.

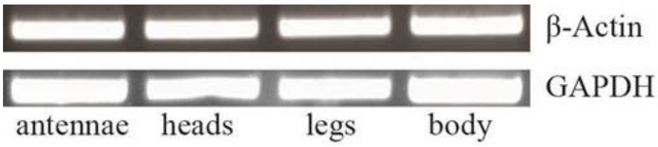

The stable expression of *M. persicae β-Actin* and *GAPDH* in different tissues measured by RT-qPCR.

Figure S2.

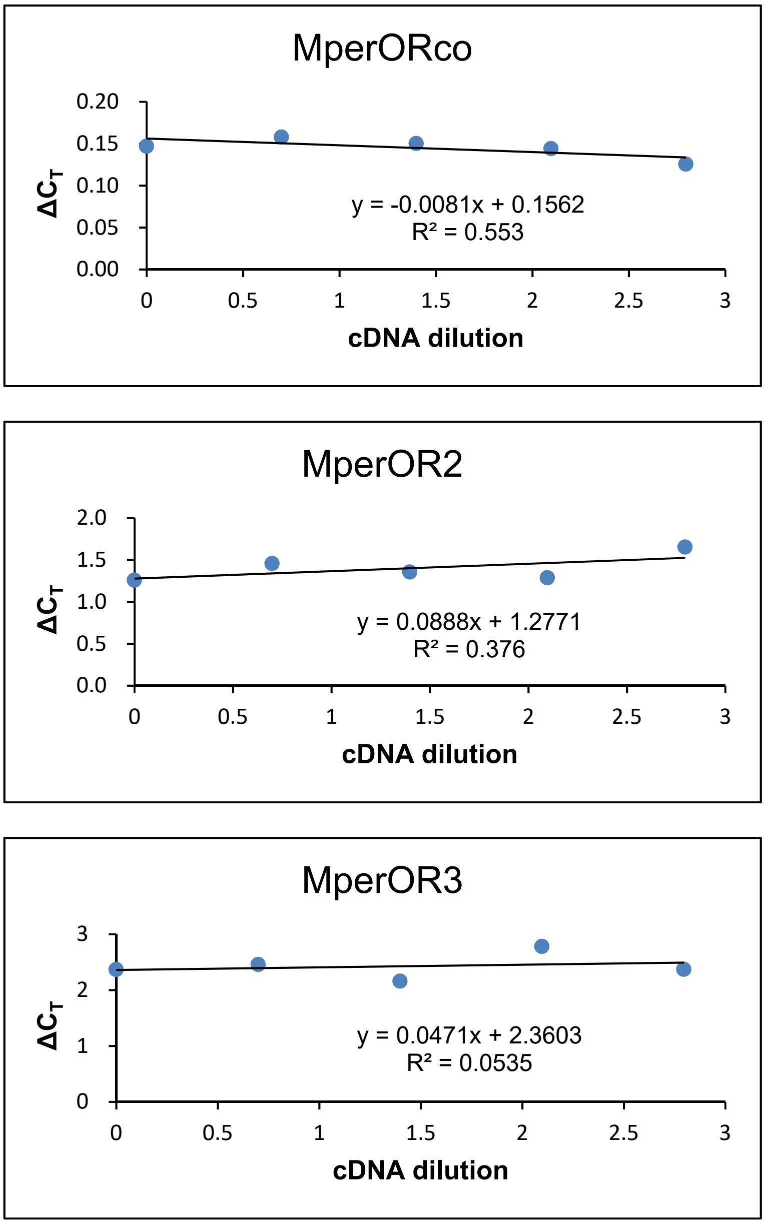

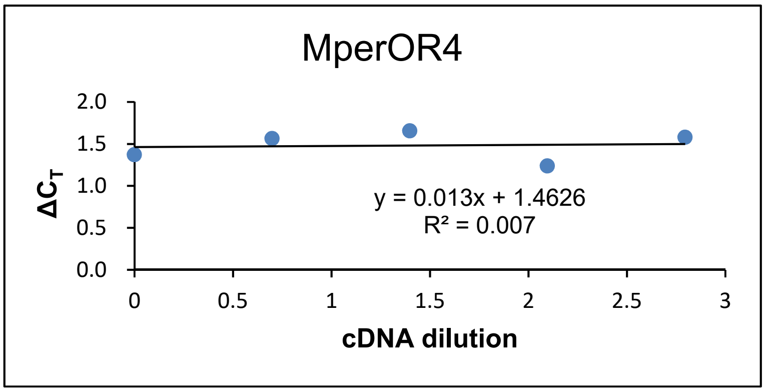

MperOR4
ΔCT
cDNA dilution
y = 0.013x + 1.4626
R² = 0.007
2.0
1.5
1.0
0.5
0.0
0
0.5
1
1.5
2
2.5
3

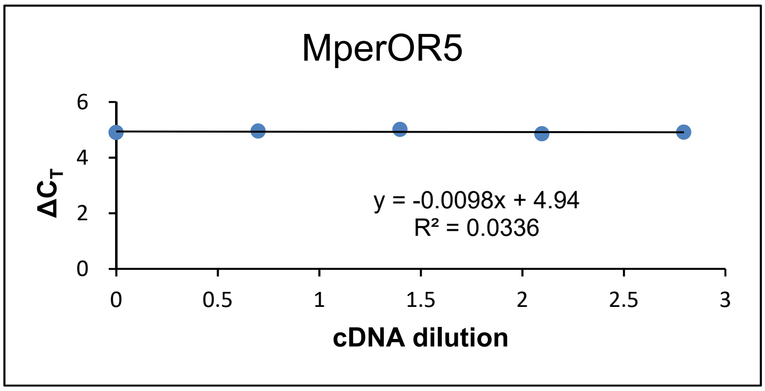

MperOR5
ΔCT
cDNA dilution
y = -0.0098x + 4.94
R² = 0.0336
6
4
2
0
0
0.5
1
1.5
2
2.5
3

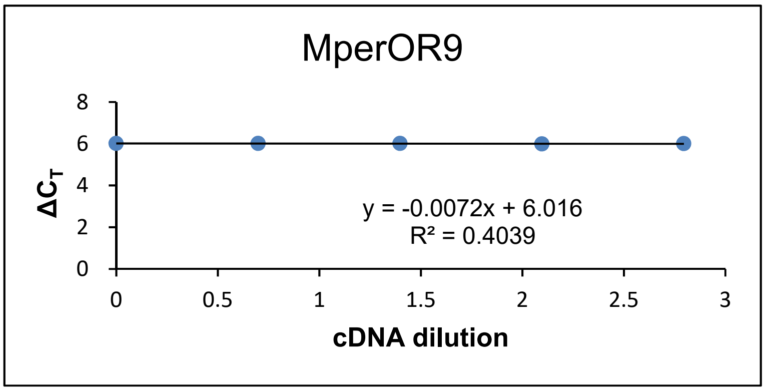

MperOR9
ΔCT
cDNA dilution
y = -0.0072x + 6.016
R² = 0.4039
8
6
4
2
0
0
0.5
1
1.5
2
2.5
3

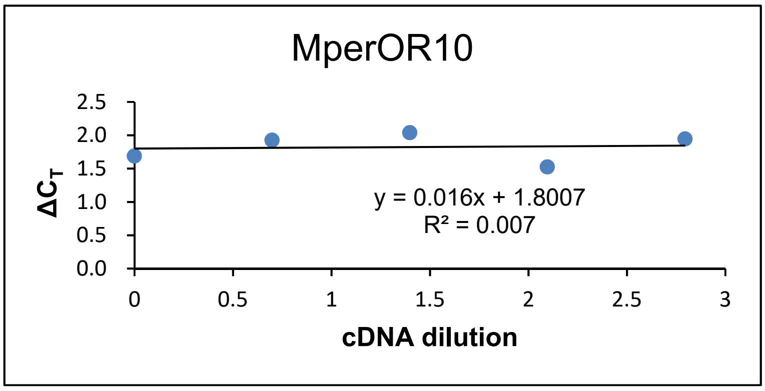

MperOR10
ΔCT
cDNA dilution
y = 0.016x + 1.8007
R² = 0.007
2.5
2.0
1.5
1.0
0.5
0.0
0
0.5
1
1.5
2
2.5
3

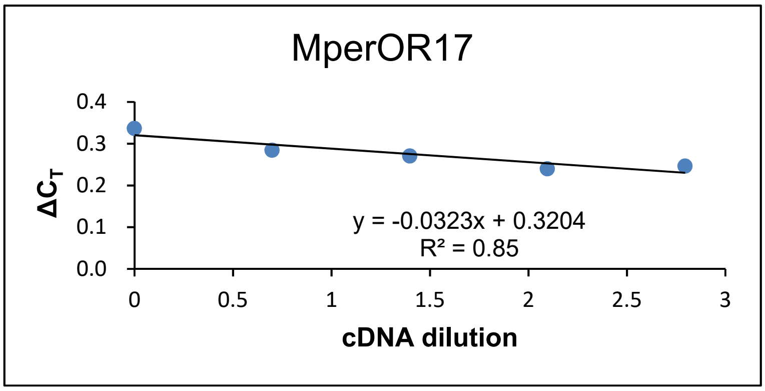
MperOR17
ΔCT
0.4
0.3
0.2
0.1
0.0
0
0.5
1
1.5
2
2.5
3
y = -0.0323x + 0.3204
R² = 0.85
cDNA dilution

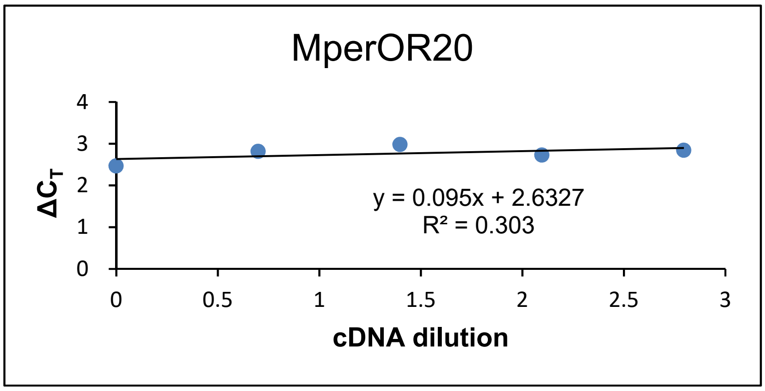
MperOR20
ΔCT
4
3
2
1
0
0
0.5
1
1.5
2
2.5
3
y = 0.095x + 2.6327
R² = 0.303
cDNA dilution

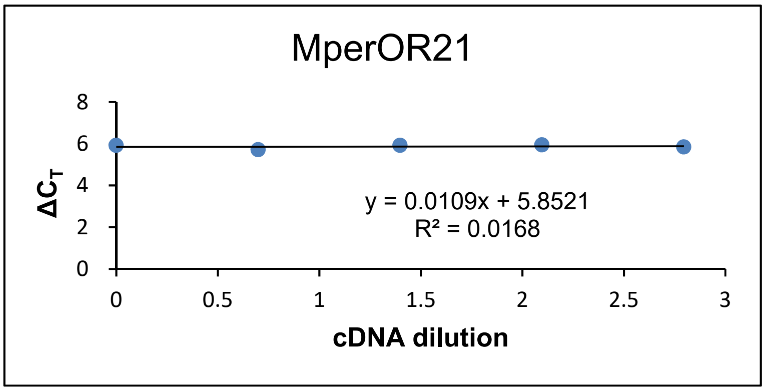
MperOR21
ΔCT
8
6
4
2
0
0
0.5
1
1.5
2
2.5
3
y = 0.0109x + 5.8521
R² = 0.0168
cDNA dilution

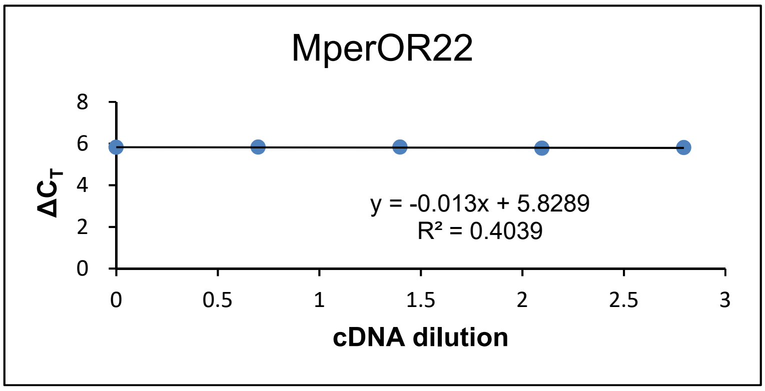
MperOR22
ΔCT
8
6
4
2
0
0
0.5
1
1.5
2
2.5
3
y = -0.013x + 5.8289
R² = 0.4039
cDNA dilution

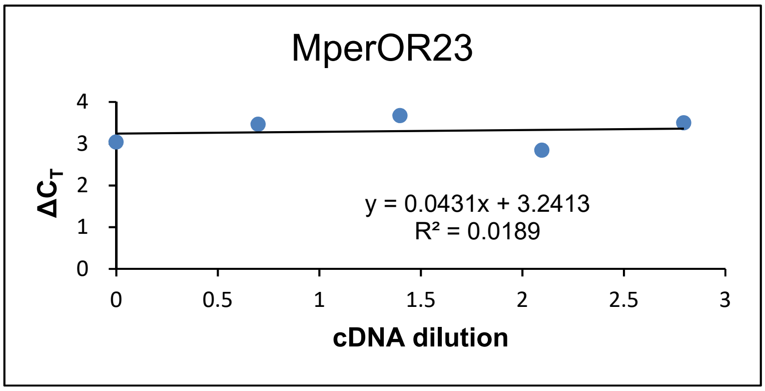
MperOR23
ΔCT
4
3
2
1
0
0
0.5
1
1.5
2
2.5
3
y = 0.0431x + 3.2413
R² = 0.0189
cDNA dilution

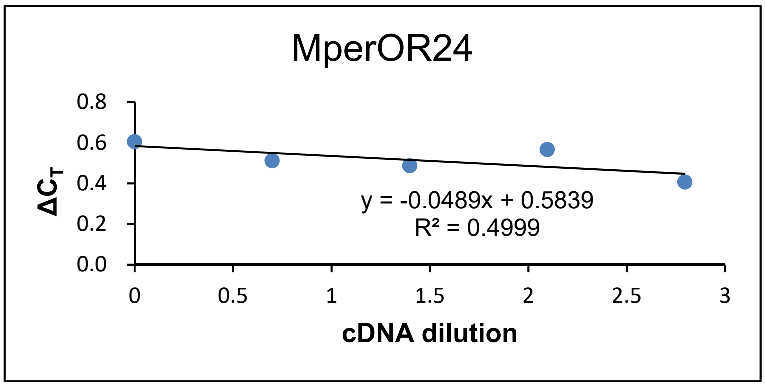
MperOR24
ΔCT
0.8
0.6
0.4
0.2
0.0
0
0.5
1
1.5
2
2.5
3
y = -0.0489x + 0.5839
R² = 0.4999
cDNA dilution

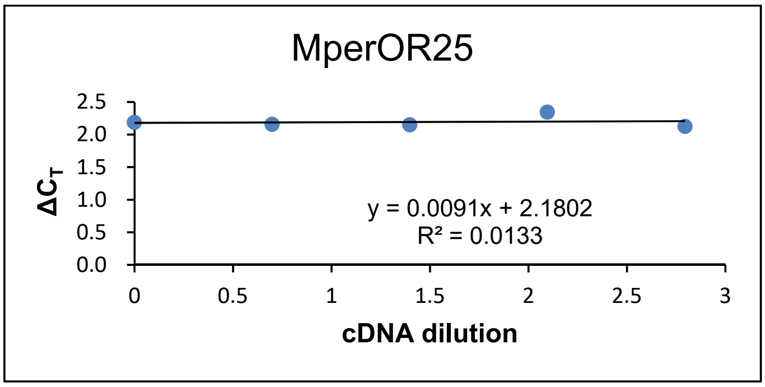
MperOR25
ΔCT
2.5
2.0
1.5
1.0
0.5
0.0
0
0.5
1
1.5
2
2.5
3
y = 0.0091x + 2.1802
R² = 0.0133
cDNA dilution

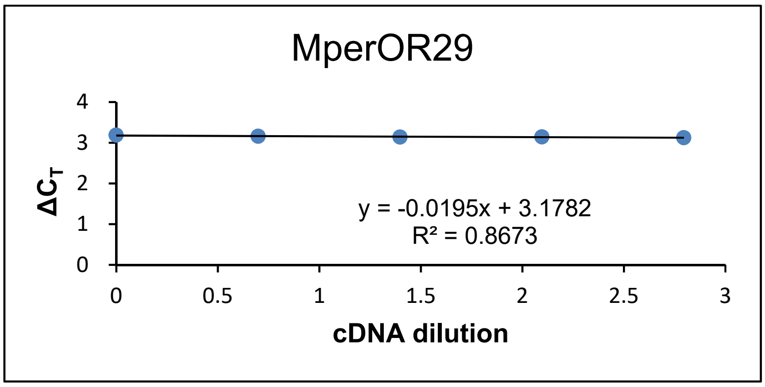
MperOR29
ΔCT
4
3
2
1
0
0
0.5
1
1.5
2
2.5
3
y = -0.0195x + 3.1782
R² = 0.8673
cDNA dilution

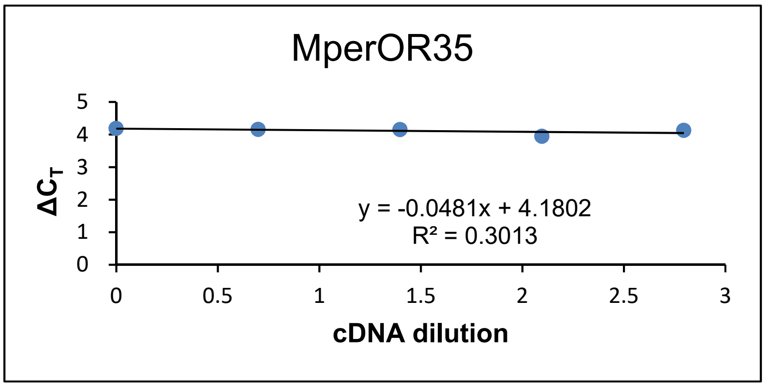

MperOR35
ΔCT
5
4
3
2
1
0
0
0.5
1
1.5
2
2.5
3
y = -0.0481x + 4.1802
R² = 0.3013
cDNA dilution

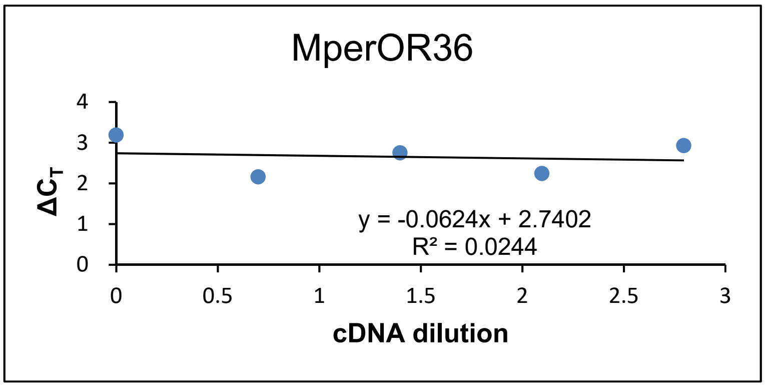

MperOR36
ΔCT
4
3
2
1
0
0
0.5
1
1.5
2
2.5
3
y = -0.0624x + 2.7402
R² = 0.0244
cDNA dilution

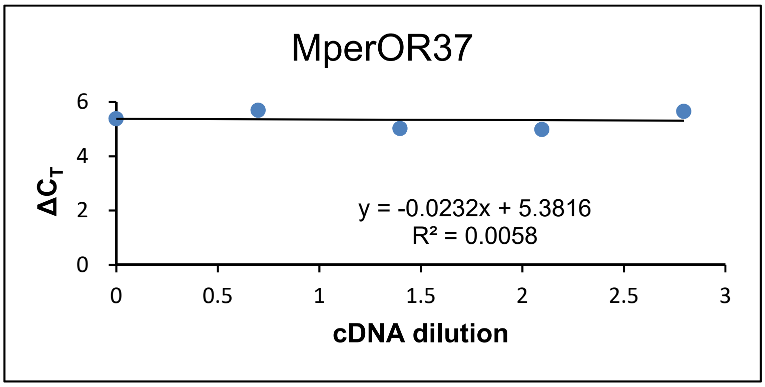

MperOR37
ΔCT
6
4
2
0
0
0.5
1
1.5
2
2.5
3
y = -0.0232x + 5.3816
R² = 0.0058
cDNA dilution

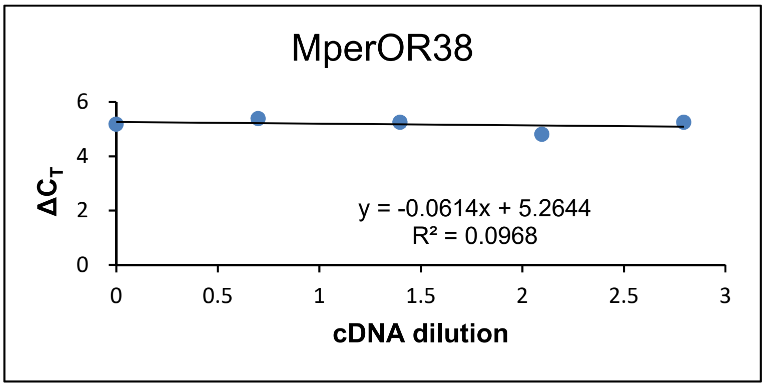

MperOR38
ΔCT
6
4
2
0
0
0.5
1
1.5
2
2.5
3
y = -0.0614x + 5.2644
R² = 0.0968
cDNA dilution

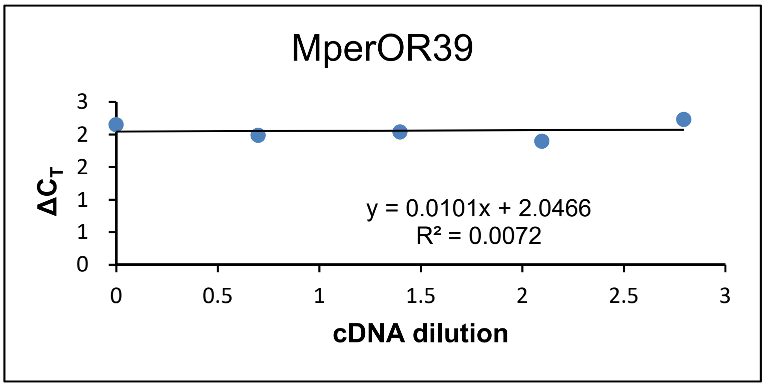
MperOR39
ΔC_T
3
2
2
1
1
0
0
0.5
1
1.5
2
2.5
3
cDNA dilution
y = 0.0101x + 2.0466
R² = 0.0072

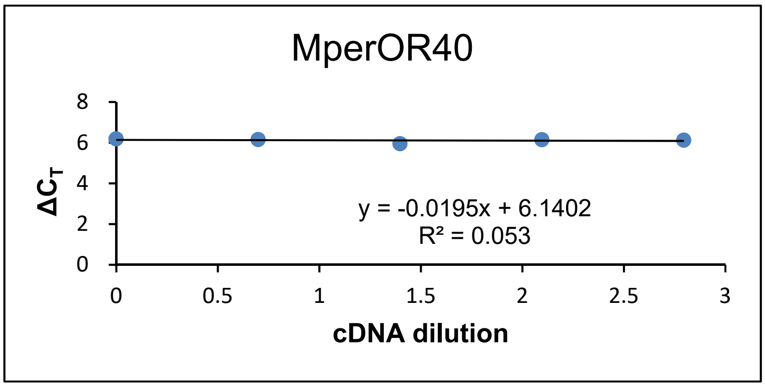
MperOR40
ΔC_T
8
6
4
2
0
0
0.5
1
1.5
2
2.5
3
cDNA dilution
y = -0.0195x + 6.1402
R² = 0.053

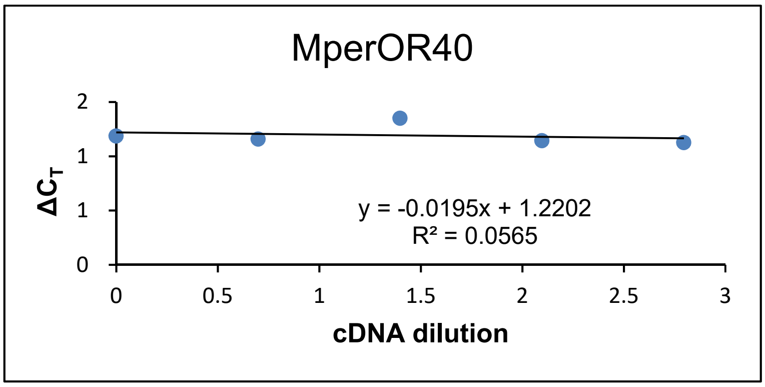
MperOR40
ΔC_T
2
1
1
0
0
0.5
1
1.5
2
2.5
3
cDNA dilution
y = -0.0195x + 1.2202
R² = 0.0565

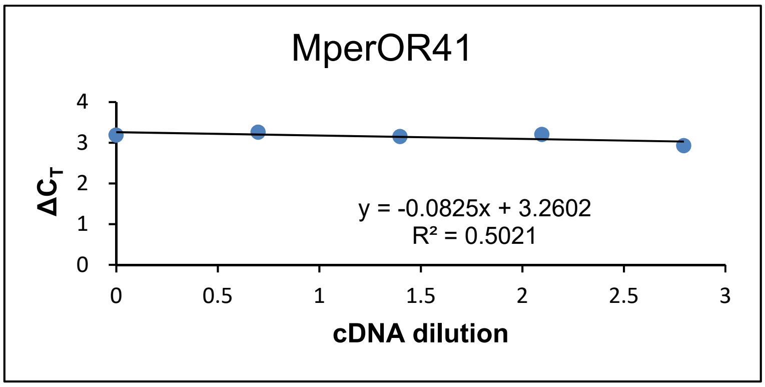
MperOR41
ΔC_T
4
3
2
1
0
0
0.5
1
1.5
2
2.5
3
cDNA dilution
y = -0.0825x + 3.2602
R² = 0.5021

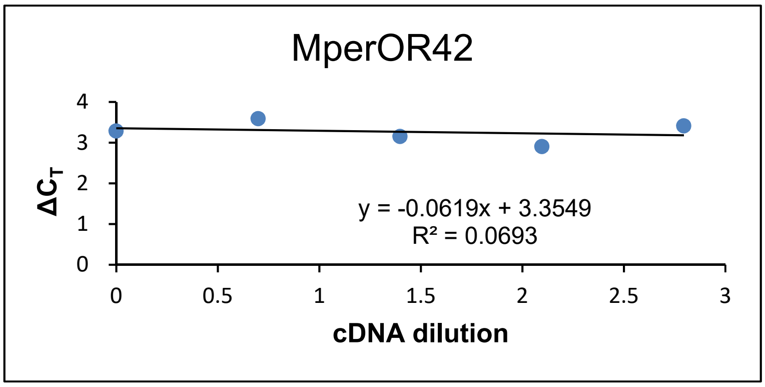
MperOR42
ΔC_T
cDNA dilution
y = -0.0619x + 3.3549
R² = 0.0693
0
0.5
1
1.5
2
2.5
3
4

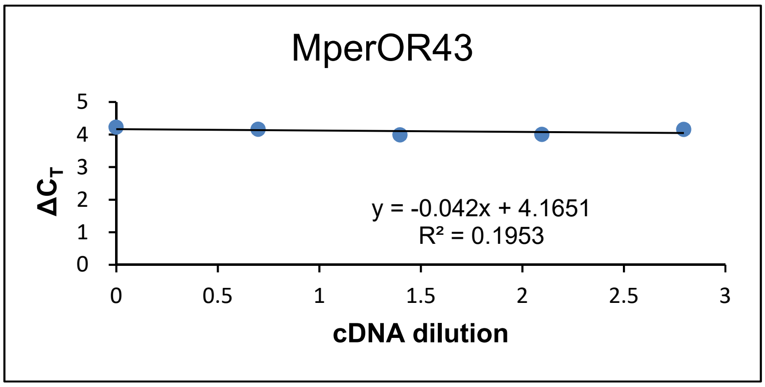
MperOR43
ΔC_T
cDNA dilution
y = -0.042x + 4.1651
R² = 0.1953
0
0.5
1
1.5
2
2.5
3
4
5

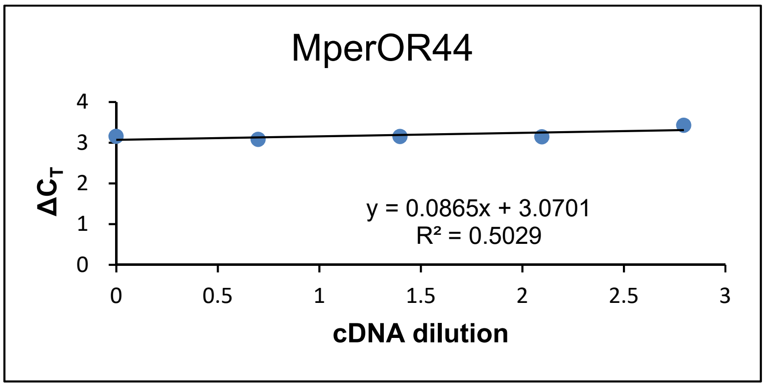
MperOR44
ΔC_T
cDNA dilution
y = 0.0865x + 3.0701
R² = 0.5029
0
0.5
1
1.5
2
2.5
3
4

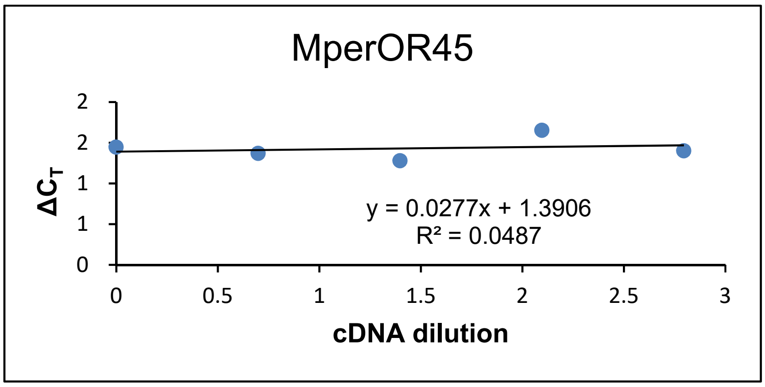
MperOR45
ΔC_T
cDNA dilution
y = 0.0277x + 1.3906
R² = 0.0487
0
0.5
1
1.5
2
2.5
3

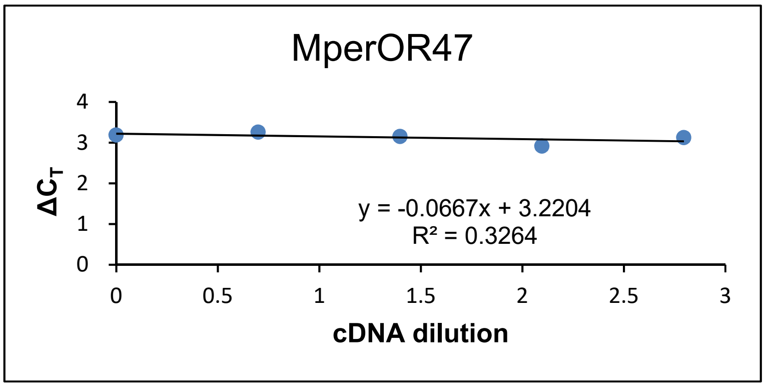
MperOR47
ΔCT
y = -0.0667x + 3.2204
R² = 0.3264
cDNA dilution

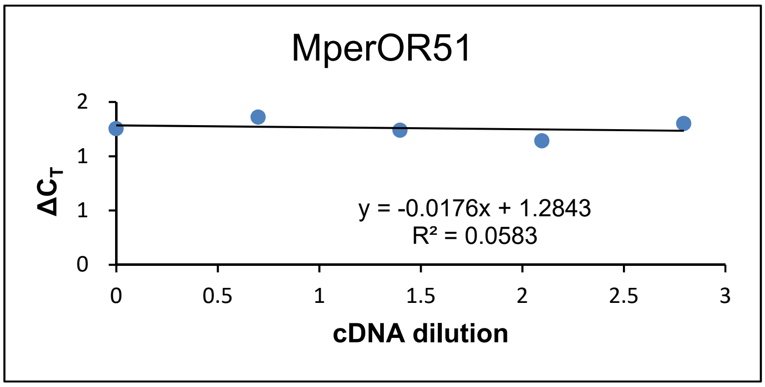
MperOR51
ΔCT
y = -0.0176x + 1.2843
R² = 0.0583
cDNA dilution

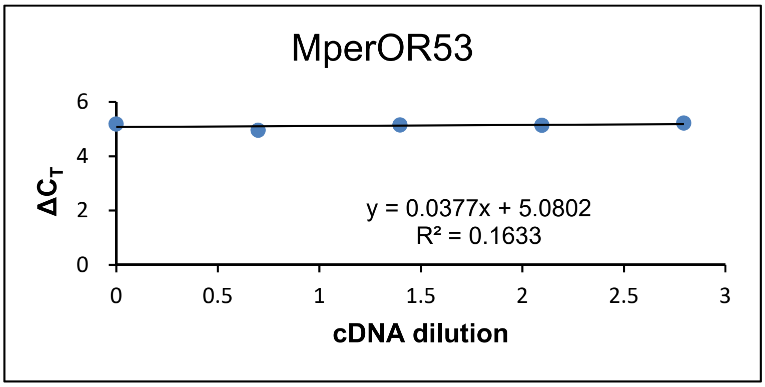
MperOR53
ΔCT
y = 0.0377x + 5.0802
R² = 0.1633
cDNA dilution

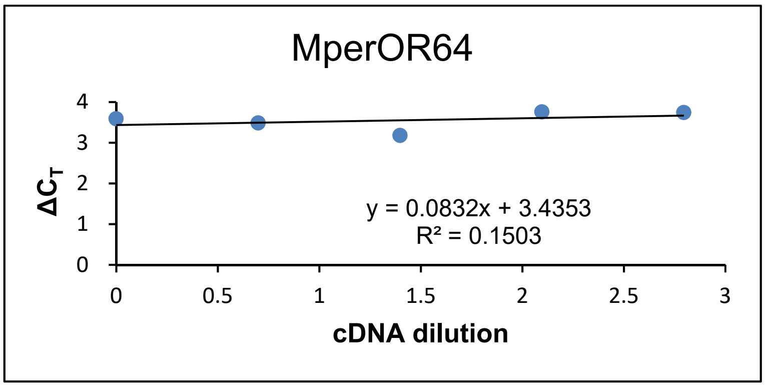
MperOR64
ΔCT
y = 0.0832x + 3.4353
R² = 0.1503
cDNA dilution

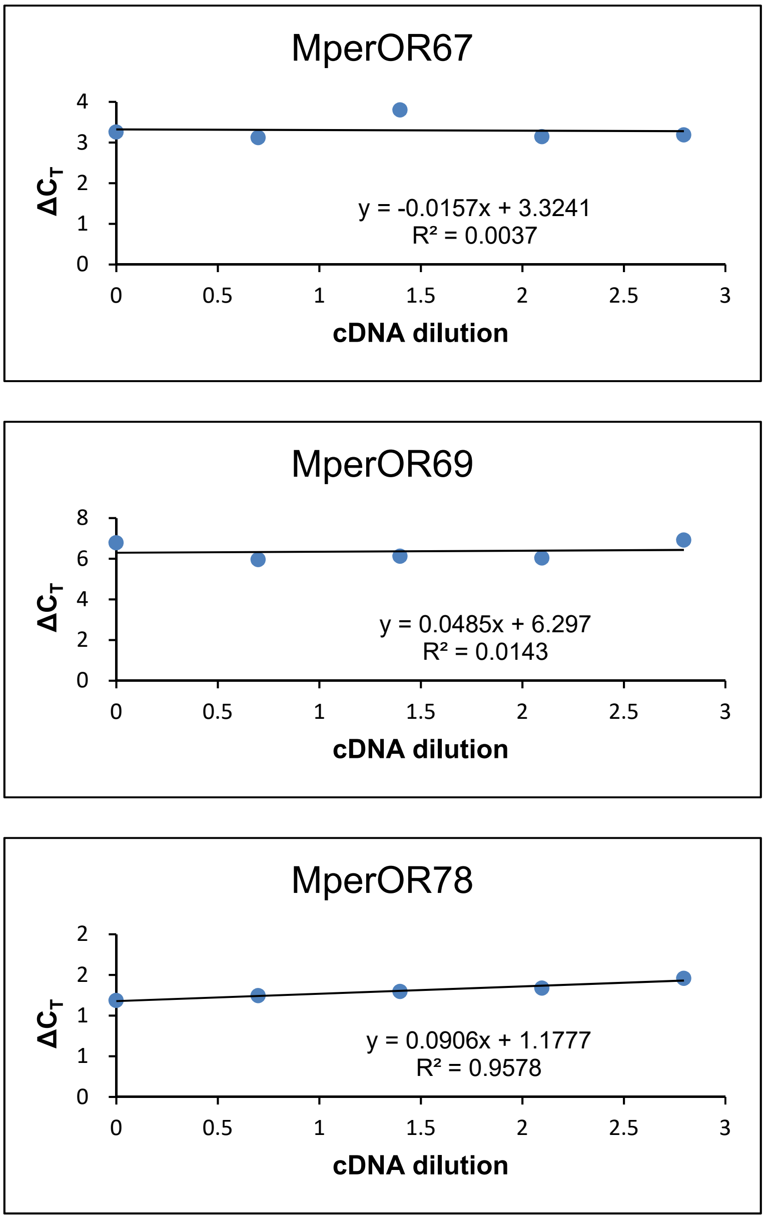

The standard curves of *MperORs* with *GAPDH* as reference gene.

Figure S3.

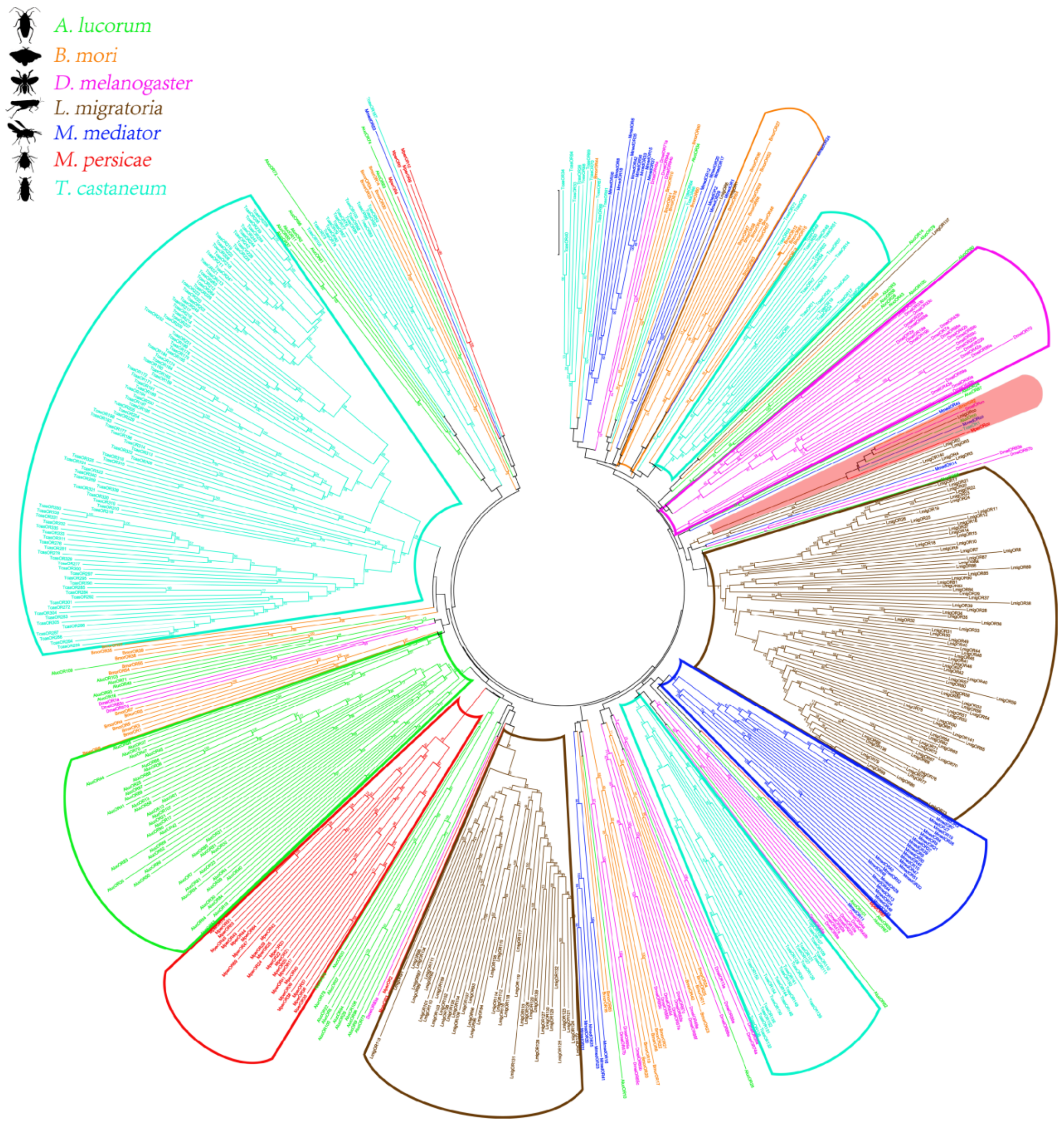

Phylogenetic tree of 606 ORs from 7 species of insects.
